# Supplementary material for: Molecular exploration of fossil eggshell uncovers hidden lineage of giant extinct bird
Source: Nat Commun. 2023 Feb 28;14:914. doi: 10.1038/s41467-023-36405-3 (PMC9974994; doi:10.1038/s41467-023-36405-3)
Supplement: Supplementary file 1 — Supplementary Information [file 41467_2023_36405_MOESM1_ESM.pdf]

Molecular exploration of fossil eggshell uncovers hidden lineage of giant extinct bird

Alicia Grealy, Gifford H Miller, Matthew Phillips, Simon J Clarke, Marilyn Fogel, Diana Patalwala, Paul Rigby, Alysia Hubbard, Beatrice Demarchi, Matthew Collins, Meaghan Mackie, Jorune Sakalauskaite, Josefin Stiller, Julia A. Clarke, Lucas J Legendre, Kristina Douglass, James Hansford, James Haile & Michael Bunce

Supplementary Material

Table of Contents

**Supplementary Note 1**	**Eggshell specimen collection**		pg 3
**Supplementary Note 2**	**Eggshell thickness**		pg 3-4
**Supplementary Note 3**	**Radiocarbon dating**		Pg 4
**Supplementary Note 4**	**Amino acid racemization**		pg 4
**Supplementary Note 5**	**aDNA analysis**		pg 4-25
5.1	aDNA extraction from eggshell		pg 4-6
5.2	Preparation of shotgun sequencing library		pg 6
		Supplementary Table 1	pg 6
5.3	Hybridisation enrichment of mitochondrial DNA		pg 7
		Supplementary Table 2	pg 7
5.4	High throughput DNA sequencing		pg 7-8
	5.4.1 Quantitation of the captured libraries and pooling		pg 7-8
	5.4.2 Size selection and purification of pooled sequencing library		pg 8
	5.4.3 Final quantitation and sequencing		pg 8
		Supplementary Table 3	pg 8
5.5	Quality control and filtering		pg 9
5.6	Reconstruction of mitochondrial genomes		pg 9-10
5.7	Authenticity		pg 10-11
		Supplementary Figure 1	pg 11
5.8	Phylogeny reconstruction		pg 11-18
	5.8.1 Alignment, gene annotation and partitioning		pg 11-13
		Supplementary Table 4	pg 13
	5.8.2 RCV analysis		pg 13-14
		Supplementary Table 5	pg 14
	5.8.3 Stemminess analysis		pg 14-17
		Supplementary Table 6	pg 16
		Supplementary Table 7	pg 16
	5.8.4 ModelTest analysis		pg 17
		Supplementary Table 8	pg 17
	5.8.5 Maximum likelihood phylogeny generation		pg 17
	5.8.6 Bayesian phylogeny generation		pg 17-18
5.9	Assessment of genetic variation in barcoding genes		pg 18-21
		Supplementary Figure 2	pg 20
5.10	Species delimitation		pg 21

		Supplementary Table 9	pg 21
5.11	Mantel test		pg 21-22
5.12	Molecular dating		pg 22-25
		Supplementary Table 10	pg 24
		Supplementary Figure 3	Pg 25
**Supplementary Note 6**	**Protein mass spectrometry**		pg 26-34
6.1	Protein extraction		pg 26
6.2	LC-MS/MS analyses		pg 26
6.3	Data Analysis		pg 26-27
6.4	Protein results		pg 27-34
		Supplementary Figure 4	pg 28
		Supplementary Figure 5	pg 29
		Supplementary Figure 6	pg 30-33
		Supplementary Figure 7	pg 34
**Supplementary Note 7**	**Characterisiation of eggshell micromorphology by microCT**		pg 35-38
		Supplementary Figure 8	pg 36
		Supplementary Table 11	pg 37
**Supplementary Note 8**	**Stable isotope analysis**		pg 38-40
		Supplementary Table 12	pg 39
		Supplementary Figure 9	pg 40
		Supplementary Figure 10	pg 40
**Supplementary Note 9**	**Body size estimation and ancestral state reconstruction**		pg 41-44
		Supplementary Figure 11	pg 43
		Supplementary Figure 12	pg 44
**Supplementary Note 10**	**Yield point value**		pg 45
**Supplementary References**	**Supplementary references**		pg 45-50

Supplementary Note 1
Eggshell specimen collection and bone identification

Aepyornithiform eggshells were collected during field seasons conducted in 2006 and 2007 with the assistance of Ramilisonina of the lnstitut de Civilizations Musée d'Art et d'Archéologie, Antananarivo, Madagascar, and Retsihisatse Analamahery, Ambovombe, Madagascar, as well as Dr. Jean-Luc Schwenninger, Oxford University, Dr. John Magee, Australian National University, Canberra, and Steve DeVogel, University of Colorado. Eggshell collecting sites were chosen based on satellite imagery. Most eggshell was found as surface exposures in recently wind-deflated sand dunes, but some were collected *in situ* in vertical exposures. AD1739 was collected *in situ* by KD and MAP team during an archaeological excavation. Samples without a field ID (Supplementary Data 2) were collected by AG, JH, KD and the MAP team in 2014, and were mostly found on the surface of rocky outcrops and beaches. Samples were placed in plastic-zip lock bags; those collected by AG and JH were collected with gloves. Two eggshell fragments from Antisirabe were donated for research by JPH. Localities sampled can be found in Supplementary Data 2.

Although five eggshell specimens thinner than 0.7 mm were collected in northern Madagascar potentially indicating the range of *Mullerornis* extended into the far north, these have not been confirmed to be ratite eggshells and were not available for molecular analysis.

The taxonomic identity of the Aepyornithid bone specimens sequenced in Yonezawa et al. (2015) was revisited in light of the framework outlined in Hansford and Turvey (2018). Tarsometatarsus 07AEP05 corresponds to the cluster of *Aepyornis maximus* according to Hansford and Turvey 2018. However, this specimen is missing over 25% of markers and therefore is an “unreliable” taxonomic assignment via morphometric clustering methods. It is of closer size to *Aepyornis maximus* size ranges reported in Hansford and Turvey 2018. Of the 23/44 measurements available this specimen fits within 16 of the measurement range of *Aepyornis maximus* and five smaller than *A. maximus* and *V. titan*, one in between the ranges and one bigger than both. However, it is important to note that this specimen appears to be still growing and many of the morphometric markers are not recorded due to the proximal surface having undeveloped features and some porosity. Distal surface of medial metatarsal has spongy porosity, distal surface of all trochlear condyles is part spongy and part fibrous, with shallow articulating grooves. Shaft surface is smooth. These growth features that correspond to stages 6-8 in Turvey and Holdaway’s (2005) growth series in *Dinornis robustus*.

Specimen 08AEP07 is a tibiotarsus. This specimen corresponds to the cluster *Vorombe titan* according to Hansford and Turvey 2018. However, the tibiotarsus clustering in Hansford and Turvey 2018 is poorly resolved.

The complex taxonomic and biogeography pattern in Aepyornithidae is brought into further question by our results. Although the number of taxa is consistent with known cranial morphotypes/ neurotypes, aepyornithid nomenclature is derived from appendicular skeletal remains, and assessments of these taxonomic units do not incorporate growth series data, have high uncertainty with highly damaged specimens (Monnier 1913) and cannot delimit tibiotarsal morphospace accurately. Southern aepyornithid skeletal remains included in this phylogeny include both a tibiotarsus (08AEP05) and a subadult tarsometatarsus (07AEP05) predicted to be *Vorombe titan* and *Aepyornis maximus,* respectively.

Supplementary Note 2
Eggshell thickness

The average thickness of each eggshell sample was calculated as the mean of the thicknesses of four sides measured with a digital caliper. The thickness of 197 elephant bird eggshells

collected from the north and 752 eggshells collected randomly from southern Madagascar (Figure 1; Supplementary Data 2) were measured to examine the distribution of eggshell thicknesses from each region. Because the south/southwest bioregion covers a large area spanning several latitudes and could comprise smaller, "cryptic" sub-regions, we plotted eggshell thickness as a function of latitude to examine whether the distribution of thicknesses varies with latitude sampled using a least squares regression modeled in PAST v3.11 (Hammer 2001). Latitude was largely uncorrelated with eggshell thickness (r^2<0.05, where r^2=1 describes a perfect correlation) i.e., a similar bimodal distribution was observed across the south. Characterising the bimodal distribution of eggshell thickness found across the south was difficult as the tail end of one distribution overlaps the other; we defined the maximum of the lower distribution to be the eggshell thickness with the lowest frequency before the frequency begins to increase again (1.42 mm), and defined the minimum of the upper distribution to be the eggshell thickness to be >1.5 mm. Summary statistics for these distributions were calculated in PAST v3.11. Based on these results, the south was treated as one bio-region for micro-CT and isotope analysis.

Supplementary Note 3
Radiocarbon dating

Eggshell samples for radiocarbon dating were mechanically cleaned then reduced by 50% with the stoichiometric addition of 2N HCl *in vacuo.* Cleaned fragments were converted to graphite at the INSTAAR Laboratory for AMS Radiocarbon Preparation and Research (NSRL) before measurement by Accelerator Mass Spectrometry at the Keck Carbon Cycle AMS Laboratory at the UC Irvine (KCCAMS). Conventional radiocarbon ages have been calibrated using CALIB v.7.1 and SHcal13 (Stuiver and Reimer, 1993; Hogg et al. 2013). Sample AD1739 was found in an archaeological deposit by KD; eggshell from the same context were radiocarbon dated as above.

Supplementary Note 4
Amino acid racemization

The utility of avian eggshell for AAR has been recognized since the work of (Brooks et al., 1990). Unlike molluscan biominerals, in which the majority of the protein residues are preserved in intercrystalline locations where they are subject to slow diffusional loss, eggshell protein is encapsulated within the calcite crystals and effectively immune from diffusional processes (Miller et al. 2000). We rely on the extent of isoleucine epimerization, in which the protein amino acid L-isoleucine racemizes about one of its two central carbon atoms to form the non-protein diastereomer D-alloisoleucine at a rate dependent on ambient temperature. The ratio of the two enantiomers (A/I) is measured by ion-exchange high-pressure liquid chromatography; A/I reflects time and the integrated thermal history experienced by the sample. Quality control is monitored with a laboratory standard, ILC-G (Wehmiller, 2013) 383 A/I analyses of the ILC-G standard in our lab average 0.457 ± 0.012. Levels of amino-acid racemisation in the eggshell samples were used to prioritise samples for aDNA extraction.

Supplementary Note 5
aDNA analysis

5.1 aDNA extraction from eggshell

Ancient DNA was extracted from eggshell samples (Supplementary Data 4) in the Trace Advanced Ultra-Clean Environment (TrACE) at Curtin University, WA (Australia). Personal protective equipment including coveralls, hairnet, double gloves, facemask, eyewear, and boots were worn to minimise the introduction of further exogenous DNA contamination. All surfaces were decontaminated with a solution of 10% bleach, followed by 70% ethanol as is

the standard protocol for contamination avoidance (Willerslev et al., 2005; Knapp et al., 2012).

The surfaces of eggshell samples were cleaned with a solution of 10% bleach, followed by 70% ethanol. The surface layer was then removed and discarded through gentle grinding at low speed with a *Dremel* drill sporting a sterile drill bit. 400 mg of eggshell was then ground into a fine powder inside a sealed, sterile, stainless-steel pot using a *Retsch* PM200 planetary ball mill at 400 rpm for 5 minutes or until completely powdered. Powder was transferred to clean 2.0-ml *Eppendorf* tubes in batches of 200 mg and stored in a -20°C freezer until extraction. Stainless steel pots were scrubbed in a solution of 10% bleach, followed by rinsing with Ultrapure water, DNAErase (*Sigma-Aldrich*) treatment for five minutes, wiping dry with 70% ethanol and UV irradiation for a minimum of one hour. Prior experiments have shown that this treatment is sufficient to avoid any cross contamination between samples powdered in the same pot at different times (data not shown).

Filter pipette tips, and certified DNase/RNase-free tubes and solutions were used throughout all DNA procedures. One DNA-free control was included for extraction alongside every 10 samples. DNA was extracted as per Dabney et al. (2013) with minor changes. Digest buffer contained final concentrations of approximately 0.25 mg/ml Proteinase K (*Astral*) and 0.45 M EDTA (*Sigma-Aldrich*) (i.e., 0.84 mg Proteinase K in 3.32 ml of 0.5 M EDTA is required per reaction). Digest buffer was inverted to mix and 1.66 ml was added to 200 mg of eggshell powder per sample in a 2-ml Safe-Lock *Eppendorf* tube. The tube was sealed tightly with Parafilm M (*Bemis NA*) and incubated for 30 minutes with rotation (10 rpm) at 55°C in a hybridisation oven. After 30 minutes, samples were centrifuged for 10 minutes at maximum speed (13,000 rpm, 17,900 x *g*) in a bench-top centrifuge (*Eppendorf*) to pellet debris. The supernatant was removed and discarded. During optimisation of the extraction protocol, this pre-digestion step (Damgaard et al. 2015) was found to reduce proportion of bacterial DNA relative to endogenous DNA without significantly decreasing endogenous DNA yield. A further 1.66 ml of digest buffer was added to the debris pellet, which was resuspended by vortexing and sealed with Parafilm. Samples were left to digest for a further 20 hours as above.

Samples were removed from the hybridisation oven and were centrifuged for 10 minutes at maximum speed (13,000 rpm, 17,900 x *g*) to pellet debris. Binding buffer was prepared with final concentrations of: 40% isopropanol, 0.05% Tween-20, 90 mM sodium acetate pH 5.2, and 5 M guanidine hydrochloride, in ultrapure water (i.e., 8.67 ml of 100% isopropanol, 10.83 µl of 100% Tween-20, 650 µl of 3 M sodium acetate pH 5.2, and 10.34 g of guanidine hydrochloride (MW 95.53 g/mol), topped up to 21.66 ml with ultra-pure water is required per sample). 1 µl of *QIAGEN* pH indicator (cat: 28004) per ml was added to the buffer to ensure that the pH remained within the DNA-binding range of the silica column. 1.66 ml of the digest supernatant was added to 21.66 ml of binding buffer in a 50 ml Falcon tube, and the solution was inverted to mix. A bleached and UV irradiated Zymo-Spin-V 15 ml extension reservoir (*Zymo Research*, cat: C1016-25) was gently but firmly fitted onto a MinElute silica spin column (*QIAGEN*, cat: 28004). Parafilm was wound around the junction between the extension reservoir and spin column to create a filter assembly that was placed inside a 50 ml Falcon tube. 10 ml of the digest-binding buffer solution was poured into the extension reservoir, and the cap was sealed with Parafilm. This assembly was centrifuged at 1,500 x *g* in a balanced bench-top centrifuge with a soft ramp for four minutes, after which the flow-through was discarded in a sealed, disposable waste container, and the remainder of the digest-binding buffer solution was poured into the extension reservoir and sealed. The assembly was centrifuged as before. The flow-through was discarded and the extension reservoir and spin column were gently disassembled. The spin column was placed in a provided 2 ml collection tube (*QIAGEN*, cat: 28004), and centrifuged for one minute at 13,000 rpm (17,900 x *g*). Flow-through was discarded, and 700 µl of Buffer PE (*QIAGEN*, cat: 28004) was added to the spin column, and the column was centrifuged for one minute at

13,000 rpm (17,900 x *g*). This step was repeated for a second time using a clean 2 ml collection tube. After discarding the flow-though, the spin column was placed in a clean 1.5 ml *Eppendorf* tube and dry spun for one minute at 13,000 rpm (17,900 x *g*). To elute the DNA, 15 µl of EB Buffer (*QIAGEN*, cat: 28004) warmed to 37°C was added directly to the membrane, the column was incubated for five minutes at 37°C, and then centrifuged at 10,000 rpm (10,640 x *g*) for one minute. This elution step was repeated such that 30 µl of eluate was obtained. The 30 µl of eluate was passed back through the column one more time after a final five-minute incubation at 37°C, and transferred to a clean 0.5 ml Lo-Bind Safe-lock *Eppendorf* tube. 1 µl of 1% TE-Tween-20 was added per 20 µl of eluate (i.e., 1.5 µl 1% TE-Tween-20) to ensure DNA did not adhere to the tube. DNA was stored at -20°C until further use.

5.2 Preparation of shotgun sequencing library

Shotgun sequencing libraries were prepared by following Gansauge and Meyer (2013), with minor changes, in an ultra-clean environment. Modifications to the adapters used are listed in Supplementary Table 1.

Supplementary Table 1 Single-stranded library build oligos used that have been modified from Gansauge and Meyer (2013).

Name	Sequence (5'-3')
CL53	ACACGACGCTCTTC/3ddC/
CL78	/5Phos/AGATCGGAAG/iSp9//iSp9//iSp9//3BioTEG/

An extraction control, no-template (water) control, and CL104 positive control were also included in the library building process. At Step 1, reactions were performed in 0.2 ml 8-well PCR strip tubes. 12 µl of DNA extract was used, and Afu UDG was replaced by ultrapure water. We elected to not use uracil-DNA glycosylase because “many aDNA researchers are reassured of the authenticity of the resulting ancient sequences when random C-T transitions are observed in cloned products of a PCR, as this form of damage is common in ancient samples” (Shapiro and Hofreiter, 2012). At Step 5, a final concentration of 2.5 U/µl CircLigase II was used (i.e., 2 µl of 100 U/µl CircLigase II). At Step 13, tubes were incubated in a thermal cycler for two min at 65°C as opposed to a thermal shaker. At Step 13, tubes were transferred to a thermal shaker (*Eppendorf*) pre-cooled to 15°C as opposed to a thermalcycler. Steps 14, 15, 18, 19, 23, and 25 were performed in a thermal shaker. At step 25, the supernatant was stored in a 1.5 ml Lo-Bind *Eppendorf* tube at -20°C. After Step 28 (in a physically separated post-PCR laboratory), 10 µl of PCR product was combined with 0.5 µl of 6X loading dye (*QIAGEN*) and run alongside 3 µl of 50 bp DNA ladder (GeneRuler, *Fermentas*) for 40 minutes at 96 V on a 2% agarose gel electrophoresis (2.2 g agarose, 110 ml 1X TAE buffer, 8 µl GelRed, 1x20-well comb) that was visualised and photographed using a *BioRad* transilluminator, in order to confirm the library building process.

At Step 30 (Gansauge and Meyer 2013), the libraries were amplified in eight replicate qPCR reactions with unique fusion-tag indexing primers suitable for the *Illumina* sequencing platforms. The PCR reaction contained reagents in final concentrations of: 1X *ABI* Power SYBR Master Mix (12.5 µl of a 2X stock), 0.4 µM *IDT* forward primer (1 µl of a 10 µM stock); 0.4 µM *IDT* reverse primer (1 µl of a 10 µM stock); 5 µl of neat library, made up to a total of 25 µl final volume with HPLC-grade water (9.5 µl). Thermal cycling conditions were: 95°C for 2 min, 20 cycles of 95°C for 15 sec, 60°C for 30 sec, 68°C for one min. Replicate reactions were combined and vortexed to mix. Reactions were purified using a *Qiagen* MinElute PCR purification kit following the manufacturer’s instructions, but eluting in 7 µl EB buffer.

5.3 Hybridisation enrichment of mitochondrial DNA

In a 0.2-ml Lo-bind PCR tube (round capped) in an ultra-clean environment, a solution was prepared (per reaction) containing final concentrations of 0.45 µg/µl Chicken Cot-1 (2.5 µl of a 1 µg/µl stock), 9.1 µM *IDT* forward blocking primer (Supplementary Table 2; 0.25 µl of a 200 µM stock), 9.1 µM *IDT* reverse blocking primer (Supplementary Table 2; 0.25 µl of a 200 µM stock). In a separate 0.2-ml tube, a solution was prepared (per reaction) containing final concentrations of 9X Hyb#1 (i.e., 9 µl of a 20X stock of Hyb#1 which is 20X SSPE), 0.0125 M Hyb#2 (i.e., 0.5 µl of a 0.5 M stock of Hyb#2 which is 0.5M EDTA, pH 8.0), 8.75X Hyb#3 (i.e., 3.5 µl of a 50X stock of Hyb#3 which is Denhardt's solution), 0.25% Hyb#4 (i.e., 0.5 µl of a 1% stock of Hyb#4, which is 10% SDS), 1 U/µl Rnase Block (*SUPERase*; i.e., 1 µl of a 20 U/ µl solution of Rnase Block), and 5.5 µl of baits for a final volume of 20 µl. Solutions were gently vortexed to mix and briefly spun in a bench-top microcentrifuge to collect the solution. In a post-PCR environment, 5 µl of the first solution was then added to 7 µl of library (in a post-PCR environment) for a total of 12 µl.

The first solution was placed in a thermalcyler and incubated for five minutes at 95°C. The second solution was then placed in the thermalcycler and both solutions were incubated for five minutes at 55°C, after which 18 µl of the second (baits) solution was added to the first (library) solution. The solution was gently vortexed to mix. 10 µl of mineral oil was added on top of the reaction to prevent evaporation, and briefly spun in a microcentrifuge to collect liquid. The solution was incubated in the thermalcycler with a heated lid for 40 hours at 55°C. The remainder of the MYbaits protocol v.3 (*MYcroarray*) was performed as per the manufacturer's instructions between steps 2A.1 and 3.1.

Supplementary Table 2 Custom blocking primers (for sequencing the NextSeq platform) used in the enrichment protocol and primers used to amplify the captured libraries.

**Name**	**Sequence (5'-3')**	**Function**
NextSeq_Blocking_F	AATGATACGGCGACCACCGAGATCTACACIIIIIIII ACACTCTTTCCCTACACGACGCTCTT /3InvdT/	Forward capture blocking primer
NextSeq_Blocking_R	CAAGCAGAAGACGGCATACGAGATIIIIIIII GTGACTGGAGTTCAGACGTGTGCTCT /3InvdT/	Reverse capture blocking primer
P5	AATGATACGGCGACCACCGAGATCTACAC	Forward library amplification primer (*Illumina*)
P7	CAAGCAGAAGACGGCATACGAGAT	Reverse library amplification primer (*Illumina*)

Captured libraries were amplified in eight replicate qPCR reactions containing final concentrations of: 1X *ABI* Power SYBR Master Mix (25 µl of a 2X stock), 0.2 µM *IDT* forward primer P5 (1 µl of a 10 µM stock; Supplementary Table 2); 0.2 µM *IDT* reverse primer P7 (1 µl of a 10 µM stock; Supplementary Table 2); 5 µl of neat library, made up to a total of 50 µl final volume with HPLC-grade water (18.5 µl). Thermal cycling conditions were: 95°C for 30 seconds, 20 cycles of 95°C for 20 sec, 60°C for 30 sec, 72°C for 30 sec. Replicate reactions were combined and vortexed to mix and purified as above.

5.4 High-throughput DNA sequencing

5.4.1 Quantitation of the captured libraries and pooling

The total DNA concentration of an aliquot of 1 µl of each captured library was quantified using a Nanodrop 2000 spectrophotometer following the manufacturer's instructions. Based on this rough quantitation, each library was diluted to between 2 and 5 ng/µl in ultrapure water for a final volume of 10 µl, in order for their concentrations to be within the dynamic range of the LabChip GX Touch HT (*Perkin Elmer*); this usually required a 1 in 10 dilution (i.e., 1 µl of sample in 9 µl of ultrapure water). 10 µl of this dilution was loaded onto the LabChip GX Touch HT for quantitation of fragments between 140 and 300 bp, following the manufacturer's instructions. Libraries were then pooled in equimolar concentrations in a total volume of 60 µl and vortexed to mix.

5.4.2 Size selection and purification of pooled sequencing library

To eliminate low-molecular weight artefacts, the 60 µl-pooled library was divided into two lots of 30 µl and run on two lanes of a Pippin Prep (*Sage Science*) ethidium bromide eGel cassette to select fragments between 140 and 300 bp, following the manufacturer's instructions. This size range was selected to capture the largest fragments representing elephant bird sequences, with the remainder of fragments below 140 representing sequencing adapters or dimer. To buffer exchange and concentrate the size-selected library, it was purified using a PCR purification kit (*QIAGEN*). After the addition of PB buffer, 3 µl of 3 M sodium acetate was added to adjust the pH. DNA was eluted in 30 µl of ultrapure water.

5.4.3 Final quantitation and sequencing

The final purified sequencing library was quantitated using a Nanodrop 2000 spectrophotometer and LabChip GX Touch HT as above.

Captured libraries were sequenced on the NextSeq next-generation sequencing platform (*Illumina*; Curtin University).

10.0 µl of the 2 nM dilution of the library was combined with 10 µl of 0.2 M molecular biology-grade NaOH and incubated for five minutes at 25°C, then placed on ice. 10 µl of 200 mM Tris-HCl was added to this mixture, and the solution was vortexed to mix and placed on ice. 970 µl of HT1 buffer was added to this mixture, vortexed, and placed on ice to make 1 ml of a 20 pM library. 97.5 µl of the 20 pM library was added to 1202.5 µl of HT1 buffer to make 1.3 ml of a 1.5 pM library. 1.2 µl of 20 pM PhiX was added to 1299 µl of the 1.5 pM library to make the final loading solution that was added to well 17 of the reagent cartridge. 6 µl of a 100 µM stock of custom sequencing primer CL72 (Supplementary Table 3) was spiked into well 20 of the reagent cartridge, and a 6 µl of a 100 µM stock of custom i5 indexing primer (Supplementary Table 3) was spiked into well 22 of the reagent cartridge.

Supplementary Table 3 Custom i5 indexing primer and read 1 sequencing primers used to sequence libraries on the NextSeq platform.

Name	Sequence (5'-3')	Function
Custom i5 indexing primer	GGAAGAGCGTCGTGTAGGGAAAGAGTGT	Custom forward index sequencing primer
CL72	ACACTCTTTCCCTACACGACGCTCTTCC	Custom Read 1 sequencing primer

The options selected when creating the sample sheet were: Other/ FASTQ only/ Sample preparation kit = TruSeq LT/ 2 Index reads/ Single end/ 150 cycles/ No custom primer/ No trimming. The library was run using a NextSeq 500 Mid-output 150 v2 kit.

5.5 Quality control and filtering

Sequencing data for each file was quality filtered in USEARCH v.8 (Edgar, 2010) using the following command:

```
usearch8 -fastq_filter [FILENAME.fastq] -fastqout [FILENAME_QF.fastq]
-fastq_truncqual 3 -fastq_maxee_rate 0.01 -fastq_minlen 35
```

Quality filtered sequence data from each sample across the four NextSeq lanes were combined using the following command:

```
cat [SAMPLE_NAME]* > [SAMPLE_NAME_QF_.CAT].fastq
```

Sequences were dereplicated using the following command:

```
usearch8 -derep_fulllength [SAMPLE_NAME_QF_CAT].fastq -fastqout
[SAMPLE_NAME_QF_CAT_DEREP].fastq –sizeout
```

Chimeric sequences were removed using the following command:

```
usearch8 -uchime_denovo [SAMPLE_NAME_QF_CAT_DEREP].fastq -
nonchimerasq [SAMPLE_NAME_QF_CAT_DEREP_CF].fastq
```

Filtered sequences were imported into Geneious v.8.1.6 (Kearse et al., 2012) and any remaining adapter sequences (that were not automatically removed by Illumina software due to sequencing errors) were trimmed from the 3' end; these were found using the 'Annotate and Predict' / Trim Ends / Remove new trimmed regions / Allow mismatches 2 / Minimum match length 9 / Trim 3' end. Sequences below 30 bp were discarded and unique sequences were found through Edit / Find Duplicates / Extract unique sequences.

5.6 Reconstruction of mitochondrial genomes

For each sample, sequences were iteratively mapped against a consensus elephant bird reference mitochondrial genome in Geneious v.8.1.6 using the default parameters under a 'medium-low sensitivity' option with 10 iterations. Mapped reads were then aligned to NCBI's GenBank reference database (Benson et al., 2006) using BLAST 2.2.30+ (Altschul et al., 1990) implemented through the Pawsey Centre's supercomputing facilities in order to obtain taxonomic assignments for the sequences. The blastn algorithm parameters evoked were as described by Grealy et al. (2017). Sequence taxonomy was assessed in MEGAN v.4.70.4. (Huson et al., 2007). LCA parameters were: Min Support 1 / Min Score 35.0 / Top Percent 10.0 / Win Score 0.0 / Min Complexity 0.44. The tree was collapsed at the order level, and sequences within the node Aves were extracted in .fasta format. To remove potential contaminating sequences, reads aligning best to avian reference genomes were remapped onto the consensus genome generated from the last round of mapping, as before.

Finally, reads with identical coordinates were collapsed using Picard Tools v.2.10.3 (http://broadinstitute.github.io/picard/): the mapping file was exported from Geneious v8.1.6 in .bam format and using a terminal window, Picard was invoked:

```
java –jar [PATH TO PICARD.JAR]/picard.jar SortSam \
INPUT=[PATH TO INPUT FOLDER]/[INPUT NAME].bam \
OUTPUT=[PATH TO OUTPUT FOLDER]/[OUTPUT NAME]_sorted.bam \
SORT_ORDER=coordinate
```

Followed by:

```
java -jar [PATH TO]/picard.jar MarkDuplicates \
INPUT=[PATH TO INPUT FOLDER]/[INPUT NAME]_sorted.bam \
OUTPUT=[PATH TO OUTPUT FOLDER]/[OUTPUT NAME]_collapsed.bam \
REMOVE_DUPLICATES=true \
METRICS_FILE=[PATH TO OUTPUT FOLDER]/[OUTPUT NAME]_metrics.txt
```

The filtered .bam file was then imported back into Geneious v8.1.6 and a final strict consensus sequence with 50% majority-ruled based calling was generated, with positions having a coverage of <2 called as an 'N' and positions with no data represented by '?'. These final mitochondrial genomes can be found in GenBank (see "Data availability statement" in the main text) or downloaded from DataDryad (see "Data availability statement" in main text). The authenticity of mapped reads was assessed by charting the frequency of nucleotide substitutions across reads in mapDamage 2.0.6 (Ginolhac et al., 2011; Jonsson et al., 2013).

5.7 Authenticity

Final mapping files were exported from Geneious v 8.1.6 as a .sam file into /mapDamage/bin, also exporting the reference sequence in .fasta format. MapDamage 2.0.06 was run by navigating to the /mapDamage/bin folder in a terminal window, and typing the command:

```
mapdamage -i [INPUT].sam -r [REFERENCE].fasta
```

Nucleotide misincorporation plots were examined for a higher proportion of C to T misincorporations at the 5' and 3' terminus of reads that is indicative of authentic ancient DNA. In the first position from the 5' end, the frequency of C to T mutations exceeded 0.3 in all samples (Supplementary Figure 1 shows a typical example), suggesting that the mapped reads were truly of ancient origin.

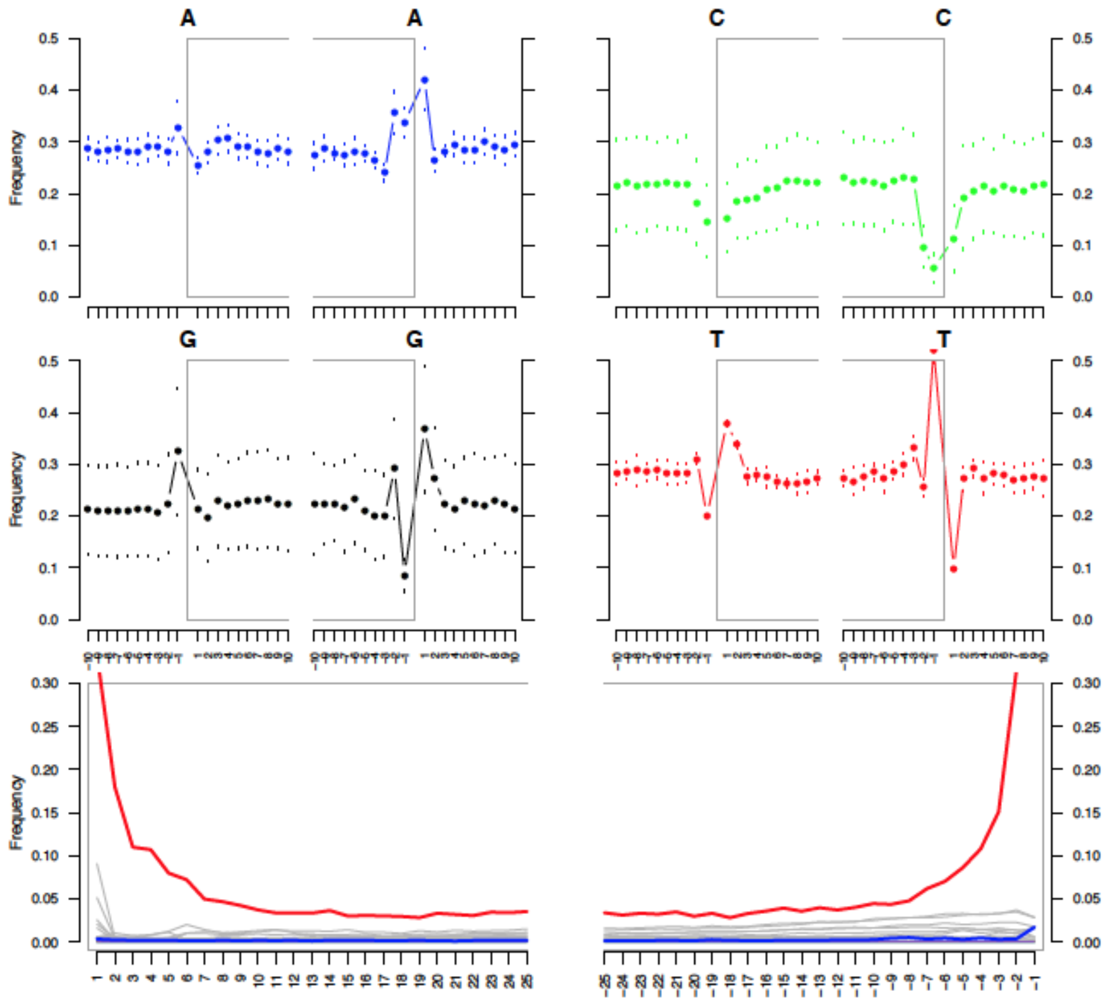

Supplementary Figure 1 Damage profile from MB2973 exemplifying a higher proportion of C to T nucleotide misincorporations (red) at both the 5’ and 3’ ends mapped reads (bottom panel). This damage pattern was observed for all that were used for subsequent analysis. Figure generated by MapDamage 2.0.6 (Jonsson et al., 2013; Ginolhac et al., 2011).

5.8 Phylogeny reconstruction

5.8.1 Alignment, gene annotation and partitioning

12 samples with more than 12,000 missing bases were excluded from downstream analysis. 21 elephant bird mitochondrial genomes were annotated by aligning them with a kiwi mitochondrial genome, and transferring the annotations from the kiwi to the elephant bird genomes in Geneious v.8.1.6 using the ‘Annotations>Copy all in selected region to’ function. Annotated genomes were then aligned with eight outgroup ratite taxa (Supplementary Table 4) and four previously published elephant bird genomes (*Aepyornis hildebrandti* KJ749824 and *Mullerornis agilis* KJ749825; (Mitchell et al. 2014), *Aepyornis maximus* AP014697 and *Mullerornis* sp. AP014698 (Yonezawa et al., 2016) using a MAFFT v. 7.308 alignment (Katoh et al., 2002) as implemented in Geneious v.8.1.6 (‘Align/Assemble>Multiple align>MAFFT alignment’) with the default parameters. The alignment was refined by using the MUSCLE v. 3.8.425 algorithm (Edgar, 2004) as implemented in Geneious v.8.1.6 with the default parameters (‘Align/Assemble>Multiple align>MUSCLE alignment’). As aDNA damage can inflate genetic diversity, potentially damaged bases were identified as any G->A or C->T transition that is present in only one sample but where the ‘wild-type’ base is otherwise fixed in *all ratites*. These positions were RY-coded. The alignment contained a

total of 32 complete mitochondrial genomes. All protein-coding genes (ATP6, ATP8, CO1, CO2, CO3, CYTB, ND1, ND2, ND3, ND4, ND4L, ND5, and ND6), rRNA genes (16SrRNA and 12SrRNA), tRNA genes (22), and the control region were individually extracted from the alignment using the 'Extract' function in Geneious v.8.1.6.

Protein coding genes were translated to ensure the ORFs were in-frame. Any termination mutations (i.e., base changes that convert an amino acid to a stop codon) were converted to RY-coding. Insertion or deletion mutations were dealt with by adding additional gaps such that the codons downstream of the mutation were still in-frame. Each protein-coding gene was partitioned by codon position in Geneious v.8.1.6 using the 'Tools>Strip alignment columns>Strip two adjacent columns per codon>Start with column 2' function (n.b.: starting with column 2 will extract 1st codon positions, starting with column 3 will extract 2nd codon positions, and starting with column 1 will extract 3rd codon positions, provided that every position in the alignment is in-frame). Codon positions for each gene were concatenated in Geneious v.8.1.6 using the 'Tools>Concatenate sequences or alignments' function, such that three files were generated, one containing the 1st codon positions for all protein-coding genes, one containing the 2nd codon positions for all protein-coding genes, and one containing the 3rd codon positions for all protein-coding genes.

The stems and loops of the *16SrRNA* gene were annotated as defined by (do Amaral et al., 2010, and were partitioned in Geneious v.8.1.6 (using the 'Extract' function, as above). The stems and loops of the 12sRNA gene were annotated as defined by (De Los Monteros, 2003), and were partitioned in Geneious v.8.1.6 as above. The secondary structure of tRNAs were predicted using the program ARWEN v1.2 (Laslett and Canback, 2008), and the stems (acceptor stem, D stem, anticodon stem, and T stem) and loops (D loop, anticodon loop, variable loop, and T loop) were partitioned as above. Loop alignments of the RNA genes were concatenated as above, as were the stem alignments of the RNA genes.

The control region for all taxa was annotated and partitioned into the three domains as defined in (Ruokonen, 2002) in Geneious v.8.1.6 using the 'Add Annotation' and 'Extract Annotation' functions. However, the control region was not used for downstream analyses.

For downstream analyses, all unknown characters marked by a '?' were designated as by 'N'.

Unstable blocks in the partitioned alignments were removed using Gblocks v0.9b (Castresana, 2000; Talavera and Castresana, 2007) (available: http://www.phylogeny.fr/one_task.cgi?task_type=gblocks&tab_index=1) using default parameters but allowing smaller final blocks and allowing gap positions within the final blocks.

Supplementary Table 4 GenBank accessions of the eight outgroup ratite taxa used for whole mitochondrial genome phylogenetic analyses.

Species name	Common name	Accession Number	Reference
Apteryx australis mantelli *APTMA*	North island brown kiwi	AY016010	(Cooper et al., 2001)
Apteryx australis mantelli *APTMA2*	North island brown kiwi	KU695537	(Liu and Gao, 2016)
Apteryx haastii *APTHA*	Great spotted kiwi	NC_002782	(Haddrath and Baker, 2001)
Apteryx owenii *APTOW*	Little spotted kiwi	NC013806	(Phillips et al., 2010)
Casuarius bennetii *CASBE*	Dwarf cassowary	AY016011	(Cooper et al., 2001)
Casuarius casuarius *CASCA*	Southern cassowary	NC_002778	(Haddrath and Baker, 2001)
Dromaius novaehollandiae *DRONO*	Emu	AY016014	(Cooper et al., 2001)
Dromaius novaehollandiae *DRONO2*	Emu	NC_002784	(Haddrath and Baker, 2001)

5.8.2 RCV analysis

Relative composition variability (RCV; Phillips et al., 2010) for each mitochondrial partition with standard nucleotide coding as well as RY coding was calculated in PAUP v.4a150 (Swofford, 2003), in order to determine which partitions may benefit from RY-coding. Partition MSAs were combined in NEXUS format, with each partition defined as a character set ('charset'):

```
charset m1 = 1-3787;
charset m2 = 3788-7573;
charset m3 = 7574-11351;
charset stems = 11352-13283;
charset loops = 13284-15175;
```

An RY-coded MSA (R = A or G; Y = C or T) in NEXUS format was also generated with partitions defined by the character sets:

```
charset m1ry = 1-3787;
charset m2ry = 3788-7573;
charset m3ry = 7574-11351;
charset stemsry = 11352-13283;
charset loopsry = 13284-15175;
```

The MSA was imported into PAUP v.4a150 (Swofford, 2003). Three estimates of base frequency for each partition were generated in which: (1) constant sites and gaps were excluded; (2) only gaps were excluded; and (3) no sites were excluded.

```
>  execute [filename].nex
>  exclude  all;  include  [partition  name];  exclude  constant
gapped; basefreqs
>  exclude  all;  include  [partition  name];  exclude  gapped;
basefreqs
> exclude all; include [partition name]; basefreqs
```

For each partition, both standard- and RY-coded, base frequencies were copied into *Microsoft's* Excel in order to calculate RCV, which is given by the sum of the absolute deviation of each base frequency for each taxon from the average across all taxa, divided by the number of taxa (Phillips et al., 2010). Chi-squared tests for significant base composition bias were also generated and are recorded in Supplementary Table 5 alongside RCV estimates.

Supplementary Table 5 RCV calculations and Chi-Squared test estimates for both nucleotide and RY-coded mitochondrial partitions when constant sites and gaps are excluded, when only gaps are excluded, and when no sites are excluded.

	Exclude constant gapped				Exclude gapped				Exclude none			
	NT		RY		NT		RY		NT		RY	
Partition	RCV	χ2 *p*-value	RCV	χ2 *p*-value	RCV	χ2 *p*-value	RCV	χ2 *p*-value	RCV	χ2 *p*-value	RCV	χ2 *p*-value
m1	0.05	0.87	0.07	0.90	0.01	1.00	0.00	1.00	0.02	1.00	0.00	1.00
m2	0.05	1.00	0.07	1.00	0.01	1.00	0.00	1.00	0.01	1.00	0.00	1.00
m3	0.03	0.73	0.05	0.78	0.02	0.97	0.01	1.00	0.02	0.04	0.01	1.00
stem	0.08	1.00	0.03	1.00	0.02	1.00	0.00	1.00	0.02	1.00	0.01	1.00
loop	0.08	1.00	0.13	0.95	0.02	1.00	0.02	1.00	0.03	1.00	0.01	1.00
all	0.02	0.07	0.04	0.81	0.01	1.00	0.00	1.00	0.01	0.00	0.01	0.03

χ2 *p*-values below 0.05 indicate significant base composition bias for that partition, but the power of this test is highly influenced by the number of sites included (Phillips et al. 2010). A reduction in RCV estimates with RY-coding indicates that base composition bias is somewhat mitigated by RY-coding, and this test gives a better indication of base composition bias.

Regardless of whether constant sites and gaps are excluded, or just gaps, or no sites, χ2 *p*-values are above 0.05 for all partitions indicating insignificant base composition bias. RY-coding each partition does not decrease the χ2 *p*-value. Likewise, RCV does not increase by RY-coding any partition (Supplementary Table 5). These results suggest that in order to resolve the relationships among elephant birds no RY coding of any partitions is necessary.

5.8.3 Stemminess analysis

"Stemminess is a measure of the amount of phylogenetic signal erosion, and can be used alongside RCV to determine whether RY-coding certain partitions will mitigate biases in the data (i.e., "compositional heterogeneity") that may lead to incorrect phylogenetic inference" (Phillips and Pratt 2008; Phillips et al., 2010). In order to calculate uncorrected stemminess, first a null hypothesis tree was generated in PAUP v.4a150.

```
> execute [filename].nex
> hsearch
> contree
> out DRONO
> showtree
```

The generated tree was converted to Newick format and was added to the NEXUS files above in a TREES block:

```
begin trees;
tree nulltree =
(DRONO,(DRONO2,((CASBE,CASCA),(((APTOW,APTHA),(APTMA,APTMA2)),((2988,
((2973,(2990,2980)),(3004,(MULAG,3221)))),((AEPHI,(2987,(2997,(2998,3
```

```
011)))),((3025,3024),(1666,(2986,(3026,(3220,((1665,3219),(3222,3258)
))))))))))));
```

Branch lengths were then generated for each partition, both standard- and RY-coded, with: (1) constant sites and gaps were excluded; (2) only gaps were excluded; and (3) no sites were excluded.

```
> execute [filename].nex
> DerootTrees
> out DRONO
> set crit = dist
> dset distance = abs negbrlen = prohibit
> exclude all; include [partition name]; exclude constant gapped
> describetrees 1/plot = phylogram brlens = yes
```

Branch lengths were imported into *Microsoft's* Excel in order to calculate uncorrected stemminess per partition, which is defined as the sum of the internal branch lengths of a tree divided by the sum of internal and external branch lengths. Results are recorded in Supplementary Table 6.

In order to calculate whether a model will correct for substitution saturation, stemminess was calculated for both standard- and RY-coded partitions after Bayesian tree generation. Standard- and RY-coded partitions were combined into a NEXUS file that was followed by a MrBayes block:

```
Begin MrBayes;

charset m1 = 1-3787;
charset m2 = 3788-7573;
charset m3 = 7574-11351;
charset stems = 11352-13283;
charset loops = 13284-15175;
charset m1ry = 15176-18962;
charset m2ry = 18963-22732;
charset m3ry = 22733-26510;
charset stemsry = 26511-28442;
charset loopsry = 28443-30334;

partition bycodon = 10: m1, m2, m3, stems, loops, m1ry, m2ry,
m3ry, stemsry, loopsry;
set partition=bycodon;
lset applyto=(1-5) nst=6 rates=invgamma;
lset applyto=(6-10) nst=1 rates=invgamma;
outgroup DRONO;

prset applyto=(all) statefreqpr = fixed(empirical);
unlink statefreq=(all);
unlink revmat=(all);
unlink shape=(all);
unlink pinvar=(all);
unlink brlens=(all);
mcmc ngen=10000000 printfreq=5000 samplefreq=5000 nruns=2
nchains=3 temp=0.1 diagnfreq=50000 savebrlens=yes
filename=[filename];
SUMT filename=[filename] relburnin=yes burnin=0.2
contype=allcompat;
SUMP filename=[filename]  relburnin=yes burnin=0.2;
end;
```

MrBayes v3.2.6 (Huelsenbeck and Ronquist, 2001) was implemented through the online bioinformatics platform CIPRES v 3.3 (Miller et al., 2010). The output files from MrBayes (.t) were modified such that they could be imported into TreeStat v1.2 (Rambaut and Drummond, 2007). The 'Treeness' values generated for each partition and each run were imported into *Microsoft's* Excel to calculate the average corrected stemminess for each partition (with 95% confidence; Supplementary Table 7).

Higher values of stemminess indicate that there is less saturation of phylogenetic signal, which can mitigate some of the base composition bias observed. Stemminess is not increased by RY coding any partitions (Supplementary Table 6); in fact, uncorrected stemminess is *decreased* by RY coding, indicating that phylogenetic signal is lost by RY coding. Note that uncorrected stemminess could not be calculated when partitions are RY coded *and* constant sites and/or gaps are excluded as the branch lengths became 0. This result further supports the notion that significant phylogenetic signal is lost by RY coding, suggesting that no partitions should be RY coded.

Supplementary Table 6 Uncorrected stemminess estimates for both nucleotide and RY-coded mitochondrial partitions when constant sites and gaps are excluded, when only gaps are excluded, and when no sites are excluded.

	Exclude constant gapped		Exclude gapped		Exclude none	
	NT	RY	NT	RY	NT	RY
Partition	Stemminess	Stemminess	Stemminess	Stemminess	Stemminess	Stemminess
m1	0.64	NA	0.64	NA	0.62	0.03
m2	0.53	NA	0.53	NA	0.53	0.02
m3	0.73	NA	0.73	NA	0.66	0.03
stem	0.60	NA	0.60	NA	0.29	0.03
loop	0.58	NA	0.58	NA	0.28	0.02
all	0.70	NA	0.70	NA	0.58	0.02

Corrected stemminess is not significantly increased by RY coding any partitions, (Supplementary Table 7), suggesting that a model alone may correct for any substitution saturation.

Supplementary Table 7 Average corrected stemminess estimates for both nucleotide and RY-coded mitochondrial partitions. Upper and lower bounds for a 95% confidence interval for the mean are also shown.

	Stemminess			Stemminess-RY		
Partition	Average	Upper	Lower	Average	Upper	Lower
m1	0.71	0.74	0.68	0.65	0.74	0.56
m2	0.55	0.60	0.49	0.50	0.62	0.38
m3	0.88	0.89	0.87	0.87	0.90	0.84
stems	0.51	0.57	0.45	0.44	0.56	0.32
loops	0.72	0.76	0.68	0.68	0.77	0.59

The RCV and stemminess results indicate that RY coding of any mitochondrial positions may result in a significant loss of phylogenetic signal.

5.8.4 ModelTest analysis

To find the best substitution model for each partition, the standard-coded MSA was executed in PAUP v.4a152, followed by execution of a modelblock.nex file:

```
> execute [filename].nex
> exclude all; include [partition name]
> execute modelblock.nex
```

The output file for each partition (model.scores) was placed in the same folder as a jModelTest3.7 (Guindon and Gascuel, 2003; Darriba et al., 2012; Posada and Crandall, 1998) executable, which was evoked through command prompt, specifying the number of characters in the partition (-n) and the number of taxa (-t):

```
> cd c:\[path to executable]
> modeltest3.7win –n3787 –t32 < m1_model.scores > m1.modeltest
```

The best scoring models as determined through hierarchical likelihood ratio tests (hLRT) and corrected Akaike information criterion (AIC) are recorded in Supplementary Table 8.

Supplementary Table 8 ModelTest results for each mitochondrial partition. MrBayes v3.2.6 parameters used when partitions are standard or RY-coded are also indicated. A GTR+G model was used for all partitions in RAxML v1.5.

Partition	hLRT	AIC	Model used	NT			RY
				nst	rates	statefreqpr	nst
m1	TrN	TIMef	SYM	6		fixed (equal)	-
m2	TrN	TIMef	SYM	6		fixed (equal)	-
m3	TrN	TrNef+G	SYM+G	6	gamma	fixed (equal)	1
stems	TvMef	TIMef	SYM	6		fixed (equal)	-
loops	TrN	TIMef	SYM	6		fixed (equal)	-

5.8.5 Maximum likelihood phylogeny generation

An input .phy file where missing data was coded with N (rather than ?) was run in RAxML v1.5 (Stamatakis, 2014) with the following parameters: Data type = DNA or mixed; Analysis / Multiple outgroups; Import partitions.txt (DNA for nt coded, BIN for ry coded); Auto mre = 500; BS brL; GTRGammaI. The output was a 'bipartitions.tre' file.

5.8.6 Bayesian phylogeny generation

To run the Bayesian phylogeny, only one outgroup taxon was included ("DRONO2"); DRONO, CASCA and CASBE sequences were removed so the tree would be rooted properly. MrBayes v3.2.6 (Huelsenbeck and Ronquist, 2001) was implemented via the online bioinformatic CIPRES v3.3 science gateway (Miller et al., 2010). Bayesian inference analyses were run with unlinked substitution models and branch-length rate multipliers among the partitions (Phillips et al., 2008). Three MCMC chains for each of two independent runs proceeded for 10^7 generations with trees sampled every 2500 generations. The parameters specified in the input NEXUS file were as follows: Ntax = number of taxa; Nchar

= number of characters; Format datatype = dna; Gap = -; Interleave = yes; Partition by codon = list of the partition names included; Set partition = bycodon; Lset applyto= [partition #]; nst=1, 2, or 6 (Supplementary Table 8); rates=invgamma/gamma [determined from ModelTest]; Outgroup DRONO2; Prset applyto = all; statefreqpr = fixed (empirical); Unlink statefreq=all; Unlink revmat=all; Unlink pinvar=all; Unlink brlens=all; Link brlens= [certain partitions]; Link brlens= [other partitions]; Prset applyto = [some partitions]; ratepr= variable; Prset applyto = [other partitions]; ratepr= variable; Mcmc ngen = 10000000; printfreq=5000; samplefreq=2500; nruns=2; nchains=3; temp=0.1; diagnfreq=5000; savebrlens=yes; filename=[filename]; SUMT filename= [filename]; relburnin=yes; burnin=0.2; SUMP filename= [filename]; relburnin=yes; burnin=0.2. The output was a 'con.tre' file.

The burn-in for each MrBayes run ensured that –lnL had plateaued, clade frequencies had converged between runs (i.e., the average standard deviation of split frequencies was <0.02, approaching 0), and that PRSF (potential scale reduction factors, a convergence diagnostic) approached 1 (Phillips et al., 2008). Output files were then opened in Tracer v1.6.1 (Rambaut et al., 2003) to ensure that estimated sample sizes (ESS) for substitution parameter estimates were above 200, and that parameter probability estimates had overlapping distributions, further confirming that runs had converged.

5.9 Assessment of genetic variation in barcoding genes

Specimens exhibiting less than 10% missing bases across a 596 bp barcoding region of cytochrome oxidase I (COI) were used to calculate the genetic distance within and between elephant birds from each region in Madagascar, as has been done for moa (Huynen et al. 2014). An alignment of 13 sequences was imported into MEGA v6.06 (Kumar et al., 2013; Stecher et al. 2020). The within-group and between-group mean Kimura 2-parameter distance (Kimura, 1980) was estimated, implementing the following options: variance estimation method = none, substitution type = nucleotide, model/method = Kimura 2-parameter model, substitutions to include = d: Transitions + Transversions, rates among sites = uniform rates, pattern among lineages = same (homogeneous), gaps/missing data treatment = pairwise deletion, select codon positions = 1st, 2nd, 3rd, noncoding sites. To gauge the limits of intra- and inter-specific variation in this barcoding region, the distance within and between species of moa, rhea, emu, cassowary, and kiwi were also estimated in the same way using published sequences obtained from GenBank (Accessions: *Euryapteryx curtus* KF888653, AY833118; *Euryapteryx geranoides* AY833116, AY833117, AY833114, AY833115, AY833119; *Emeus crassus* AY833120, AF338712, NC_002673, AY833121; *Anomalopteryx didiformis* AY833122, AF338714, NC_002779; *Dinornis robustus* AY833123, AY833124, AY822127, AY833128, AY8833125, AY833126; *Dinornis giganteus* AY016013, NC_003672; *Dinornis novaezealandiae* AY833129*; Megalapteryx didinus* AY833130; *Pachyornis elephantopus* AY833105, AY833106, AY833107, AY833108; *Pachyornis mappini* AY833109, AY833110, AY833111; *Pachyornis australis* AY833112, AY833113; *Rhea americana* AF090339, JN801968, JN801969, NC_000846, Y16884; *Rhea pennata* (*Pterocnemia pennata*) AF338709, JN801970, JN801971, JN801972, NC_002783; *Casuarius casuarius* AF338713, NC_002778; *Casuarius bennetti* AY016011, CBU76058; *Dromaius novaehollandiae* AY016014, NC_002784; *Apteryx australis* AAU76057; *Apteryx australis mantelli* EU525309, EU525313, EU525314, EU525315, EU525318, EU525316, EU525317, AY016010, EU525308, 525311, 525312, 525310; *Apteryx rowi* EU525322, EU525323, EU525324; *Apteryx haastii* AF338708, NC_002782, EU525306, EU525307, EU525305; *Apteryx owenii* EU525319, EU525320, GU071052, NC_013806, EU525321). Analyses were also conducted using the full COI gene with (i) complete deletion of missing bases, (ii) pairwise deletion of missing bases, and (iii) partial deletion of missing bases (85%), as well as with (a) data from all 20 taxa sequenced and (b) data from the 13 taxa exhibiting less than 10% missing bases. The overall pattern of intra- versus inter-specific variation in COI obtained from these alternative analyses was not significantly different from the results

reported in the main manuscript (Supplementary Data 5). Supplementary Figure 2 summaries the results in Supplementary Data 5.

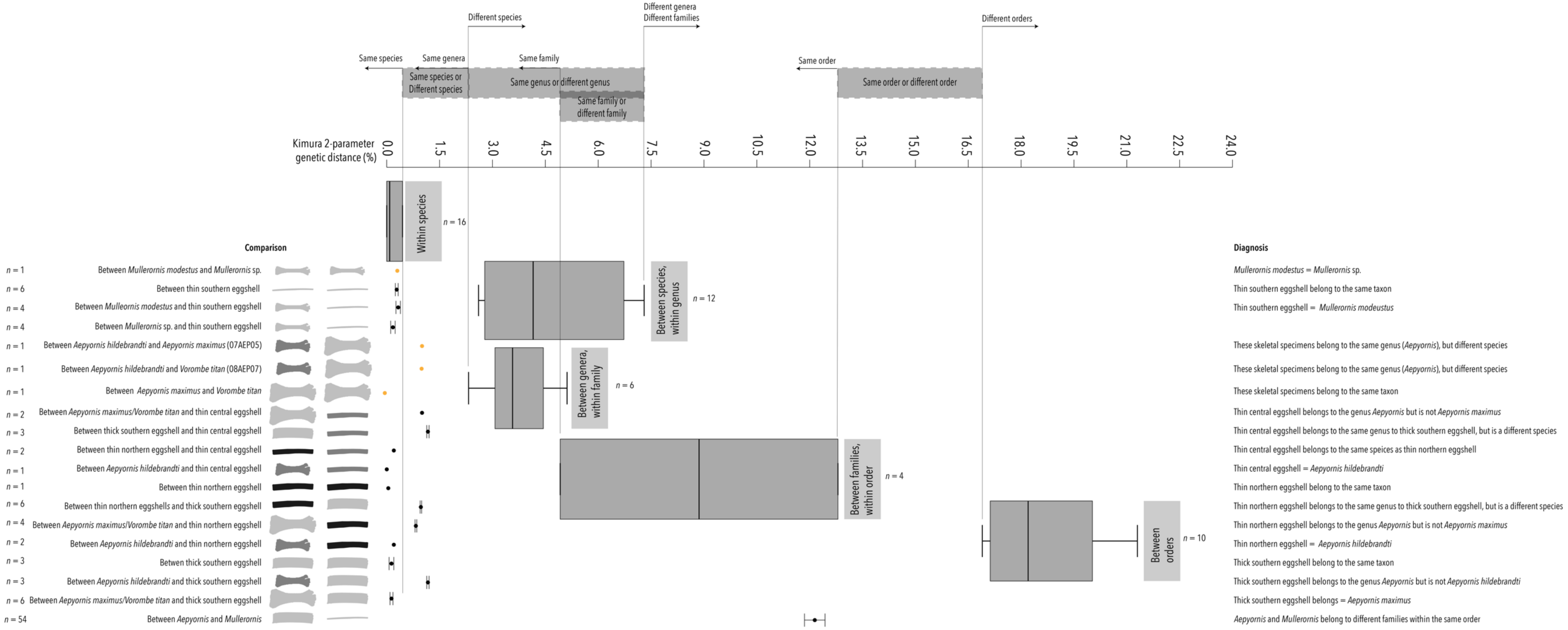

Supplementary Figure 2 | Mean Kimura-2 parameter genetic distance (%) both within and between elephant bird specimens (gold circles; whiskers represent 95% CI of the standard error) relative to other ratites (box plots). Box plots show the mean, a 95% confidence interval of the standard error (grey box) and the range (minimum to maximum, whiskers). *n* refers to the number of independent comparisons used (see Supplementary Data 5).

5.10 Species delimitation

The maximum likelihood phylogenetic tree generated was imported into Geneious v. 10.0.5 for analysis using the Species Delimitation plugin (v. 1.03; Masters et al. 2010). The central Madagascan clade containing *Aepyornis hildebrandti* was designated as a group, while the northern Madagascan clade was designated as another group. Results were obtained for the statistics P(Randomly Distinct) and Rosenberg's P(AB), where separate species are likely to exist where P(Randomly Distinct) <0.05, and Rosenberg's P(AB) <0.05. Results can be found in Supplementary Table 9.

Supplementary Table 9. Output from the species delimitation software (Masters et al. 2010) as implemented in Geneious v. 10.0.5 comparing the central Madagascan clade to the northern Madagascan clade.

Statistic	Definition (Masters et al., 2010)	Central	Northern
**Monophyletic?**	Whether the species is monophyletic or not.	Yes	Yes
**Intra Dist**	The average pairwise tree distance among members of the focal species.	6.36E-4	0.001
**Inter Dist - Closest**	The average pairwise tree distance between members of the focal species and members of the next closes species.	0.009	0.009
**Intra / Inter**	Provides a measure of genetic differentiation between the focal species and its nearest neighboring species.	0.07	0.13
**P ID (Strict)**	The mean probability, with 95% confidence interval (CI) for the prediction, of making a correct identification of an unknown specimen of the focal species using placement on a tree and the criterion that it must fall within but not sister to the species clade.	0.56 (0.41, 0.71)	0.78 (0.64, 0.92)
**P ID (Liberal)**	The mean probability, with 95% CI for the prediction, of making a correct identification of an unknown specimen of the focal species using BLAST, DNA barcoding, or placement on a tree, with the criterion that it falls sister to or within a monophyletic species clade.	0.94 (0.79, 1.0)	0.95 (0.84, 1.0)
**Av(MRCA – tips)**	The mean distance between the most recent common ancestor of a species and its members.	3.180E-4	7.685E-4
**P(Randomly Distinct)**	The probability that a clade has the observed degree of distinctiveness due to random coalescent processes. A value <0.05 indicates the possibility that a cryptic species is present, as the lineage is not conforming to a Wright-Fisher model (we can only conclude that the focal group has branching significantly different to what we would expect under the coalescent process if p<0.05.	<0.05	<0.05
**Clade Support**	Bootstrap support (%).	100.0	100.0
**Rosenberg's P(AB)**	The probability that species A represented by a sequences in a clade of a+b sequences will be reciprocally monophyletic with the remaining b sequences under the null model of random coalescence.	0.03	0.03

5.11 Mantel test

Mullerornis

```
> mullerornis_loc<-read.csv("mul_loc.csv", header=TRUE)
> mullerornis_loc_dist<-dist(cbind(mullerornis_loc$Lat, mullerornis_loc$Lon))
> mullerornis_loc_dist_mat<-as.matrix(mullerornis_loc_dist)
> mullerornis_patristic_dist<-
  read.csv("patristic_distances_mul.csv",header=FALSE)
> mullerornis_patristic_dist_mat<-as.matrix(mullerornis_patristic_dist)
>
  mantel.test(mullerornis_loc_dist_mat,mullerornis_patristic_dist_mat,nrepeat=9
  999)
```

```
$z.stat
[1] 0.2685826

$p
[1] 0.135

$alternative
[1] "two.sided"
```

Southern *Aepyornis*

```
data<-read.csv("patristic_distances_aepS_b.csv", header=FALSE)
data2<-read.csv("euclidian_loc_distance_aepS_b.csv", header=FALSE)

data_dist<-as.matrix(data)
data2_dist<-as.matrix(data2)
mantel.test(data_dist,data2_dist,nrepeat = 9999)

$z.stat
[1] 0.307282

$p
[1] 0.093

$alternative
[1] "two.sided"
```

All *Aepyornis*

```
> aepyornis_loc<-read.csv("aep_loc.csv", header=TRUE)
> aepyornis_loc_dist<-dist(cbind(aepyornis_loc$Lat, aepyornis_loc$Lon))
> aepyornis_loc_dist_mat<-as.matrix(aepyornis_loc_dist)
> aepyornis_patristic_dist<-read.csv("patristic_distances_aep.csv",
  header=FALSE)
> aepyornis_patristic_dist_mat<-as.matrix(aepyornis_patristic_dist)
> mantel.test(aepyornis_loc_dist_mat, aepyornis_patristic_dist_mat,
  nrepeat=9999)
$z.stat
[1] 9.728106

$p
[1] 0.001

$alternative
[1] "two.sided"
```

5.12 Molecular dating

For molecular dating, the mitochondrial third codon positions were RY coded to alleviate saturation at deep time scales as per Grealy et al. (2017). The alignment included the nuclear data and all taxa from Grealy et al. (2017), but in addition included the best representative eggshell samples from each elephant bird clade sequenced in this paper for a total of 37 taxa.

The input parameters for molecular dating analysis using MCMCtree (within PAML v4.4d; Yang et al., 2006; Yang, 2007) were: seed = -1, ndata = no. partitions (depending on the dataset), seqtype = 0, usedata = 3, RootAge = >66.4<124.1, model = 7, alpha = 1, ncatG =4, cleandata = 0, BDparas = 1 1 0, kappa_gamma = 6 2, alpha_gamma = 1 1, rgene+gamma = 1 2, sigma2_gamma = 1 1, finetune = 1, print = 1, burnin =

12,000, sampfreq = 50, nsample = 1500. The output .BV file was renamed to in.BV and used as the input to rerun the analysis (i.e. usedata = 2). Analyses with both independent (clock = 2) and correlated (clock =3) rates were performed using the phylogenetic tree topology in Figure 2. In addition, analyses with both independent (clock = 2) and correlated (clock = 3) rates were performed using a phylogenetic topology where rheas are deepest among the notopalaeognathae (since the placement of rheas or moa/tinamous as deepest among the notopalaeognathae is not well resolved). Each analysis was replicated.

With independent rates there appears to be more of a tendency for rate slowdowns in large, long-lived lineages to be inferred as starting deeper in the tree; this is less likely to happen with autocorrelated rates because this model biases against substantial rate shifts across short branches (e.g., deep among the palaeognath radiation). While there are likely to be multiple independent rate decelerations among ratites, the *a priori* expectation would be that these would not occur along the short basal-most lineages, but the longer lineages leading to large and/or long-lived ostrich, rheas, moa, elephant birds, etc. (since rate correlates like size and longevity are predicted to shift along these lineages). Further, ratograms suggest that there is a somewhat bimodal distribution of rates across the branches of the tree: slow rates among the flightless bird tips and their shallow stems, then faster rates among outgroups and among deeper palaeognaths. The independent (lognormal) rates distribution is a poor fit for a distribution that is somewhat bimodal, whereas such as distribution does not violate the autocorrelated rates model. For this reason, dates estimated using the autocorrelated rates model was presented in Figure 2, but all analyses can be found on DataDryad (see "Data availability statement" in the main text). The results of both are also summarised in Supplementary Data 6 below.

A total of nine calibrations were used (four palaeognath, Supplementary Table 10), most of which are identical to Grealy et al. (2017), with the following changes:

- Elephant bird/Kiwi calibration as a minimum bound set to 16 Ma (0.16) for St Bathans fauna kiwi
- Emu/Cassowary shifted from >17 Ma to 'B(0.245,0.723,0.05,0.025)' so that the minimum is 5%, maximum is 2.5%. Note that the minimum bounds 2.5% by default, but we used a more conservative 5% when prudent (i.e., when the placement of the fossil taxon within the calibrated clade is agreed among recent studies, but statistical support does not warrant 2.5%; 1% was used in cases where appropriate, i.e., very high statistical phylogenetic support).
- Emu/Cass vs Elephant bird/Kiwi calibration is now largely redundant, so is removed
- Base of Notopalaeognathae (non-ostrich ratites) stays 56-72.3 Ma, but is now a normal distribution with 56 Ma at 5% and 72.3 Ma at 2.5%: this is 'SN(0.6344,0.0452,0)'
- Note that skew normal with shape=0 is NORMAL. Then location is mean and scale is the number of Mya per S.D.
- Root is 66.5–124.5 Ma, but is now normal and 1% either end: 'SN(0.953,0.1238,0)'.
- Neoaves is 60.5–72.3 Ma, but is now normal and 1% at both ends: 'SN(0.664,0.0254,0)'.
- Note that *Asterornis* (Field et al., 2020) now provides a slightly older minimum for the root (66.7 Ma), and Tsidiiyazhi 62.221 Ma (Ksepka et al., 2017) provides an older minimum for Neoaves; however, these new calibrations would slightly change these skew-normal priors but would have such little effect on the timing that the divergence estimates would essentially be within the small variation expected from running the same calibrations many times. This is because the posterior distributions for both nodes do not even come close to the new or previous minimum bounds.

Supplementary Table 10 Palaeognath fossil calibrations used for molecular dating.

Node	Minimum age (Ma)	Maximum age (Ma)	Justification	Reference
Casuariiformes	24.5	72.5	*Emuarius* (Riversleigh Upper Site) Minimum= *Emuarius* = oldest generally agreed crown member. Maximum covers absence of non-ostrich (or any) palaeognathae from global Maastrichtian avifaunas.	Woodhead et al. 2014 Gondwana Research Worthy TH, Hand SJ, Archer M. 2014. Phylogenetic relationship of the Australian Oligo-Miocene ratite *Emuarius gidju* Casuariidae. *Integr. Zool.* **9** (148-166).
Tinamiformes	16.9	58.7		Ksepka DT & Phillips MJ. 2015. Avian diversification patterns across the K-PG boundary: influence of calibrations, datasets, and model misspecification. *Annals of the Missouri Botanical Garden* **100** (300-328).
Non-ostrich ratites	56	72.3	Minimum = *Diogenornis* (variously found to be a stem rhea or cassuariiform); maximum covers absence of non-ostrich palaeognaths (or any) palaeognathae from global Maastrichtian avifaunas.	Alvarenga HME. 1983. Uma ave ratita do paleoceno brasileiro: bacia caladria de Itaborai, estato do rio de Janeiro, Brasil. Boletim do Museu Nacional (Rio de Janeiro). *Geologica* **37** (1-194). Phillips et al. (2011)
Elephant birds/Kiwi	16		*Proapteryx*	Worthy TH, Worthy JP, Tennyson AJD, Salisbury SW, Hand SJ & Scofield RP. 2013. Miocene fossils show that kiwi (Apteryx, Apterygidae) are probably not phyletic dwarves. In: Göhlich, U.B. & Kroh, A. (eds) Paleornithological Research 2013: Proceedings of the 8th International Meeting of the Society of Avian Paleontology and Evolution, Vienna, 2012. Verlag des Naturhistorischen Museums in Wien, Vienna, 2013. Verlag des Naturhistorischen Museums in Wien, Wien.: 63-80.

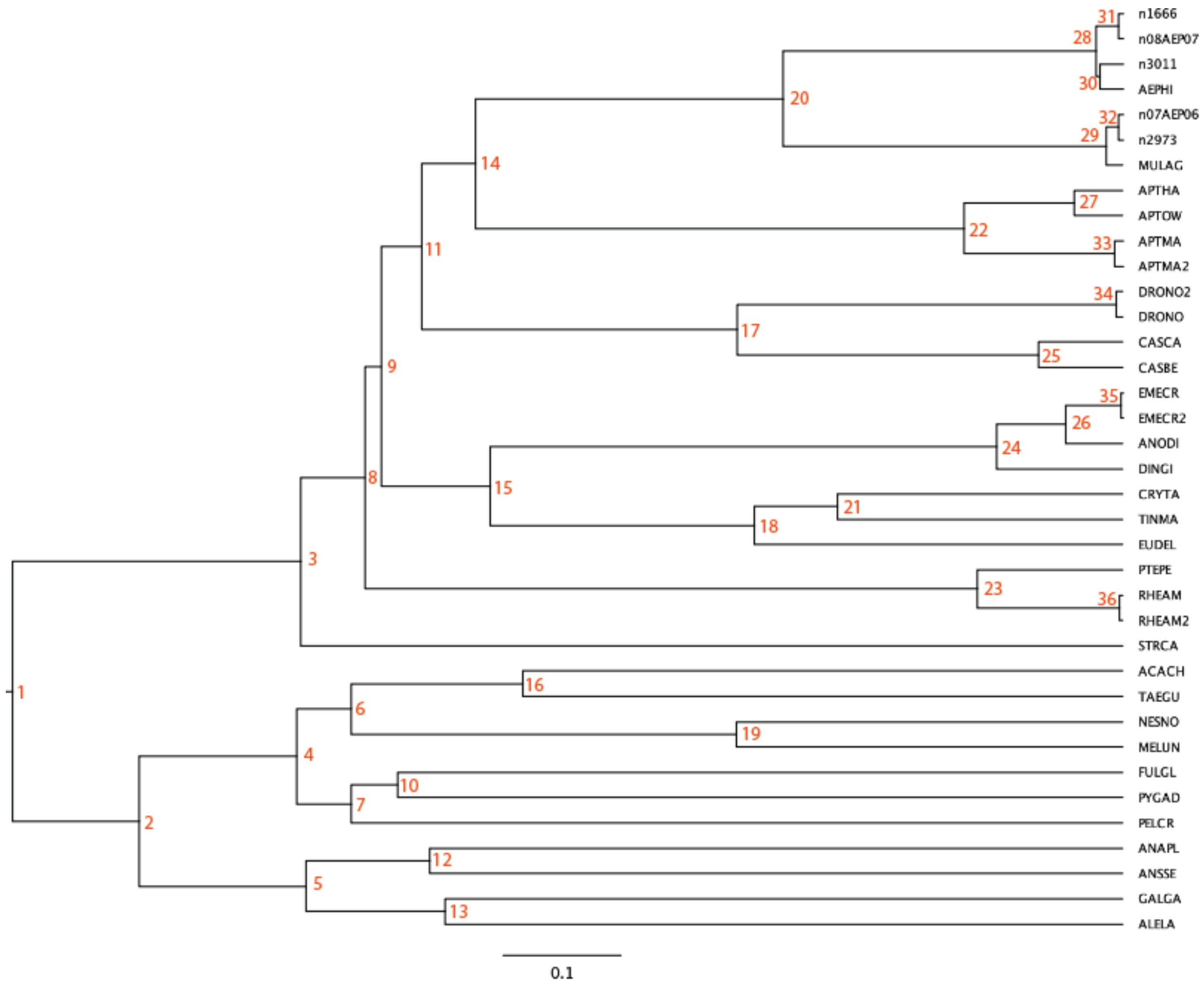

Supplementary Figure 3 The dated phylogeny from Figure 2 with nodes labelled (c.f., Supplementary Data 6).

There is very little difference in divergence estimates depending on whether moas/tinamous or rheas are deepest among notopalaeognathae. However, there are differences between estimates generated using independent versus autocorrelated rate models, namely, that the dates at the tips and middle nodes of the tree become younger with independent rates. Of note, the split between Aepyornithids and Mullerornithids is 13.02 mya younger (17.13 mya with independent rates vs 30.15 mya with autocorrelated rates), and the split between southern and north/central *Aepyornis* is also about halved in age (0.75 mya with independent rates vs 1.4 mya with autocorrelated rates). While this is a significant difference, for the reasons detailed above, the autocorrelated rates appear to be a better model.

While a 17.13 mya divergence date for the split between Aepyornithidae and Mullerornithide puts their divergence within the Miocene rather than Oligocene, Madagascar would have been continuing to move northward closer to the equator with increased precipitation but warm; however, there is evidence of a geological uplift during the mid-late Miocene, establishing the Madagascan high plateau (Delaunay et al. 2017). This altitudinal difference may have been a driver of divergence between elephant bird families, if it occurred at this time. A date of 750,000 years ago still puts the divergence between *Aepyornis* lineages within the Pleistocene ice age. In fact, this estimation would mean that the evolution of extreme gigantism in *Aepyornis maximus* occurred in an even shorter time-frame, within the span of about 500,000 years. Thus, regardless whether independent or autocorrelated rates are used to estimate divergence, most conclusions remain the same.

Supplementary Note 6
Protein mass spectrometry

6.1 Protein extraction

Protein extraction followed published protocols for ostrich eggshell proteomics analyses (Demarchi et al., 2016) at the Archaeobiomics laboratory at the University of Turin (Italy). Briefly, 30-35 mg of each of the eggshells (Supplementary Data 7) were powdered using a clean agate pestle and mortar and bleached in sodium hypochlorite (NaOCl, 12% w/v) for 72 hours in order to isolate a stable fraction of intracrystalline molecules (Crisp et al., 2013). Bleached powders were rinsed in ultrapure water and methanol and demineralised in cold 0.6 M hydrochloridric acid (HCl). Solutions containing the organic extracts were ultrafiltered (Nanosep Centrifugal Devices, 3-kDa MWCO, Pall Laboratory) and resuspended in ammonium bicarbonate buffer (50 mM). Following reduction and alkylation of disulphide bonds with DTT and IAA, samples were split into two subsamples ("T" and "E", where T=trypsin and E=elastase) and digestion was carried out overnight at 37°C by adding 4 µL trypsin (0.5 µg/µL; Promega, 2800 Woods Hollow Road Madison, WI 53,711 USA) for 'T' subsamples and 4 µL elastase (1 µg/µL; Worthington, Lakewood, NJ, USA) for 'E' subsamples. Digests were purified using C_{18} solid-phase extraction (Pierce zip-tip; Thermo-Fisher) according to the manufacturer's instructions. Eluted peptides were evaporated to dryness before being sent for LC-MS/MS analyses.

6.2 LC-MS/MS analyses

Eluted and dried down peptides were received at the Novo Nordisk Centre for Protein Research (Copenhagen, Denmark) and suspended in 50 µL 80% acetonitrile (ACN) before combining both trypsin and elastase digested fractions to make 100 µL. Protein concentration was then determined by measuring the absorption at 205 nm (= the peptide bond) using a NanoDrop Spectrophotometer (Thermo Fisher Scientific, Wilmington, DE, USA). Depending on concentration, 1.5-4 µL of peptide solution was then transferred to a 96-well MS plate, to equal about 0.75 µg of protein for each sample. 40% ACN, 0.1% formic acid (FA) was added to make a total of 25 µl before using a SpeedVac (Thermo Fischer Scientific, Bremen, Germany) to concentrate the samples until ≈ 3 µL of solution was left. Samples were then resuspended with 5 µL of 0.1% trifluoroacetic acid (TFA), 5% ACN.

Samples analyzed by an EASY-nLC 1200 (Thermo Fischer Scientific, Bremen, Germany) connected to a Q-Exactive HF-X (Thermo Fischer Scientific, Bremen, Germany) on a 77 min gradient. The column was 15 cm (75 µm inner diameter), in-house laser pulled and packed with 1.9 µm C18 beads (Dr. Maisch, Germany), and maintained at 40°C using an integrated column oven. Buffer A was milliQ water. The peptides were separated with increasing buffer B (80% ACN and 0.1% FA), going from 5% to 30% in 50 min, 30% to 45% in 10 min, 45% to 80% in 2 min, held at 80% for 5 min before dropping back down to 5% in 5 min and held for 5 min. Flow rate was 250 nL/min. A wash-blank method using 0.1% TFA, 5% ACN was run in between each sample to hinder cross contamination.

The Q-Exactive HF-X was operated in data dependent top 10 mode. Spray voltage was 2 kV, S-lens RF level at 50, and heated capillary at 275°C. Full scan mass spectra were recorded at a resolution of 120,000 at m/z 200 over the m/z range 350–1400 with a target value of 3e6 and a maximum injection time of 25 ms. HCD-generated product ions were recorded with a maximum ion injection time set to 118 ms and a target value set to 2e5 and recorded at a resolution of 60,000. Normalized collision energy was set at 28% and the isolation window was 1.2 m/z with the dynamic exclusion set to 20 s.

6.3 Data Analysis

Resulting .raw files were searched using PEAKS v.8.5 (Zhang et al., 2012). Parent ion and fragment ion mass tolerance were set to 10 ppm and 0.05 Da respectively, with unspecific digestion. In ancient samples,

hydrolytic damage due to natural diagenesis is expected, as much as cleavages due to the action of the enzymes. Therefore, a non-specific search is used to maximise recovery. Deamidation of N and Q, as well as Oxidation of M, H, and W were set as variable PTMs. The thresholds for peptide and protein identification were set as follows: false discovery rate ≤ 0.5%, de novo sequences scores (ALC%) ≥ 80, unique peptides ≥ 2. The NCBI database (taxonomy restricted to Aves) was used for carrying out preliminary searches, and a database including common contaminants was included (Common Repository of Adventitious Proteins: https://www.thegpm.org/crap/). Further searches were performed against XCA-1 and XCA-2 sequences from the B10K genomes project (https://b10k.genomics.cn/), annotated manually as described in Demarchi et al. (2022). The proteomics datasets have been deposited to the ProteomeXchange Consortium via the Proteomics Identifications Database (PRIDE; see "Data availability statement" in the main text). Sequence reconstruction was carried out on the basis of the *Spider* output of the software PEAKS, taking into account both identified peptides (i.e., those fully matched to one of the reference sequences) and *de novo* only peptides (i.e., those software-reconstructed peptide sequences that cannot be matched fully but share at least seven amino acid residues with the reference sequence). Sequence reconstruction was performed manually, evaluating each peptide sequence and product ion spectrum. The protein structure of XCA-1 and XCA-2 was inferred using the ColabFold AlphaFold2 notebook (Steinegger et al., n.d.; Jumper et al., 2021). Where coverage was missing, short sequence insertions were included from the closest phylogenetic taxon (*Apteryx*) for XCA1 (residues 26, 87-88, 101, 131) and XCA2 (residues 59-74).

6.4 Protein results

Similar to other ratites (Mann, 2004; Mann and Siedler, 2004), elephant bird also contains both XCA-1 and XCA-2 C-lectins within its intracrystalline fraction (Supplementary Data 7 reports coverages for the top protein hits for each sample type). All partial reconstructed sequences were aligned and examined for evidence of amino acid substitutions between the morphotypes (Supplementary Figure 4 and Supplementary Figure 5). See Supplementary Data 7 for results.

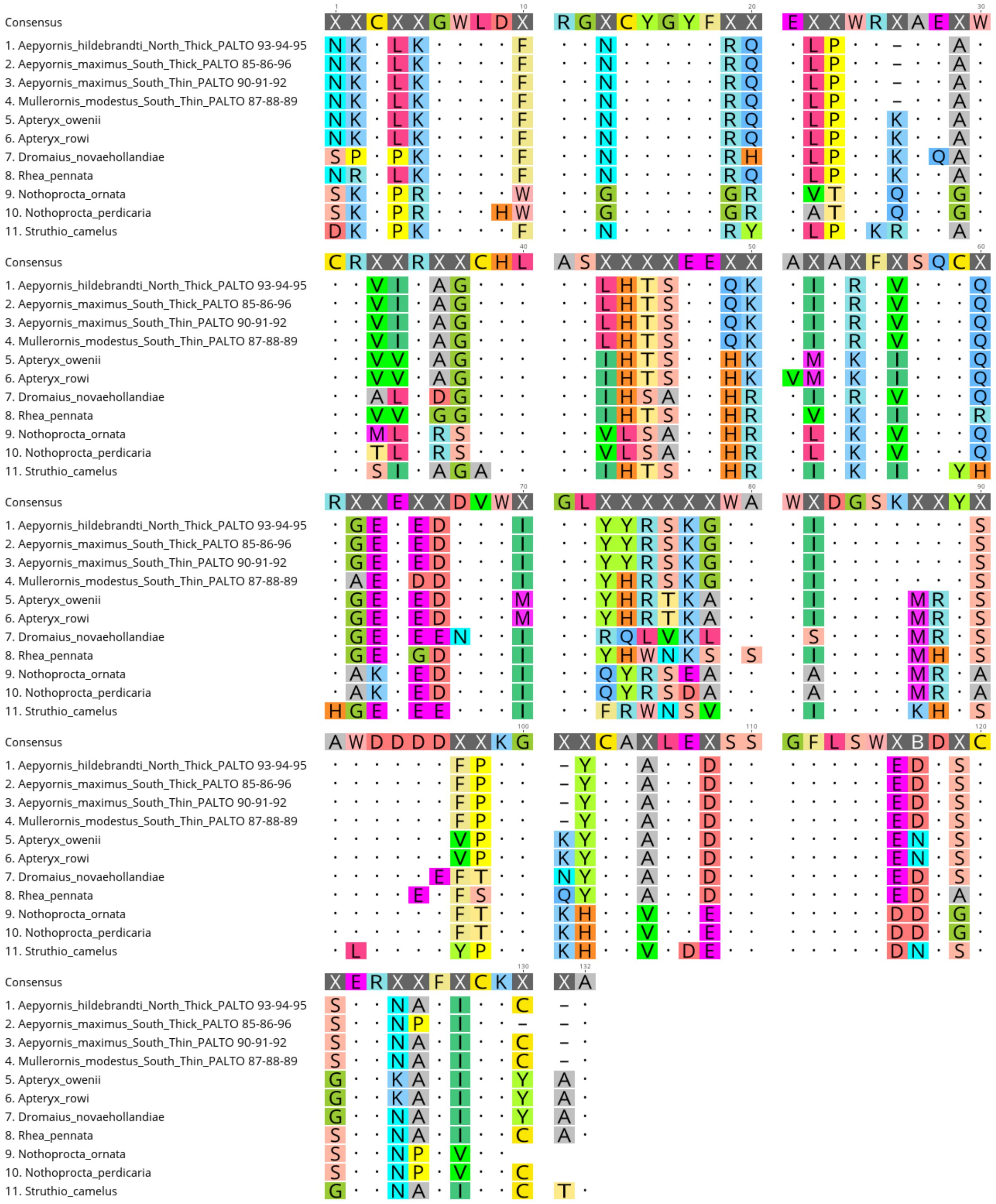

Supplementary Figure 4 Alignment of the partial type-1 C-lectins (XCA-1) sequences recovered from the elephant bird eggshells and reference sequences ("X" indicates indeterminate and "-" absent residue). Note that "Aepyornis_hildebranti_North_Thick" refers to the northern clade of eggshell, possibly a subspecies of *A. hildebrandti*; "Aepyornis_maximus_South_Thick" refers to eggshell >3 mm thick from the south, likely corresponding to *A. maximus*; "Aepyornis_maximus_South_Thin" refers to eggshell >1.6-3 mm thick from the south, also likely corresponding to *A. maximus*; and "Mullerornis_modestus_South_Thin" refers to eggshell <1.6 mm thick from the south, likely corresponding to *Mullerornis modestus*.

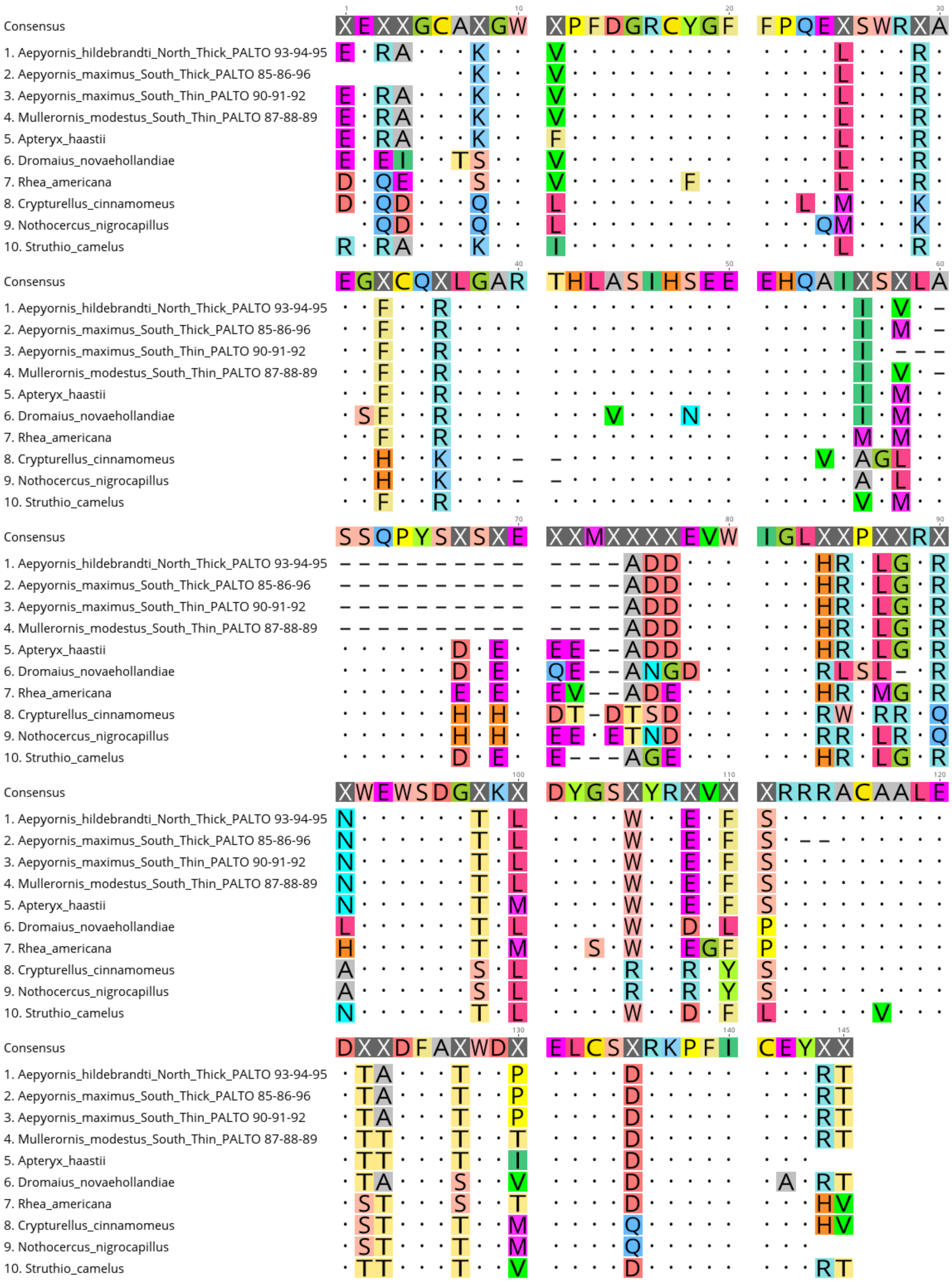

Supplementary Figure 5 Alignment of the partial type-2 C-lectins (XCA-2) sequences recovered from the elephant bird eggshells and reference sequences.

A Annotated raw MS/MS showing positions 62, 65 and 74 for XCA-1 of *Aepyornis maximus*.

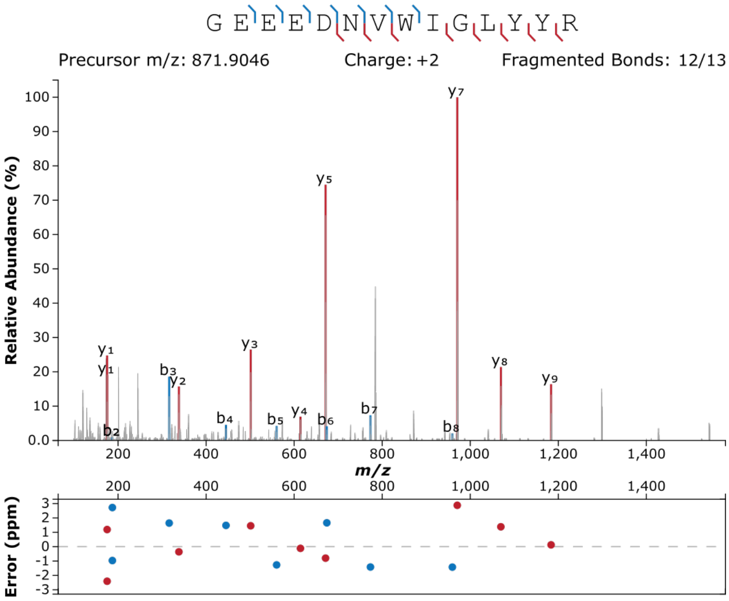

B Annotated raw MS/MS showing positions 62, 65 and 74 for XCA-1 of *Mullerornis modestus*.

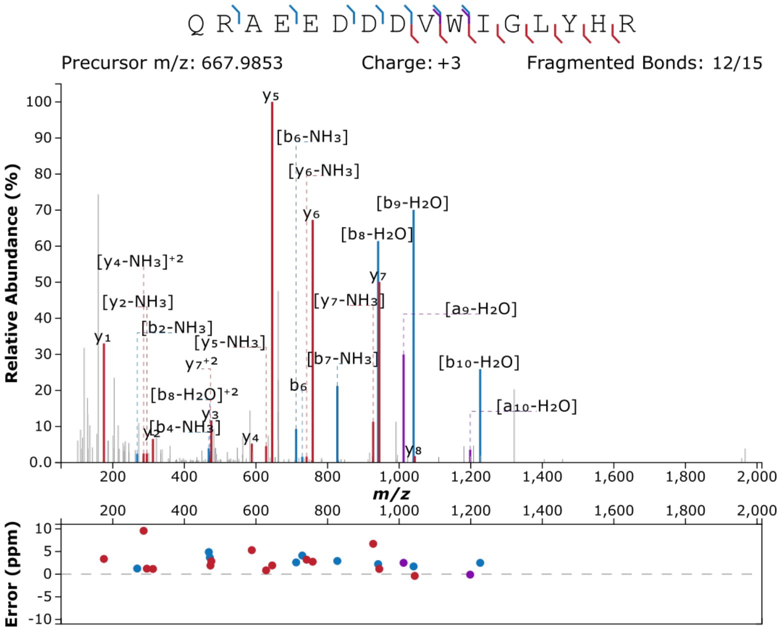

C Annotated raw MS/MS showing position 58 for XCA-2 of *Aepyornis maximus*.

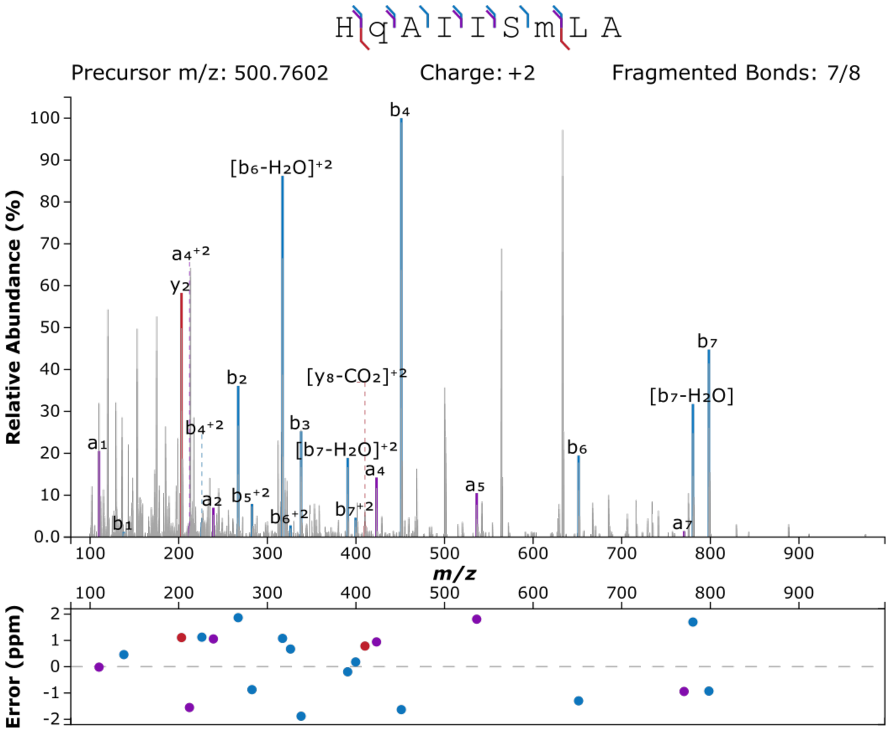

D Annotated raw MS/MS showing position 58 for XCA-2 of *Mullerornis modestus*.

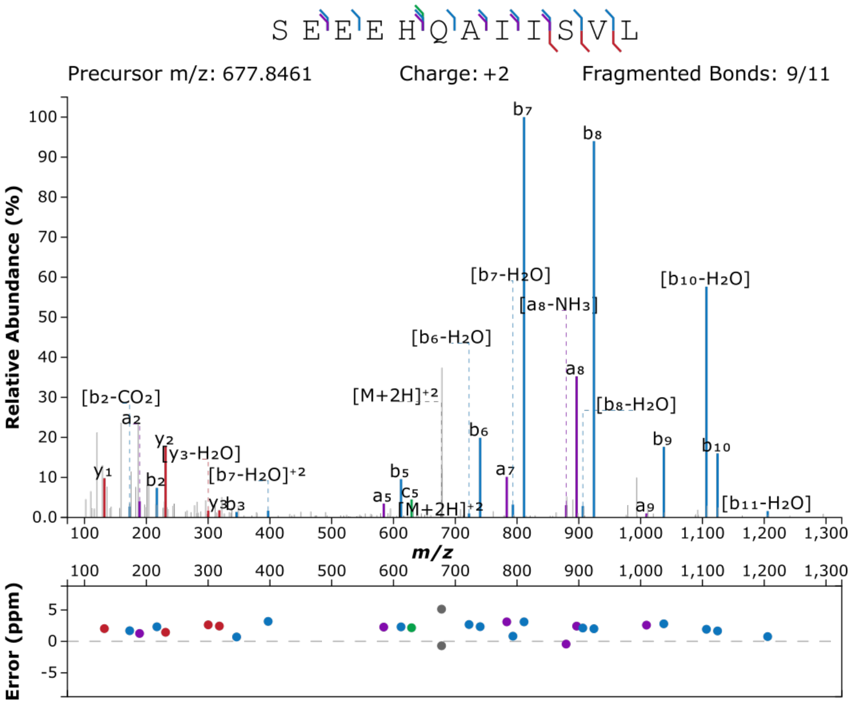

E Annotated raw MS/MS showing position 123 for XCA-2 of *Aepyornis maximus*.

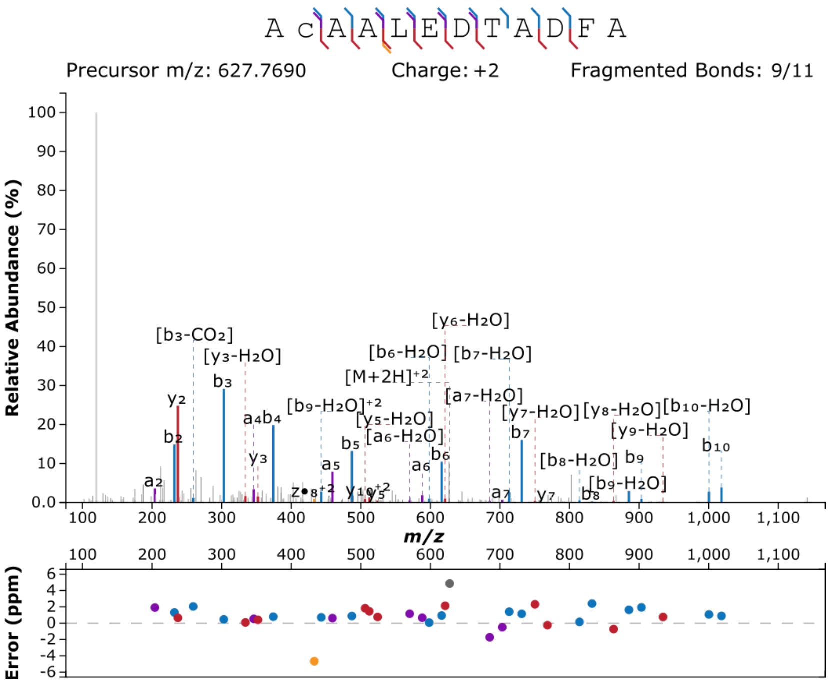

F Annotated raw MS/MS showing position 123 for XCA-2 of *Mullerornis modestus*.

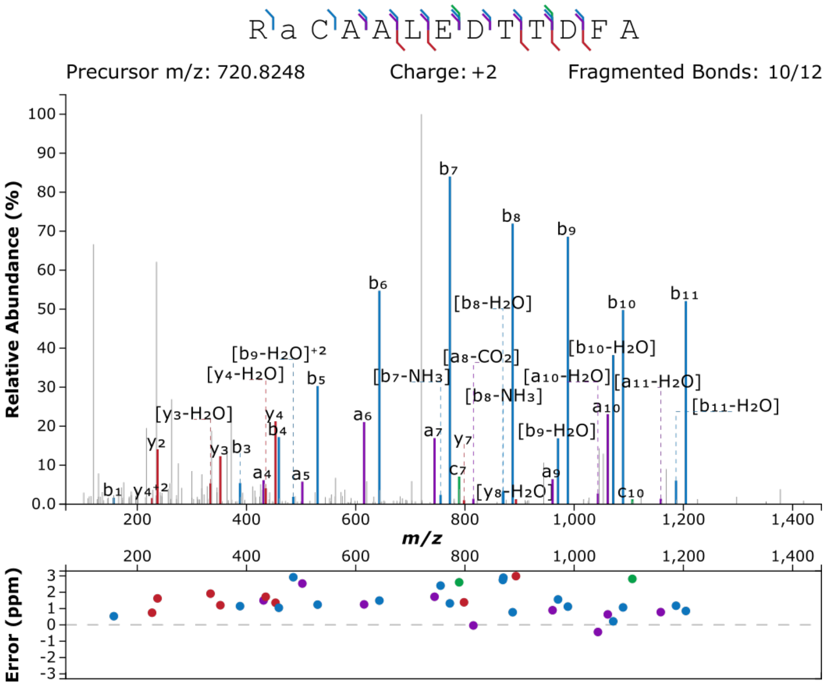

G Annotated raw MS/MS showing positions 130 for XCA-2 of *Aepyornis maximus*.

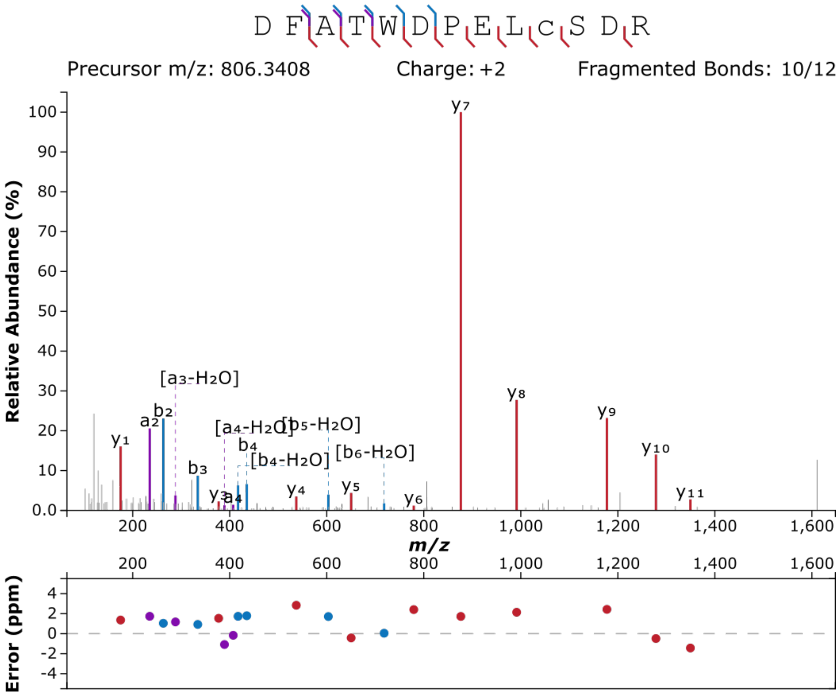

H Annotated raw MS/MS showing position 130 for XCA-2 of *Mullerornis modestus*.

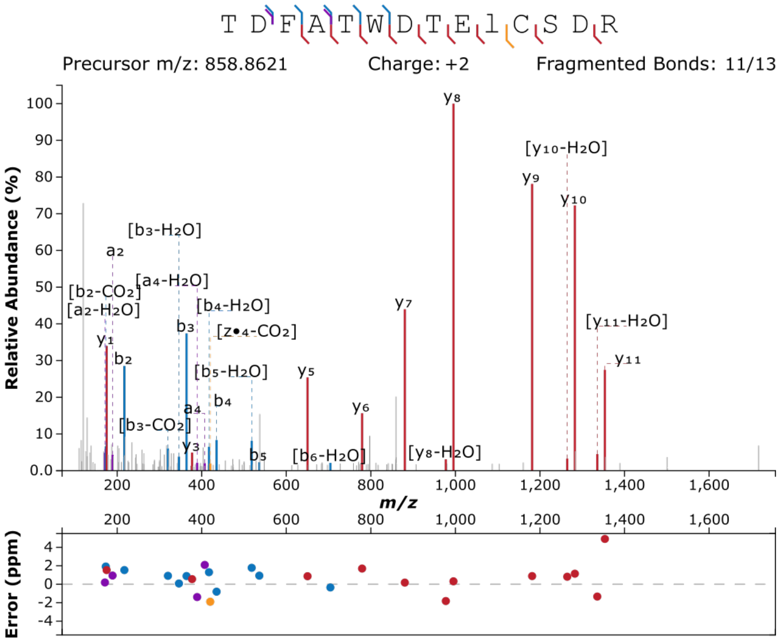

Supplementary Figure 6 De novo peptides (annotated tandem mass spectra) supporting amino acid substitutions discussed in the text, for *Aepyornis* and *Mullerornis*.

A

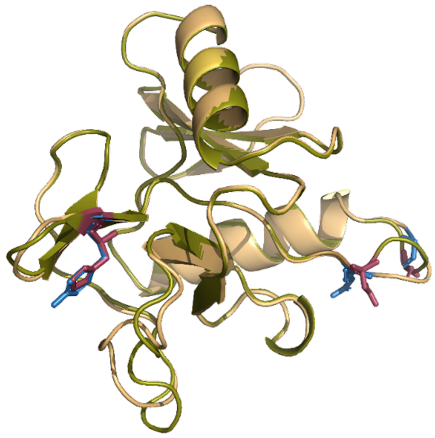

B

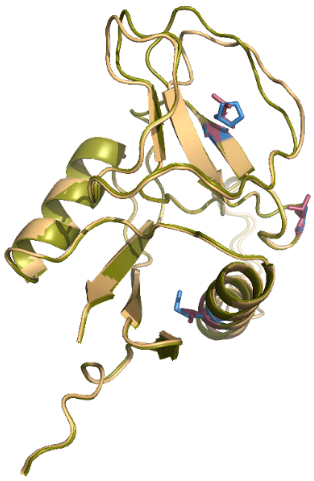

Supplementary Figure 7 Structure of XCA-1 (**A**) and XCA-2 (**B**) displaying amino acid substitutions between *Aepyornis* (light orange chain, residues highlighted blue) and *Mullerornis* (olive chain, residues highlighted red).

Supplementary Note 7
Characterisation of eggshell micromorphology by micro-CT

20 eggshell specimens (Supplementary Data 8) were submitted to the Centre for Microscopy, Characterisation and Analysis at the University of Western Australia (WA, Australia) for imaging on the Skyscan 1175 micro-CT (*Bruker-microCT*, Kontich, Belgium). The operating conditions of the CT include: a 90 kV voltage, a 278 μA current, a 0.1 mm thick copper filter, an exposure time of 2092 ms, a resolution setting with pixel size set to 9 microns, a rotation angle and range of 0.7° of a 360° rotation, and a frame averaging of 2.

Bruker Nrecon software was used to reconstruct projection images using a modified Feldkamp cone-beam algorithm. Image compensation settings were set to: smoothing = 3, beam hardening correction = 30%, ring artifact reduction = 20, thresholding = 0.00-0.06. *Bruker CTAn v1.16.4.1+* (SkyScan 2003-2011, Bruker microCT 2012-2016) software was used for analysis of the pores within the eggshells. These analyses included a 2D analysis of pore density and pore area, as well as a 3D analysis of pore volume and percentage porosity. All settings were optimised. An approximately 20.07 mm^2 (4.48 x 4.48 mm) region of interest was selected over a 1 mm thick volume of interest (VOI; approximately 100 slices). The region of interest (ROI) was placed in the centre of the specimen (Supplementary Figure 8) in order to minimise the effects of imaging artifacts, and potential weathering or erosion of pores close to the surfaces of the eggshell, on the analysis. The volume of interest was spread approximately 50 slices above and below the centre slice. For segmentation and analysis, a task list comprised of the following plug-ins was used: (1) global thresholding = 0-65, (2) despeckle (remove white speckles in 3D less than *x* voxels) = 400 voxels, (3) bitwise operation = Image + ROI, (4) despeckle (remove white speckles in 2D less than *x* pixels) = 15 pixels, (5) save bitmaps (save all 2D slices), (6) 3D analysis, (7) bitwise operation = NOT Image, and (8) 3D Analysis. Pore density was calculated as the total number of objects divided by the total area for the ROI. Average pore area was calculated as the mean of area of pores within the ROI. Total pore area was calculated as the sum of the pore areas within the ROI. Average pore volume was calculated as the total pore volume divided by the number of pores within the VOI. Percent porosity was calculated as the total pore volume as a percentage of the total VOI.

Images were visualised and rendered in the software *FEI Avizo Fire v8.1.1* (Konrad-Zeuse-Zentrum Berlin 1995-2014; FEI, SAS 1999-2014). The outer and inner surface views were volume rendered directly over the reconstructed slices. The pore structure surface view was performed on a 3D Model (.stl), which was created using the Marching Cubes 33 algorithm in the *Bruker CTAn* software. Reconstructed projection images, and rendered images are available online at DataDryad (see "Data availability statement" in the main text). Raw data is available upon request as image slice files are very large in size.

To compare pore dimensions between eggshell morphotypes, we tested several *a priori* hypotheses to avoid reducing statistical power through multiple comparisons. We began by examining differences in porosity because porosity is a function of pore volume (or pore area, as thickness is controlled in the VOI) and pore density. If there was found to be a difference in porosity between morphotypes, we went on to examine differences in pore volume/area, followed by pore density.

For each hypothesis, we first tested whether the residuals are normally distributed in PAST v3.11 (normality is an assumption of parametric statistical tests, such as ANOVA and t-tests). Tests for normality estimated Shapiro-Wilk, Anderson-Darling, and Jarque-Bera statistics. If the data did not conform to normality, non-parametric Mann-Whitney tests were employed. Tukey's pairwise comparisons were made where ANOVA suggested a significant effect. All tests were 2-tailed.
The hypotheses tested were:

(1) Does porosity differ between the eggshell 1.6-3 mm thick and the eggshell >3 mm thick from the South?
(2) Does porosity differ between the eggshell 1.6-3 mm thick and the eggshell <1.6 mm thick from the South?

(3) Does porosity differ between morphotypes?
(4) Does pore volume/area differ between morphotypes?
(5) Does pore density differ between morphotypes?

Results can be found in Supplementary Data 8 and Supplementary Table 11.

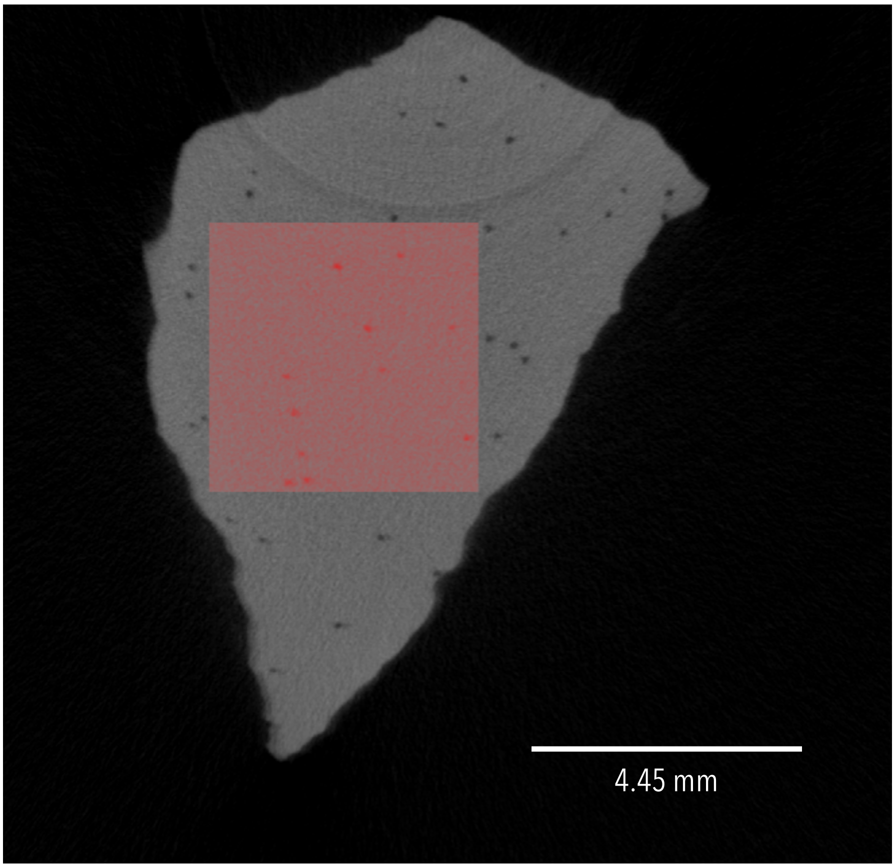

Supplementary Figure 8 An example of the 20.07 mm^2 (4.48 x 4.48 mm) region of interest (ROI) selected for both 2D and 3D analysis of pore density, area, volume, and porosity. The sample shown is AD#1666. The rings visible on the slice are artifacts created by the scanning process and are removed by the 'despeckling' plug-in.

Supplementary Table 11 Statistical comparisons (two-sided) employed to test for difference in various pore dimensions between morphotypes. The hypotheses tested are above (see Supplementary Note 7). Asterisks indicate the difference is significant at the 5%-level[1].

Hypothesis	Pairwise-comparison	Normality	Total df	Test statistic	p-value
1 Porosity	South >1.5<3 mm vs >3 mm	Non-normal p=0.012	9	Mann-Whitney Z=1.045	0.296
2 Porosity	South >1.5<3 mm vs <1.5 mm	Non-normal p=0.020	9	Mann-Whitney Z=2.089	0.037*
3 Porosity		Non-normal p=0.007			
	South >2 mm vs South <2 mm		14	Mann-Whitney Z=2.143	0.032*
	South >2 mm vs North		14	Mann-Whitney Z=0.061	0.951
	South <2 mm vs North		9	Mann-Whitney Z=2.089	0.037*
4 Pore volume/area		Non-normal p=0.016			
	South >2 mm vs South <2 mm		14	Mann-Whitney Z=1.286	0.198
	South >2 mm vs North		14	Mann-Whitney Z=0.061	0.951
	South <2 mm vs North		9	Mann-Whitney Z=1.462	0.144
5 Pore density		Normal p=0.17	19	ANOVA F=4.984	0.020*
	South >2 mm vs South <2 mm		14	Tukey's Q=3.785	0.019*
	South >2 mm vs North		14	Tukey's Q=0.079	0.957
	South <2 mm vs North		9	Tukey's Q=5.302	0.006*

There was no difference in porosity between eggshell 1.6-3 mm thick and above 3 mm thick in the South (p=0.3, Supplementary Table 11). Conversely, there was a significant difference in porosity between eggshell <1.6 mm thick and 1.6-3 mm thick (p=0.037, Supplementary Table 11). These results suggest that the eggshell 1.6-3 mm thick probably belong to the same taxon as the thick morphotype from the South (>3 mm thick) rather than the thin morphotype from the South (<1.6 mm thick), which is supported by the phylogenetic analyses. We concluded that the 1.6-3 mm thick from the South to simply be at those at the lower-tail-end of the "thick" morphotype's distribution of thicknesses, and further analysis of microstructure considered all eggshell >2 mm in thickness to be the same morphotype.

[1] Note that if a Bonferroni correction is applied where multiple comparisons were conducted, the threshold for significance becomes α=0.025, making some of the texts non-significant (Hypothesis 3 in Supplementary Table 11). However, this correction is extremely conservative, and since the difference in porosity is attributable to differences in pore density, we may only need to consider this variable instead. The family-wise error rate for comparisons of pore density (Hypothesis 5) is accounted for by Tukey's correction and thus these p-values remain significant.

The thick (>2 mm) and thin (<2 mm) morphotypes from the South significantly differ in porosity (*p*=0.03, Supplementary Table 11). This supports the idea that these morphotypes belong to two different taxa.

The thick (>2 mm) morphotype from the South and the medium (1.5-2.5 mm) morphotype from the North do not significantly differ in porosity (*p*=0.95, Supplementary Table 11). This supports the idea that these morphotypes belong to the same taxon.

The thin (<1.5 mm) morphotype from the South and the thin (>1.5 mm) morphotype from the North significantly differ in porosity (*p*=0.04, Supplementary Table 11). This supports the idea that these morphotypes belong to two different taxa.

Pore volume does not contribute to the difference in porosity observed between these morphotypes (*p*>0.14, Supplementary Table 11). However, pore density does contribute significantly to the difference in porosity observed between morphotypes (*p*=0.02, Supplementary Table 11).

Classification of the northern eggshell within the genus *Aepyornis* is further supported by the finding that there is no difference in porosity, average pore volume, or pore density (*p*>0.118; Figure 3) between northern eggshell and other Aepyornithid eggshell, but that there is a significant difference in porosity and pore density (*p*<0.006; Figure 3) between the northern eggshell and *Mullerornis* eggshell.

Supplementary Note 8
Stable isotope analysis

Prior to preparing eggshell (Supplementary Data 9) for isotopic analyses the fragments are mechanically cleaned by grinding, then reduced in mass by one third with the stoichiometric addition of 2N HCl *in vacuo* following procedures outlined in (Miller et al., 2005). For $\delta^{15}N_{org}$ and $\delta^{13}C_{org}$ an approximately 5-mg subsample was placed in a silver capsule and dissolved with three sequential 20-microliter aliquots of 6 N HCl-sequanal grade. The residual acid was evaporated at room temperature under a fume hood for approximately 24 hr; remaining water was removed in an oven at 85°C for 24 to 36 hr and stored at 50°C under N_2 until analysis. $\delta^{15}N_{org}$ and $\delta^{13}C_{org}$ were determined using an elemental analyzer (NC 2500; CE Elantech, Lakewood, NJ) interfaced with Thermo Finnigan (San Jose, CA) Delta Plus XL or Delta V Plus mass spectrometers (Carnegie Institution of Washington, Washington, DC). For $\delta^{13}C_{carb}$ and $\delta^{18}O$ approximately 0.1 to 0.2 mg of powdered eggshell was placed in an exetainer vial and flushed with helium to remove air in a *Thermo Fisher* Gas Bench. Six drops of 100% H_3PO_4 were added to each vial manually, then placed into a heating block at 70°C. For each batch of 40 to 80 eggshell powders we analyzed several blank vials and multiple standards, including internal lab standards along with NBS-18, NBS-19, and RO-22 (Isoanalytical). Evolved CO_2 was analyzed on Thermo Finnigan Delta XL Plus or Delta V Plus mass spectrometer. The $\delta^{18}O$ is measured in ‰ with respect to Standard Mean Ocean Water (SMOW) and converted to PDB; an internal carbonate standard analyzed six times had standard deviations of ±0.15‰ for $\delta^{18}O$ and ±0.25 for $\delta^{13}C_{calcite}$.

Isotopic data was also compiled from Hansford and Turvey (2022). To correct for stable isotope enrichment in eggshell from dietary sources and differences in atmospheric CO_2 between today and the past, 2‰ was subtracted from $\delta^{13}C_{org}$ for diet (Johnson et al. 1998) and a further 1.22‰ was subtracted from $\delta^{13}C_{org}$ for the Suess effect. 3‰ was subtracted from $\delta^{15}N_{org}$ for diet (Keeling 1979). ISOERROR v1.04 (Phillips and Gregg 2001) was used to calculate the relative contribution of C3 and CAM vegetation to diet using source values of $\delta^{13}C$ from Crowley et al. (2011), and 127 new plants collected from the southwest (Supplementary Data 9).

Diet corrected values of $\delta^{15}N_{org}$ and $\delta^{13}C_{org}$ were plotted against one another in PAST v3.11 (Figure 4), alongside nitrogen and carbon stable isotope values for a range of Madagascan plants published by Crowley et al. (2011). The centroid of these distributions ± 95% confidence intervals of the mean were also plotted.

Pairwise comparisons between each eggshell morphotype were made for each stable isotope (Supplementary Table 12). Tests for normally distributed residuals were estimated Shapiro-Wilk, Anderson-Darling, and Jarque-Bera statistics. If the data did not conform to normality, non-parametric Mann-Whitney tests were employed. Tukey's pairwise comparisons were made otherwise. All tests were 2-tailed.

PERMANOVAs (9999 permutations) were also used to compare $\delta^{15}N_{org}$, $\delta^{13}C_{org,}$ and $\delta^{18}O$ between eggshell morphotypes in a multivariate analysis (Supplementary Figure 9, Supplementary Figure 10). Significant differences in isotopic signature were found between all three eggshell morphotypes (F = 15.04, p-value = 0.0001; Supplementary Table 12). Comparing isotopic signature between eggshell >1.5<3 mm and >3 mm from the south showed no significant difference (Supplementary Figure 9, F = 2.251, p-value = 0.0906), supporting the idea that the medium-sized eggshell from the south belong to the same taxon as the thickest eggshells and simply belong to the left tail of the distribution of the "thick" morphotype.

Supplementary Table 12 Pairwise comparisons (two-sided) of each stable isotope between eggshell morphotypes. Asterisks indicate statistically significant differences in the mean stable isotope composition. The application of Bonferroni correction for multiple comparisons does not change the significance.

Stable isotope	Pairwise-comparison	Normality	*n*	Test statistic	p-value
Oxygen	South >2 mm vs South <2 mm	Non-normal $p<0.00001$	289	Mann-Whitney $Z=-8.0137$	1.11e-15*
	South >2 mm vs North >2 mm	Non-normal $p<0.00001$	228	Mann-Whitney $Z=-2.8311$	0.005*
	South <2 mm vs North	Non-normal $p<0.00001$	145	Mann-Whitney $Z=-2.669$	0.008*
Carbon (Calcite)	South >2 mm vs South <2 mm	Non-normal $P<0.0001$	289	Mann-Whitney $Z=-6.996$	2.64e-12*
	South >2 mm vs North	Normal $p=0.234$	228	Student's T =1.459	0.146
	South <2 mm vs North	Non-normal $P<0.0001$	145	Mann-Whitney $Z=-6.678$	2.44e-11*
Carbon (Organic)	South >2 mm vs South <2 mm	Non-normal $p<0.00001$	150	Mann-Whitney $Z=-2.595$	0.009*
	South >2 mm vs North >2 mm	Non-normal $P<0.0001$	170	Mann-Whitney Z=-5.943	2.78e-9*
	South <2 mm vs North	Non-normal $p<0.00001$	64	Mann-Whitney $Z=-4.994$	5.91e-7*
Nitrogen (Organic)	South >2 mm vs South <2 mm	Normal $p>0.18$	90	Tukey's $Q=6.401$	3.82e-5*
	South >2 mm vs North	Normal $p>0.6$	110	Tukey's $Q=5.99$	8.63e-5*
	South <2 mm vs North	Normal $p>0.18$	64	Tukey's $Q=0.411$	0.955
Multivariate Isotopic signature	South >2 mm vs South <2 mm	NA		PERMANOVA $F=11.91$	0.0001*
	South >2 mm vs North	NA		PERMANOVA $F=24.89$	0.0001*
	South <2 mm vs North	NA		PERMANOVA $F=4.886$	0.0065*

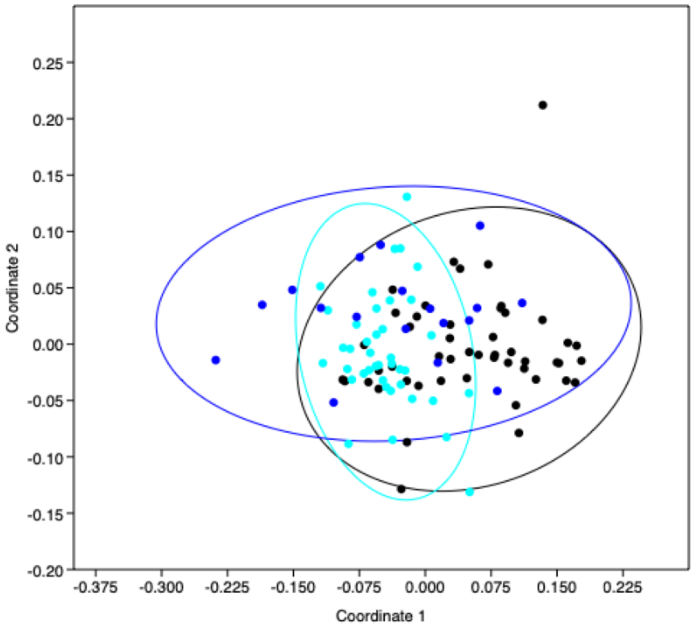

Supplementary Figure 9 NMDS plot of $\delta^{15}N_{org}$, $\delta^{13}C_{org}$, and $\delta^{18}O$ stable isotopes from eggshell found in the north (light blue), south with a thickness <1.5 mm (dark blue), and south >1.5 mm (black).

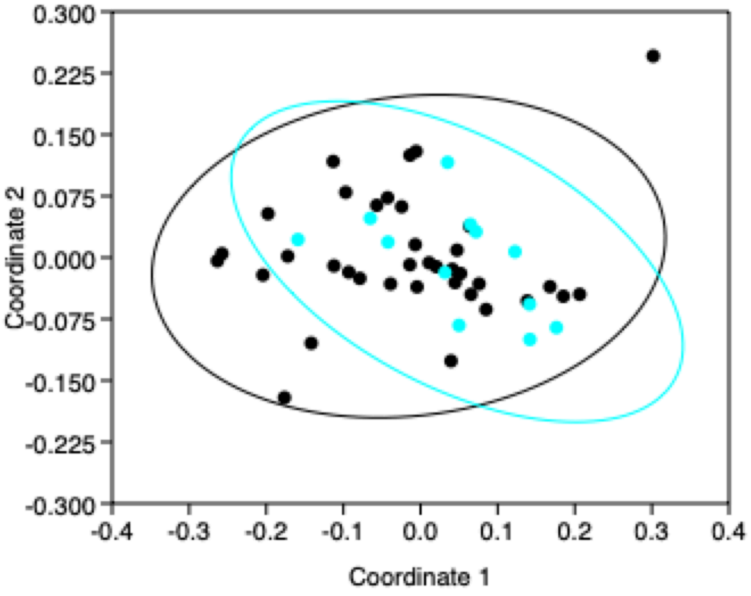

Supplementary Figure 10 NMDS plot of $\delta^{15}N_{org}$, $\delta^{13}C_{org}$, and $\delta^{18}O$ stable isotopes from eggshell found in the south with a thickness >1.5<3 mm (light blue), and south with a thickness >3 mm (black).

Supplementary Note 9
Body size estimation and ancestral state reconstruction

All statistical analyses were performed in R v.4.2.0 (R Core Team, 2022) on log-transformed data (e.g. Gingerich, 2000) and archived as a Markdown file (Supplementary Code 1; see also DataDryad (see "Data availability statement" in the main text). Estimates of egg mass and body mass for eggshell specimens were obtained using phylogenetic generalized least squares (PGLS; Grafen, 1989; Symonds and Blomberg, 2014) regressions of eggshell thickness (in µm) on egg mass and body mass (both in g), respectively (see model 1 and 2). The sample for these analyses (n = 65) includes the elephant bird eggshell specimens from the molecular phylogeny (i.e., the best representative of each clade), as well as 14 palaeognaths and 45 neognaths sampled from the literature. Measurements for all three traits of interest were taken from Legendre and Clarke (2021), except for three palaeognath species: *Apteryx haastii* (eggshell thickness: Vieco-Galvez et al., 2021; egg mass: Body and Reid, 1987), *Apteryx owenii*, and *Casuarius bennetti* (values of eggshell thickness and egg mass taken from Schönwetter, 1960–1992, for both species; see also Silyn-Roberts and Sharp, 1985, for *C. bennetti*). Values taken from Schönwetter (1960-1992) were not measured directly, but derived from equations that use egg dimensions and shell weight as predictors (see Maurer et al., 2010). Eggshell thickness values for *Rhea pennata* were taken from Sales (2006), and for *Anomalopteryx didiformis* from Gill (2021). and Body mass information for all three species was taken from Dunning (2008); an estimate for the body mass of *Aepyornis hildebrandti* was taken from Crouch and Clarke (2019). The phylogeny used in all analyses was pruned from the tree of Legendre and Clarke (2021), with information for topology and divergence times taken from the molecular phylogeny presented here (Figure 2).

In a second analysis, all body masses for large extant flightless palaeognaths (n = 9) were replaced with those of male members of the species to account for incubation constraints, since in all these species the males incubate the eggs (e.g. Huynen et al., 2010). Male body masses were taken from Olson and Turvey (2013). The body mass for *Casuarius bennetti*, for which no male body mass data were available, was left as the average body mass. Additionally, estimates of body mass for fossil taxa were removed from the analysis in order to estimate body mass in elephant birds solely from extant bird data (see model 3 and 4).

For both PGLS models (eggshell thickness ~ egg mass and eggshell thickness ~ body mass), several correlation structures corresponding to different evolutionary models were fitted using corresponding functions in 'ape' (Paradis and Schliep, 2019) and 'nlme' (Pinheiro et al., 2022), and ranked using the Aikaike Information Criterion corrected for small sample sizes (AICc – Burnham and Anderson, 2002) in AICcmodavg (Mazerolle, 2020). Tested evolutionary models (see e.g. Benoit et al., 2019) include Brownian Motion, Pagel's Lambda, Ornstein-Uhlenbeck, Early Burst, and White Noise (i.e. non-phylogenetic ordinary least squares regression). For the model with egg mass as predictor, a Lambda model ($\lambda = 0.82$) was selected as the best fit; for the one with body mass as predictor, a white noise model was selected. For the lambda model, a pseudo R-squared (R_{pred}) was compiled using 'rr2' (Ives and Li, 2018) to estimate effect size, as standard R-squared cannot be compiled for GLS models (Ives, 2019). Both models show a very high fit (egg mass as predictor: $R_{pred} = 0.96$; body mass as predictor: $R^2 = 0.95$; $p < 2.2e\text{-}16$).

Model 1 $\ln(eggshell\ thickness\ um) = (0.439850 * \ln(egg\ mass\ g)) + 4.042591$

Model 2 $\ln(eggshell\ thickness\ um) = (0.339752 * \ln(body\ mass\ g)) + 3.400739$

Model 3 $\ln(eggshell\ thickness\ um) = (0.443596 * \ln(egg\ mass\ g)) + 4.013223$

Model 4 $\ln(eggshell\ thickness\ um) = (0.341402 * \ln(bodymass\ g)) + 3.382209$

Ancestral state reconstructions (ASR) following a Brownian Motion model were compiled for our sample of palaeognaths for eggshell thickness, egg mass, and body mass, using contMap in 'phytools v1.2-0' (Revell, 2012, 2013): First, egg mass and body mass for elephant birds was estimated using PGLS models 1 and 2 above, and ASR was performed using the tree topology and divergence times from Figure 2 (n = 6, including the three eggshell samples that represented the best genome data from each clade, and the bone specimens

for which eggshell thickness was inferred to be the average from eggshell specimens in each clade) (see Supplementary Figure 11). Second, egg mass and body mass for elephant bird specimens was estimated using PGLS models 3 and 4: (I) using the average eggshell thickness for each taxon with ASR performed including only one representative from each taxon in the tree (Supplementary Figure 12Ia-c); (II) using the true eggshell thickness for specimens with ASR performed including three elephant bird specimens per taxon that cover the distribution of thicknesses (i.e., one at the left tail, one at the center, and one at the right tail of the thickness distribution) (Supplementary Figure 12IIa-c), and; (III) using the true eggshell thickness for eggshell specimens and the average eggshell thickness for bone specimens with ASR performed including the three eggshell samples that represented the best genome data from each clade, and the bone specimens for which eggshell thickness was inferred to be the average from eggshell specimens in each clade (Supplementary Figure 12IIIa-c).

The choice of a Brownian Motion model was justified by the high phylogenetic signal ($\lambda > 0.8$) for all traits of interest (see Supplementary Code 1). Brownian Motion and Lambda models were the best fit over OU, EB and null models. Ancestral state was also reconstructed using StableTraits v 1.4 (Elliot and Mooers 2014) (see https://mickelliot.com), but results were similar. Given that flight has been independently lost among multiple lineages of palaeognaths, it is likely that traits such as large body mass and egg mass have also converged; thus, most models may be inaccurate for predicting ancestral state between such lineages, resulting in inflated estimates of body and egg mass (that do not make biological sense for a volant common ancestor). However, estimates of ancestral state within each convergently flightless clade (such as within elephant birds) may be more accurate. Ancestral state reconstructions were also performed with different topologies (i.e., rheas deepest among notopalaeognathae), and with the inclusion of neognath data; results were similar (see Supplementary Code 1).

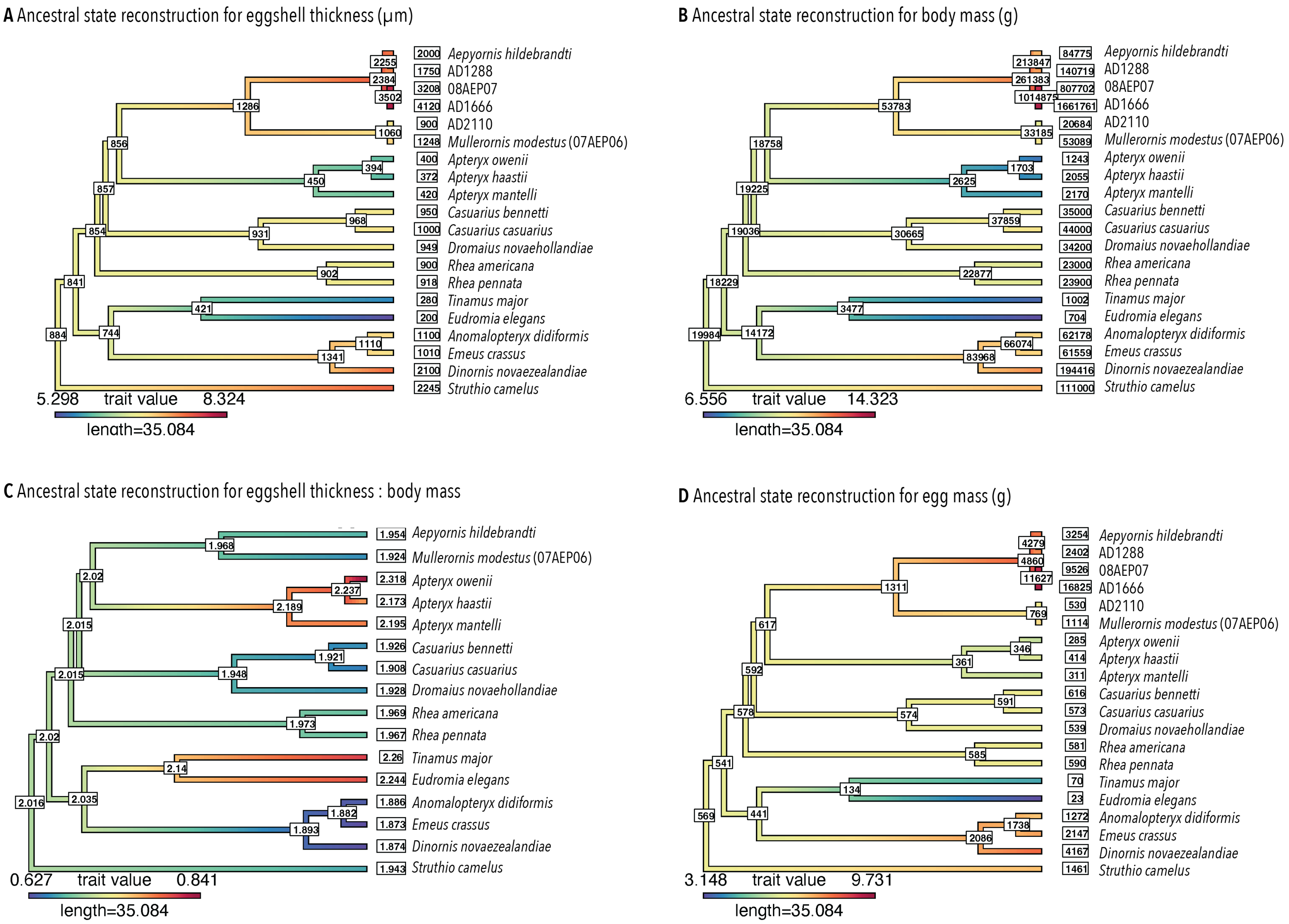

Supplementary Figure 11 Ancestral state reconstructions for **A** eggshell thickness, **B** body mass, **C** the ratio of eggshell thickness to body mass, and **D** egg mass. Estimates of body mass were generated with a PGLS model of eggshell thickness vs average body mass of 45 neognaths and 14 palaeognaths, including *A. hildebrandti* and *Mullerornis modestus*, as described above (model 1 and 2).

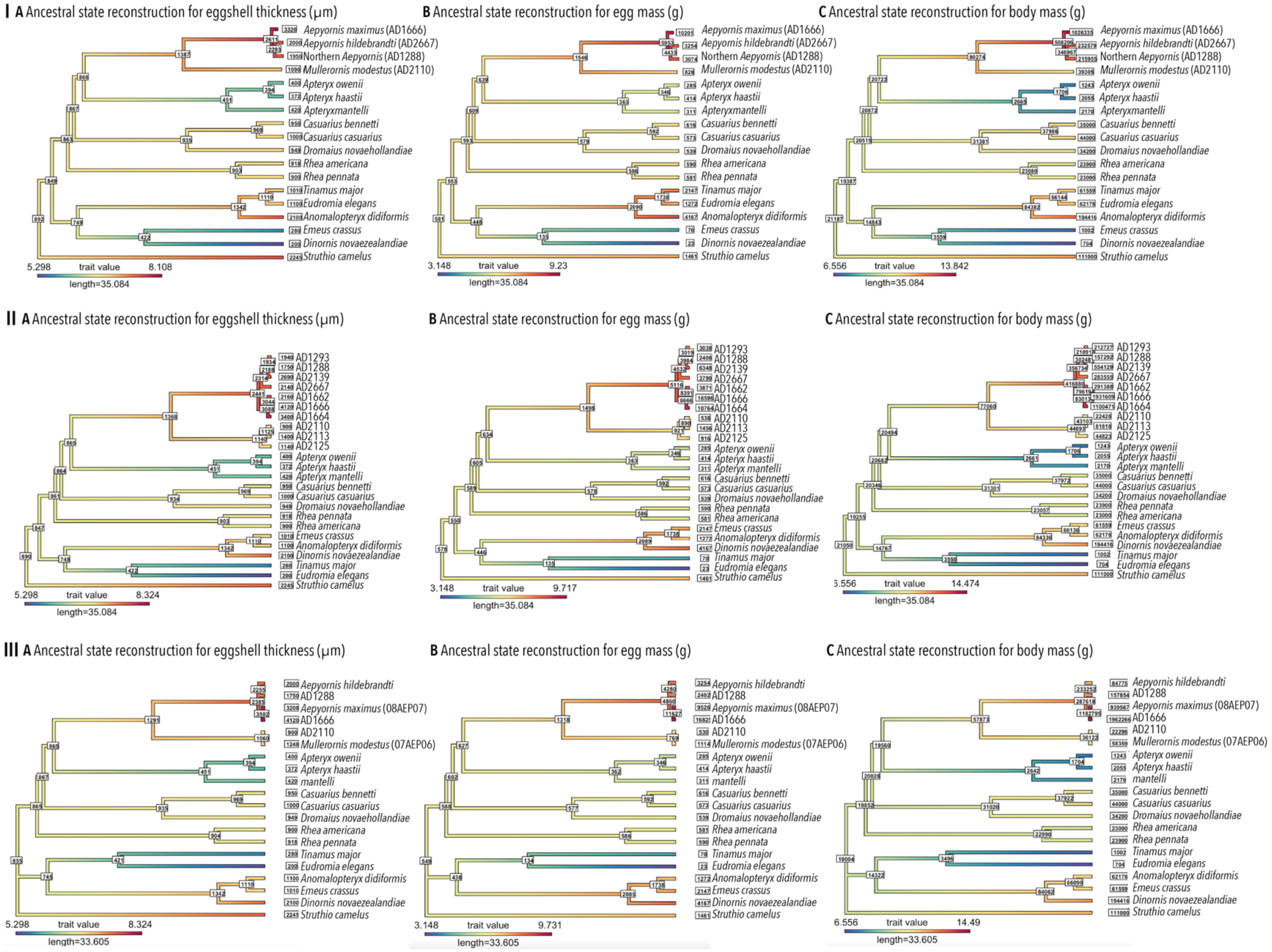

Supplementary Figure 12 Ancestral state reconstructions for **A** eggshell thickness, **B** egg mass, and **C** body mass. Estimates of body mass were generated with a PGLS model of eggshell thickness vs average body mass of the incubating bird (males in palaeognaths), and excluding elephant birds as known values in the model (model 3 and 4 above). Ancestral states are derived from inputs including: **I** one specimen per elephant bird taxon representing the average values for that taxon; **II** three specimens per elephant bird taxon—one at the lower-end of the thickness distribution, one in the centre, and one at the higher-end of the thickness distribution, and; **III** three eggshell and three bone specimens (see Figure 2b) where the thickness for the bone specimens is given the average value for the taxon. Note that ancestral states and body mass estimates reported in the main text are derived from **I**.

Supplementary Note 10
Yield point value

Using the thickness of the eggshell to estimate the force an egg can withstand without breaking (or the "yield point value", *F*; Ar et al. 1979), we find that it would take upwards of 177.33–285.66 kg to fracture an average-sized (3.36 mm thick) *Aepyornis* egg from the south—far less than the mass (*M*) of even the smallest *A. maximus* at 334 kg. The Safety Factor $S = \left(\frac{F}{M}\right) - 1$ using the average mass of *Vorombe* (642.9 kg; Hansford and Turvey 2018) is -0.68—comparable to that of the female members of the moa genera *Emeus*, *Pachyornis* and *Euryapteryx* (Huynen et al. 2010), while *S* estimated using the average mass of *A. maximus* (409.5 kg; Hansford and Turvey 2018) is -0.5, comparable to the male members of these same genera. With every *S* value decrease of 0.1 unit doubling the likelihood of egg breakage, the possibility of the average *Vorombe* being able to incubate an egg without breaking it is low, supporting the idea that *Vorombe* may be females of *A. maximus,* and that, like most other ratites, the male of the species incubated the eggs. However, estimates of yield point force do not take into account that force may be spread among a clutch of eggs, reducing the pressure on individual eggs. It also doesn't consider whether a nest, mound or depression in the earth may support the weight of the incubating bird, or that the bird is unlikely to place its entire resting body weight on its eggs—potentially only a brood patch near the breast is in contact with the eggs, or the bird's mass is supported by its legs. This hypothesis is further supported by our calculation that the mass of the bird that laid such thick eggshell was approximately the size of *Vorombe*, whereas a bird the size of *A. maximus* would have been expected to lay a much thinner egg.

Supplementary references

1. Altschul, S. *et al.* Basic local alignment search tool. *Journal of Molecular Biology* **215**, 403-410 (1990).

2. Ar, A., *et al*. The avian egg: mass and strength. *Condor* **81**, 331-337 (1979).

3. Benoit, J. *et al.* Brain evolution in Proboscidea (Mammalia, Afrotheria) across the Cenozoic. *Sci Rep* **9**, 9323 (2019).

4. Benson, D. A. *et al*. GenBank. *Nucleic Acids Research* **34**, D16-D20, doi:10.1093/nar/gkj157 (2006).

5. Body, D.R. & Reid, B. The lipid, fatty acid and amino acid composition of ratite eggs from three different species of kiwis *Apteryx australis mantelli*, *A. haasti* and *A. oweni* bred in captivity on the same diet. *Biochemical Systematics and Ecology* **15**, 625–628 (1987).

6. Brooks, A. S. *et al*. Dating Pleistocene archaeological sites by protein diagenesis in ostrich eggshell. *Science* **248**, 60-64 (1990).

7. Burnham, K. P. & Anderson, D. R. *Model selection and multimodel inference: a practical information-theoretic approach.* (Springer, 2002).

8. CALIB 5.0 (2005).

9. Castresana, J. Selection of conserved blocks from multiple alignments for their use in phylogenetic analysis. *Molecular Biology and Evolution* **17**, 540-552 (2000).

10. Cooper, A. *et al.* Complete mitochondrial genome sequences of two extinct moas clarify ratite evolution. *Nature* **409**, 704-707 (2001).

11. cRAP protein sequences [online], 2019. Available from: https://www.thegpm.org/crap/ [Accessed 1 Mar 2019].

12. Crisp, M., Demarchi, B., Collins, M., Morgan-Williams, M., Pilgrim, E., Penkman, K. Isolation of the intra-crystalline proteins and kinetic studies in *Struthio camelus* (ostrich) eggshell for amino acid geochronology. *Quaternary geochronology* **16**, 110–128 (2013).

13. Crouch, N.M.A. & Clarke, J.A. Body size evolution in palaeognath birds is consistent with Neogene cooling-linked gigantism. *Palaeogeography Palaeoclimatolology Palaeoecology* **532**, 109224 (2019).

14. Crowley, B. E. *et al.*. Explaining geographical variation in the isotope composition of mouse lemurs (*Microcebus*). *Journal of Biogeography* **38**, 2106-2121 (2011).

15. Dabney, J. *et al.* Complete mitochondrial genomes of a middle Pleistocene cave bear reconstructed from ultrashort DNA fragments. *Proc. Natl. Acad. Sci.* **110**, 15758-15763 (2013).

16. Darriba, D. *et al.* jModelTest 2: more models, new heuristics and parallel computing. *Nat Methods* **9**, 772 (2012).

17. Damgaard, P. B., Margaryan, A., Schroeder, H., Orlando, L., Willerslev, E. & Allentoft, M. E. Improving access to endogenous DNA in ancient bones and teeth. *Scientific Reports* **5**, 11184 (2015).

18. Delaunay, A. D., Robin, C., Guillocheau, F., Dall'Asta, M. & Calves, G. Mid to Late Miocene uplift and doming of Madagascar: constraints from topology, Cenozoic stratigraphy and paleogeography. *Third EAGE Eastern Africa Petroleum Geoscience Forum* Nov 2017, p.1-2 (2017).

19. De Los Monteros, A.E. Models of the primary and secondary structure for the 12S rRNA of birds: a guideline for sequence alignment. *DNA sequence,* **14**, 241-256 (2003).

20. Demarchi, B. *et al.* Protein sequences bound to mineral surfaces persist into deep time. eLife **5**:e1709.

21. Demarchi, B., Stiller, J., Grealy, A., Mackie, M., Deng, Y. et al. Ancient proteins resolve controversy over the identity of *Genyornis* eggshell. *Proceedings of the National Academy of Sciences* https://doi.org/10.1073/pnas.2109326119 (2022).

22. do Amaral, F.R. *et al.* Towards and assessment of character interdependence in avian RNA phylogenetics: a general secondary structure model for the avian mitochondrial 16s rRNA. *Molecular Phylogenetics and Evolution* **56**, 498-506 (2010).

23. Dunning, J.B. *CRC Handbook of Avian Body Masses, Second Edition*. CRC Press, Boca Raton (2008).

24. Edgar, R. C. Search and clustering orders of magnitude faster than BLAST. *Bioinformatics* **26**, 2460-2461 (2010).

25. Edgar, R. C. MUSCLE: multiple sequence alignment with high accuracy and high throughput. *Nucleic Acids Research* **32**, 1792-1797 (2004).

26. Elliot, M. G., & Moors, A. Ø. Inferring ancestral states without assuming neutrality or gradualism using a stable model of continuous character evolution. *BMC Evolutionary Biology* **14**, 226 (2014).

27. Field, D. J., Benito, J., Chen, A., Jagt, J. W. M., Ksepka, D. T. Late Cretaceous neornithine from Europe illuminates the origins of crown birds. *Nature* **579**, 397-401 (2020).

28. Gansauge, M.T. & Meyer, M. Single-stranded DNA library preparation for the sequencing of ancient or damaged DNA. *Nature Protocols* **8**, 737-748 (2013).

29. Grealy, A. *et al.* Eggshell palaeogenomics: palaeognath evolutionary history revealed through ancient nuclear and mitochondrial DNA from Madagascan elephant bird (*Aepyornis* sp.) eggshell. Molecular Phylogenetics and Evolution **109**, 151-163 (2017).

30. Gill, B. J. Thickness histograms of Holocene fossil eggshell fragments indicate diversity and relative abundance of moas (Aves: Dinornithiformes) at North Island sites. *New Zealand Journal of Zoology* **49**, 143-165 (2021).

31. Gingerich, P. D. Arithmetic or geometric normality of biological variation: an empirical test of theory. *J Theor Biol* **204**, 201–221 (2000).

32. Ginolhac, A. *et al*. mapDamage: testing for damage patterns in ancient DNA sequences. *Bioinformatics* **27**, 2153-2155 (2011).

33. Grafen, A. The Phylogenetic Regression. *Philos Trans R Soc B* **326**, 119–157 (1989).

34. Guindon, S. & Gascuel, O. A simple, fast and accurate method to estimate large phylogenies by maximum-likelihood. *Syst Biol* **52**, 696-704 (2003).

35. Haddrath, O. & Baker, A. J. Complete mitochondrial DNA genome sequences of extinct birds: ratite phylogenetics and the vicariance biogeography hypothesis. *Proceedings of the Royal Society B-Biological Sciences* **268**, 939-945 (2001).

36. Hammer, Ø. *et al*. PAST: Paleontological statistics software package for education and data analysis. *Palaeontologia Electronica* **4**, 9 (2001).

37. Hansford, J. P. & Turvey ST. Unexpected diversity within the extinct elephant birds (Aves: Aepyornithidae) and a new identity for the world's largest bird. *R. Soc. Open. Sci.* **5**, 181295. http://dx.doi.org/10.1098/rsos.181295 (2018).

38. Hansford, J. P. & Turvey, S. T. Dietary isotopes of Madagascar's extinct megafauna reveal Holocene browsing and grazing guilds. *Biology Letters* **18**, 4, 20220094 (2022).

39. Hogg, A. *et al*. SHCAL13 Southern Hemisphere calibration, 0-50,000 years cal BP. *Radiocarbon* **55**, 1-15 (2013).

40. Huelsenbeck, J. P. & Ronquist, F. MrBayes: Bayesian inference of phylogenetic trees. *Bioinformatics* **17**, 754-755 (2001).

41. Huson, D. *et al.* MEGAN analysis of metagenomic data. *Genome Research* **17**, 377-386 (2007).

42. Huynen, L. *et al*. Ancient DNA reveals extreme egg morphology and nesting behavior in New Zealand's extinct moa. *Proceedings of the National Academy of Sciences*, **107** (37), 16201-16206 (2010).

43. Huynen, L. *et al*. Complex species status for extinct moa (Aves: Dinornithiformes) from the genus *Euryapteryx*. *Plos One* **9**, e90212 (2014).

44. Ives, A. R. & Li, D. rr2: An R package to calculate R^2s for regression models. *J Open Source Softw* **3**, 1028 (2018).

45. Ives, A. R. R^2s for correlated data: phylogenetic models, LMMs, and GLMMs. *Syst Biol* **68**, 234–251 (2019).

46. Jumper, J., Evans, R., Pritzel, A., Green, T., Figurnov, M., *et al*. Highly accurate protein structure prediction with AlphaFold. *Nature* **doi**:10.1038/s41586-021-03819-2 (2021).

47. Jonsson, H. *et al*. mapDamage2.0: fast approximate Bayesian estimates of ancient DNA damage parameters. *Bioinformatics* **29**, 1682-1684 (2013).

48. Johnson, B. J., Fogel, M. L. & Miller, G. H. Stable isotopes in modern ostrich eggshell: a calibration for paleoenvironmental applications in semi-arid regions of southern Africa. *Geochim, Cosmochim. Acta* **62**, 2451–2461 (1998)

49. Katoh, K. *et al*. MAFFT: a novel method for rapid multiple sequence alignment based on fast Fourier transform. *Nucleic Acids Research* **30**, 3059-3066 (2002).

50. Kearse, M. *et al*. Geneious Basic: an integrated extendable desktop software platform for the organization and analysis of sequence data. *Bioinformatics* **28** (2012).

51. Keeling, C. D. The Suess effect: 13Carbon-14Carbon interrelations. *Environ. Int.* **2**, 229–300 (1979).

52. Ksepka, D. T., Stidham, T. A., & Williamson, T. E. Early Paleocene landbird supports rapid phylogenetic and morphological diversification of crown birds after the K–Pg mass extinction. *Proceedings of the National Academy of Sciences* 114 (30), 8047-8052 (2017).

53. Kimura, M. A simple method for estimating evolutionary rate of base substitutions through comparative studies of nucleotide sequences. *Journal of Molecular Evolution* **16**, 111-120 (1980).

54. Knapp, M. *et al*. Setting the stage—building and working in an ancient DNA laboratory. *Annals of Anatomy-Anatomischer Anzeiger* **194**, 3-6 (2012).

55. Kumar, S. *et al*. MEGA X: Molecular Evolutionary Genetics Analysis across computing platforms. *Molecular Biology and Evolution* **35**, 1547-1549 (2018).

56. Laslett, D. & Canback, B. ARWEN: a program to detect tRNA genes in metazoan mitochondrial nucleotide sequences. *Bioinformatics* **24**, 172-175 (2008).

57. Legendre, L.J. & Clarke, J.A. Shifts in eggshell thickness are related to changes in locomotor ecology in dinosaurs. *Evolution* **75**, 1415-1430 (2021).

58. Liu, J., Ding, Q. & Gao, L. The complete mitochondrial genome of North Island brown kiwi *Apteryx mantelli*. *Mitochondrial DNA Part B* **2**, 1-2 (2016)

59. Ma, B. *et al*., PEAKS: powerful software for peptide de novo sequencing by tandem mass spectrometry. *Rapid communications in mass spectrometry: RCM* **17**, 2337–2342 (2003).

60. Mann, K. Identification of the major proteins of the organic matrix of emu (*Dromaius novaehollandiae*) and rhea (*Rhea americana*) eggshell calcified layer. *British poultry science* **45**, 483–490 (2004).

Mann, K., Siedler, F. Ostrich (*Struthio camelus*) eggshell matrix contains two different C-type lectin-like proteins. Isolation, amino acid sequence, and posttranslational modifications. *Biochimica et biophysica acta 1696*, 41–50 (2004).

61. Masters, B., Fan, V. & Ross, H. Species delimitation—a Geneious plugin for the exploration of species boundaries. *Molecular Ecology Resources* **11**, 154-157 (2010).

62. Maurer, C., Russell, D.G.D. & Cassey, P. Interpreting the Lists and Equations of Egg Dimensions in Schönwetter's *Handbuch Der Oologie*. *The Auk* **127**, 940–947 (2010).

63. Mazerolle, M. *AICcmodavg: Model Selection and Multimodel Inference Based on (Q)AIC(c)*. (2020).

64. Miller, G. H., Hart, C. P., Roark, E. B. & Johnson, B. J. Isoleucine epimerization in eggshells of the flightless Australian birds *Genyornis* and *Dromaius*. in *Perspectives in amino acid and protein geochemistry* 161-181, Oxford University Press, New York (2000).

65. Miller, G. H. *et al*. Ecosystem collapse in Pleistocene Australia and a human role in megafaunal extinction. *Science* **309**, 287-290 (2005).

66. Miller, M. *et al*. Creating the CIPRES Science Gateway for inference of large phylogenetic trees. *Proceedings of the Gateway Computing Environments Workshopt (GCE)*, 1-8 (2010).

67. Mitchell, K. *et al.* Ancient DNA reveals elephant birds and kiwi are sister taxa and clarifies ratite bird evolution. *Science* **344**, 898-900 (2014).

68. Monnier, L. Paléontologie de Madagascar VII, Les Aepyornis. *Ann. Paléontol.* **8**, 125–172 (1913).

69. Olson, V. A. & Turvey, S. T. The evolution of sexual dimorphism in New Zealand giant moa (Dinornis) and other ratites. *Proceedings of the Royal Society B: Biological Sciences* **280**, 20130401 (2013).

70. Paradis, E. & Schliep, K. ape 5.0: an environment for modern phylogenetics and evolutionary analyses in R. *Bioinformatics* **35**, 526–528 (2019).

71. Phillips, M. J., Gibb, G. C., Crimp, E. A., Penny, D. Tinamous and Moa Flock Together: Mitochondrial Genome Sequence Analysis Reveals Independent Losses of Flight among Ratites. *Systems Biology* **59**, 90-107 (2010).

72. Phillips, M. *et al.* Family-level relationships among the Australasian marsupial "herbivores" (Diprotodontia: Koala, wombats, kangaroos and possums). *Molecular Phylogenetics and Evolution* **46**, 594-605 (2008).

73. Phillips, D. L. & Gregg, J.W. Uncertainty in source partitioning using stable isotopes. *Oecologia1* **27**, 171–179 (2001).

74. Pinheiro, J. *et al. nlme: Linear and Nonlinear Mixed Effects Models*. (2022).

75. Posada, D. & Crandall, K. A. MODELTEST: testing the model of DNA substitution. *Bioinformatics* **14**, 817-818 (1998).

76. R Core Team. *R: A language and environment for statistical computing*. (R Foundation for Statistical Computing, 2022).

77. Rambaut, A. & Drummond, A. J. TreeStat v1.2: Tree statistic calculation tool. Version 1.2 (2007).

78. Rambaut, A. *et al.* Tracer: MCMC Trace Analysis Tool v. 1.6.1pre (2003).
Revell, L. J. phytools: an R package for phylogenetic comparative biology (and other things). *Methods Ecol Evol* **3**, 217–223 (2012).

79. Revell, L. J. Two new graphical methods for mapping trait evolution on phylogenies. *Methods Ecol Evol* **4**, 754–759 (2013).

80. Ruokonen, M. K. L. Structure and evolution of the avian mitochondrial control region. *Molecular Phylogenetics and Evolution* **2**, 422-432 (2002).

81. Sales, J. The rhea, a ratite native to South America. *Avian and Poultry Biology Reviews* **17**, 105-124 (2006).

82. Schönwetter, M. *Handbuch der Oologie*. Akademie Verlag, Berlin. (1960-1992).

83. Shapiro, B. & Hofreiter, M. *Ancient DNA: methods and protocols*. New York Humana Press (Springer Science) (2012).

84. Silyn-Roberts, H. & Sharp, R.M. Preferred orientation of calcite in the ratite and tinamou eggshells. *Journal of Zoology* **205**, 39–52 (1985).

85. Stecher, G., Tamura, K., & Kumar, S. Molecular Evolutionary Genetics Analysis (MEGA) for macOS. *Molecular Biology and Evolution* **37**, 1237-1239 (2020).

86. Steinegger, M., Ovchinnikov, S., Mirdita, M. AlphaFold2 w/ MMseqs2 [WWW Document]. Google Colaboratory.
URL https://colab.research.google.com/github/sokrypton/ColabFold/blob/main/AlphaFold2.ipynb (accessed 25/06/22) (2022).

87. Stuiver, M. & Reimer, P. J. Extended 14C data base and revised CALIB 3.0 14C age calibration program. *Radiocarbon* **35**, 215-230 (1993).

88. Swofford, D. L. PAUP* v. 4.0b (Sinauer Associates, Sunderland, Massachusetts, 2003).

89. Symonds, M. R. E. & Blomberg, S. P. A Primer on Phylogenetic Generalised Least Squares. in *Modern Phylogenetic Comparative Methods and Their Application in Evolutionary Biology: Concepts and Practice* (ed. Garamszegi, L. Z.) 105–130 (Springer, 2014).

90. Talavera, G. & Castresana, J. Improvement of phylogenies after removing divergent and ambiguously aligned blocks from protein sequence alignments. *Systematic Biology* **56**, 564-577 (2007).

91. Turvey, S. T., & Holdaway, R. N. Postnatal ontogeny, population structure, and extinction of the giant moa *Dinornis*. *Journal of Morphology* **265**, 70-86 (2005).

92. Vieco-Galvez, D., Castro, I., Morel, P.C.H., Chua, W.H. & Loh, M. The eggshell structure in *Apteryx*; form, function, and adaptation. *Ecology and Evolution* **11**, 3184–3202 (2021).

93. Wehmiller, J. Interlaboratory comparison of amino acid enantiomeric ratios in Pleistocene fossils. *Quaternary Geochronology* **16**, 173-182 (2013).

94. Willerslev, E. & Cooper, A. Ancient DNA. *Proceedings of the Royal Society B-Biological Sciences* **272**, 3-16 (2005).

95. Yang, Z. *et al*. Bayesian estimation of species divergence times under a molecular clock using multiple fossil calibrations with soft bounds. *Mol Biol Evol* **23** (2006).
Yang, Z. PAML 4: a program package for phylogenetic analysis by maximum likelihood. *Mol Biol Evol* **24**, 1586-1591 (2007).

96. Yonezawa, T. *et al*. Phylogenomics and morphology of extinct palaeognaths reveal the origin and evolution of the ratites. *Curr Biol* **27**, 1-10 (2016).

97. Zhang, J., Xin, L., Shan, B., Chen, W., Xie, M., Yuen, D., Zhang, W., Zhang, Z., Lajoie, G.A., Ma, B. PEAKS DB: de novo sequencing assisted database search for sensitive and accurate peptide identification. *Molecular & cellular proteomics* **11**, M111.010587 (2012).
