# Supplementary material for: Molecular exploration of fossil eggshell uncovers hidden lineage of giant extinct bird
Source: Nat Commun. 2023 Feb 28;14:914. doi: 10.1038/s41467-023-36405-3 (PMC9974994; doi:10.1038/s41467-023-36405-3)
Supplement: Supplementary file 3 — Description of Additional Supplementary Files [file 41467_2023_36405_MOESM3_ESM.pdf]

Description of Additional Supplementary Files

Supplementary Data 1-4 Sample_Metadata_and_eggshell_thickness

Supplementary **Data 1**

Localities described in the literature where elephant bird fossils have been found.

Supplementary **Data 2**

A Thickness measurements of eggshell fragments.

B Estimations of egg mass and laying bird mass from the mean eggshell thickness and regression described in Supplementary Note 9.

C Summary statistics for the distribution of eggshell thicknesses for each morphotype, and predicted egg mass and body masses.

Supplementary **Data 3**

Radiocarbon dated eggshell specimens. AI refers to amino acid racemisation Ile ratio.

Supplementary **Data 4**

A Summary table of the reads lost during quality control, the number of reads mapped and their statistics, and the average coverage of the mitochondrial genome across the number of base-pairs retrieved for each sample.

B Summary table of the reads lost during quality control, the number of reads mapped and their statistics, and the average coverage of the mitochondrial genome across the number of base-pairs retrieved for each library, including those that were excluded from subsequent analysis due to poor quality.

Supplementary Data 5 Summary CO1

Estimates of average evolutionary divergence in a ~600 bp barcoding region of cytochrome oxidase 1 between sequences both within groups and between groups. Analyses were conducted using the Kimura 2-parameter model. Codon positions included were 1st+2nd+3rd+Noncoding. All ambiguous positions were removed for each sequence pair. Evolutionary analyses were conducted in MEGA7.

Within species
Between species within genus
Between genera within family
Between families within order
Between orders
Elephant birds
Rheas

Kiwis
Cassowaries
Emus
Casuariiformes
Dinornis
Pachyornis
Euryapteryx
Emeus
Anomalopteryx
Emeidae
Dinornithiformes
Ratites

Supplementary Data 6 Molecular Dating

Comparison of molecular divergence times derived from both autocorrelated ("auto") and independent ("ind") rates models, for two topologies, (one where rheas ("_RH") are at the base of notopalaeognathae, and one where tinamous and moas are at the base of notopalaeognathae ("_TM"). The blue columns are those published in Figure 2 of the main text.

Supplementary Data 7 Samples used for protein analysis

Eggshell specimens used for protein analysis and summary statistics for the eggshell-specific C-type lectins retrieved from 12 samples.

Supplementary Data 8 CT data

Eggshell specimens imaged with micro-CT. Features of eggshell microstructure including various pore dimensions, within the region and volume of interest (4.48 * 4.48 * 1 mm), determined through micro-CT analysis.

Supplementary Data 9 Isotope data

A Carbon, nitrogen and oxygen stable isotope data for the different morphotypes of eggshell.

B Summary statistics for carbon, nitrogen and oxygen stable isotope data for the different morphotypes of eggshell and bone.

C Carbon and nitrogen stable isotope data for Madagascan plants.

D Summary statistics for carbon and nitrogen stable isotope data for Southwestern Madagascan plants.

Supplementary Data 10 Genetic data minus filtered reads

Filtered reads can be found on NCBI's Short Read Archive. The remaining folders contain the reference used for mapping, mapping files, reconstructed mitochondrial genomes, mitochondrial alignments used for phylogenetic analysis, phylogenetic analyses, and molecular dating analyses. Data has been uploaded to DataDryad (see README there).

Supplementary Data 11 Ancestral state reconstruction

Input files used to conduct various ancestral state reconstruction analyses, and the R code used to conduct those analyses. R code can also be found in Supplementary Code 1. Data has been uploaded to DataDryad (see README there).

Supplementary Data 12 Micro CT raw data

Results from the micro-CT scans of eggshell specimens. Data has been uploaded to DataDryad (see README there).

Supplementary Code 1
Code used in ancestral state reconstruction analysis.
