# Supplementary figures and images for: Molecular exploration of fossil eggshell uncovers hidden lineage of giant extinct bird (part 1 of 2)
Source: Nat Commun. 2023 Feb 28;14:914. doi: 10.1038/s41467-023-36405-3 (PMC9974994; doi:10.1038/s41467-023-36405-3)

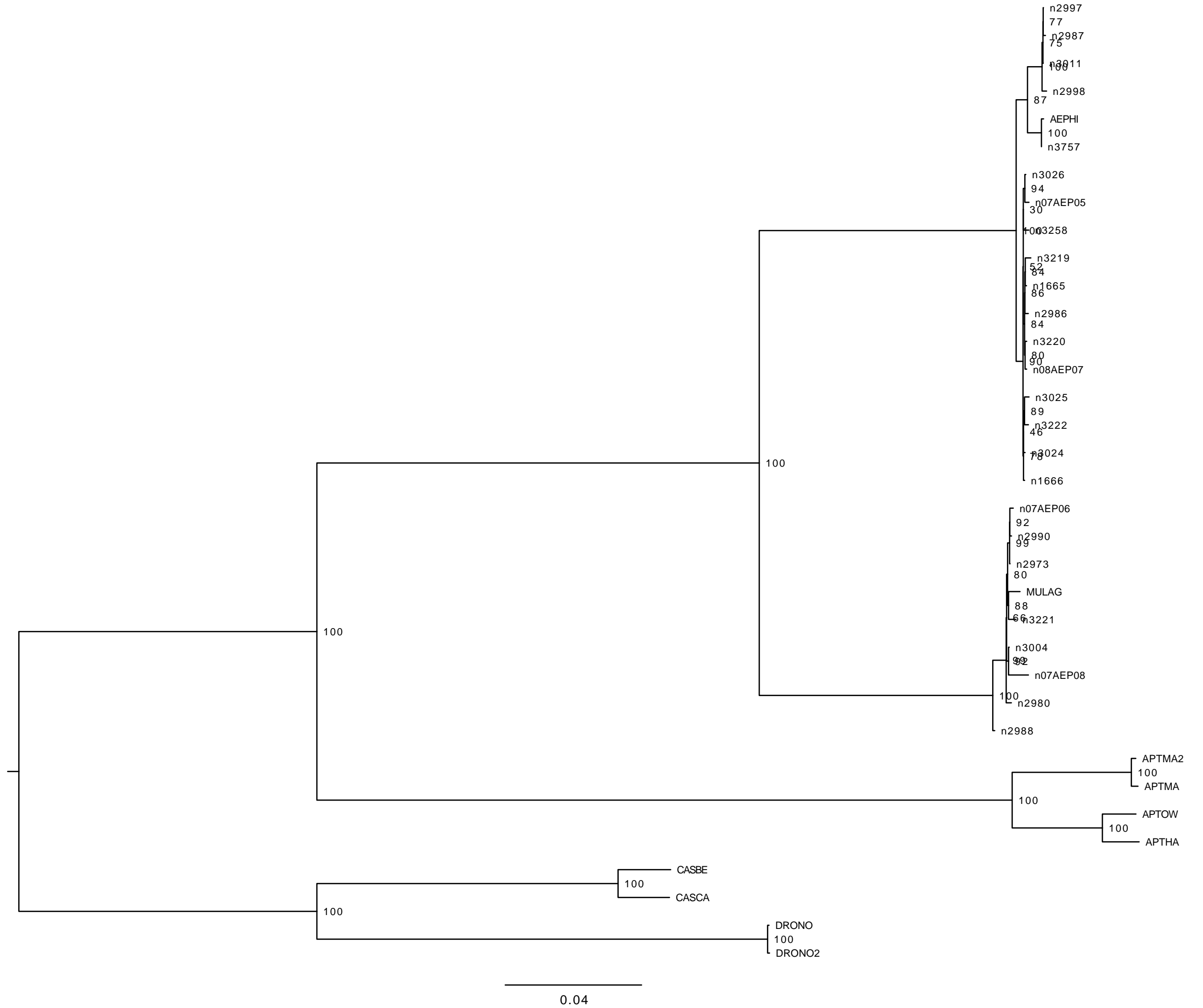

Supplement: Supplementary file 10 — Supplementary Data 10 [file 41467_2023_36405_MOESM10_ESM.zip › Supplementary Data 10 Genetic_data_minus_filtered_reads/5_Phylogeny_generation/RaxML/RAxML_bipartitions.35tax_MAY18_gblocks.tre.pdf]

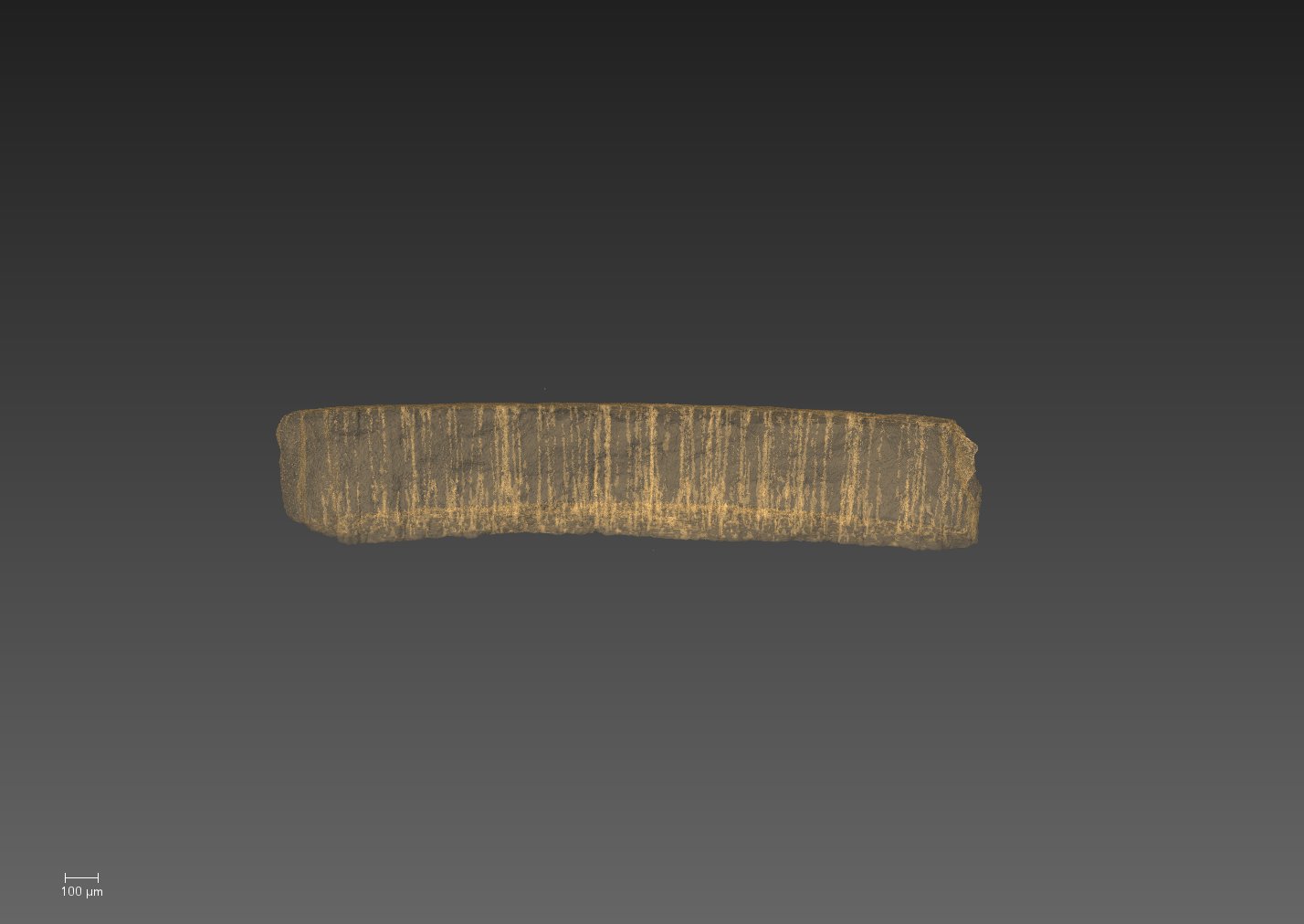

Supplement: Supplementary file 12 — Supplementary Data 12 [file 41467_2023_36405_MOESM12_ESM.zip › Micro_CT_raw_data/Southern_Aepyornis_thin/AD2384/Results/snapshot2.tif]

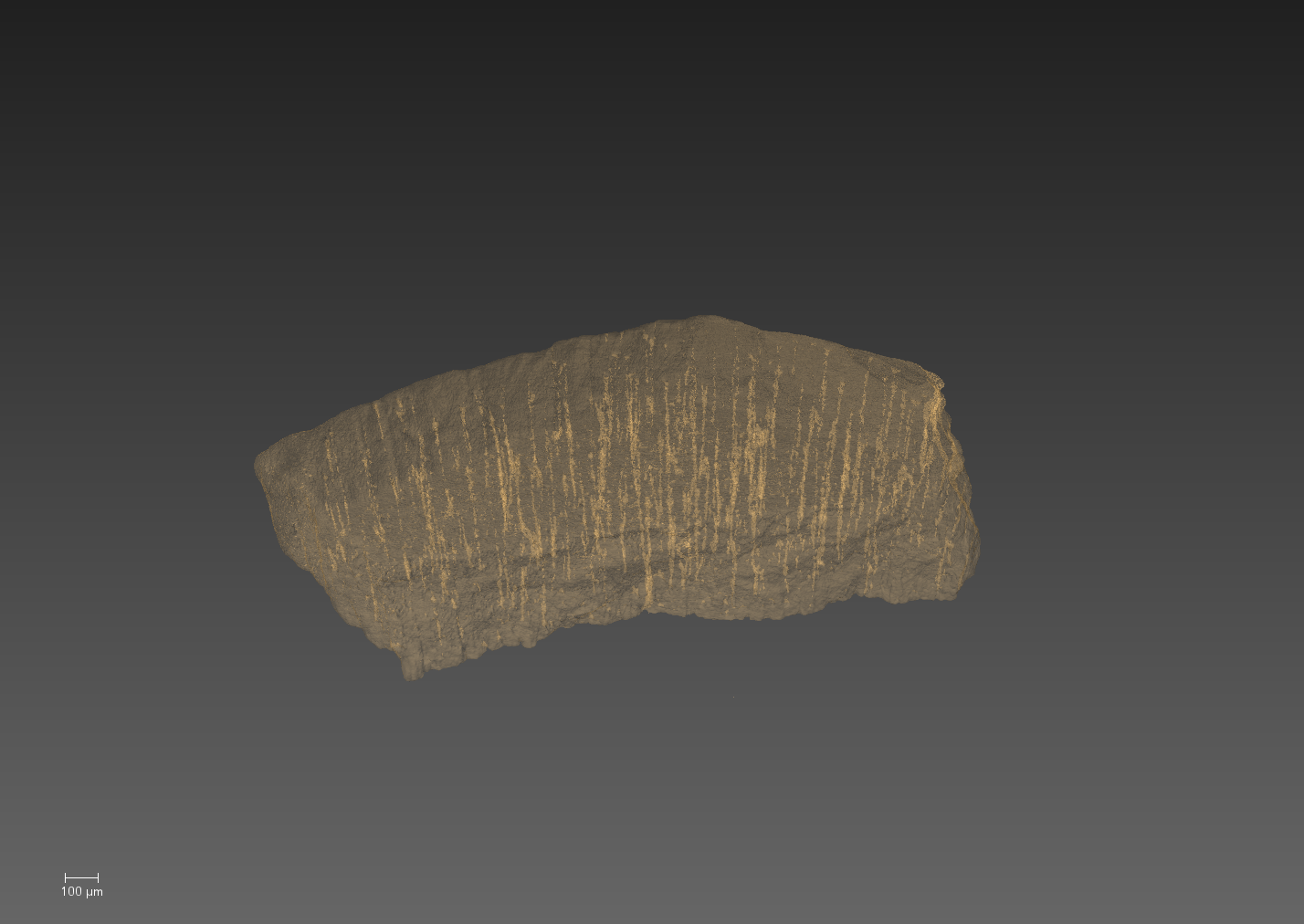

Supplement: Supplementary file 12 — Supplementary Data 12 [file 41467_2023_36405_MOESM12_ESM.zip › Micro_CT_raw_data/Southern_Aepyornis_thin/AD2384/Results/snapshot1.tif]

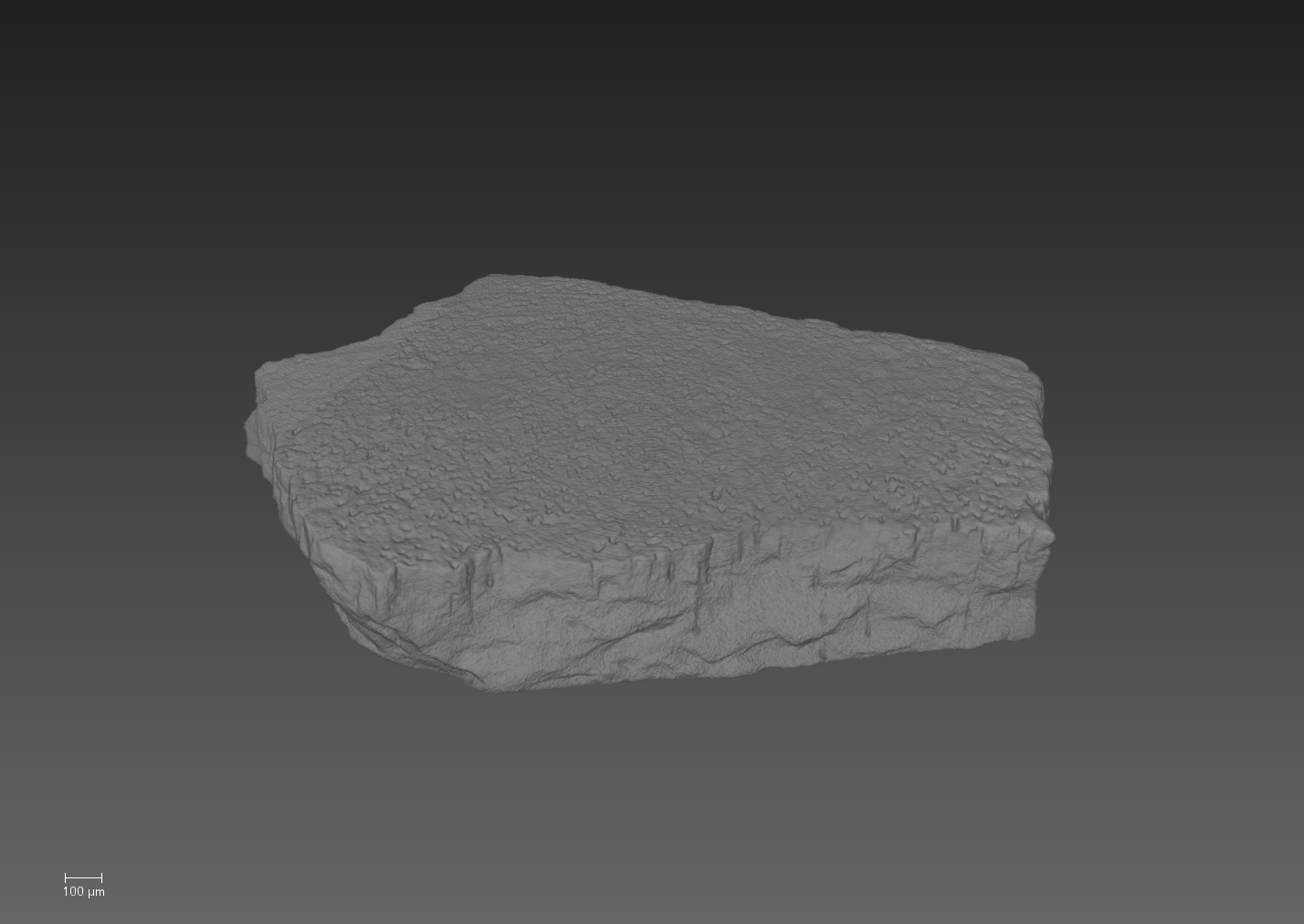

Supplement: Supplementary file 12 — Supplementary Data 12 [file 41467_2023_36405_MOESM12_ESM.zip › Micro_CT_raw_data/Southern_Aepyornis_thin/AD2384/Results/Inner surface.tif]

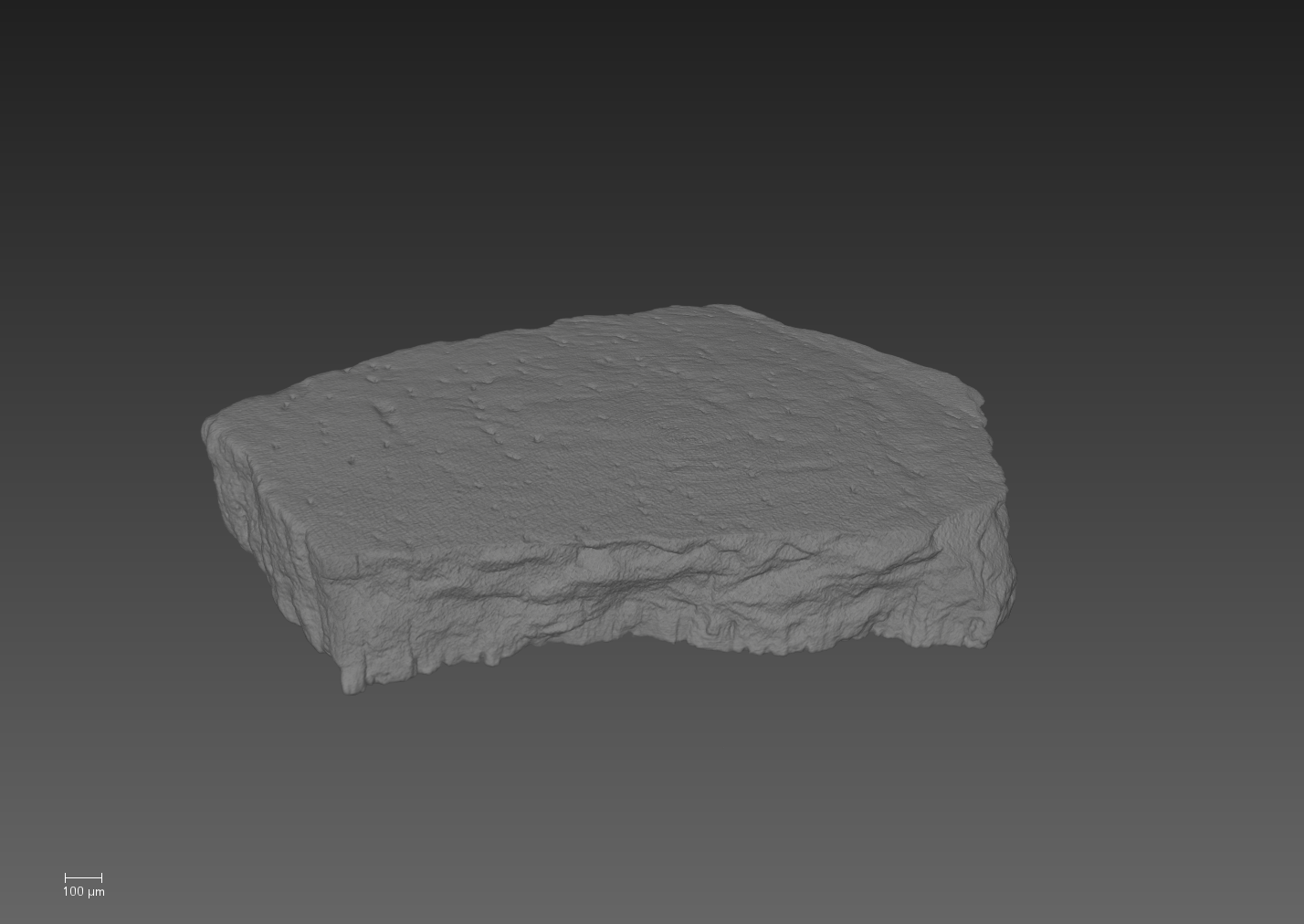

Supplement: Supplementary file 12 — Supplementary Data 12 [file 41467_2023_36405_MOESM12_ESM.zip › Micro_CT_raw_data/Southern_Aepyornis_thin/AD2384/Results/Outer surface.tif]

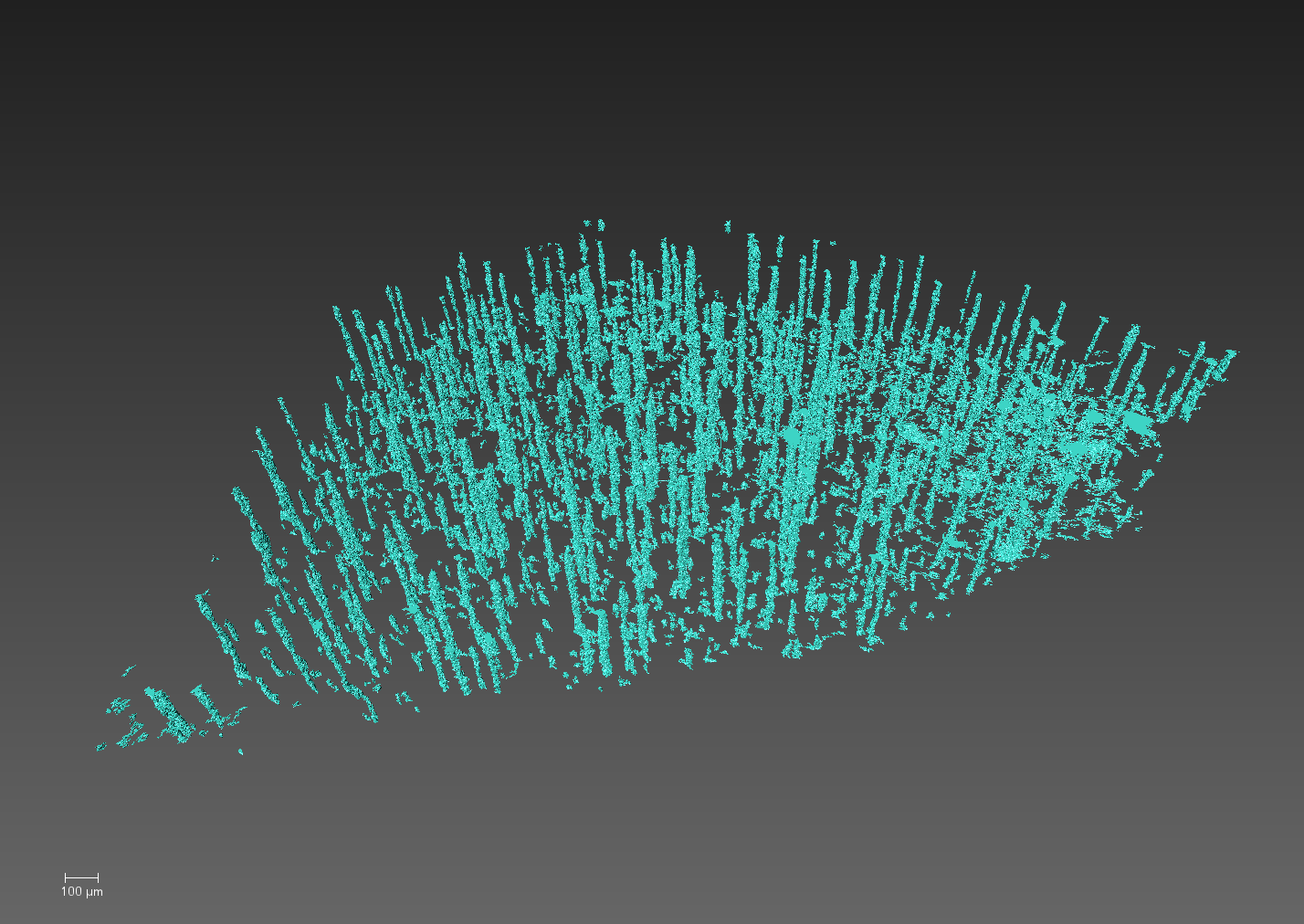

Supplement: Supplementary file 12 — Supplementary Data 12 [file 41467_2023_36405_MOESM12_ESM.zip › Micro_CT_raw_data/Southern_Aepyornis_thin/AD2384/Results/Pore structure.tif]

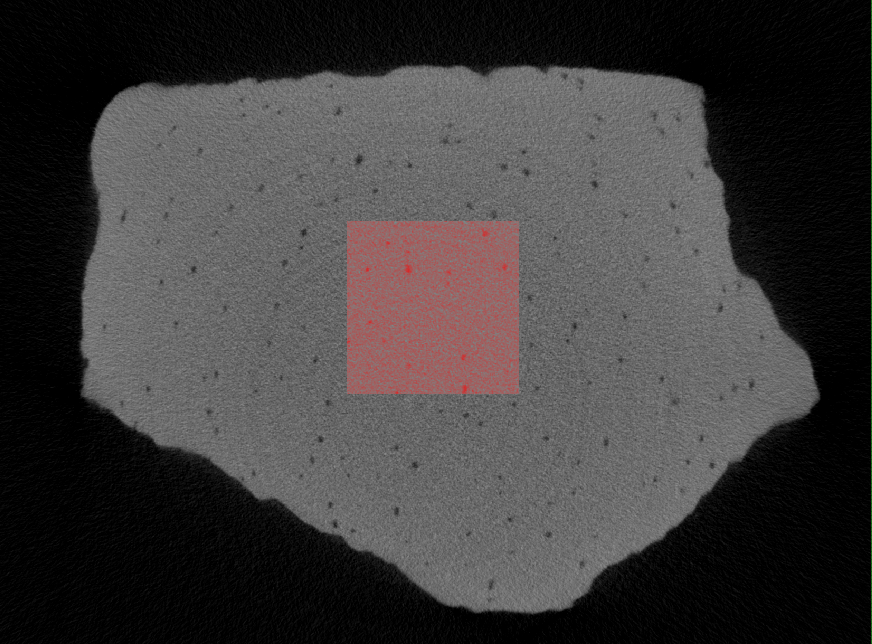

Supplement: Supplementary file 12 — Supplementary Data 12 [file 41467_2023_36405_MOESM12_ESM.zip › Micro_CT_raw_data/Southern_Aepyornis_thin/AD2384/Results/ROI Selection.tif]

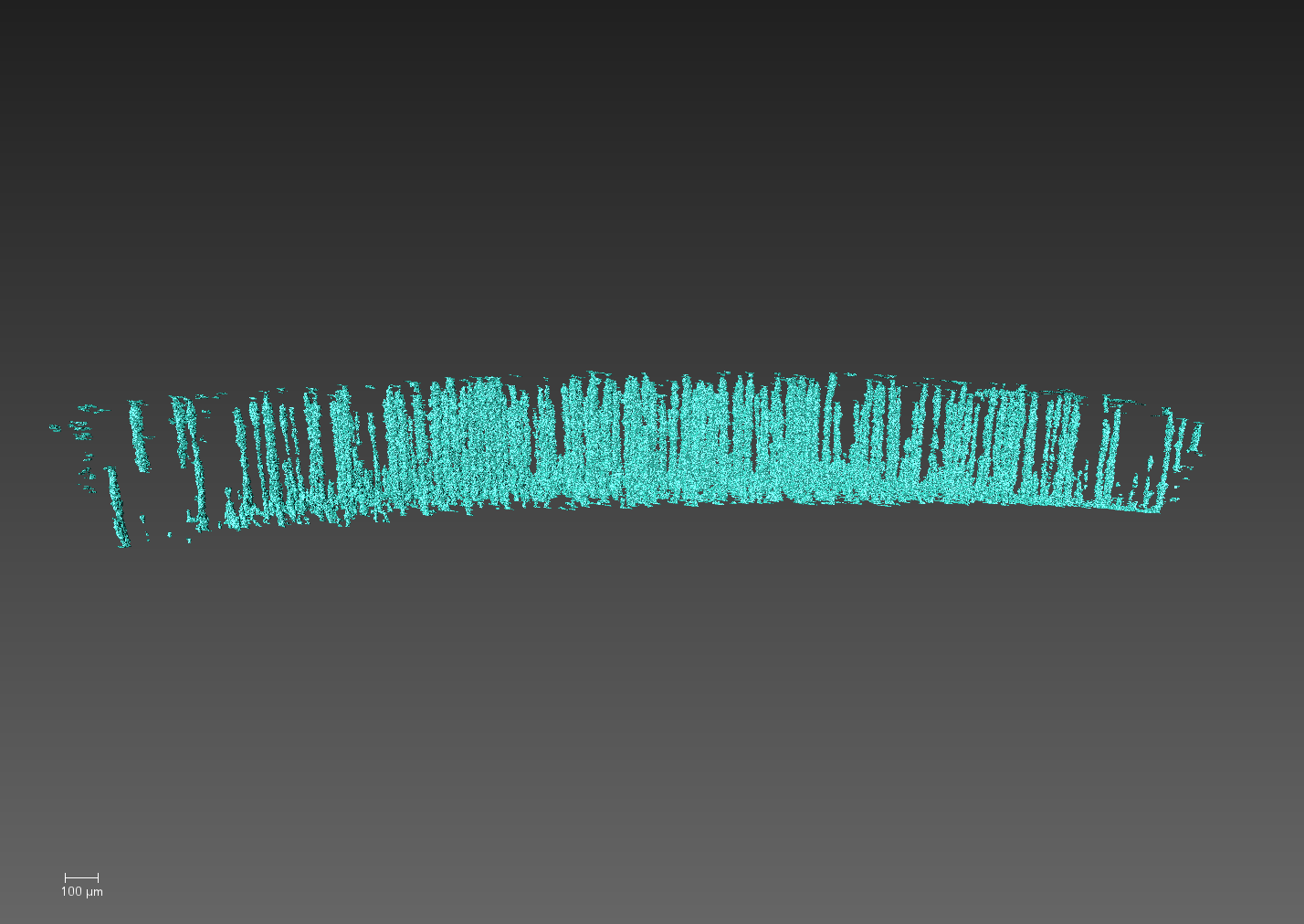

Supplement: Supplementary file 12 — Supplementary Data 12 [file 41467_2023_36405_MOESM12_ESM.zip › Micro_CT_raw_data/Southern_Aepyornis_thin/AD2384/Results/Pore structure2.tif]

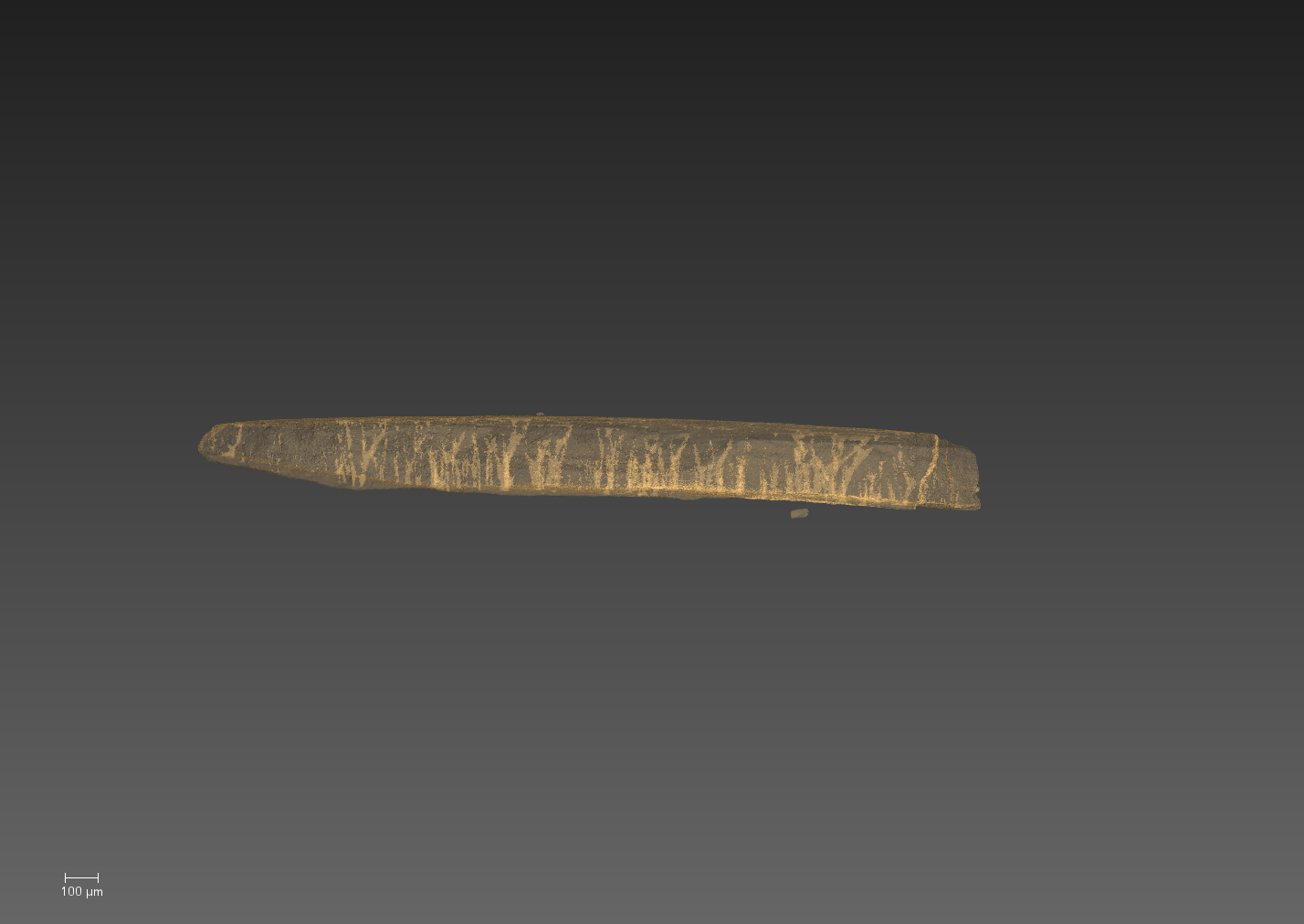

Supplement: Supplementary file 12 — Supplementary Data 12 [file 41467_2023_36405_MOESM12_ESM.zip › Micro_CT_raw_data/Southern_Aepyornis_thin/AD1662/Results/snapshot2.tif]

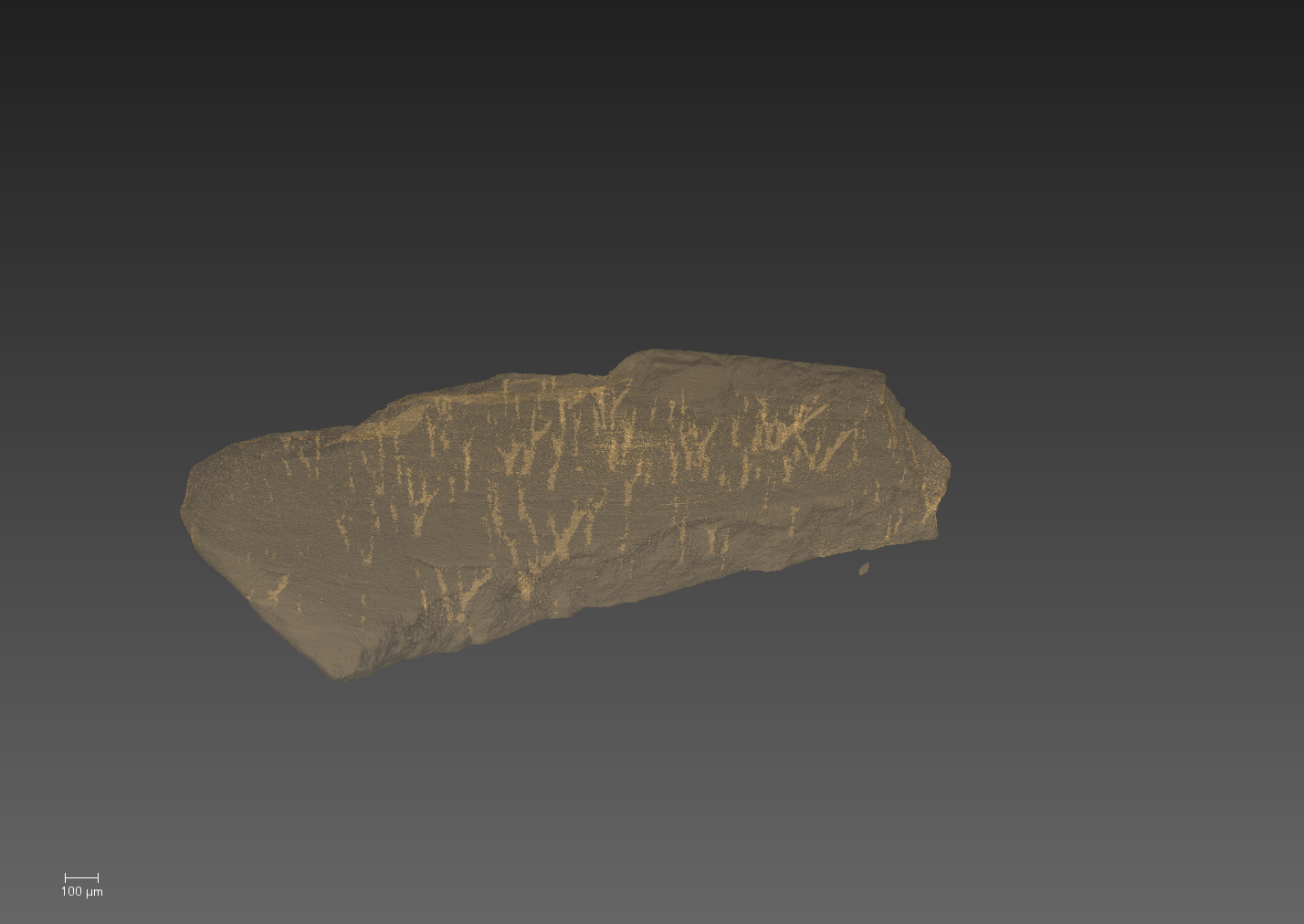

Supplement: Supplementary file 12 — Supplementary Data 12 [file 41467_2023_36405_MOESM12_ESM.zip › Micro_CT_raw_data/Southern_Aepyornis_thin/AD1662/Results/snapshot1.tif]

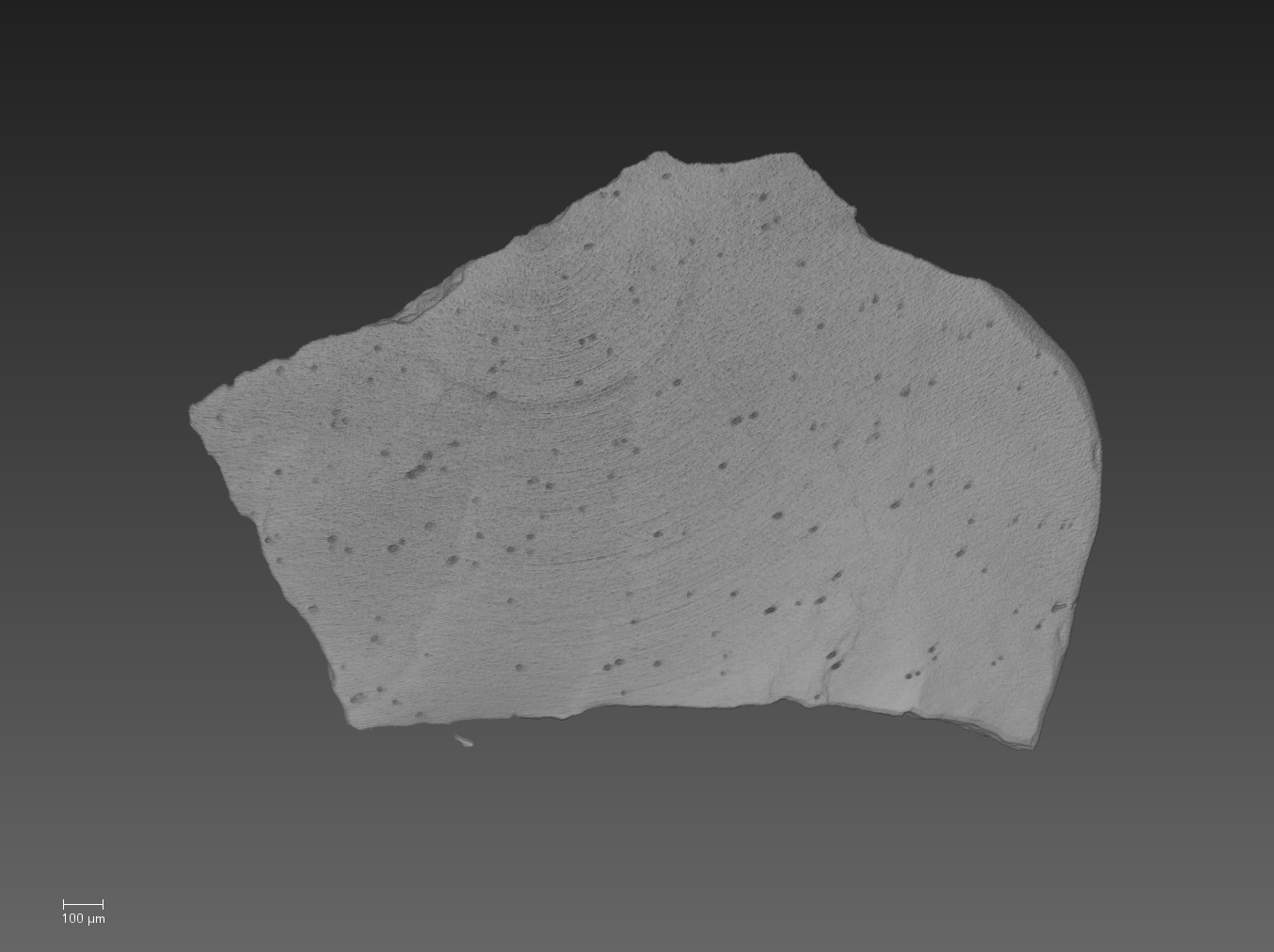

Supplement: Supplementary file 12 — Supplementary Data 12 [file 41467_2023_36405_MOESM12_ESM.zip › Micro_CT_raw_data/Southern_Aepyornis_thin/AD1662/Results/Inner surface.tif]

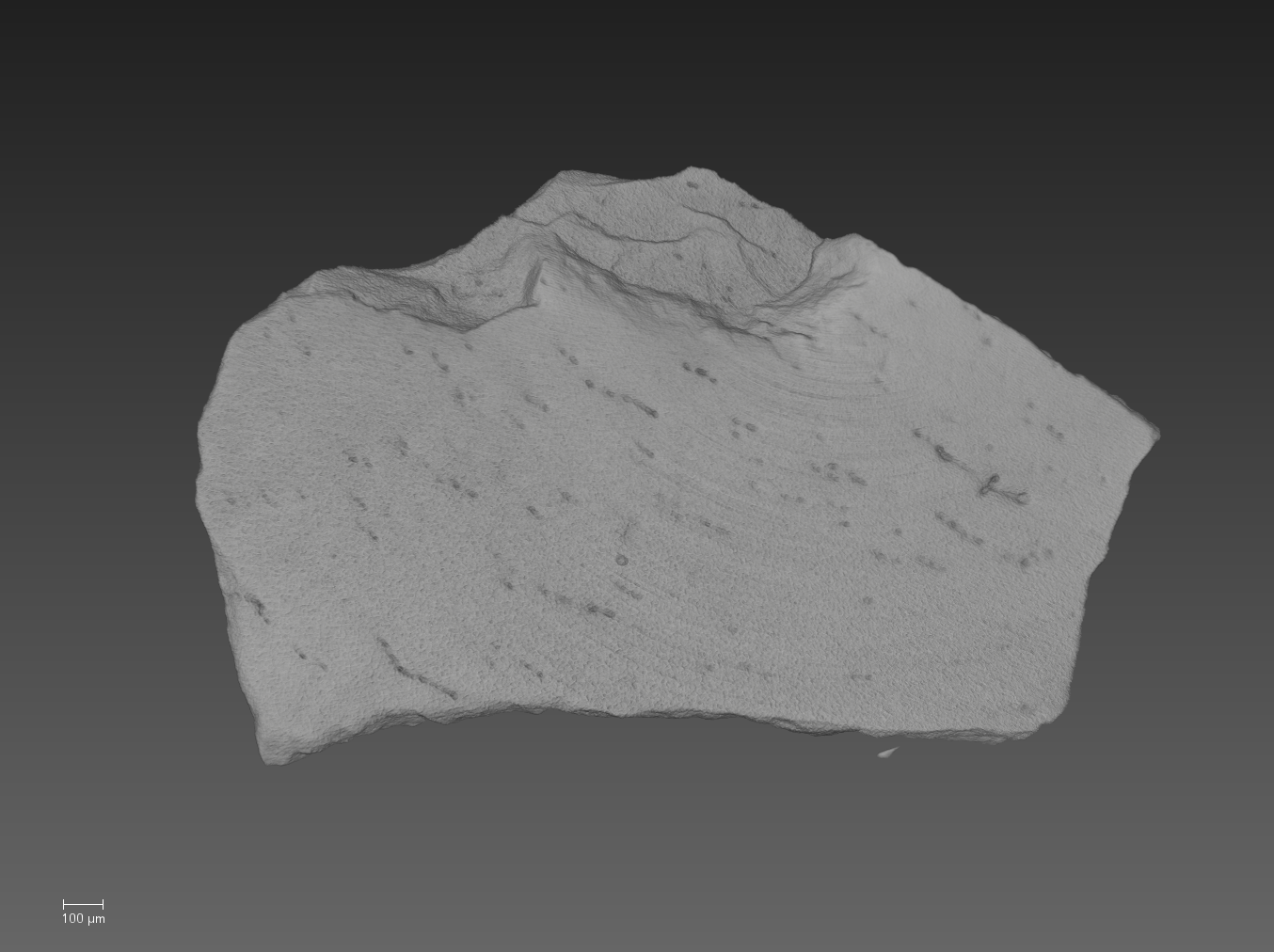

Supplement: Supplementary file 12 — Supplementary Data 12 [file 41467_2023_36405_MOESM12_ESM.zip › Micro_CT_raw_data/Southern_Aepyornis_thin/AD1662/Results/Outer surface.tif]

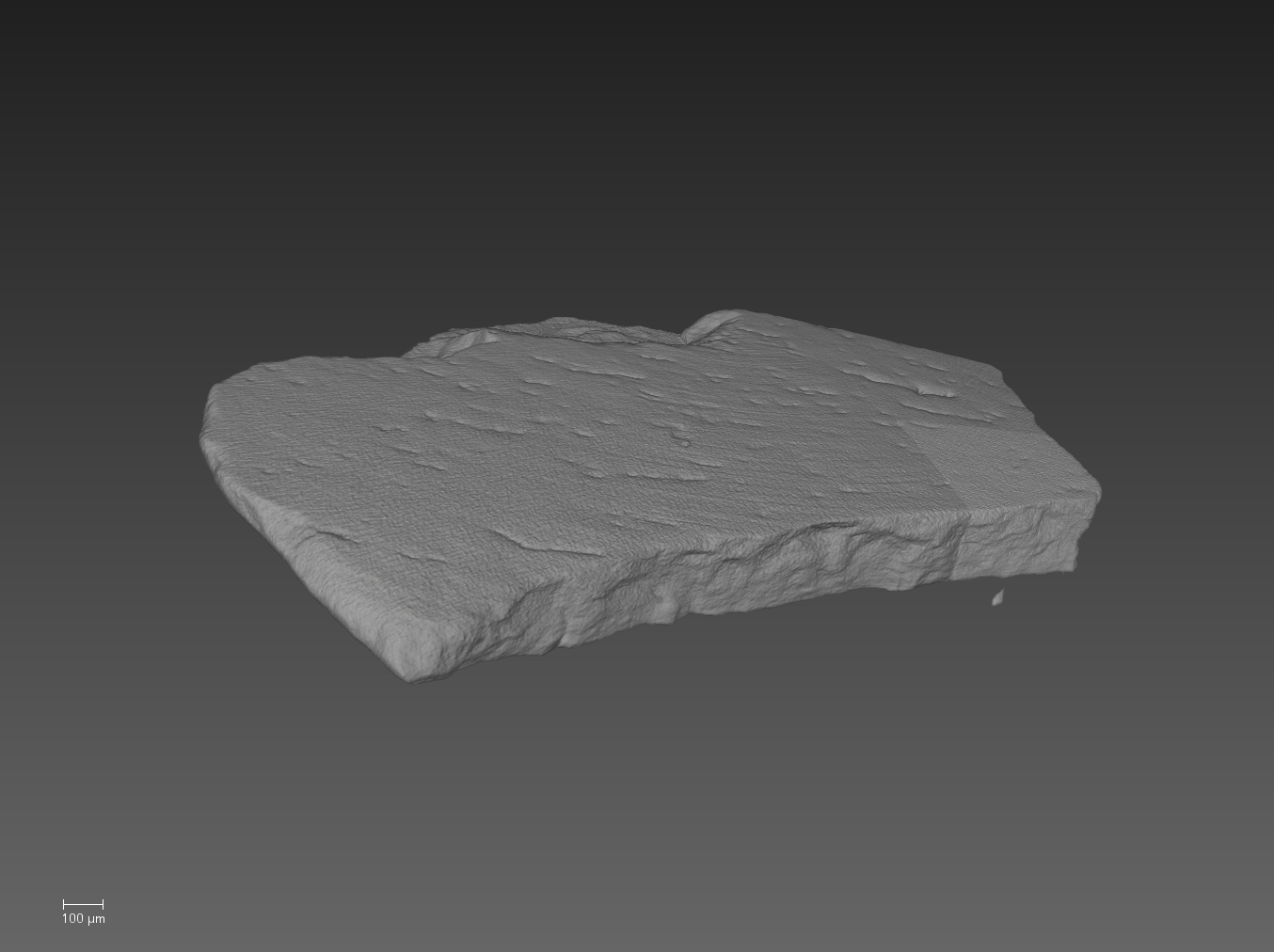

Supplement: Supplementary file 12 — Supplementary Data 12 [file 41467_2023_36405_MOESM12_ESM.zip › Micro_CT_raw_data/Southern_Aepyornis_thin/AD1662/Results/Outer surface2.tif]

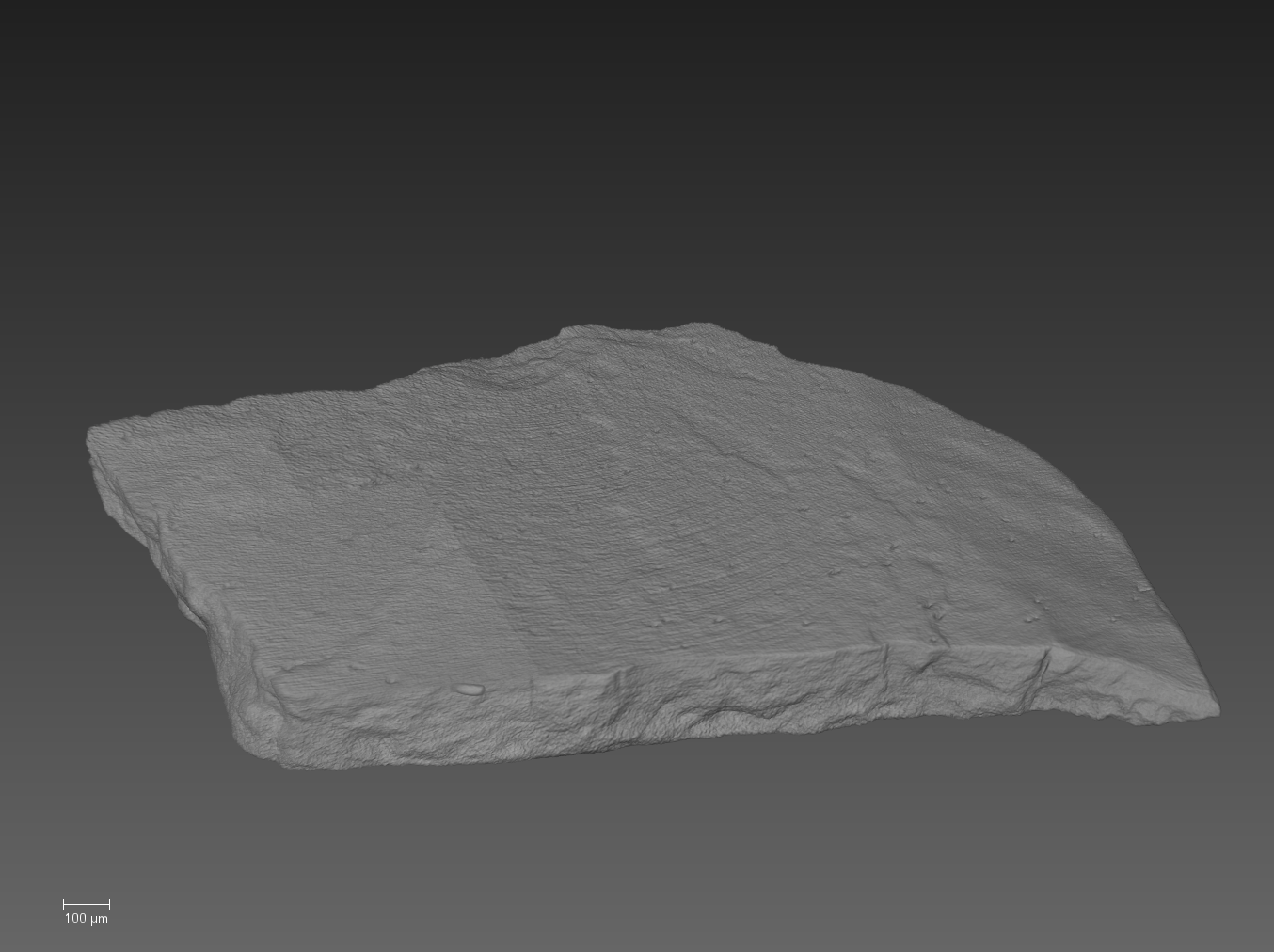

Supplement: Supplementary file 12 — Supplementary Data 12 [file 41467_2023_36405_MOESM12_ESM.zip › Micro_CT_raw_data/Southern_Aepyornis_thin/AD1662/Results/Inner surface2.tif]

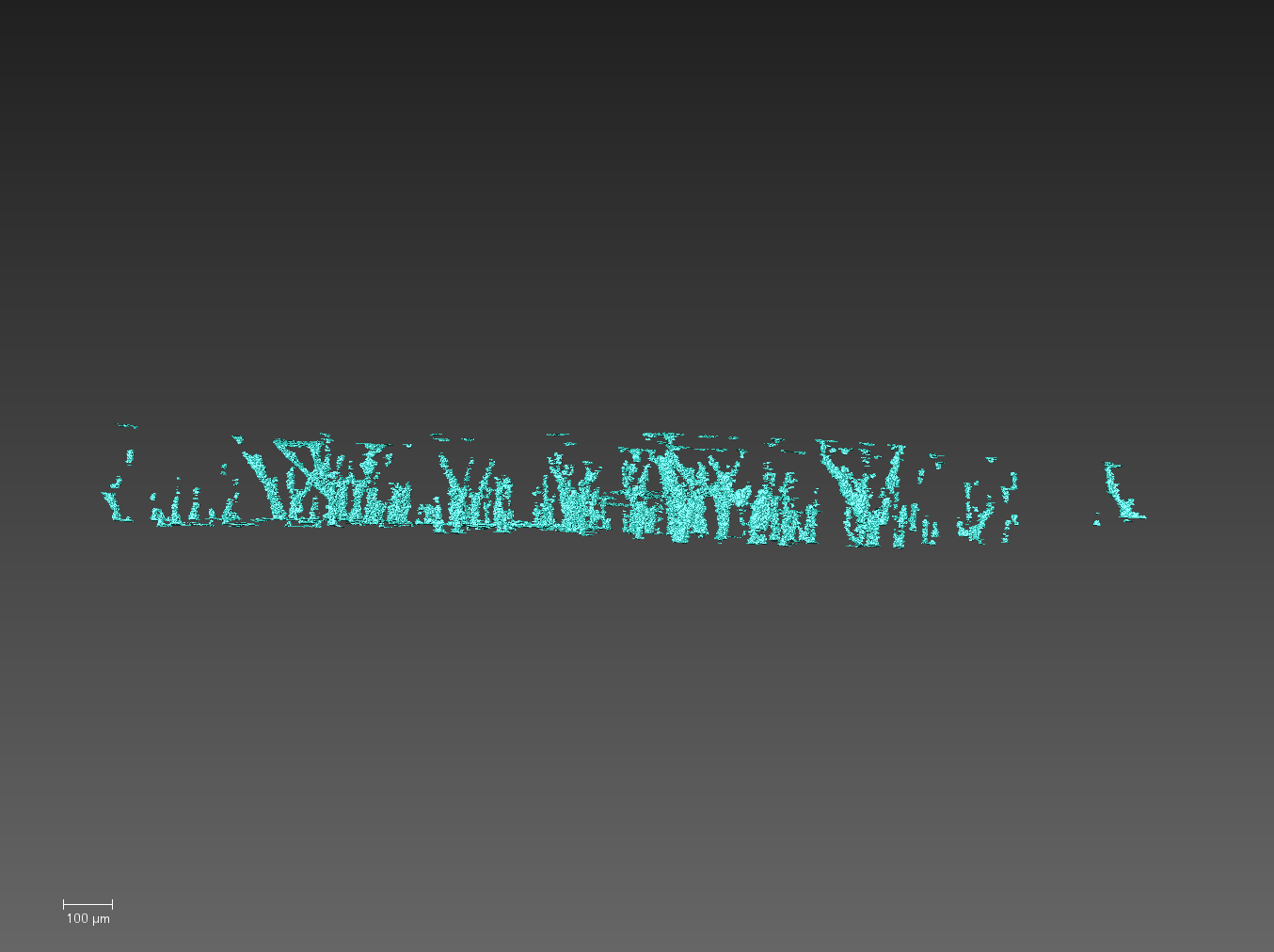

Supplement: Supplementary file 12 — Supplementary Data 12 [file 41467_2023_36405_MOESM12_ESM.zip › Micro_CT_raw_data/Southern_Aepyornis_thin/AD1662/Results/Pore structure.tif]

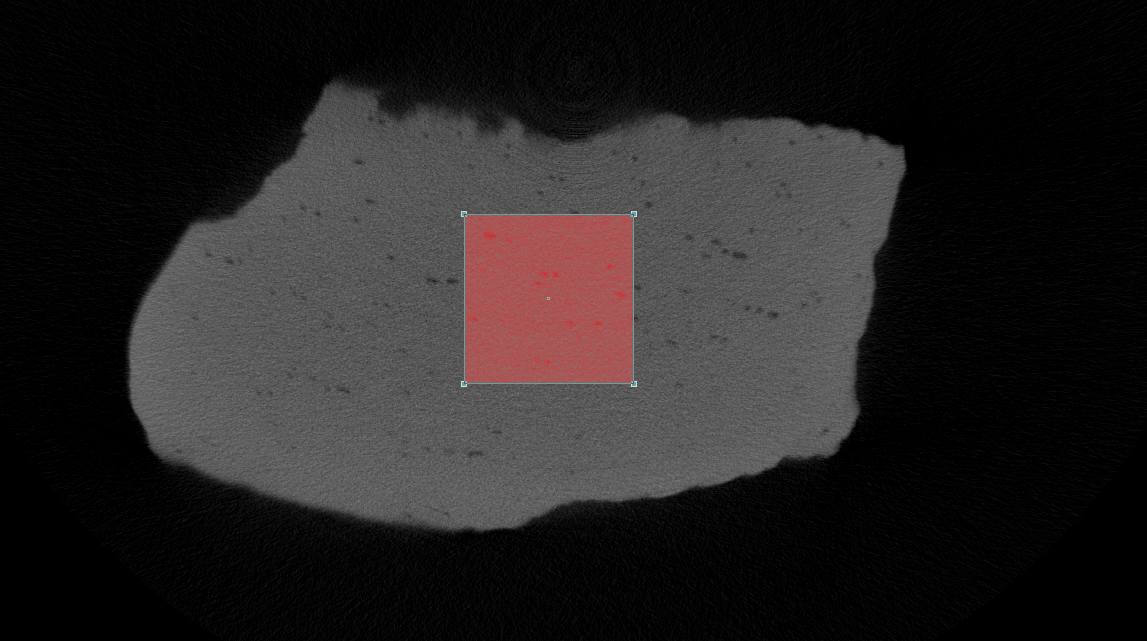

Supplement: Supplementary file 12 — Supplementary Data 12 [file 41467_2023_36405_MOESM12_ESM.zip › Micro_CT_raw_data/Southern_Aepyornis_thin/AD1662/Results/ROI Selection.tif]

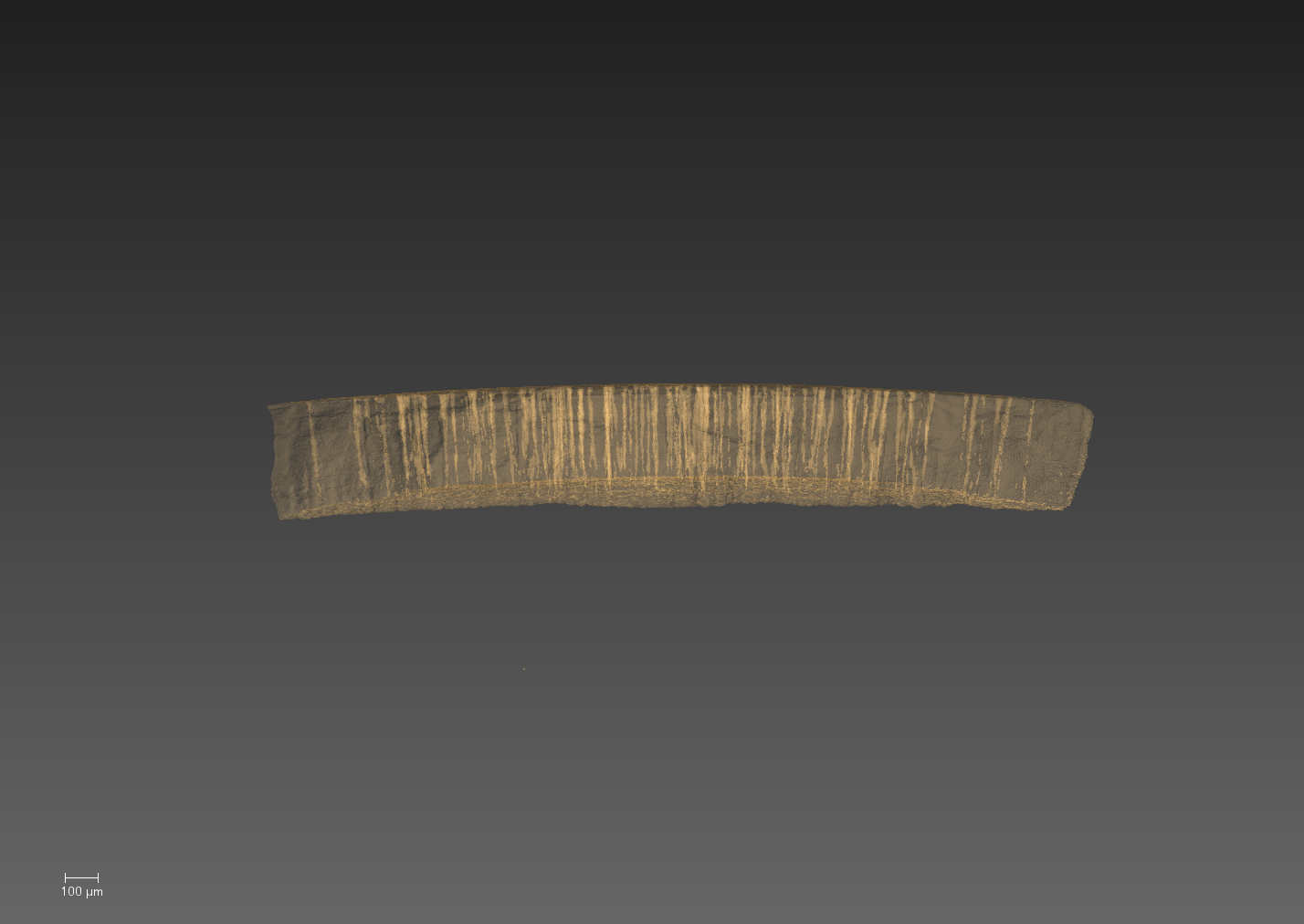

Supplement: Supplementary file 12 — Supplementary Data 12 [file 41467_2023_36405_MOESM12_ESM.zip › Micro_CT_raw_data/Southern_Aepyornis_thin/AD2134/Results/snapshot2.tif]

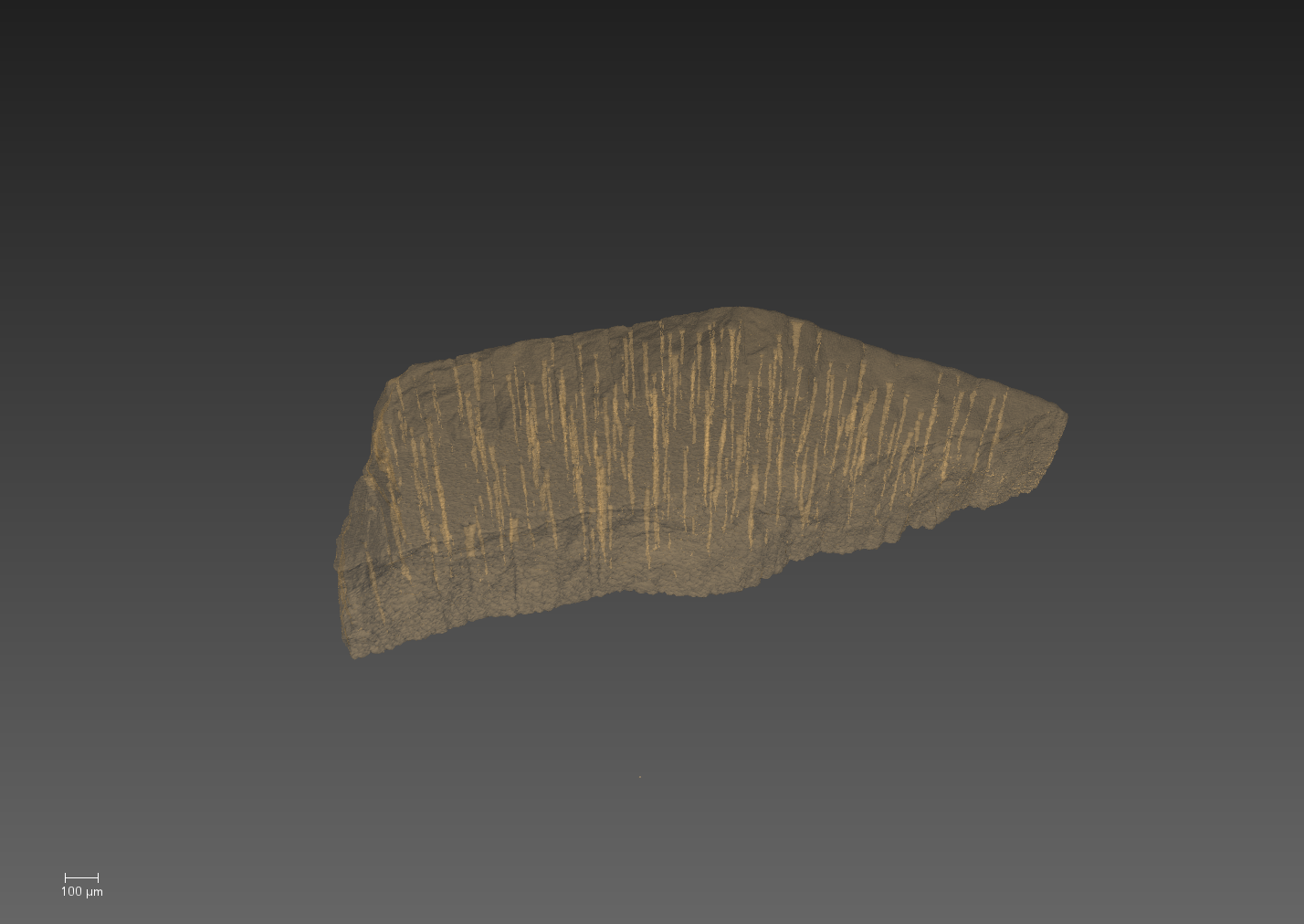

Supplement: Supplementary file 12 — Supplementary Data 12 [file 41467_2023_36405_MOESM12_ESM.zip › Micro_CT_raw_data/Southern_Aepyornis_thin/AD2134/Results/snapshot1.tif]

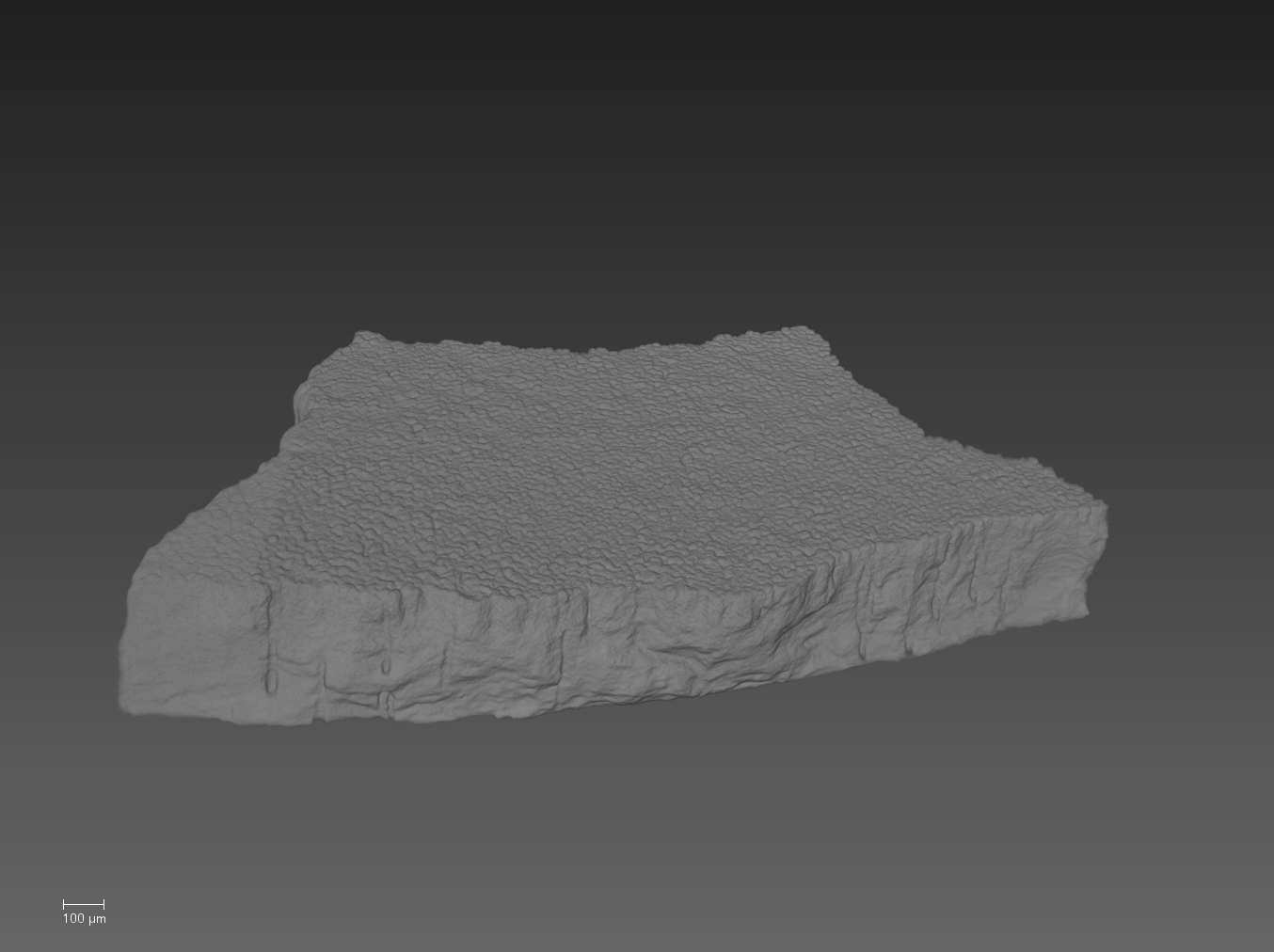

Supplement: Supplementary file 12 — Supplementary Data 12 [file 41467_2023_36405_MOESM12_ESM.zip › Micro_CT_raw_data/Southern_Aepyornis_thin/AD2134/Results/Inner surface.tif]

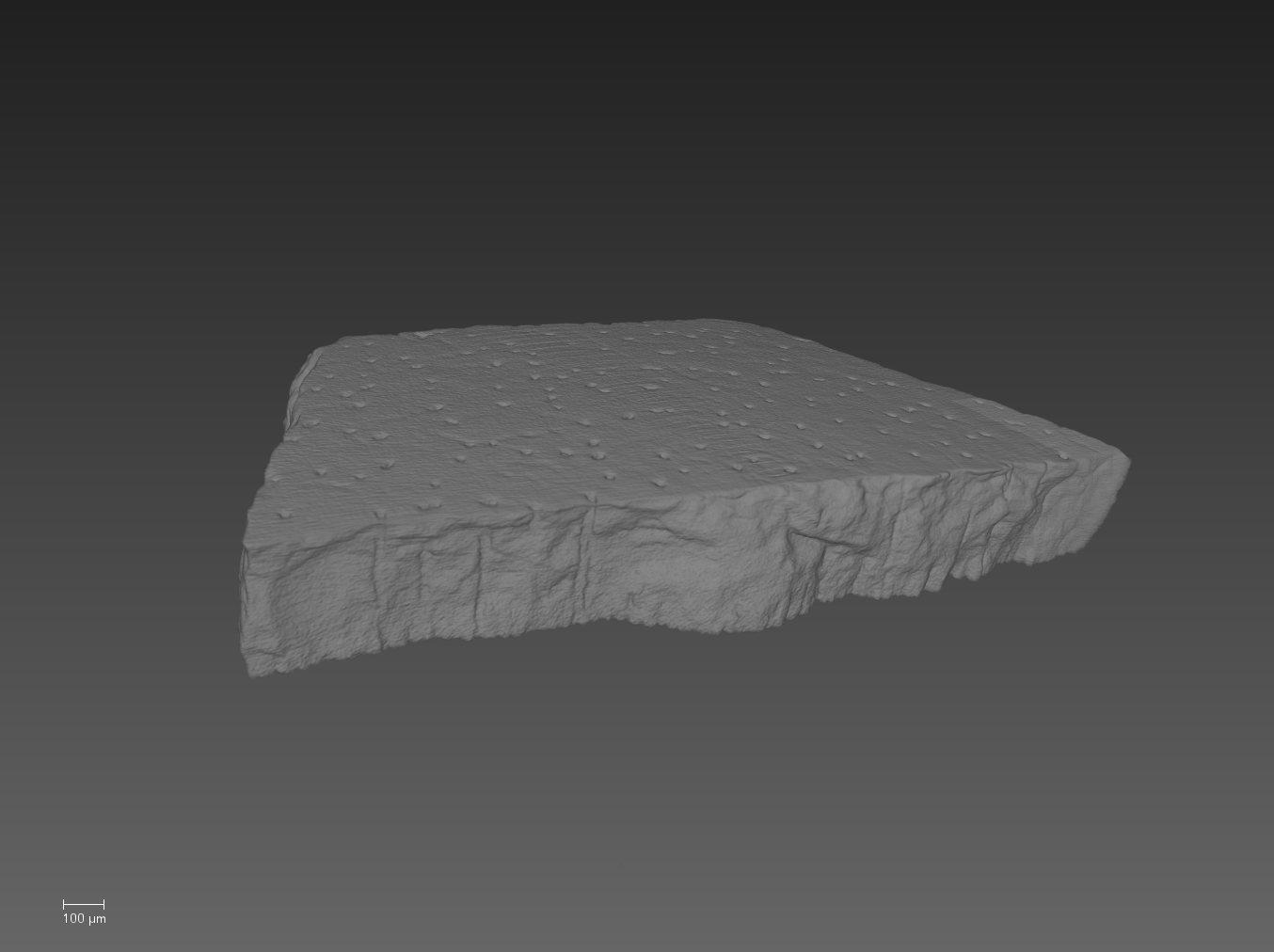

Supplement: Supplementary file 12 — Supplementary Data 12 [file 41467_2023_36405_MOESM12_ESM.zip › Micro_CT_raw_data/Southern_Aepyornis_thin/AD2134/Results/Outer surface.tif]

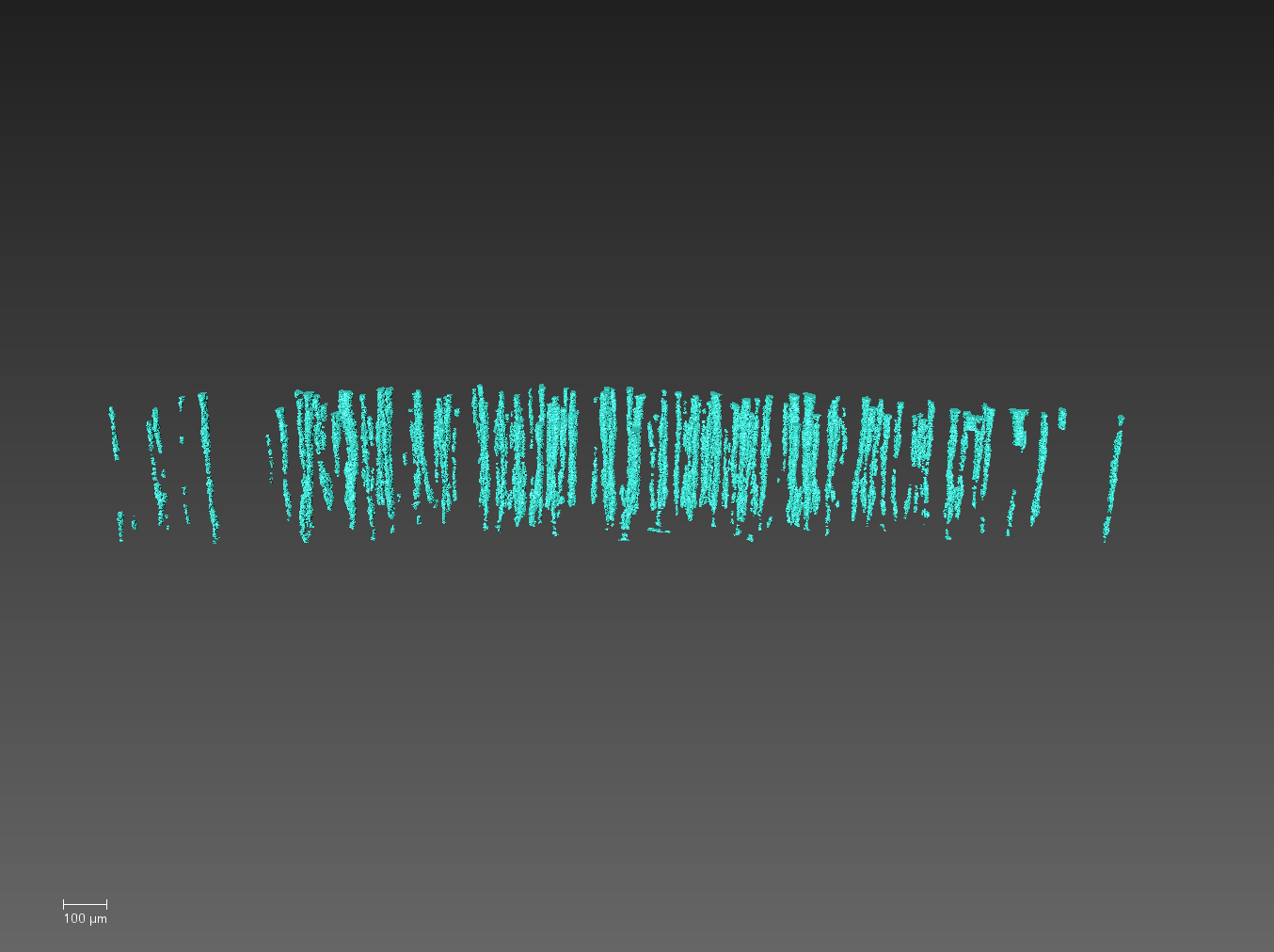

Supplement: Supplementary file 12 — Supplementary Data 12 [file 41467_2023_36405_MOESM12_ESM.zip › Micro_CT_raw_data/Southern_Aepyornis_thin/AD2134/Results/Pore structure.tif]

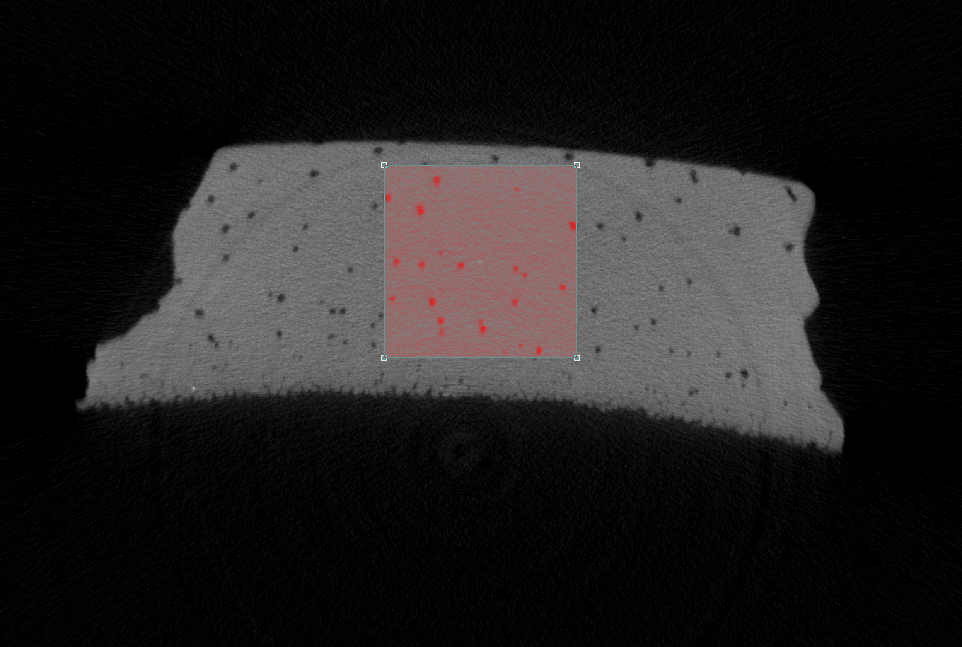

Supplement: Supplementary file 12 — Supplementary Data 12 [file 41467_2023_36405_MOESM12_ESM.zip › Micro_CT_raw_data/Southern_Aepyornis_thin/AD2134/Results/ROI Selection.tif]

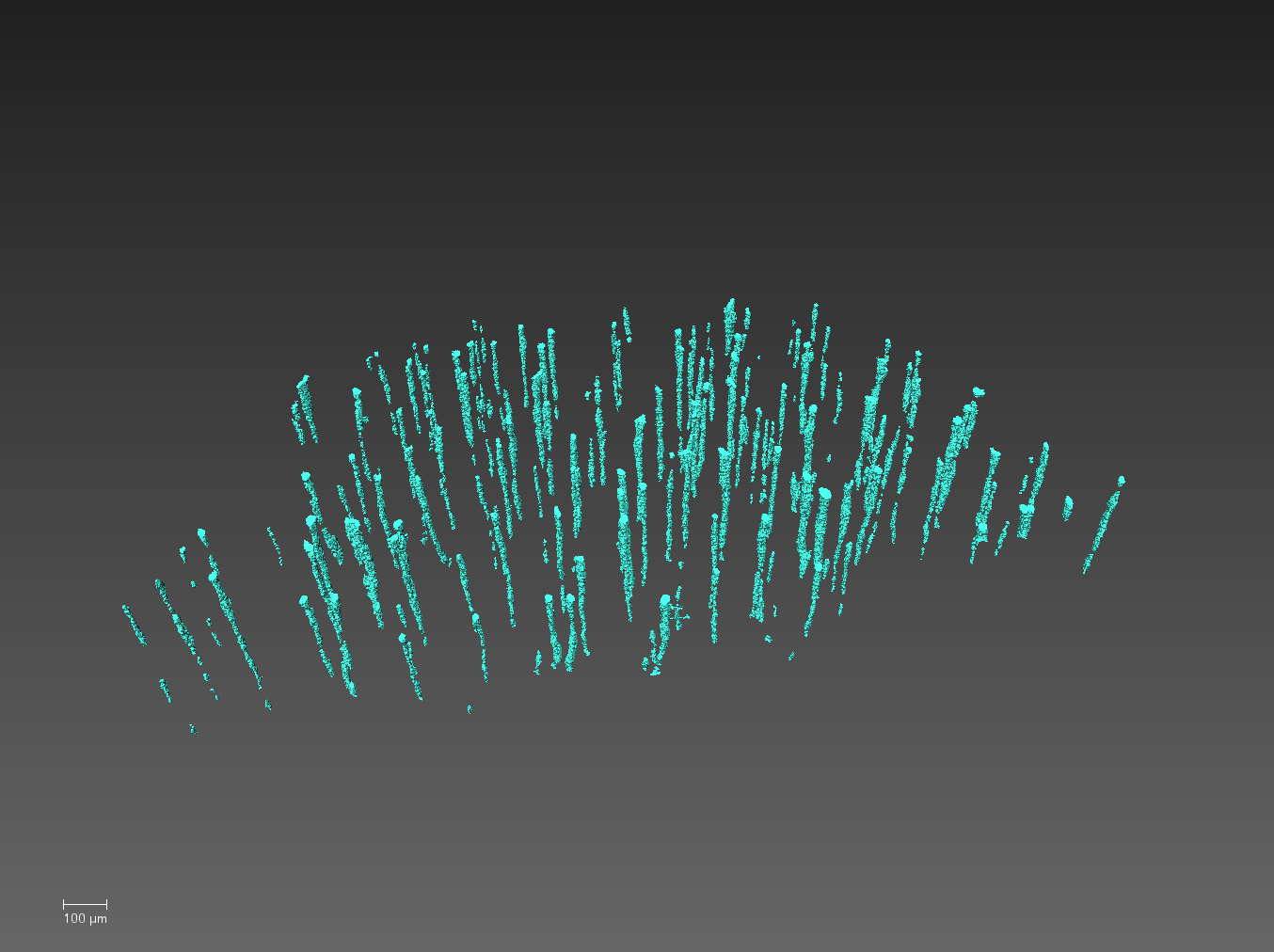

Supplement: Supplementary file 12 — Supplementary Data 12 [file 41467_2023_36405_MOESM12_ESM.zip › Micro_CT_raw_data/Southern_Aepyornis_thin/AD2134/Results/Pore structure2.tif]

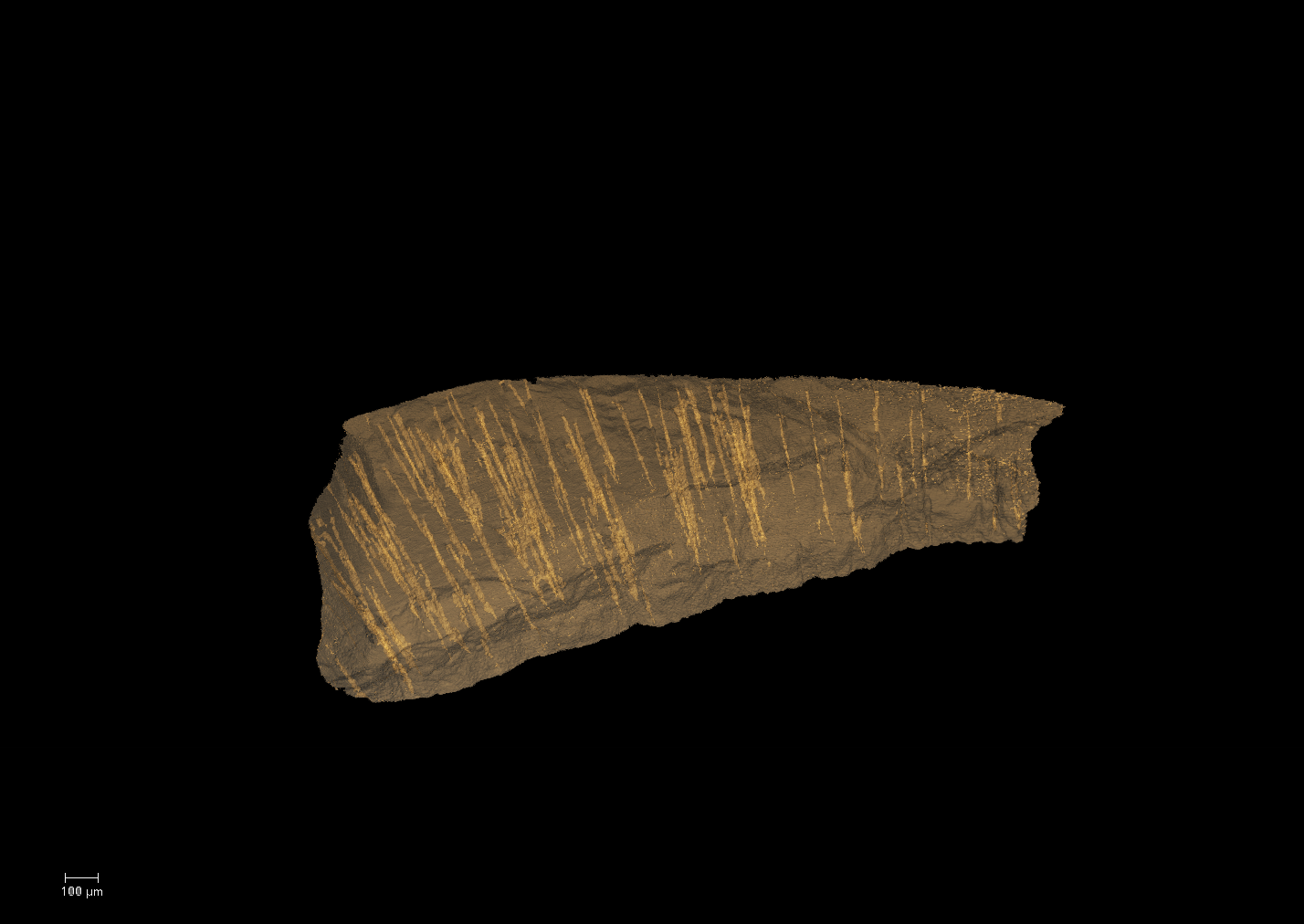

Supplement: Supplementary file 12 — Supplementary Data 12 [file 41467_2023_36405_MOESM12_ESM.zip › Micro_CT_raw_data/Southern_Aepyornis_thin/AD2135/Results/snapshot3.tif]

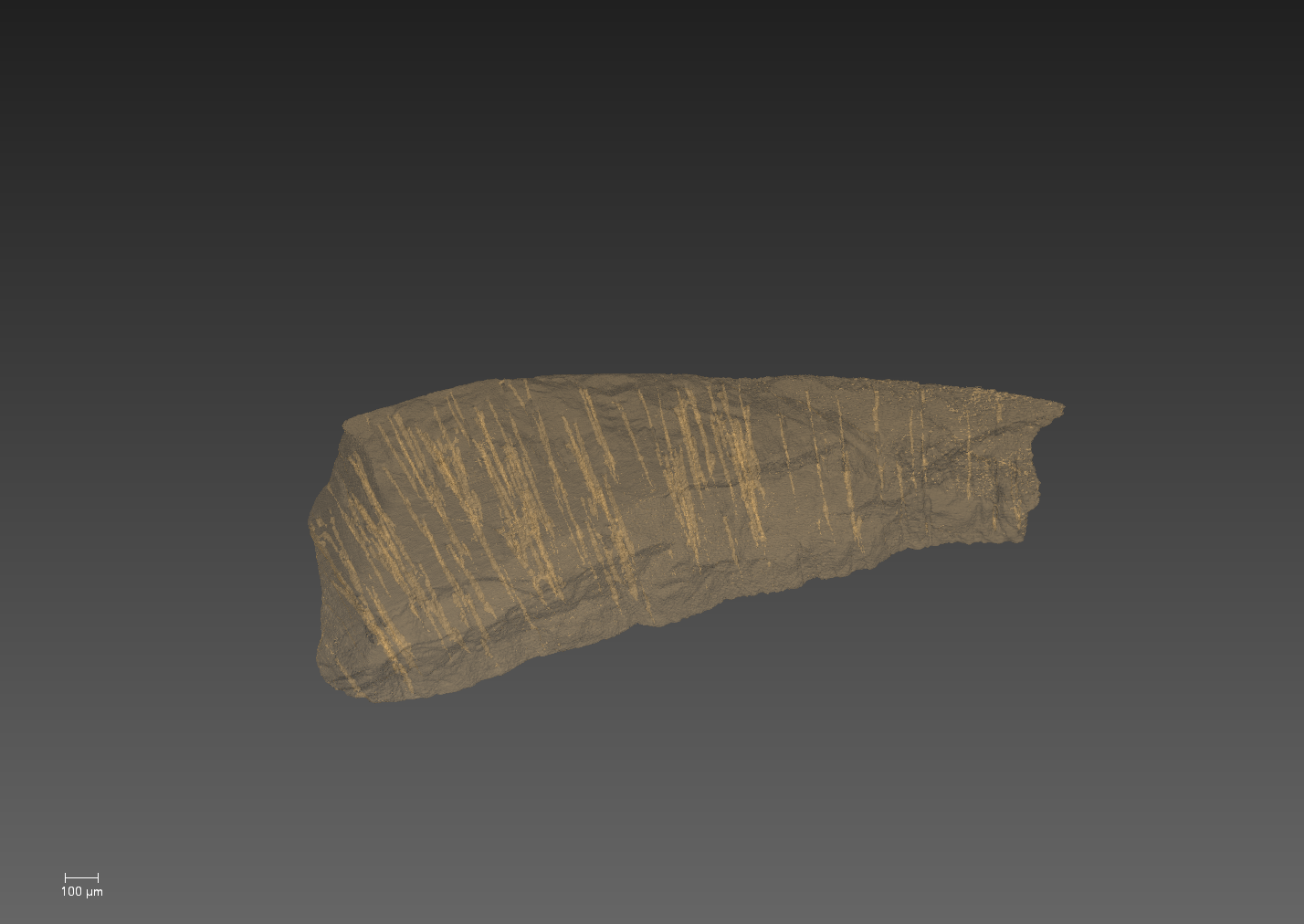

Supplement: Supplementary file 12 — Supplementary Data 12 [file 41467_2023_36405_MOESM12_ESM.zip › Micro_CT_raw_data/Southern_Aepyornis_thin/AD2135/Results/snapshot2.tif]

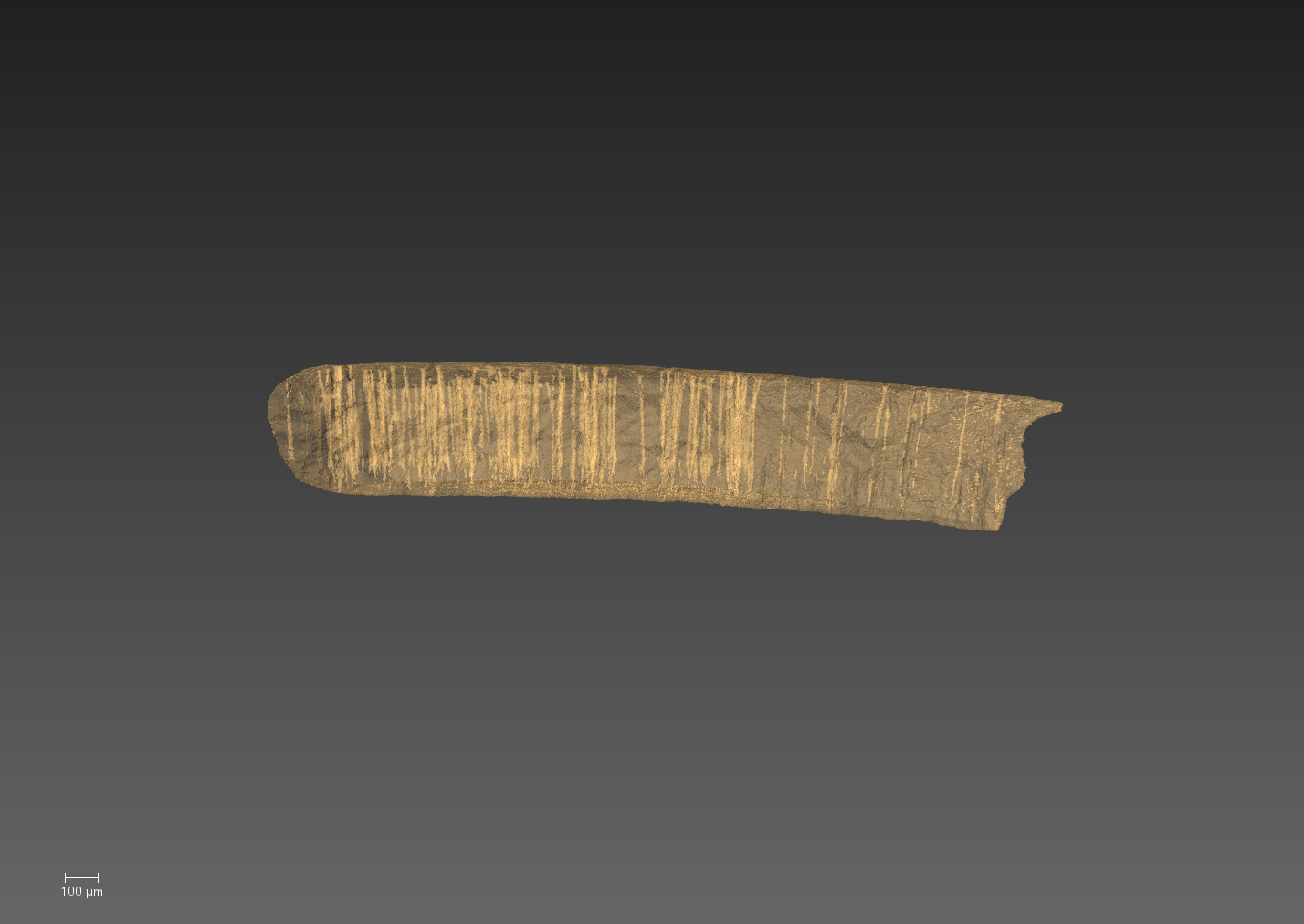

Supplement: Supplementary file 12 — Supplementary Data 12 [file 41467_2023_36405_MOESM12_ESM.zip › Micro_CT_raw_data/Southern_Aepyornis_thin/AD2135/Results/snapshot.tif]

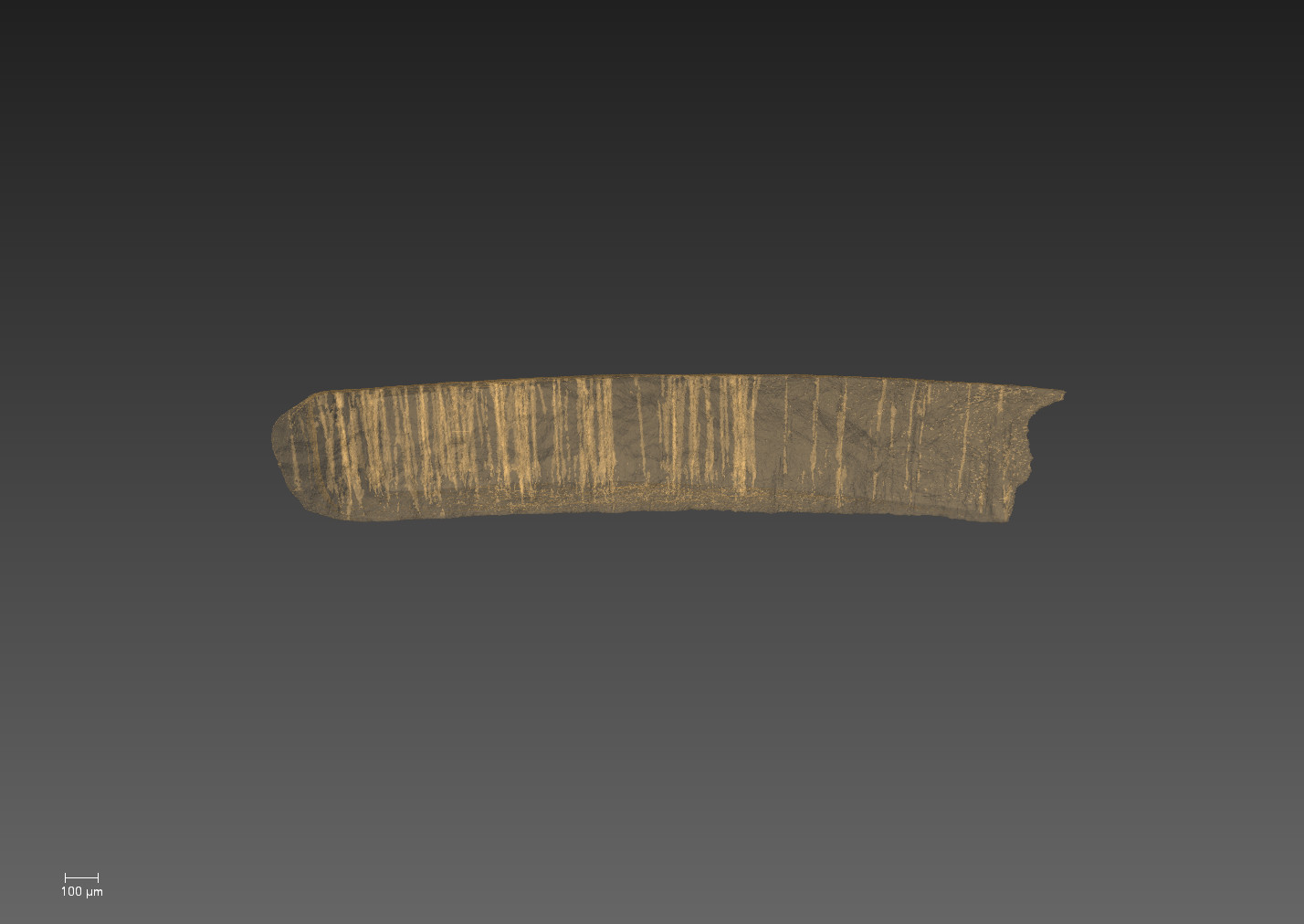

Supplement: Supplementary file 12 — Supplementary Data 12 [file 41467_2023_36405_MOESM12_ESM.zip › Micro_CT_raw_data/Southern_Aepyornis_thin/AD2135/Results/snapshot4.tif]

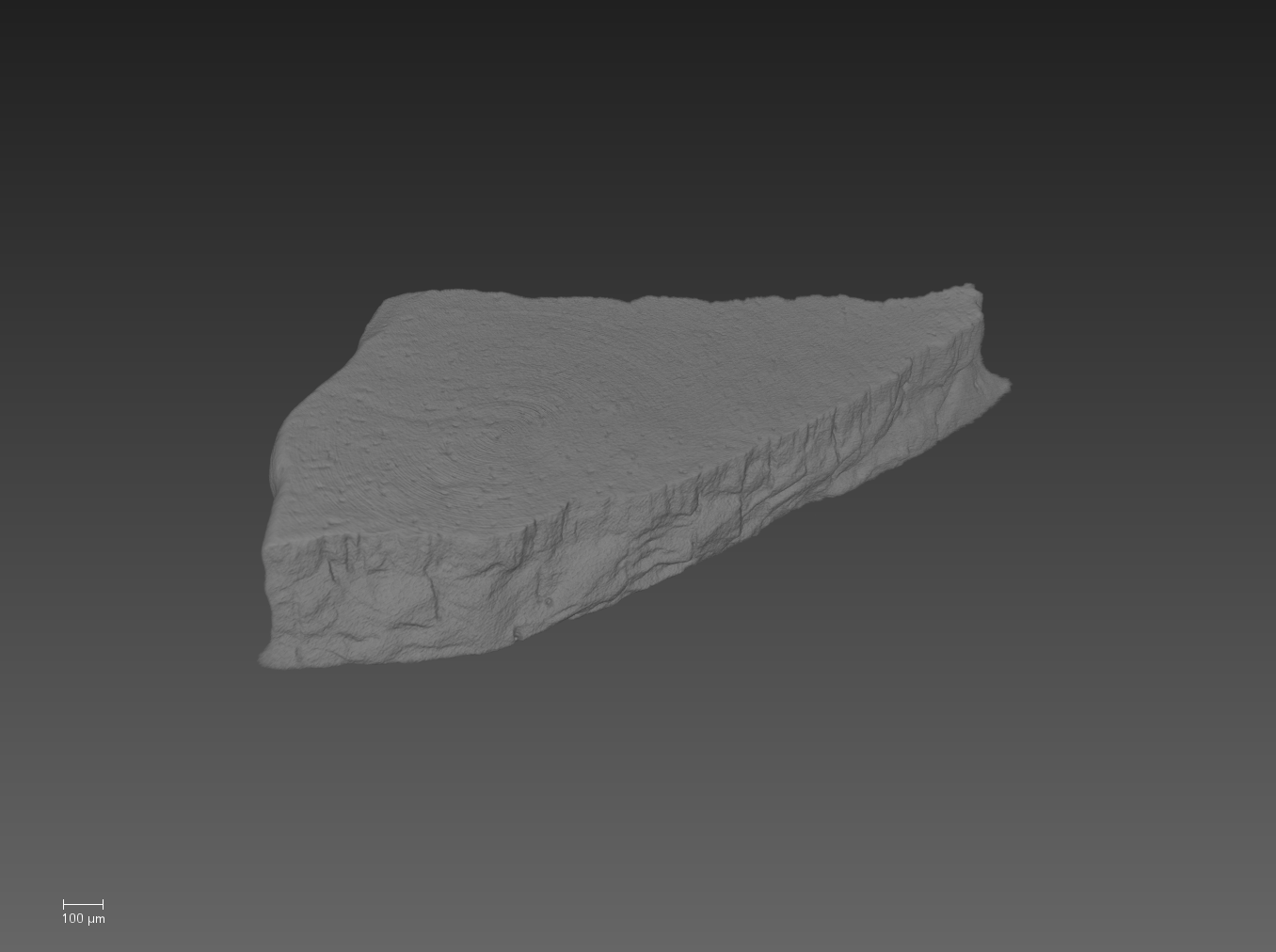

Supplement: Supplementary file 12 — Supplementary Data 12 [file 41467_2023_36405_MOESM12_ESM.zip › Micro_CT_raw_data/Southern_Aepyornis_thin/AD2135/Results/Inner surface.tif]

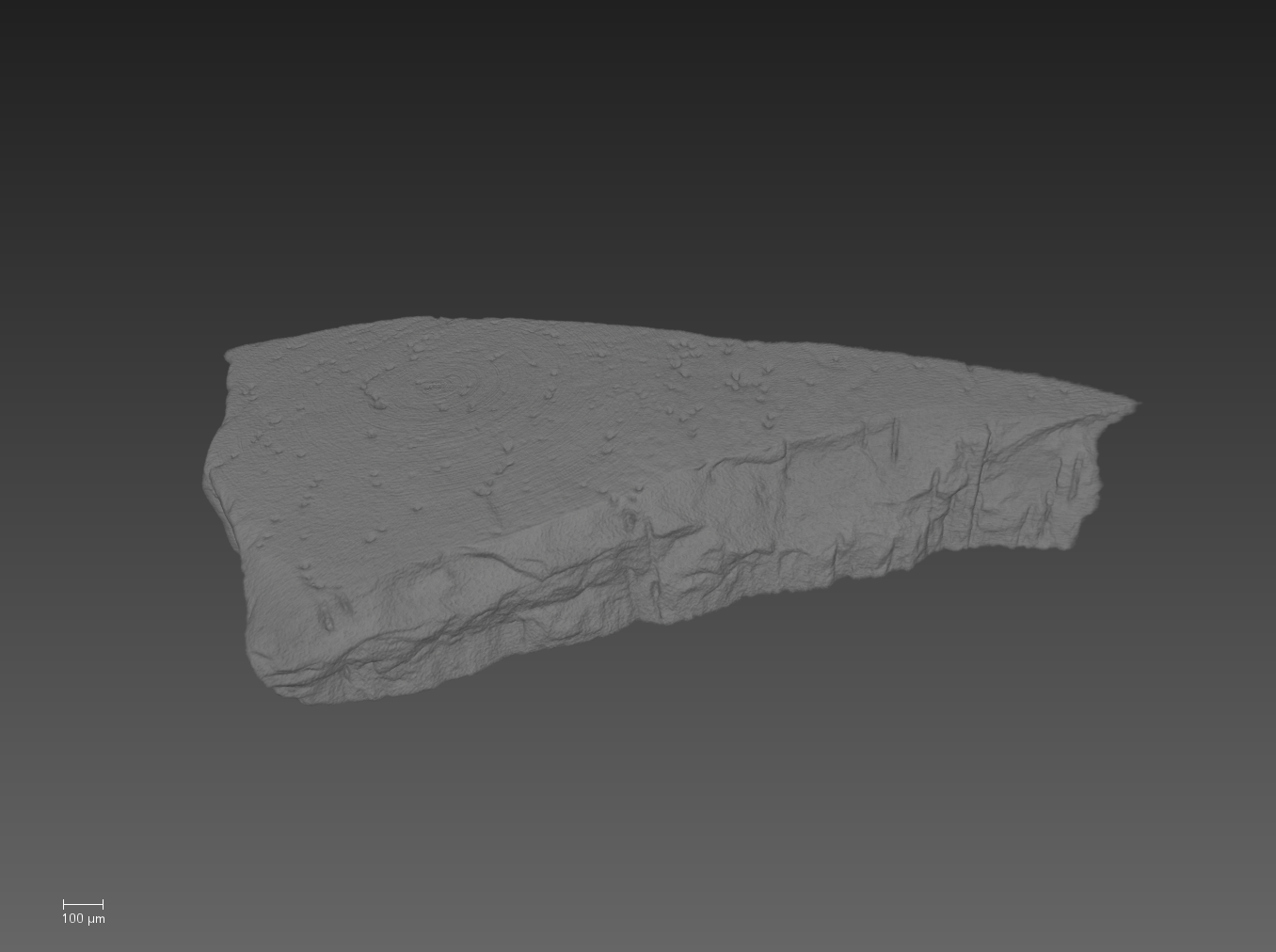

Supplement: Supplementary file 12 — Supplementary Data 12 [file 41467_2023_36405_MOESM12_ESM.zip › Micro_CT_raw_data/Southern_Aepyornis_thin/AD2135/Results/Outer surface.tif]

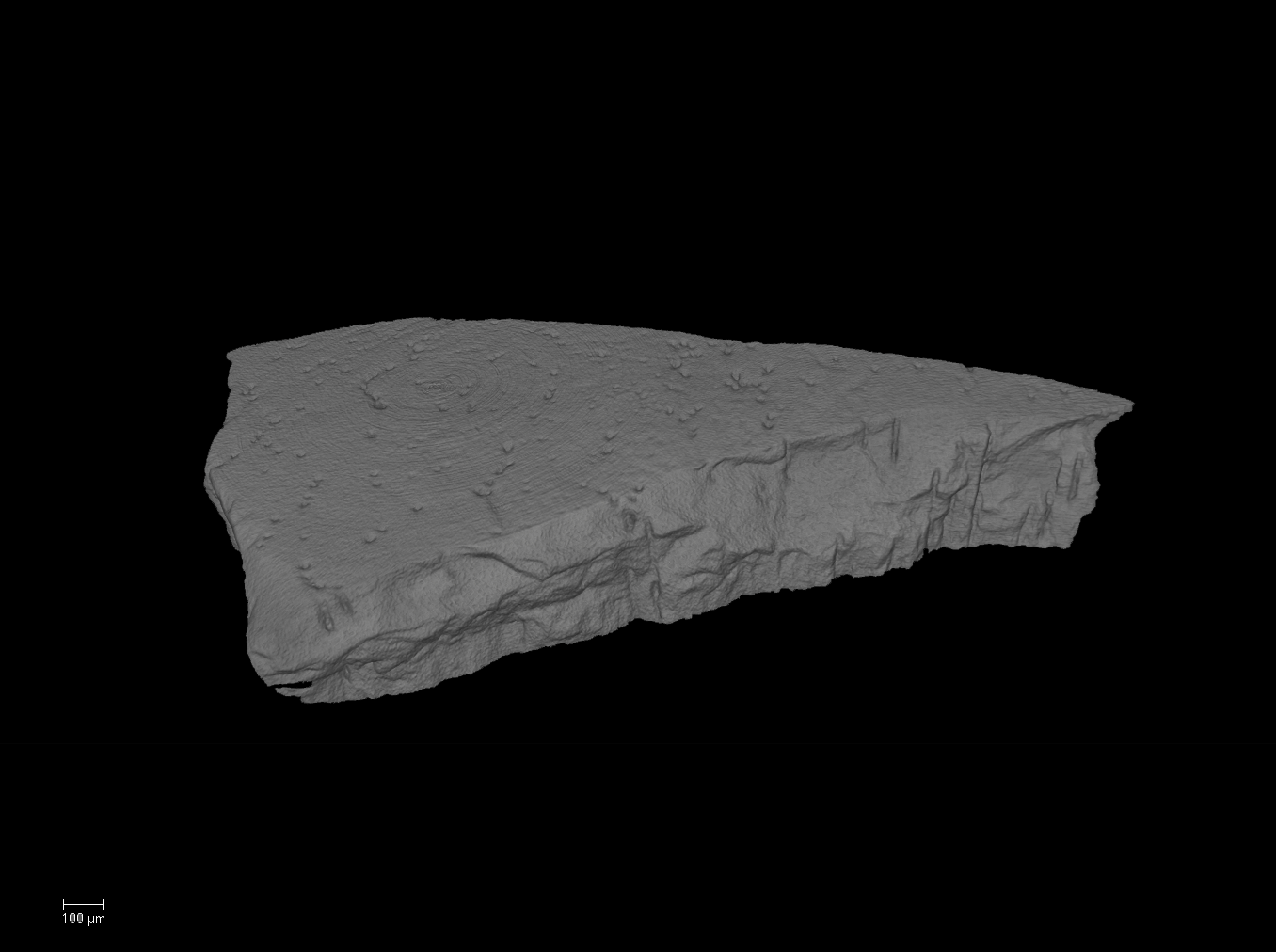

Supplement: Supplementary file 12 — Supplementary Data 12 [file 41467_2023_36405_MOESM12_ESM.zip › Micro_CT_raw_data/Southern_Aepyornis_thin/AD2135/Results/Outer surface2.tif]

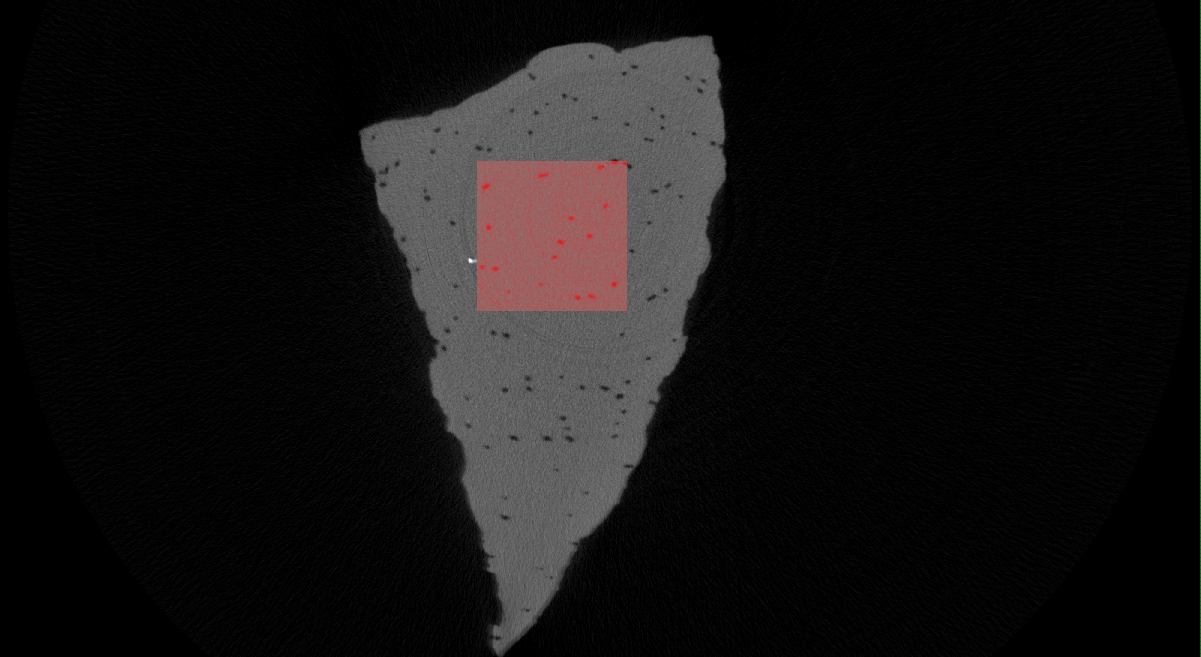

Supplement: Supplementary file 12 — Supplementary Data 12 [file 41467_2023_36405_MOESM12_ESM.zip › Micro_CT_raw_data/Southern_Aepyornis_thin/AD2135/Results/ROI Selection.tif]

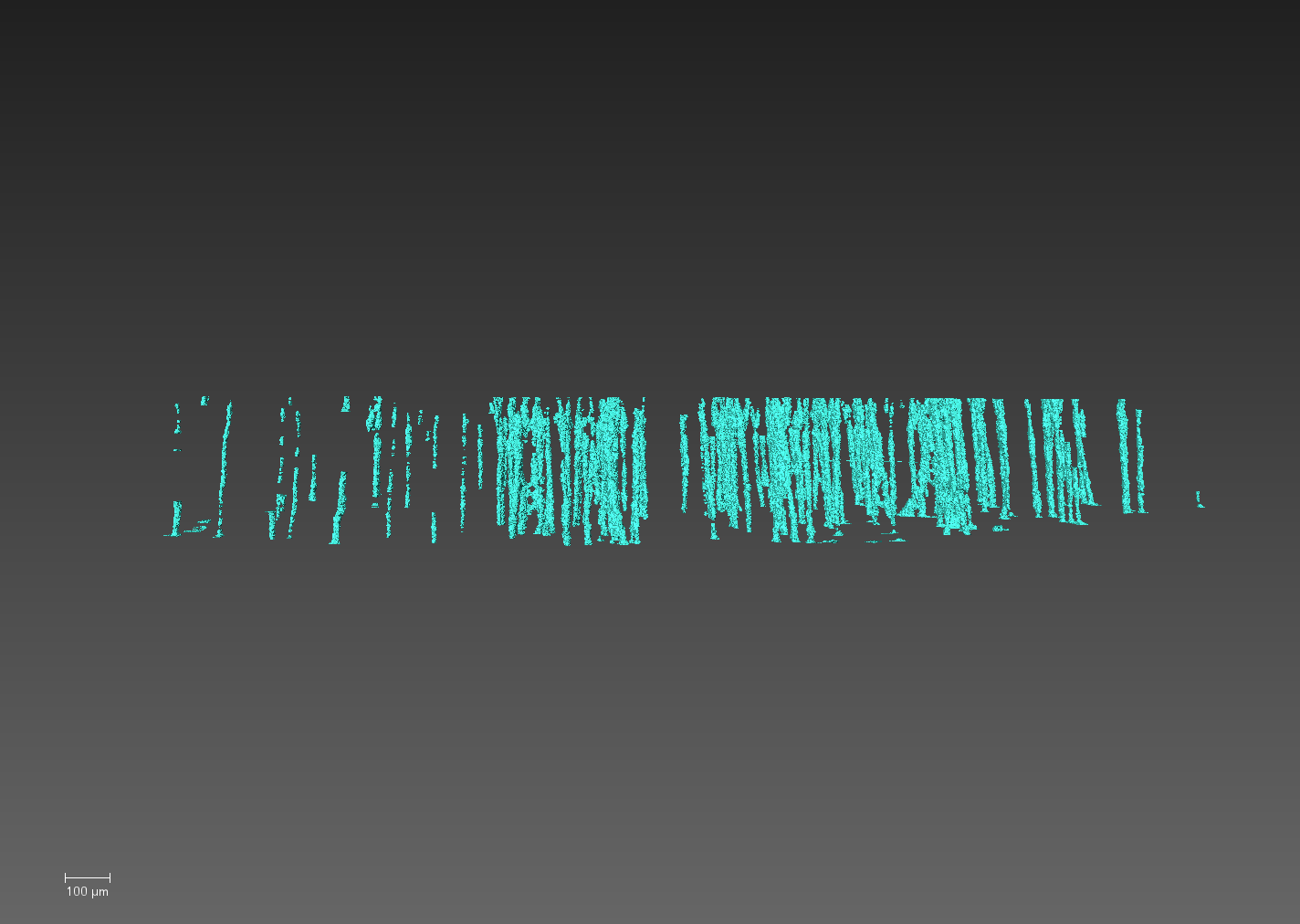

Supplement: Supplementary file 12 — Supplementary Data 12 [file 41467_2023_36405_MOESM12_ESM.zip › Micro_CT_raw_data/Southern_Aepyornis_thin/AD2135/Results/Pore structure1.tif]

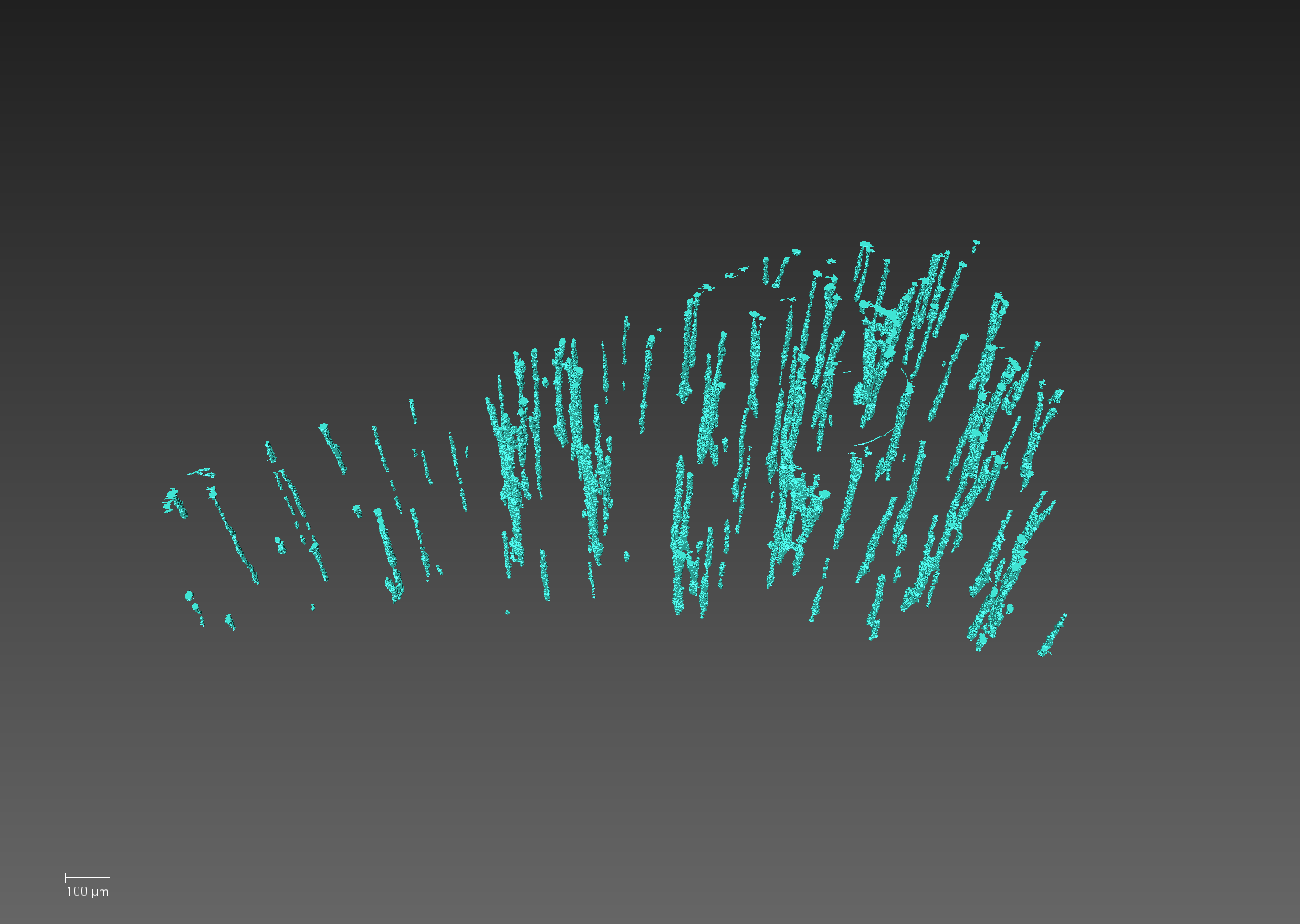

Supplement: Supplementary file 12 — Supplementary Data 12 [file 41467_2023_36405_MOESM12_ESM.zip › Micro_CT_raw_data/Southern_Aepyornis_thin/AD2135/Results/Pore structure2.tif]

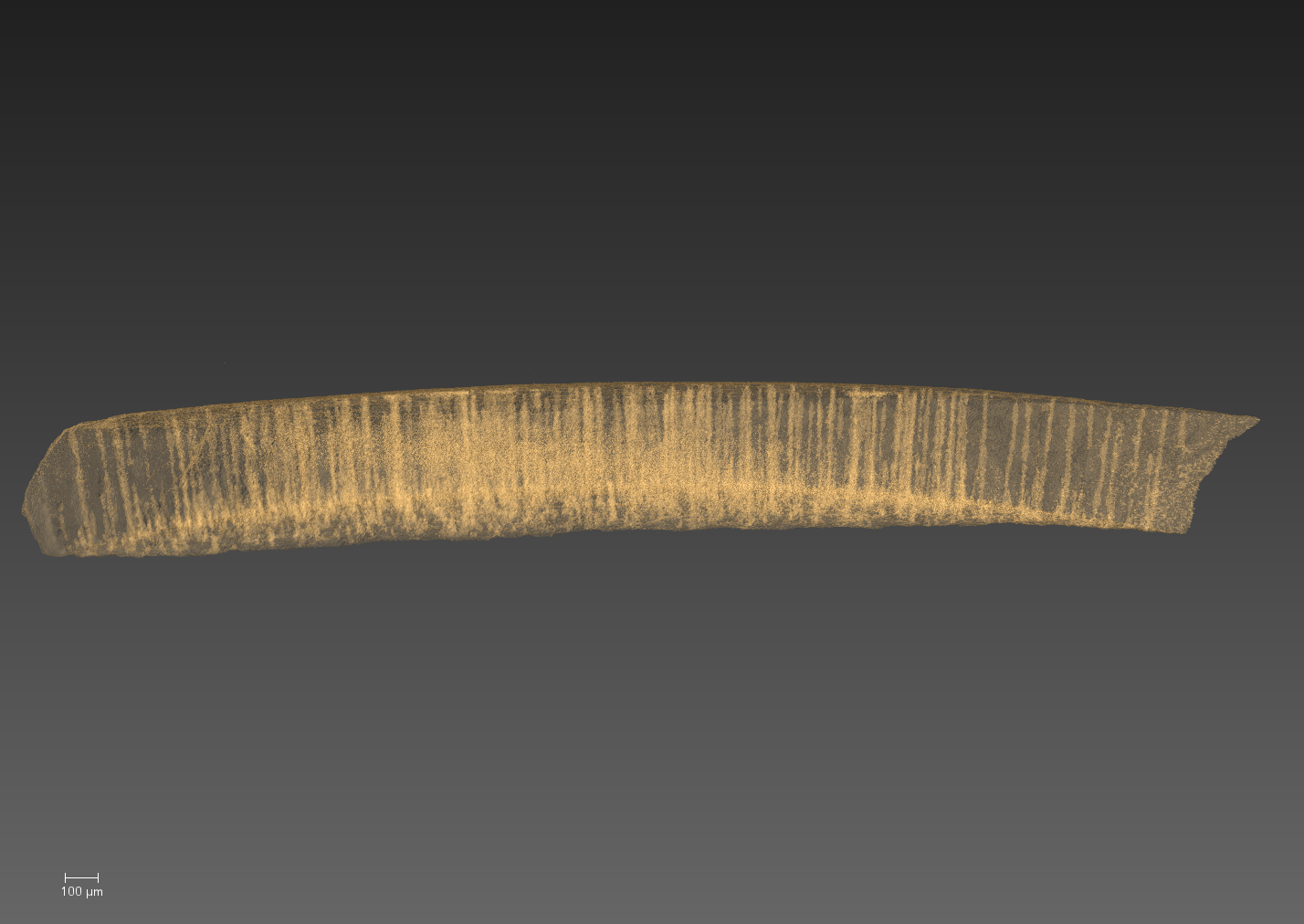

Supplement: Supplementary file 12 — Supplementary Data 12 [file 41467_2023_36405_MOESM12_ESM.zip › Micro_CT_raw_data/Southern_Aepyornis_thin/AD2387/Results/snapshot2.tif]

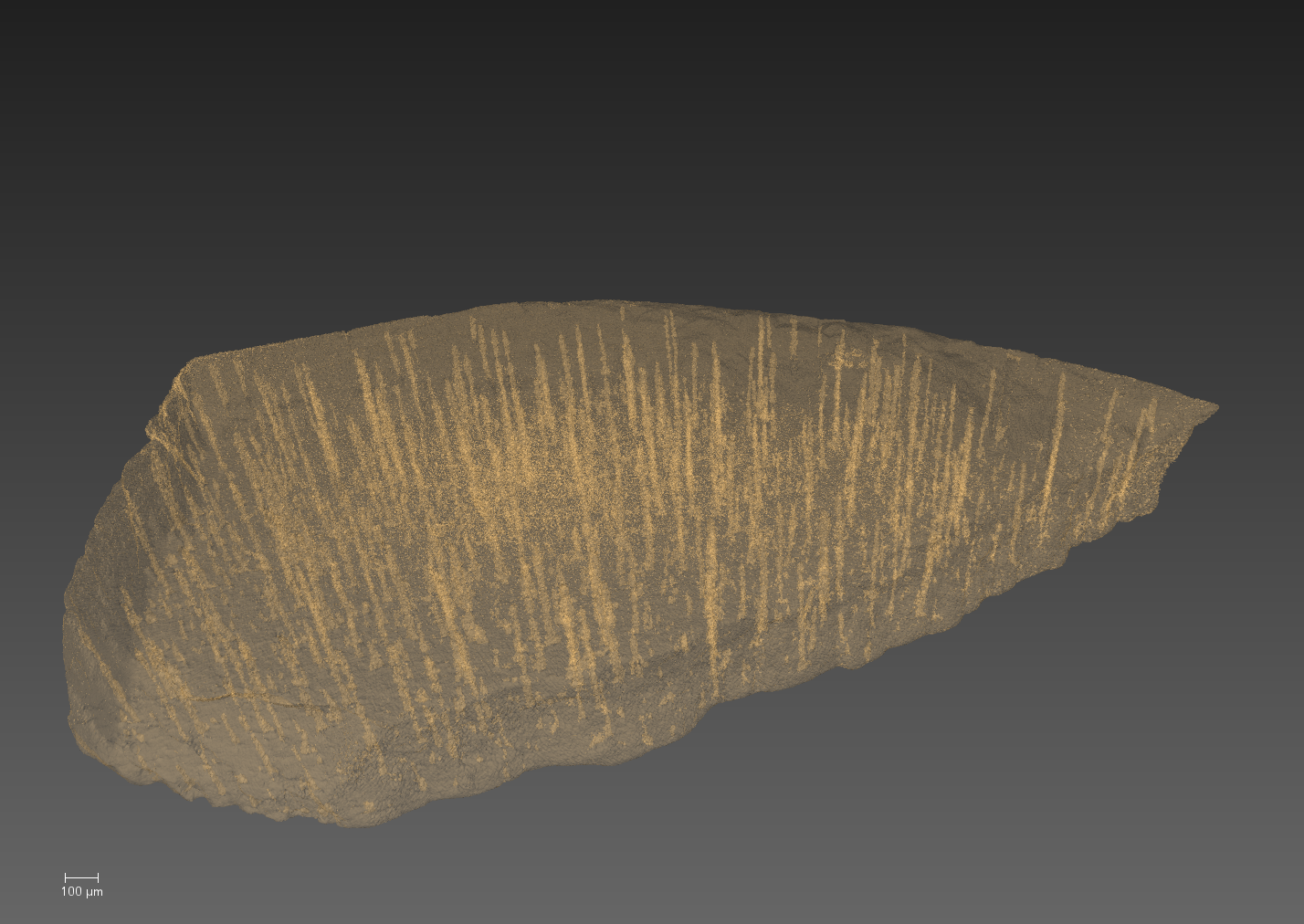

Supplement: Supplementary file 12 — Supplementary Data 12 [file 41467_2023_36405_MOESM12_ESM.zip › Micro_CT_raw_data/Southern_Aepyornis_thin/AD2387/Results/snapshot1.tif]

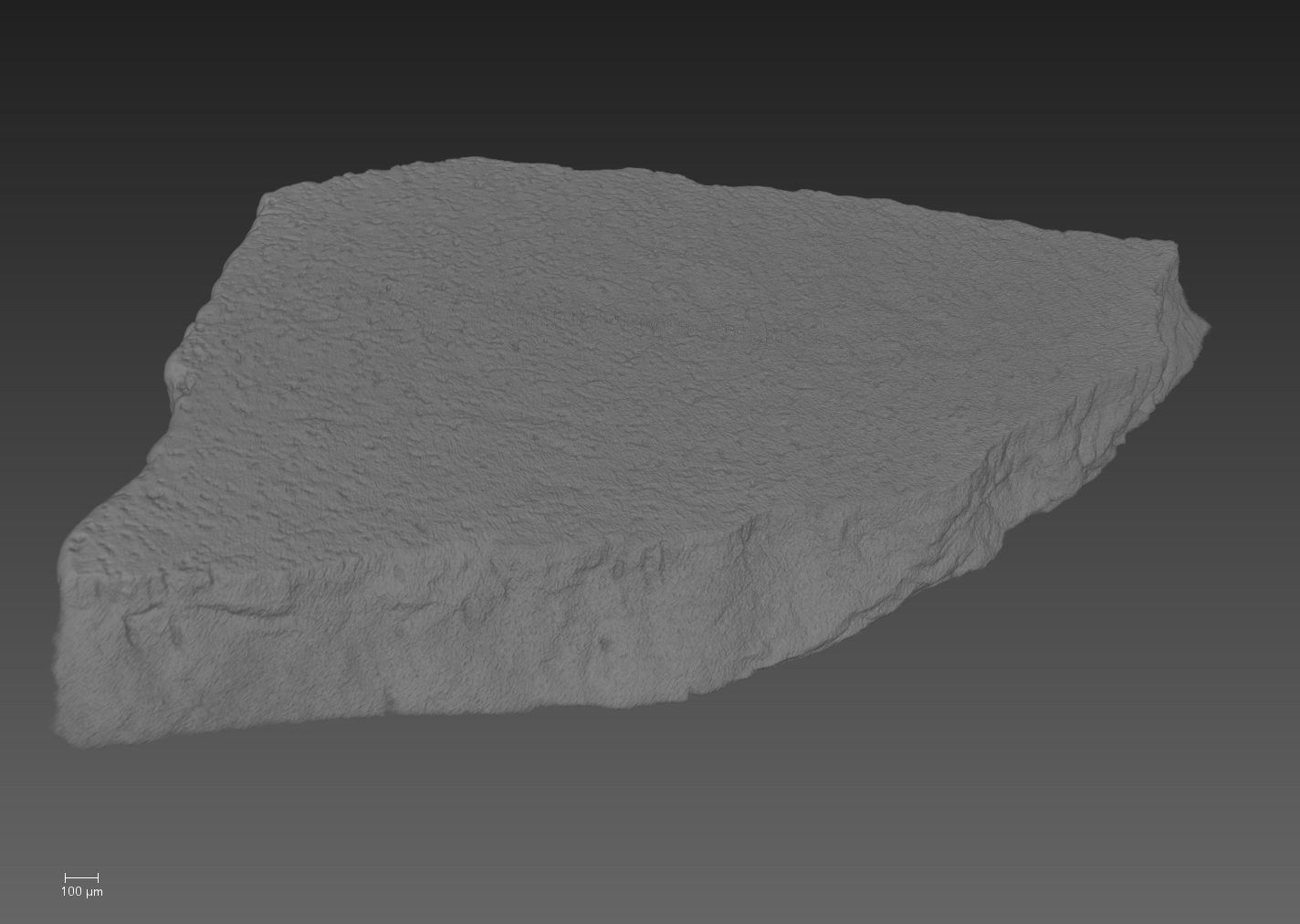

Supplement: Supplementary file 12 — Supplementary Data 12 [file 41467_2023_36405_MOESM12_ESM.zip › Micro_CT_raw_data/Southern_Aepyornis_thin/AD2387/Results/Inner surface.tif]

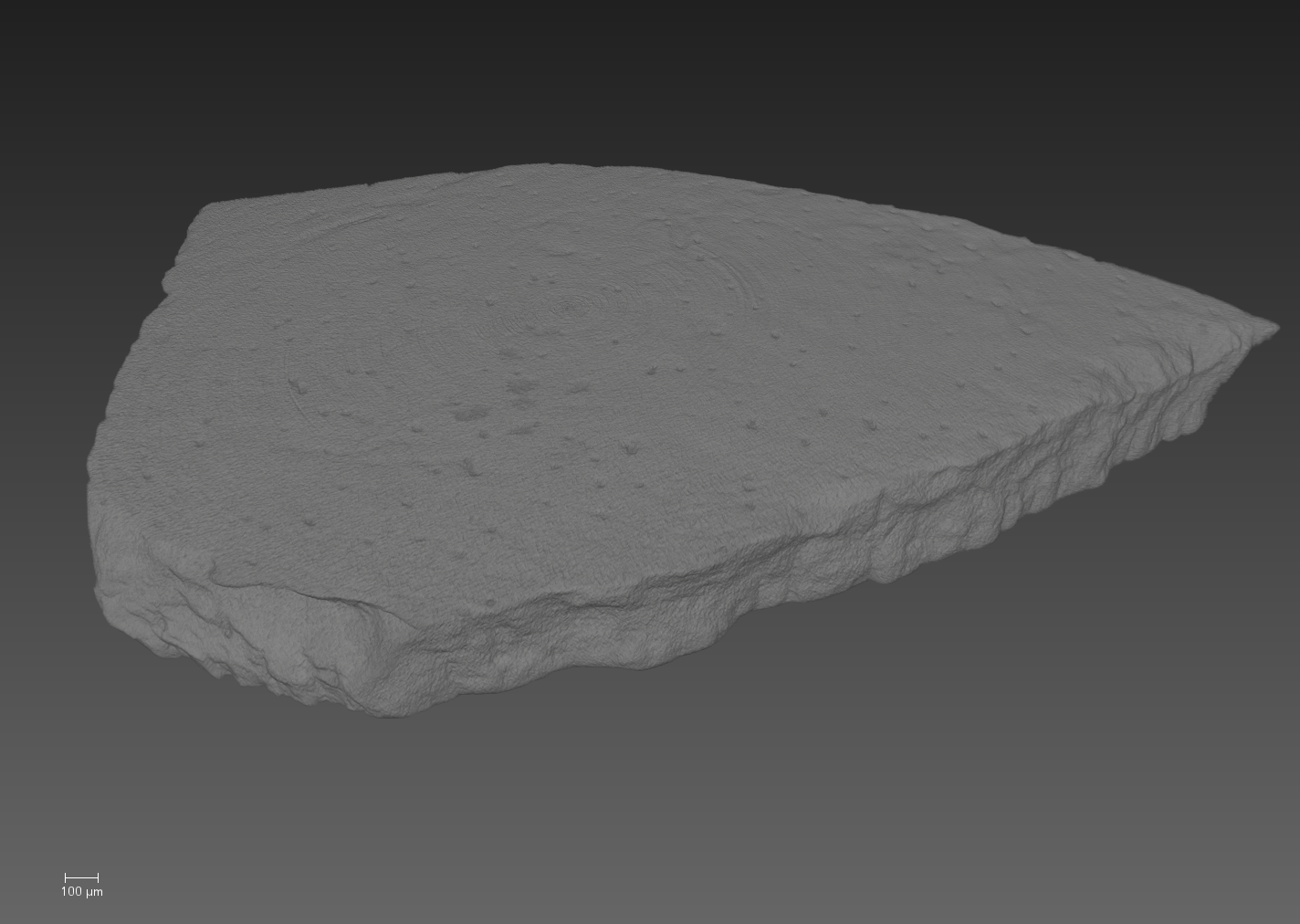

Supplement: Supplementary file 12 — Supplementary Data 12 [file 41467_2023_36405_MOESM12_ESM.zip › Micro_CT_raw_data/Southern_Aepyornis_thin/AD2387/Results/Outer surface.tif]

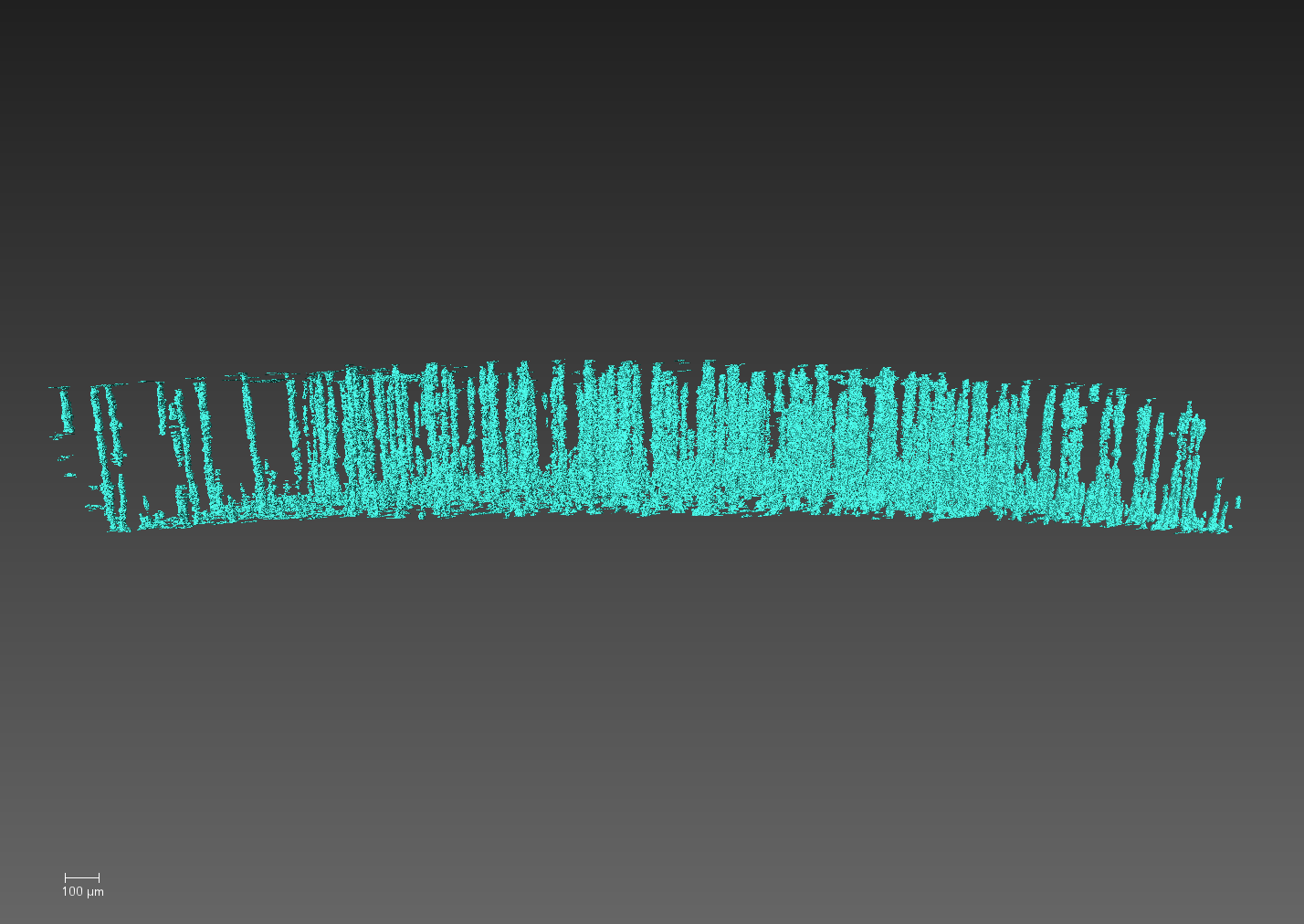

Supplement: Supplementary file 12 — Supplementary Data 12 [file 41467_2023_36405_MOESM12_ESM.zip › Micro_CT_raw_data/Southern_Aepyornis_thin/AD2387/Results/Pore structure.tif]

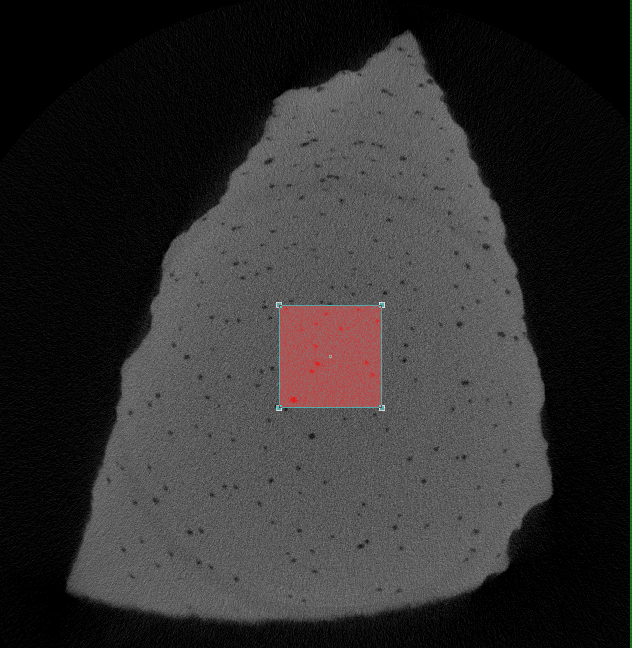

Supplement: Supplementary file 12 — Supplementary Data 12 [file 41467_2023_36405_MOESM12_ESM.zip › Micro_CT_raw_data/Southern_Aepyornis_thin/AD2387/Results/ROI Selection.tif]

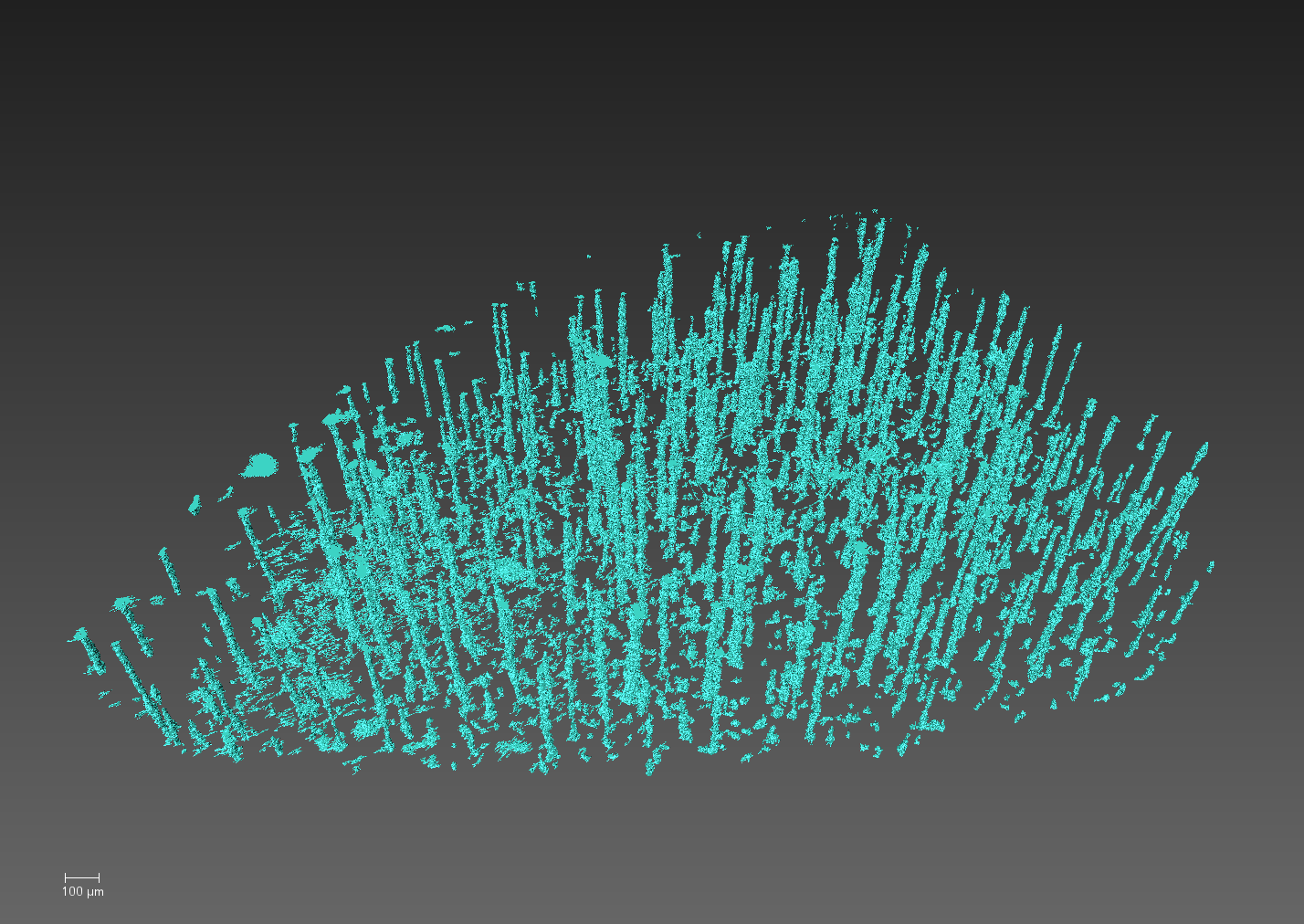

Supplement: Supplementary file 12 — Supplementary Data 12 [file 41467_2023_36405_MOESM12_ESM.zip › Micro_CT_raw_data/Southern_Aepyornis_thin/AD2387/Results/Pore structure2.tif]

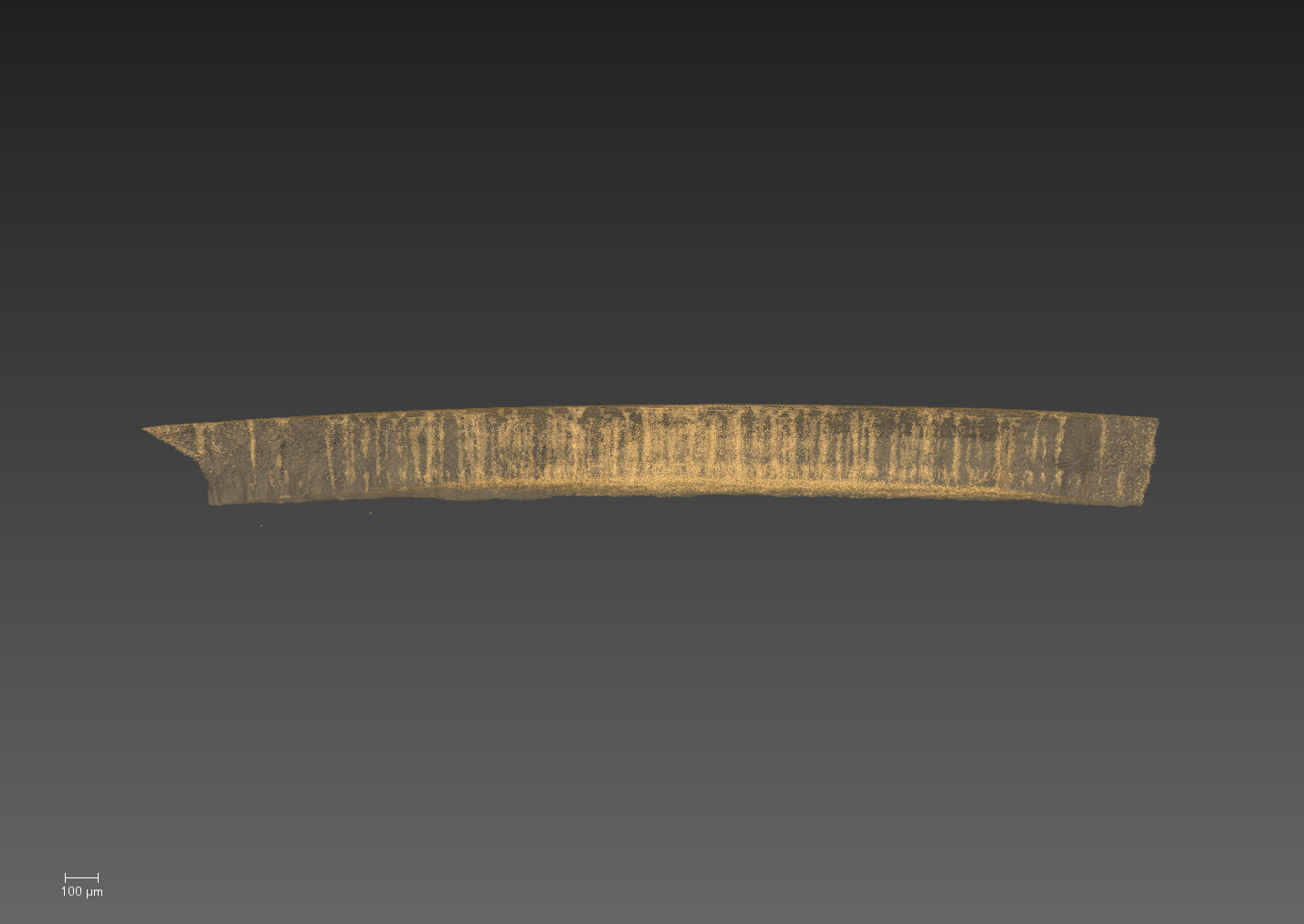

Supplement: Supplementary file 12 — Supplementary Data 12 [file 41467_2023_36405_MOESM12_ESM.zip › Micro_CT_raw_data/Northern_Aepyornis/AD2140/Results/snapshot2.tif]

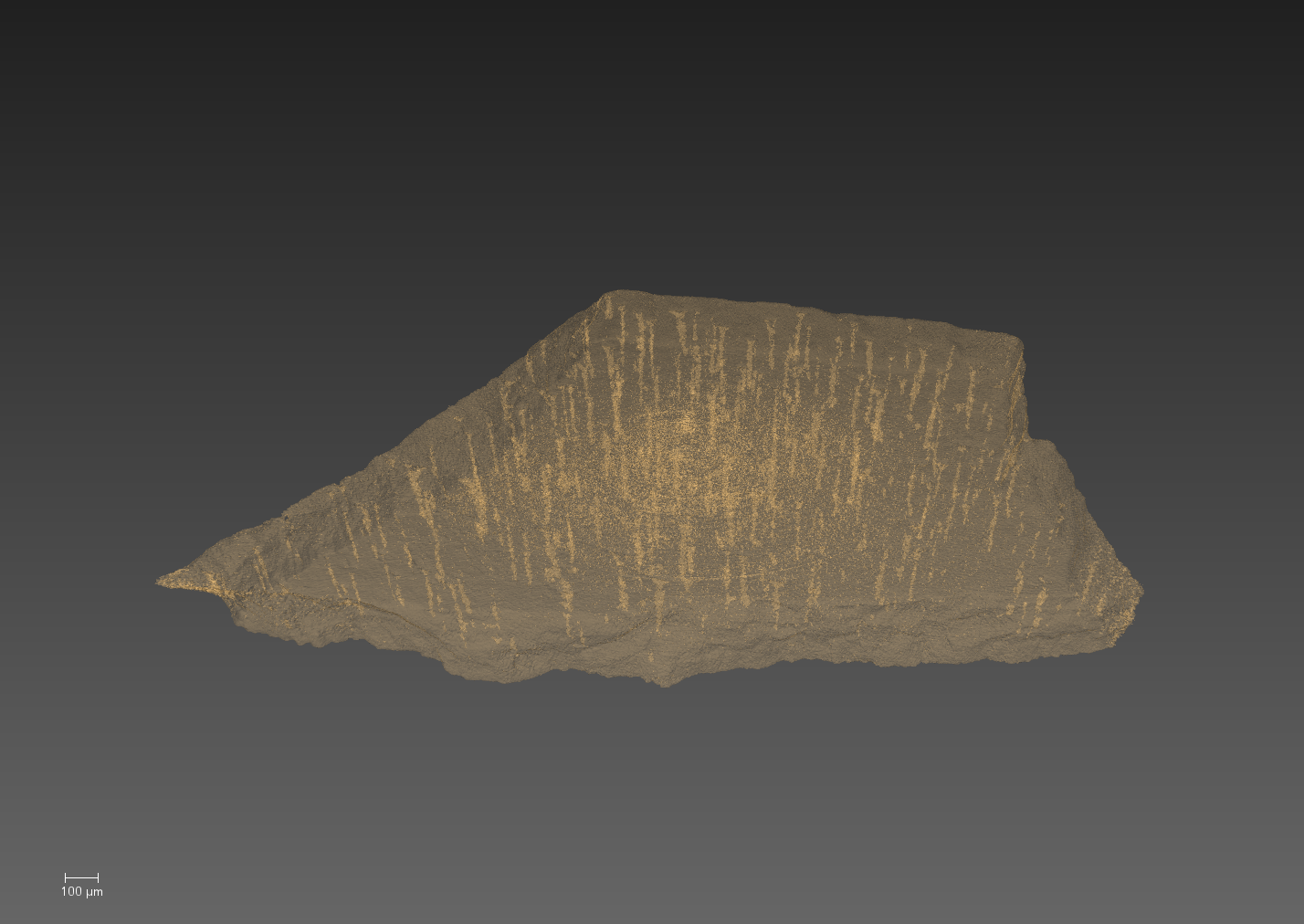

Supplement: Supplementary file 12 — Supplementary Data 12 [file 41467_2023_36405_MOESM12_ESM.zip › Micro_CT_raw_data/Northern_Aepyornis/AD2140/Results/snapshot1.tif]

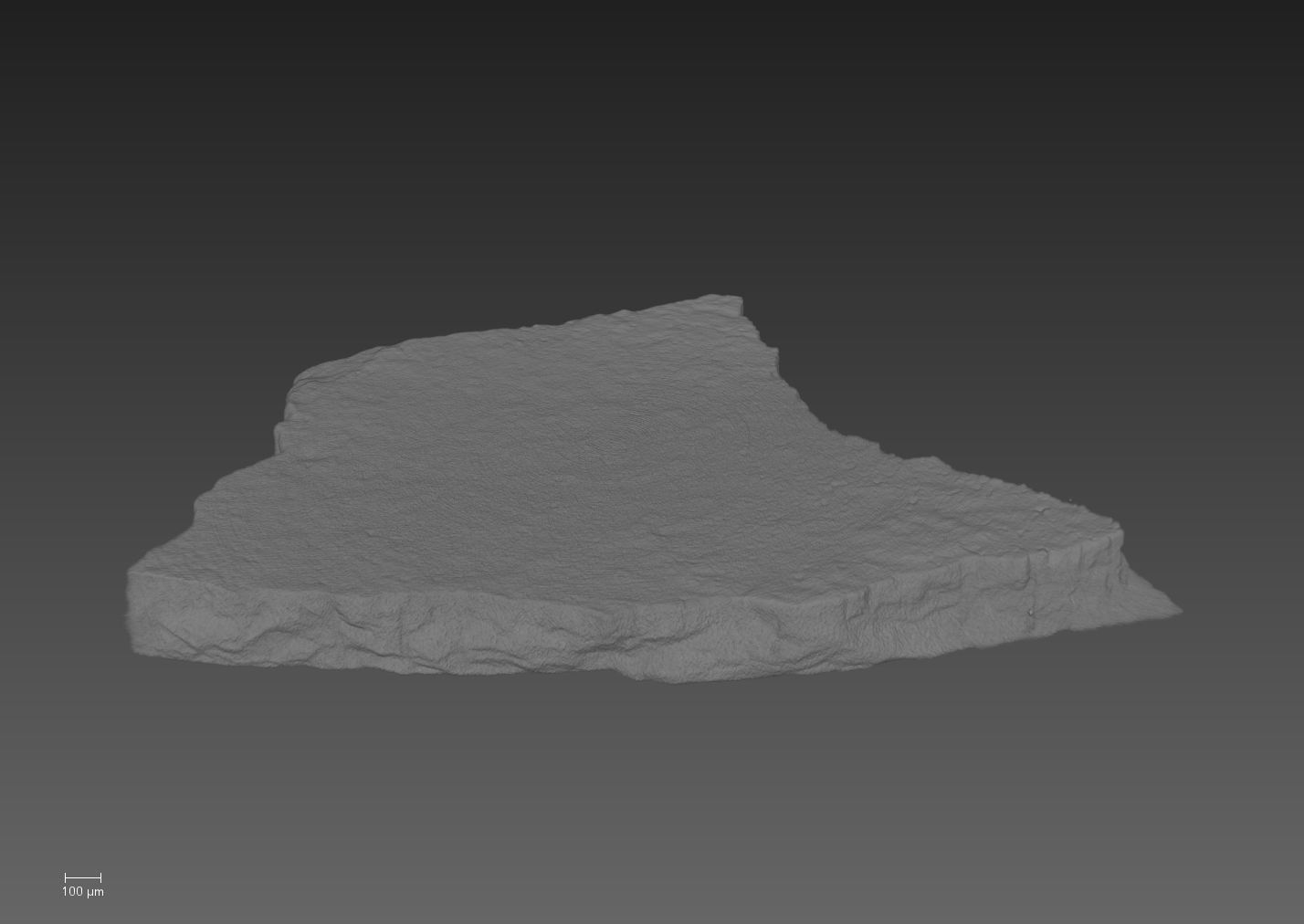

Supplement: Supplementary file 12 — Supplementary Data 12 [file 41467_2023_36405_MOESM12_ESM.zip › Micro_CT_raw_data/Northern_Aepyornis/AD2140/Results/Inner surface.tif]

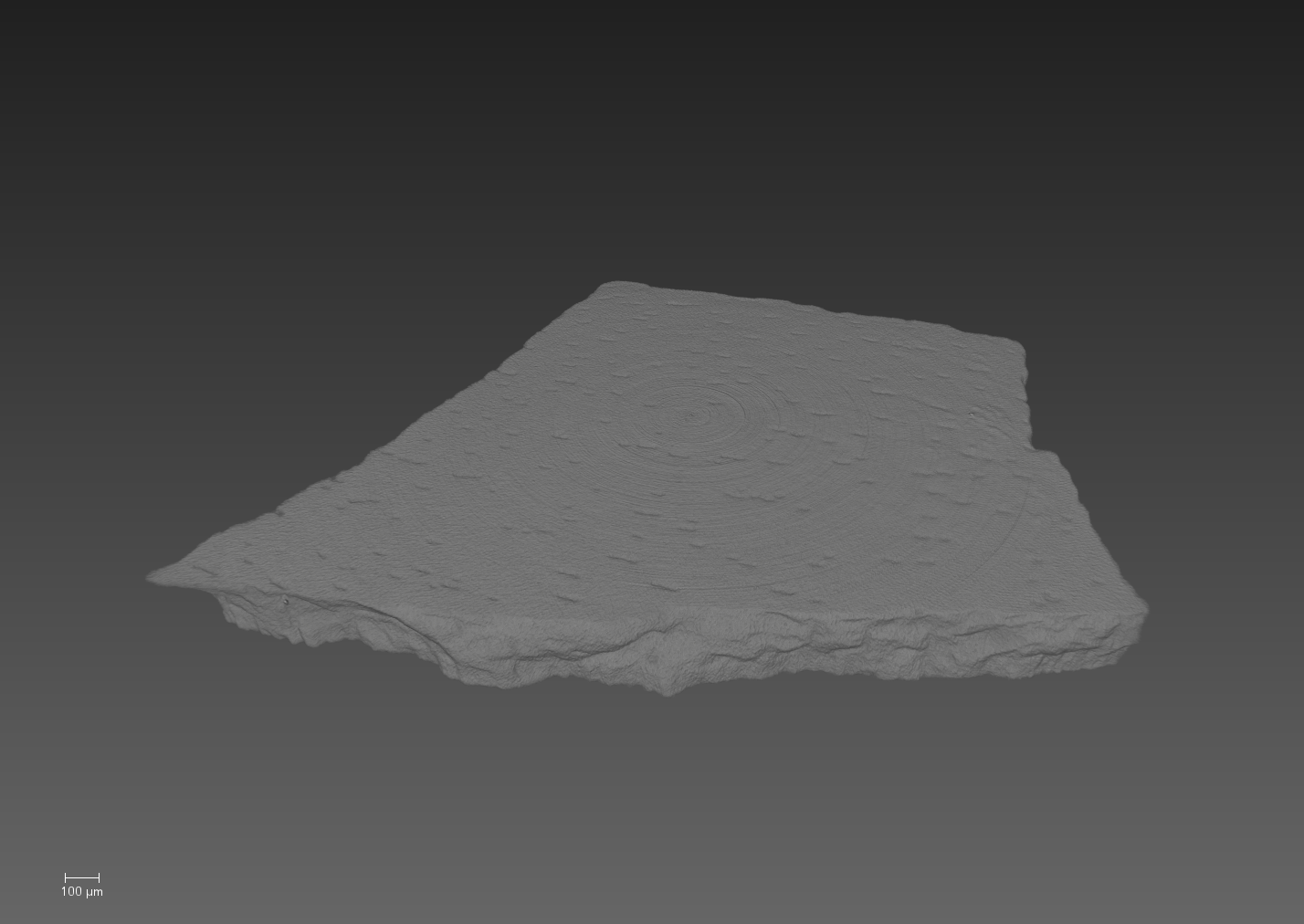

Supplement: Supplementary file 12 — Supplementary Data 12 [file 41467_2023_36405_MOESM12_ESM.zip › Micro_CT_raw_data/Northern_Aepyornis/AD2140/Results/Outer surface.tif]

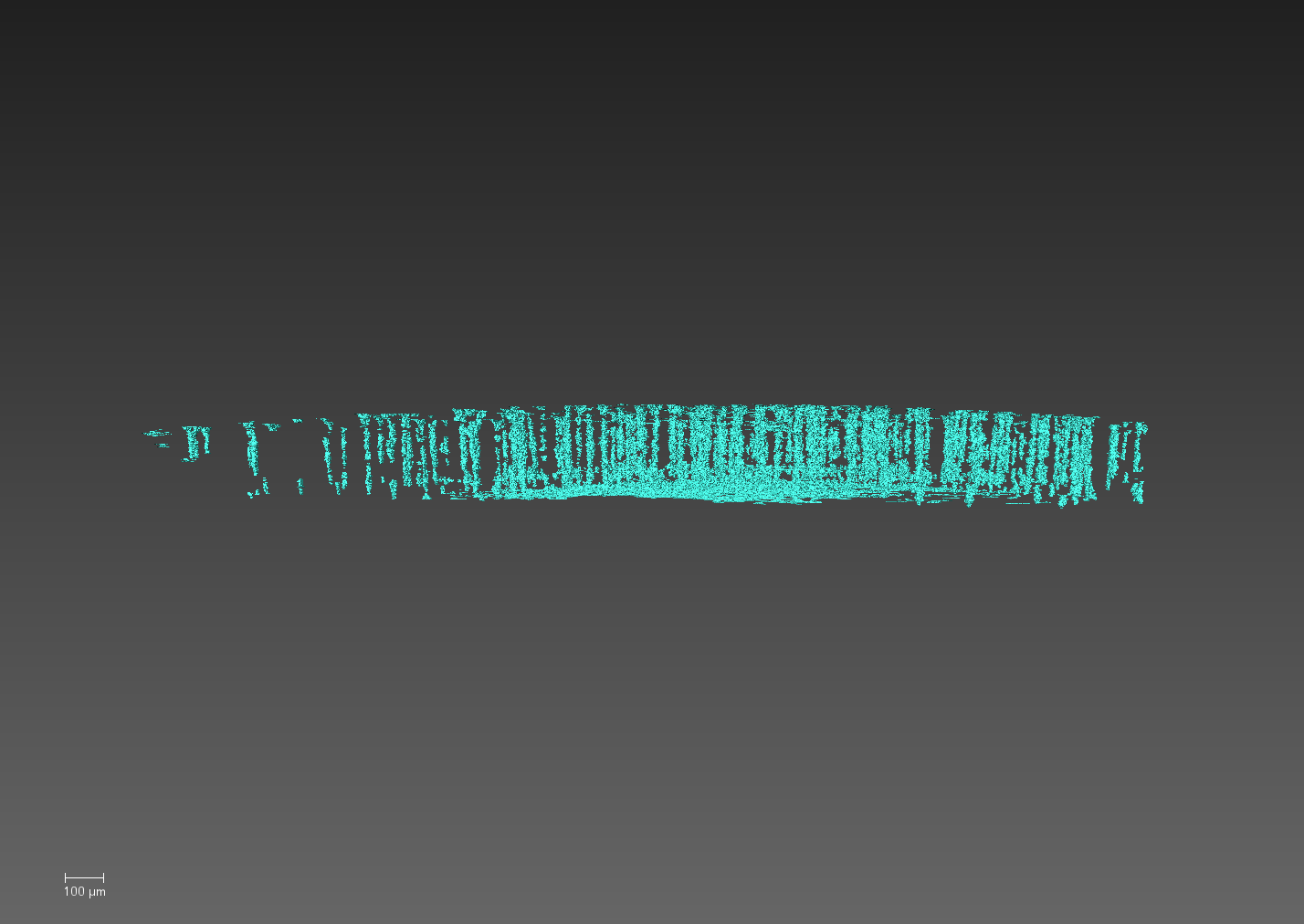

Supplement: Supplementary file 12 — Supplementary Data 12 [file 41467_2023_36405_MOESM12_ESM.zip › Micro_CT_raw_data/Northern_Aepyornis/AD2140/Results/Pore structure.tif]

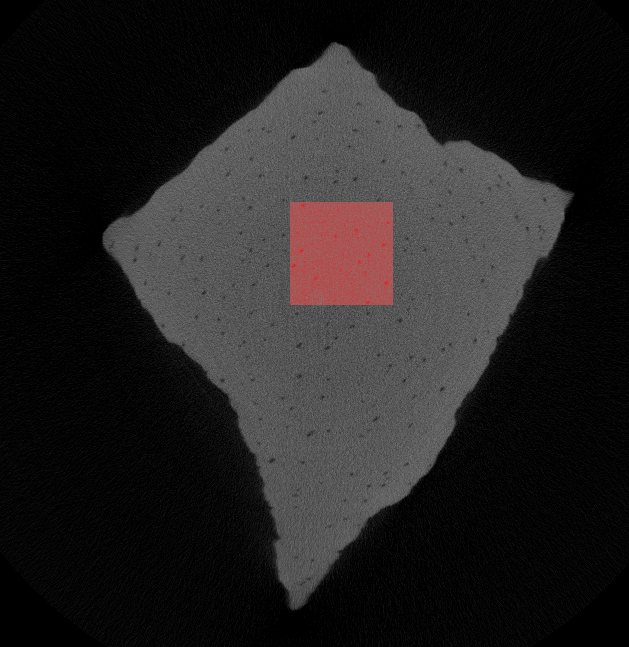

Supplement: Supplementary file 12 — Supplementary Data 12 [file 41467_2023_36405_MOESM12_ESM.zip › Micro_CT_raw_data/Northern_Aepyornis/AD2140/Results/ROI Selection.tif]

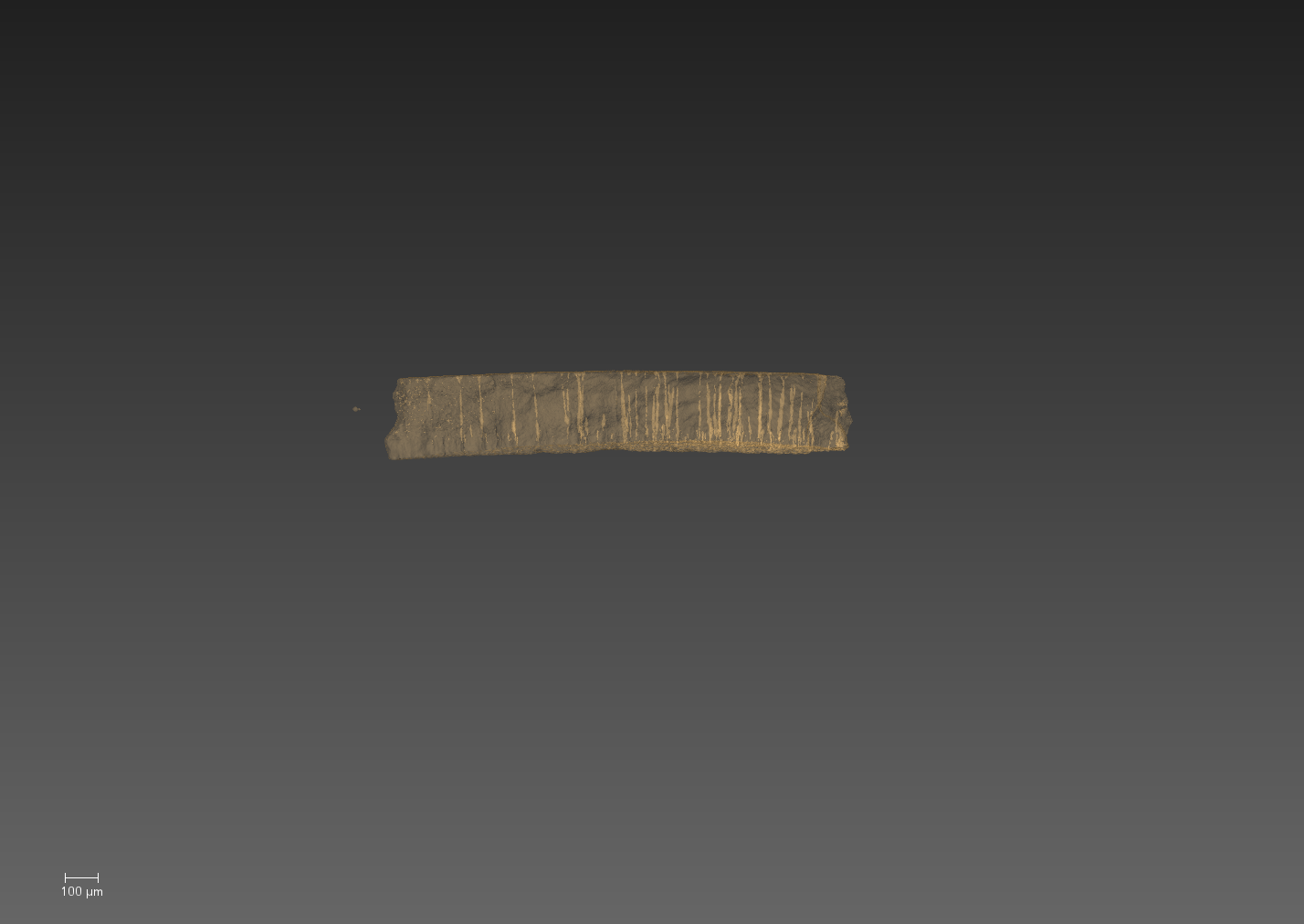

Supplement: Supplementary file 12 — Supplementary Data 12 [file 41467_2023_36405_MOESM12_ESM.zip › Micro_CT_raw_data/Northern_Aepyornis/AD1293/Results/snapshot2.tif]

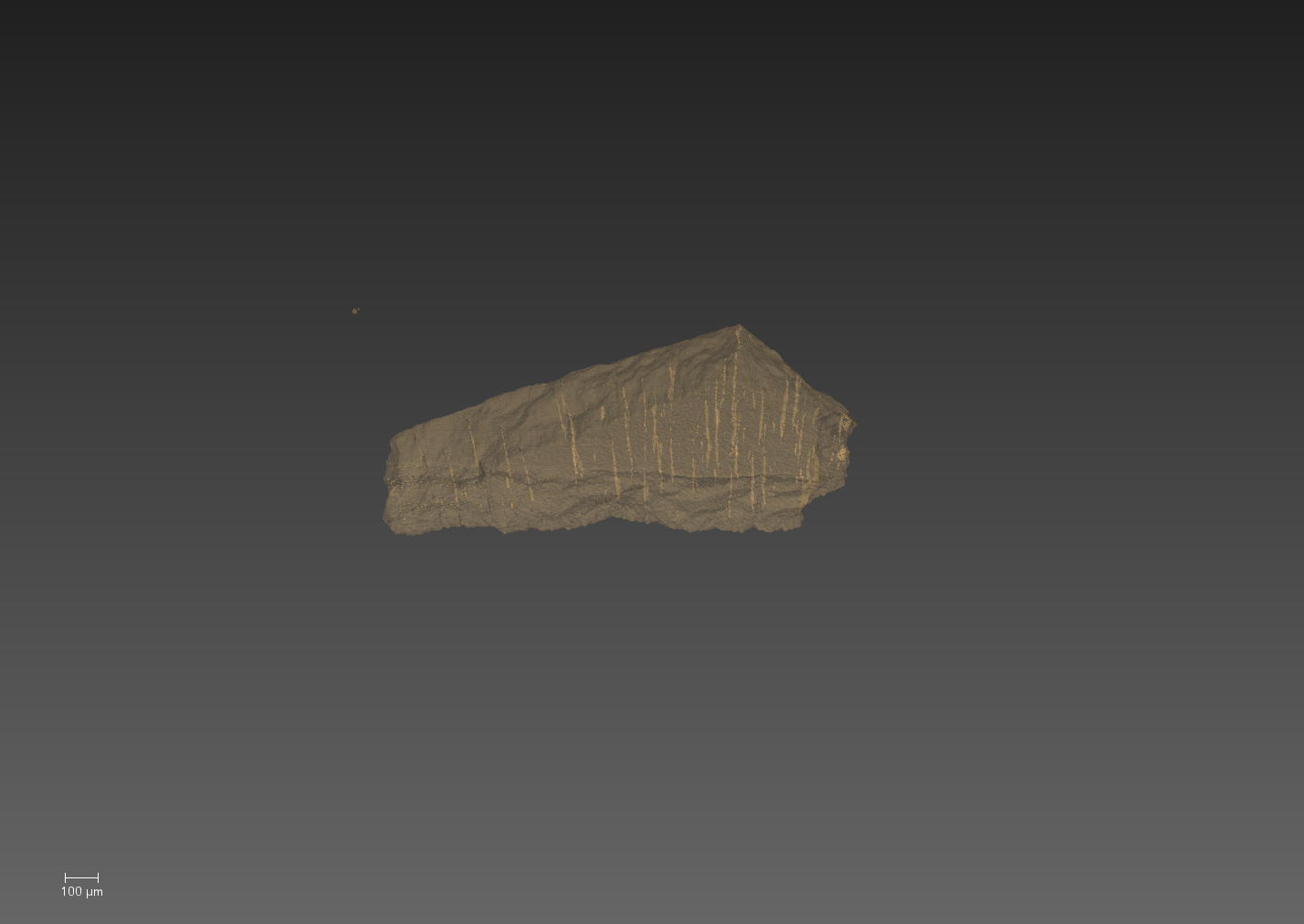

Supplement: Supplementary file 12 — Supplementary Data 12 [file 41467_2023_36405_MOESM12_ESM.zip › Micro_CT_raw_data/Northern_Aepyornis/AD1293/Results/snapshot.tif]

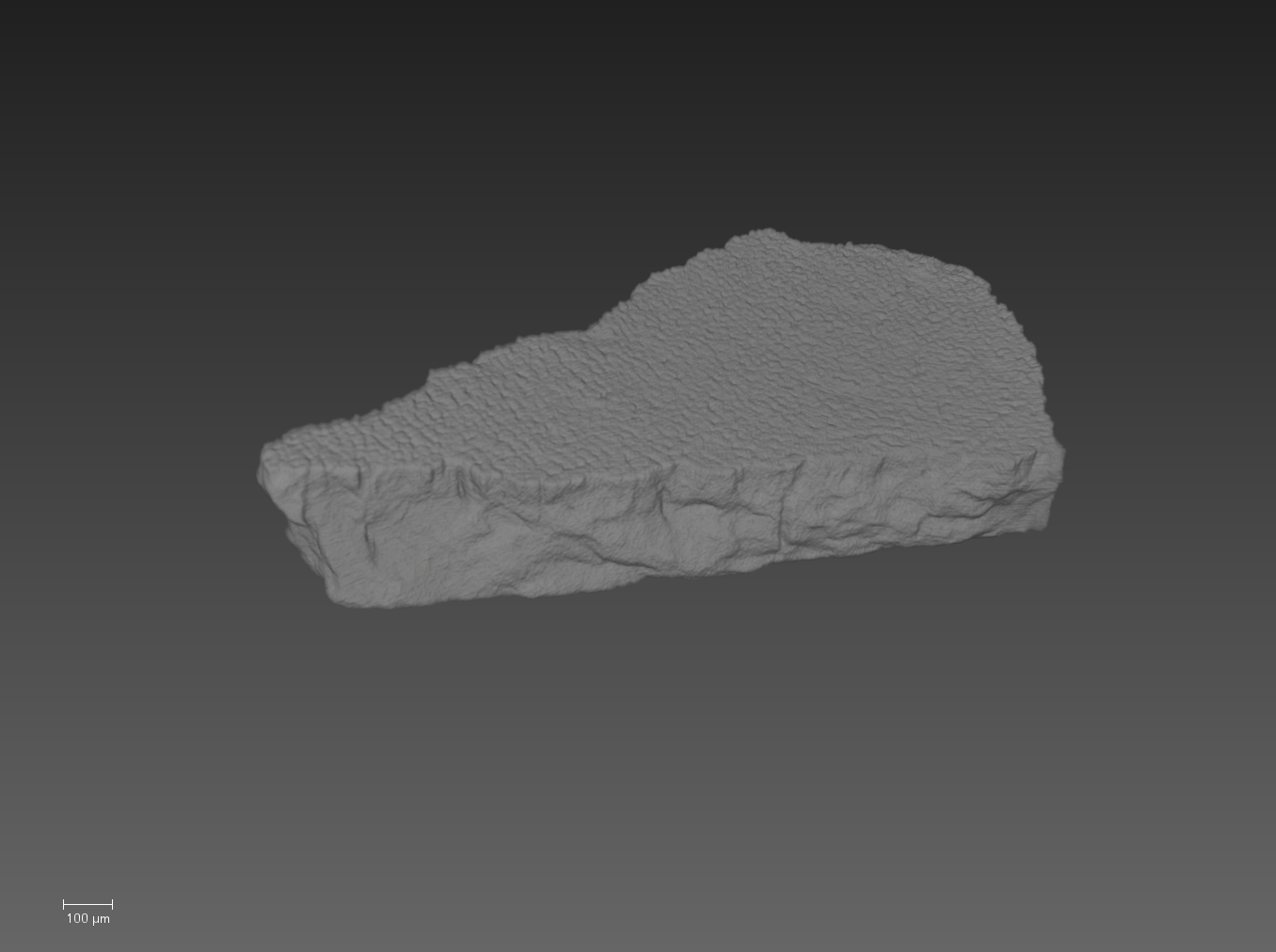

Supplement: Supplementary file 12 — Supplementary Data 12 [file 41467_2023_36405_MOESM12_ESM.zip › Micro_CT_raw_data/Northern_Aepyornis/AD1293/Results/Inner surface.tif]

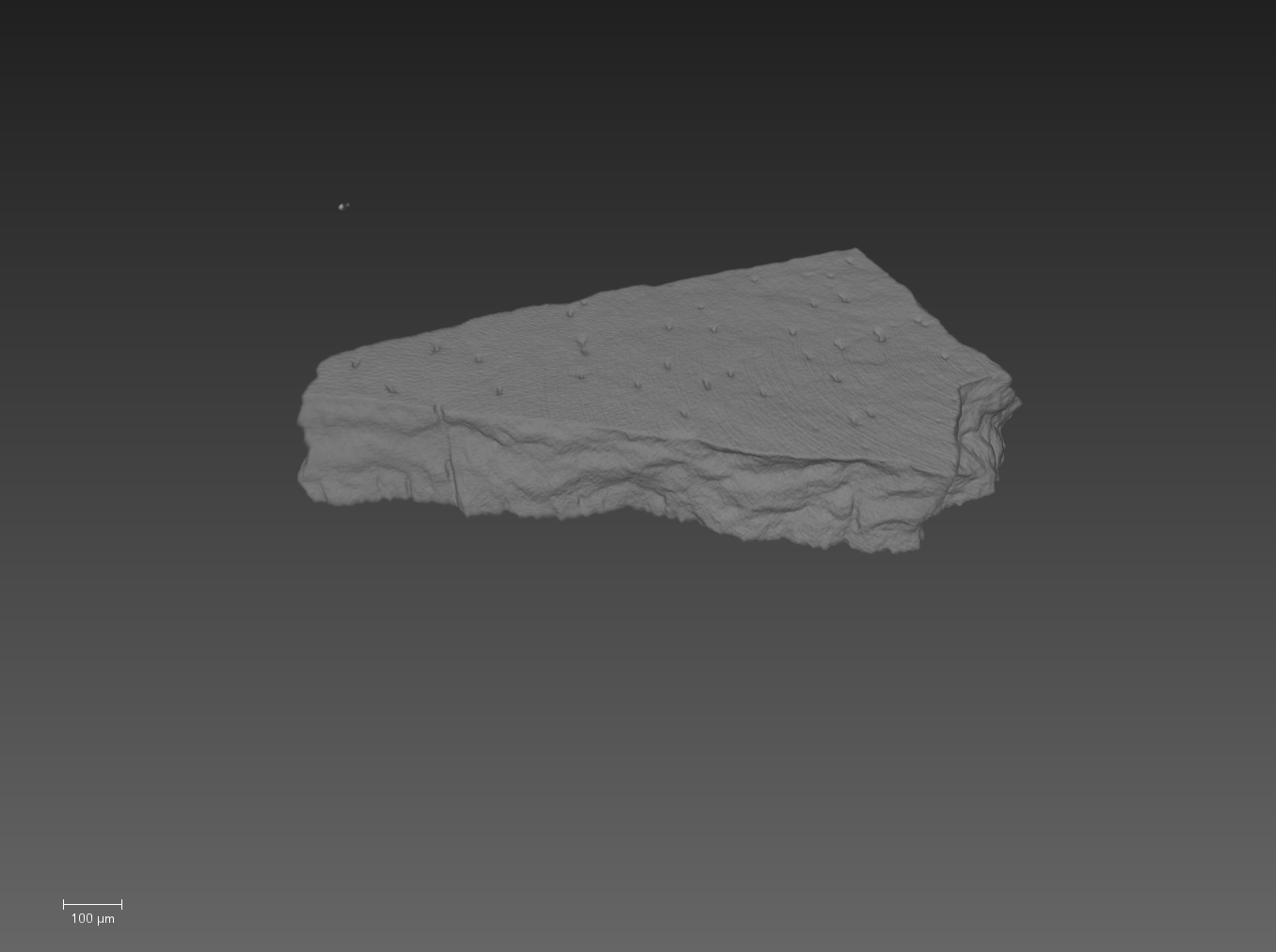

Supplement: Supplementary file 12 — Supplementary Data 12 [file 41467_2023_36405_MOESM12_ESM.zip › Micro_CT_raw_data/Northern_Aepyornis/AD1293/Results/Outer surface.tif]

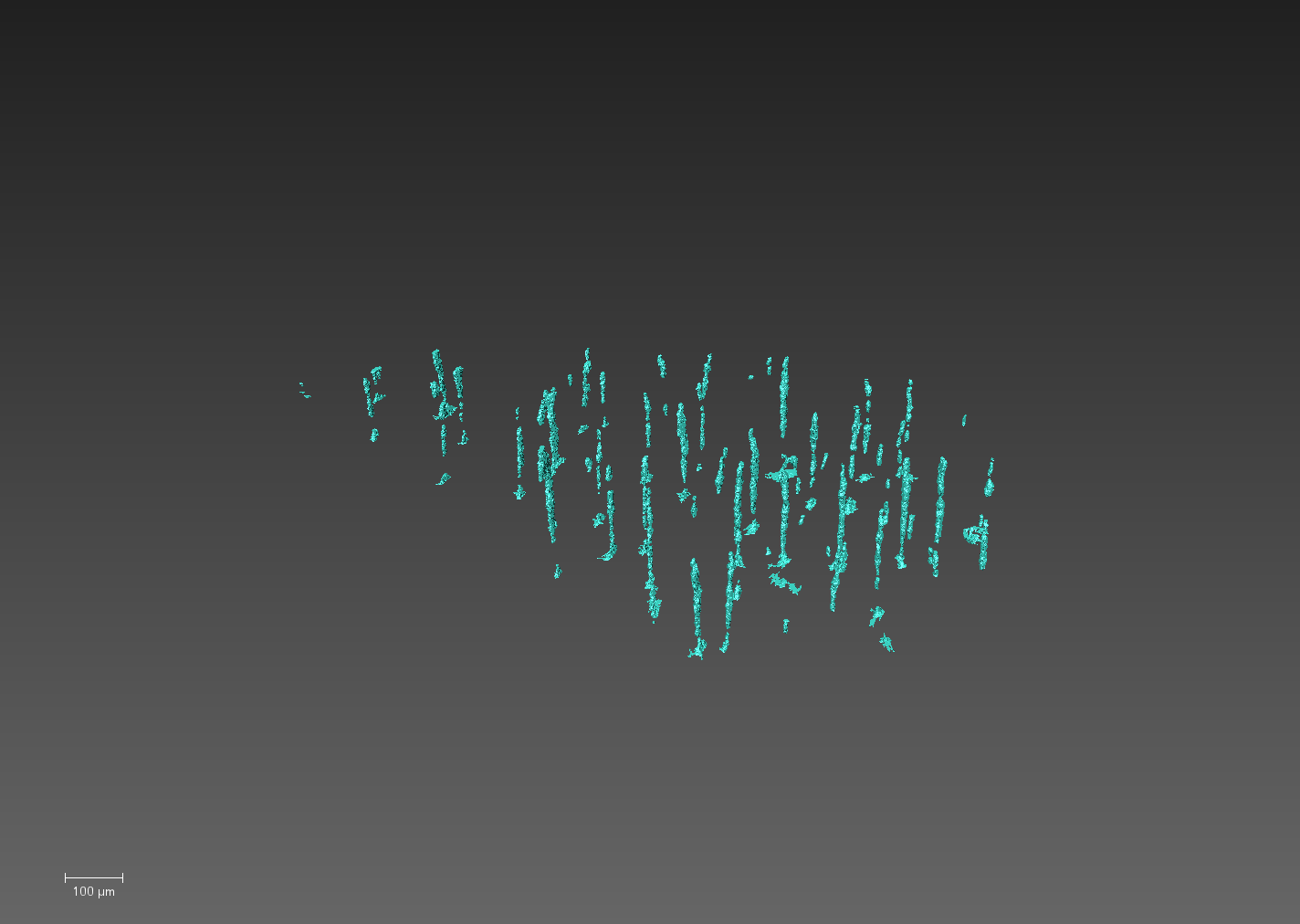

Supplement: Supplementary file 12 — Supplementary Data 12 [file 41467_2023_36405_MOESM12_ESM.zip › Micro_CT_raw_data/Northern_Aepyornis/AD1293/Results/Pore structure.tif]

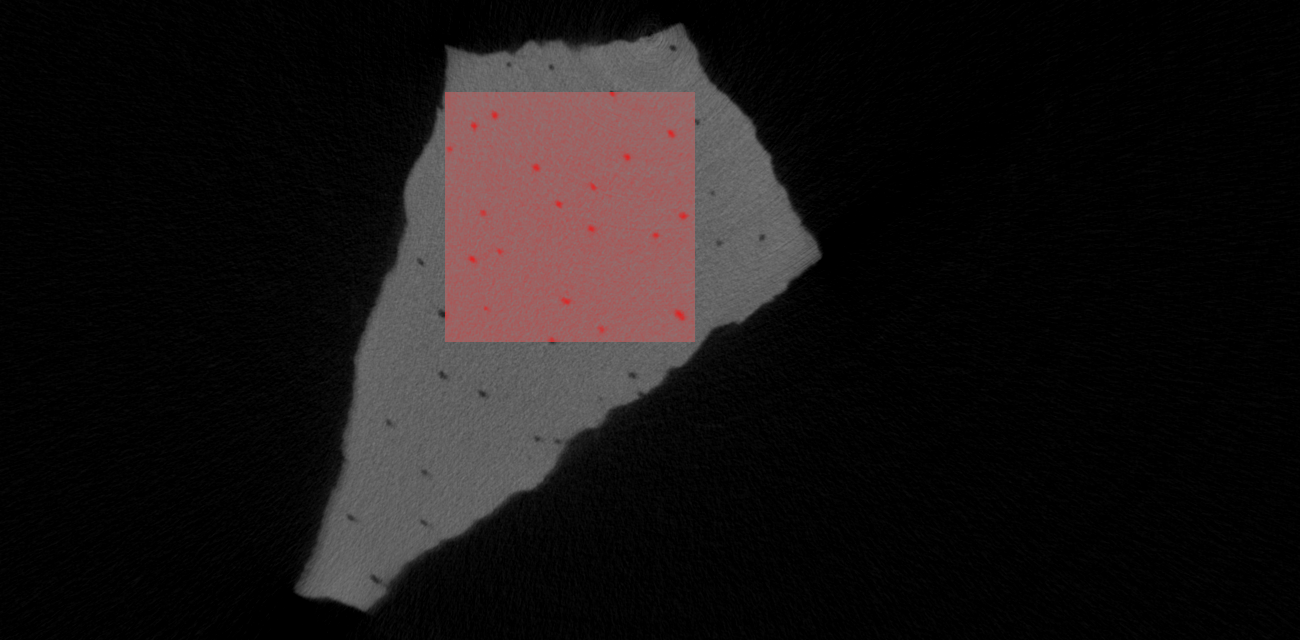

Supplement: Supplementary file 12 — Supplementary Data 12 [file 41467_2023_36405_MOESM12_ESM.zip › Micro_CT_raw_data/Northern_Aepyornis/AD1293/Results/ROI selection.tif]

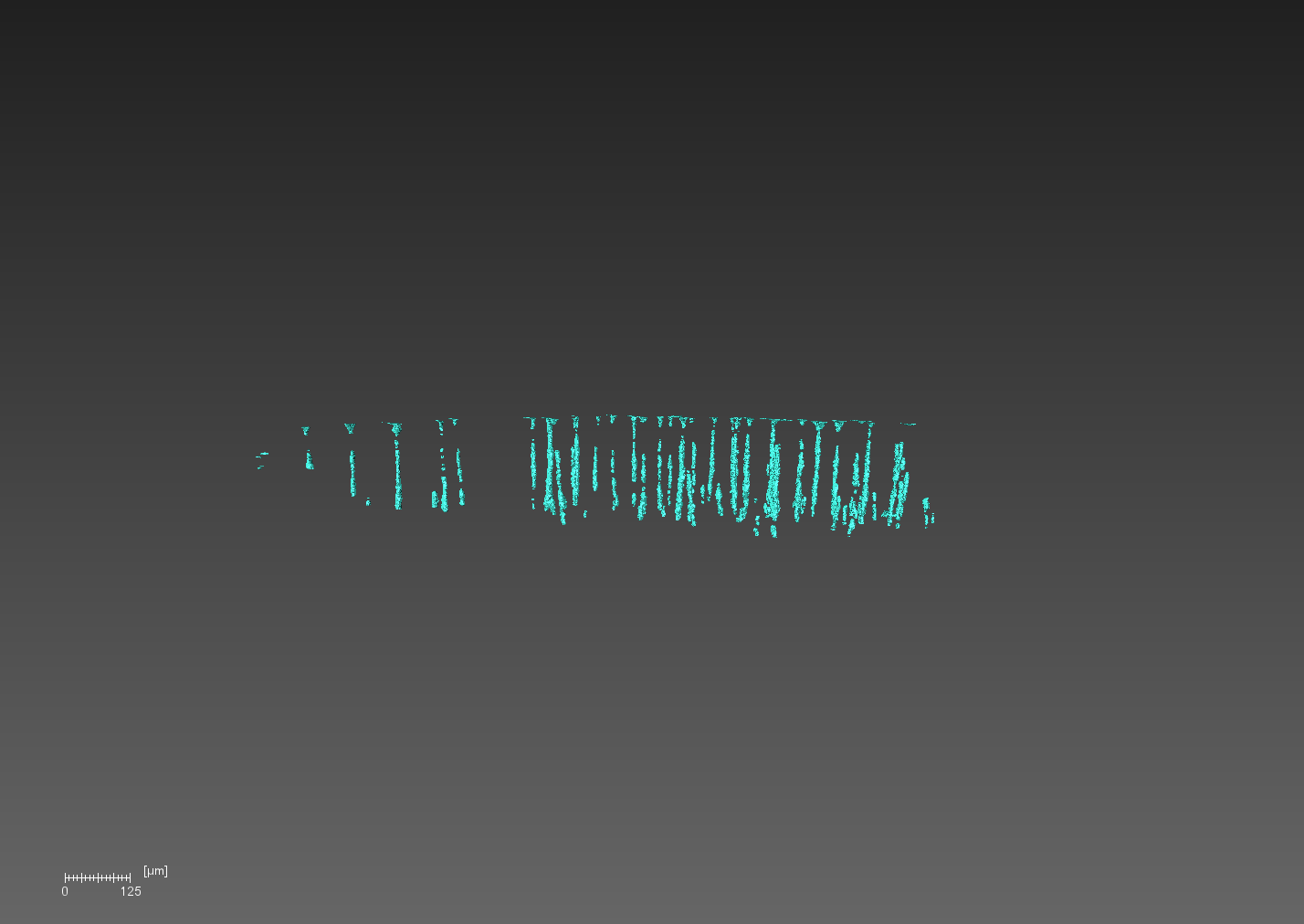

Supplement: Supplementary file 12 — Supplementary Data 12 [file 41467_2023_36405_MOESM12_ESM.zip › Micro_CT_raw_data/Northern_Aepyornis/AD1293/Results/Pore structure3.tif]

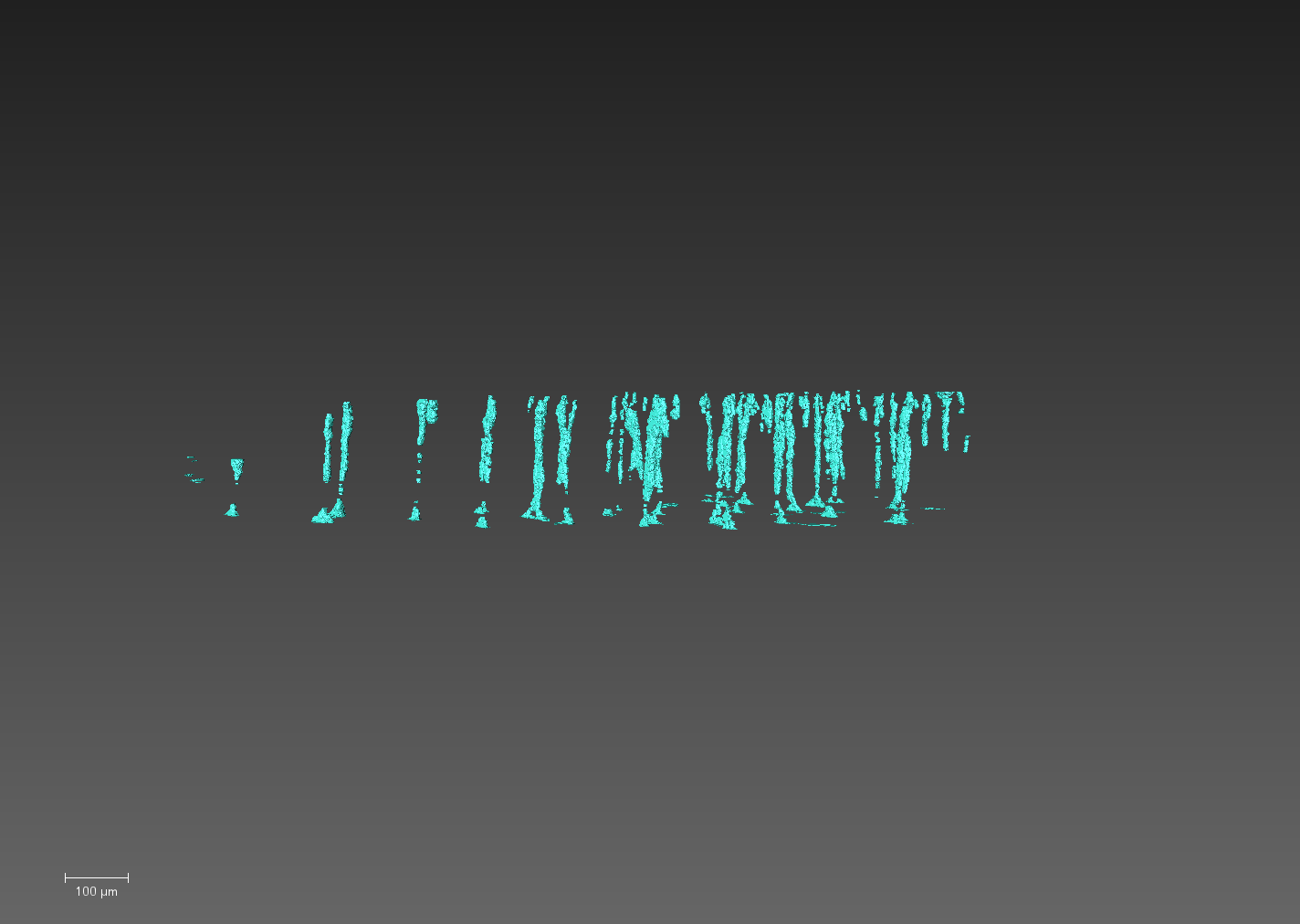

Supplement: Supplementary file 12 — Supplementary Data 12 [file 41467_2023_36405_MOESM12_ESM.zip › Micro_CT_raw_data/Northern_Aepyornis/AD1293/Results/Pore structure2.tif]

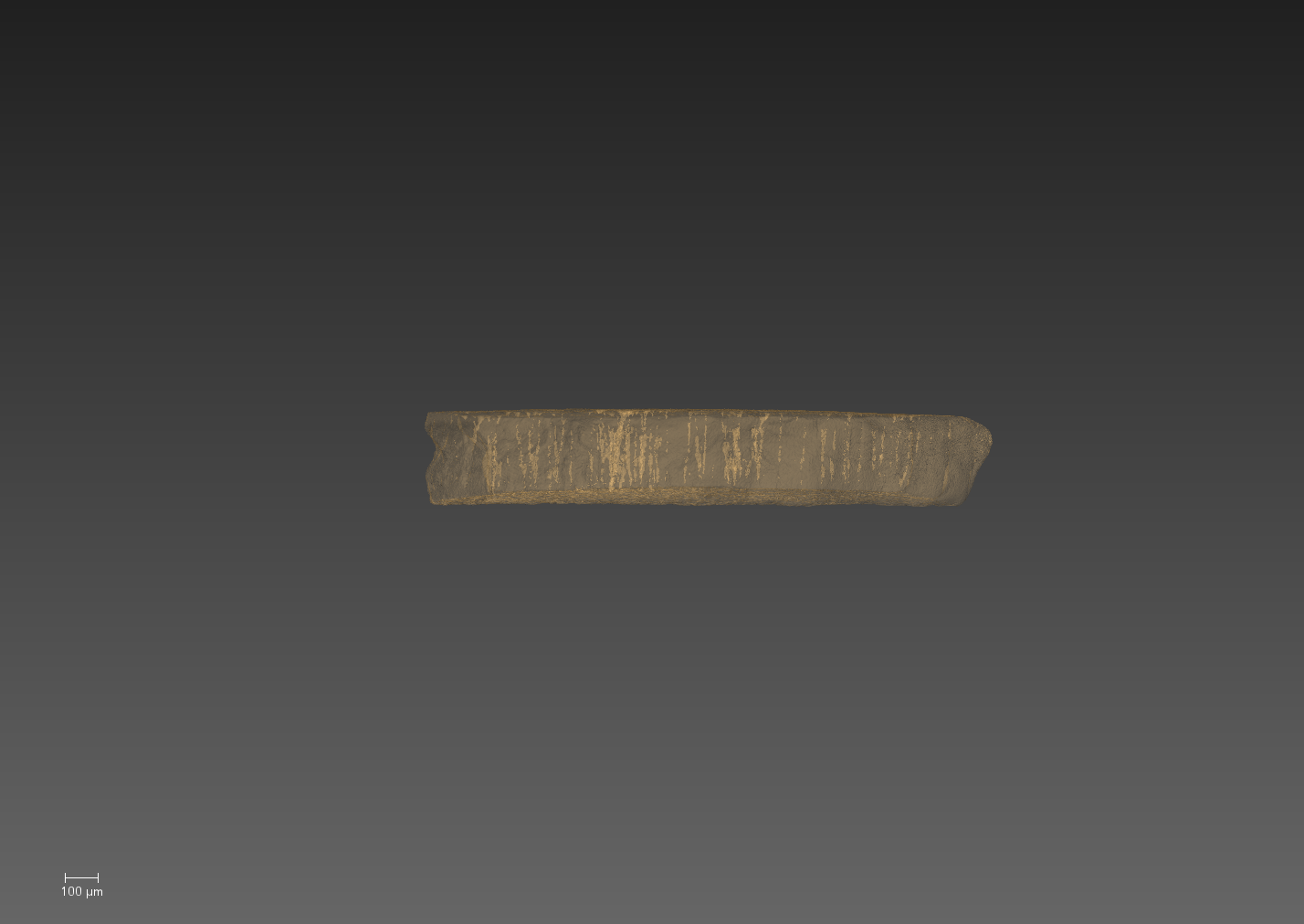

Supplement: Supplementary file 12 — Supplementary Data 12 [file 41467_2023_36405_MOESM12_ESM.zip › Micro_CT_raw_data/Northern_Aepyornis/AD1295/Results/snapshot2.tif]

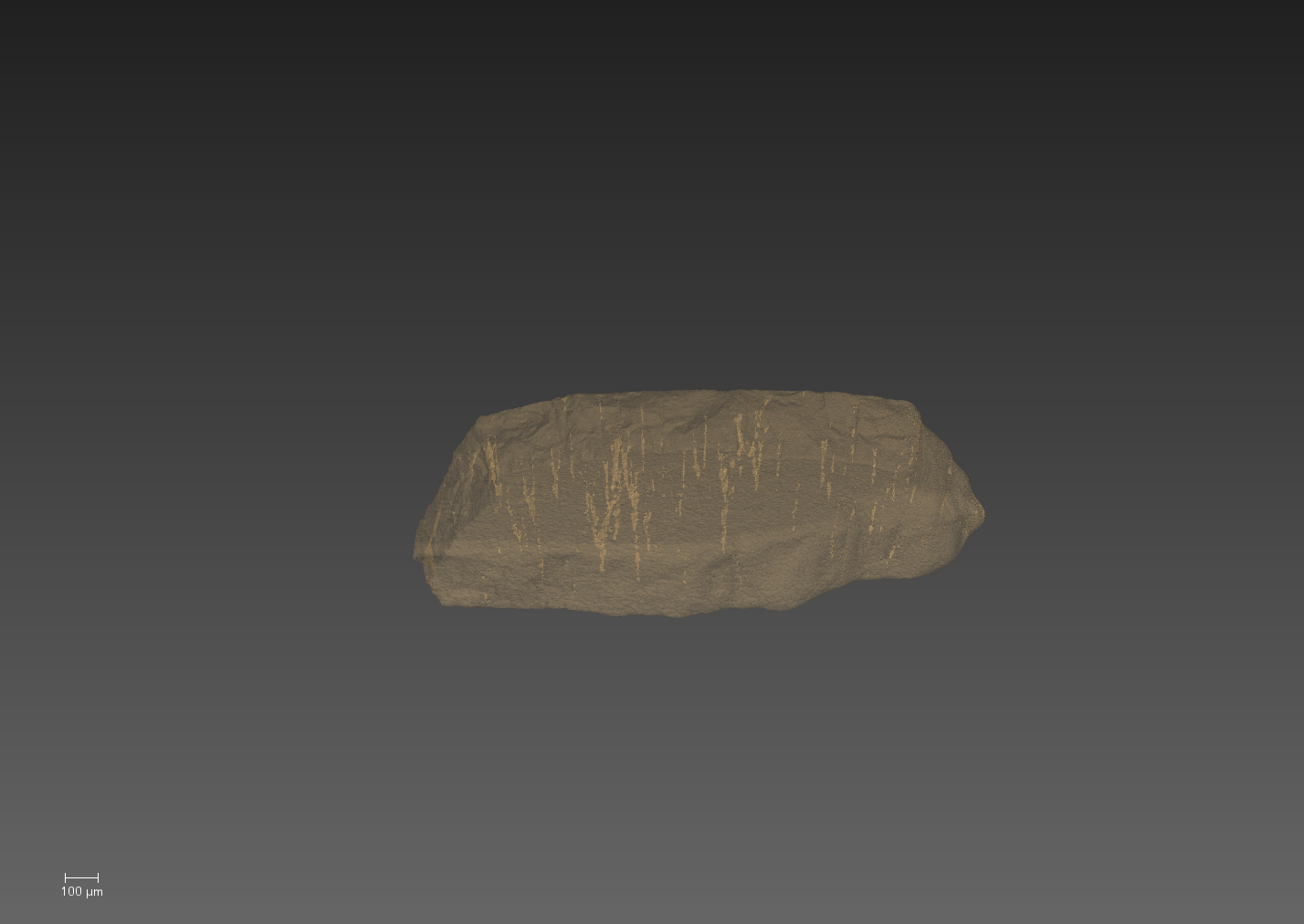

Supplement: Supplementary file 12 — Supplementary Data 12 [file 41467_2023_36405_MOESM12_ESM.zip › Micro_CT_raw_data/Northern_Aepyornis/AD1295/Results/snapshot1.tif]

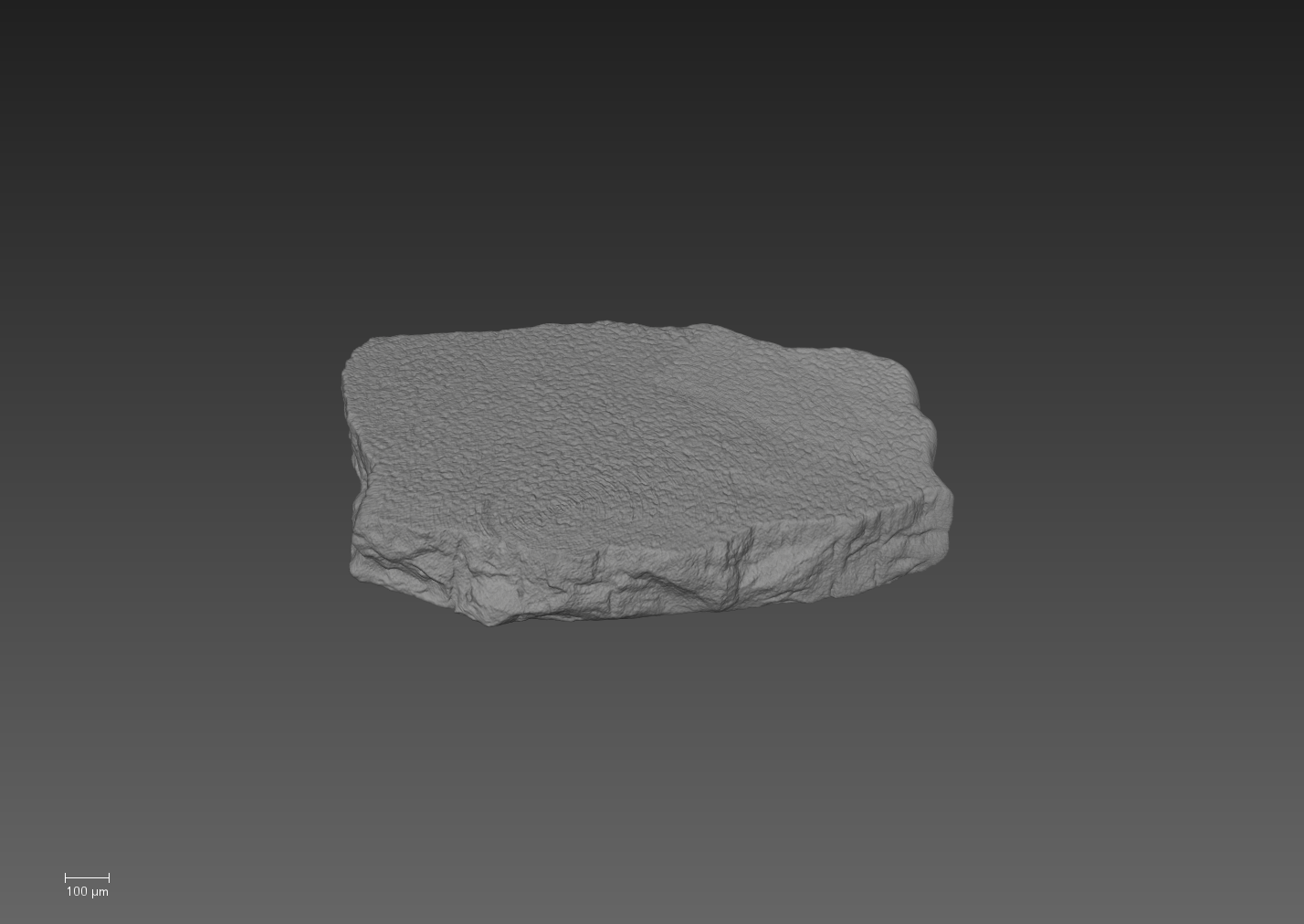

Supplement: Supplementary file 12 — Supplementary Data 12 [file 41467_2023_36405_MOESM12_ESM.zip › Micro_CT_raw_data/Northern_Aepyornis/AD1295/Results/Inner surface.tif]

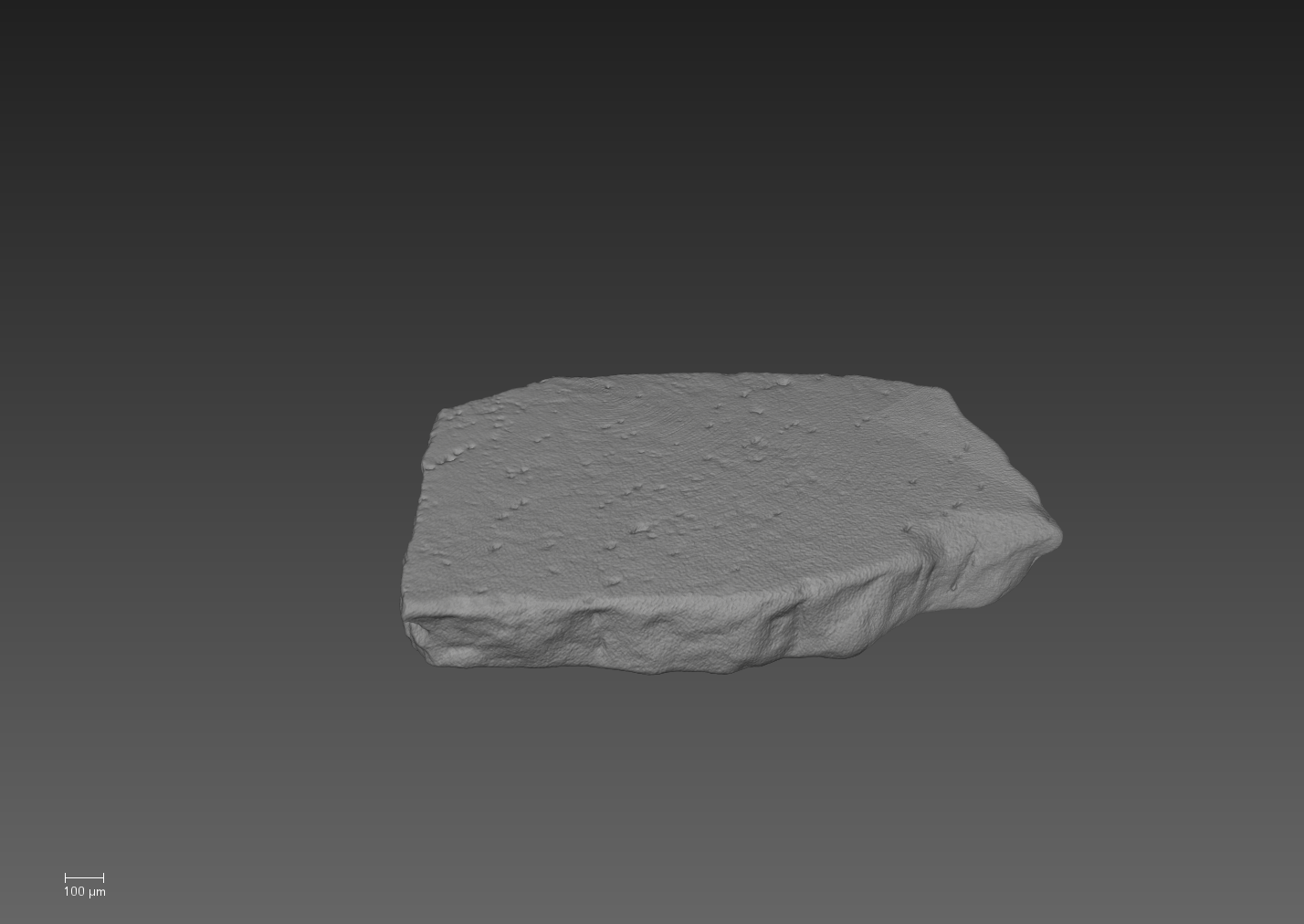

Supplement: Supplementary file 12 — Supplementary Data 12 [file 41467_2023_36405_MOESM12_ESM.zip › Micro_CT_raw_data/Northern_Aepyornis/AD1295/Results/Outer surface.tif]

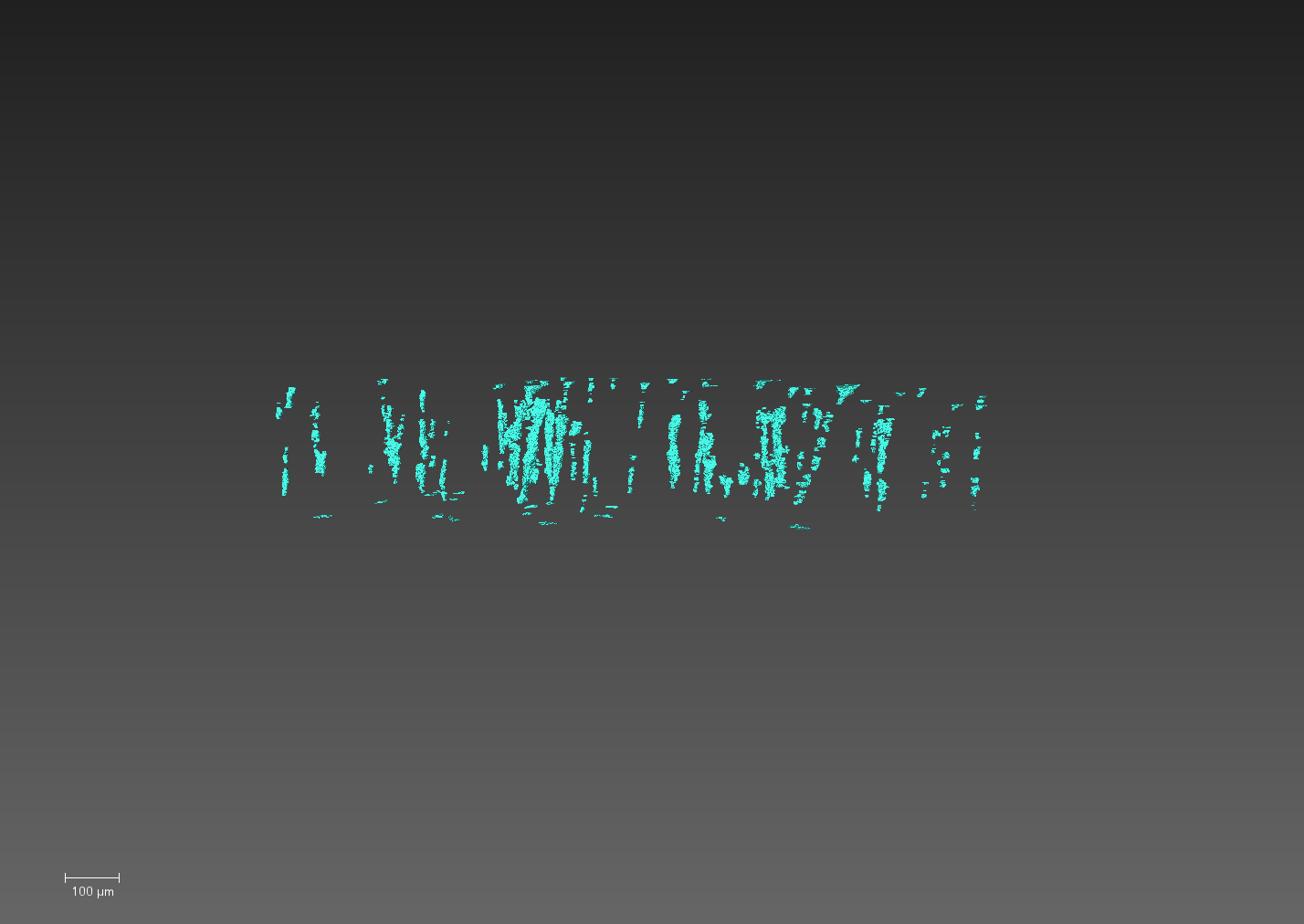

Supplement: Supplementary file 12 — Supplementary Data 12 [file 41467_2023_36405_MOESM12_ESM.zip › Micro_CT_raw_data/Northern_Aepyornis/AD1295/Results/Pore structure.tif]

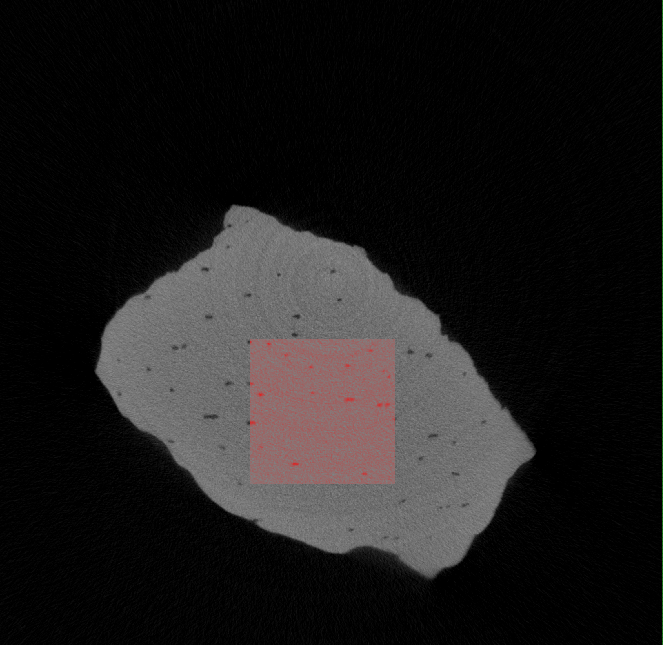

Supplement: Supplementary file 12 — Supplementary Data 12 [file 41467_2023_36405_MOESM12_ESM.zip › Micro_CT_raw_data/Northern_Aepyornis/AD1295/Results/ROI selection.tif]

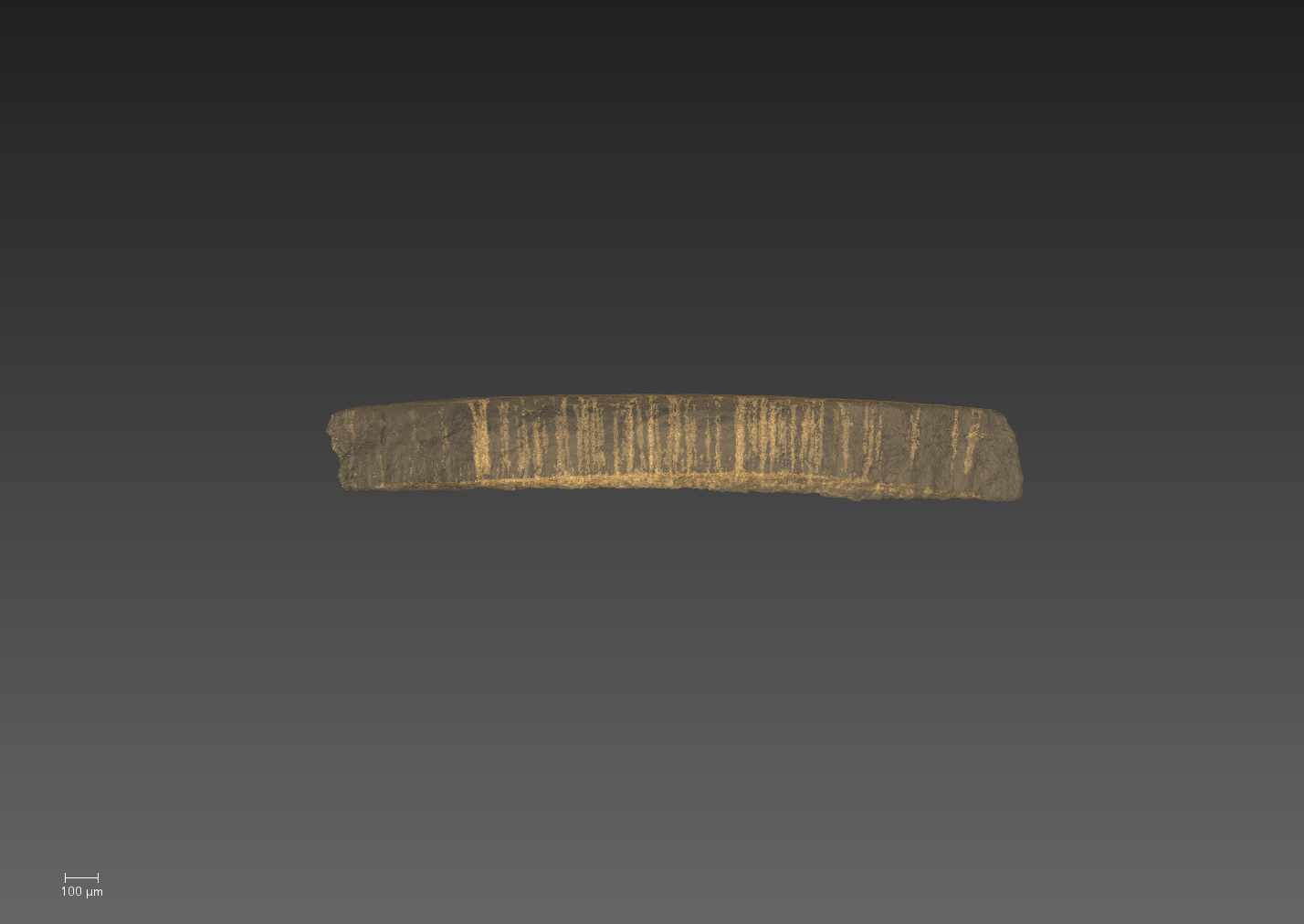

Supplement: Supplementary file 12 — Supplementary Data 12 [file 41467_2023_36405_MOESM12_ESM.zip › Micro_CT_raw_data/Northern_Aepyornis/AD1292/Results/snapshot2.tif]

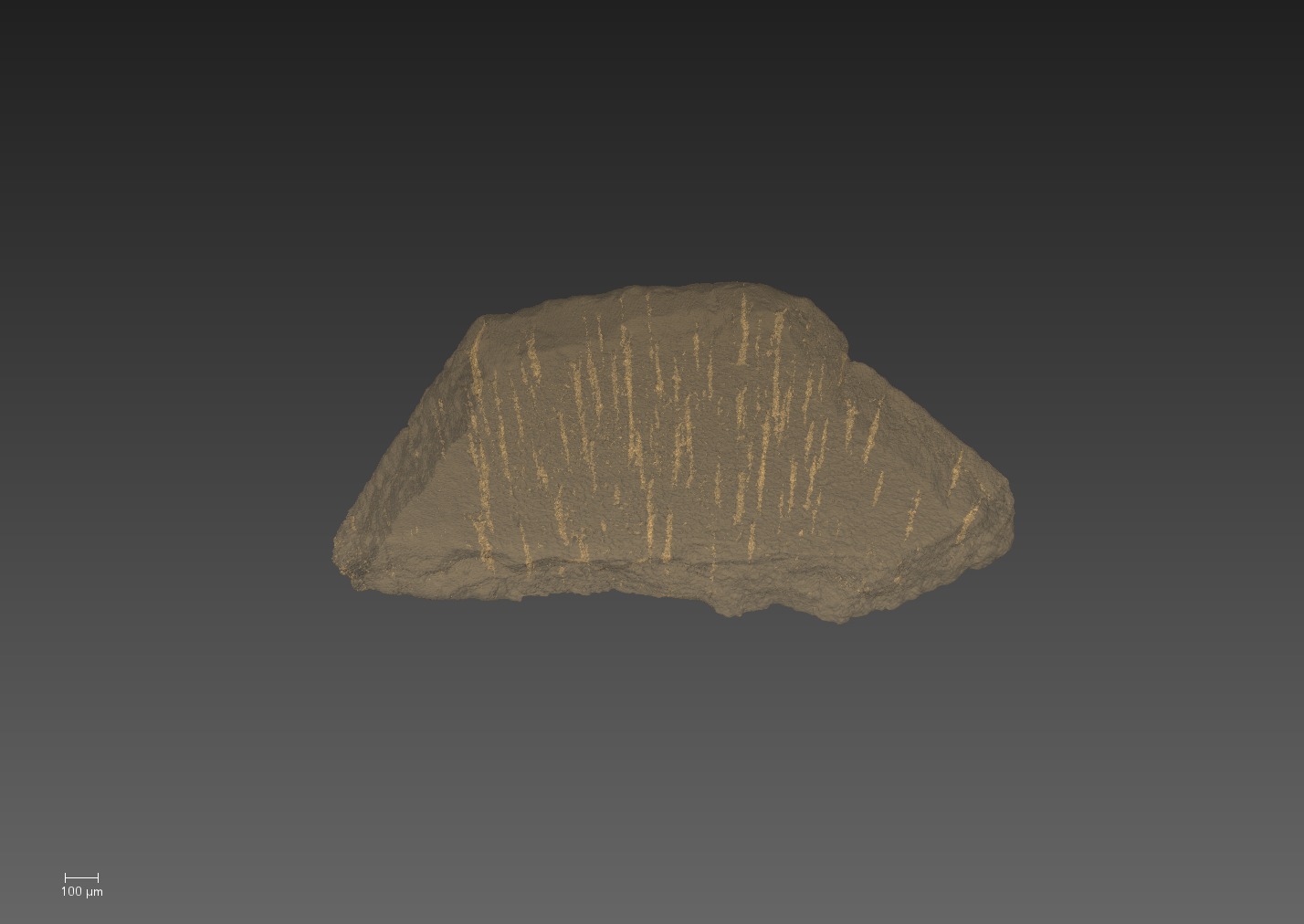

Supplement: Supplementary file 12 — Supplementary Data 12 [file 41467_2023_36405_MOESM12_ESM.zip › Micro_CT_raw_data/Northern_Aepyornis/AD1292/Results/snapshot1.tif]

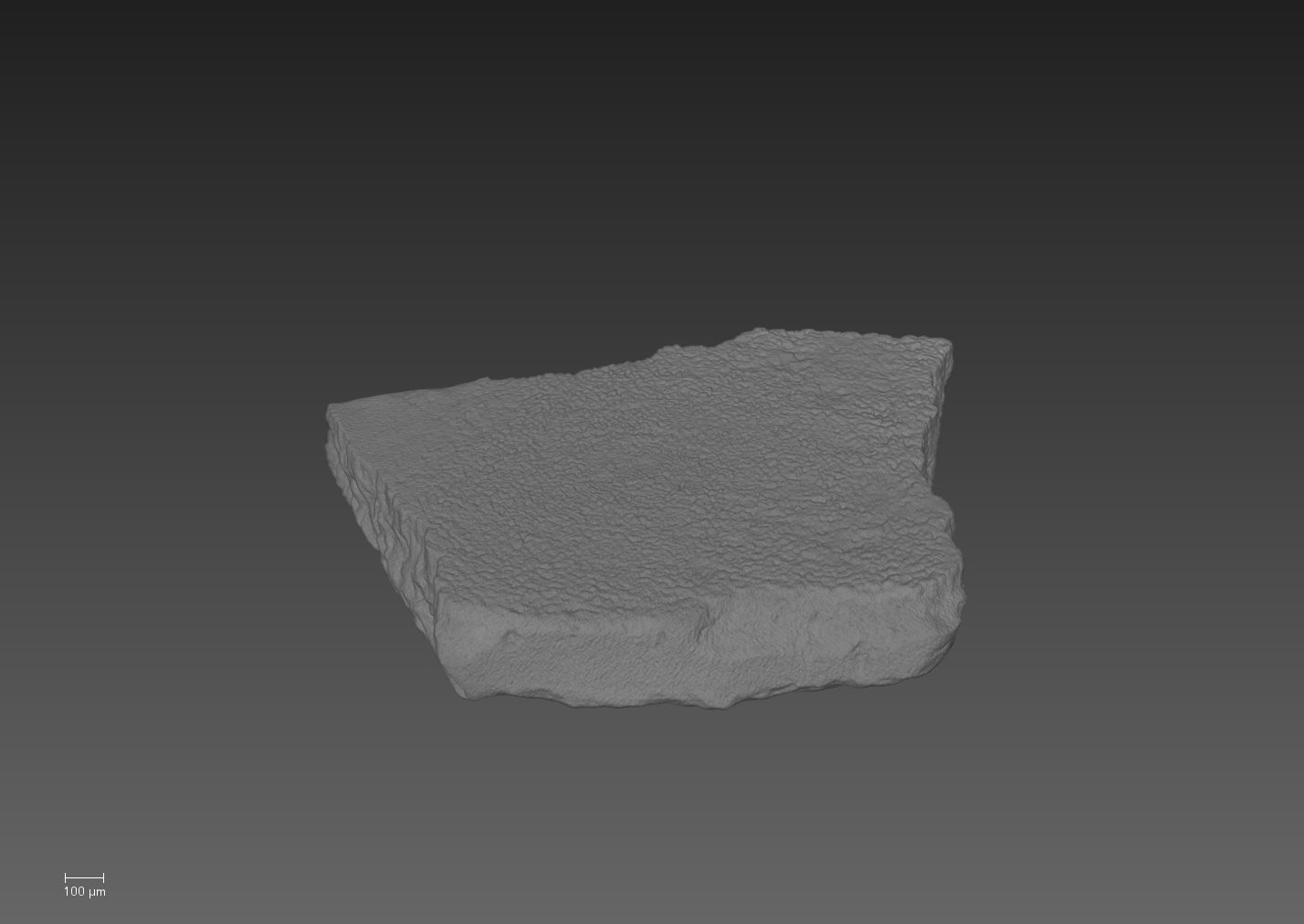

Supplement: Supplementary file 12 — Supplementary Data 12 [file 41467_2023_36405_MOESM12_ESM.zip › Micro_CT_raw_data/Northern_Aepyornis/AD1292/Results/Inner surface.tif]

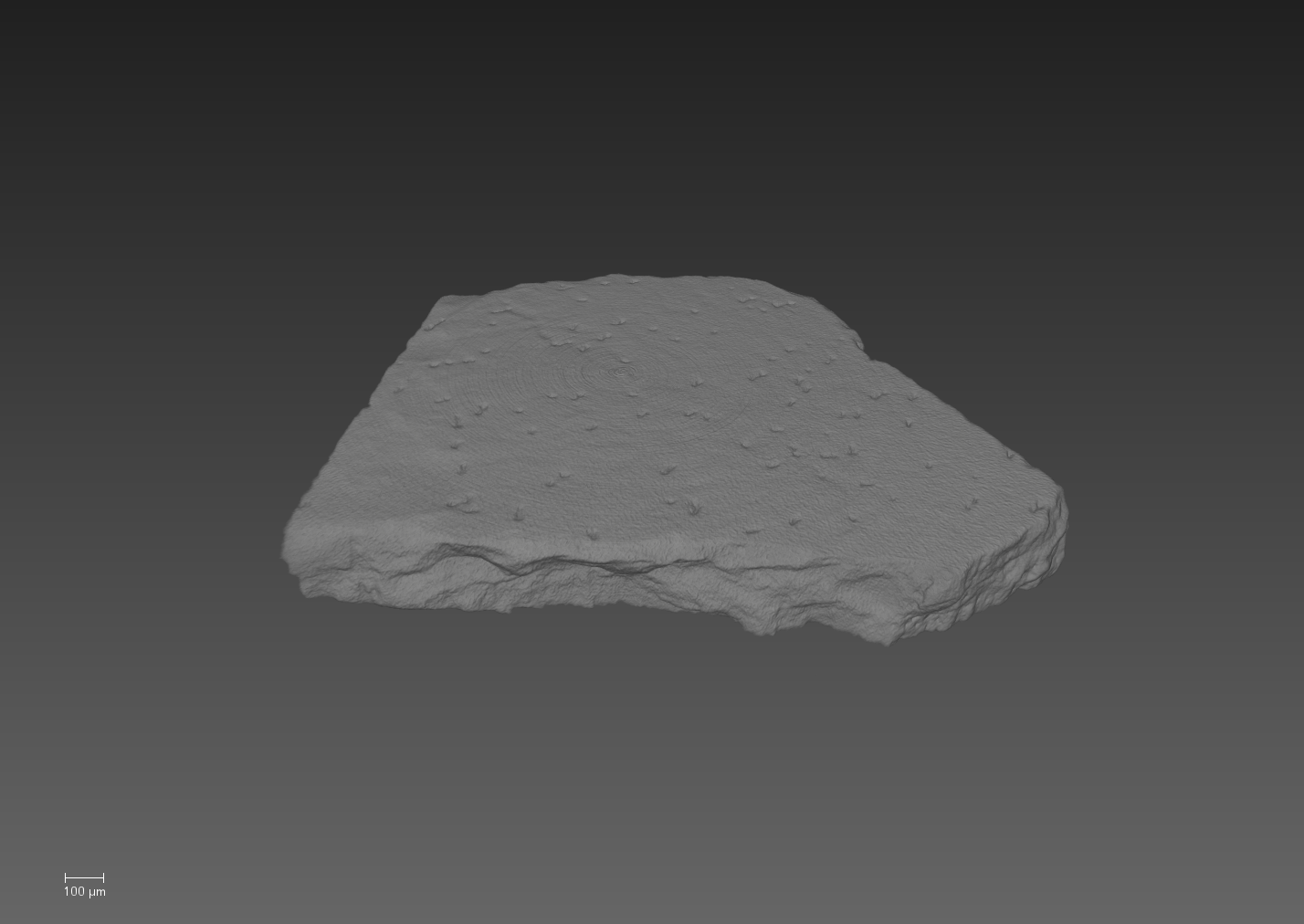

Supplement: Supplementary file 12 — Supplementary Data 12 [file 41467_2023_36405_MOESM12_ESM.zip › Micro_CT_raw_data/Northern_Aepyornis/AD1292/Results/Outer surface.tif]

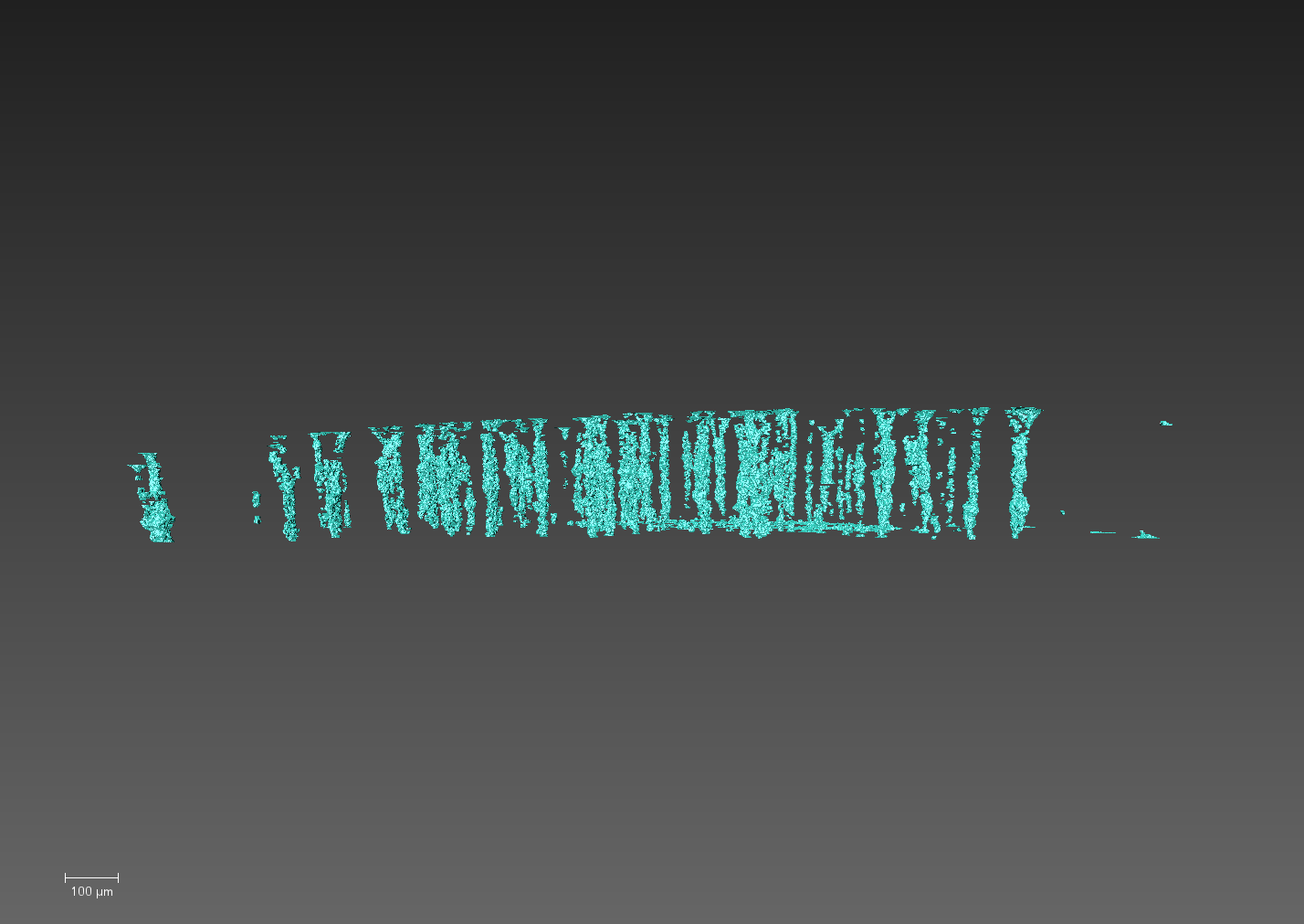

Supplement: Supplementary file 12 — Supplementary Data 12 [file 41467_2023_36405_MOESM12_ESM.zip › Micro_CT_raw_data/Northern_Aepyornis/AD1292/Results/Pore structure.tif]

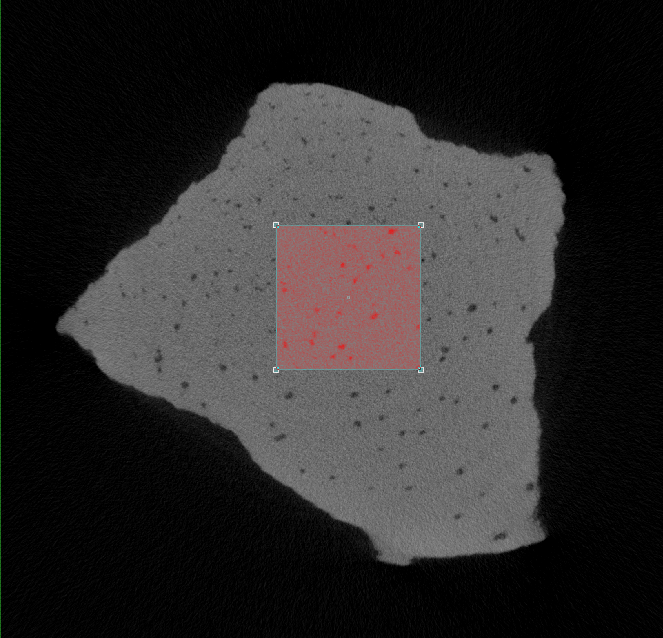

Supplement: Supplementary file 12 — Supplementary Data 12 [file 41467_2023_36405_MOESM12_ESM.zip › Micro_CT_raw_data/Northern_Aepyornis/AD1292/Results/ROI Selection.tif]

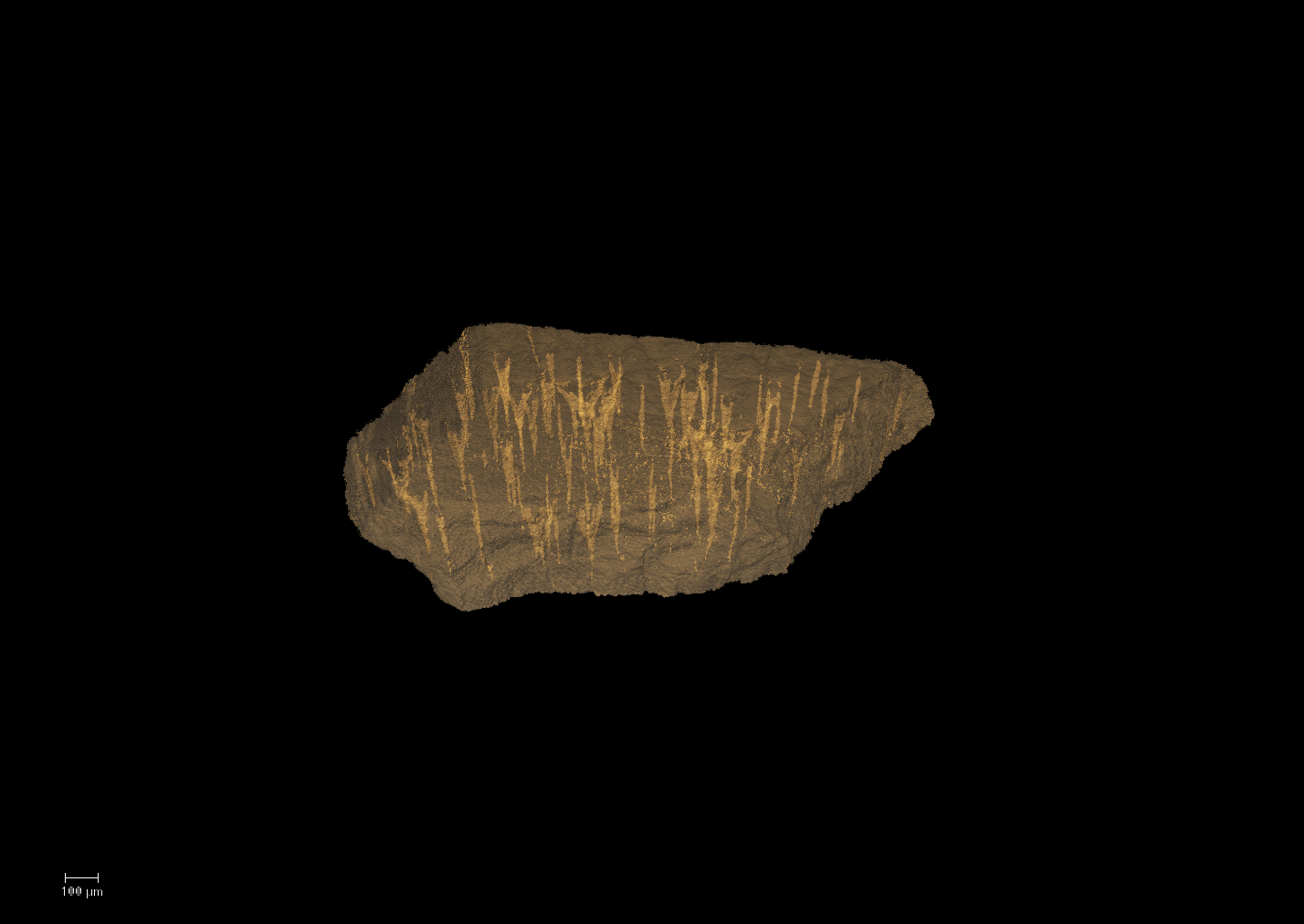

Supplement: Supplementary file 12 — Supplementary Data 12 [file 41467_2023_36405_MOESM12_ESM.zip › Micro_CT_raw_data/Northern_Aepyornis/AD2139/Results/snapshot3.tif]

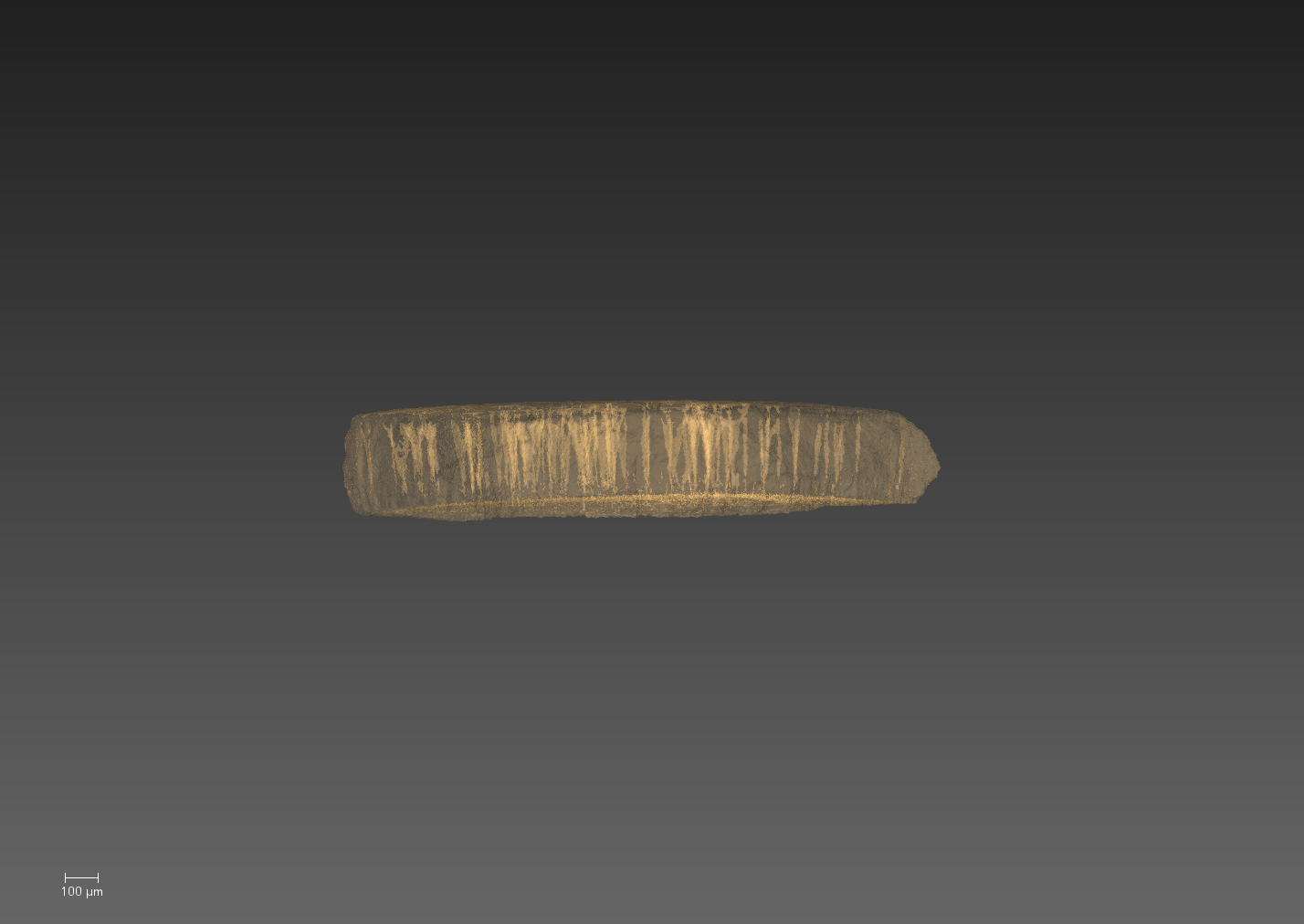

Supplement: Supplementary file 12 — Supplementary Data 12 [file 41467_2023_36405_MOESM12_ESM.zip › Micro_CT_raw_data/Northern_Aepyornis/AD2139/Results/snapshot2.tif]

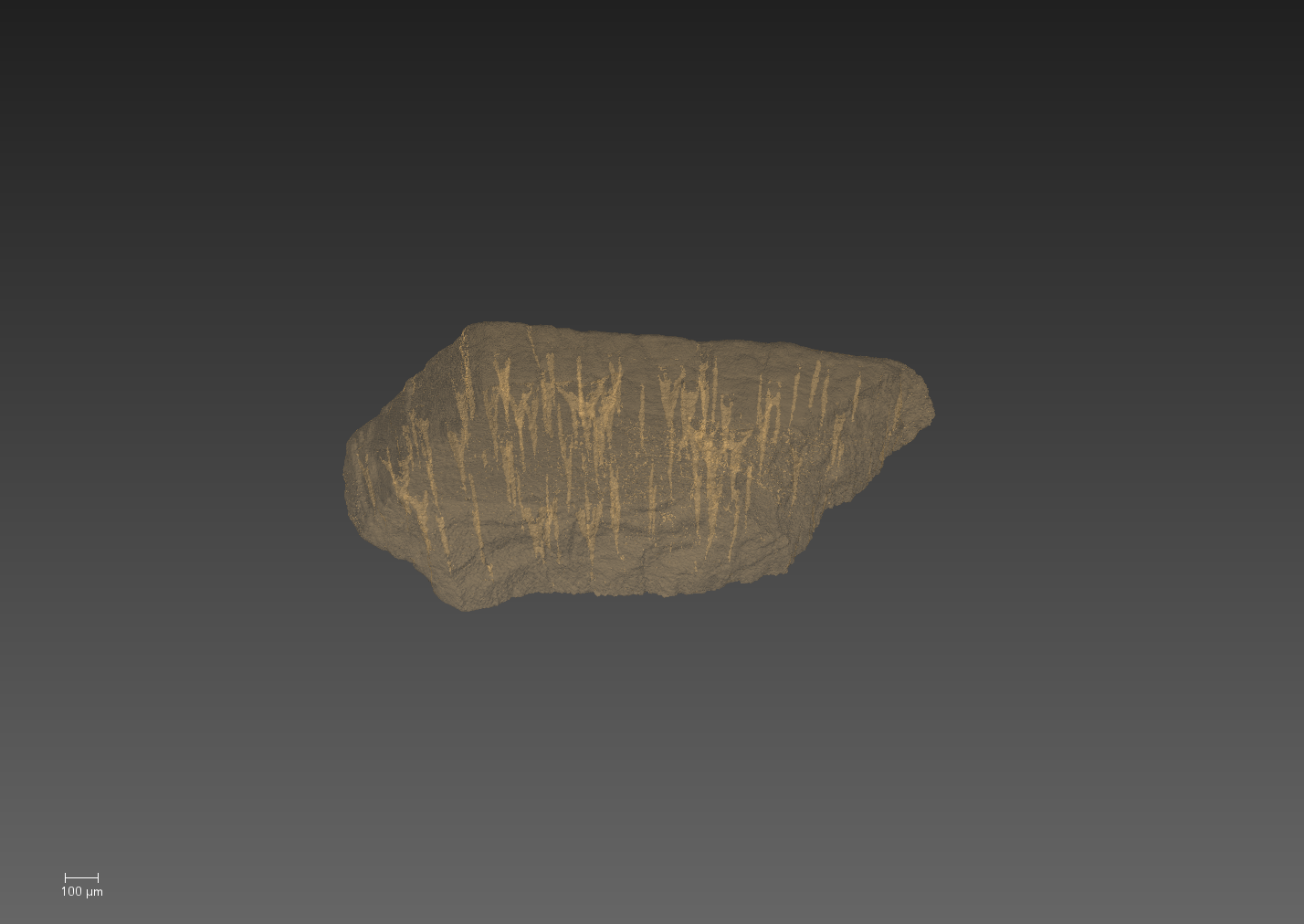

Supplement: Supplementary file 12 — Supplementary Data 12 [file 41467_2023_36405_MOESM12_ESM.zip › Micro_CT_raw_data/Northern_Aepyornis/AD2139/Results/snapshot1.tif]

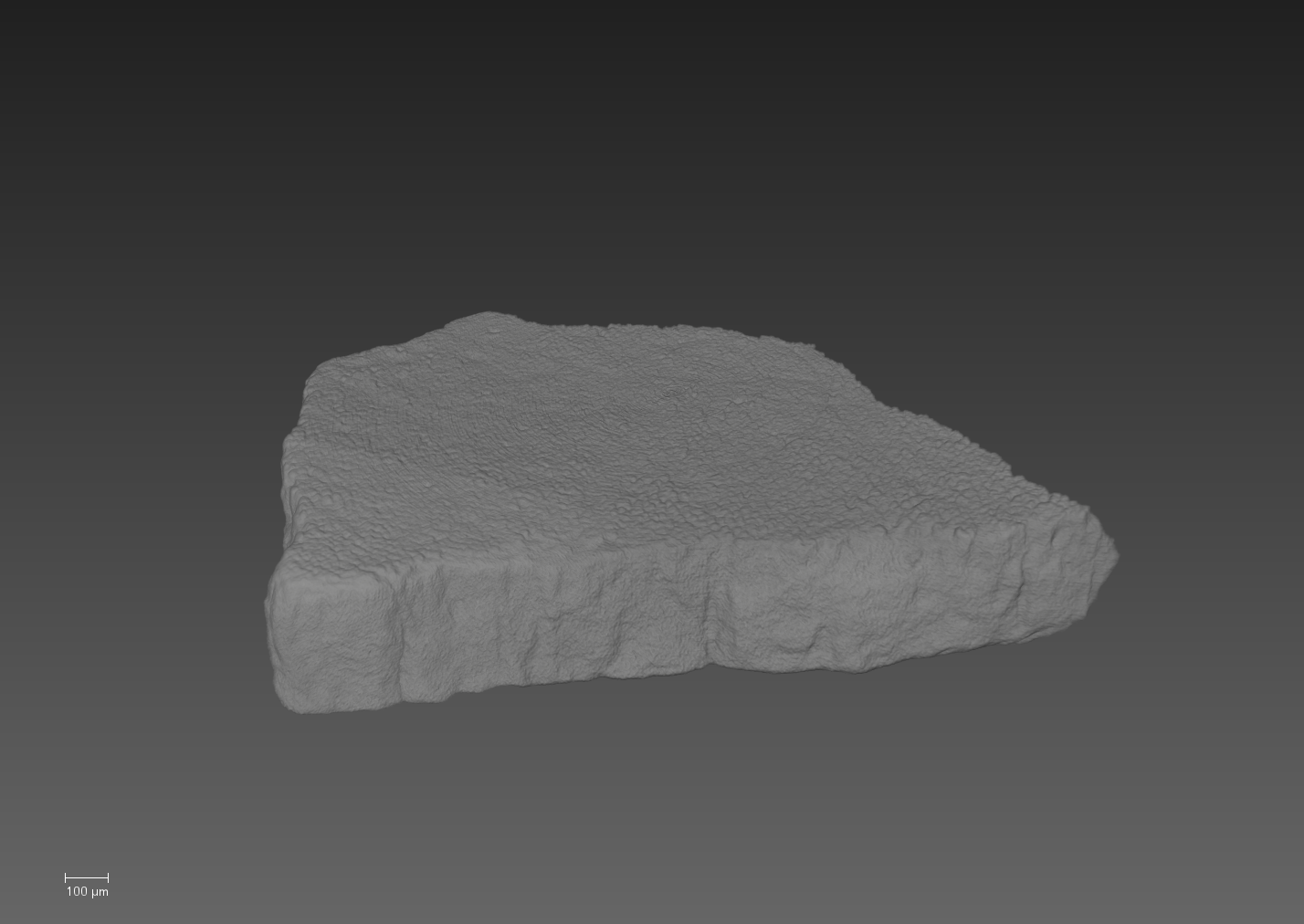

Supplement: Supplementary file 12 — Supplementary Data 12 [file 41467_2023_36405_MOESM12_ESM.zip › Micro_CT_raw_data/Northern_Aepyornis/AD2139/Results/Inner surface.tif]

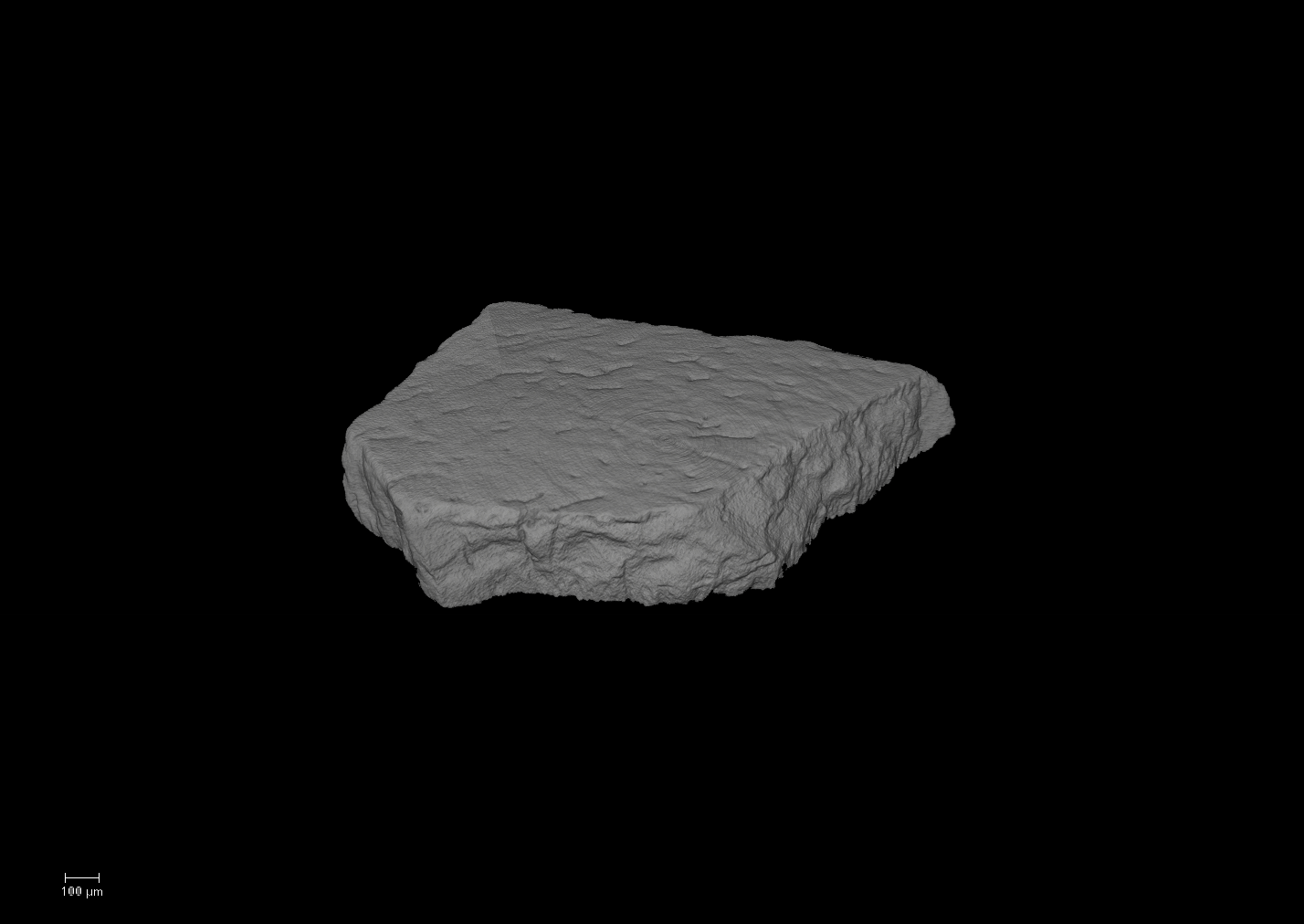

Supplement: Supplementary file 12 — Supplementary Data 12 [file 41467_2023_36405_MOESM12_ESM.zip › Micro_CT_raw_data/Northern_Aepyornis/AD2139/Results/Outer surface.tif]

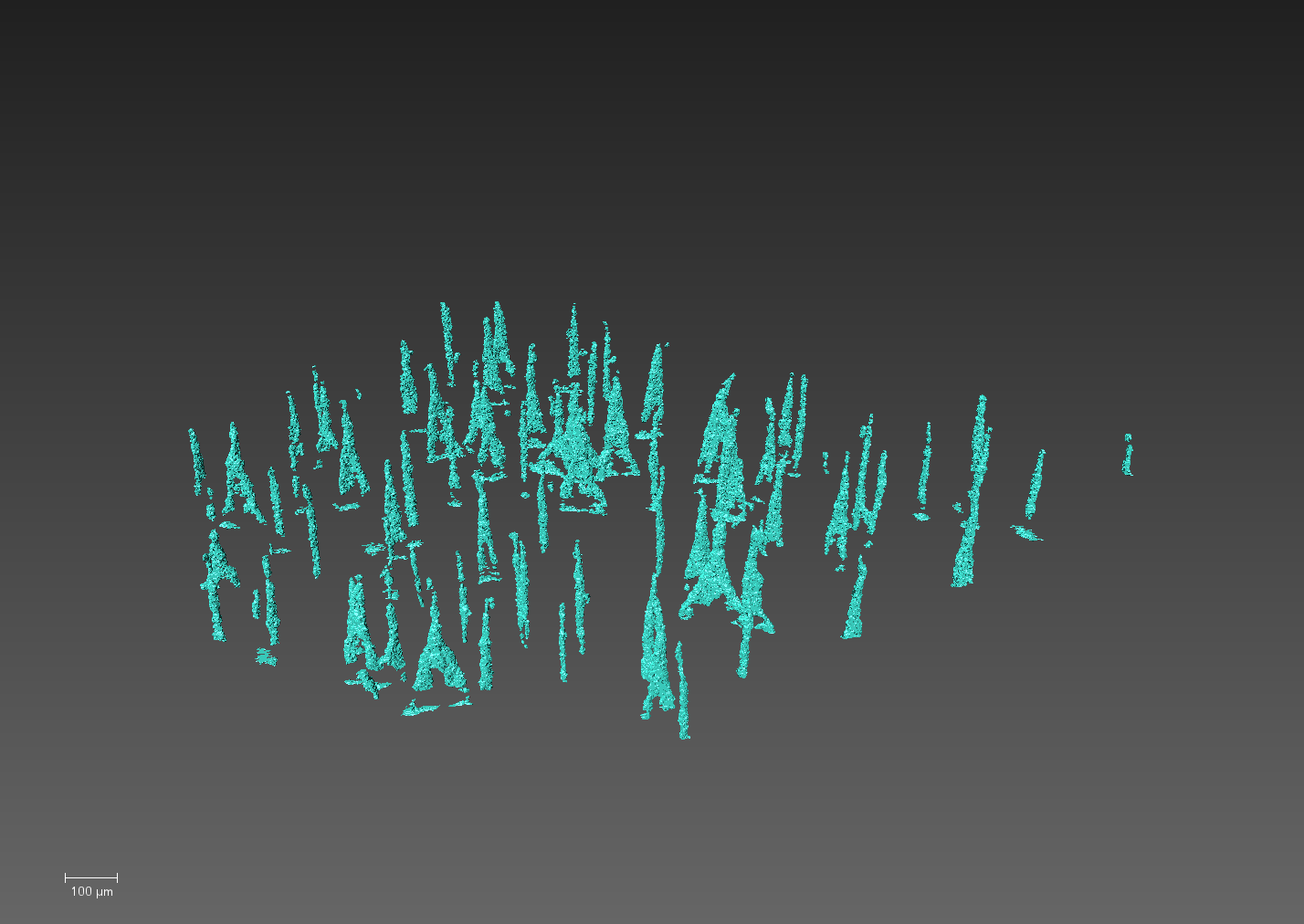

Supplement: Supplementary file 12 — Supplementary Data 12 [file 41467_2023_36405_MOESM12_ESM.zip › Micro_CT_raw_data/Northern_Aepyornis/AD2139/Results/Pore structure.tif]

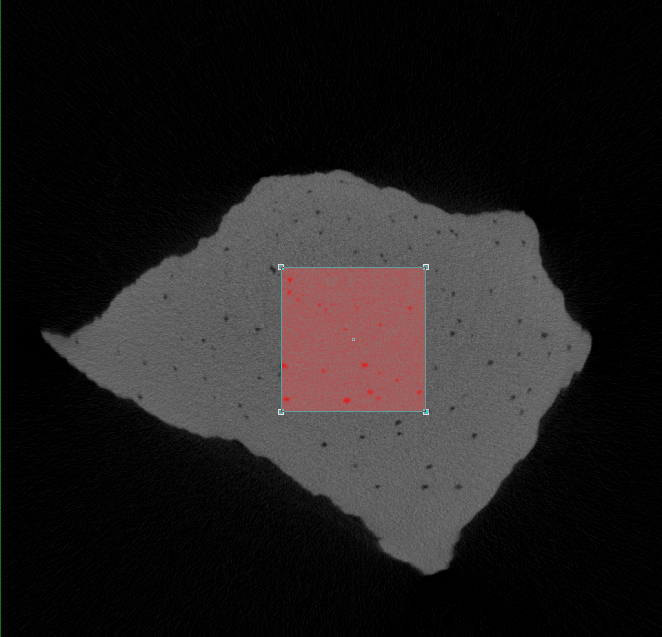

Supplement: Supplementary file 12 — Supplementary Data 12 [file 41467_2023_36405_MOESM12_ESM.zip › Micro_CT_raw_data/Northern_Aepyornis/AD2139/Results/ROI Selection.tif]

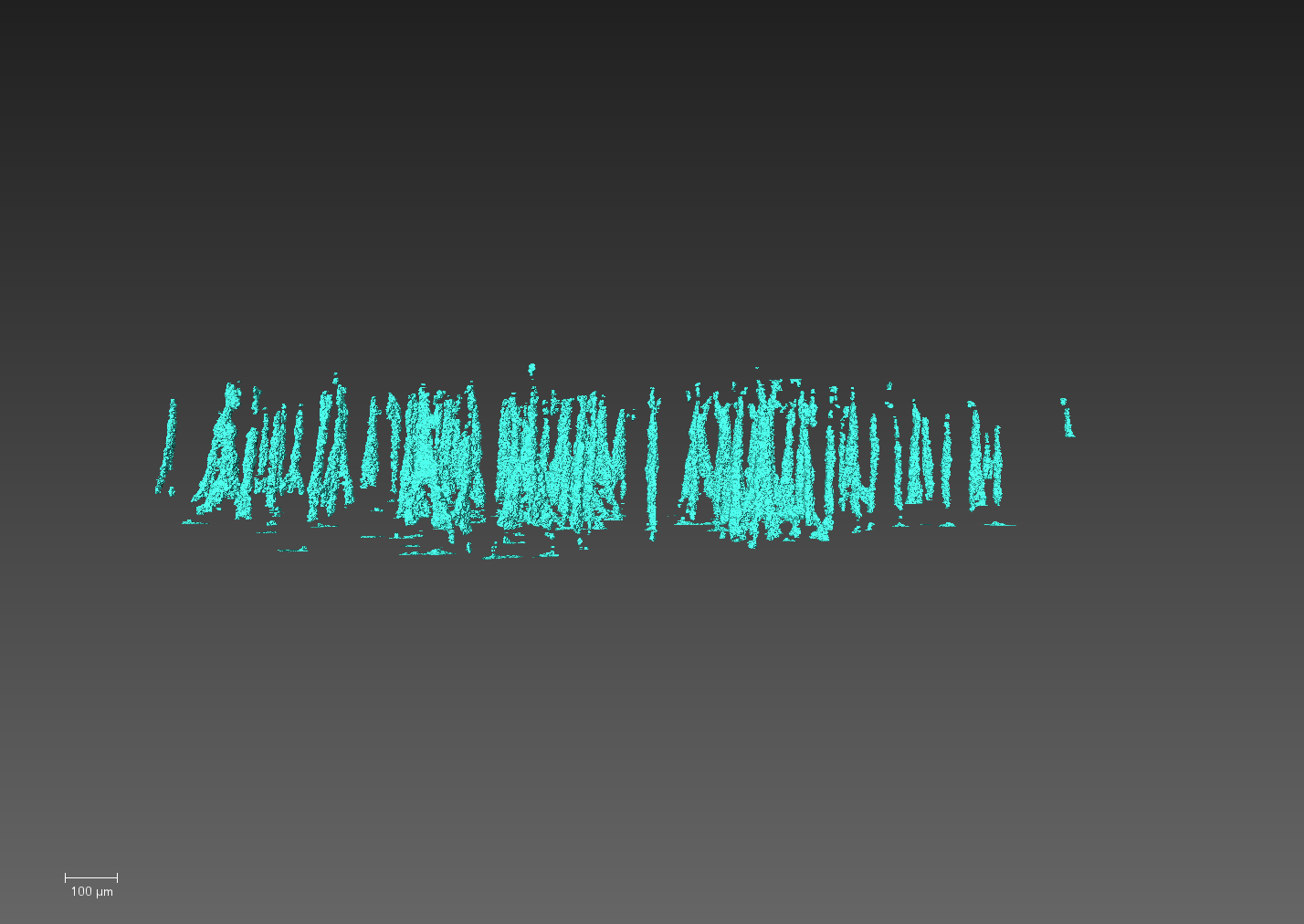

Supplement: Supplementary file 12 — Supplementary Data 12 [file 41467_2023_36405_MOESM12_ESM.zip › Micro_CT_raw_data/Northern_Aepyornis/AD2139/Results/Pore structure2.tif]

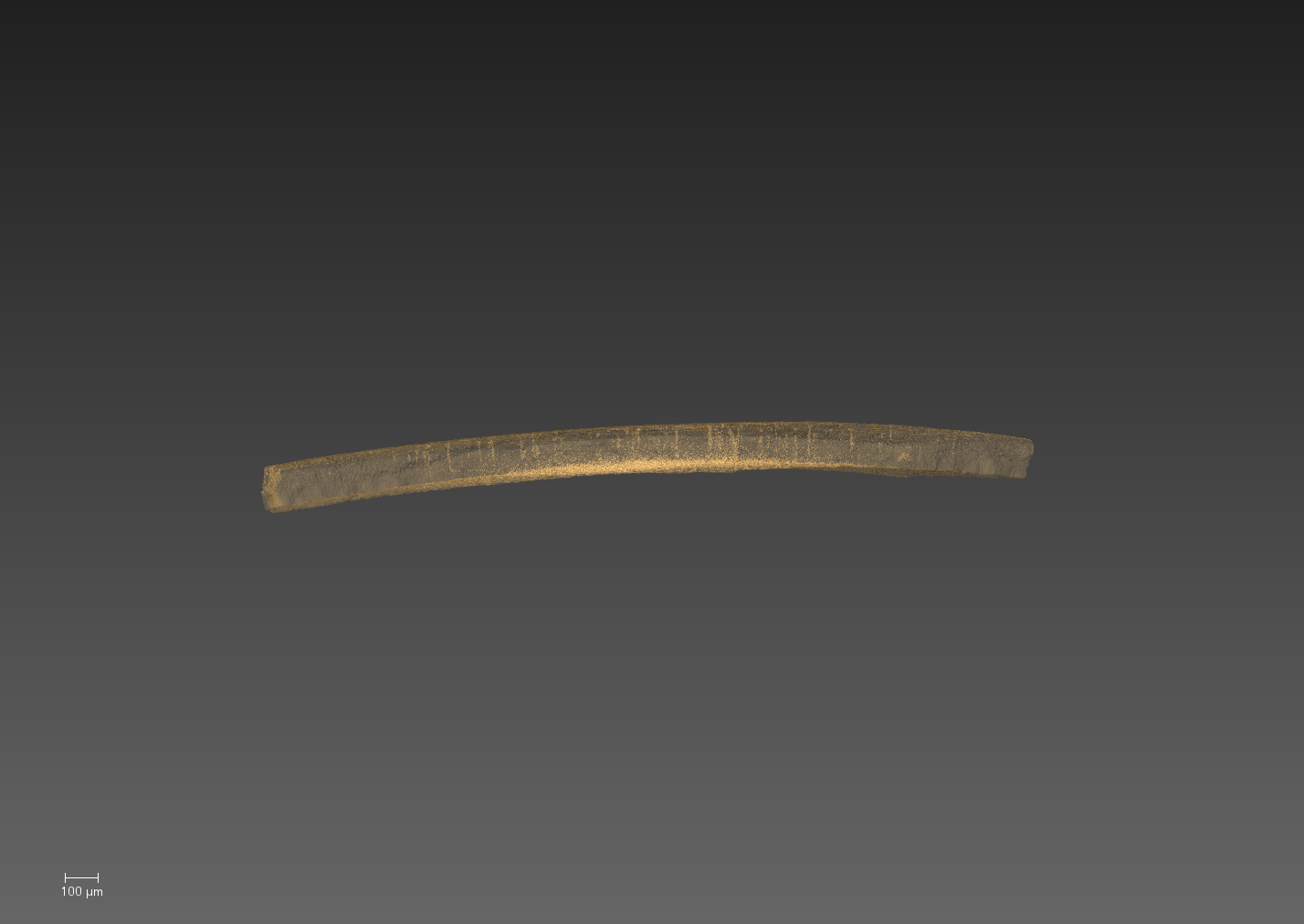

Supplement: Supplementary file 12 — Supplementary Data 12 [file 41467_2023_36405_MOESM12_ESM.zip › Micro_CT_raw_data/Mullerornis/AD2111/Results/snapshot2.tif]

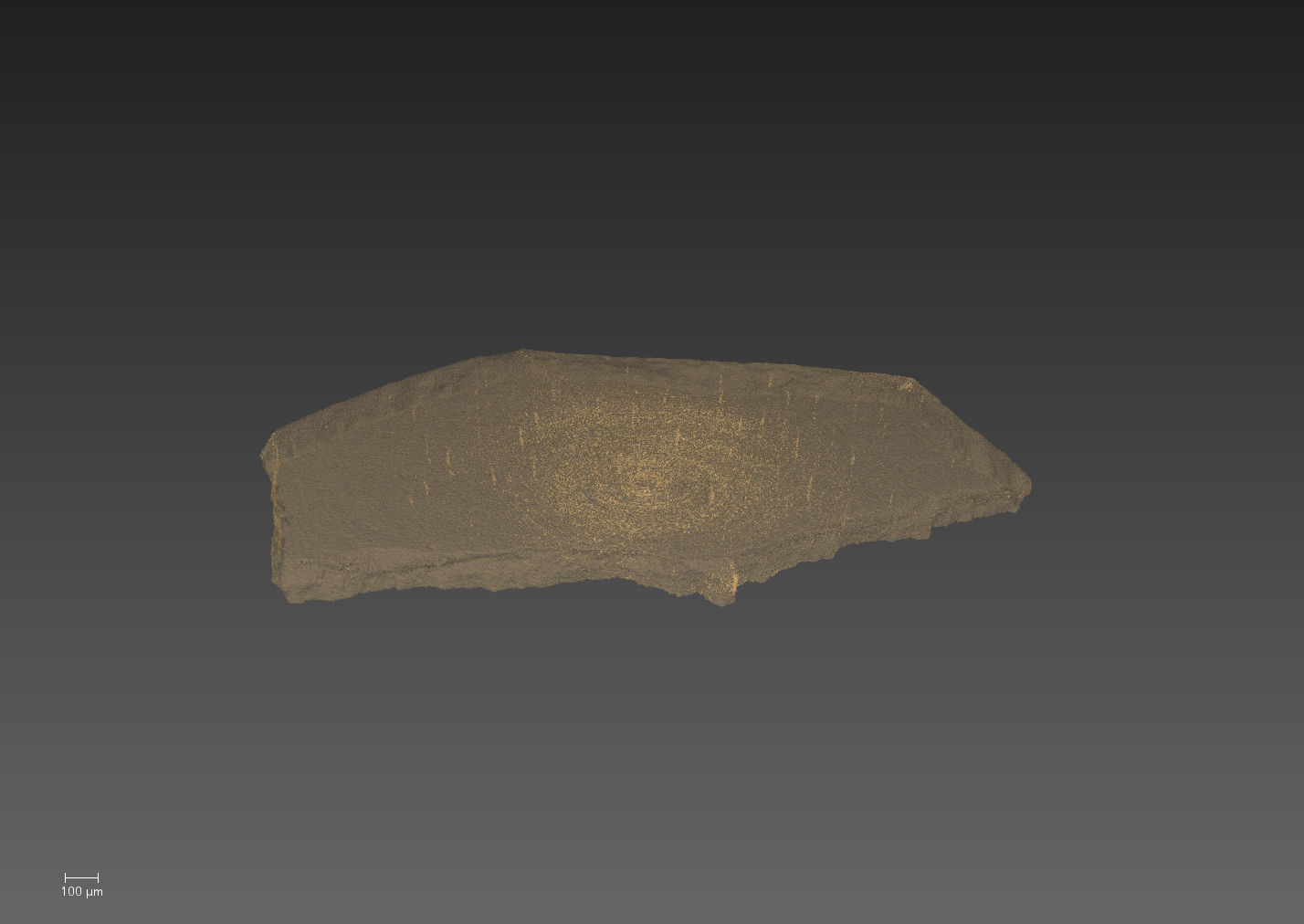

Supplement: Supplementary file 12 — Supplementary Data 12 [file 41467_2023_36405_MOESM12_ESM.zip › Micro_CT_raw_data/Mullerornis/AD2111/Results/snapshot1.tif]

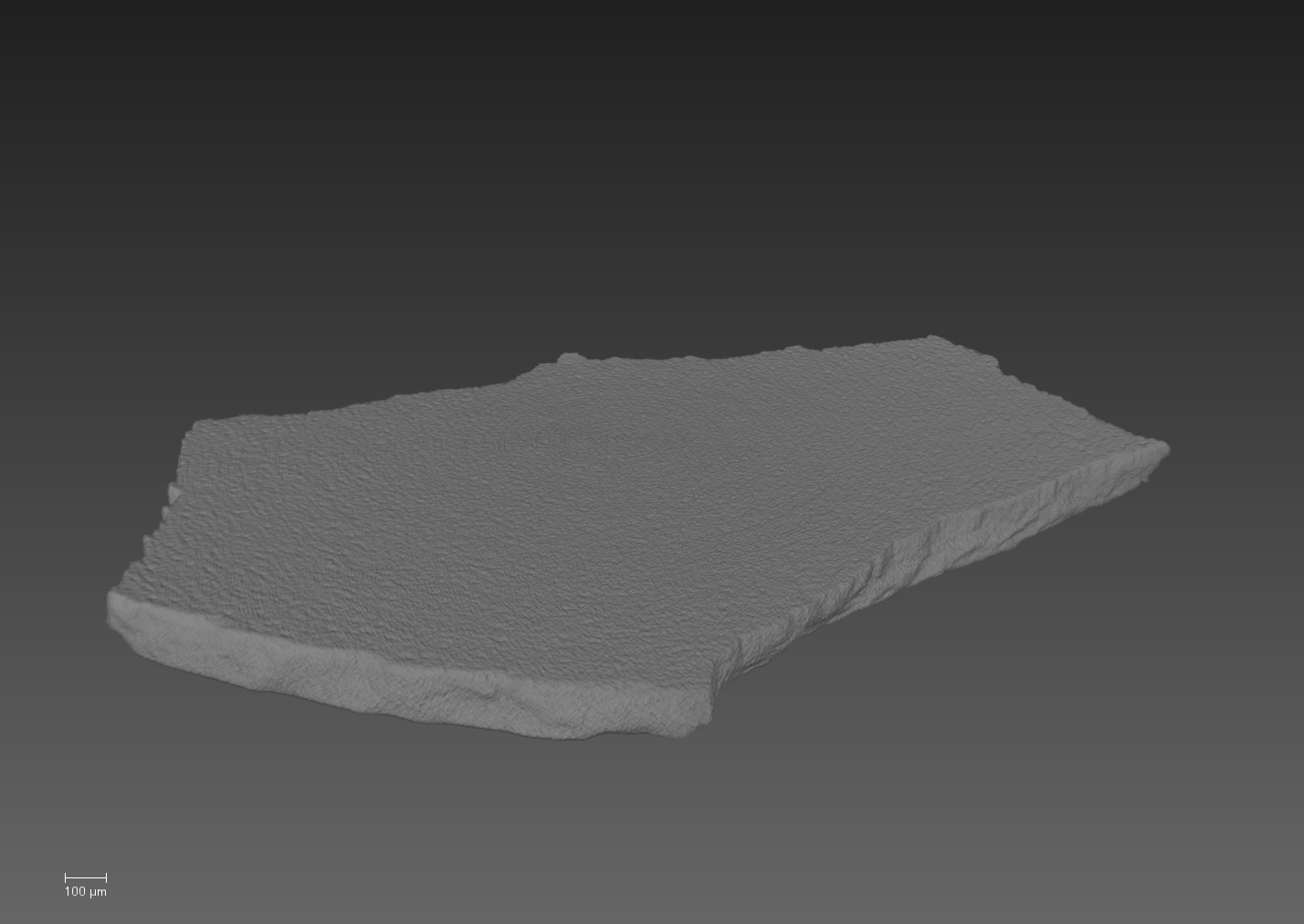

Supplement: Supplementary file 12 — Supplementary Data 12 [file 41467_2023_36405_MOESM12_ESM.zip › Micro_CT_raw_data/Mullerornis/AD2111/Results/Inner surface.tif]

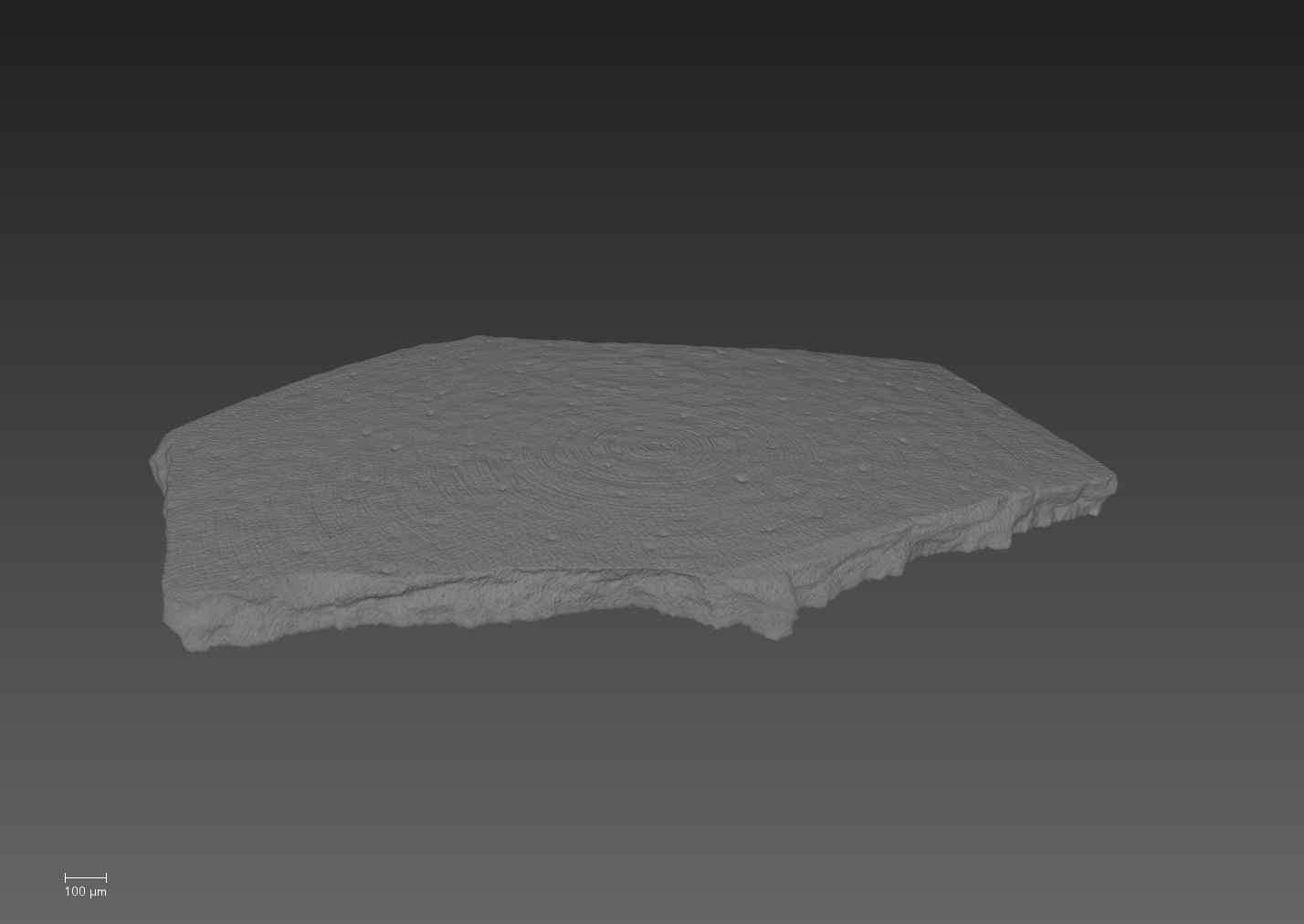

Supplement: Supplementary file 12 — Supplementary Data 12 [file 41467_2023_36405_MOESM12_ESM.zip › Micro_CT_raw_data/Mullerornis/AD2111/Results/Outer surface.tif]

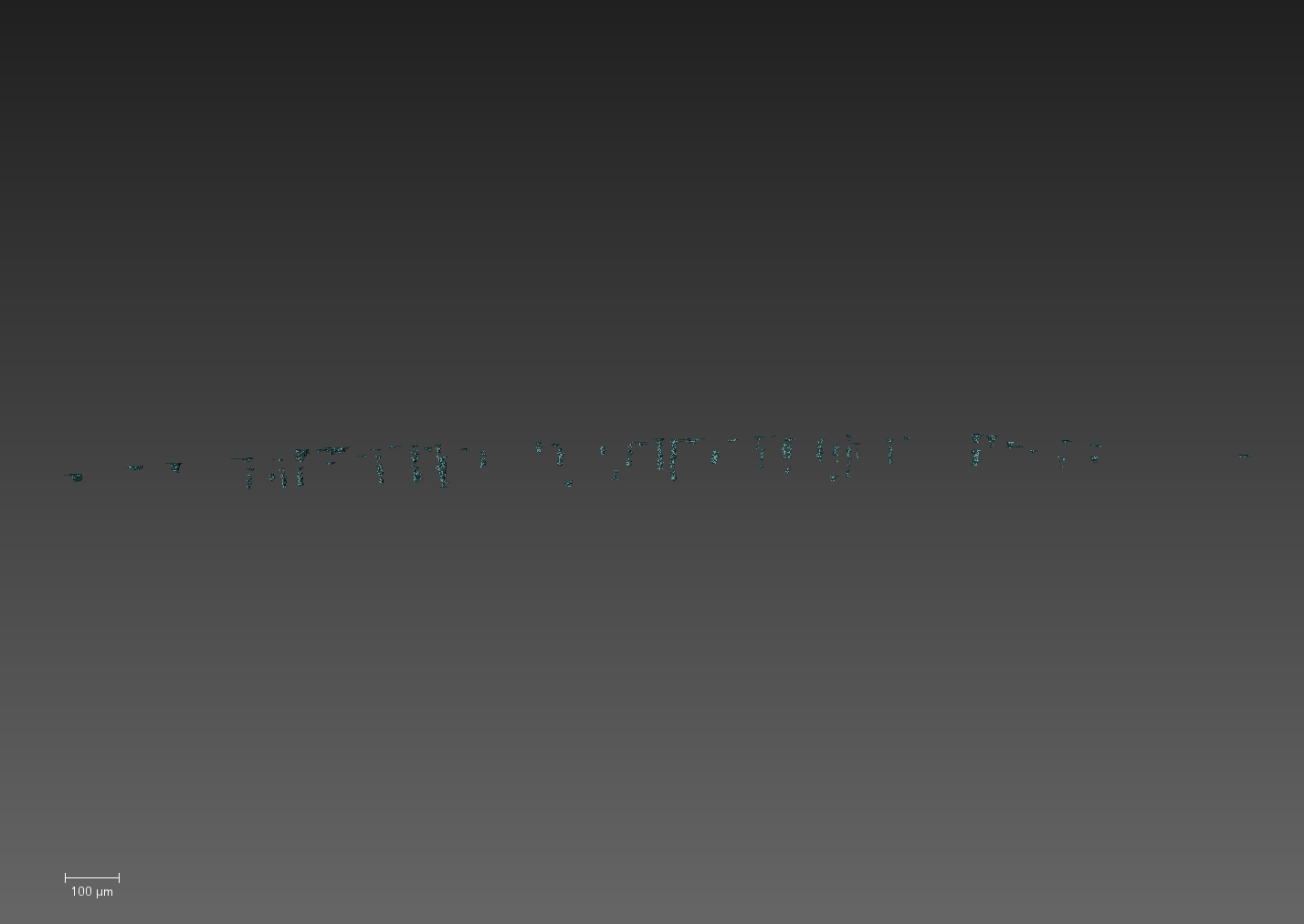

Supplement: Supplementary file 12 — Supplementary Data 12 [file 41467_2023_36405_MOESM12_ESM.zip › Micro_CT_raw_data/Mullerornis/AD2111/Results/Pore structure.tif]

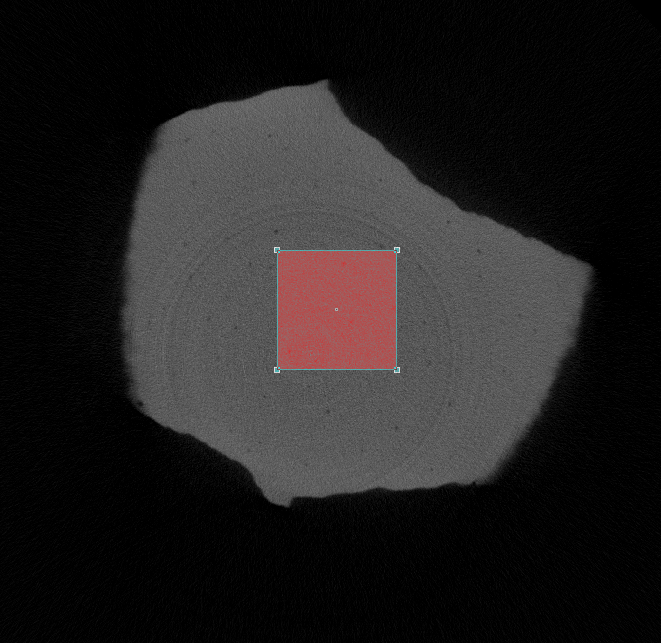

Supplement: Supplementary file 12 — Supplementary Data 12 [file 41467_2023_36405_MOESM12_ESM.zip › Micro_CT_raw_data/Mullerornis/AD2111/Results/ROI Selection.tif]

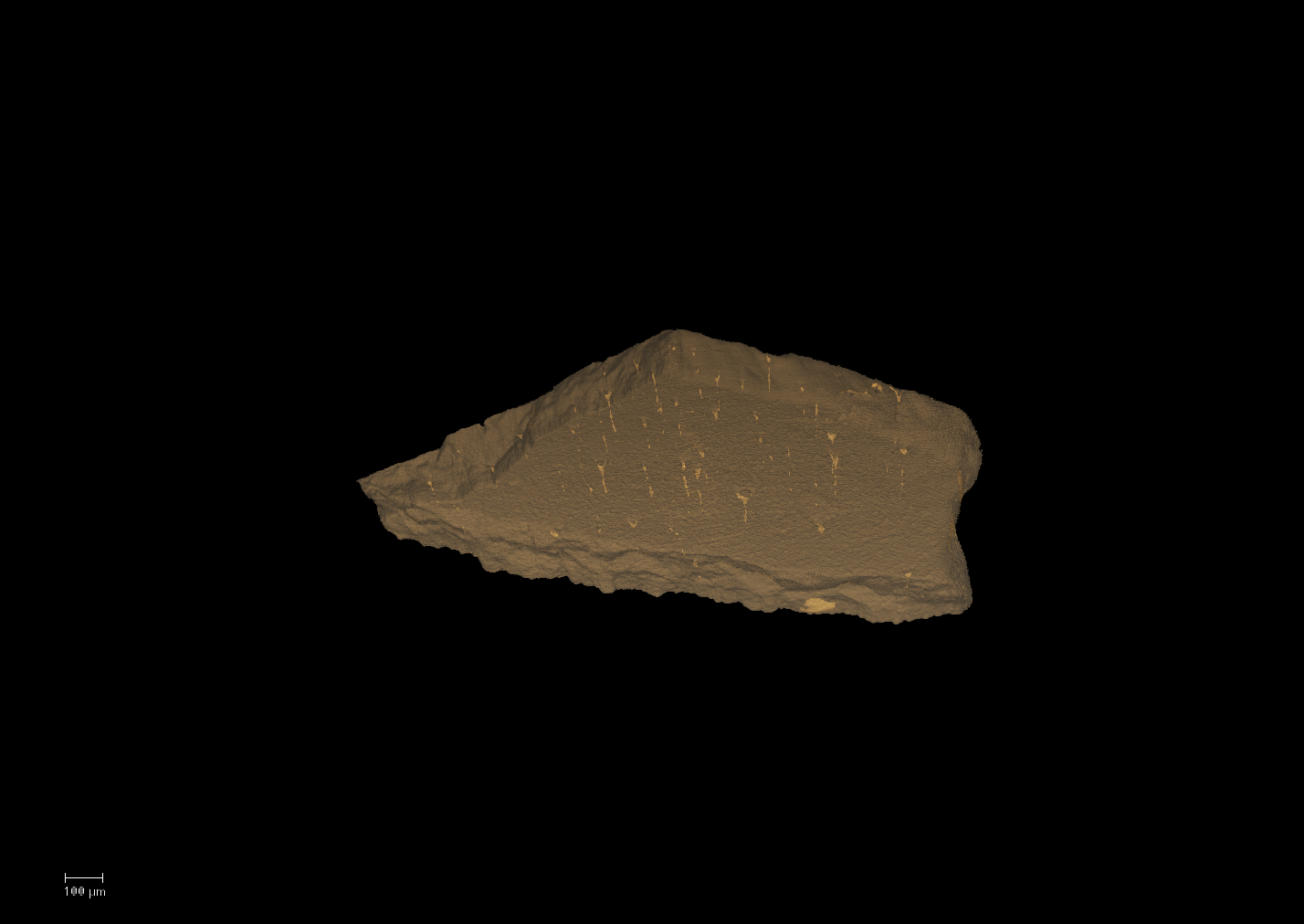

Supplement: Supplementary file 12 — Supplementary Data 12 [file 41467_2023_36405_MOESM12_ESM.zip › Micro_CT_raw_data/Mullerornis/AD2113/Results/snapshot3.tif]

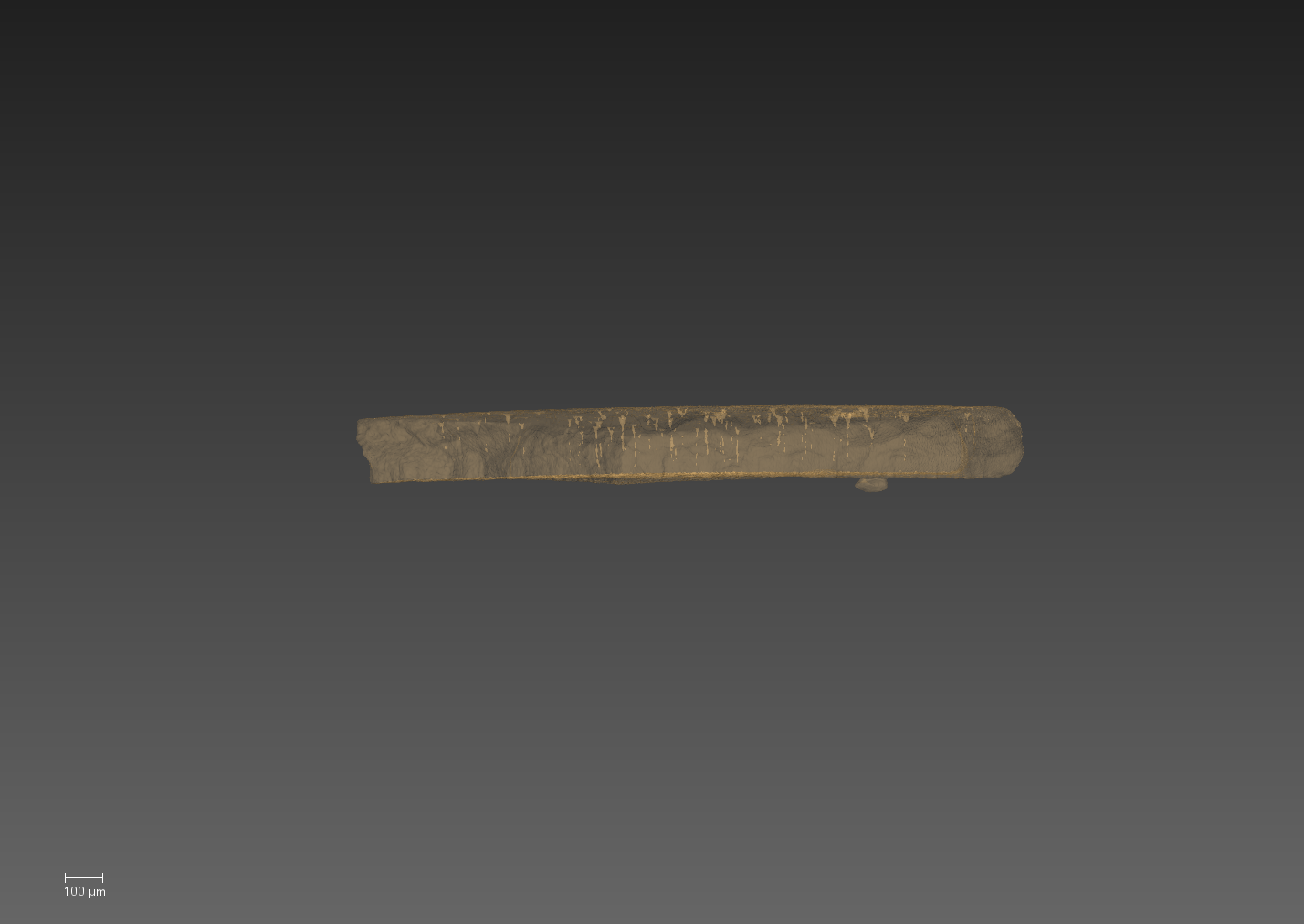

Supplement: Supplementary file 12 — Supplementary Data 12 [file 41467_2023_36405_MOESM12_ESM.zip › Micro_CT_raw_data/Mullerornis/AD2113/Results/snapshot2.tif]

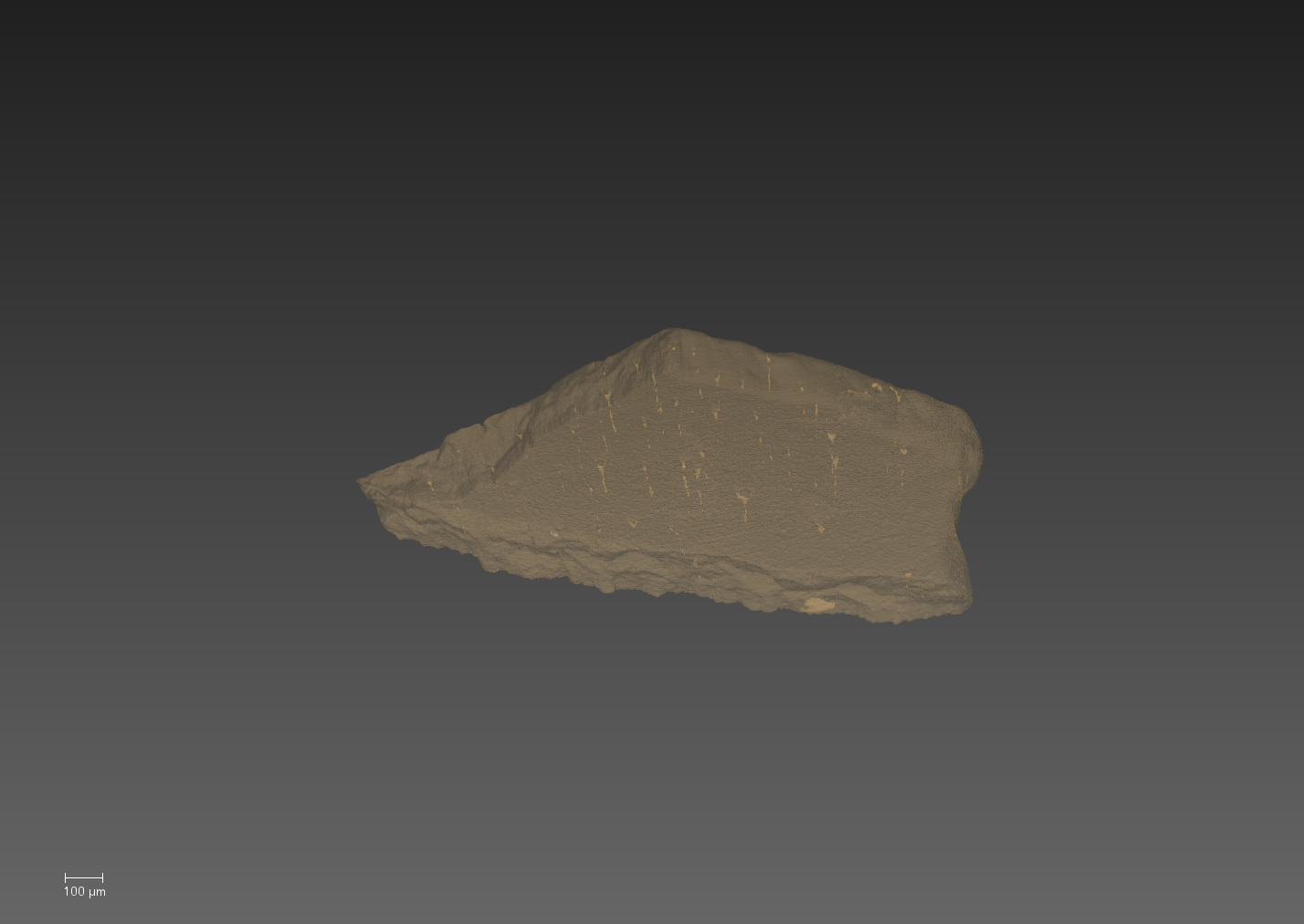

Supplement: Supplementary file 12 — Supplementary Data 12 [file 41467_2023_36405_MOESM12_ESM.zip › Micro_CT_raw_data/Mullerornis/AD2113/Results/snapshot1.tif]

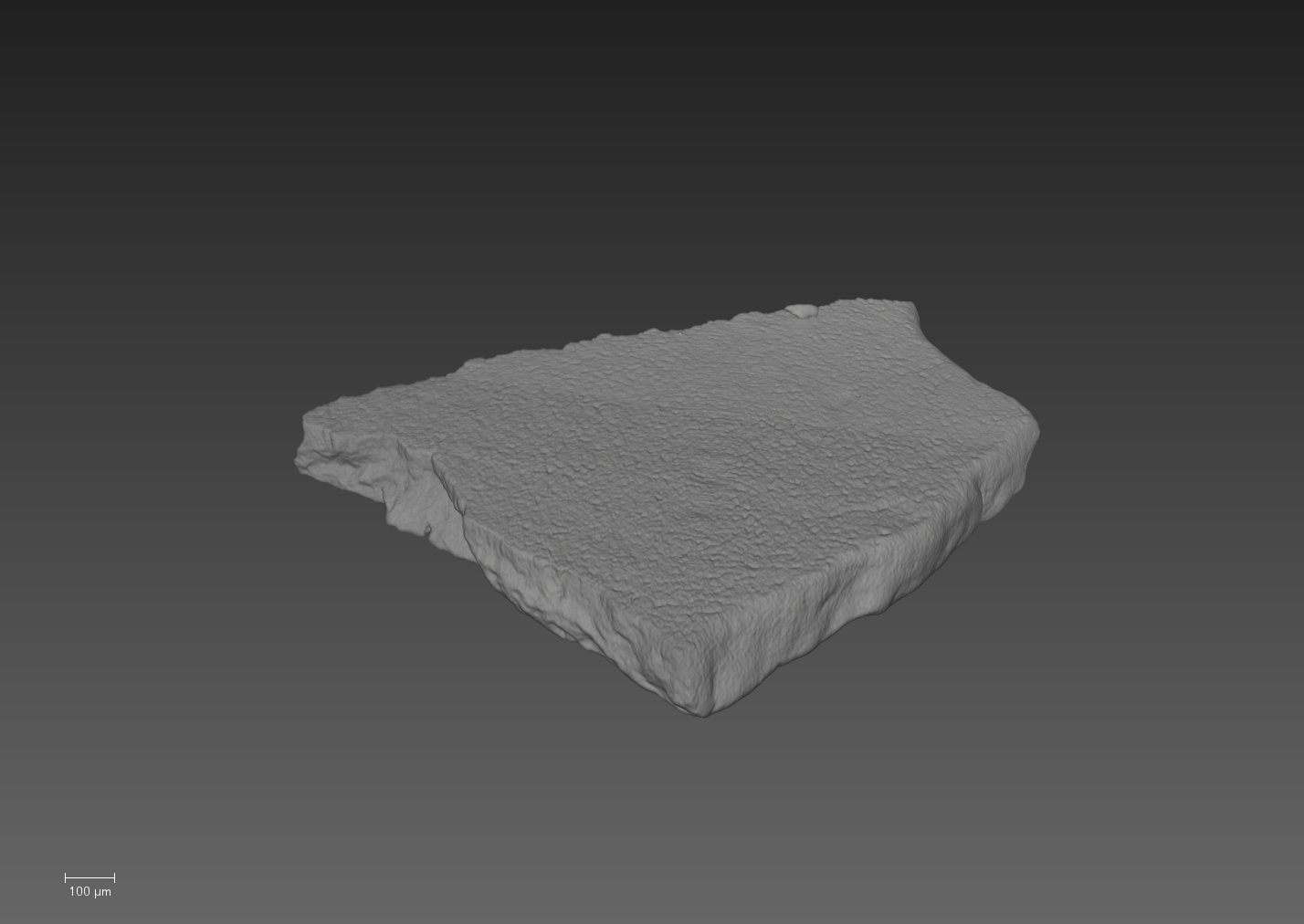

Supplement: Supplementary file 12 — Supplementary Data 12 [file 41467_2023_36405_MOESM12_ESM.zip › Micro_CT_raw_data/Mullerornis/AD2113/Results/Inner surface.tif]

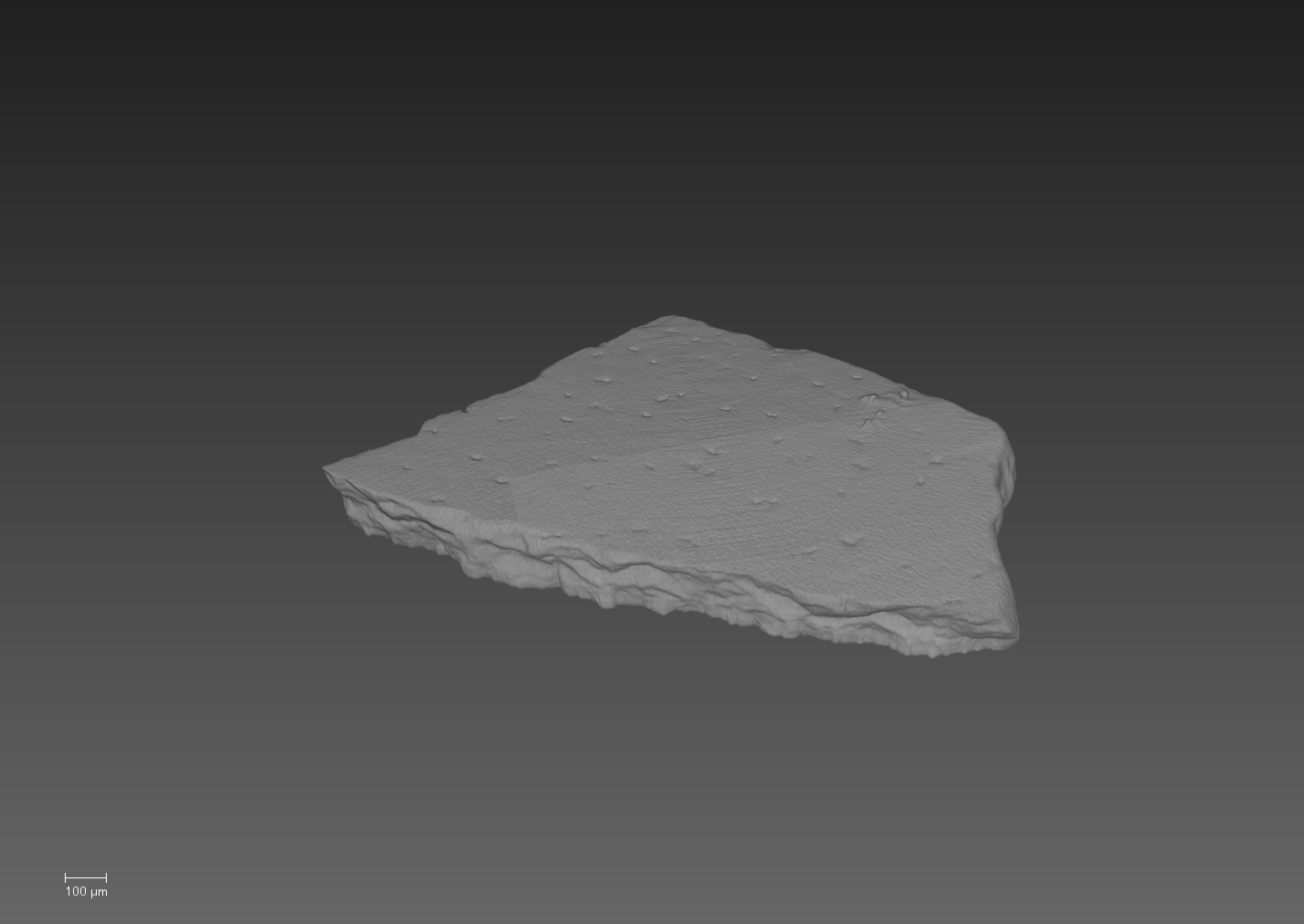

Supplement: Supplementary file 12 — Supplementary Data 12 [file 41467_2023_36405_MOESM12_ESM.zip › Micro_CT_raw_data/Mullerornis/AD2113/Results/Outer surface.tif]

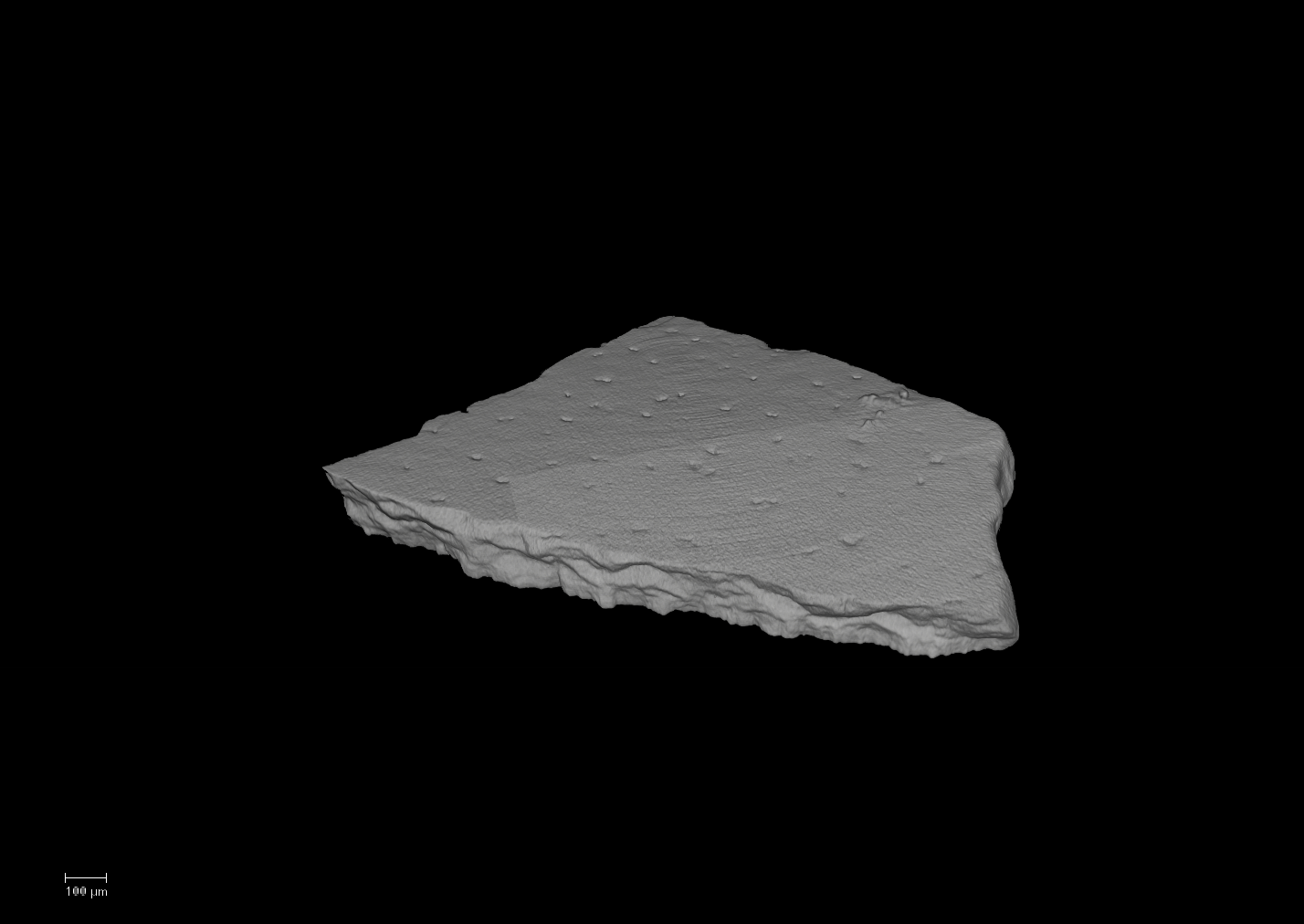

Supplement: Supplementary file 12 — Supplementary Data 12 [file 41467_2023_36405_MOESM12_ESM.zip › Micro_CT_raw_data/Mullerornis/AD2113/Results/Outer surface2.tif]

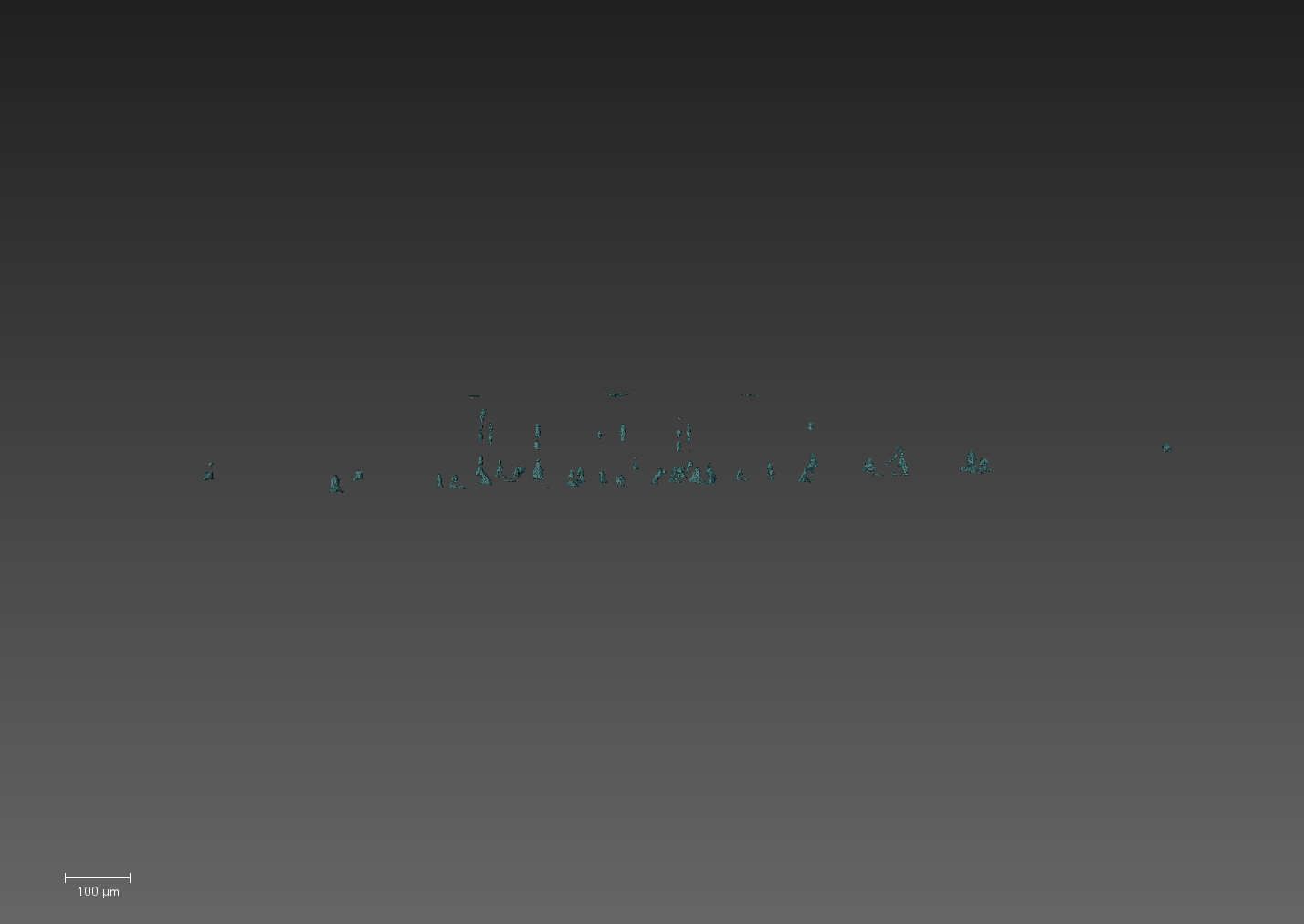

Supplement: Supplementary file 12 — Supplementary Data 12 [file 41467_2023_36405_MOESM12_ESM.zip › Micro_CT_raw_data/Mullerornis/AD2113/Results/Pore structure.tif]

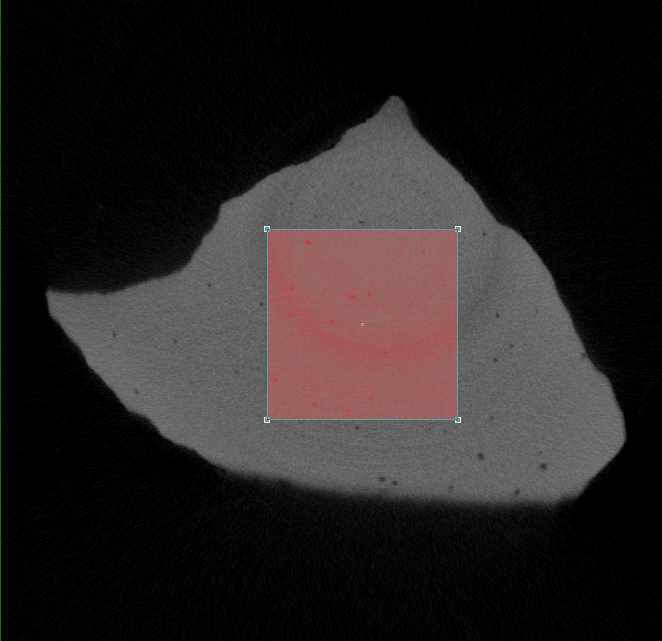

Supplement: Supplementary file 12 — Supplementary Data 12 [file 41467_2023_36405_MOESM12_ESM.zip › Micro_CT_raw_data/Mullerornis/AD2113/Results/ROI Selection.tif]

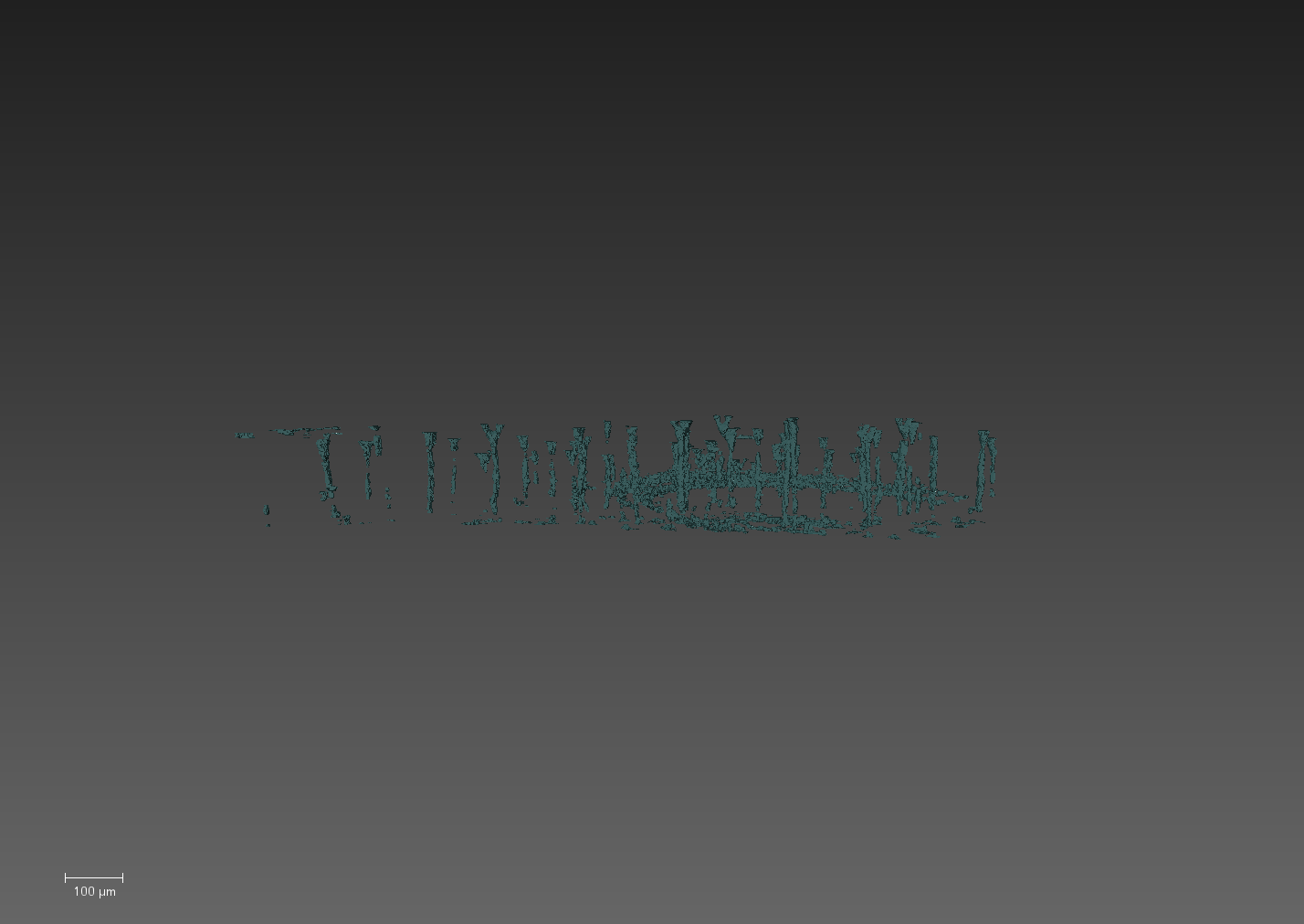

Supplement: Supplementary file 12 — Supplementary Data 12 [file 41467_2023_36405_MOESM12_ESM.zip › Micro_CT_raw_data/Mullerornis/AD2113/Results/Pore structure2.tif]

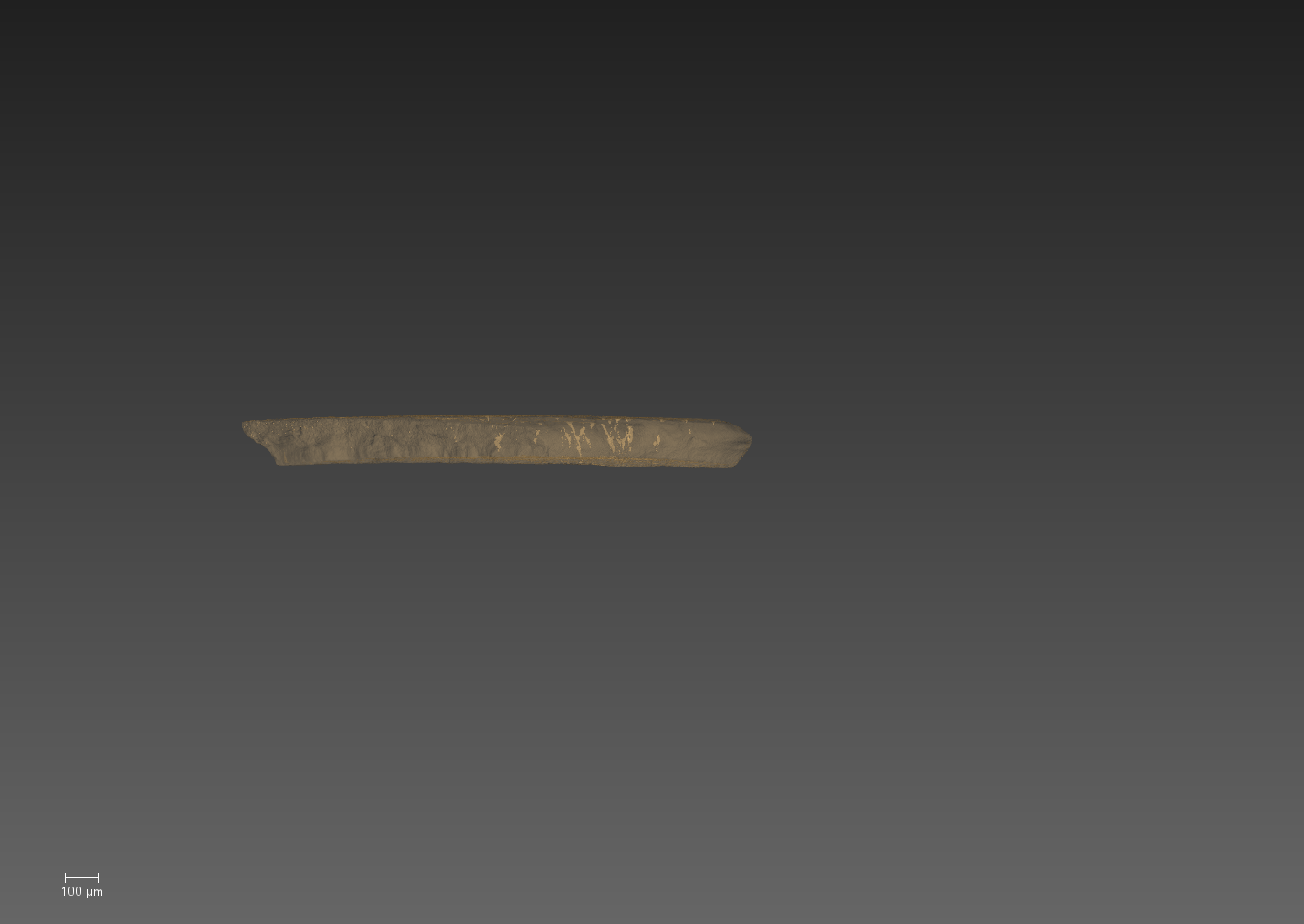

Supplement: Supplementary file 12 — Supplementary Data 12 [file 41467_2023_36405_MOESM12_ESM.zip › Micro_CT_raw_data/Mullerornis/AD2125/Results/snapshot2.tif]

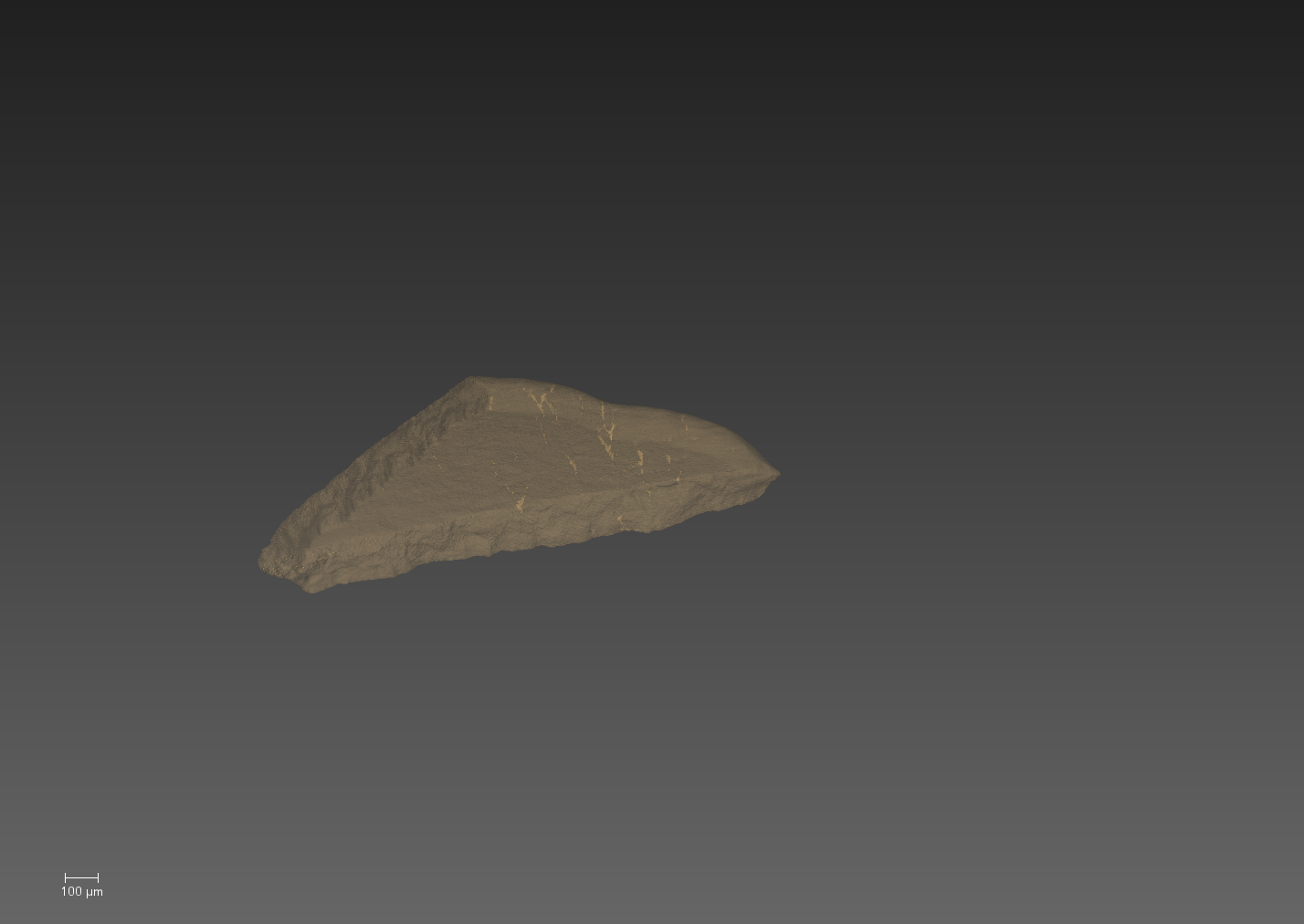

Supplement: Supplementary file 12 — Supplementary Data 12 [file 41467_2023_36405_MOESM12_ESM.zip › Micro_CT_raw_data/Mullerornis/AD2125/Results/snapshot.tif]

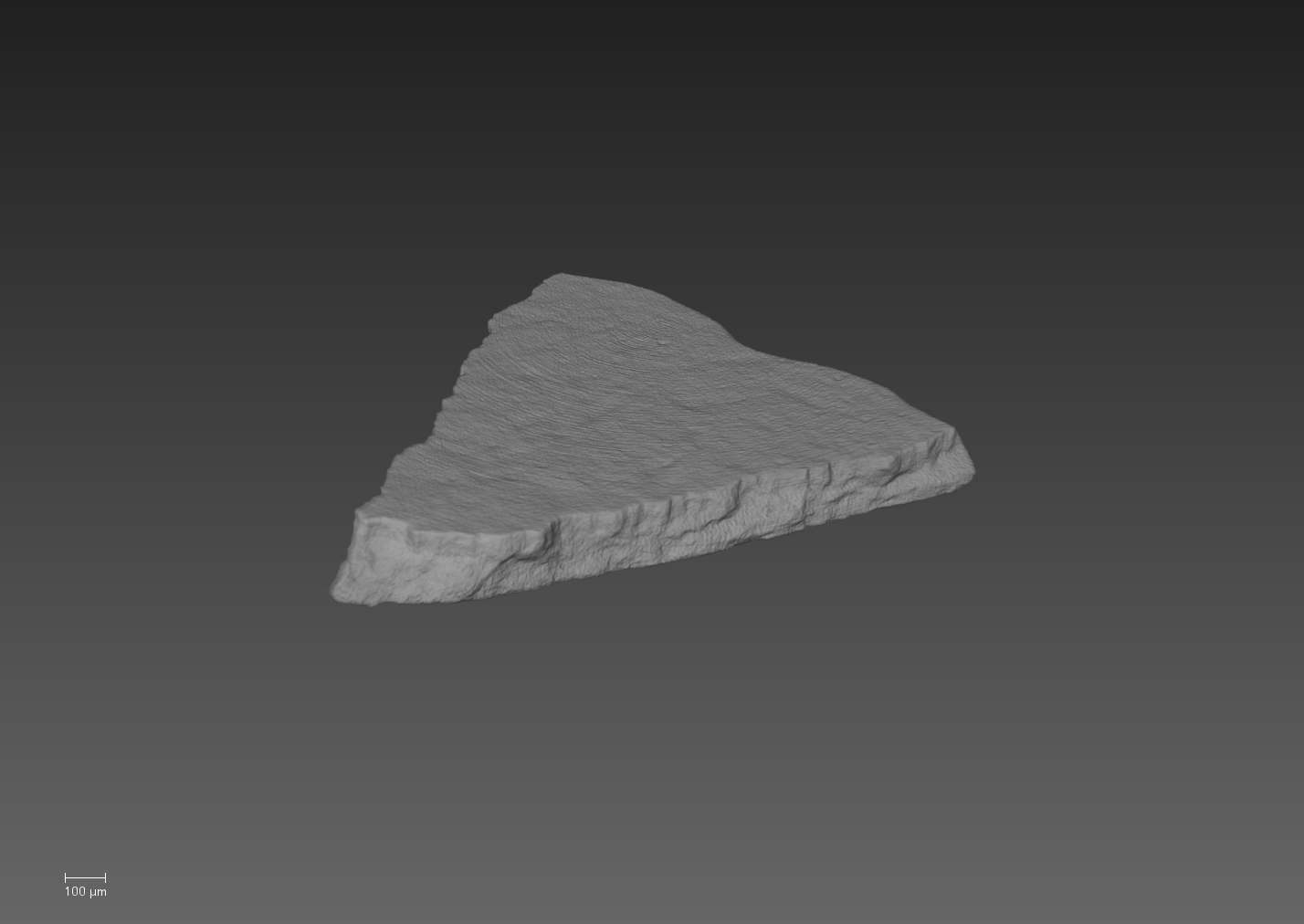

Supplement: Supplementary file 12 — Supplementary Data 12 [file 41467_2023_36405_MOESM12_ESM.zip › Micro_CT_raw_data/Mullerornis/AD2125/Results/Inner surface.tif]

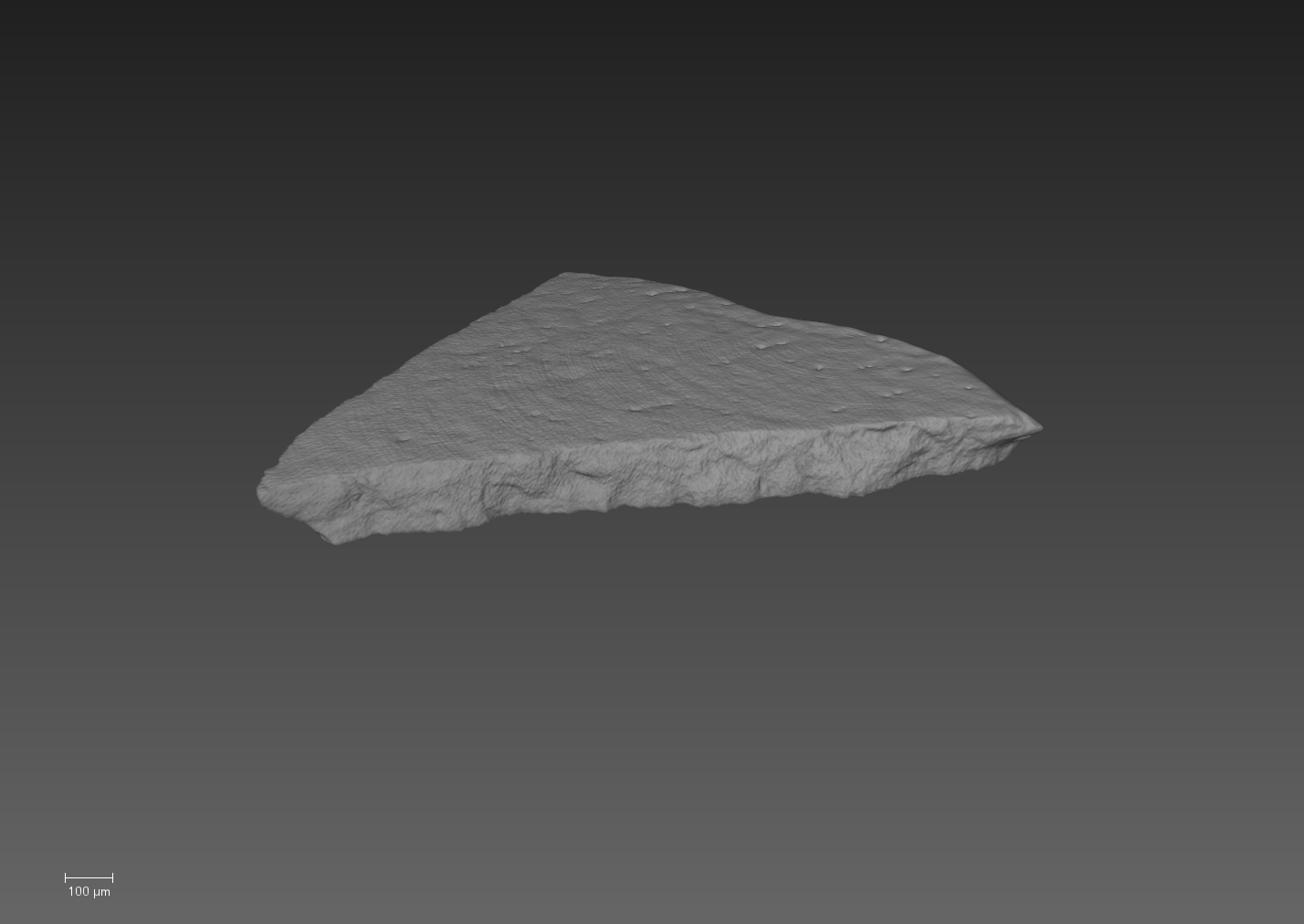

Supplement: Supplementary file 12 — Supplementary Data 12 [file 41467_2023_36405_MOESM12_ESM.zip › Micro_CT_raw_data/Mullerornis/AD2125/Results/Outer surface.tif]

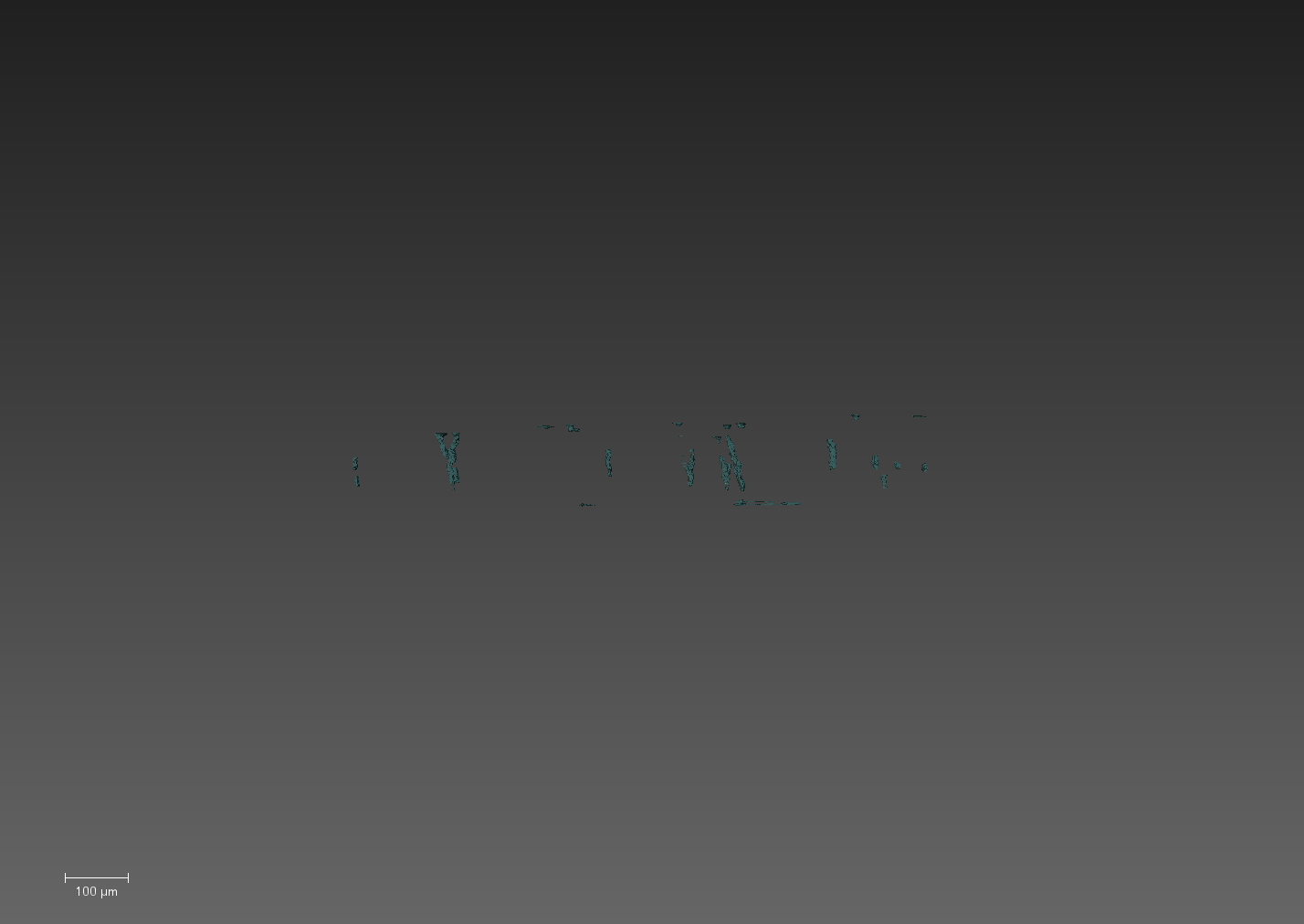

Supplement: Supplementary file 12 — Supplementary Data 12 [file 41467_2023_36405_MOESM12_ESM.zip › Micro_CT_raw_data/Mullerornis/AD2125/Results/Pore structure.tif]

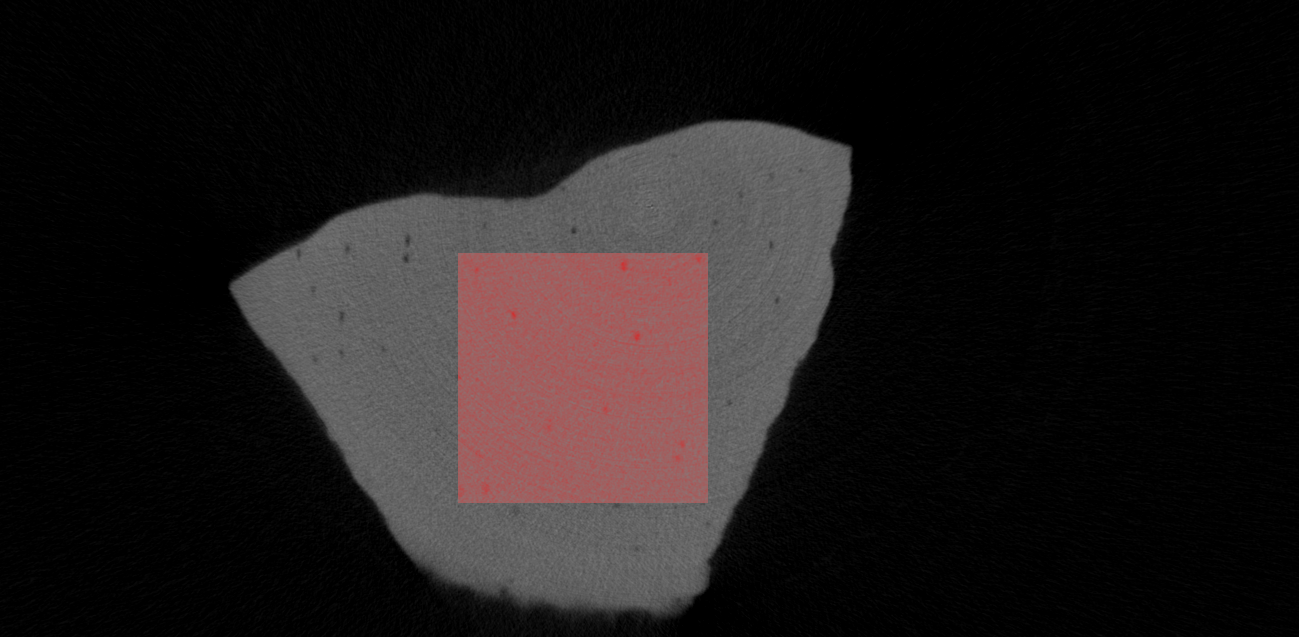

Supplement: Supplementary file 12 — Supplementary Data 12 [file 41467_2023_36405_MOESM12_ESM.zip › Micro_CT_raw_data/Mullerornis/AD2125/Results/ROI selection.tif]

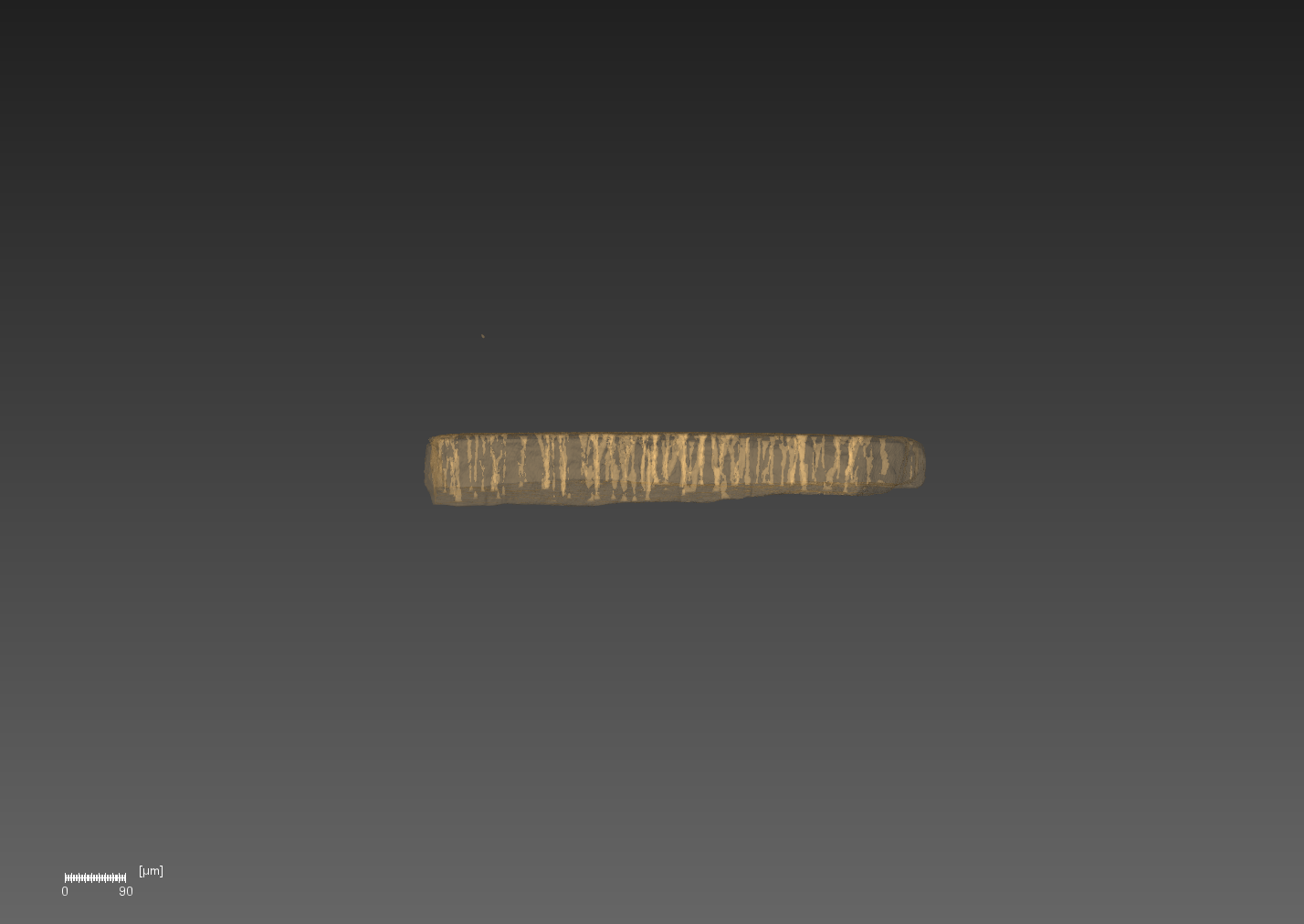

Supplement: Supplementary file 12 — Supplementary Data 12 [file 41467_2023_36405_MOESM12_ESM.zip › Micro_CT_raw_data/Mullerornis/AD2112/Results/snapshot2.tif]

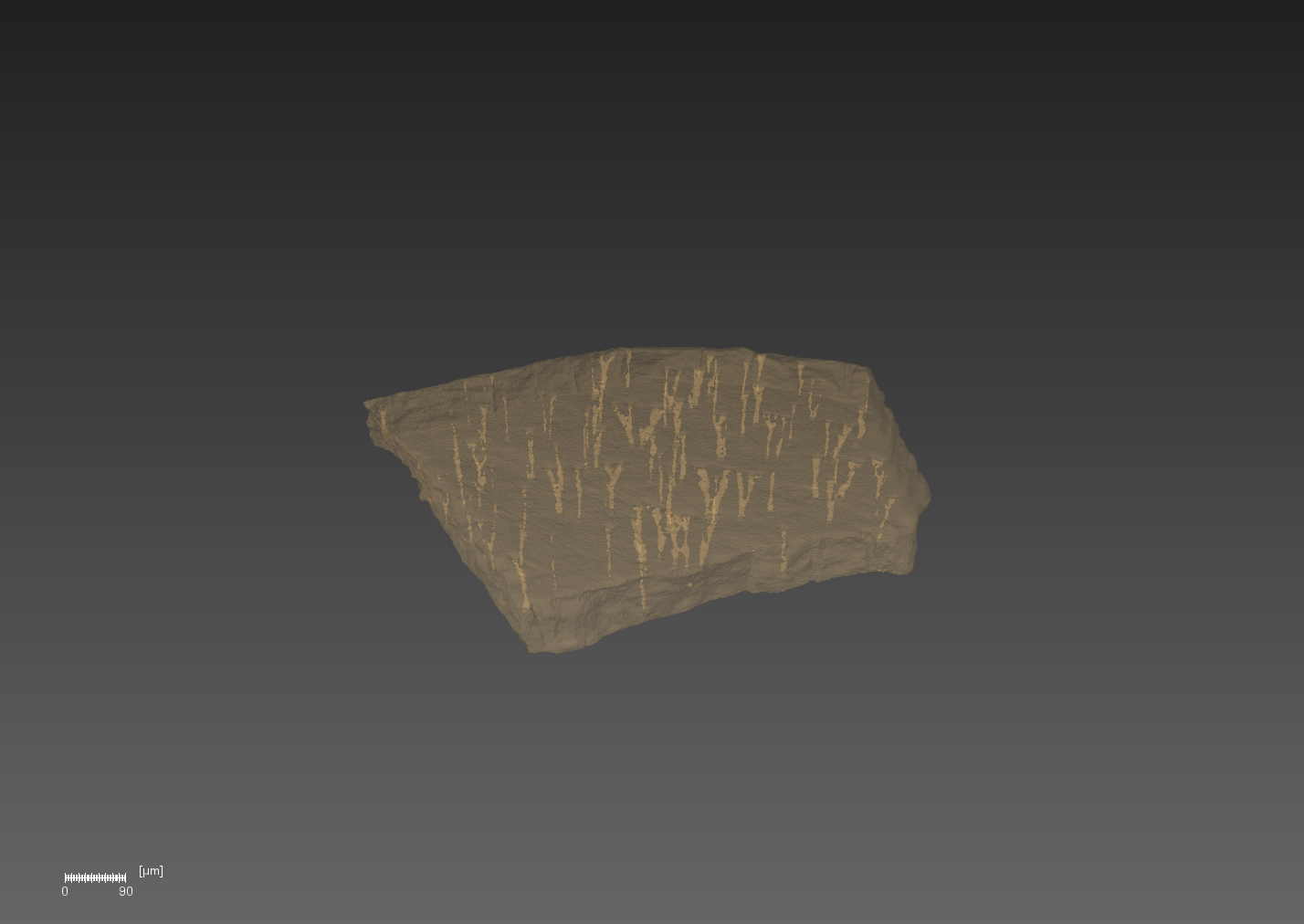

Supplement: Supplementary file 12 — Supplementary Data 12 [file 41467_2023_36405_MOESM12_ESM.zip › Micro_CT_raw_data/Mullerornis/AD2112/Results/snapshot1.tif]

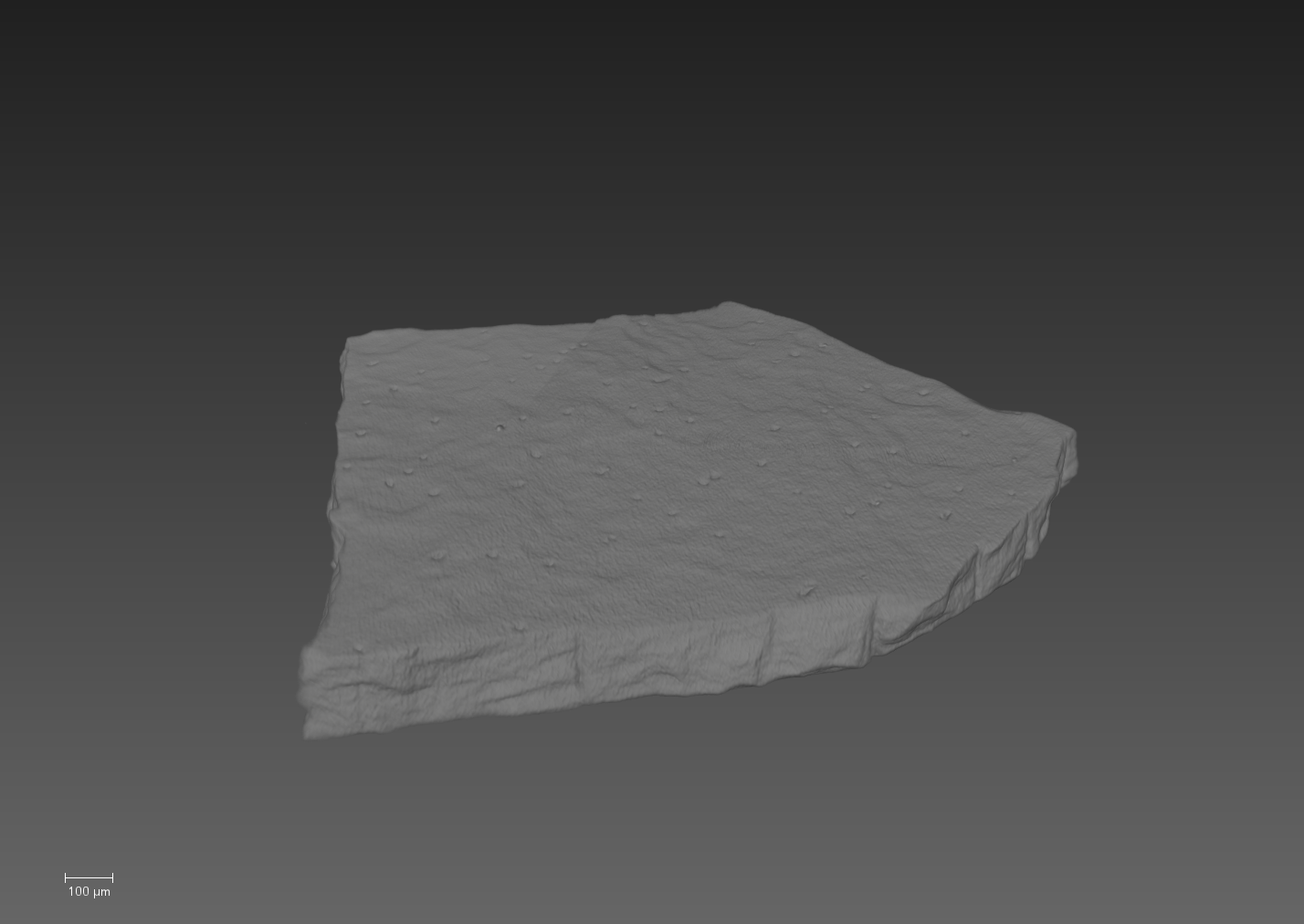

Supplement: Supplementary file 12 — Supplementary Data 12 [file 41467_2023_36405_MOESM12_ESM.zip › Micro_CT_raw_data/Mullerornis/AD2112/Results/Inner surface.tif]

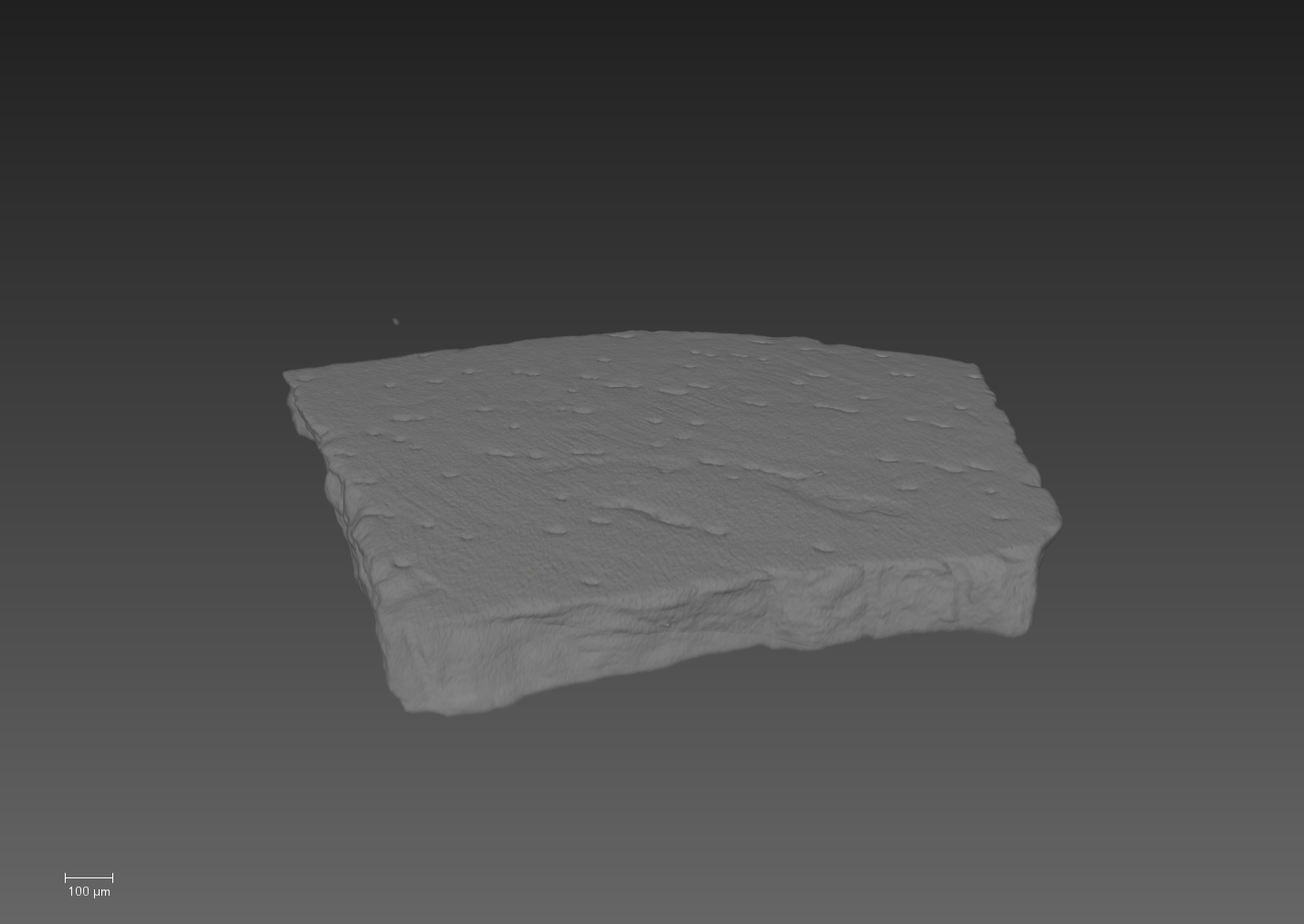

Supplement: Supplementary file 12 — Supplementary Data 12 [file 41467_2023_36405_MOESM12_ESM.zip › Micro_CT_raw_data/Mullerornis/AD2112/Results/Outer surface.tif]

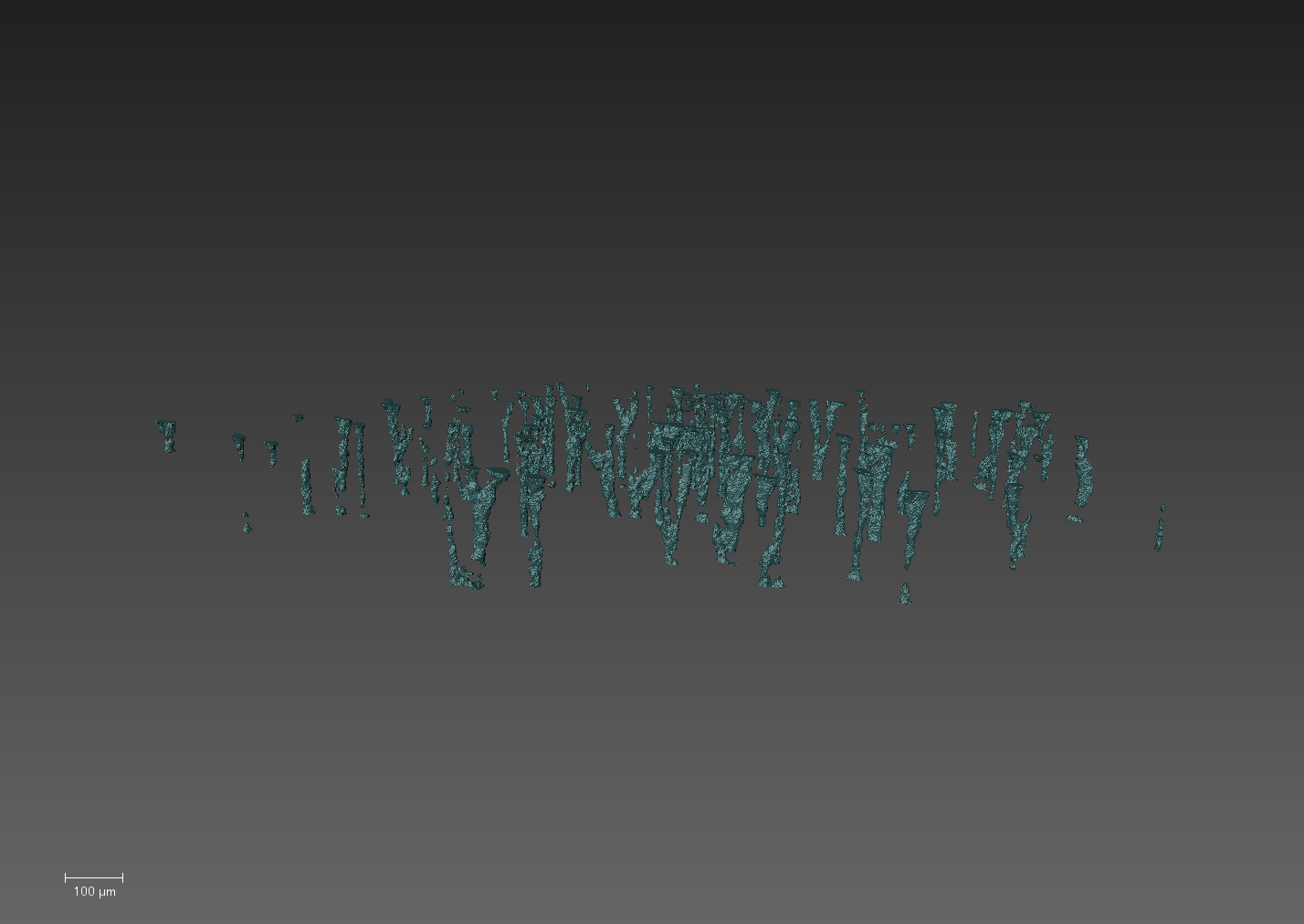

Supplement: Supplementary file 12 — Supplementary Data 12 [file 41467_2023_36405_MOESM12_ESM.zip › Micro_CT_raw_data/Mullerornis/AD2112/Results/Pore structure.tif]
