# Supplementary material for: Molecular exploration of fossil eggshell uncovers hidden lineage of giant extinct bird
Source: Nat Commun. 2023 Feb 28;14:914. doi: 10.1038/s41467-023-36405-3 (PMC9974994; doi:10.1038/s41467-023-36405-3)
Supplement: Supplementary file 11 — Supplementary Data 11 [file 41467_2023_36405_MOESM11_ESM.zip › Ancestral_state_reconstruction/Supplementary Code 1.html]

 

 

 

 
 
 


 

 

 Supplementary Code – Grealy et al. (2022) 

 
 
 
 
 
 
 
 
 
 
 
 
 
 
 
 

 

 
 


 


 

 

 


 


 

 


 


 
 
 
 
 
 

 


 


 Supplementary Code – Grealy et
al. (2022) 
 Lucas Legendre 
 06/29/2022 

 


 Compiled under R version 4.2.0 (2022-04-22) 
  WARNING : edit the working directory to your preferred
folder. 
 I chose to use the dataset from our recent paper (Legendre and
Clarke, 2021), because using only a small sample of ~10 paleognaths
would not have allowed me to build models with good predictive power.
However, since our data included the dwarf cassowary and two additional
species of kiwi that we did not have, we got data for eggshell
thickness, egg mass, and body mass for those three species. 
 References for egg mass and eggshell thickness data come from: 
–  Apteryx haastii : eggshell thickness: Vieco-Galvez et
al. (2021); egg mass: Body and Reid (1987); 
–  Apteryx owenii : Schönwetter (1960–1992); 
–  Casuarius bennetti : Schönwetter (1960–1992) – see also
Silyn-Roberts and Sharp (1985). 
Body mass taken from Dunning (2008) for all three species; divergence
times between them and other paleognaths taken from Yonezawa et
al. (2017). 
  Note : Values taken from Schönwetter (1960–1992) are
estimates calculated from egg dimensions and shell weight, not actual
measurements – see Maurer et al. (2010). 
 Body mass estimates for  Aepyornis hildebrandti  and
 Mullerornis agilis , which we did not sample in Legendre and
Clarke (2021), are taken from Crouch and Clarke (2019). 
  Update after first round of reviews : All body masses
for large extant flightless paleognaths (n = 9) have been replaced with
those of male members of the species to account for incubation
constraints, since in all of them the males incubate the eggs
(e.g. Huynen, 2010). Male body masses were taken from Olson and Turvey
(2013). The body mass for  Casuarius bennetti , for which no male
body mass data were available, was left as such. Additionally, estimates
of body mass for fossil taxa have been removed from the analysis in
order to estimate body mass in elephant birds solely from extant bird
data. 
 
 Loading packages 
 
  library(ape)
library(nlme)
library(dplyr)
library(ggplot2)
library(phytools)
library(AICcmodavg)
library(evobiR)
library(rr2)
library(caper)
library(geiger)
library(windex)
library(RColorBrewer)
library(wesanderson)
library(treeio)  
 
 Tree and data 
 
  treeAG<-read.nexus("treeAG_newanalyses.nex")
dataAG<-read.table("Paleognathdata_maleBM.txt", header=T)
treeAGnew<-drop.tip(treeAG, setdiff(treeAG$tip.label, dataAG$Species))
plotTree(treeAGnew)  
   
  rownames(dataAG)<-dataAG$Species
dataAG<-ReorderData(treeAGnew, dataAG, taxa.names="row names")  
 
 PGLS 
 
 With egg mass as predictor 
  # Data and tree
dataEM<-subset(dataAG, !is.na(dataAG$Eggmass))
treeEM<-drop.tip(treeAGnew, setdiff(treeAGnew$tip.label, dataEM$Species))

# Correcting the variance-covariance matrix for the non-ultrametric tree
Wem<-diag(vcv.phylo(treeEM))

# Best fit for the alpha parameter in the Ornstein-Uhlenbeck (OU) model
alpha <- seq(0, 1, 0.1)
fit <- list()
form <- log(Thickness)~log(Eggmass)
for (i in seq_along(alpha)) {
  cor <- corMartins(alpha[i], phy = treeEM, fixed = T)
  fit[[i]] <- gls(form, correlation = cor, data = dataEM, weights=varFixed(~Wem), na.action=na.exclude, method = "ML")
}
plot(sapply(fit, logLik)) # best fit: 0.1  
   
  # Best fit for the g parameter in the Early Burst (EB) model
g <- seq(0.1, 1, 0.1)
fit <- list()
form <- log(Thickness)~log(Eggmass)
for (i in seq_along(g)) {
  cor <- corBlomberg(g[i], phy = treeEM, fixed = T, form=~1)
  fit[[i]] <- gls(form, correlation = cor, data = dataEM, weights=varFixed(~Wem), na.action=na.exclude, method = "ML")
}
plot(sapply(fit, logLik)) # best fit: 0.2  
   
  # PGLS models
BM<-gls(log(Thickness)~log(Eggmass), data=dataEM, correlation=corBrownian(phy=treeEM), weights=varFixed(~Wem), method="ML")
OU<-gls(log(Thickness)~log(Eggmass), data=dataEM, correlation=corMartins(0.1, phy=treeEM, fixed=T), weights=varFixed(~Wem), method="ML")
Lambda<-gls(log(Thickness)~log(Eggmass), data=dataEM, correlation=corPagel(1, phy=treeEM), weights=varFixed(~Wem), method="ML")
EB<-gls(log(Thickness)~log(Eggmass), data=dataEM, correlation=corBlomberg(0.2, phy=treeEM, fixed=T), weights=varFixed(~Wem), method="ML")
OLS<-gls(log(Thickness)~log(Eggmass), data=dataEM, method="ML")

Cand.models = list()
Cand.models[[1]] = BM
Cand.models[[2]] = OU
Cand.models[[3]] = Lambda
Cand.models[[4]] = EB
Cand.models[[5]] = OLS

Modnames = paste(c("BM", "OU", "Lambda", "EB", "OLS"), sep = " ")
aictab(cand.set = Cand.models, modnames = Modnames, sort = T)  
 
 
 
  # Best model
summary(Lambda)  
  ## Generalized least squares fit by maximum likelihood
##   Model: log(Thickness) ~ log(Eggmass) 
##   Data: dataEM 
##         AIC      BIC   logLik
##   -18.87435 -10.5642 13.43718
## 
## Correlation Structure: corPagel
##  Formula: ~1 
##  Parameter estimate(s):
##  lambda 
## 0.81927 
## Variance function:
##  Structure: fixed weights
##  Formula: ~Wem 
## 
## Coefficients:
##                 Value Std.Error  t-value p-value
## (Intercept)  4.043591 0.1399570 28.89167       0
## log(Eggmass) 0.439850 0.0216308 20.33441       0
## 
##  Correlation: 
##              (Intr)
## log(Eggmass) -0.657
## 
## Standardized residuals:
##        Min         Q1        Med         Q3        Max 
## -3.1831148 -0.4604374 -0.1068960  0.5120886  1.9226593 
## 
## Residual standard error: 0.02176148 
## Degrees of freedom: 59 total; 57 residual  
  R2.pred(Lambda) # R2 = 0.96: very high pseudo R-squared  
  ## [1] 0.958176  
  # Compare with estimate from 'caper'
datacompEM<-comparative.data(treeEM, dataEM, names.col="Species")
pglsEM<-pgls(log(Thickness)~log(Eggmass), datacompEM, lambda="ML")
summary(pglsEM) # highly significant  
  ## 
## Call:
## pgls(formula = log(Thickness) ~ log(Eggmass), data = datacompEM, 
##     lambda = "ML")
## 
## Residuals:
##       Min        1Q    Median        3Q       Max 
## -0.050075 -0.007712  0.001303  0.011582  0.077122 
## 
## Branch length transformations:
## 
## kappa  [Fix]  : 1.000
## lambda [ ML]  : 0.885
##    lower bound : 0.000, p = 1.9637e-06
##    upper bound : 1.000, p = 0.0039169
##    95.0% CI   : (0.636, 0.980)
## delta  [Fix]  : 1.000
## 
## Coefficients:
##              Estimate Std. Error t value  Pr(>|t|)    
## (Intercept)  4.037575   0.149339  27.036 < 2.2e-16 ***
## log(Eggmass) 0.441647   0.023205  19.033 < 2.2e-16 ***
## ---
## Signif. codes:  0 '***' 0.001 '**' 0.01 '*' 0.05 '.' 0.1 ' ' 1
## 
## Residual standard error: 0.02202 on 54 degrees of freedom
## Multiple R-squared: 0.8703,  Adjusted R-squared: 0.8679 
## F-statistic: 362.2 on 1 and 54 DF,  p-value: < 2.2e-16  
 
 Plot of the regression and estimates of egg mass for elephant bird
eggshells 
 
  # Estimates based on the PGLS model
plotdataEM<-dataAG[,c(1:3,5)]
plotdataEM[c(57:62),4]<-exp((log(dataAG[c(57:62),3])-Lambda$coef[1])/Lambda$coef[2])

# Some of the estimates can be hard to differentiate from species with actual egg mass values
# If you want to see them, use this line of code:
plotdataEM[c(57:62),1]<-"Predicted"

# Plot the regression with the estimates on the regression line
ggplot(plotdataEM, aes(log(Eggmass), log(Thickness), color=Clade)) +
  geom_point(size=5) +
  geom_text(aes(label=Species),hjust=-0.1, vjust=0.4) +
  xlab("ln egg mass (g)") +
  ylab("ln eggshell thickness (µm)") +
  geom_abline(intercept=Lambda$coefficients[1], slope=Lambda$coefficients[2],
              colour="skyblue2", size=1.3) +
  theme(panel.background = element_rect(fill="black")) +
  scale_colour_brewer("Clade", palette="Dark2")  
  ## Warning: Using `size` aesthetic for lines was deprecated in ggplot2 3.4.0.
## ℹ Please use `linewidth` instead.  
   
  # If you want to look at the values:
plotdataEM[c(57:62),c(2:4)]  
 
 
 
 Looking at the values on the plot, n2973 has the thinnest eggshell,
and ranges close to rhea, emu, and cassowaries. n07AEP06 is a bit
larger, between rhea/emu/cassowary and  Emeus . All other samples
have values of egg mass similar to, or larger than that of,
 Aepyornis .  This seems to confirm the phylogenetic pattern
from the molecular tree  – n07AEP06 and n2973 forming a clade
with  Mullerornis , and the other three grouped with
 Aepyornis . 
 
 
 With body mass as predictor 
  # Data and tree
dataBM<-subset(dataAG, !is.na(dataAG$Bodymass))
treeBM<-drop.tip(treeAGnew, setdiff(treeAGnew$tip.label, dataBM$Species))

# Correcting the variance-covariance matrix for the non-ultrametric tree
Wbm<-diag(vcv.phylo(treeBM))

# Best fit for the alpha parameter in the Ornstein-Uhlenbeck (OU) model
alpha <- seq(0, 1, 0.1)
fit <- list()
form <- log(Thickness)~log(Bodymass)
for (i in seq_along(alpha)) {
  cor <- corMartins(alpha[i], phy = treeBM, fixed = T)
  fit[[i]] <- gls(form, correlation = cor, data = dataBM, weights=varFixed(~Wbm), na.action=na.exclude, method = "ML")
}
plot(sapply(fit, logLik)) # best fit: 1  
   
  # Best fit for the g parameter in the Early Burst (EB) model
g <- seq(0.1, 1, 0.1)
fit <- list()
form <- log(Thickness)~log(Bodymass)
for (i in seq_along(g)) {
  cor <- corBlomberg(g[i], phy = treeBM, fixed = T, form=~1)
  fit[[i]] <- gls(form, correlation = cor, data = dataBM, weights=varFixed(~Wbm), na.action=na.exclude, method = "ML")
}
plot(sapply(fit, logLik)) # best fit: 0.1  
   
  # Best fit for Pagel's lambda in the Lambda model
lambda <- seq(0, 1, 0.1)
fit <- list()
form <- log(Thickness)~log(Bodymass)
for (i in seq_along(alpha)) {
  cor <- corPagel(lambda[i], phy = treeBM, fixed = T)
  fit[[i]] <- gls(form, correlation = cor, data = dataBM, weights=varFixed(~Wbm), na.action=na.exclude, method = "ML")
}
plot(sapply(fit, logLik)) # best fit: 0.7  
   
  # PGLS models
BM<-gls(log(Thickness)~log(Bodymass), data=dataBM, correlation=corBrownian(phy=treeBM), weights=varFixed(~Wbm), method="ML")
OU<-gls(log(Thickness)~log(Bodymass), data=dataBM, correlation=corMartins(0.1, phy=treeBM, fixed=T), weights=varFixed(~Wbm), method="ML")
Lambda<-gls(log(Thickness)~log(Bodymass), data=dataBM, correlation=corPagel(0.7, phy=treeBM, fixed=T), weights=varFixed(~Wbm), method="ML")
EB<-gls(log(Thickness)~log(Bodymass), data=dataBM, correlation=corBlomberg(0.1, phy=treeBM, fixed=T), weights=varFixed(~Wbm), method="ML")
OLS<-gls(log(Thickness)~log(Bodymass), data=dataBM, method="ML")

Cand.models = list()
Cand.models[[1]] = BM
Cand.models[[2]] = OU
Cand.models[[3]] = Lambda
Cand.models[[4]] = EB
Cand.models[[5]] = OLS

Modnames = paste(c("BM", "OU", "Lambda", "EB", "OLS"), sep = " ")
aictab(cand.set = Cand.models, modnames = Modnames, sort = T)  
 
 
 
  # Best model
summary(Lambda)  
  ## Generalized least squares fit by maximum likelihood
##   Model: log(Thickness) ~ log(Bodymass) 
##   Data: dataBM 
##         AIC       BIC   logLik
##   -23.13459 -17.05853 14.56729
## 
## Correlation Structure: corPagel
##  Formula: ~1 
##  Parameter estimate(s):
## lambda 
##    0.7 
## Variance function:
##  Structure: fixed weights
##  Formula: ~Wbm 
## 
## Coefficients:
##                  Value  Std.Error  t-value p-value
## (Intercept)   3.400739 0.15126099 22.48259       0
## log(Bodymass) 0.339752 0.01644095 20.66496       0
## 
##  Correlation: 
##               (Intr)
## log(Bodymass) -0.788
## 
## Standardized residuals:
##         Min          Q1         Med          Q3         Max 
## -1.73276030 -0.54666777  0.05129172  0.51538557  2.05149883 
## 
## Residual standard error: 0.01978636 
## Degrees of freedom: 56 total; 54 residual  
  R2.pred(Lambda) # R2 = 0.95: very high pseudo R-squared  
  ## [1] 0.9481275  
  # Compare with estimate from 'caper'
datacompBM<-comparative.data(treeBM, dataBM, names.col="Species")
pglsBM<-pgls(log(Thickness)~log(Bodymass), datacompBM, lambda="ML")
summary(pglsBM) # highly significant  
  ## 
## Call:
## pgls(formula = log(Thickness) ~ log(Bodymass), data = datacompBM, 
##     lambda = "ML")
## 
## Residuals:
##       Min        1Q    Median        3Q       Max 
## -0.040872 -0.010882  0.003683  0.013769  0.053600 
## 
## Branch length transformations:
## 
## kappa  [Fix]  : 1.000
## lambda [ ML]  : 0.698
##    lower bound : 0.000, p = 0.48374
##    upper bound : 1.000, p = 0.00035266
##    95.0% CI   : (NA, 0.953)
## delta  [Fix]  : 1.000
## 
## Coefficients:
##               Estimate Std. Error t value  Pr(>|t|)    
## (Intercept)   3.400276   0.151080  22.506 < 2.2e-16 ***
## log(Bodymass) 0.339808   0.016431  20.681 < 2.2e-16 ***
## ---
## Signif. codes:  0 '***' 0.001 '**' 0.01 '*' 0.05 '.' 0.1 ' ' 1
## 
## Residual standard error: 0.02013 on 54 degrees of freedom
## Multiple R-squared: 0.8879,  Adjusted R-squared: 0.8858 
## F-statistic: 427.7 on 1 and 54 DF,  p-value: < 2.2e-16  
 
 Plot of the regression and estimates of body mass for elephant bird
eggshells 
 
  # Estimates based on the OLS model
plotdataBM<-dataAG[,c(1:4)]
plotdataBM[c(58:62),4]<-exp((log(dataAG[c(58:62),3])-Lambda$coefficients[1])/
                              Lambda$coefficients[2])

# Some of the estimates can be hard to differentiate from species with actual body mass values
# If you want to see them, use this line of code:
plotdataBM[c(58:62),1]<-"Predicted"

# Plot the regression with the estimates on the regression line
ggplot(plotdataBM, aes(log(Bodymass), log(Thickness), color=Clade)) +
  geom_point(size=5) +
  geom_text(aes(label=Species),hjust=-0.1, vjust=0.4) +
  xlab("ln body mass (kg)") +
  ylab("ln eggshell thickness (µm)") +
  geom_abline(intercept=Lambda$coefficients[1], slope=Lambda$coefficients[2],
              colour="skyblue2", size=1.3) +
  theme(panel.background = element_rect(fill="black")) +
  scale_color_manual(values=wes_palette("Zissou1")[c(1,3,5)])  
  ## Warning: Removed 4 rows containing missing values (`geom_point()`).  
  ## Warning: Removed 4 rows containing missing values (`geom_text()`).  
   
  # If you want to look at the values:
plotdataBM[c(58:62),c(2:4)]  
 
 
 
 As expected,  results are similar to those obtained for egg
mass and support the tree topology for the elephant bird eggshell
specimens . Body mass estimates for n08AEP07 and n1666 are
unrealistically high (over 900 and 1900 kg, respectively), suggesting
these specimens had very thick eggshells for their body mass (n1666 has
a 4 mm-thick eggshell, much higher than the 3.4 mm found in  Aepyornis
maximus ). This hypothesis is supported by the fact that  Aepyornis
hildebrandti , the large moa (attributed to  Dinornis ), and the
ostrich also have an eggshell thicker than expected for their body mass.
This might be a trend in giant birds to prevent shell breakage in such
large eggs (Birchard and Deeming, 2009). 
 
 
 
 Ancestral state reconstruction (for paleognaths) 
 
 For eggshell thickness, egg mass, and body mass 
 
 Prepare the plots 
 
  # Prepare the data and tree
dataAG2<-read.table("Paleognathdata.txt", header=TRUE)
rownames(dataAG2)<-dataAG2$Species
dataAG2<-ReorderData(treeAGnew, dataAG2, taxa.names="row names")
dataAG2[c(58:62),4]<-plotdataBM[c(58:62),4]
ASRdata<-cbind(plotdataEM,dataAG2[,4]); rownames(ASRdata)<-rownames(dataAG)
ASRdata<-filter(ASRdata, Clade=="Paleognaths"|Clade=="Predicted")
ASRtree<-drop.tip(treeAGnew, setdiff(treeAGnew$tip.label, ASRdata$Species))
ASRdata<-log(ASRdata[,3:5]); colnames(ASRdata)[3]<-"Bodymass"

# Phylogenetic signal (Pagel's lambda) of each variable
var=list(); phy=list()
for (i in 1:3) {
  var<-ASRdata[,i]; names(var)<-rownames(ASRdata)
  phy[[i]]<-phylosig(ASRtree, var, method="lambda", test=T)
}
phy  
  ## [[1]]
## 
## Phylogenetic signal lambda : 0.915632 
## logL(lambda) : -17.2669 
## LR(lambda=0) : 14.9578 
## P-value (based on LR test) : 0.000109941 
## 
## 
## [[2]]
## 
## Phylogenetic signal lambda : 0.893677 
## logL(lambda) : -31.8735 
## LR(lambda=0) : 12.8981 
## P-value (based on LR test) : 0.000328912 
## 
## 
## [[3]]
## 
## Phylogenetic signal lambda : 0.861943 
## logL(lambda) : -39.1053 
## LR(lambda=0) : 13.0664 
## P-value (based on LR test) : 0.000300638  
  # Check best evolutionary model
models=c("BM", "OU", "EB", "rate_trend","lambda", "white")
# If you need more information on each tested model, check '?fitContinuous')

var=list(); fit<-list(); mod<-list()
for (i in 1:ncol(ASRdata)) {
  var<-ASRdata[,i]; names(var)<-rownames(ASRdata)
  for (m in 1:length(models)) {
    fit[[m]]=fitContinuous(ASRtree, var, model=models[m], ncores=2)
  }
  mod[[i]]<-modSel.geiger(fit[[1]],fit[[2]],fit[[3]],fit[[4]],fit[[5]],fit[[6]])
}  
  ## Warning in fitContinuous(ASRtree, var, model = models[m], ncores = 2): Non-
## ultrametric tree with OU model, using VCV method.  
  ## Warning in cache$dat - mu: Recycling array of length 1 in vector-array arithmetic is deprecated.
##   Use c() or as.vector() instead.

## Warning in cache$dat - mu: Recycling array of length 1 in vector-array arithmetic is deprecated.
##   Use c() or as.vector() instead.  
  ## Warning in fitContinuous(ASRtree, var, model = models[m], ncores = 2): 
## Parameter estimates appear at bounds:
##  a  
  ## Warning in fitContinuous(ASRtree, var, model = models[m], ncores = 2): Non-
## ultrametric tree with OU model, using VCV method.  
  ## Warning in cache$dat - mu: Recycling array of length 1 in vector-array arithmetic is deprecated.
##   Use c() or as.vector() instead.

## Warning in cache$dat - mu: Recycling array of length 1 in vector-array arithmetic is deprecated.
##   Use c() or as.vector() instead.  
  ## Warning in fitContinuous(ASRtree, var, model = models[m], ncores = 2): 
## Parameter estimates appear at bounds:
##  slope  
  ## Warning in fitContinuous(ASRtree, var, model = models[m], ncores = 2): Non-
## ultrametric tree with OU model, using VCV method.  
  ## Warning in cache$dat - mu: Recycling array of length 1 in vector-array arithmetic is deprecated.
##   Use c() or as.vector() instead.

## Warning in cache$dat - mu: Recycling array of length 1 in vector-array arithmetic is deprecated.
##   Use c() or as.vector() instead.  
  mod # Lambda model is the best fit in all traits  
  ## [[1]]
##          K    logLik     AICc deltaAICc Weight Evidence ratio
## fit[[5]] 3 -17.26693 42.03386  0.000000 0.8887       1.000000
## fit[[2]] 3 -19.45035 46.40070  4.366840 0.1001       8.876611
## fit[[4]] 3 -22.13473 51.76947  9.735604 0.0068     130.034756
## fit[[6]] 2 -24.69330 54.09249 12.058622 0.0021     415.428644
## fit[[1]] 2 -24.87767 54.46123 12.427365 0.0018     499.537293
## fit[[3]] 3 -24.87783 57.25567 15.221803 0.0004    2020.098379
## 
## [[2]]
##          K    logLik     AICc deltaAICc Weight Evidence ratio
## fit[[5]] 3 -31.87351 71.24701  0.000000 0.8126       1.000000
## fit[[2]] 3 -33.41572 74.33145  3.084437 0.1738       4.674949
## fit[[6]] 2 -37.53969 79.78525  8.538244 0.0114      71.458864
## fit[[4]] 3 -38.07150 83.64300 12.395993 0.0017     491.762885
## fit[[1]] 2 -40.88224 86.47037 15.223359 0.0004    2021.670263
## fit[[3]] 3 -40.88241 89.26482 18.017814 0.0001    8175.580147
## 
## [[3]]
##          K    logLik      AICc deltaAICc Weight Evidence ratio
## fit[[5]] 3 -39.10529  85.71058  0.000000 0.7612       1.000000
## fit[[2]] 3 -40.31690  88.13380  2.423228 0.2266       3.358901
## fit[[6]] 2 -44.88334  94.47255  8.761979 0.0095      79.917055
## fit[[4]] 3 -45.06391  97.62782 11.917245 0.0020     387.076586
## fit[[1]] 2 -47.78970 100.28528 14.574704 0.0005    1461.694819
## fit[[3]] 3 -47.78987 103.07973 17.369159 0.0001    5911.053860  
  # Plots
dataplot=list(); fit=list(); obj=list()
for (i in 1:ncol(ASRdata)) {
  dataplot[[i]]<-as.matrix(ASRdata)[,i]
  fit[[i]]<-fastAnc(ASRtree, dataplot[[i]], vars=TRUE, CI=TRUE)
  obj[[i]]<-setMap(contMap(ASRtree, dataplot[[i]]),
                   colors=rev(brewer.pal(10,"Spectral")))
}  
 
 Plots for three traits (all plots generated at once) 
 
  for (i in 1:3) {
  plot(obj[[i]])
  title(paste('Ancestral state reconstruction for', colnames(ASRdata)[i]))
}  
     
  fit # Ancestral state values for each trait, if you need them  
  ## [[1]]
## Ancestral character estimates using fastAnc:
##       21       22       23       24       25       26       27       28 
## 6.548221 6.565757 6.593316 6.593490 6.449877 7.171073 5.760483 6.615779 
##       29       30       31       32       33       34       35       36 
## 6.793186 6.872677 6.635189 7.145014 6.966374 7.776454 7.720713 8.160978 
##       37       38       39 
## 6.046038 5.972085 6.024379 
## 
## Variances on ancestral states:
##       21       22       23       24       25       26       27       28 
## 0.420476 0.413568 0.353518 0.341939 0.488964 0.235461 0.430740 0.356544 
##       29       30       31       32       33       34       35       36 
## 0.466271 0.117240 0.416472 0.459574 0.003649 0.019967 0.017609 0.004449 
##       37       38       39 
## 0.190728 0.070797 0.095487 
## 
## Lower & upper 95% CIs:
##       lower    upper
## 21 5.277276 7.819167
## 22 5.305295 7.826219
## 23 5.427951 7.758681
## 24 5.447369 7.739610
## 25 5.079327 7.820426
## 26 6.219996 8.122150
## 27 4.474120 7.046847
## 28 5.445438 7.786120
## 29 5.454818 8.131553
## 30 6.201566 7.543789
## 31 5.370310 7.900067
## 32 5.816293 8.473736
## 33 6.847984 7.084764
## 34 7.499498 8.053409
## 35 7.460621 7.980804
## 36 8.030243 8.291712
## 37 5.190059 6.902017
## 38 5.450575 6.493595
## 39 5.418720 6.630037
## 
## 
## [[2]]
## Ancestral character estimates using fastAnc:
##       21       22       23       24       25       26       27       28 
## 5.731643 5.773769 5.980870 6.007569 5.825132 7.709274 4.337564 6.078319 
##       29       30       31       32       33       34       35       36 
## 6.262130 6.373694 6.190590 7.145900 6.645238 8.488262 8.361170 9.361035 
##       37       38       39 
## 5.892685 5.848837 5.886971 
## 
## Variances on ancestral states:
##       21       22       23       24       25       26       27       28 
## 2.083585 2.049354 1.751787 1.694408 2.422963 1.166780 2.134445 1.766780 
##       29       30       31       32       33       34       35       36 
## 2.310512 0.580961 2.063741 2.277327 0.018079 0.098941 0.087259 0.022046 
##       37       38       39 
## 0.945113 0.350819 0.473166 
## 
## Lower & upper 95% CIs:
##       lower     upper
## 21 2.902456  8.560830
## 22 2.967918  8.579620
## 23 3.386710  8.575030
## 24 3.456248  8.558890
## 25 2.774222  8.876043
## 26 5.592129  9.826418
## 27 1.474055  7.201073
## 28 3.473082  8.683556
## 29 3.282858  9.241402
## 30 4.879767  7.867622
## 31 3.374908  9.006273
## 32 4.188101 10.103700
## 33 6.381696  6.908779
## 34 7.871746  9.104779
## 35 7.782194  8.940147
## 36 9.070014  9.652056
## 37 3.987233  7.798137
## 38 4.687929  7.009744
## 39 4.538745  7.235197
## 
## 
## [[3]]
## Ancestral character estimates using fastAnc:
##        21        22        23        24        25        26        27        28 
##  8.906345  8.973149  9.274003  9.308257  9.139811 11.270081  7.519432  9.388970 
##        29        30        31        32        33        34        35        36 
## 10.175071 10.534839  9.440068 10.886562 10.494431 12.568060 12.358852 13.983273 
##        37        38        39 
##  7.726317  7.440873  7.720386 
## 
## Variances on ancestral states:
##       21       22       23       24       25       26       27       28 
## 4.157174 4.088877 3.495170 3.380688 4.834302 2.327962 4.258651 3.525083 
##       29       30       31       32       33       34       35       36 
## 4.609940 1.159135 4.117581 4.543728 0.036072 0.197408 0.174099 0.043987 
##       37       38       39 
## 1.885692 0.699955 0.944062 
## 
## Lower & upper 95% CIs:
##        lower     upper
## 21  4.910072 12.902619
## 22  5.009839 12.936460
## 23  5.609710 12.938297
## 24  5.704474 12.912040
## 25  4.830350 13.449272
## 26  8.279580 14.260582
## 27  3.474679 11.564186
## 28  5.709030 13.068911
## 29  5.966800 14.383342
## 30  8.424641 12.645036
## 31  5.462870 13.417265
## 32  6.708622 15.064503
## 33 10.122175 10.866688
## 34 11.697220 13.438901
## 35 11.541038 13.176665
## 36 13.572201 14.394345
## 37  5.034835 10.417799
## 38  5.801073  9.080674
## 39  5.815994  9.624777  
  # WARNING: All values are log-converted (ln)  
 All three plots look very similar. The biggest discrepancy between
them is the values for kiwi, which eggshell thickness ASR matches that
of body mass but not that of egg mass, due to them laying very large
eggs for their body size. Overall,  there appears to be an
increase in eggshell thickness in Aepyornithiformes , but
 it is much more pronounced in   Aepyornis  than in
 Mullerornis . 
 
  If you want the same plots with ancestral state values
mapped on the tree:  
 
  for (i in 1:3) {
  plot(obj[[i]], offset=3)
  nodelabels(as.factor(round(exp(fit[[i]]$ace), digits=0)), bg="white", cex=0.6, font=2)
  tiplabels(as.factor(round(exp(ASRdata[,i]), digits=0)), bg="white", cex=0.6, font=2, offset=8)
  title(paste('Ancestral state reconstruction for', colnames(ASRdata)[i]))
}  
     
  # The color scale on the tree was generated with log-converted values, but the values displayed on these plots for both tips and nodes are the raw values.  
 I did not generate ancestral states on this sample for relative
eggshell thickness (i.e. ratios of eggshell thickness over either egg
mass or body mass), because we are interested in trends for the elephant
bird isolated eggshells. Since I estimated the egg mass and body mass of
these taxa from their eggshell thickness, compiling such ratios for
these would be circular, and therefore meaningless. 
 However, we can generate such ancestral states for the ratio
(eggshell thickness/body mass) with only  Aepyornis hildebrandti 
and n07AEP06 (assumed to be  Mullerornis agilis ) among elephant
birds, since body mass estimates for these two taxa come from Crouch and
Clarke (2019), and not from our data. 
 
 
 For the ratio (eggshell thickness/body mass) 
  # Prepare the data and tree
ASREBM<-ASRdata[-c(13:15,17),]
ASREBM[13,3]<-10.89776 # change the value for n07AEP06 to the Crouch & Clarke estimate
dataplot<-ASREBM[,1]/ASREBM[,3]
names(dataplot)<-rownames(ASREBM)
treeplot<-drop.tip(ASRtree, setdiff(ASRtree$tip.label, rownames(ASREBM)))

# Phylogenetic signal
phylosig(treeplot, dataplot, method="lambda", test=TRUE)  
  ## 
## Phylogenetic signal lambda : 0.963456 
## logL(lambda) : 21.4522 
## LR(lambda=0) : 9.68686 
## P-value (based on LR test) : 0.00185591  
  # Check for best evolutionary model
fit<-list()
for (m in 1:length(models)) {
  fit[[m]]=fitContinuous(treeplot, dataplot, model=models[m], ncores=2)
}
modSel.geiger(fit[[1]],fit[[2]],fit[[3]],fit[[4]],fit[[5]],fit[[6]]) # BM is the best fit  
 
 
 
  # Prepare the plot
fitEBM<-fastAnc(treeplot, dataplot, vars=TRUE, CI=TRUE)
obj<-contMap(treeplot, dataplot)  
 
 Plot 
 
  plot(setMap(obj, colors=rev(brewer.pal(10,"Spectral"))));
title('Ancestral state reconstruction for (eggshell thickness/body mass)')  
   
  # Add ancestral state values for the nodes on the tree  (and values for the tips)
plot(setMap(obj, colors=rev(brewer.pal(10,"Spectral"))), offset=2); nodelabels(as.factor(round(exp(fitEBM$ace), digits=3)), bg="white", cex=0.6, font=2); tiplabels(as.factor(round(exp(ASREBM[,1]/ASREBM[,3]), digits=3)), bg="white", cex=0.6, font=2, offset=6);
title('Ancestral state reconstruction for (eggshell thickness/body mass)')  
   
  # The color scale on the tree was generated with log-converted values, but the values displayed on these plots for both tips and nodes are the raw values.

fitEBM # Ancestral state values for each trait, if you need them  
  ## Ancestral character estimates using fastAnc:
##       17       18       19       20       21       22       23       24 
## 0.755839 0.752390 0.731415 0.729036 0.728233 0.641309 0.781866 0.725348 
##       25       26       27       28       29       30       31 
## 0.675067 0.653271 0.724931 0.682217 0.787136 0.804529 0.781644 
## 
## Variances on ancestral states:
##       17       18       19       20       21       22       23       24 
## 0.001205 0.001185 0.001014 0.000980 0.001401 0.000675 0.001234 0.001023 
##       25       26       27       28       29       30       31 
## 0.001336 0.000336 0.001195 0.001334 0.000547 0.000203 0.000274 
## 
## Lower & upper 95% CIs:
##       lower    upper
## 17 0.687796 0.823883
## 18 0.684908 0.819873
## 19 0.669015 0.793814
## 20 0.667664 0.790408
## 21 0.654858 0.801607
## 22 0.590395 0.692223
## 23 0.713002 0.850729
## 24 0.662674 0.788023
## 25 0.603418 0.746716
## 26 0.617344 0.689198
## 27 0.657179 0.792684
## 28 0.610635 0.753799
## 29 0.741312 0.832960
## 30 0.776611 0.832447
## 31 0.749221 0.814067  
  # WARNING: All values are log-converted (ln).  
 The two elephant birds have values similar to those of cassowaries,
emu, rhea and ostrich – their very thick eggshells are “compensated” by
their large body size. There is, however, a visible difference between
 Aepyornis  and  Mullerornis , since their difference in
eggshell thickness is much more pronounced than their difference in body
mass. Conversely, both moa show a relatively thin eggshell for such
large animals. Smaller taxa (kiwi, tinamous,  Lithornis ) show high
values due to their small size. 
 
 
 For the ratio (egg mass/body mass) 
  dataplot<-(ASREBM[,2]/ASREBM[,3])
names(dataplot)<-rownames(ASREBM)
treeplot<-drop.tip(ASRtree, setdiff(ASRtree$tip.label, rownames(ASREBM)))

# Phylogenetic signal
phylosig(treeplot, dataplot, method="lambda", test=TRUE)  
  ## 
## Phylogenetic signal lambda : 0.999934 
## logL(lambda) : 24.9419 
## LR(lambda=0) : 14.5992 
## P-value (based on LR test) : 0.00013297  
  # Check for best evolutionary model
fit<-list()
for (m in 1:length(models)) {
  fit[[m]]=fitContinuous(treeplot, dataplot, model=models[m], ncores=2)
}
modSel.geiger(fit[[1]],fit[[2]],fit[[3]],fit[[4]],fit[[5]],fit[[6]]) # BM is the best fit  
 
 
 
  # Prepare the plot
fitEBM<-fastAnc(treeplot, dataplot, vars=TRUE, CI=TRUE)
obj<-contMap(treeplot, dataplot)  
 
 Plot 
 
  plot(setMap(obj, colors=rev(brewer.pal(10,"Spectral"))));
title('Ancestral state reconstruction for (egg mass/body mass)')  
   
  # Add ancestral state values for the nodes on the tree (and values for the tips)
plot(setMap(obj, colors=rev(brewer.pal(10,"Spectral"))), offset=2); nodelabels(as.factor(round(exp(fitEBM$ace), digits=3)), bg="white", cex=0.6, font=2); tiplabels(as.factor(round(exp(ASREBM[,2]/ASREBM[,3]), digits=3)), bg="white", cex=0.6, font=2, offset=5);
title('Ancestral state reconstruction for (egg mass/body mass)')  
   
  # The color scale on the tree was generated with log-converted values, but the values displayed on these plots for both tips and nodes are the raw values.

fitEBM # Ancestral state values for each trait, if you need them  
  ## Ancestral character estimates using fastAnc:
##       17       18       19       20       21       22       23       24 
## 0.644958 0.644899 0.646361 0.646878 0.628646 0.681776 0.565415 0.650501 
##       25       26       27       28       29       30       31 
## 0.617434 0.605361 0.661923 0.672988 0.765964 0.786896 0.763401 
## 
## Variances on ancestral states:
##       17       18       19       20       21       22       23       24 
## 0.000750 0.000738 0.000631 0.000610 0.000873 0.000420 0.000769 0.000637 
##       25       26       27       28       29       30       31 
## 0.000832 0.000209 0.000744 0.000831 0.000340 0.000126 0.000170 
## 
## Lower & upper 95% CIs:
##       lower    upper
## 17 0.591266 0.698651
## 18 0.591649 0.698150
## 19 0.597122 0.695601
## 20 0.598450 0.695307
## 21 0.570747 0.686546
## 22 0.641600 0.721952
## 23 0.511074 0.619755
## 24 0.601045 0.699957
## 25 0.560896 0.673972
## 26 0.577012 0.633711
## 27 0.608460 0.715386
## 28 0.616503 0.729472
## 29 0.729805 0.802124
## 30 0.764866 0.808926
## 31 0.737816 0.788986  
  # WARNING: All values are log-converted (ln).  
 All major paleognath clades (ostrich, rhea, moa + tinamous,
cassowaries + emu) show a decrease in relative egg mass compared to the
value at the previous node, except the clade formed by kiwi and elephant
birds, which show an increase. This is likely due to the very high
relative egg mass found in all kiwi. Moa also present a strong increase
in relative egg mass.  This seems to support the hypothesis that
the kiwi body mass decreased faster than their egg mass, and that they
present an enlarged egg, possibly inherited from a larger ancestor prior
to their miniaturization, and likely associated with extreme k life
history strategy  (e.g. Calder, 1979; Hume and Robertson,
2021). 
 
 
 Ancestral reconstructions with the tree generated for this
paper 
 
 For eggshell thickness, egg mass, and body mass 
 The sample for these analyses matches the tree built in the present
study, and is thus slightly different from the previous ancestral
reconstructions; three species were removed ( R. rufescens ,  L.
plebius , and  A. australis ), and three were added using new
references: 
 –  Rhea pennata : shell thickness: Tyler and Simkiss (1959); egg
mass: Navarro et al. (2003); 
–  Anomalopteryx didiformis : shell thickness: Gill (2021); egg
mass: Gill (2006); 
–  Tinamus major : both measurements taken from Juang et
al. (2017). 
 
 All body mass measurements taken from Dunning (2008); body mass
estimate for  A. didiformis  taken from Crouch and Clarke
(2019). 
 
 Prepare the plots 
 
  # Prepare the data and tree
ASRdatanew<-read.table("ASR_newdata.txt", header=TRUE)
rownames(ASRdatanew)<-ASRdatanew$Species
ASRtreenew<-read.nexus("MOLDAT_APR19_NEW.trees.nex")
plotTree(ASRtreenew); ASRtreenew<-force.ultrametric(ASRtreenew)  
   
  ## ***************************************************************
## *                          Note:                              *
## *    force.ultrametric does not include a formal method to    *
## *    ultrametricize a tree & should only be used to coerce    *
## *   a phylogeny that fails is.ultramtric due to rounding --   *
## *    not as a substitute for formal rate-smoothing methods.   *
## ***************************************************************  
  setdiff(ASRtreenew$tip.label,ASRdatanew$Species)  
  ## character(0)  
  # Log-conversion and adding data for elephant bird eggshell fragments
ASRdatanew[,c(2:4)]<-log(ASRdatanew[,c(2:4)])
ASRdatanew[c(10:14),3]<-ASRdata[c(13:17),3]; ASRdatanew[c(9:14),4]<-ASRdata[c(12:17),2]
ASRdatanew<-ASRdatanew[,-1]
ASRdatanew<-ReorderData(ASRtreenew, ASRdatanew)

# Phylogenetic signal (Pagel's lambda) of each variable
var=list(); phy=list()
for (i in 1:3) {
  var<-ASRdatanew[,i]; names(var)<-rownames(ASRdatanew)
  phy[[i]]<-phylosig(ASRtreenew, var, method="lambda", test=T)
}
phy  
  ## [[1]]
## 
## Phylogenetic signal lambda : 0.896577 
## logL(lambda) : -16.4416 
## LR(lambda=0) : 14.0349 
## P-value (based on LR test) : 0.000179447 
## 
## 
## [[2]]
## 
## Phylogenetic signal lambda : 0.81573 
## logL(lambda) : -37.6052 
## LR(lambda=0) : 11.6577 
## P-value (based on LR test) : 0.000639383 
## 
## 
## [[3]]
## 
## Phylogenetic signal lambda : 0.850136 
## logL(lambda) : -30.5371 
## LR(lambda=0) : 11.6364 
## P-value (based on LR test) : 0.000646737  
  # Check best evolutionary model
models=c("BM", "OU", "EB", "rate_trend","lambda", "white")
# If you need more information on each tested model, check '?fitContinuous')

var=list(); fit<-list(); mod<-list()
for (i in 1:ncol(ASRdatanew)) {
  var<-ASRdatanew[,i]; names(var)<-rownames(ASRdatanew)
  for (m in 1:length(models)) {
    fit[[m]]=fitContinuous(ASRtreenew, var, model=models[m], ncores=2)
  }
  mod[[i]]<-modSel.geiger(fit[[1]],fit[[2]],fit[[3]],fit[[4]],fit[[5]],fit[[6]])
}  
  ## Warning in matrix(unlist(children), nrow = 2): data length [37] is not a sub-
## multiple or multiple of the number of rows [2]

## Warning in matrix(unlist(children), nrow = 2): data length [37] is not a sub-
## multiple or multiple of the number of rows [2]

## Warning in matrix(unlist(children), nrow = 2): data length [37] is not a sub-
## multiple or multiple of the number of rows [2]

## Warning in matrix(unlist(children), nrow = 2): data length [37] is not a sub-
## multiple or multiple of the number of rows [2]  
  ## Warning in fitContinuous(ASRtreenew, var, model = models[m], ncores = 2): 
## Parameter estimates appear at bounds:
##  slope  
  ## Warning in matrix(unlist(children), nrow = 2): data length [37] is not a sub-
## multiple or multiple of the number of rows [2]

## Warning in matrix(unlist(children), nrow = 2): data length [37] is not a sub-
## multiple or multiple of the number of rows [2]

## Warning in matrix(unlist(children), nrow = 2): data length [37] is not a sub-
## multiple or multiple of the number of rows [2]

## Warning in matrix(unlist(children), nrow = 2): data length [37] is not a sub-
## multiple or multiple of the number of rows [2]

## Warning in matrix(unlist(children), nrow = 2): data length [37] is not a sub-
## multiple or multiple of the number of rows [2]

## Warning in matrix(unlist(children), nrow = 2): data length [37] is not a sub-
## multiple or multiple of the number of rows [2]

## Warning in matrix(unlist(children), nrow = 2): data length [37] is not a sub-
## multiple or multiple of the number of rows [2]

## Warning in matrix(unlist(children), nrow = 2): data length [37] is not a sub-
## multiple or multiple of the number of rows [2]

## Warning in matrix(unlist(children), nrow = 2): data length [37] is not a sub-
## multiple or multiple of the number of rows [2]

## Warning in matrix(unlist(children), nrow = 2): data length [37] is not a sub-
## multiple or multiple of the number of rows [2]

## Warning in matrix(unlist(children), nrow = 2): data length [37] is not a sub-
## multiple or multiple of the number of rows [2]

## Warning in matrix(unlist(children), nrow = 2): data length [37] is not a sub-
## multiple or multiple of the number of rows [2]  
  ## Warning in fitContinuous(ASRtreenew, var, model = models[m], ncores = 2): 
## Parameter estimates appear at bounds:
##  slope  
  ## Warning in matrix(unlist(children), nrow = 2): data length [37] is not a sub-
## multiple or multiple of the number of rows [2]

## Warning in matrix(unlist(children), nrow = 2): data length [37] is not a sub-
## multiple or multiple of the number of rows [2]  
  mod # Lambda model is the best fit in all traits  
  ## [[1]]
##          K    logLik     AICc deltaAICc Weight Evidence ratio
## fit[[2]] 3 -17.20694 41.91389  0.000000 0.6718        1.00000
## fit[[6]] 2 -19.54146 43.78880  1.874914 0.2631        2.55348
## fit[[5]] 3 -19.54146 46.58292  4.669032 0.0651       10.32446
## fit[[4]] 3 -27.66781 62.83563 20.921744 0.0000    34921.98509
## fit[[1]] 2 -30.61776 65.94140 24.027519 0.0000   165009.69748
## fit[[3]] 3 -30.61791 68.73582 26.821931 0.0000   667280.41208
## 
## [[2]]
##          K    logLik      AICc deltaAICc Weight Evidence ratio
## fit[[2]] 3 -33.11765  73.73529  0.000000 0.7234   1.000000e+00
## fit[[6]] 2 -35.69689  76.09965  2.364363 0.2218   3.261481e+00
## fit[[5]] 3 -35.69689  78.89377  5.158480 0.0549   1.318711e+01
## fit[[4]] 3 -44.79393  97.08785 23.352562 0.0000   1.177455e+05
## fit[[1]] 2 -47.80827 100.32243 26.587138 0.0000   5.933675e+05
## fit[[3]] 3 -47.80842 103.11685 29.381556 0.0000   2.399516e+06
## 
## [[3]]
##          K    logLik     AICc deltaAICc Weight Evidence ratio
## fit[[2]] 3 -27.92321 63.34642  0.000000 0.6621   1.000000e+00
## fit[[6]] 2 -30.23458 65.17505  1.828627 0.2654   2.495061e+00
## fit[[5]] 3 -30.13473 67.76946  4.423038 0.0725   9.129574e+00
## fit[[4]] 3 -40.44645 88.39291 25.046490 0.0000   2.746479e+05
## fit[[1]] 2 -43.52042 91.74672 28.400301 0.0000   1.469086e+06
## fit[[3]] 3 -43.52057 94.54114 31.194723 0.0000   5.940843e+06  
  # Plots
dataplot=list(); fit=list(); obj=list()
for (i in 1:ncol(ASRdatanew)) {
  dataplot[[i]]<-as.matrix(ASRdatanew)[,i]
  fit[[i]]<-fastAnc(ASRtreenew, dataplot[[i]], vars=TRUE, CI=TRUE)
  obj[[i]]<-setMap(contMap(ASRtreenew, dataplot[[i]]),
                   colors=rev(brewer.pal(10,"Spectral")))
}  
     
 
 Plots for three traits (all plots generated at once) 
 
  for (i in 1:3) {
  plot(obj[[i]])
  title(paste('Ancestral state reconstruction for', colnames(ASRdatanew)[i]))
}  
     
  fit # Ancestral state values for each trait, if you need them  
  ## [[1]]
## Ancestral character estimates using fastAnc:
##       21       22       23       24       25       26       27       28 
## 6.750645 6.613750 7.201676 7.012343 6.043464 6.762840 6.806468 6.765219 
##       29       30       31       32       33       34       35       36 
## 6.840194 6.876008 6.762587 6.111918 5.975426 7.163084 6.966427 7.776756 
##       37       38 
## 8.161004 7.720945 
## 
## Variances on ancestral states:
##       21       22       23       24       25       26       27       28 
## 0.406860 0.463060 0.207421 0.079293 0.517146 0.366106 0.239561 0.363016 
##       29       30       31       32       33       34       35       36 
## 0.403293 0.129920 0.389459 0.265789 0.067969 0.439346 0.003604 0.019718 
##       37       38 
## 0.004395 0.017391 
## 
## Lower & upper 95% CIs:
##       lower    upper
## 21 5.500449 8.000842
## 22 5.279999 7.947501
## 23 6.309022 8.094329
## 24 6.460426 7.564260
## 25 4.633972 7.452956
## 26 5.576908 7.948771
## 27 5.847146 7.765790
## 28 5.584303 7.946134
## 29 5.595490 8.084898
## 30 6.169538 7.582478
## 31 5.539416 7.985758
## 32 5.101445 7.122391
## 33 5.464437 6.486415
## 34 5.863933 8.462234
## 35 6.848767 7.084087
## 36 7.501530 8.051981
## 37 8.031074 8.290935
## 38 7.462470 7.979421
## 
## 
## [[2]]
## Ancestral character estimates using fastAnc:
##        21        22        23        24        25        26        27        28 
##  9.852418  9.569567 11.339310 11.098766  8.159354  9.896061 10.042834  9.904907 
##        29        30        31        32        33        34        35        36 
## 10.342371 10.543297  9.881222  7.879191  7.440822 10.966013 10.494665 12.569388 
##        37        38 
## 13.983391 12.359874 
## 
## Variances on ancestral states:
##       21       22       23       24       25       26       27       28 
## 3.986882 4.537597 2.032554 0.777005 5.067593 3.587531 2.347500 3.557250 
##       29       30       31       32       33       34       35       36 
## 3.951927 1.273103 3.816374 2.604510 0.666039 4.305222 0.035313 0.193221 
##       37       38 
## 0.043063 0.170418 
## 
## Lower & upper 95% CIs:
##        lower     upper
## 21  5.938851 13.765985
## 22  5.394446 13.744688
## 23  8.544984 14.133636
## 24  9.371067 12.826466
## 25  3.747136 12.571571
## 26  6.183669 13.608454
## 27  7.039810 13.045858
## 28  6.208215 13.601599
## 29  6.445998 14.238744
## 30  8.331793 12.754802
## 31  6.052255 13.710188
## 32  4.716046 11.042336
## 33  5.841242  9.040401
## 34  6.899203 15.032822
## 35 10.126346 10.862985
## 36 11.707834 13.430943
## 37 13.576661 14.390120
## 38 11.550753 13.168995
## 
## 
## [[3]]
## Ancestral character estimates using fastAnc:
##       21       22       23       24       25       26       27       28 
## 6.308560 6.083312 7.642412 7.460257 4.897711 6.377480 6.373226 6.399820 
##       29       30       31       32       33       34       35       36 
## 6.358116 6.382737 6.440442 5.891473 5.845676 7.183696 6.645349 8.488894 
##       37       38 
## 9.361091 8.361657 
## 
## Variances on ancestral states:
##       21       22       23       24       25       26       27       28 
## 2.014990 2.293324 1.027263 0.392702 2.561187 1.813156 1.186438 1.797852 
##       29       30       31       32       33       34       35       36 
## 1.997323 0.643433 1.928815 1.316332 0.336619 2.175881 0.017847 0.097655 
##       37       38 
## 0.021764 0.086130 
## 
## Lower & upper 95% CIs:
##       lower     upper
## 21 3.526334  9.090787
## 22 3.115142  9.051483
## 23 5.655874  9.628950
## 24 6.232004  8.688510
## 25 1.760985  8.034438
## 26 3.738272  9.016688
## 27 4.238320  8.508131
## 28 3.771773  9.027866
## 29 3.588112  9.128119
## 30 4.810538  7.954936
## 31 3.718359  9.162525
## 32 3.642735  8.140210
## 33 4.708505  6.982846
## 34 4.292527 10.074866
## 35 6.383504  6.907194
## 36 7.876399  9.101389
## 37 9.071940  9.650243
## 38 7.786438  8.936876  
  # WARNING: All values are log-converted (ln)  
 All three plots look very similar. The biggest discrepancy between
them is the values for kiwi, which eggshell thickness ASR matches that
of body mass but not that of egg mass, due to them laying very large
eggs for their body size. Overall,  there appears to be an
increase in eggshell thickness in Aepyornithiformes , but
 it is much more pronounced in   Aepyornis  than in
 Mullerornis . 
 
  If you want the same plots with ancestral state values
mapped on the tree:  
 
  for (i in 1:3) {
  plot(obj[[i]], offset=3)
  nodelabels(as.factor(round(exp(fit[[i]]$ace), digits=0)), bg="white", cex=0.6, font=2)
  tiplabels(as.factor(round(exp(ASRdatanew[,i]), digits=0)), bg="white", cex=0.6, font=2, offset=8)
  title(paste('Ancestral state reconstruction for', colnames(ASRdatanew)[i]))
}  
     
  # The color scale on the tree was generated with log-converted values, but the values displayed on these plots for both tips and nodes are the raw values.  
 I did not generate ancestral states on this sample for relative
eggshell thickness (i.e. ratios of eggshell thickness over either egg
mass or body mass), because we are interested in trends for the elephant
bird isolated eggshells. Since I estimated the egg mass and body mass of
these taxa from their eggshell thickness, compiling such ratios for
these would be circular, and therefore meaningless. 
 However, we can generate such ancestral states for the ratio
(eggshell thickness/body mass) with only  Aepyornis hildebrandti 
and n07AEP06 (assumed to be  Mullerornis agilis ) among elephant
birds, since body mass estimates for these two taxa come from Crouch and
Clarke (2019), and not from our data. 
 
 
 Same reconstructions with neognaths added to the sample 
 
 For eggshell thickness, egg mass, and body mass 
 
  # Prepare the data and tree
neodata<-cbind(plotdataEM,plotdataBM[,4]); rownames(neodata)<-rownames(dataAG)
dataneo<-neodata %>% filter(Clade=="Neognaths"); dataneo<-dataneo[,c(3,5,4)]
names(dataneo)[2]<-"Bodymass"; dataneo<-log(dataneo)
AllASRdata<-rbind(dataneo,ASRdatanew)

AllASRtree<-read.nexus("Newwholetree.trees.nex")
setdiff(AllASRtree$tip.label,rownames(AllASRdata))  
  ## character(0)  
  AllASRtree<-force.ultrametric(AllASRtree)  
  ## ***************************************************************
## *                          Note:                              *
## *    force.ultrametric does not include a formal method to    *
## *    ultrametricize a tree & should only be used to coerce    *
## *   a phylogeny that fails is.ultramtric due to rounding --   *
## *    not as a substitute for formal rate-smoothing methods.   *
## ***************************************************************  
  # Phylogenetic signal (Pagel's lambda) of each variable
var=list(); phy=list()
for (i in 1:3) {
  var<-AllASRdata[,i]; names(var)<-rownames(AllASRdata)
  phy[[i]]<-phylosig(AllASRtree, var, method="lambda", test=T)
}
phy  
  ## [[1]]
## 
## Phylogenetic signal lambda : 0.92573 
## logL(lambda) : -54.2194 
## LR(lambda=0) : 81.1626 
## P-value (based on LR test) : 2.07889e-19 
## 
## 
## [[2]]
## 
## Phylogenetic signal lambda : 0.887613 
## logL(lambda) : -123.387 
## LR(lambda=0) : 79.9049 
## P-value (based on LR test) : 3.92873e-19 
## 
## 
## [[3]]
## 
## Phylogenetic signal lambda : 0.91549 
## logL(lambda) : -103.718 
## LR(lambda=0) : 91.6324 
## P-value (based on LR test) : 1.0437e-21  
  # Check best evolutionary model
var=list(); fit<-list(); mod<-list()
for (i in 1:ncol(AllASRdata)) {
  var<-AllASRdata[,i]; names(var)<-rownames(AllASRdata)
  for (m in 1:length(models)) {
    fit[[m]]=fitContinuous(AllASRtree, var, model=models[m], ncores=2)
  }
  mod[[i]]<-modSel.geiger(fit[[1]],fit[[2]],fit[[3]],fit[[4]],fit[[5]],fit[[6]])
}
mod # Lambda model is the best fit in all traits  
  ## [[1]]
##          K    logLik      AICc deltaAICc Weight Evidence ratio
## fit[[5]] 3 -44.73601  95.86547  0.000000 0.9532   1.000000e+00
## fit[[2]] 3 -48.09898 102.59141  6.725942 0.0330   2.887485e+01
## fit[[6]] 2 -50.07536 104.34426  8.478796 0.0137   6.936608e+01
## fit[[4]] 3 -64.73454 135.86252 39.997051 0.0000   4.844503e+08
## fit[[1]] 2 -66.40680 137.00715 41.141682 0.0000   8.586235e+08
## fit[[3]] 3 -66.40709 139.20763 43.342160 0.0000   2.580065e+09
## 
## [[2]]
##          K     logLik     AICc deltaAICc Weight Evidence ratio
## fit[[5]] 3  -82.45674 171.3069  0.000000 0.9768   1.000000e+00
## fit[[2]] 3  -86.58181 179.5571  8.250142 0.0158   6.187220e+01
## fit[[6]] 2  -88.44253 181.0786  9.771688 0.0074   1.324022e+02
## fit[[4]] 3 -101.78159 209.9566 38.649711 0.0000   2.469893e+08
## fit[[1]] 2 -103.16526 210.5241 39.217144 0.0000   3.280156e+08
## fit[[3]] 3 -103.16553 212.7245 41.417574 0.0000   9.856255e+08
## 
## [[3]]
##          K    logLik     AICc deltaAICc Weight Evidence ratio
## fit[[5]] 3 -72.95166 152.2968   0.00000 0.9928   1.000000e+00
## fit[[2]] 3 -78.26770 162.9288  10.63207 0.0049   2.035753e+02
## fit[[6]] 2 -80.12648 164.4465  12.14974 0.0023   4.347937e+02
## fit[[4]] 3 -93.67961 193.7527  41.45590 0.0000   1.004693e+09
## fit[[1]] 2 -95.50188 195.1973  42.90054 0.0000   2.068870e+09
## fit[[3]] 3 -95.50217 197.3978  45.10103 0.0000   6.216755e+09  
  # Plots
dataplot=list(); fit=list(); obj=list()
for (i in 1:ncol(AllASRdata)) {
  dataplot[[i]]<-as.matrix(AllASRdata)[,i]
  fit[[i]]<-fastAnc(AllASRtree, dataplot[[i]], vars=TRUE, CI=TRUE)
  obj[[i]]<-setMap(contMap(AllASRtree, dataplot[[i]]),
                   colors=rev(brewer.pal(10,"Spectral")))
}  
     
 
 Plots for three traits (all plots generated at once) 
 
  for (i in 1:3) {
  plot(obj[[i]])
  title(paste('Ancestral state reconstruction for', colnames(AllASRdata)[i]))
}  
     
  fit # Ancestral state values for each trait, if you need them  
  ## [[1]]
## Ancestral character estimates using fastAnc:
##       66       67       68       69       70       71       72       73 
## 6.255315 6.530365 6.458325 5.965302 7.185168 7.008789 6.596607 6.786878 
##       74       75       76       77       78       79       80       81 
## 6.614932 6.797950 6.869859 6.629226 6.091649 5.972838 7.115923 6.966288 
##       82       83       84       85       86       87       88       89 
## 7.775967 7.720338 8.160934 5.557426 5.714968 5.845869 5.705727 5.673080 
##       90       91       92       93       94       95       96       97 
## 5.412393 5.389555 5.184701 5.119351 5.084014 5.441469 5.460655 5.468435 
##       98       99      100      101      102      103      104      105 
## 5.456592 5.450426 5.445644 5.286086 5.382451 5.354046 5.343463 5.352437 
##      106      107      108      109      110      111      112      113 
## 5.334253 5.309058 5.528485 5.670426 5.760129 5.536670 5.551994 5.495014 
##      114      115      116      117      118      119      120      121 
## 5.027594 5.121543 5.001661 4.977699 4.856779 4.673121 4.608570 4.778815 
##      122      123      124      125      126      127      128 
## 4.698041 4.303309 4.641436 4.598155 4.589573 4.642025 4.619708 
## 
## Variances on ancestral states:
##       66       67       68       69       70       71       72       73 
## 0.347943 0.148232 0.165671 0.186222 0.074817 0.028607 0.133367 0.086445 
##       74       75       76       77       78       79       80       81 
## 0.132018 0.145581 0.046874 0.141335 0.095909 0.024522 0.158609 0.001300 
##       82       83       84       85       86       87       88       89 
## 0.007114 0.006274 0.001585 0.117240 0.157382 0.198258 0.188013 0.153698 
##       90       91       92       93       94       95       96       97 
## 0.080307 0.084466 0.130482 0.142287 0.161516 0.091859 0.094274 0.100571 
##       98       99      100      101      102      103      104      105 
## 0.108224 0.118402 0.137947 0.069466 0.071951 0.080484 0.099845 0.133806 
##      106      107      108      109      110      111      112      113 
## 0.099920 0.121743 0.090130 0.115983 0.142452 0.127005 0.137028 0.143005 
##      114      115      116      117      118      119      120      121 
## 0.073239 0.146971 0.073156 0.073853 0.077542 0.131152 0.146710 0.085235 
##      122      123      124      125      126      127      128 
## 0.090117 0.090392 0.095112 0.120409 0.110912 0.110855 0.118579 
## 
## Lower & upper 95% CIs:
##        lower    upper
## 66  5.099177 7.411454
## 67  5.775748 7.284982
## 68  5.660552 7.256097
## 69  5.119494 6.811110
## 70  6.649055 7.721280
## 71  6.677285 7.340293
## 72  5.880825 7.312389
## 73  6.210606 7.363149
## 74  5.902781 7.327083
## 75  6.050110 7.545790
## 76  6.445513 7.294205
## 77  5.892374 7.366078
## 78  5.484652 6.698646
## 79  5.665913 6.279764
## 80  6.335339 7.896508
## 81  6.895616 7.036960
## 82  7.610654 7.941281
## 83  7.565086 7.875591
## 84  8.082892 8.238977
## 85  4.886316 6.228536
## 86  4.937408 6.492528
## 87  4.973156 6.718582
## 88  4.855862 6.555592
## 89  4.904676 6.441485
## 90  4.856958 5.967828
## 91  4.819920 5.959190
## 92  4.476704 5.892698
## 93  4.380019 5.858683
## 94  4.296307 5.871720
## 95  4.847426 6.035512
## 96  4.858855 6.062454
## 97  4.846860 6.090009
## 98  4.811804 6.101381
## 99  4.775998 6.124854
## 100 4.717675 6.173613
## 101 4.769499 5.802673
## 102 4.856709 5.908194
## 103 4.798000 5.910092
## 104 4.724138 5.962789
## 105 4.635479 6.069396
## 106 4.714696 5.953811
## 107 4.625182 5.992935
## 108 4.940060 6.116909
## 109 5.002924 6.337928
## 110 5.020371 6.499888
## 111 4.838171 6.235169
## 112 4.826455 6.277533
## 113 4.753821 6.236207
## 114 4.497164 5.558025
## 115 4.370143 5.872943
## 116 4.471533 5.531788
## 117 4.445050 5.510348
## 118 4.310989 5.402569
## 119 3.963307 5.382934
## 120 3.857836 5.359304
## 121 4.206593 5.351038
## 122 4.109657 5.286424
## 123 3.714032 4.892587
## 124 4.036967 5.245905
## 125 3.918036 5.278274
## 126 3.936826 5.242320
## 127 3.989444 5.294606
## 128 3.944776 5.294641
## 
## 
## [[2]]
## Ancestral character estimates using fastAnc:
##        66        67        68        69        70        71        72        73 
##  8.239009  9.334196  9.201044  7.974028 11.300169 11.090339  9.503316  9.996550 
##        74        75        76        77        78        79        80        81 
##  9.549838 10.242565 10.528770  9.566141  7.831305  7.434707 10.854589 10.494337 
##        82        83        84        85        86        87        88        89 
## 12.567526 12.358440 13.983225  6.289072  6.666110  6.920938  6.711234  6.645839 
##        90        91        92        93        94        95        96        97 
##  5.907156  5.947197  5.551814  5.439468  5.359037  6.117495  6.185351  6.239668 
##        98        99       100       101       102       103       104       105 
##  6.261066  6.264760  6.297778  5.445414  5.650140  5.528955  5.488618  5.506518 
##       106       107       108       109       110       111       112       113 
##  5.439565  5.339716  6.069896  6.436796  6.692303  6.175608  6.286317  6.021631 
##       114       115       116       117       118       119       120       121 
##  4.681754  4.799402  4.613297  4.554691  4.284270  3.818464  3.630054  4.124310 
##       122       123       124       125       126       127       128 
##  3.928090  3.151668  3.774099  3.639632  3.673142  3.831299  3.637489 
## 
## Variances on ancestral states:
##       66       67       68       69       70       71       72       73 
## 3.350037 1.427194 1.595102 1.792972 0.720347 0.275428 1.284078 0.832309 
##       74       75       76       77       78       79       80       81 
## 1.271084 1.401675 0.451305 1.360788 0.923428 0.236100 1.527109 0.012518 
##       82       83       84       85       86       87       88       89 
## 0.068493 0.060410 0.015265 1.128802 1.515297 1.908854 1.810214 1.479822 
##       90       91       92       93       94       95       96       97 
## 0.773208 0.813249 1.256299 1.369963 1.555102 0.884434 0.907681 0.968314 
##       98       99      100      101      102      103      104      105 
## 1.041993 1.139990 1.328176 0.668831 0.692749 0.774911 0.961320 1.288304 
##      106      107      108      109      110      111      112      113 
## 0.962040 1.172157 0.867784 1.116697 1.371545 1.222818 1.319324 1.376869 
##      114      115      116      117      118      119      120      121 
## 0.705158 1.415052 0.704353 0.711070 0.746588 1.262753 1.412545 0.820654 
##      122      123      124      125      126      127      128 
## 0.867662 0.870302 0.915752 1.159311 1.067873 1.067331 1.141697 
## 
## Lower & upper 95% CIs:
##         lower     upper
## 66   4.651600 11.826419
## 67   6.992677 11.675715
## 68   6.725617 11.676472
## 69   5.349551 10.598505
## 70   9.636652 12.963685
## 71  10.061707 12.118971
## 72   7.282300 11.724332
## 73   8.208423 11.784677
## 74   7.340088 11.759588
## 75   7.922075 12.563055
## 76   9.212057 11.845484
## 77   7.279745 11.852536
## 78   5.947840  9.714770
## 79   6.482341  8.387073
## 80   8.432495 13.276684
## 81  10.275047 10.713628
## 82  12.054571 13.080481
## 83  11.876704 12.840177
## 84  13.741066 14.225384
## 85   4.206668  8.371475
## 86   4.253401  9.078819
## 87   4.212977  9.628900
## 88   4.074168  9.348300
## 89   4.261540  9.030139
## 90   4.183683  7.630628
## 91   4.179663  7.714732
## 92   3.354953  7.748675
## 93   3.145378  7.733558
## 94   2.914844  7.803231
## 95   4.274225  7.960764
## 96   4.318013  8.052688
## 97   4.310970  8.168366
## 98   4.260337  8.261796
## 99   4.172062  8.357459
## 100  4.038945  8.556610
## 101  3.842484  7.048343
## 102  4.018801  7.281478
## 103  3.803586  7.254324
## 104  3.566899  7.410338
## 105  3.281849  7.731186
## 106  3.517126  7.362004
## 107  3.217698  7.461734
## 108  4.244059  7.895733
## 109  4.365588  8.508004
## 110  4.396888  8.987717
## 111  4.008218  8.342998
## 112  4.035025  8.537608
## 113  3.721766  8.321496
## 114  3.035870  6.327639
## 115  2.467865  7.130939
## 116  2.968352  6.258241
## 117  2.901922  6.207460
## 118  2.590725  5.977814
## 119  1.615967  6.020960
## 120  1.300584  5.959525
## 121  2.348747  5.899873
## 122  2.102382  5.753799
## 123  1.323185  4.980152
## 124  1.898479  5.649720
## 125  1.529274  5.749989
## 126  1.647718  5.698565
## 127  1.806390  5.856208
## 128  1.543224  5.731753
## 
## 
## [[3]]
## Ancestral character estimates using fastAnc:
##       66       67       68       69       70       71       72       73 
## 4.893916 5.937202 5.820883 4.765739 7.614539 7.454255 6.091829 6.339562 
##       74       75       76       77       78       79       80       81 
## 6.141570 6.285524 6.372171 6.211277 5.856644 5.841229 7.102656 6.645110 
##       82       83       84       85       86       87       88       89 
## 8.487540 8.360614 9.360971 3.316796 3.571156 3.852475 3.475747 3.317922 
##       90       91       92       93       94       95       96       97 
## 3.026580 3.003922 2.637924 2.524138 2.449251 3.111793 3.160142 3.205920 
##       98       99      100      101      102      103      104      105 
## 3.224294 3.249132 3.277211 2.744136 3.018753 3.008023 2.986927 3.021948 
##      106      107      108      109      110      111      112      113 
## 3.018134 2.997967 3.308522 3.591497 3.790370 3.322111 3.397859 3.282792 
##      114      115      116      117      118      119      120      121 
## 2.093446 2.239631 2.032772 1.981812 1.733142 1.342872 1.207189 1.576100 
##      122      123      124      125      126      127      128 
## 1.401053 0.579161 1.275357 1.191650 1.130422 1.215285 1.091278 
## 
## Variances on ancestral states:
##       66       67       68       69       70       71       72       73 
## 1.734202 0.738811 0.825731 0.928161 0.372900 0.142580 0.664724 0.430858 
##       74       75       76       77       78       79       80       81 
## 0.657997 0.725600 0.233626 0.704434 0.478028 0.122221 0.790533 0.006480 
##       82       83       84       85       86       87       88       89 
## 0.035457 0.031272 0.007902 0.584343 0.784419 0.988150 0.937087 0.766054 
##       90       91       92       93       94       95       96       97 
## 0.400264 0.420992 0.650344 0.709184 0.805024 0.457842 0.469876 0.501264 
##       98       99      100      101      102      103      104      105 
## 0.539405 0.590135 0.687552 0.346232 0.358613 0.401145 0.497643 0.666912 
##      106      107      108      109      110      111      112      113 
## 0.498016 0.606787 0.449223 0.578076 0.710003 0.633012 0.682970 0.712759 
##      114      115      116      117      118      119      120      121 
## 0.365037 0.732525 0.364620 0.368097 0.386484 0.653685 0.731227 0.424825 
##      122      123      124      125      126      127      128 
## 0.449160 0.450526 0.474054 0.600136 0.552802 0.552521 0.591018 
## 
## Lower & upper 95% CIs:
##         lower    upper
## 66   2.312810 7.475023
## 67   4.252502 7.621903
## 68   4.039837 7.601930
## 69   2.877452 6.654025
## 70   6.417655 8.811423
## 71   6.714164 8.194346
## 72   4.493829 7.689829
## 73   5.053022 7.626102
## 74   4.551676 7.731464
## 75   4.615954 7.955094
## 76   5.424808 7.319534
## 77   4.566237 7.856316
## 78   4.501509 7.211779
## 79   5.156010 6.526447
## 80   5.359982 8.845329
## 81   6.487333 6.802888
## 82   8.118474 8.856606
## 83   8.014009 8.707219
## 84   9.186740 9.535202
## 85   1.818527 4.815066
## 86   1.835235 5.307077
## 87   1.904122 5.800827
## 88   1.578404 5.373091
## 89   1.602441 5.033402
## 90   1.786558 4.266602
## 91   1.732198 4.275646
## 92   1.057303 4.218544
## 93   0.873562 4.174714
## 94   0.690677 4.207825
## 95   1.785578 4.438007
## 96   1.816611 4.503674
## 97   1.818240 4.593599
## 98   1.784788 4.663800
## 99   1.743455 4.754808
## 100  1.652003 4.902419
## 101  1.590844 3.897429
## 102  1.845020 4.192486
## 103  1.766637 4.249410
## 104  1.604269 4.369586
## 105  1.421320 4.622576
## 106  1.634958 4.401311
## 107  1.471195 4.524738
## 108  1.994850 4.622194
## 109  2.101282 5.081711
## 110  2.138841 5.441899
## 111  1.762695 4.881527
## 112  1.778076 5.017642
## 113  1.628061 4.937523
## 114  0.909248 3.277645
## 115  0.562113 3.917150
## 116  0.849250 3.216294
## 117  0.792661 3.170964
## 118  0.514653 2.951631
## 119 -0.241804 2.927547
## 120 -0.468842 2.883221
## 121  0.298599 2.853600
## 122  0.087473 2.714633
## 123 -0.736416 1.894737
## 124 -0.074134 2.624848
## 125 -0.326732 2.710032
## 126 -0.326851 2.587695
## 127 -0.241618 2.672188
## 128 -0.415525 2.598081  
  # WARNING: All values are log-converted (ln)  
 
 
 For the ratio (eggshell thickness/body mass) 
  # Prepare the data and tree
ASREBMnew<-ASRdatanew[-c(1:3,6),]
ASREBMnew[2,2]<-10.89776 # change the value for n07AEP06 to the Crouch & Clarke estimate
dataplot<-ASREBMnew[,1]/ASREBMnew[,2]
names(dataplot)<-rownames(ASREBMnew)
treeplot<-drop.tip(ASRtreenew, setdiff(ASRtreenew$tip.label, rownames(ASREBMnew)))

# Phylogenetic signal
phylosig(treeplot, dataplot, method="lambda", test=TRUE)  
  ## 
## Phylogenetic signal lambda : 0.926905 
## logL(lambda) : 24.8 
## LR(lambda=0) : 11.1954 
## P-value (based on LR test) : 0.000820003  
  # Check best evolutionary model
fit<-list()
for (m in 1:length(models)) {
  fit[[m]]=fitContinuous(treeplot, dataplot, model=models[m], ncores=2)
}  
  ## Warning in matrix(unlist(children), nrow = 2): data length [29] is not a sub-
## multiple or multiple of the number of rows [2]

## Warning in matrix(unlist(children), nrow = 2): data length [29] is not a sub-
## multiple or multiple of the number of rows [2]  
  ## Warning in fitContinuous(treeplot, dataplot, model = models[m], ncores = 2): 
## Parameter estimates appear at bounds:
##  alpha  
  ## Warning in matrix(unlist(children), nrow = 2): data length [29] is not a sub-
## multiple or multiple of the number of rows [2]  
  ## Warning in fitContinuous(treeplot, dataplot, model = models[m], ncores = 2): 
## Parameter estimates appear at bounds:
##  a  
  ## Warning in matrix(unlist(children), nrow = 2): data length [29] is not a sub-
## multiple or multiple of the number of rows [2]  
  ## Warning in fitContinuous(treeplot, dataplot, model = models[m], ncores = 2): 
## Parameter estimates appear at bounds:
##  slope  
  ## Warning in matrix(unlist(children), nrow = 2): data length [29] is not a sub-
## multiple or multiple of the number of rows [2]

## Warning in matrix(unlist(children), nrow = 2): data length [29] is not a sub-
## multiple or multiple of the number of rows [2]  
  modSel.geiger(fit[[1]],fit[[2]],fit[[3]],fit[[4]],fit[[5]],fit[[6]]) # BM is the best fit  
 
 
 
  # Prepare the plot
fitEBMnew<-fastAnc(treeplot, dataplot, vars=TRUE, CI=TRUE)
obj<-contMap(treeplot, dataplot)  
 
 Plot 
 
  plot(setMap(obj, colors=rev(brewer.pal(10,"Spectral"))));
title('Ancestral state reconstruction for (eggshell thickness/body mass)')  
   
  # Add ancestral state values for the nodes on the tree  (and values for the tips)
plot(setMap(obj, colors=rev(brewer.pal(10,"Spectral"))), offset=2); nodelabels(as.factor(round(exp(fitEBMnew$ace), digits=3)), bg="white", cex=0.6, font=2); tiplabels(as.factor(round(exp(ASREBMnew[,1]/ASREBMnew[,2]), digits=3)), bg="white", cex=0.6, font=2, offset=6);
title('Ancestral state reconstruction for (eggshell thickness/body mass)')  
   
  # The color scale on the tree was generated with log-converted values, but the values displayed on these plots for both tips and nodes are the raw values.

fitEBMnew # Ancestral state values for each trait, if you need them  
  ## Ancestral character estimates using fastAnc:
##       17       18       19       20       21       22       23       24 
## 0.702405 0.710805 0.638276 0.632474 0.761029 0.699972 0.679710 0.699906 
##       25       26       27       28       29       30 
## 0.666509 0.652988 0.702725 0.783525 0.805261 0.676563 
## 
## Variances on ancestral states:
##       17       18       19       20       21       22       23       24 
## 0.000882 0.001003 0.000449 0.000172 0.001120 0.000794 0.000519 0.000787 
##       25       26       27       28       29       30 
## 0.000873 0.000281 0.000845 0.000576 0.000147 0.000963 
## 
## Lower & upper 95% CIs:
##       lower    upper
## 17 0.644210 0.760600
## 18 0.648729 0.772881
## 19 0.596734 0.679818
## 20 0.606789 0.658159
## 21 0.695433 0.826625
## 22 0.644758 0.755185
## 23 0.635065 0.724355
## 24 0.644922 0.754891
## 25 0.608581 0.724436
## 26 0.620111 0.685866
## 27 0.645762 0.759687
## 28 0.736499 0.830551
## 29 0.781480 0.829041
## 30 0.615733 0.737394  
  # WARNING: All values are log-converted (ln).  
 The two elephant birds have values similar to those of cassowaries,
emu, rheas and ostrich – their very thick eggshells are “compensated” by
their large body size. There is, however, a visible difference between
 Aepyornis  and  Mullerornis , since their difference in
eggshell thickness is much more pronounced than their difference in body
mass. Conversely, moa show a relatively thin eggshell for such large
animals. Smaller taxa (kiwi, tinamous) show high values due to their
small size. 
 
 
 Same reconstruction with neognaths added to the sample 
  # Prepare the data and tree
AllASREBM<-AllASRdata[-c(46:48,51),]
AllASREBM[47,2]<-10.89776 # change the value for n07AEP06 to the Crouch & Clarke estimate
dataplot<-AllASREBM[,1]/AllASREBM[,2]
names(dataplot)<-rownames(AllASREBM)
treeplot<-drop.tip(AllASRtree, setdiff(AllASRtree$tip.label, rownames(AllASREBM)))

# Phylogenetic signal
phylosig(treeplot, dataplot, method="lambda", test=TRUE)  
  ## 
## Phylogenetic signal lambda : 1.01962 
## logL(lambda) : 1.85383 
## LR(lambda=0) : 40.2791 
## P-value (based on LR test) : 2.20148e-10  
  # Check best evolutionary model
fit<-list()
for (m in 1:length(models)) {
  fit[[m]]=fitContinuous(treeplot, dataplot, model=models[m], ncores=2)
}  
  ## Warning in matrix(unlist(children), nrow = 2): data length [119] is not a sub-
## multiple or multiple of the number of rows [2]

## Warning in matrix(unlist(children), nrow = 2): data length [119] is not a sub-
## multiple or multiple of the number of rows [2]

## Warning in matrix(unlist(children), nrow = 2): data length [119] is not a sub-
## multiple or multiple of the number of rows [2]

## Warning in matrix(unlist(children), nrow = 2): data length [119] is not a sub-
## multiple or multiple of the number of rows [2]

## Warning in matrix(unlist(children), nrow = 2): data length [119] is not a sub-
## multiple or multiple of the number of rows [2]

## Warning in matrix(unlist(children), nrow = 2): data length [119] is not a sub-
## multiple or multiple of the number of rows [2]  
  modSel.geiger(fit[[1]],fit[[2]],fit[[3]],fit[[4]],fit[[5]],fit[[6]]) # BM is the best fit  
 
 
 
  # Prepare the plot
fitAllEBM<-fastAnc(treeplot, dataplot, vars=TRUE, CI=TRUE)
obj<-contMap(treeplot, dataplot)  
 
 Plot 
 
  plot(setMap(obj, colors=rev(brewer.pal(10,"Spectral"))));
title('Ancestral state reconstruction for (eggshell thickness/body mass)')  
   
  # Add ancestral state values for the nodes on the tree  (and values for the tips)
plot(setMap(obj, colors=rev(brewer.pal(10,"Spectral"))), offset=2); nodelabels(as.factor(round(exp(fitAllEBM$ace), digits=3)), bg="white", cex=0.6, font=2); tiplabels(as.factor(round(exp(AllASREBM[,1]/AllASREBM[,2]), digits=3)), bg="white", cex=0.6, font=2, offset=6);
title('Ancestral state reconstruction for (eggshell thickness/body mass)')  
   
  # The color scale on the tree was generated with log-converted values, but the values displayed on these plots for both tips and nodes are the raw values.

fitAllEBM # Ancestral state values for each trait, if you need them  
  ## Ancestral character estimates using fastAnc:
##       62       63       64       65       66       67       68       69 
## 0.826415 0.728221 0.729581 0.770471 0.640270 0.632904 0.719572 0.682020 
##       70       71       72       73       74       75       76       77 
## 0.717630 0.671490 0.653713 0.718457 0.785916 0.805566 0.682188 0.953440 
##       78       79       80       81       82       83       84       85 
## 0.899473 0.876067 0.877982 0.863108 0.989177 0.962412 0.973470 0.973558 
##       86       87       88       89       90       91       92       93 
## 0.976500 0.936278 0.926807 0.915126 0.906242 0.902045 0.894470 1.062054 
##       94       95       96       97       98       99      100      101 
## 1.022841 1.029881 1.029355 1.020116 1.038025 1.049895 0.971550 0.935728 
##      102      103      104      105      106      107      108      109 
## 0.910787 0.940610 0.920101 0.938833 1.191064 1.153342 1.203540 1.214383 
##      110      111      112      113      114      115      116      117 
## 1.263371 1.366961 1.426158 1.287329 1.326716 1.400710 1.365022 1.387114 
##      118      119      120 
## 1.417396 1.371851 1.404157 
## 
## Variances on ancestral states:
##       62       63       64       65       66       67       68       69 
## 0.022485 0.009584 0.010709 0.012035 0.004835 0.001849 0.008626 0.005586 
##       70       71       72       73       74       75       76       77 
## 0.008540 0.009408 0.003029 0.009146 0.006198 0.001585 0.010376 0.007576 
##       78       79       80       81       82       83       84       85 
## 0.010170 0.012812 0.012150 0.009932 0.005190 0.005458 0.008432 0.009195 
##       86       87       88       89       90       91       92       93 
## 0.010437 0.005936 0.006092 0.006499 0.006994 0.007651 0.008914 0.004489 
##       94       95       96       97       98       99      100      101 
## 0.004650 0.005201 0.006452 0.008647 0.006457 0.007867 0.005824 0.007495 
##      102      103      104      105      106      107      108      109 
## 0.009205 0.008207 0.008855 0.009241 0.004733 0.009497 0.004727 0.004772 
##      110      111      112      113      114      115      116      117 
## 0.005011 0.008475 0.009481 0.005508 0.005823 0.005841 0.006146 0.007781 
##      118      119      120 
## 0.007167 0.007164 0.007663 
## 
## Lower & upper 95% CIs:
##        lower    upper
## 62  0.532512 1.120317
## 63  0.536343 0.920099
## 64  0.526755 0.932406
## 65  0.555455 0.985488
## 66  0.503987 0.776554
## 67  0.548633 0.717174
## 68  0.537540 0.901604
## 69  0.535526 0.828513
## 70  0.536505 0.898754
## 71  0.481378 0.861603
## 72  0.545841 0.761585
## 73  0.531016 0.905899
## 74  0.631610 0.940222
## 75  0.727543 0.883589
## 76  0.482540 0.881836
## 77  0.782839 1.124041
## 78  0.701812 1.097134
## 79  0.654218 1.097916
## 80  0.661940 1.094023
## 81  0.667775 1.058442
## 82  0.847981 1.130372
## 83  0.817607 1.107217
## 84  0.793493 1.153448
## 85  0.785615 1.161501
## 86  0.776259 1.176740
## 87  0.785269 1.087288
## 88  0.773826 1.079789
## 89  0.757118 1.073134
## 90  0.742332 1.070151
## 91  0.730601 1.073489
## 92  0.709416 1.079525
## 93  0.930735 1.193374
## 94  0.889194 1.156488
## 95  0.888530 1.171232
## 96  0.871918 1.186791
## 97  0.837860 1.202372
## 98  0.880530 1.195521
## 99  0.876049 1.223741
## 100 0.821968 1.121131
## 101 0.766045 1.105412
## 102 0.722736 1.098839
## 103 0.763047 1.118173
## 104 0.735664 1.104537
## 105 0.750417 1.127249
## 106 1.056225 1.325903
## 107 0.962331 1.344353
## 108 1.068778 1.338301
## 109 1.078980 1.349786
## 110 1.124628 1.402114
## 111 1.186522 1.547400
## 112 1.235316 1.616999
## 113 1.141866 1.432792
## 114 1.177145 1.476287
## 115 1.250912 1.550508
## 116 1.211363 1.518682
## 117 1.214223 1.560005
## 118 1.251464 1.583329
## 119 1.205960 1.537741
## 120 1.232585 1.575729  
  # WARNING: All values are log-converted (ln).  
 
 
 For the ratio (egg mass/body mass) 
  dataplot<-ASREBMnew[,3]/ASREBMnew[,2]
names(dataplot)<-rownames(ASREBMnew)
treeplot<-drop.tip(ASRtreenew, setdiff(ASRtreenew$tip.label, rownames(ASREBMnew)))

# Phylogenetic signal
phylosig(treeplot, dataplot, method="lambda", test=TRUE)  
  ## 
## Phylogenetic signal lambda : 0.996562 
## logL(lambda) : 24.3058 
## LR(lambda=0) : 11.8016 
## P-value (based on LR test) : 0.000591807  
  # Check best evolutionary model
fit<-list()
for (m in 1:length(models)) {
  fit[[m]]=fitContinuous(treeplot, dataplot, model=models[m], ncores=2)
}  
  ## Warning in matrix(unlist(children), nrow = 2): data length [29] is not a sub-
## multiple or multiple of the number of rows [2]

## Warning in matrix(unlist(children), nrow = 2): data length [29] is not a sub-
## multiple or multiple of the number of rows [2]  
  ## Warning in fitContinuous(treeplot, dataplot, model = models[m], ncores = 2): 
## Parameter estimates appear at bounds:
##  alpha  
  ## Warning in matrix(unlist(children), nrow = 2): data length [29] is not a sub-
## multiple or multiple of the number of rows [2]  
  ## Warning in fitContinuous(treeplot, dataplot, model = models[m], ncores = 2): 
## Parameter estimates appear at bounds:
##  a  
  ## Warning in matrix(unlist(children), nrow = 2): data length [29] is not a sub-
## multiple or multiple of the number of rows [2]  
  ## Warning in fitContinuous(treeplot, dataplot, model = models[m], ncores = 2): 
## Parameter estimates appear at bounds:
##  slope  
  ## Warning in matrix(unlist(children), nrow = 2): data length [29] is not a sub-
## multiple or multiple of the number of rows [2]

## Warning in matrix(unlist(children), nrow = 2): data length [29] is not a sub-
## multiple or multiple of the number of rows [2]  
  modSel.geiger(fit[[1]],fit[[2]],fit[[3]],fit[[4]],fit[[5]],fit[[6]]) # BM is the best fit  
 
 
 
  # Prepare the plot
fitEBMnew<-fastAnc(treeplot, dataplot, vars=TRUE, CI=TRUE)
obj<-contMap(treeplot, dataplot)  
 
 Plot 
 
  plot(setMap(obj, colors=rev(brewer.pal(10,"Spectral"))));
title('Ancestral state reconstruction for (egg mass/body mass)')  
   
  # Add ancestral state values for the nodes on the tree (and values for the tips)
plot(setMap(obj, colors=rev(brewer.pal(10,"Spectral"))), offset=2); nodelabels(as.factor(round(exp(fitEBMnew$ace), digits=3)), bg="white", cex=0.6, font=2); tiplabels(as.factor(round(exp(ASREBMnew[,3]/ASREBMnew[,2]), digits=3)), bg="white", cex=0.6, font=2, offset=5);
title('Ancestral state reconstruction for (egg mass/body mass)')  
   
  # The color scale on the tree was generated with log-converted values, but the values displayed on these plots for both tips and nodes are the raw values.

fitEBMnew # Ancestral state values for each trait, if you need them  
  ## Ancestral character estimates using fastAnc:
##       17       18       19       20       21       22       23       24 
## 0.640625 0.630592 0.672461 0.671842 0.589305 0.648250 0.635072 0.651172 
##       25       26       27       28       29       30 
## 0.616731 0.605792 0.659025 0.753809 0.786779 0.671360 
## 
## Variances on ancestral states:
##       17       18       19       20       21       22       23       24 
## 0.000843 0.000959 0.000429 0.000164 0.001070 0.000758 0.000496 0.000752 
##       25       26       27       28       29       30 
## 0.000835 0.000269 0.000807 0.000550 0.000141 0.000921 
## 
## Lower & upper 95% CIs:
##       lower    upper
## 17 0.583733 0.697517
## 18 0.569905 0.691278
## 19 0.631849 0.713073
## 20 0.646732 0.696951
## 21 0.525177 0.653432
## 22 0.594273 0.702227
## 23 0.591427 0.678718
## 24 0.597419 0.704926
## 25 0.560100 0.673361
## 26 0.573651 0.637933
## 27 0.603339 0.714712
## 28 0.707836 0.799782
## 29 0.763531 0.810027
## 30 0.611892 0.730828  
  # WARNING: All values are log-converted (ln).  
 All major paleognath clades (ostrich, rhea, moa + tinamous,
cassowaries + emu) show a decrease in relative egg mass compared to the
value at the previous node, except the clade formed by kiwi and elephant
birds, which show an increase. This is likely due to the very high
relative egg mass found in all kiwi. Moa also present a strong increase
in relative egg mass.  This seems to support the hypothesis that
the kiwi body mass decreased faster than their egg mass, and that they
present an enlarged egg, possibly inherited from a larger ancestor prior
to their miniaturization, and likely associated with extreme k life
history strategy  (e.g. Calder, 1979; Hume and Robertson,
2021). 
 
 
 Same reconstruction with neognaths added to the sample 
  dataplot<-AllASREBM[,3]/AllASREBM[,2]
names(dataplot)<-rownames(AllASREBM)
treeplot<-drop.tip(AllASRtree, setdiff(AllASRtree$tip.label, rownames(AllASREBM)))

# Phylogenetic signal
phylosig(treeplot, dataplot, method="lambda", test=TRUE)  
  ## 
## Phylogenetic signal lambda : 1.02954 
## logL(lambda) : 46.5585 
## LR(lambda=0) : 56.7344 
## P-value (based on LR test) : 4.98828e-14  
  # Check best evolutionary model
fit<-list()
for (m in 1:length(models)) {
  fit[[m]]=fitContinuous(treeplot, dataplot, model=models[m], ncores=2)
}  
  ## Warning in matrix(unlist(children), nrow = 2): data length [119] is not a sub-
## multiple or multiple of the number of rows [2]

## Warning in matrix(unlist(children), nrow = 2): data length [119] is not a sub-
## multiple or multiple of the number of rows [2]

## Warning in matrix(unlist(children), nrow = 2): data length [119] is not a sub-
## multiple or multiple of the number of rows [2]  
  ## Warning in fitContinuous(treeplot, dataplot, model = models[m], ncores = 2): 
## Parameter estimates appear at bounds:
##  a  
  ## Warning in matrix(unlist(children), nrow = 2): data length [119] is not a sub-
## multiple or multiple of the number of rows [2]

## Warning in matrix(unlist(children), nrow = 2): data length [119] is not a sub-
## multiple or multiple of the number of rows [2]

## Warning in matrix(unlist(children), nrow = 2): data length [119] is not a sub-
## multiple or multiple of the number of rows [2]  
  modSel.geiger(fit[[1]],fit[[2]],fit[[3]],fit[[4]],fit[[5]],fit[[6]]) # BM is the best fit  
 
 
 
  # Prepare the plot
fitAllEBM<-fastAnc(treeplot, dataplot, vars=TRUE, CI=TRUE)
obj<-contMap(treeplot, dataplot)  
 
 Plot 
 
  plot(setMap(obj, colors=rev(brewer.pal(10,"Spectral"))));
title('Ancestral state reconstruction for (egg mass/body mass)')  
   
  # Add ancestral state values for the nodes on the tree (and values for the tips)
plot(setMap(obj, colors=rev(brewer.pal(10,"Spectral"))), offset=2); nodelabels(as.factor(round(exp(fitAllEBM$ace), digits=3)), bg="white", cex=0.6, font=2); tiplabels(as.factor(round(exp(AllASREBM[,3]/AllASREBM[,2]), digits=3)), bg="white", cex=0.6, font=2, offset=5);
title('Ancestral state reconstruction for (egg mass/body mass)')  
   
  # The color scale on the tree was generated with log-converted values, but the values displayed on these plots for both tips and nodes are the raw values.

fitAllEBM # Ancestral state values for each trait, if you need them  
  ## Ancestral character estimates using fastAnc:
##       62       63       64       65       66       67       68       69 
## 0.565256 0.630401 0.623648 0.585813 0.671724 0.671683 0.639743 0.634070 
##       70       71       72       73       74       75       76       77 
## 0.643480 0.614568 0.605477 0.652197 0.752771 0.786647 0.668918 0.496483 
##       78       79       80       81       82       83       84       85 
## 0.515512 0.539635 0.504834 0.494176 0.480898 0.480729 0.455736 0.447553 
##       86       87       88       89       90       91       92       93 
## 0.442659 0.489243 0.493022 0.498506 0.501920 0.507051 0.509875 0.464378 
##       94       95       96       97       98       99      100      101 
## 0.508290 0.523975 0.525997 0.533311 0.538834 0.548561 0.523305 0.537951 
##      102      103      104      105      106      107      108      109 
## 0.547948 0.524041 0.531032 0.538606 0.392100 0.424917 0.384514 0.378075 
##      110      111      112      113      114      115      116      117 
## 0.343797 0.292691 0.271992 0.321446 0.294824 0.162300 0.276387 0.272308 
##      118      119      120 
## 0.235450 0.253276 0.246832 
## 
## Variances on ancestral states:
##       62       63       64       65       66       67       68       69 
## 0.005262 0.002243 0.002506 0.002816 0.001131 0.000433 0.002018 0.001307 
##       70       71       72       73       74       75       76       77 
## 0.001998 0.002202 0.000709 0.002140 0.001450 0.000371 0.002428 0.001773 
##       78       79       80       81       82       83       84       85 
## 0.002380 0.002998 0.002843 0.002324 0.001214 0.001277 0.001973 0.002152 
##       86       87       88       89       90       91       92       93 
## 0.002442 0.001389 0.001426 0.001521 0.001636 0.001790 0.002086 0.001050 
##       94       95       96       97       98       99      100      101 
## 0.001088 0.001217 0.001510 0.002023 0.001511 0.001841 0.001363 0.001754 
##      102      103      104      105      106      107      108      109 
## 0.002154 0.001920 0.002072 0.002162 0.001107 0.002222 0.001106 0.001117 
##      110      111      112      113      114      115      116      117 
## 0.001173 0.001983 0.002218 0.001289 0.001363 0.001367 0.001438 0.001821 
##      118      119      120 
## 0.001677 0.001676 0.001793 
## 
## Lower & upper 95% CIs:
##        lower    upper
## 62  0.423085 0.707427
## 63  0.537583 0.723220
## 64  0.525534 0.721762
## 65  0.481802 0.689823
## 66  0.605798 0.737649
## 67  0.630918 0.712447
## 68  0.551687 0.727798
## 69  0.563206 0.704934
## 70  0.555863 0.731096
## 71  0.522604 0.706533
## 72  0.553296 0.657659
## 73  0.561525 0.742868
## 74  0.678128 0.827414
## 75  0.748904 0.824389
## 76  0.572342 0.765495
## 77  0.413958 0.579009
## 78  0.419897 0.611128
## 79  0.432319 0.646952
## 80  0.400327 0.609341
## 81  0.399686 0.588665
## 82  0.412596 0.549199
## 83  0.410681 0.550776
## 84  0.368675 0.542797
## 85  0.356638 0.538467
## 86  0.345796 0.539522
## 87  0.416194 0.562292
## 88  0.419020 0.567025
## 89  0.422072 0.574940
## 90  0.422632 0.581209
## 91  0.424118 0.589985
## 92  0.420358 0.599392
## 93  0.400854 0.527902
## 94  0.443640 0.572940
## 95  0.455598 0.592351
## 96  0.449839 0.602154
## 97  0.445148 0.621475
## 98  0.462648 0.615020
## 99  0.464466 0.632656
## 100 0.450947 0.595662
## 101 0.455869 0.620033
## 102 0.456980 0.638915
## 103 0.438147 0.609934
## 104 0.441813 0.620250
## 105 0.447462 0.629749
## 106 0.326873 0.457326
## 107 0.332518 0.517315
## 108 0.319325 0.449703
## 109 0.312576 0.443574
## 110 0.276682 0.410912
## 111 0.205406 0.379976
## 112 0.179675 0.364308
## 113 0.251081 0.391812
## 114 0.222471 0.367176
## 115 0.089837 0.234762
## 116 0.202056 0.350717
## 117 0.188674 0.355941
## 118 0.155183 0.315718
## 119 0.173029 0.333523
## 120 0.163836 0.329827  
  # WARNING: All values are log-converted (ln).  
 
 
 
 
 New analyses with separate datasets for eggshell specimens and
skeletal specimens 
 
 Load data and trees for new elephant bird data 
 
  # Data for all birds except elephant birds, which we can add later
datalitt<-read.table("Paleognathdata_maleBM_nonewdata.txt", header=TRUE)

# Elephant bird data
EBeggonly<-read.table("EBeggonly.txt", header=TRUE)
EBboneonly<-read.table("EBboneonly.txt", header=TRUE)
EBeggall<-read.table("EBeggall.txt", header=TRUE)

# Trees
treeegg<-read.nexus("Newwholetree.eggs.nex"); plotTree(treeegg)  
   
  treebone<-read.nexus("Newwholetree.bones.nex"); plotTree(treebone)  
   
  treealleggs<-read.nexus("Newwholetree.alleggs.nex"); plotTree(treealleggs)  
   
 
 Tree with elephant bird egg specimens only 
 
 PGLS regressions to estimate egg mass and body mass of eggshell
specimens 
 
 For body mass 
 
  # Data and tree
treeeggBM<-drop.tip(treeegg, setdiff(treeegg$tip.label, datalitt$Species))
rownames(datalitt)<-datalitt$Species
datalitt<-ReorderData(treeeggBM, datalitt)

# Correcting the variance-covariance matrix for the non-ultrametric tree
Wbm<-diag(vcv.phylo(treeeggBM))

# Best fit for the alpha parameter in the Ornstein-Uhlenbeck (OU) model
alpha <- seq(0, 1, 0.1)
fit <- list()
form <- log(Thickness)~log(Bodymass)
for (i in seq_along(alpha)) {
  cor <- corMartins(alpha[i], phy = treeeggBM, fixed = T, form=~Species)
  fit[[i]] <- gls(form, correlation = cor, data = datalitt, weights=varFixed(~Wbm), na.action=na.exclude, method = "ML")
}
plot(sapply(fit, logLik)) # best fit: 0.1  
   
  # Best fit for the g parameter in the Early Burst (EB) model
g <- seq(0.1, 1, 0.1)
fit <- list()
form <- log(Thickness)~log(Bodymass)
for (i in seq_along(g)) {
  cor <- corBlomberg(g[i], phy = treeeggBM, fixed = T, form=~Species)
  fit[[i]] <- gls(form, correlation = cor, data = datalitt, weights=varFixed(~Wbm), na.action=na.exclude, method = "ML")
}
plot(sapply(fit, logLik)) # best fit: 0.1  
   
  # Best fit for Pagel's lambda in the Lambda model
lambda <- seq(0, 1, 0.1)
fit <- list()
form <- log(Thickness)~log(Bodymass)
for (i in seq_along(alpha)) {
  cor <- corPagel(lambda[i], phy = treeeggBM, fixed = T, form=~Species)
  fit[[i]] <- gls(form, correlation = cor, data = datalitt, weights=varFixed(~Wbm), na.action=na.exclude, method = "ML")
}
plot(sapply(fit, logLik)) # best fit: 0.6  
   
  # PGLS models
BM<-gls(log(Thickness)~log(Bodymass), data=datalitt, correlation=corBrownian(phy=treeeggBM, form=~Species), weights=varFixed(~Wbm), method="ML")
OU<-gls(log(Thickness)~log(Bodymass), data=datalitt, correlation=corMartins(0.1, phy=treeeggBM, fixed=T, form=~Species), weights=varFixed(~Wbm), method="ML")
Lambda<-gls(log(Thickness)~log(Bodymass), data=datalitt, correlation=corPagel(0.6, phy=treeeggBM, fixed=T, form=~Species), weights=varFixed(~Wbm), method="ML")
EB<-gls(log(Thickness)~log(Bodymass), data=datalitt, correlation=corBlomberg(0.1, phy=treeeggBM, fixed=T, form=~Species), weights=varFixed(~Wbm), method="ML")
OLS<-gls(log(Thickness)~log(Bodymass), data=datalitt, method="ML")

Cand.models = list()
Cand.models[[1]] = BM
Cand.models[[2]] = OU
Cand.models[[3]] = Lambda
Cand.models[[4]] = EB
Cand.models[[5]] = OLS

Modnames = paste(c("BM", "OU", "Lambda", "EB", "OLS"), sep = " ")
aictab(cand.set = Cand.models, modnames = Modnames, sort = T)  
 
 
 
  # Best model
OUBM<-OU
summary(OUBM)  
  ## Generalized least squares fit by maximum likelihood
##   Model: log(Thickness) ~ log(Bodymass) 
##   Data: datalitt 
##       AIC       BIC logLik
##   -25.856 -19.62339 15.928
## 
## Correlation Structure: corMartins
##  Formula: ~Species 
##  Parameter estimate(s):
## alpha 
##   0.1 
## Variance function:
##  Structure: fixed weights
##  Formula: ~Wbm 
## 
## Coefficients:
##                  Value  Std.Error  t-value p-value
## (Intercept)   3.382209 0.06714841 50.36916       0
## log(Bodymass) 0.341402 0.01009591 33.81584       0
## 
##  Correlation: 
##               (Intr)
## log(Bodymass) -0.928
## 
## Standardized residuals:
##         Min          Q1         Med          Q3         Max 
## -2.03026727 -0.63453091  0.02208392  0.65580806  2.47317669 
## 
## Residual standard error: 0.01659935 
## Degrees of freedom: 59 total; 57 residual  
  R2.pred(OUBM) # R2 = 0.96: very high pseudo R-squared  
  ## [1] 0.9564577  
 
 Plot of the regression and estimates of body mass for elephant bird
eggshells 
 
  # Estimates based on the OU model
massestim<-exp((log(EBeggonly[,3])-OU$coefficients[1])/
                              OU$coefficients[2])
dataeggplot<-as.data.frame(cbind(c(rep("Neognathae",45),rep("Palaeognathae",18)),
                            c(datalitt$Species,EBeggonly$Species),
                            c(datalitt$Thickness,
                                         EBeggonly$Eggshell_thickness_average),
                            c(datalitt$Bodymass,massestim),
                            c(datalitt$Eggmass,rep(NA,4))))
colnames(dataeggplot)<-c("Group",colnames(datalitt))
for (i in 3:5) {
  dataeggplot[,i]<-as.numeric(dataeggplot[,i])
}

# Some of the estimates can be hard to differentiate from species with actual body mass values
# If you want to see them, use this line of code:
dataeggplot[c(60:63),1]<-"Predicted"

# Plot the regression with the estimates on the regression line
ggplot(dataeggplot, aes(log(Bodymass), log(Thickness), color=Group)) +
  geom_point(size=5) +
  geom_text(aes(label=Species),hjust=-0.1, vjust=0.4) +
  xlab("ln body mass (kg)") +
  ylab("ln eggshell thickness (µm)") +
  geom_abline(intercept=OU$coefficients[1], slope=OU$coefficients[2],
              colour="skyblue2", size=1.3) +
  theme(panel.background = element_rect(fill="black")) +
  scale_color_manual(values=wes_palette("Zissou1")[c(1,3,5)])  
   
  # If you want to look at the values:
dataeggplot[c(60:63),c(2:4)]  
 
 
 
 
 For egg mass 
 
  # Data and tree
treeeggEM<-drop.tip(treeegg, setdiff(treeegg$tip.label, datalitt$Species))

# Correcting the variance-covariance matrix for the non-ultrametric tree
Wem<-diag(vcv.phylo(treeeggEM))

# Best fit for the alpha parameter in the Ornstein-Uhlenbeck (OU) model
alpha <- seq(0, 1, 0.1)
fit <- list()
form <- log(Thickness)~log(Eggmass)
for (i in seq_along(alpha)) {
  cor <- corMartins(alpha[i], phy = treeeggEM, fixed = T, form=~Species)
  fit[[i]] <- gls(form, correlation = cor, data = datalitt, weights=varFixed(~Wem), na.action=na.exclude, method = "ML")
}
plot(sapply(fit, logLik)) # best fit: 0.1  
   
  # Best fit for the g parameter in the Early Burst (EB) model
g <- seq(0.1, 1, 0.1)
fit <- list()
form <- log(Thickness)~log(Eggmass)
for (i in seq_along(g)) {
  cor <- corBlomberg(g[i], phy = treeeggEM, fixed = T, form=~Species)
  fit[[i]] <- gls(form, correlation = cor, data = datalitt, weights=varFixed(~Wem), na.action=na.exclude, method = "ML")
}
plot(sapply(fit, logLik)) # best fit: 0.1  
   
  # Best fit for Pagel's lambda in the Lambda model
lambda <- seq(0, 1, 0.1)
fit <- list()
form <- log(Thickness)~log(Eggmass)
for (i in seq_along(alpha)) {
  cor <- corPagel(lambda[i], phy = treeeggEM, fixed = T, form=~Species)
  fit[[i]] <- gls(form, correlation = cor, data = datalitt, weights=varFixed(~Wem), na.action=na.exclude, method = "ML")
}
plot(sapply(fit, logLik)) # best fit: 0.8  
   
  # PGLS models
BM<-gls(log(Thickness)~log(Eggmass), data=datalitt, correlation=corBrownian(phy=treeeggEM, form=~Species), weights=varFixed(~Wem), method="ML")
OU<-gls(log(Thickness)~log(Eggmass), data=datalitt, correlation=corMartins(0.1, phy=treeeggEM, fixed=T, form=~Species), weights=varFixed(~Wem), method="ML")
Lambda<-gls(log(Thickness)~log(Eggmass), data=datalitt, correlation=corPagel(0.8, phy=treeeggEM, fixed=T, form=~Species), weights=varFixed(~Wem), method="ML")
EB<-gls(log(Thickness)~log(Eggmass), data=datalitt, correlation=corBlomberg(0.1, phy=treeeggEM, fixed=T, form=~Species), weights=varFixed(~Wem), method="ML")
OLS<-gls(log(Thickness)~log(Eggmass), data=datalitt, method="ML")

Cand.models = list()
Cand.models[[1]] = BM
Cand.models[[2]] = OU
Cand.models[[3]] = Lambda
Cand.models[[4]] = EB
Cand.models[[5]] = OLS

Modnames = paste(c("BM", "OU", "Lambda", "EB", "OLS"), sep = " ")
aictab(cand.set = Cand.models, modnames = Modnames, sort = T)  
 
 
 
  # Best model
lambdaEM<-Lambda
summary(lambdaEM)  
  ## Generalized least squares fit by maximum likelihood
##   Model: log(Thickness) ~ log(Eggmass) 
##   Data: datalitt 
##         AIC       BIC   logLik
##   -20.04925 -13.81663 13.02462
## 
## Correlation Structure: corPagel
##  Formula: ~Species 
##  Parameter estimate(s):
## lambda 
##    0.8 
## Variance function:
##  Structure: fixed weights
##  Formula: ~Wem 
## 
## Coefficients:
##                 Value  Std.Error  t-value p-value
## (Intercept)  4.013223 0.14419488 27.83194       0
## log(Eggmass) 0.443596 0.02202765 20.13813       0
## 
##  Correlation: 
##              (Intr)
## log(Eggmass) -0.658
## 
## Standardized residuals:
##         Min          Q1         Med          Q3         Max 
## -3.16438617 -0.36473900 -0.02512109  0.62911231  1.94345987 
## 
## Residual standard error: 0.02166992 
## Degrees of freedom: 59 total; 57 residual  
  R2.pred(lambdaEM) # R2 = 0.96: very high pseudo R-squared  
  ## [1] 0.961307  
 
 Plot of the regression and estimates of egg mass for elephant bird
eggshells 
 
  # Estimates based on the OU model
massestim<-exp((log(EBeggonly[,3])-lambdaEM$coefficients[1])/
                              lambdaEM$coefficients[2])
dataeggplot[c(60:63),5]<-massestim

# Some of the estimates can be hard to differentiate from species with actual body mass values
# If you want to see them, use this line of code:
dataeggplot[c(60:63),1]<-"Predicted"

# Plot the regression with the estimates on the regression line
ggplot(dataeggplot, aes(log(Eggmass), log(Thickness), color=Group)) +
  geom_point(size=5) +
  geom_text(aes(label=Species),hjust=-0.1, vjust=0.4) +
  xlab("ln egg mass (g)") +
  ylab("ln eggshell thickness (µm)") +
  geom_abline(intercept=lambdaEM$coefficients[1], slope=lambdaEM$coefficients[2],
              colour="skyblue2", size=1.3) +
  theme(panel.background = element_rect(fill="black")) +
  scale_color_manual(values=wes_palette("Zissou1")[c(1,3,5)])  
   
  # If you want to look at the values:
dataeggplot[c(60:63),c(2,3,5)]  
 
 
 
 
 
 Ancestral reconstructions with mass estimates from the
regressions 
 
 For eggshell thickness, egg mass, and body mass 
 
  datapalegg<-dataeggplot[c(46:63),]; rownames(datapalegg)<-datapalegg$Species
treepalegg<-drop.tip(treeegg, setdiff(treeegg$tip.label, datapalegg$Species))
datapalegg<-ReorderData(treepalegg, datapalegg)

# Phylogenetic signal (Pagel's lambda) of each variable
var=list(); phy=list()
for (i in 3:5) {
  var<-log(datapalegg[,i]); names(var)<-datapalegg$Species
  phy[[i]]<-phylosig(treepalegg, var, method="lambda", test=T)
}
phy # Highly significant for all three traits  
  ## [[1]]
## NULL
## 
## [[2]]
## NULL
## 
## [[3]]
## 
## Phylogenetic signal lambda : 0.949215 
## logL(lambda) : -14.084 
## LR(lambda=0) : 12.7992 
## P-value (based on LR test) : 0.000346769 
## 
## 
## [[4]]
## 
## Phylogenetic signal lambda : 0.935014 
## logL(lambda) : -31.5046 
## LR(lambda=0) : 14.1759 
## P-value (based on LR test) : 0.000166487 
## 
## 
## [[5]]
## 
## Phylogenetic signal lambda : 0.916484 
## logL(lambda) : -26.3165 
## LR(lambda=0) : 10.8846 
## P-value (based on LR test) : 0.000969694  
  # Check best evolutionary model
var=list(); fit=list(); mod=list()
for (i in 3:5) {
  var<-log(datapalegg[,i]); names(var)<-datapalegg$Species
  for (m in 1:length(models)) {
    fit[[m]]=fitContinuous(treepalegg, var, model=models[m], ncores=2)
  }
  mod[[i]]<-modSel.geiger(fit[[1]],fit[[2]],fit[[3]],fit[[4]],fit[[5]],fit[[6]])
}
mod # BM model is the best fit for eggshell thickness, lambda is the best fit for egg mass and body mass.  
  ## [[1]]
## NULL
## 
## [[2]]
## NULL
## 
## [[3]]
##          K    logLik     AICc  deltaAICc Weight Evidence ratio
## fit[[1]] 2 -15.05245 34.90489  0.0000000 0.4133       1.000000
## fit[[5]] 3 -14.08395 35.88219  0.9772963 0.2535       1.630111
## fit[[2]] 3 -14.77422 37.26272  2.3578295 0.1271       3.250844
## fit[[4]] 3 -14.93760 37.58948  2.6845944 0.1080       3.827827
## fit[[3]] 3 -15.05247 37.81922  2.9143286 0.0963       4.293766
## fit[[6]] 2 -20.46457 45.72914 10.8242509 0.0018     224.107413
## 
## [[4]]
##          K    logLik     AICc  deltaAICc Weight Evidence ratio
## fit[[5]] 3 -31.50457 70.72342  0.0000000 0.3521       1.000000
## fit[[1]] 2 -33.02154 70.84307  0.1196472 0.3316       1.061649
## fit[[2]] 3 -32.48670 72.68769  1.9642702 0.1318       2.670151
## fit[[4]] 3 -32.70536 73.12500  2.4015762 0.1060       3.322735
## fit[[3]] 3 -33.02157 73.75742  3.0339996 0.0772       4.558528
## fit[[6]] 2 -38.57025 81.94050 11.2170775 0.0013     272.745392
## 
## [[5]]
##          K    logLik     AICc deltaAICc Weight Evidence ratio
## fit[[5]] 3 -26.31653 60.34734 0.0000000 0.4097       1.000000
## fit[[1]] 2 -28.20720 61.21440 0.8670616 0.2656       1.542695
## fit[[2]] 3 -27.34803 62.41035 2.0630150 0.1460       2.805292
## fit[[4]] 3 -27.64049 62.99526 2.6479216 0.1090       3.758278
## fit[[3]] 3 -28.20725 64.12878 3.7814396 0.0619       6.624135
## fit[[6]] 2 -31.73434 68.26868 7.9213421 0.0078      52.492540  
  # Plots
dataplot=list(); fit=list(); obj=list()
for (i in 3:5) {
  dataplot[[i]]<-as.vector(log(datapalegg[,i]))
  names(dataplot[[i]])<-datapalegg$Species
  fit[[i]]<-fastAnc(treepalegg, dataplot[[i]], vars=TRUE, CI=TRUE)
  obj[[i]]<-setMap(contMap(treepalegg, dataplot[[i]]),
                   colors=rev(brewer.pal(10,"Spectral")))
}  
 
 Plots for three traits (all plots generated at once) 
 
  # Just the trees
for (i in 3:5) {
  plot(obj[[i]])
  title(paste('Ancestral state reconstruction for', colnames(datapalegg)[i]))
}  
     
  # Trees with values mapped on branches
for (i in 3:5) {
  plot(setMap(obj[[i]], colors=rev(brewer.pal(10,"Spectral"))), offset=2);
  nodelabels(as.factor(round(exp(fit[[i]]$ace), digits=0)), bg="white", cex=0.6,
             font=2);
  tiplabels(as.factor(round(exp(dataplot[[i]]), digits=0)), bg="white",
            cex=0.6, font=2, offset=4);
  title(paste('Ancestral state reconstruction for', colnames(datapalegg)[i]))
}  
     
  # The color scale on the tree was generated with log-converted values, but the values displayed on these plots for both tips and nodes are the raw values.
# I did not include digits after the decimal point to make the figures easier to read. If you want to display more digits, just change the value of argument 'digits' in the above code.

fit # Ancestral state values for each trait, if you need them  
  ## [[1]]
## NULL
## 
## [[2]]
## NULL
## 
## [[3]]
## Ancestral character estimates using fastAnc:
##       19       20       21       22       23       24       25       26 
## 6.793107 6.743600 6.618497 6.045851 7.202181 7.012452 6.760477 6.806189 
##       27       28       29       30       31       32       33       34 
## 6.765244 6.840201 6.876009 6.766566 6.112522 5.975503 7.234910 7.867675 
##       35 
## 7.737417 
## 
## Variances on ancestral states:
##       19       20       21       22       23       24       25       26 
## 0.147321 0.116463 0.131945 0.149895 0.060386 0.023096 0.105972 0.069772 
##       27       28       29       30       31       32       33       34 
## 0.105253 0.117435 0.037843 0.113185 0.077415 0.019798 0.120915 0.009382 
##       35 
## 0.008283 
## 
## Lower & upper 95% CIs:
##       lower    upper
## 19 6.040813 7.545401
## 20 6.074718 7.412482
## 21 5.906540 7.330453
## 22 5.287012 6.804690
## 23 6.720538 7.683823
## 24 6.714585 7.310319
## 25 6.122433 7.398521
## 26 6.288469 7.323910
## 27 6.129365 7.401122
## 28 6.168532 7.511870
## 29 6.494724 7.257294
## 30 6.107163 7.425968
## 31 5.567181 6.657864
## 32 5.699718 6.251289
## 33 6.553362 7.916459
## 34 7.677826 8.057525
## 35 7.559035 7.915799
## 
## 
## [[4]]
## Ancestral character estimates using fastAnc:
##        19        20        21        22        23        24        25        26 
##  9.961158  9.872363  9.605290  8.177317 11.343105 11.099584  9.928917 10.046706 
##        27        28        29        30        31        32        33        34 
##  9.946147 10.353963 10.544984  9.938932  7.887962  7.441941 11.293198 13.138642 
##        35 
## 12.756985 
## 
## Variances on ancestral states:
##       19       20       21       22       23       24       25       26 
## 1.084828 0.857599 0.971610 1.103785 0.444667 0.170071 0.780345 0.513779 
##       27       28       29       30       31       32       33       34 
## 0.775056 0.864761 0.278666 0.833463 0.570063 0.145790 0.890388 0.069088 
##       35 
## 0.060994 
## 
## Lower & upper 95% CIs:
##        lower     upper
## 19  7.919719 12.002598
## 20  8.057273 11.687453
## 21  7.673313 11.537268
## 22  6.118118 10.236517
## 23 10.036112 12.650099
## 24 10.291287 11.907881
## 25  8.197509 11.660326
## 26  8.641810 11.451602
## 27  8.220616 11.671678
## 28  8.531309 12.176617
## 29  9.510323 11.579646
## 30  8.149565 11.728298
## 31  6.408113  9.367810
## 32  6.693565  8.190317
## 33  9.443735 13.142661
## 34 12.623462 13.653822
## 35 12.272924 13.241045
## 
## 
## [[5]]
## Ancestral character estimates using fastAnc:
##       19       20       21       22       23       24       25       26 
## 6.364987 6.315564 6.105091 4.908663 7.644726 7.460755 6.385109 6.374125 
##       27       28       29       30       31       32       33       34 
## 6.411822 6.361489 6.383228 6.460438 5.894512 5.846064 7.343653 8.691594 
##       35 
## 8.396793 
## 
## Variances on ancestral states:
##       19       20       21       22       23       24       25       26 
## 0.635397 0.502306 0.569084 0.646501 0.260447 0.099613 0.457058 0.300927 
##       27       28       29       30       31       32       33       34 
## 0.453960 0.506502 0.163218 0.488170 0.333893 0.085391 0.521511 0.040466 
##       35 
## 0.035725 
## 
## Lower & upper 95% CIs:
##       lower    upper
## 19 4.802636 7.927338
## 20 4.926442 7.704686
## 21 4.626513 7.583669
## 22 3.332719 6.484606
## 23 6.644459 8.644992
## 24 6.842151 8.079360
## 25 5.060030 7.710188
## 26 5.298931 7.449318
## 27 5.091241 7.732402
## 28 4.966578 7.756400
## 29 5.591382 7.175074
## 30 5.091003 7.829873
## 31 4.761956 7.027067
## 32 5.273318 6.418810
## 33 5.928225 8.759082
## 34 8.297317 9.085871
## 35 8.026333 8.767254  
  # WARNING: All values are log-converted (ln)  
 
 
 
 Tree with elephant bird bone specimens only 
 
 Ancestral reconstructions of body mass using estimates from Hansford
and Turvey (2018) 
 
 Data 
 
  # Average
Bdataavg<-EBboneonly[,c(1,2)]; colnames(Bdataavg)[2]<-"Bodymass"
databoneavg<-rbind(datalitt[,c(1,3)],Bdataavg)

# Min
Bdatamin<-EBboneonly[,c(1,3)]; colnames(Bdatamin)[2]<-"Bodymass"
databonemin<-rbind(datalitt[,c(1,3)],Bdatamin)

# Max
Bdatamax<-EBboneonly[,c(1,4)]; colnames(Bdatamax)[2]<-"Bodymass"
databonemax<-rbind(datalitt[,c(1,3)],Bdatamax)

# Group them in one dataframe
Bdata<-cbind(databoneavg, databonemin[,2], databonemax[,2])
colnames(Bdata)[2:4]<-c("Average","Minimum", "Maximum")  
 
 Prepare data for plots 
 
  datapalbone<-Bdata[c(46:63),]; rownames(datapalbone)<-datapalbone$Species
treepalbone<-drop.tip(treebone, setdiff(treebone$tip.label, datapalbone$Species))
datapalbone<-ReorderData(treepalbone, datapalbone)

# Phylogenetic signal (Pagel's lambda) of each variable
var=list(); phy=list()
for (i in 2:4) {
  var<-log(datapalbone[,i]); names(var)<-datapalbone$Species
  phy[[i]]<-phylosig(treepalbone, var, method="lambda", test=T)
}
phy # Highly significant for all three traits  
  ## [[1]]
## NULL
## 
## [[2]]
## 
## Phylogenetic signal lambda : 0.984362 
## logL(lambda) : -28.6321 
## LR(lambda=0) : 20.5437 
## P-value (based on LR test) : 5.82856e-06 
## 
## 
## [[3]]
## 
## Phylogenetic signal lambda : 0.978949 
## logL(lambda) : -28.7598 
## LR(lambda=0) : 19.2759 
## P-value (based on LR test) : 1.13128e-05 
## 
## 
## [[4]]
## 
## Phylogenetic signal lambda : 0.993499 
## logL(lambda) : -28.4703 
## LR(lambda=0) : 21.9287 
## P-value (based on LR test) : 2.82965e-06  
  # Check best evolutionary model
var=list(); fit=list(); mod=list()
for (i in 2:4) {
  var<-log(datapalbone[,i]); names(var)<-datapalbone$Species
  for (m in 1:length(models)) {
    fit[[m]]=fitContinuous(treepalbone, var, model=models[m], ncores=2)
  }
  mod[[i]]<-modSel.geiger(fit[[1]],fit[[2]],fit[[3]],fit[[4]],fit[[5]],fit[[6]])
}
mod # BM model is the best fit for all traits  
  ## [[1]]
## NULL
## 
## [[2]]
##          K    logLik     AICc deltaAICc Weight Evidence ratio
## fit[[1]] 2 -29.35971 63.51941  0.000000 0.4541       1.000000
## fit[[5]] 3 -28.63215 64.97858  1.459162 0.2189       2.074211
## fit[[3]] 3 -29.31451 66.34331  2.823894 0.1106       4.103938
## fit[[4]] 3 -29.31480 66.34389  2.824471 0.1106       4.105121
## fit[[2]] 3 -29.35970 66.43369  2.914273 0.1058       4.293646
## fit[[6]] 2 -38.87210 82.54421 19.024790 0.0000   13526.353697
## 
## [[3]]
##          K    logLik     AICc deltaAICc Weight Evidence ratio
## fit[[1]] 2 -29.74988 64.29976  0.000000 0.4281       1.000000
## fit[[5]] 3 -28.75979 65.23387  0.934110 0.2684       1.595289
## fit[[2]] 3 -29.70818 67.13065  2.830889 0.1040       4.118316
## fit[[4]] 3 -29.74985 67.21398  2.914214 0.0997       4.293520
## fit[[3]] 3 -29.74985 67.21398  2.914215 0.0997       4.293522
## fit[[6]] 2 -38.36966 81.53932 17.239562 0.0001    5540.174274
## 
## [[4]]
##          K    logLik     AICc deltaAICc Weight Evidence ratio
## fit[[1]] 2 -28.61087 62.02173  0.000000 0.4650       1.000000
## fit[[3]] 3 -28.27180 64.25788  2.236142 0.1520       3.058949
## fit[[4]] 3 -28.28480 64.28388  2.262149 0.1500       3.098985
## fit[[5]] 3 -28.47031 64.65490  2.633163 0.1246       3.730646
## fit[[2]] 3 -28.61086 64.93602  2.914281 0.1083       4.293664
## fit[[6]] 2 -39.39932 83.59864 21.576902 0.0000   48457.913051  
  # Plots
dataplot=list(); fit=list(); obj=list()
for (i in 2:4) {
  dataplot[[i]]<-as.vector(log(datapalbone[,i]))
  names(dataplot[[i]])<-datapalbone$Species
  fit[[i]]<-fastAnc(treepalbone, dataplot[[i]], vars=TRUE, CI=TRUE)
  obj[[i]]<-setMap(contMap(treepalbone, dataplot[[i]]),
                   colors=rev(brewer.pal(10,"Spectral")))
}  
 
 Plots for three traits (all plots generated at once) 
 
  # Just the trees
for (i in 2:4) {
  plot(obj[[i]])
  title(paste("Ancestral reconstruction for", colnames(datapalbone)[i], "Body Mass"))
}  
     
  # Trees with values mapped on branches
for (i in 2:4) {
  plot(obj[[i]], offset=2);
  nodelabels(as.factor(round(exp(fit[[i]]$ace), digits=0)), bg="white", cex=0.6,
             font=2);
  tiplabels(as.factor(round(exp(dataplot[[i]]), digits=0)), bg="white",
            cex=0.6, font=2, offset=4);
  title(paste('Ancestral state reconstruction for', colnames(datapalbone)[i], 'Body Mass'))
}  
     
  # The color scale on the tree was generated with log-converted values, but the values displayed on these plots for both tips and nodes are the raw values.
# I did not include digits after the decimal point to make the figures easier to read. If you want to display more digits, just change the value of argument 'digits' in the above code.

fit # Ancestral state values for each trait, if you need them  
  ## [[1]]
## NULL
## 
## [[2]]
## Ancestral character estimates using fastAnc:
##        19        20        21        22        23        24        25        26 
## 10.017703  9.931939  9.650041  8.199822 11.347858 11.100608 10.001306 10.055237 
##        27        28        29        30        31        32        33        34 
## 10.025649 10.376311 10.548237 10.035214  7.902594  7.443810 11.615233 13.035030 
##        35 
## 13.085574 
## 
## Variances on ancestral states:
##       19       20       21       22       23       24       25       26 
## 0.791472 0.625647 0.708906 0.805417 0.324475 0.124102 0.569205 0.374904 
##       27       28       29       30       31       32       33       34 
## 0.565300 0.631001 0.203344 0.607798 0.415969 0.106384 0.645412 0.022841 
##       35 
## 0.013746 
## 
## Lower & upper 95% CIs:
##        lower     upper
## 19  8.273994 11.761411
## 20  8.381621 11.482257
## 21  7.999788 11.300293
## 22  6.440819  9.958825
## 23 10.231389 12.464327
## 24 10.410138 11.791078
## 25  8.522571 11.480041
## 26  8.855140 11.255334
## 27  8.551994 11.499304
## 28  8.819374 11.933247
## 29  9.664401 11.432073
## 30  8.507171 11.563257
## 31  6.638479  9.166709
## 32  6.804526  8.083093
## 33 10.040618 13.189848
## 34 12.738812 13.331248
## 35 12.855776 13.315372
## 
## 
## [[3]]
## Ancestral character estimates using fastAnc:
##        19        20        21        22        23        24        25        26 
##  9.982826  9.895192  9.622439  8.185941 11.344927 11.099976  9.956656 10.049975 
##        27        28        29        30        31        32        33        34 
##  9.976612 10.362527 10.546231  9.975827  7.893569  7.442657 11.416601 12.826492 
##        35 
## 12.884052 
## 
## Variances on ancestral states:
##       19       20       21       22       23       24       25       26 
## 0.826539 0.653367 0.740315 0.841102 0.338851 0.129600 0.594424 0.391515 
##       27       28       29       30       31       32       33       34 
## 0.590347 0.658958 0.212353 0.634727 0.434399 0.111097 0.674007 0.023853 
##       35 
## 0.014355 
## 
## Lower & upper 95% CIs:
##        lower     upper
## 19  8.200908 11.764744
## 20  8.310902 11.479483
## 21  7.936024 11.308853
## 22  6.388393  9.983489
## 23 10.203993 12.485861
## 24 10.394376 11.805577
## 25  8.445518 11.467795
## 26  8.823580 11.276370
## 27  8.470665 11.482559
## 28  8.771473 11.953581
## 29  9.643027 11.449434
## 30  8.414300 11.537354
## 31  6.601753  9.185384
## 32  6.789365  8.095949
## 33  9.807481 13.025721
## 34 12.523783 13.129201
## 35 12.649219 13.118886
## 
## 
## [[4]]
## Ancestral character estimates using fastAnc:
##        19        20        21        22        23        24        25        26 
## 10.061353  9.977930  9.684587  8.217195 11.351527 11.101398 10.057187 10.061822 
##        27        28        29        30        31        32        33        34 
## 10.087022 10.393562 10.550748 10.109541  7.913891  7.445252 11.863836 13.237427 
##        35 
## 13.288748 
## 
## Variances on ancestral states:
##       19       20       21       22       23       24       25       26 
## 0.728283 0.575697 0.652309 0.741115 0.298570 0.114194 0.523761 0.344973 
##       27       28       29       30       31       32       33       34 
## 0.520169 0.580623 0.187110 0.559273 0.382759 0.097890 0.593884 0.021017 
##       35 
## 0.012649 
## 
## Lower & upper 95% CIs:
##        lower     upper
## 19  8.388699 11.734008
## 20  8.490785 11.465075
## 21  8.101580 11.267594
## 22  6.529869  9.904520
## 23 10.280553 12.422502
## 24 10.439064 11.763732
## 25  8.638709 11.475666
## 26  8.910628 11.213017
## 27  8.673417 11.500628
## 28  8.900069 11.887056
## 29  9.702927 11.398569
## 30  8.643764 11.575319
## 31  6.701287  9.126495
## 32  6.832019  8.058486
## 33 10.353384 13.374287
## 34 12.953280 13.521575
## 35 13.068314 13.509182  
  # WARNING: All values are log-converted (ln)  
 
 
 
 Tree with all elephant bird egg specimens (average, minimum, and
maximum) 
 
 PGLS regressions to estimate egg mass and body mass of eggshell
specimens 
 
 Dataset for all eggs 
 
  dataeggall<-cbind(c(rep("Neognathae",45),rep("Palaeognathae",24)),
                  rbind(datalitt[,c(1,2)],EBeggall),
                  c(datalitt$Bodymass, rep(NA, 10)),
                  c(datalitt$Eggmass, rep(NA, 10)))
colnames(dataeggall)<-c("Group",colnames(datalitt))  
 
 Estimates for body mass (using regression compiled at line 734) 
 
  # Estimates based on the OU model
massestim<-exp((log(EBeggall[,2])-OUBM$coefficients[1])/
                              OUBM$coefficients[2])

dataeggall[c(60:69),4]<-massestim

# Some of the estimates can be hard to differentiate from species with actual body mass values
# If you want to see them, use this line of code:
dataeggall[c(60:69),1]<-"Predicted"

# Plot the regression with the estimates on the regression line
ggplot(dataeggall, aes(log(Bodymass), log(Thickness), color=Group)) +
  geom_point(size=5) +
  geom_text(aes(label=Species),hjust=-0.1, vjust=0.4) +
  xlab("ln body mass (kg)") +
  ylab("ln eggshell thickness (µm)") +
  geom_abline(intercept=OUBM$coefficients[1], slope=OUBM$coefficients[2],
              colour="skyblue2", size=1.3) +
  theme(panel.background = element_rect(fill="black")) +
  scale_color_manual(values=wes_palette("Zissou1")[c(1,3,5)])  
   
  # If you want to look at the values:
dataeggall[c(60:69),c(2:4)]  
 
 
 
 
 Estimates for egg mass (using regression compiled at line 830) 
 
  # Estimates based on the OU model
massestim<-exp((log(EBeggall[,2])-lambdaEM$coefficients[1])/
                              lambdaEM$coefficients[2])

dataeggall[c(60:69),5]<-massestim

# Some of the estimates can be hard to differentiate from species with actual body mass values
# If you want to see them, use this line of code:
dataeggall[c(60:69),1]<-"Predicted"

# Plot the regression with the estimates on the regression line
ggplot(dataeggall, aes(log(Eggmass), log(Thickness), color=Group)) +
  geom_point(size=5) +
  geom_text(aes(label=Species),hjust=-0.1, vjust=0.4) +
  xlab("ln egg mass (g)") +
  ylab("ln eggshell thickness (µm)") +
  geom_abline(intercept=lambdaEM$coefficients[1], slope=lambdaEM$coefficients[2],
              colour="skyblue2", size=1.3) +
  theme(panel.background = element_rect(fill="black")) +
  scale_color_manual(values=wes_palette("Zissou1")[c(1,3,5)])  
   
  # If you want to look at the values:
dataeggall[c(60:69),c(2,3,5)]  
 
 
 
 
 
 Ancestral reconstructions with mass estimates from the
regressions 
 
 For eggshell thickness, egg mass, and body mass 
 
  datapaleggall<-dataeggall[c(46:69),]
rownames(datapaleggall)<-datapaleggall$Species
treepaleggall<-drop.tip(treealleggs, setdiff(treealleggs$tip.label,
                                             datapaleggall$Species))
datapaleggall<-ReorderData(treepaleggall, datapaleggall)

# Phylogenetic signal (Pagel's lambda) of each variable
var=list(); phy=list()
for (i in 3:5) {
  var<-log(datapaleggall[,i]); names(var)<-datapaleggall$Species
  phy[[i]]<-phylosig(treepaleggall, var, method="lambda", test=TRUE)
}
phy # Highly significant for all three traits  
  ## [[1]]
## NULL
## 
## [[2]]
## NULL
## 
## [[3]]
## 
## Phylogenetic signal lambda : 0.914125 
## logL(lambda) : -16.1707 
## LR(lambda=0) : 23.3641 
## P-value (based on LR test) : 1.3406e-06 
## 
## 
## [[4]]
## 
## Phylogenetic signal lambda : 0.89792 
## logL(lambda) : -40.165 
## LR(lambda=0) : 24.2863 
## P-value (based on LR test) : 8.30279e-07 
## 
## 
## [[5]]
## 
## Phylogenetic signal lambda : 0.882331 
## logL(lambda) : -33.3425 
## LR(lambda=0) : 20.0813 
## P-value (based on LR test) : 7.42169e-06  
  # Check best evolutionary model
var=list(); fit=list(); mod=list()
for (i in 3:5) {
  var<-log(datapaleggall[,i]); names(var)<-datapaleggall$Species
  for (m in 1:length(models)) {
    fit[[m]]=fitContinuous(treepaleggall, var, model=models[m], ncores=2)
  }
  mod[[i]]<-modSel.geiger(fit[[1]],fit[[2]],fit[[3]],fit[[4]],fit[[5]],fit[[6]])
}
mod # lambda is the best fit for all three traits.  
  ## [[1]]
## NULL
## 
## [[2]]
## NULL
## 
## [[3]]
##          K    logLik     AICc deltaAICc Weight Evidence ratio
## fit[[5]] 3 -16.17070 39.54140  0.000000 0.9929         1.0000
## fit[[2]] 3 -21.14858 49.49717  9.955764 0.0068       145.1666
## fit[[4]] 3 -24.84527 56.89054 17.349137 0.0002      5852.1752
## fit[[1]] 2 -27.29814 59.16771 19.626308 0.0001     18272.5318
## fit[[6]] 2 -27.85273 60.27689 20.735489 0.0000     31816.6312
## fit[[3]] 3 -27.29828 61.79655 22.255152 0.0000     68021.2938
## 
## [[4]]
##          K    logLik      AICc deltaAICc Weight Evidence ratio
## fit[[5]] 3 -40.16498  87.52997   0.00000 0.9956         1.0000
## fit[[2]] 3 -45.61491  98.42983  10.89986 0.0043       232.7418
## fit[[4]] 3 -50.13301 107.46601  19.93604 0.0000     21333.2479
## fit[[6]] 2 -52.30813 109.18769  21.65772 0.0000     50456.1914
## fit[[1]] 2 -52.77341 110.11826  22.58829 0.0000     80349.8173
## fit[[3]] 3 -52.77356 112.74712  25.21715 0.0000    299112.1647
## 
## [[5]]
##          K    logLik      AICc deltaAICc Weight Evidence ratio
## fit[[5]] 3 -33.34248  73.88496  0.000000 0.9906         1.0000
## fit[[2]] 3 -38.02551  83.25102  9.366061 0.0092       108.0972
## fit[[6]] 2 -43.38315  91.33773 17.452777 0.0002      6163.4301
## fit[[4]] 3 -43.75095  94.70190 20.816948 0.0000     33139.2623
## fit[[1]] 2 -46.46809  97.50762 23.622661 0.0000    134770.7500
## fit[[3]] 3 -46.46824 100.13648 26.251525 0.0000    501702.3416  
  # Plots
dataplot=list(); fit=list(); obj=list()
for (i in 3:5) {
  dataplot[[i]]<-as.vector(log(datapaleggall[,i]))
  names(dataplot[[i]])<-datapaleggall$Species
  fit[[i]]<-fastAnc(treepaleggall, dataplot[[i]], vars=TRUE, CI=TRUE)
  obj[[i]]<-setMap(contMap(treepaleggall, dataplot[[i]]),
                   colors=rev(brewer.pal(10,"Spectral")))
}  
 
 Plots for three traits (all plots generated at once) 
 
  # Just the trees
for (i in 3:5) {
  plot(obj[[i]])
  title(paste('Ancestral state reconstruction for', colnames(datapaleggall)[i]))
}  
     
  # Trees with values mapped on branches
for (i in 3:5) {
  plot(setMap(obj[[i]], colors=rev(brewer.pal(10,"Spectral"))), offset=2);
  nodelabels(as.factor(round(exp(fit[[i]]$ace), digits=0)), bg="white", cex=0.6,
             font=2);
  tiplabels(as.factor(round(exp(dataplot[[i]]), digits=0)), bg="white",
            cex=0.6, font=2, offset=5);
  title(paste('Ancestral state reconstruction for', colnames(datapaleggall)[i]))
}  
     
  # The color scale on the tree was generated with log-converted values, but the values displayed on these plots for both tips and nodes are the raw values.
# I did not include digits after the decimal point to make the figures easier to read. If you want to display more digits, just change the value of argument 'digits' in the above code.

fit # Ancestral state values for each trait, if you need them  
  ## [[1]]
## NULL
## 
## [[2]]
## NULL
## 
## [[3]]
## Ancestral character estimates using fastAnc:
##       25       26       27       28       29       30       31       32 
## 6.790900 6.741275 6.616750 6.044972 7.201995 7.012412 6.757652 6.805856 
##       33       34       35       36       37       38       39       40 
## 6.762141 6.839329 6.875882 6.762808 6.111951 5.975431 7.220980 7.039190 
##       41       42       43       44       45       46       47 
## 7.025408 7.800007 8.035280 8.021031 7.746869 7.690612 7.567525 
## 
## Variances on ancestral states:
##       25       26       27       28       29       30       31       32 
## 0.590790 0.466979 0.529186 0.601285 0.242243 0.092651 0.424788 0.279890 
##       33       34       35       36       37       38       39       40 
## 0.421840 0.471071 0.151810 0.453475 0.310543 0.079423 0.480697 0.056818 
##       41       42       43       44       45       46       47 
## 0.013614 0.055481 0.008692 0.006572 0.041312 0.009272 0.004295 
## 
## Lower & upper 95% CIs:
##       lower    upper
## 25 5.284388 8.297412
## 26 5.401892 8.080658
## 27 5.190945 8.042555
## 28 4.525138 7.564806
## 29 6.237320 8.166671
## 30 6.415817 7.609008
## 31 5.480206 8.035097
## 32 5.768925 7.842787
## 33 5.489137 8.035145
## 34 5.494091 8.184567
## 35 6.112211 7.639553
## 36 5.442933 8.082684
## 37 5.019713 7.204189
## 38 5.423063 6.527798
## 39 5.862066 8.579893
## 40 6.571996 7.506385
## 41 6.796718 7.254098
## 42 7.338340 8.261675
## 43 7.852548 8.218012
## 44 7.862136 8.179926
## 45 7.348491 8.145247
## 46 7.501884 7.879340
## 47 7.439068 7.695982
## 
## 
## [[4]]
## Ancestral character estimates using fastAnc:
##        25        26        27        28        29        30        31        32 
##  9.954676  9.865533  9.600160  8.174737 11.342560 11.099467  9.920618 10.045728 
##        33        34        35        36        37        38        39        40 
##  9.937032 10.351401 10.544612  9.927893  7.886284  7.441727 11.252337 10.712042 
##        41        42        43        44        45        46        47 
## 10.671351 12.940554 13.629344 13.587598 12.784747 12.619774 12.259224 
## 
## Variances on ancestral states:
##       25       26       27       28       29       30       31       32 
## 4.936441 3.901920 4.421697 5.024133 2.024097 0.774157 3.549388 2.338668 
##       33       34       35       36       37       38       39       40 
## 3.524751 3.936107 1.268474 3.789086 2.594796 0.663629 4.016543 0.474749 
##       41       42       43       44       45       46       47 
## 0.113753 0.463583 0.072627 0.054915 0.345191 0.077471 0.035891 
## 
## Lower & upper 95% CIs:
##        lower     upper
## 25  5.599928 14.309424
## 26  5.993890 13.737175
## 27  5.478705 13.721615
## 28  3.781480 12.567994
## 29  8.554053 14.131068
## 30  9.374938 12.823996
## 31  6.228014 13.613222
## 32  7.048358 13.043098
## 33  6.257265 13.616799
## 34  6.462835 14.239968
## 35  8.337131 12.752092
## 36  6.112640 13.743146
## 37  4.729043 11.043524
## 38  5.845044  9.038411
## 39  7.324239 15.180435
## 40  9.361562 12.062522
## 41 10.010295 11.332406
## 42 11.606051 14.275057
## 43 13.101136 14.157551
## 44 13.128294 14.046902
## 45 11.633188 13.936305
## 46 12.074233 13.165314
## 47 11.887904 12.630544
## 
## 
## [[5]]
## Ancestral character estimates using fastAnc:
##       25       26       27       28       29       30       31       32 
## 6.359824 6.310124 6.101005 4.906608 7.644292 7.460662 6.378499 6.373346 
##       33       34       35       36       37       38       39       40 
## 6.404562 6.359449 6.382931 6.451646 5.893175 5.845893 7.311688 6.825093 
##       41       42       43       44       45       46       47 
## 6.790863 8.540214 9.067174 9.034957 8.418847 8.290155 8.012536 
## 
## Variances on ancestral states:
##       25       26       27       28       29       30       31       32 
## 2.918883 2.307179 2.614519 2.970735 1.196835 0.457753 2.098729 1.382838 
##       33       34       35       36       37       38       39       40 
## 2.084161 2.327393 0.750040 2.240461 1.534285 0.392399 2.374955 0.280716 
##       41       42       43       44       45       46       47 
## 0.067261 0.274113 0.042944 0.032471 0.204109 0.045808 0.021222 
## 
## Lower & upper 95% CIs:
##       lower     upper
## 25 3.011215  9.708433
## 26 3.333002  9.287246
## 27 2.931788  9.270222
## 28 1.528387  8.284829
## 29 5.500053  9.788531
## 30 6.134575  8.786748
## 31 3.539049  9.217949
## 32 4.068500  8.678191
## 33 3.574983  9.234140
## 34 3.369313  9.349585
## 35 4.685476  8.080386
## 36 3.517885  9.385407
## 37 3.465397  8.320954
## 38 4.618114  7.073672
## 39 4.291154 10.332222
## 40 5.786634  7.863553
## 41 6.282541  7.299186
## 42 7.514040  9.566388
## 43 8.661006  9.473342
## 44 8.681773  9.388142
## 45 7.533350  9.304345
## 46 7.870658  8.709651
## 47 7.727007  8.298065  
  # WARNING: All values are log-converted (ln)  
 
 
 
 
 References 
 
 Birchard, G.F., Deeming, D.C., 2009. Avian eggshell thickness:
scaling and maximum body mass in birds.  Journal of Zoology  279,
95–101.  https://doi.org/10.1111/j.1469-7998.2009.00596.x  
 
 Body, D.R., Reid, B., 1987. The lipid, fatty acid and amino acid
composition of ratite eggs from three different species of kiwis
 Apteryx australis mantelli ,  A. haasti  and  A. oweni 
bred in captivity on the same diet.  Biochemical Systematics and
Ecology  15, 625–628.  https://doi.org/10.1016/0305-1978(87)90115-3]  
 
 Calder, W.A., 1979. The Kiwi and Egg Design: Evolution as a Package
Deal.  BioScience  29, 461–467.  https://doi.org/10.2307/1307538  
 
 Crouch, N.M.A., Clarke, J.A., 2019. Body size evolution in
palaeognath birds is consistent with Neogene cooling-linked gigantism.
 Palaeogeography Palaeoclimatolology Palaeoecology  532, 109224.  https://doi.org/10.1016/j.palaeo.2019.05.046  
 
 Dunning, J.B., 2008.  CRC Handbook of Avian Body Masses, Second
Edition . CRC Press, Boca Raton.  https://doi.org/10.1201/9781420064452  
 
 Gill, B.J., 2022. Thickness histograms of Holocene fossil eggshell
fragments indicate diversity and relative abundance of moas (Aves:
Dinornithiformes) at North Island sites.  New Zealand Journal of
Zoology  49, 143–165.  https://doi.org/10.1080/03014223.2021.1970585  
 
 Gill, B.J., 2006. A catalogue of moa eggs (Aves: Dinornithiformes).
 Records of the Auckland Museum  43, 55–80.  http://www.jstor.org/stable/42905885  
 
 Hansford, J.P., Turvey, S.T., 2018. Unexpected diversity within the
extinct elephant birds (Aves: Aepyornithidae) and a new identity for the
world’s largest bird.  Royal Society Open Science  5, 181295.  https://doi.org/10.1098/rsos.181295  
 
 Hume, J.P., Robertson, C., 2021. Eggs of extinct dwarf island emus
retained large size.  Biology Letters  17, 20210012.  https://doi.org/10.1098/rsbl.2021.0012  
 
 Huynen, L., Gill, B. J., Millar, C. D., & Lambert, D. M., 2010.
Ancient DNA reveals extreme egg morphology and nesting behavior in New
Zealand’s extinct moa.  Proceedings of the National Academy of
Sciences  107, 16201–16206.  https://doi.org/10.1073/pnas.0914096107  
 
 Juang, J.-Y., Chen, P.-Y., Yang, D.-C., Wu, S.-P., Yen, A., Hsieh,
H.-I., 2017. The avian egg exhibits general allometric invariances in
mechanical design.  Scientific Reports  7, 14205.  https://doi.org/10.1038/s41598-017-14552-0  
 
 Legendre, L.J., Clarke, J.A., 2021. Shifts in eggshell thickness are
related to changes in locomotor ecology in dinosaurs.  Evolution 
75, 1415–1430.  https://doi.org/10.1111/evo.14245  
 
 Maurer, C., Russell, D.G.D., Cassey, P., 2010. Interpreting the Lists
and Equations of Egg Dimensions in Schönwetter’s  Handbuch Der
Oologie .  The Auk  127, 940–947.  https://doi.org/10.1525/auk.2010.09260  
 
 Navarro, J.L., Barri, F.R., Maestri, D.M., Labuckas, D.O., Martella,
M.B., 2003. Physical characteristics and chemical composition of Lesser
Rhea ( Pterocnemia pennata ) eggs from farmed populations.
 British Poultry Science  44, 586–590.  https://doi.org/10.1080/00071660310001616237  
 
 Olson, V. A., Turvey, S. T., 2013. The evolution of sexual dimorphism
in New Zealand giant moa ( Dinornis ) and other ratites.
 Proceedings of the Royal Society B: Biological Sciences  280,
20130401.  https://doi.org/10.1098/rspb.2013.0401  
 
 Schönwetter, M., 1960–1992.  Handbuch der Oologie . Akademie
Verlag, Berlin.  https://www.biodiversitylibrary.org/bibliography/61353  
 
 Silyn-Roberts, H., Sharp, R.M., 1985. Preferred orientation of
calcite in the ratite and tinamou eggshells.  Journal of Zoology 
205, 39–52.  https://doi.org/10.1111/j.1469-7998.1985.tb05612.x  
 
 Tyler, C., Simkiss, K., 1959. A Study of the Egg Shells of Ratite
Birds.  Proceedings of the Zoological Society of London  133,
201–243.  https://doi.org/10.1111/j.1469-7998.1959.tb05561.x  
 
 Vieco‐Galvez, D., Castro, I., Morel, P.C.H., Chua, W.H., Loh, M.,
2021. The eggshell structure in  Apteryx ; form, function, and
adaptation.  Ecology and Evolution  11, 3184–3202.  https://doi.org/10.1002/ece3.7266  
 
 Yonezawa, T., Segawa, T., Mori, H., Campos, P.F., Hongoh, Y., Endo,
H., Akiyoshi, A., Kohno, N., Nishida, S., Wu, J., Jin, H., Adachi, J.,
Kishino, H., Kurokawa, K., Nogi, Y., Tanabe, H., Mukoyama, H., Yoshida,
K., Rasoamiaramanana, A., Yamagishi, S., Hayashi, Y., Yoshida, A.,
Koike, H., Akishinonomiya, F., Willerslev, E., Hasegawa, M., 2017.
Phylogenomics and Morphology of Extinct Paleognaths Reveal the Origin
and Evolution of the Ratites.  Current Biology  27, 68–77.  https://doi.org/10.1016/j.cub.2016.10.029  
 
 


 
 

 

 

 

 

 

 

 
 

 
 
