# Supplementary figures and images for: Molecular exploration of fossil eggshell uncovers hidden lineage of giant extinct bird (part 2 of 2)
Source: Nat Commun. 2023 Feb 28;14:914. doi: 10.1038/s41467-023-36405-3 (PMC9974994; doi:10.1038/s41467-023-36405-3)

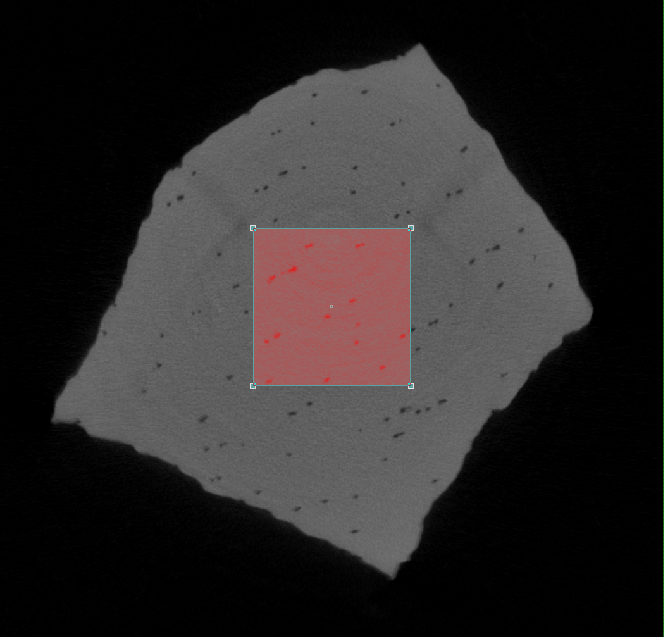

Supplement: Supplementary file 12 — Supplementary Data 12 [file 41467_2023_36405_MOESM12_ESM.zip › Micro_CT_raw_data/Mullerornis/AD2112/Results/ROI Selection.tif]

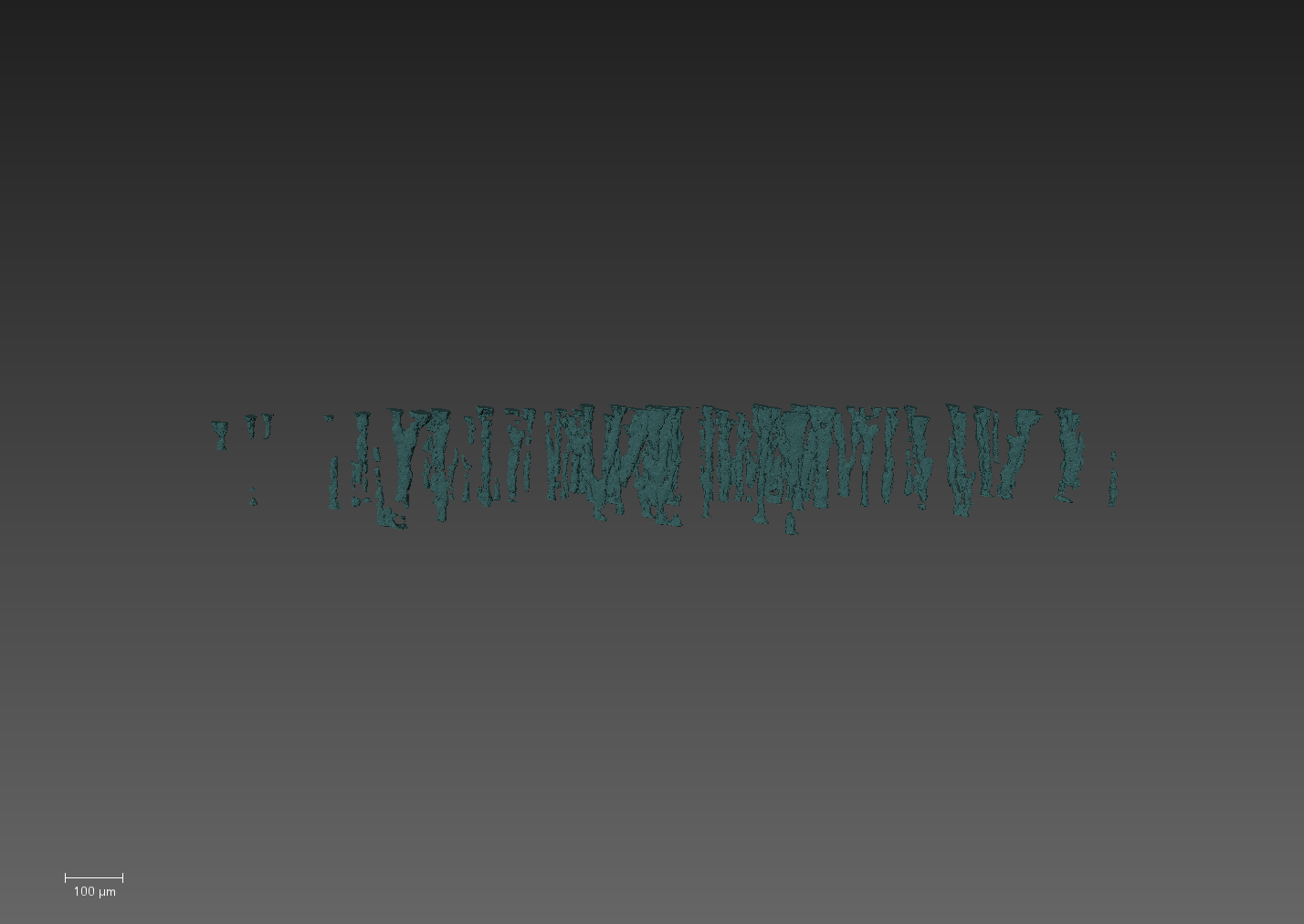

Supplement: Supplementary file 12 — Supplementary Data 12 [file 41467_2023_36405_MOESM12_ESM.zip › Micro_CT_raw_data/Mullerornis/AD2112/Results/Pore structure2.tif]

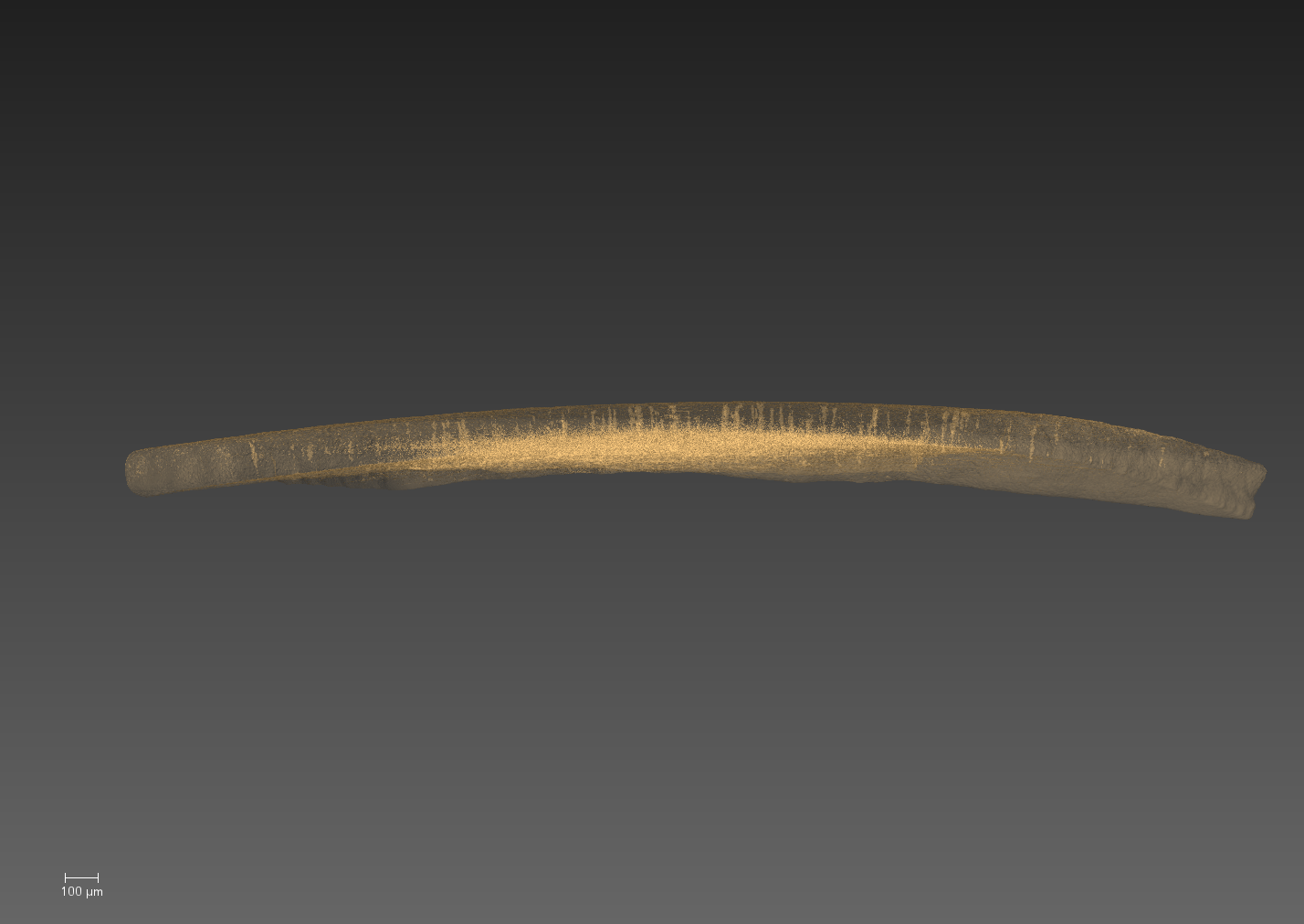

Supplement: Supplementary file 12 — Supplementary Data 12 [file 41467_2023_36405_MOESM12_ESM.zip › Micro_CT_raw_data/Mullerornis/AD2109/Results/snapshot2.tif]

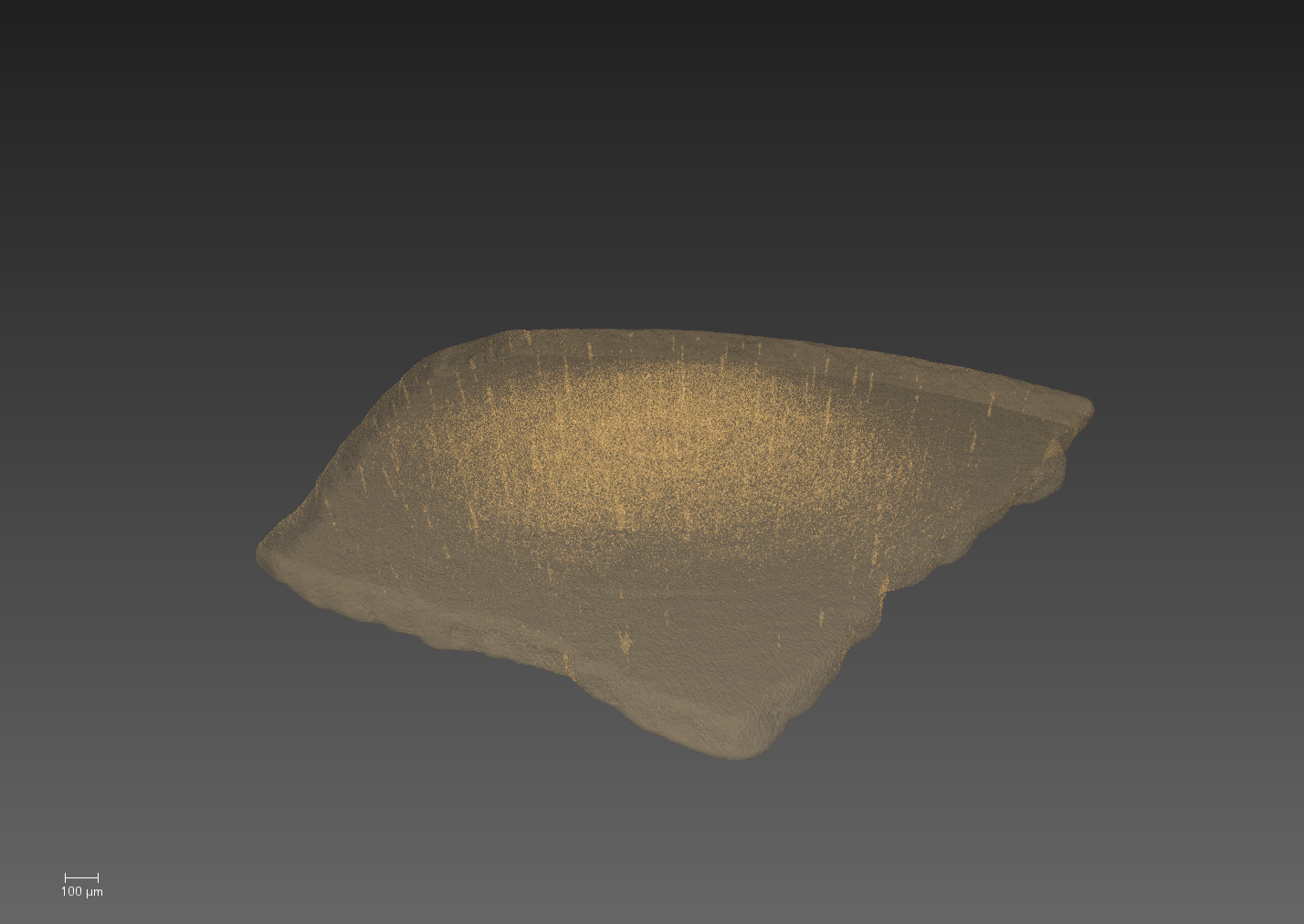

Supplement: Supplementary file 12 — Supplementary Data 12 [file 41467_2023_36405_MOESM12_ESM.zip › Micro_CT_raw_data/Mullerornis/AD2109/Results/snapshot1.tif]

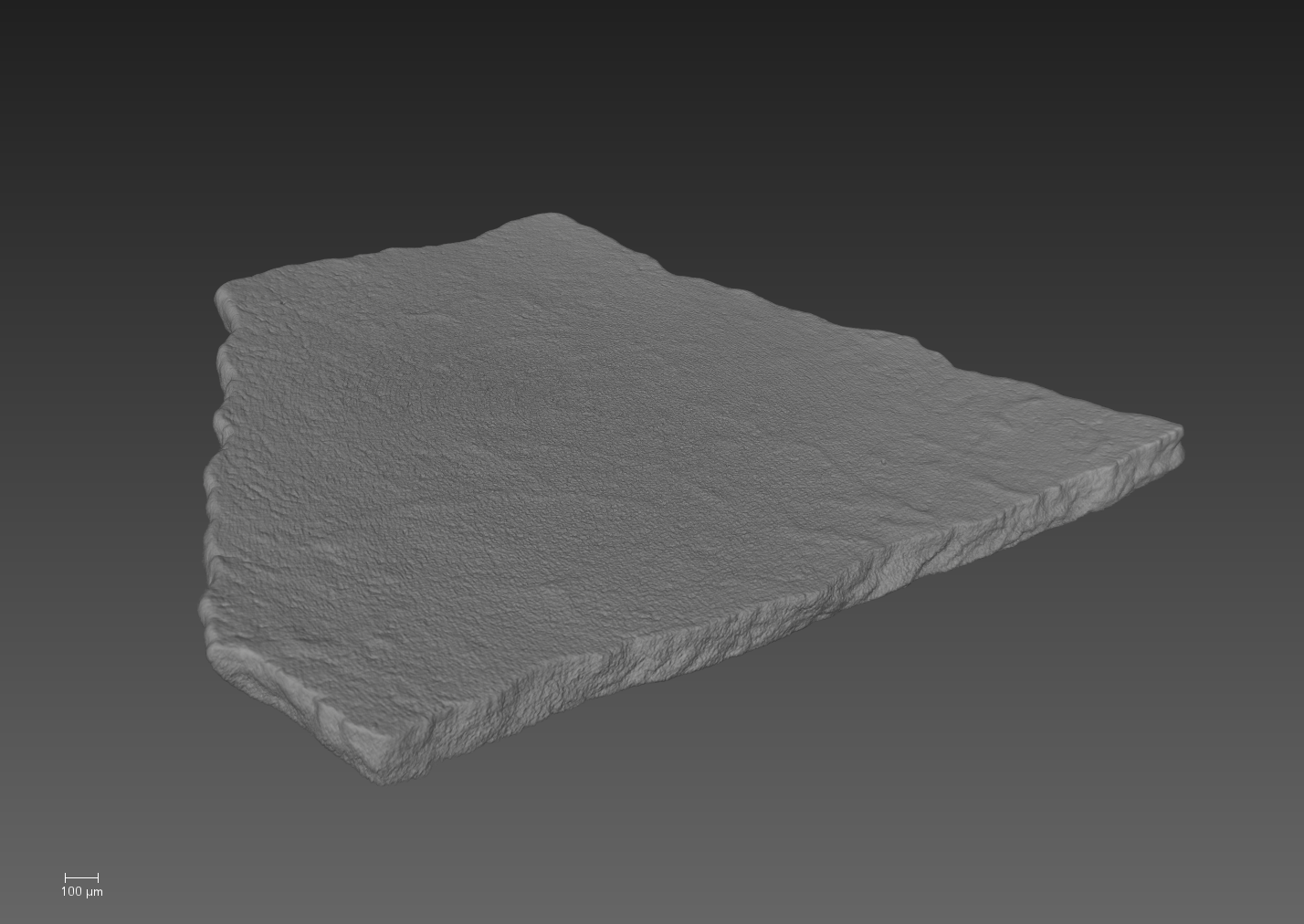

Supplement: Supplementary file 12 — Supplementary Data 12 [file 41467_2023_36405_MOESM12_ESM.zip › Micro_CT_raw_data/Mullerornis/AD2109/Results/Inner surface.tif]

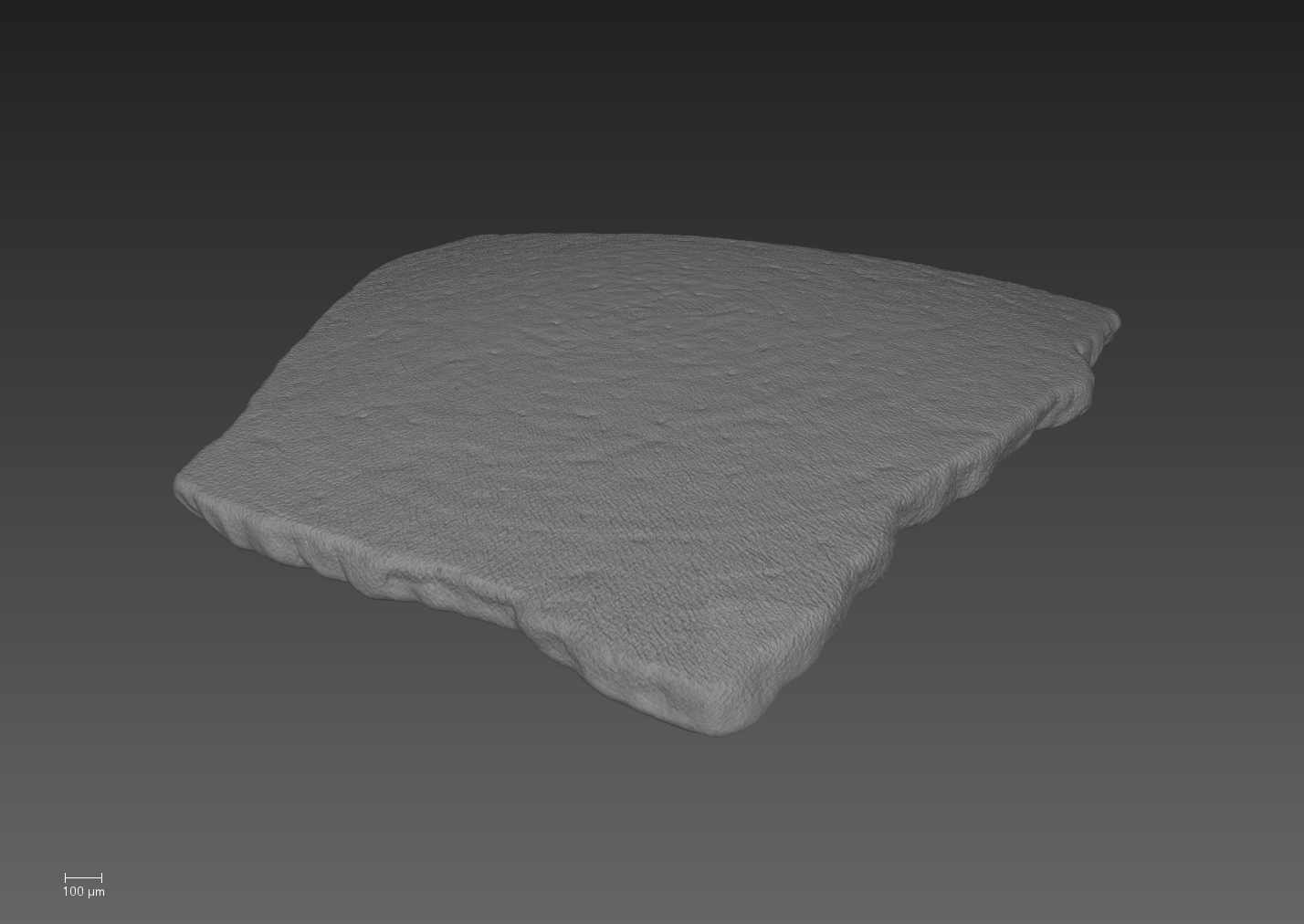

Supplement: Supplementary file 12 — Supplementary Data 12 [file 41467_2023_36405_MOESM12_ESM.zip › Micro_CT_raw_data/Mullerornis/AD2109/Results/Outer surface.tif]

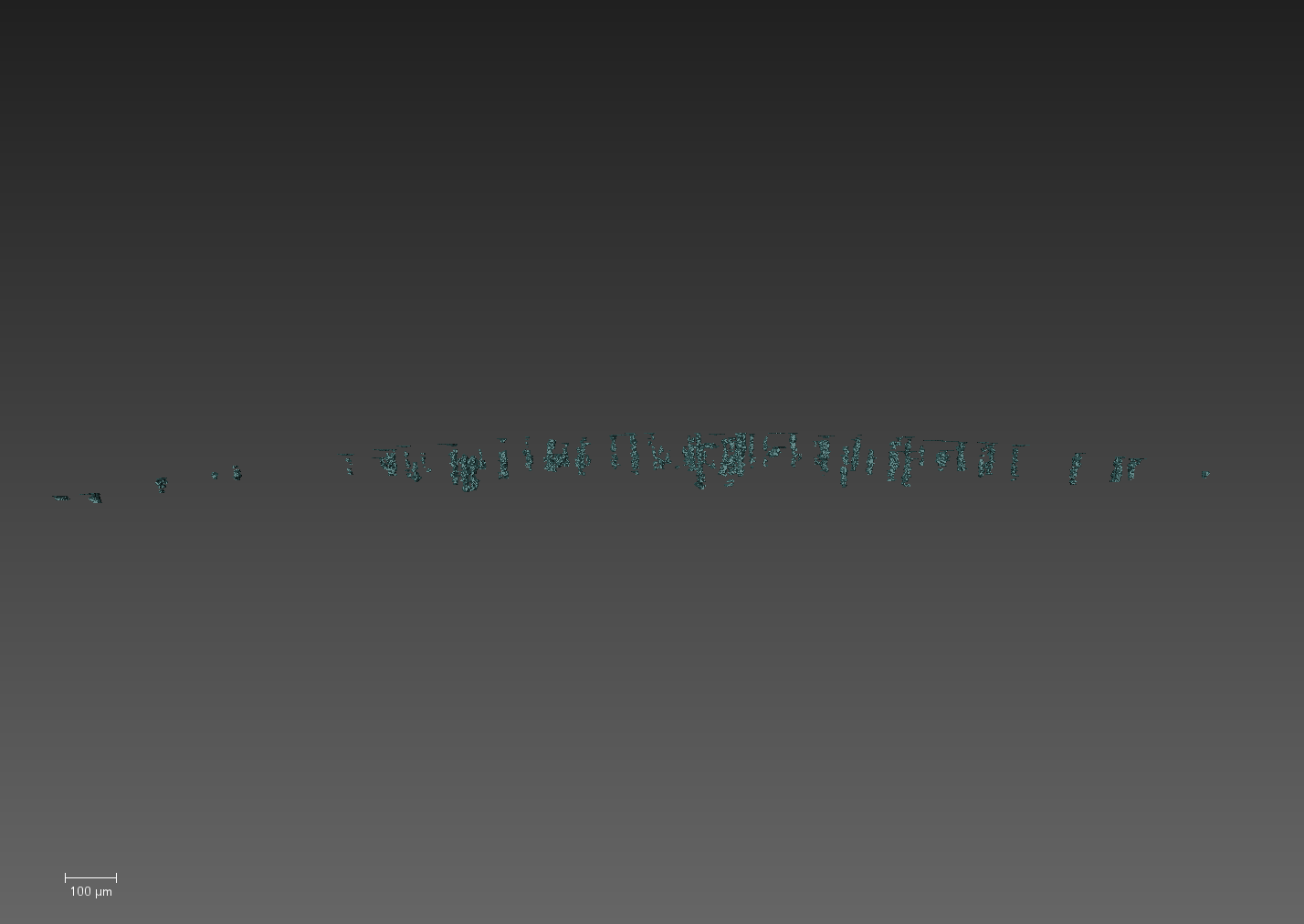

Supplement: Supplementary file 12 — Supplementary Data 12 [file 41467_2023_36405_MOESM12_ESM.zip › Micro_CT_raw_data/Mullerornis/AD2109/Results/Pore structure.tif]

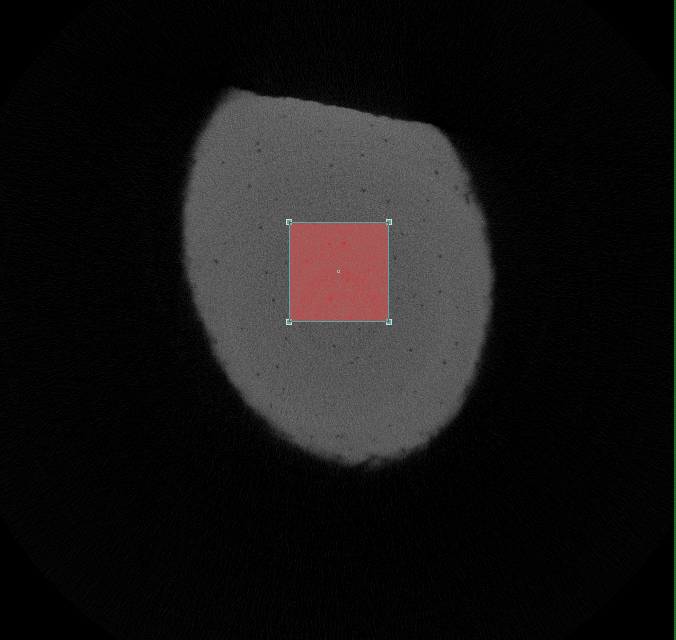

Supplement: Supplementary file 12 — Supplementary Data 12 [file 41467_2023_36405_MOESM12_ESM.zip › Micro_CT_raw_data/Mullerornis/AD2109/Results/ROI Selection.tif]

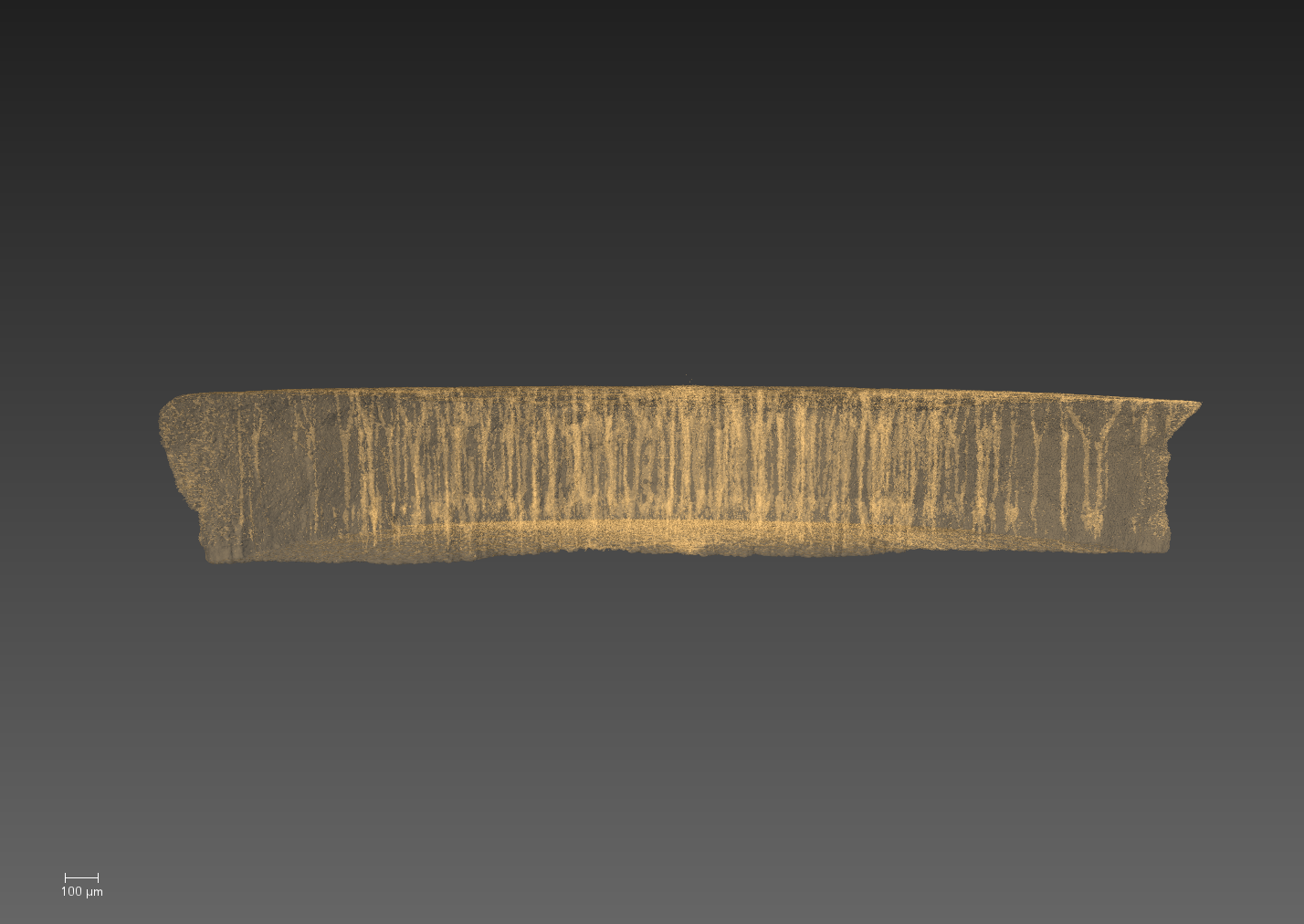

Supplement: Supplementary file 12 — Supplementary Data 12 [file 41467_2023_36405_MOESM12_ESM.zip › Micro_CT_raw_data/Southern_Aepyornis_thick/AD2117/Results/snapshot2.tif]

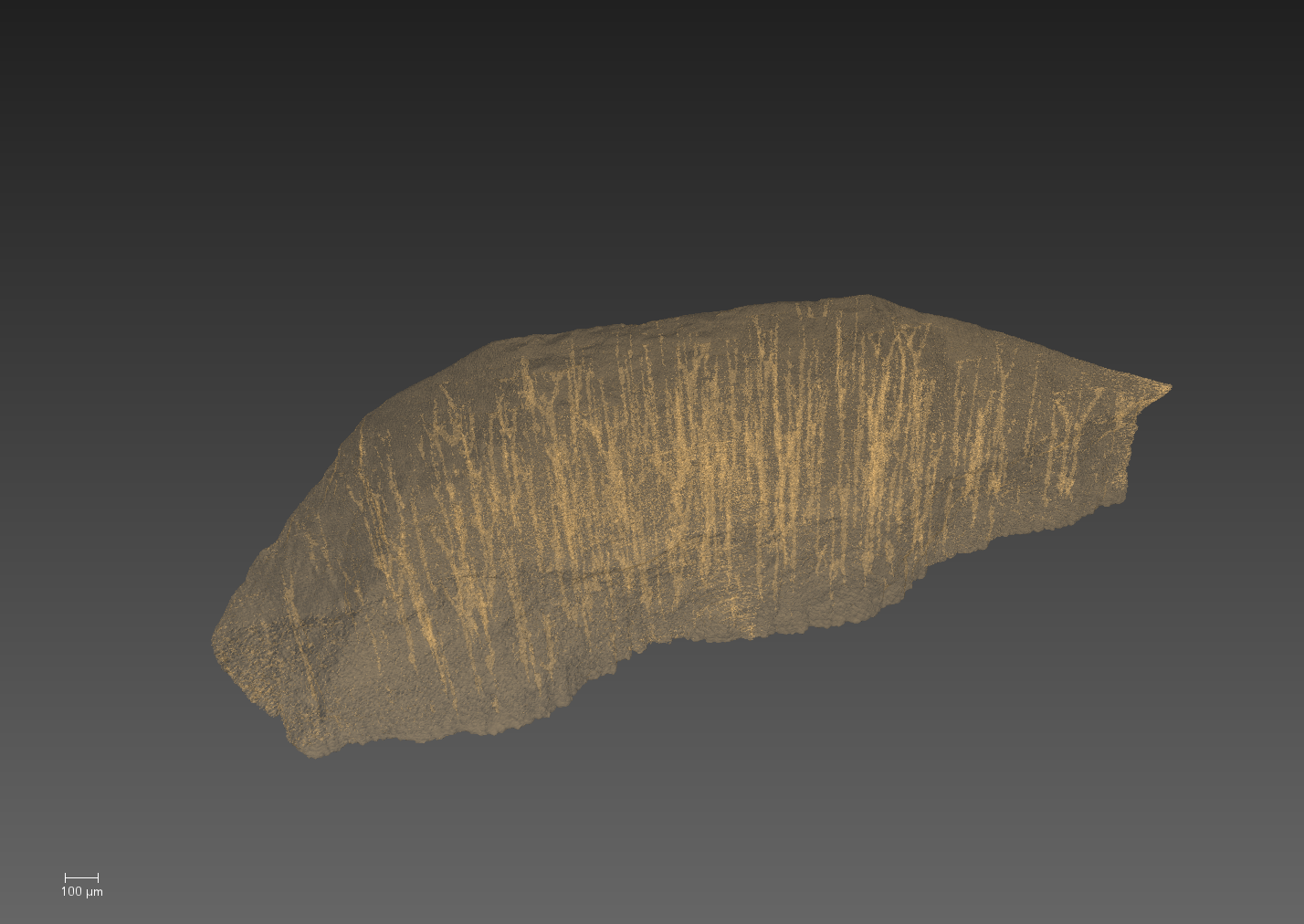

Supplement: Supplementary file 12 — Supplementary Data 12 [file 41467_2023_36405_MOESM12_ESM.zip › Micro_CT_raw_data/Southern_Aepyornis_thick/AD2117/Results/snapshot.tif]

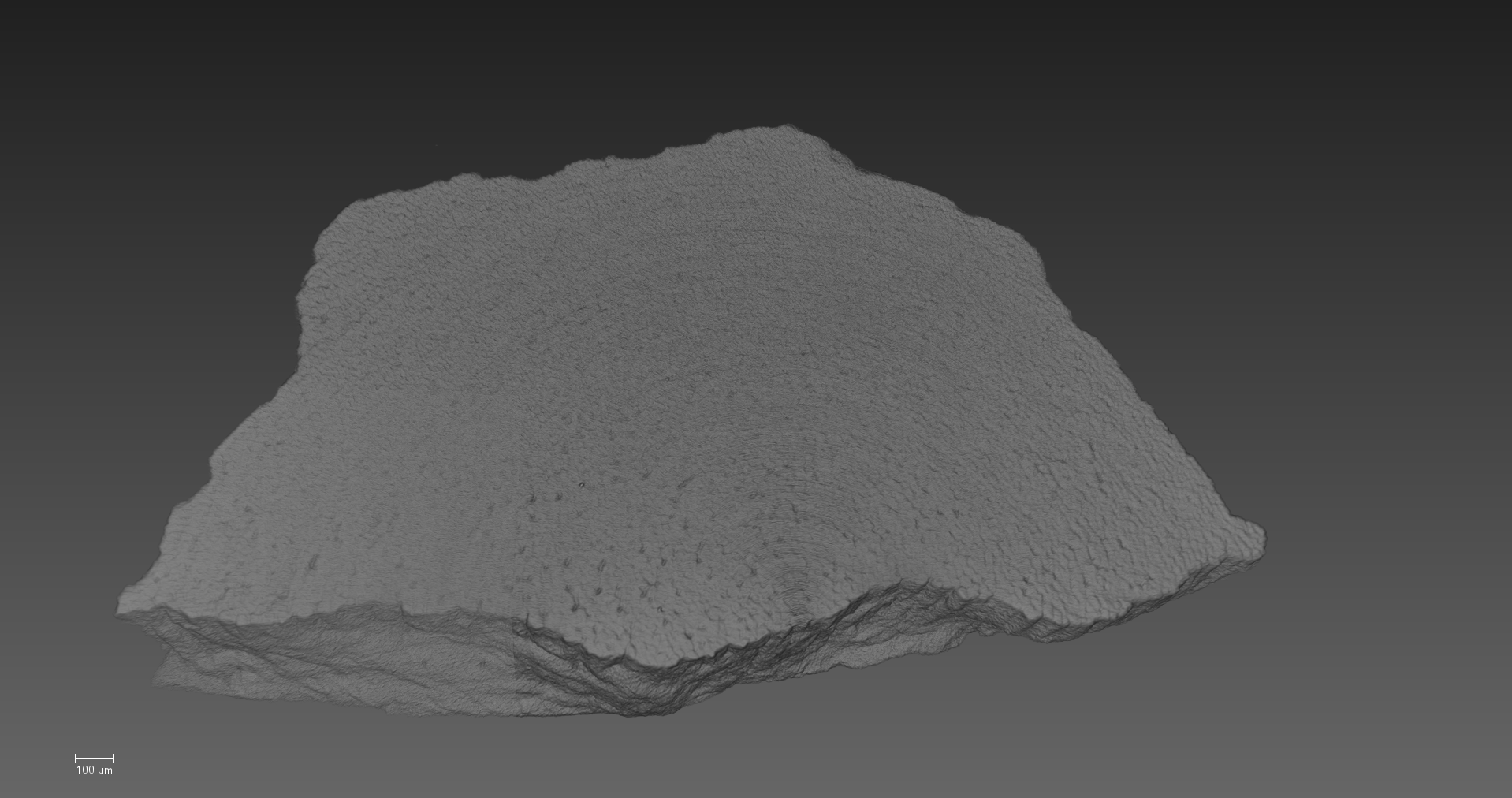

Supplement: Supplementary file 12 — Supplementary Data 12 [file 41467_2023_36405_MOESM12_ESM.zip › Micro_CT_raw_data/Southern_Aepyornis_thick/AD2117/Results/Inner surface.tif]

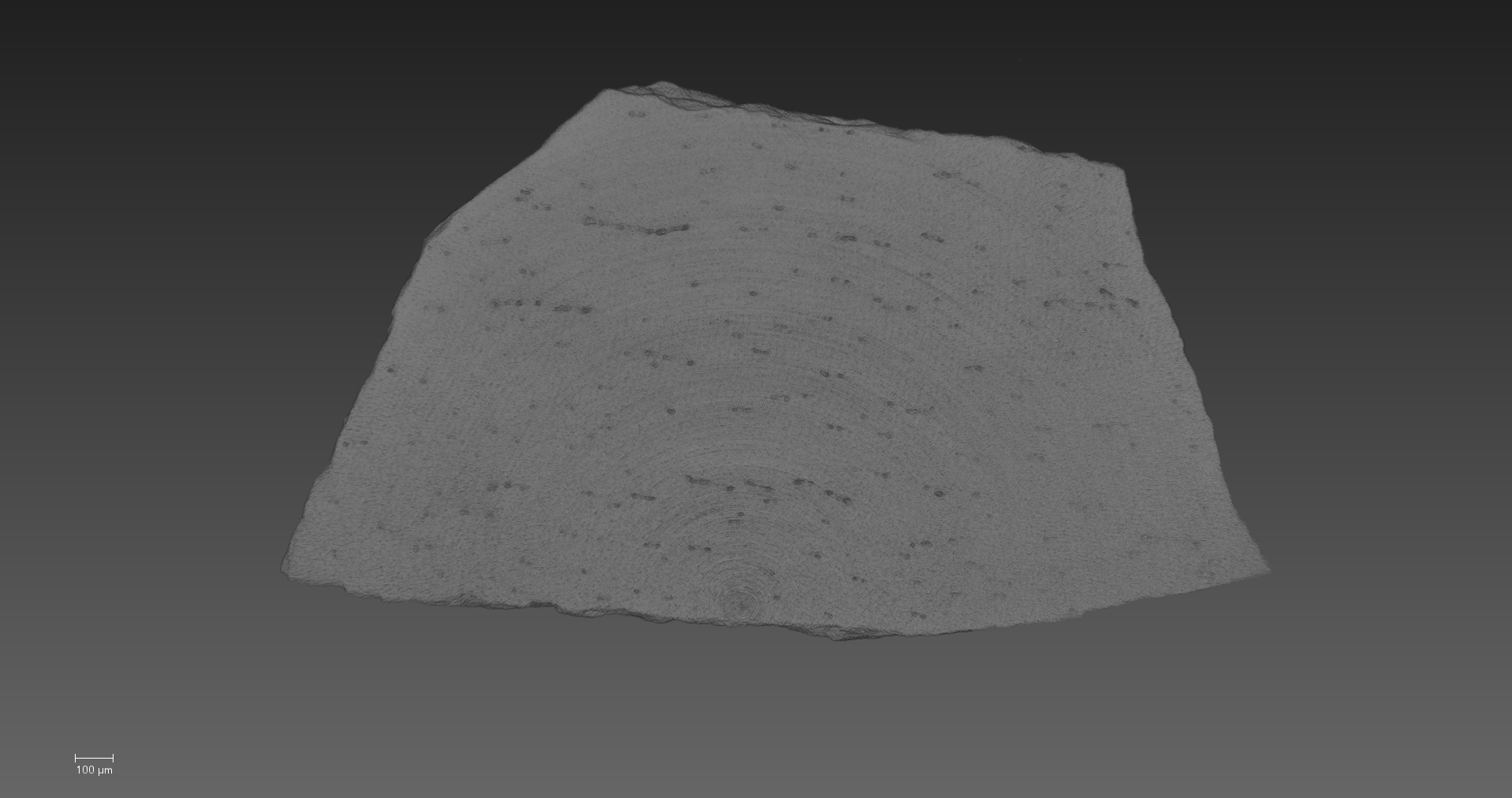

Supplement: Supplementary file 12 — Supplementary Data 12 [file 41467_2023_36405_MOESM12_ESM.zip › Micro_CT_raw_data/Southern_Aepyornis_thick/AD2117/Results/Outer surface.tif]

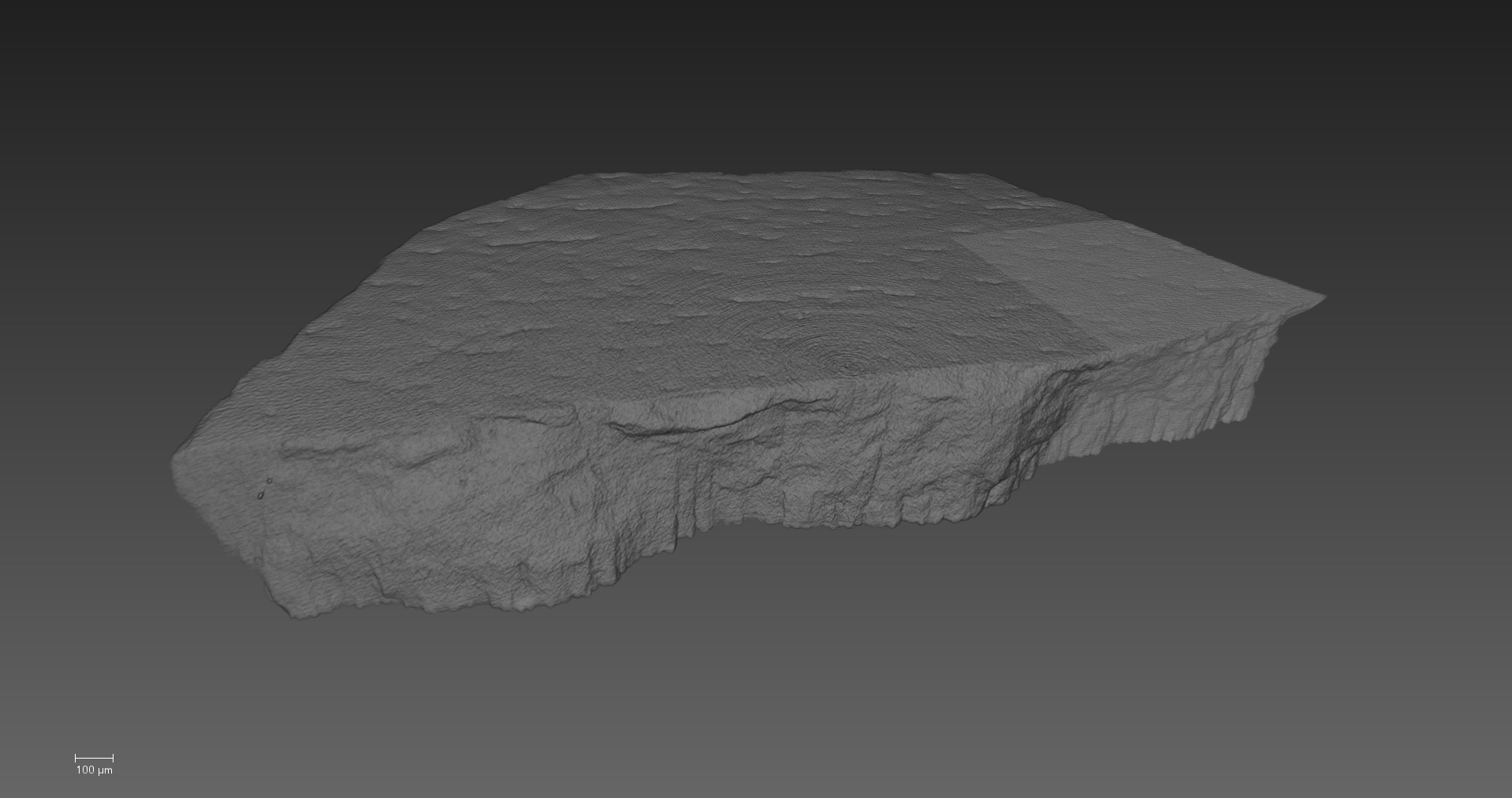

Supplement: Supplementary file 12 — Supplementary Data 12 [file 41467_2023_36405_MOESM12_ESM.zip › Micro_CT_raw_data/Southern_Aepyornis_thick/AD2117/Results/Outer surface2.tif]

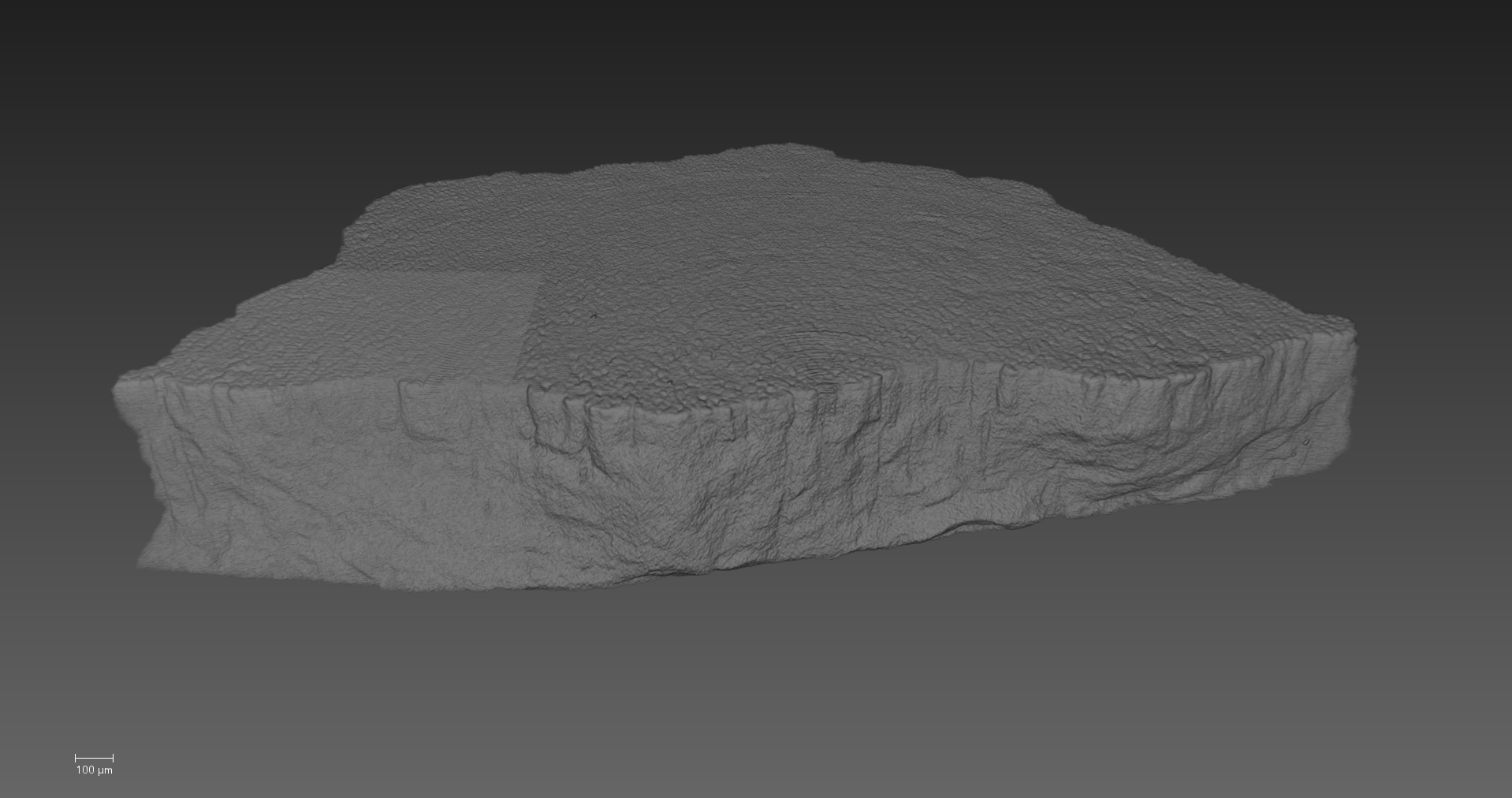

Supplement: Supplementary file 12 — Supplementary Data 12 [file 41467_2023_36405_MOESM12_ESM.zip › Micro_CT_raw_data/Southern_Aepyornis_thick/AD2117/Results/Inner surface2.tif]

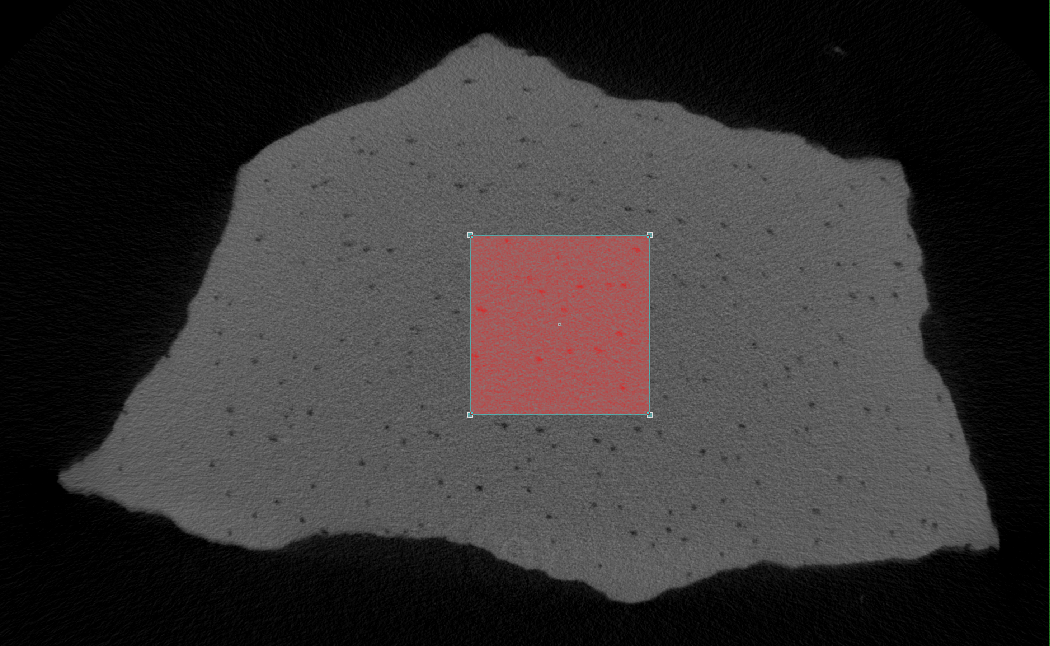

Supplement: Supplementary file 12 — Supplementary Data 12 [file 41467_2023_36405_MOESM12_ESM.zip › Micro_CT_raw_data/Southern_Aepyornis_thick/AD2117/Results/ROI Selection.tif]

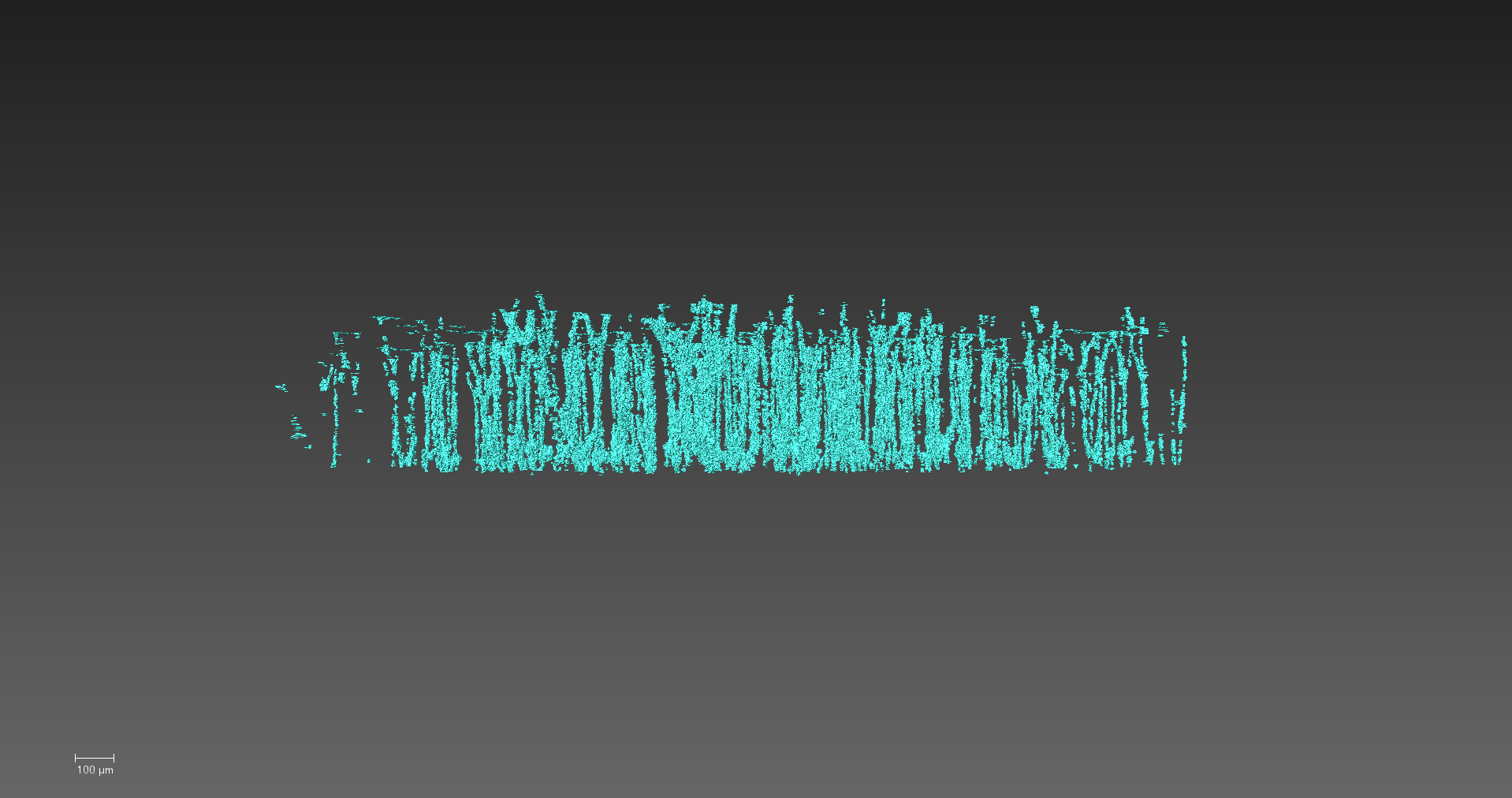

Supplement: Supplementary file 12 — Supplementary Data 12 [file 41467_2023_36405_MOESM12_ESM.zip › Micro_CT_raw_data/Southern_Aepyornis_thick/AD2117/Results/Pore structure1.tif]

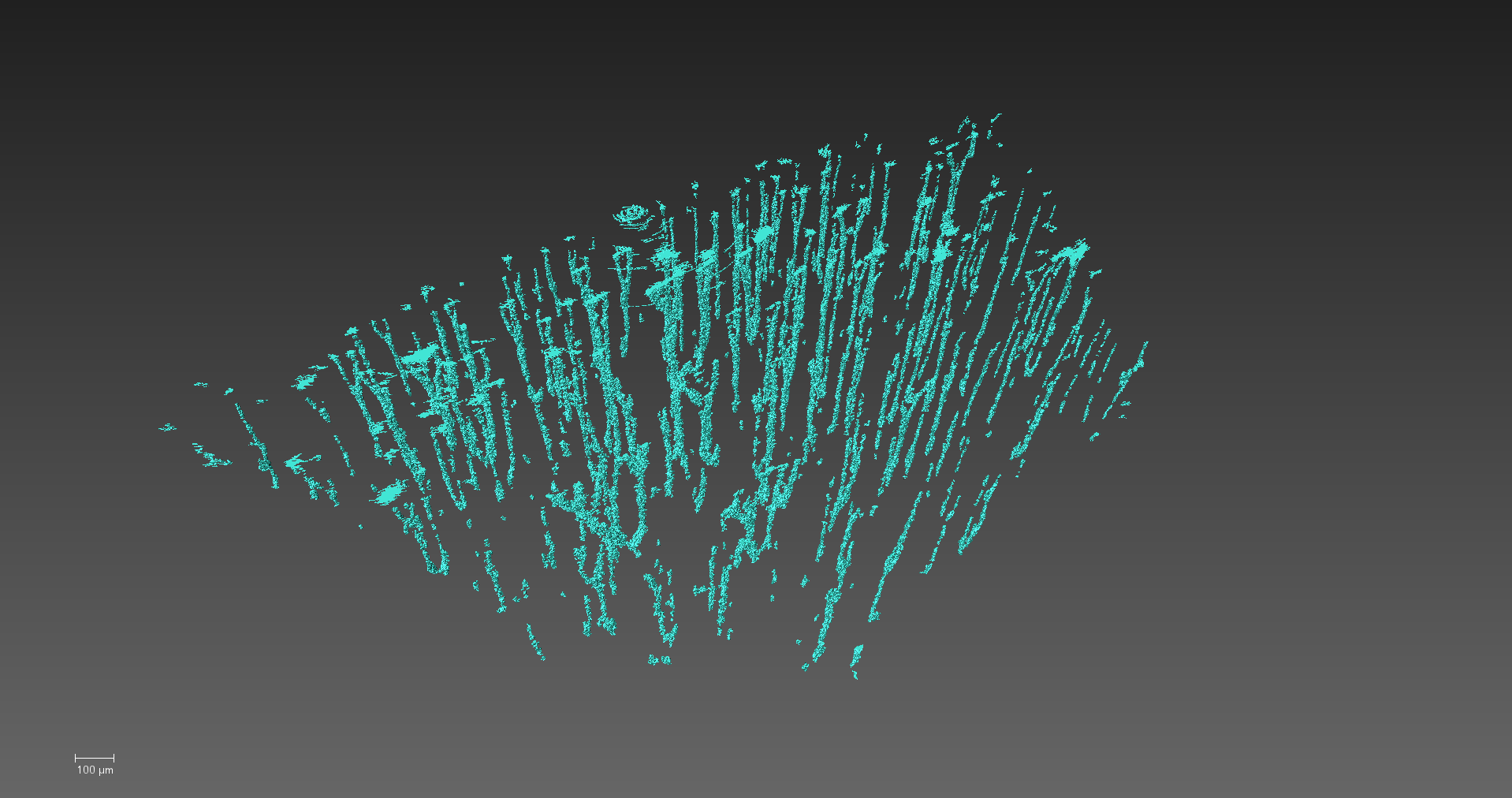

Supplement: Supplementary file 12 — Supplementary Data 12 [file 41467_2023_36405_MOESM12_ESM.zip › Micro_CT_raw_data/Southern_Aepyornis_thick/AD2117/Results/Pore structure2.tif]

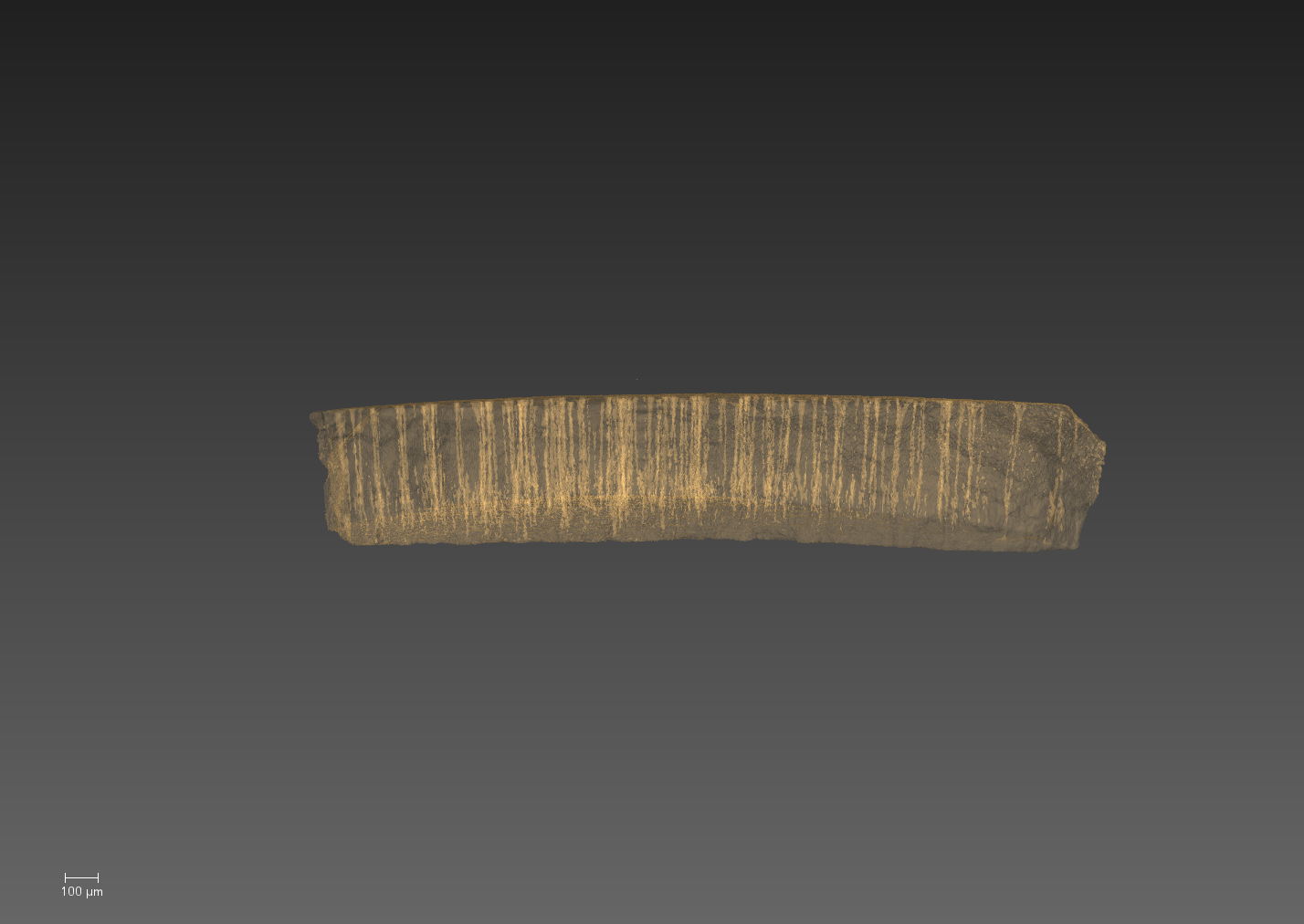

Supplement: Supplementary file 12 — Supplementary Data 12 [file 41467_2023_36405_MOESM12_ESM.zip › Micro_CT_raw_data/Southern_Aepyornis_thick/AD2118/Results/snapshot2.tif]

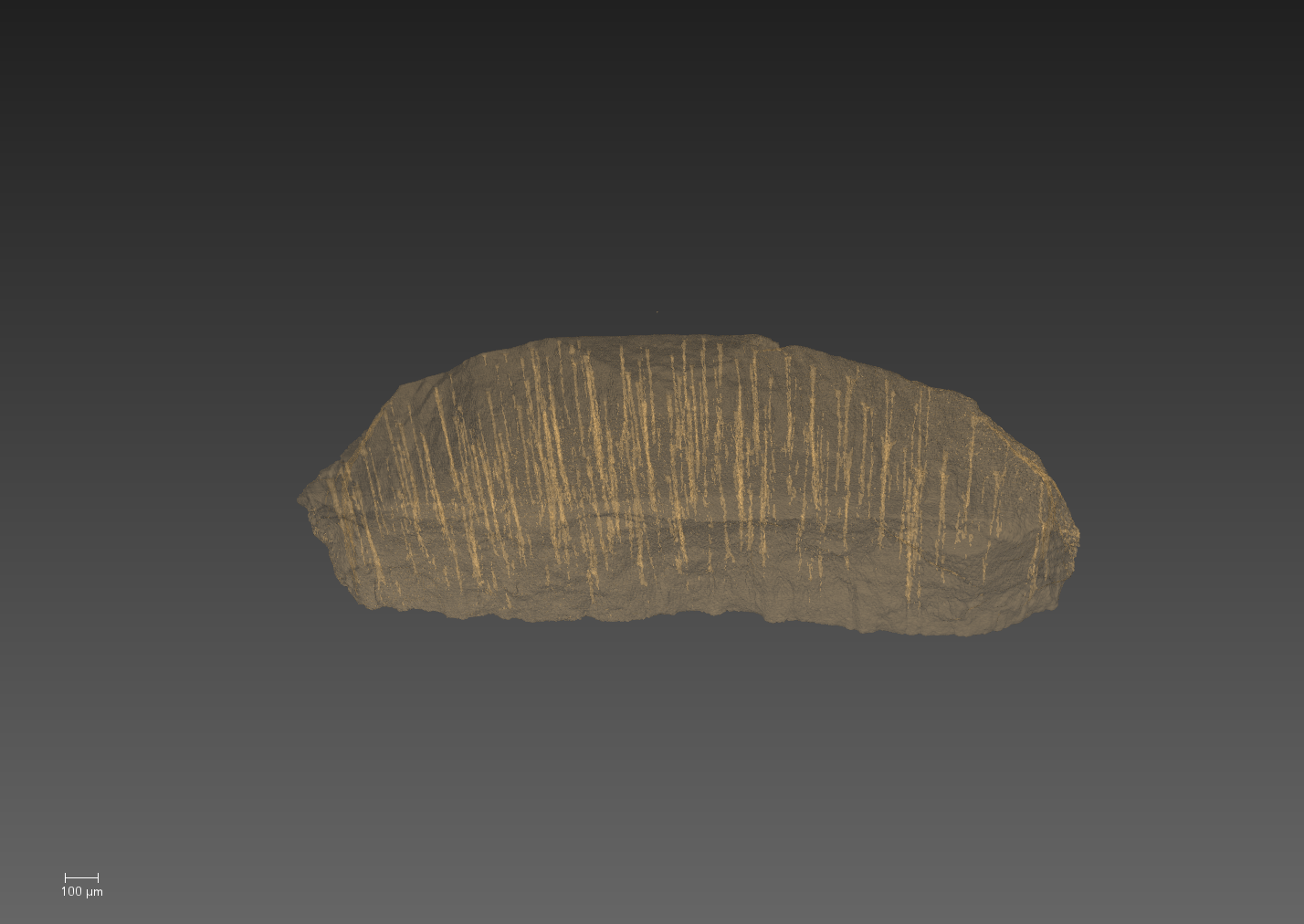

Supplement: Supplementary file 12 — Supplementary Data 12 [file 41467_2023_36405_MOESM12_ESM.zip › Micro_CT_raw_data/Southern_Aepyornis_thick/AD2118/Results/snapshot.tif]

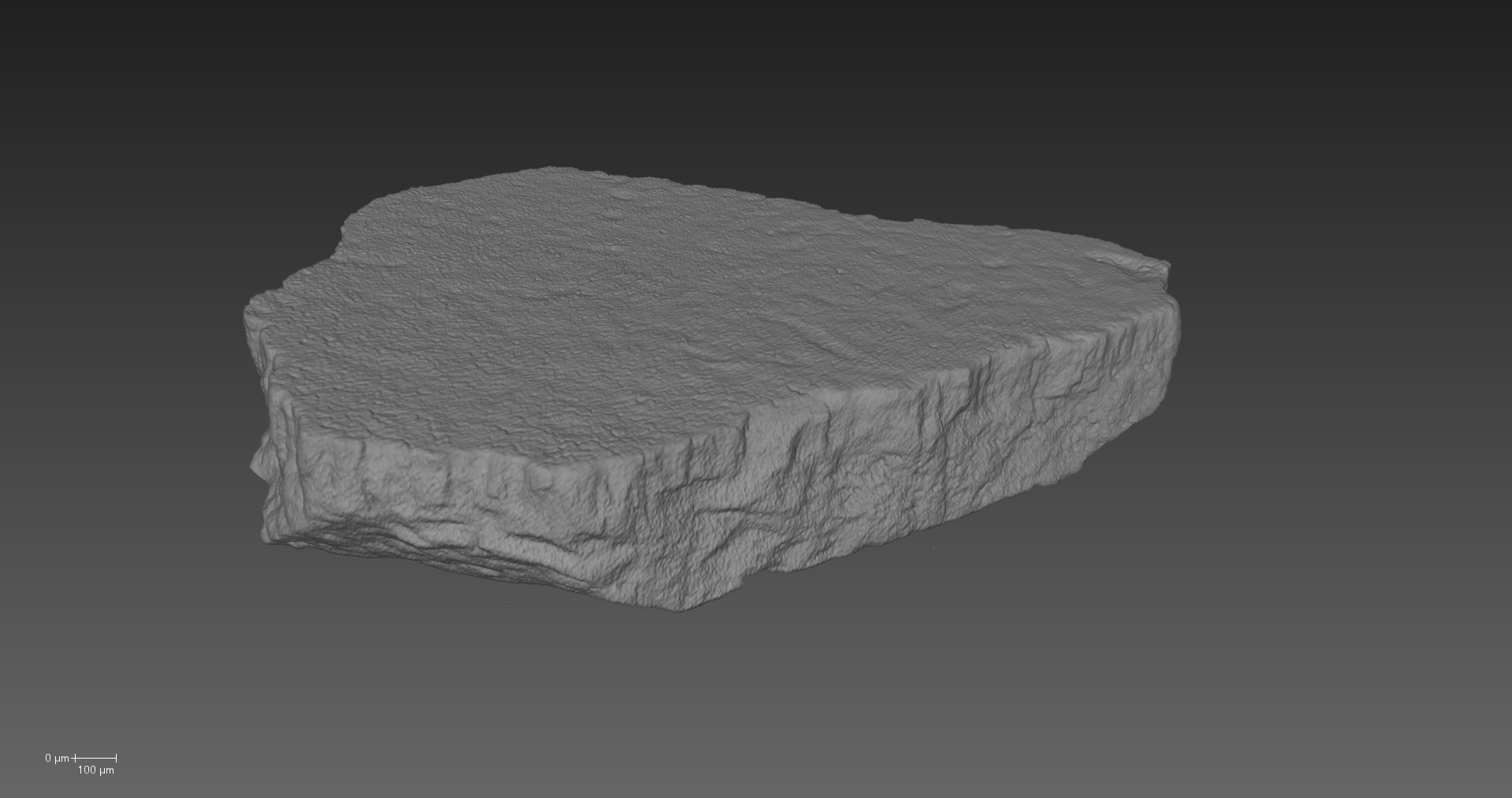

Supplement: Supplementary file 12 — Supplementary Data 12 [file 41467_2023_36405_MOESM12_ESM.zip › Micro_CT_raw_data/Southern_Aepyornis_thick/AD2118/Results/Inner surface.tif]

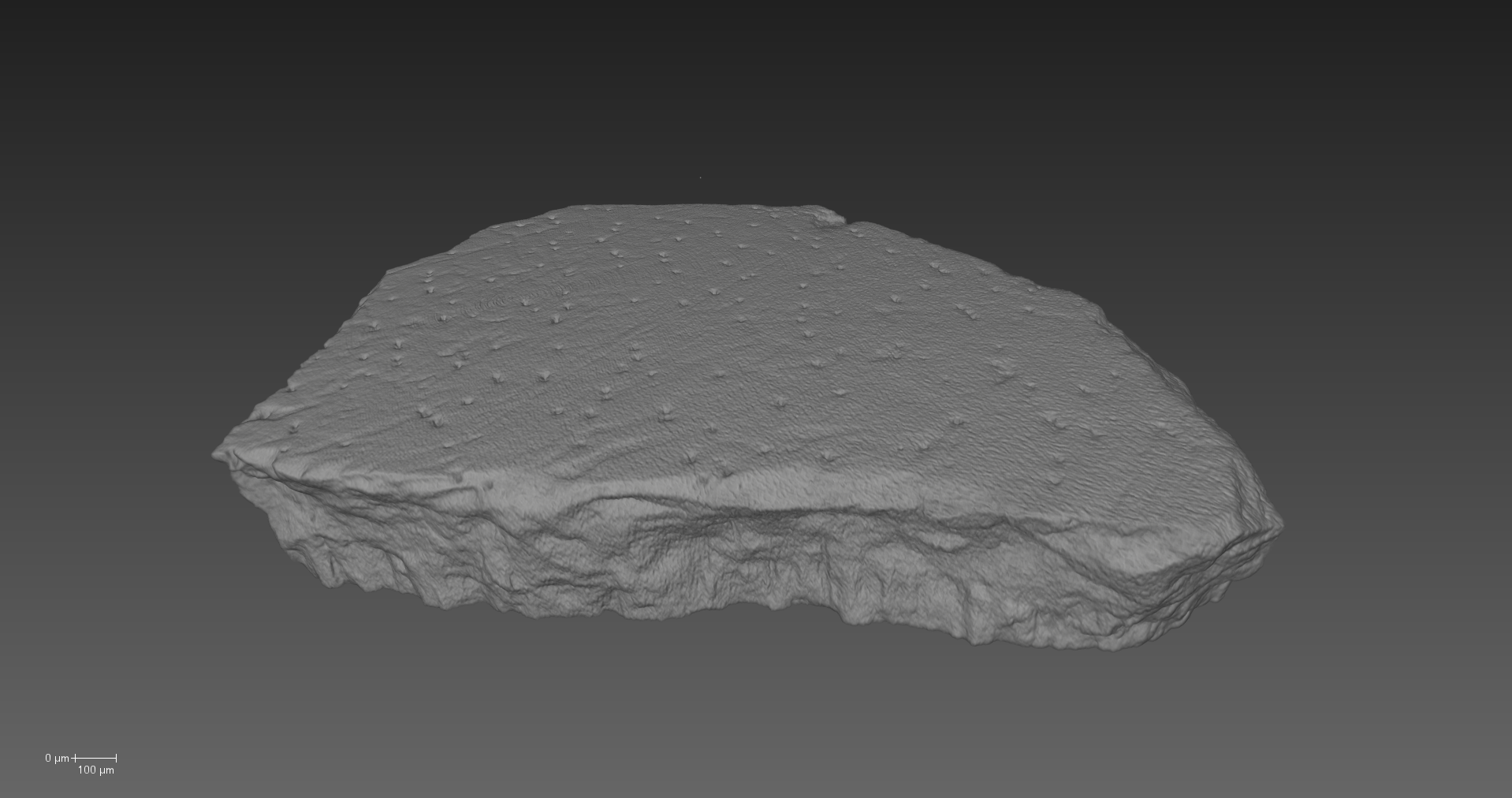

Supplement: Supplementary file 12 — Supplementary Data 12 [file 41467_2023_36405_MOESM12_ESM.zip › Micro_CT_raw_data/Southern_Aepyornis_thick/AD2118/Results/Outer surface.tif]

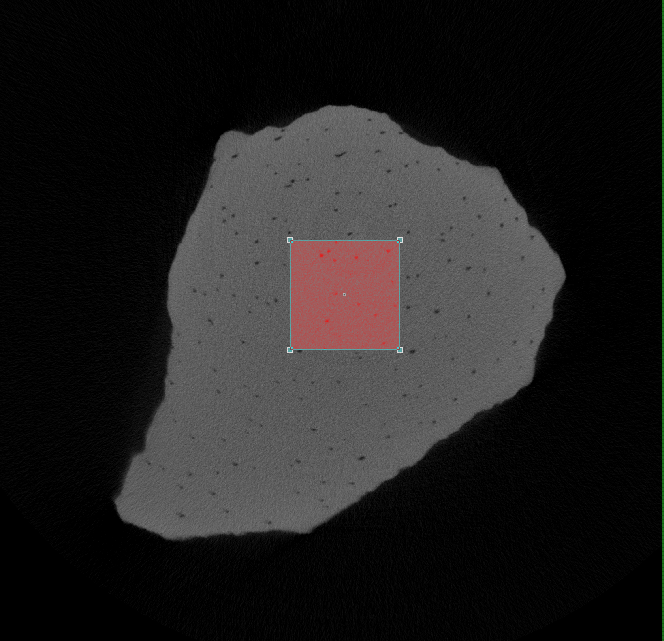

Supplement: Supplementary file 12 — Supplementary Data 12 [file 41467_2023_36405_MOESM12_ESM.zip › Micro_CT_raw_data/Southern_Aepyornis_thick/AD2118/Results/ROI Selection.tif]

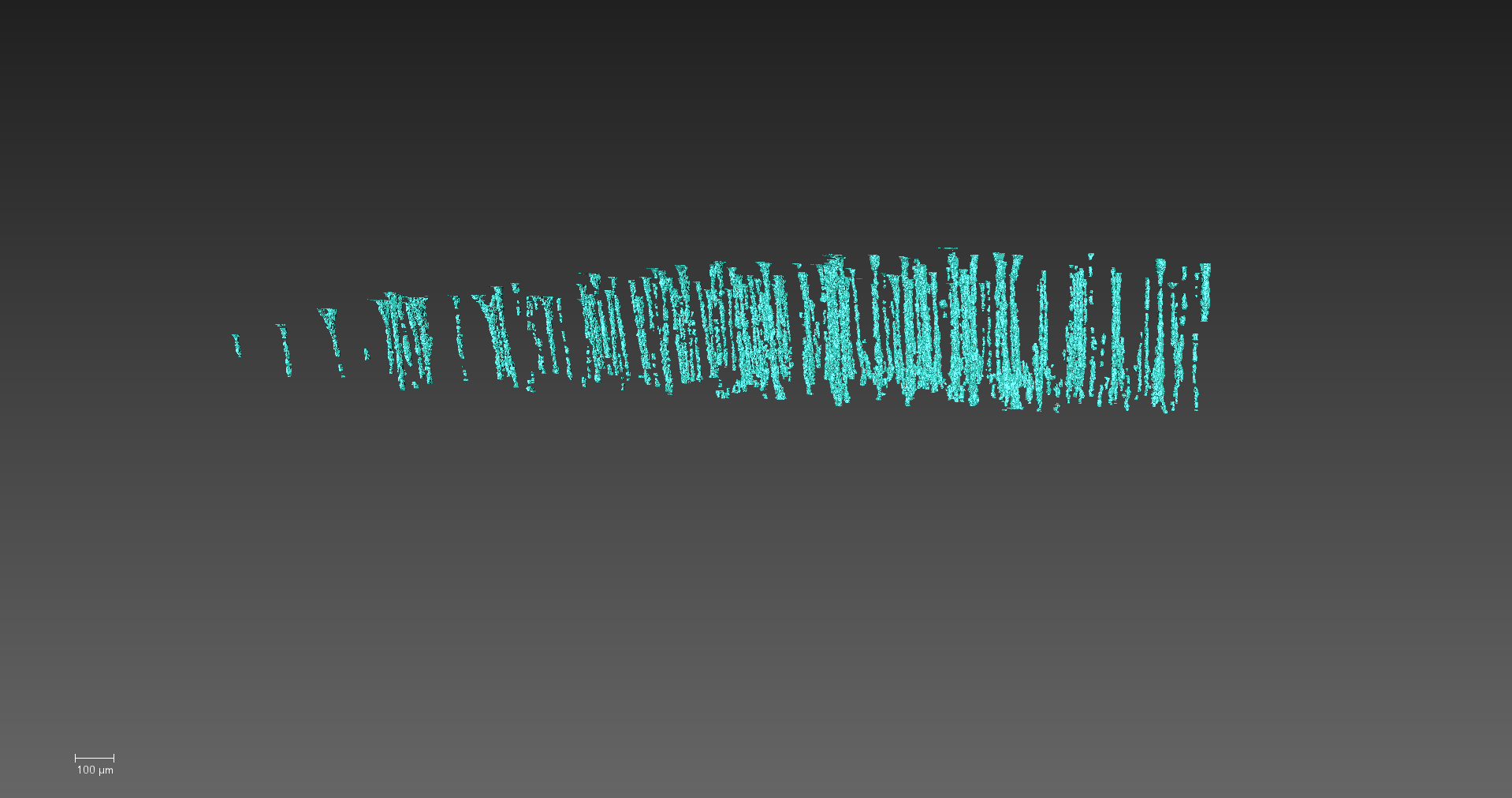

Supplement: Supplementary file 12 — Supplementary Data 12 [file 41467_2023_36405_MOESM12_ESM.zip › Micro_CT_raw_data/Southern_Aepyornis_thick/AD2118/Results/Pore structure1.tif]

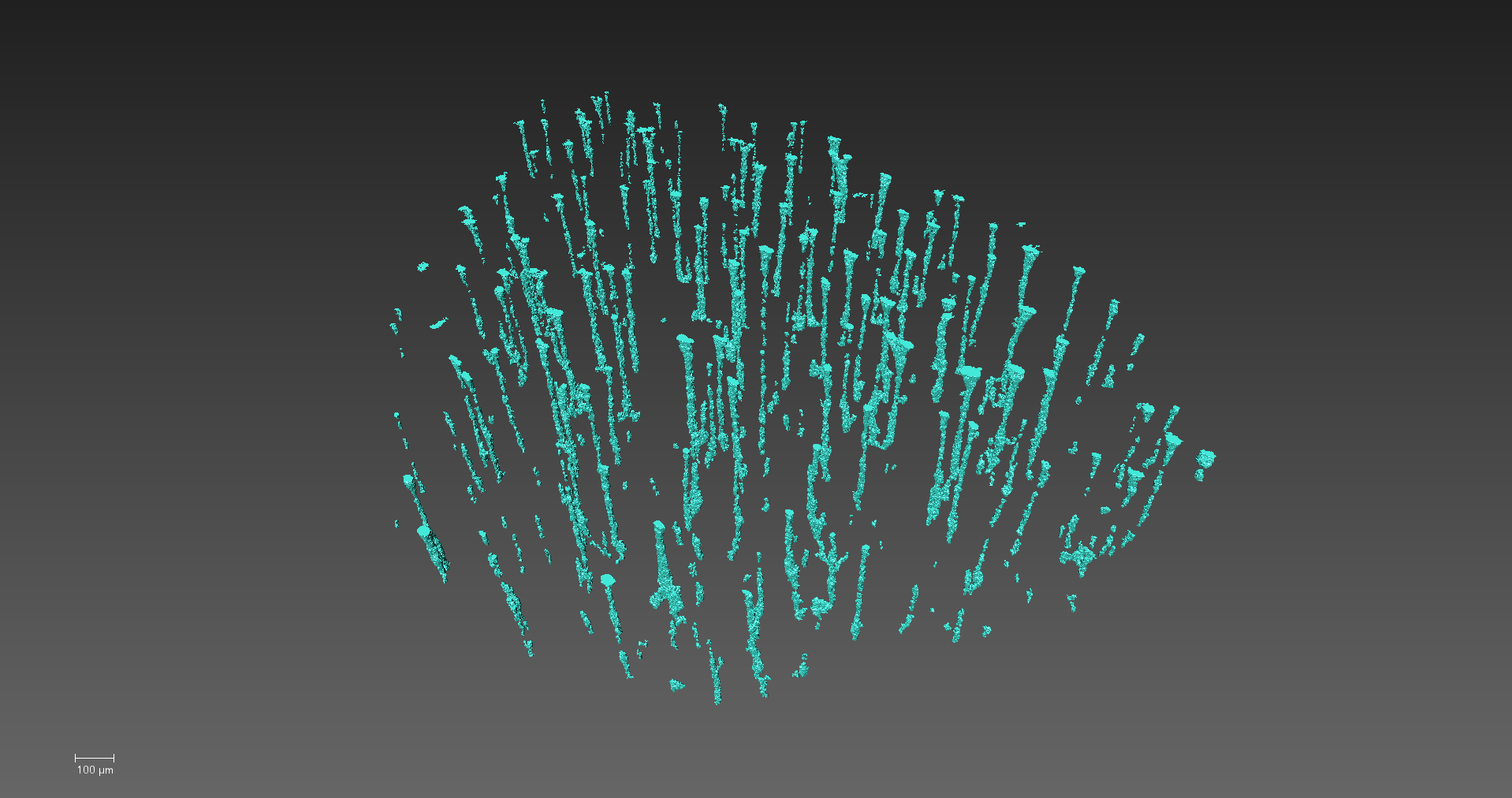

Supplement: Supplementary file 12 — Supplementary Data 12 [file 41467_2023_36405_MOESM12_ESM.zip › Micro_CT_raw_data/Southern_Aepyornis_thick/AD2118/Results/Pore structure2.tif]

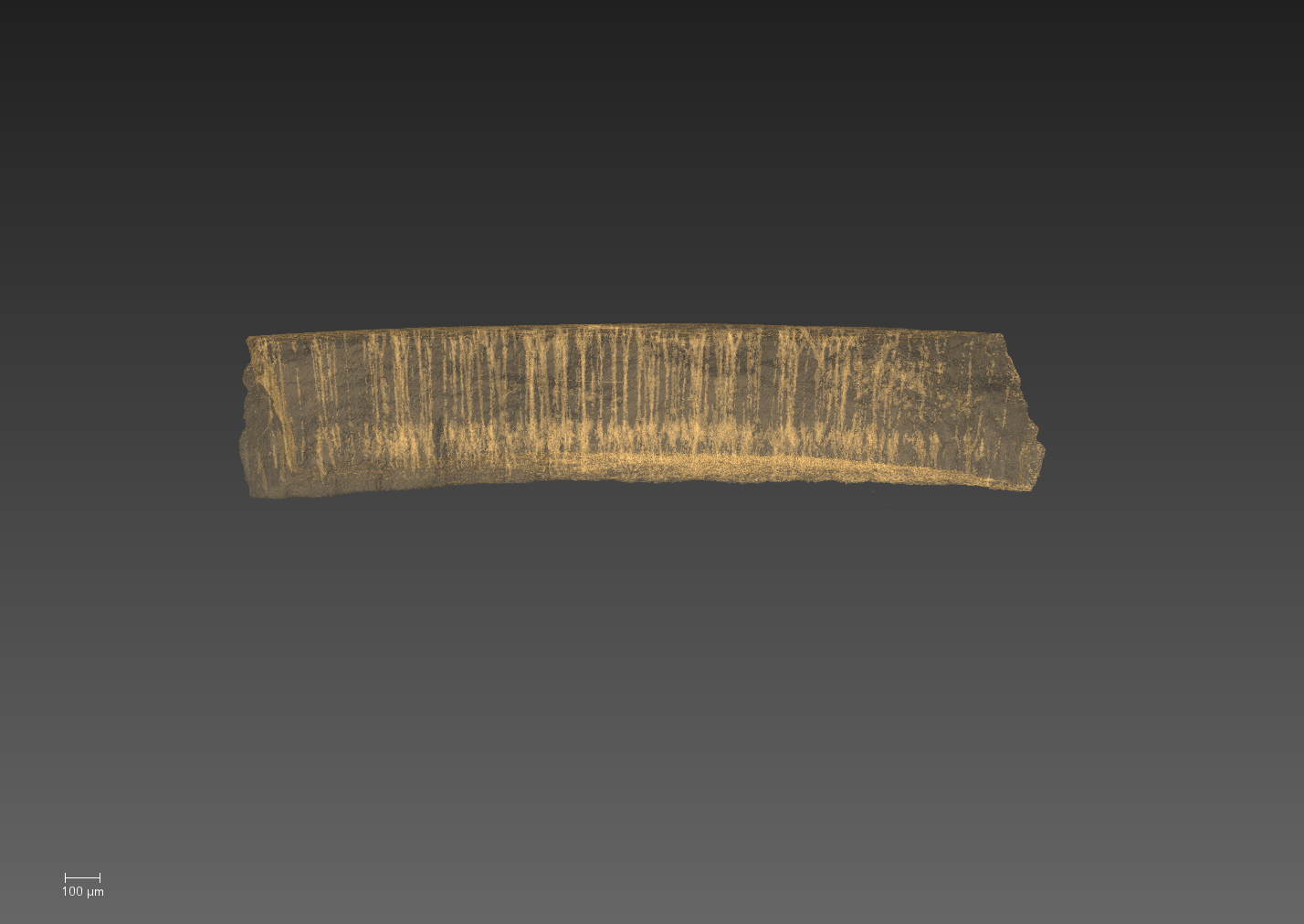

Supplement: Supplementary file 12 — Supplementary Data 12 [file 41467_2023_36405_MOESM12_ESM.zip › Micro_CT_raw_data/Southern_Aepyornis_thick/AD2409/Results/snapshot2.tif]

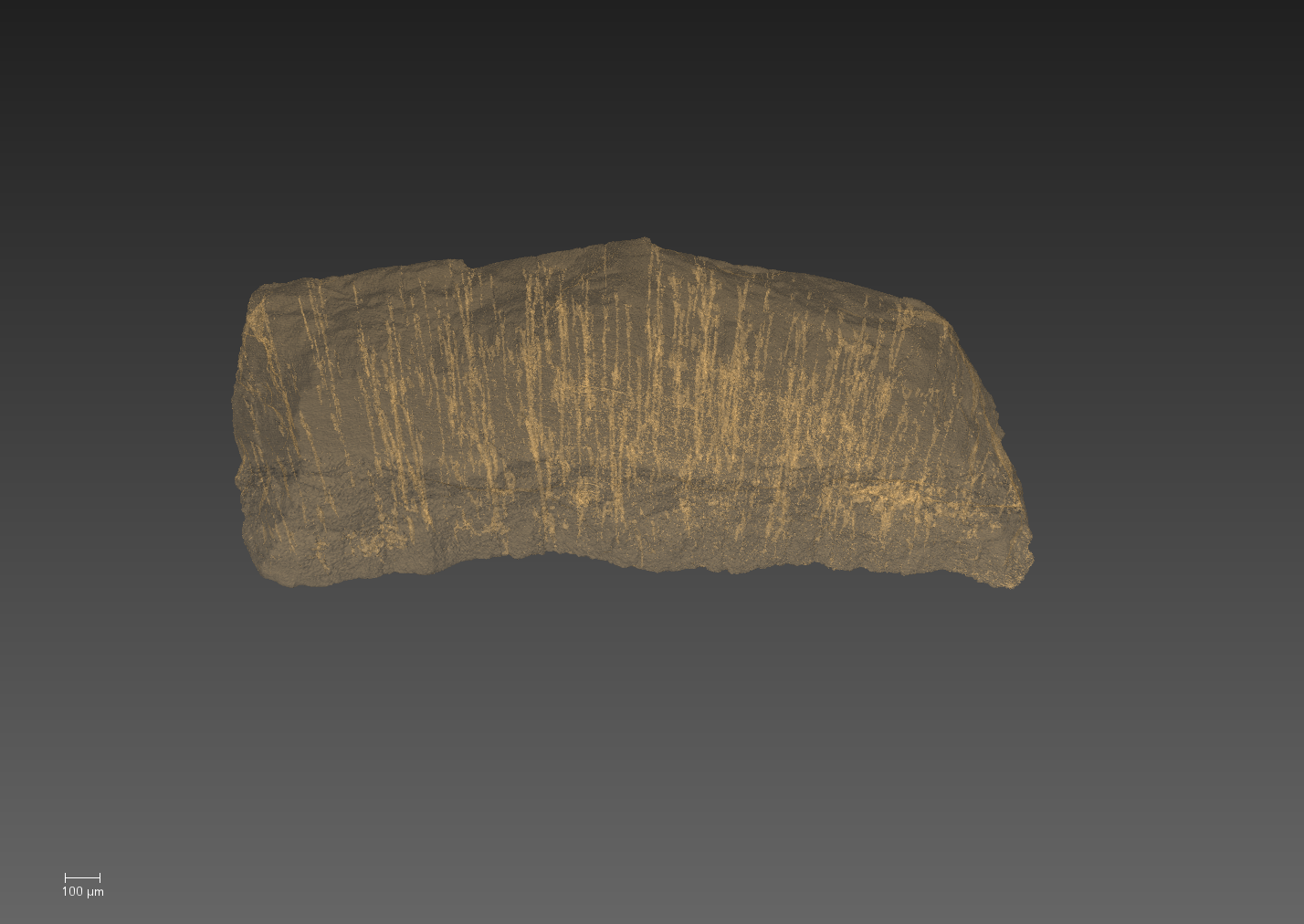

Supplement: Supplementary file 12 — Supplementary Data 12 [file 41467_2023_36405_MOESM12_ESM.zip › Micro_CT_raw_data/Southern_Aepyornis_thick/AD2409/Results/snapshot.tif]

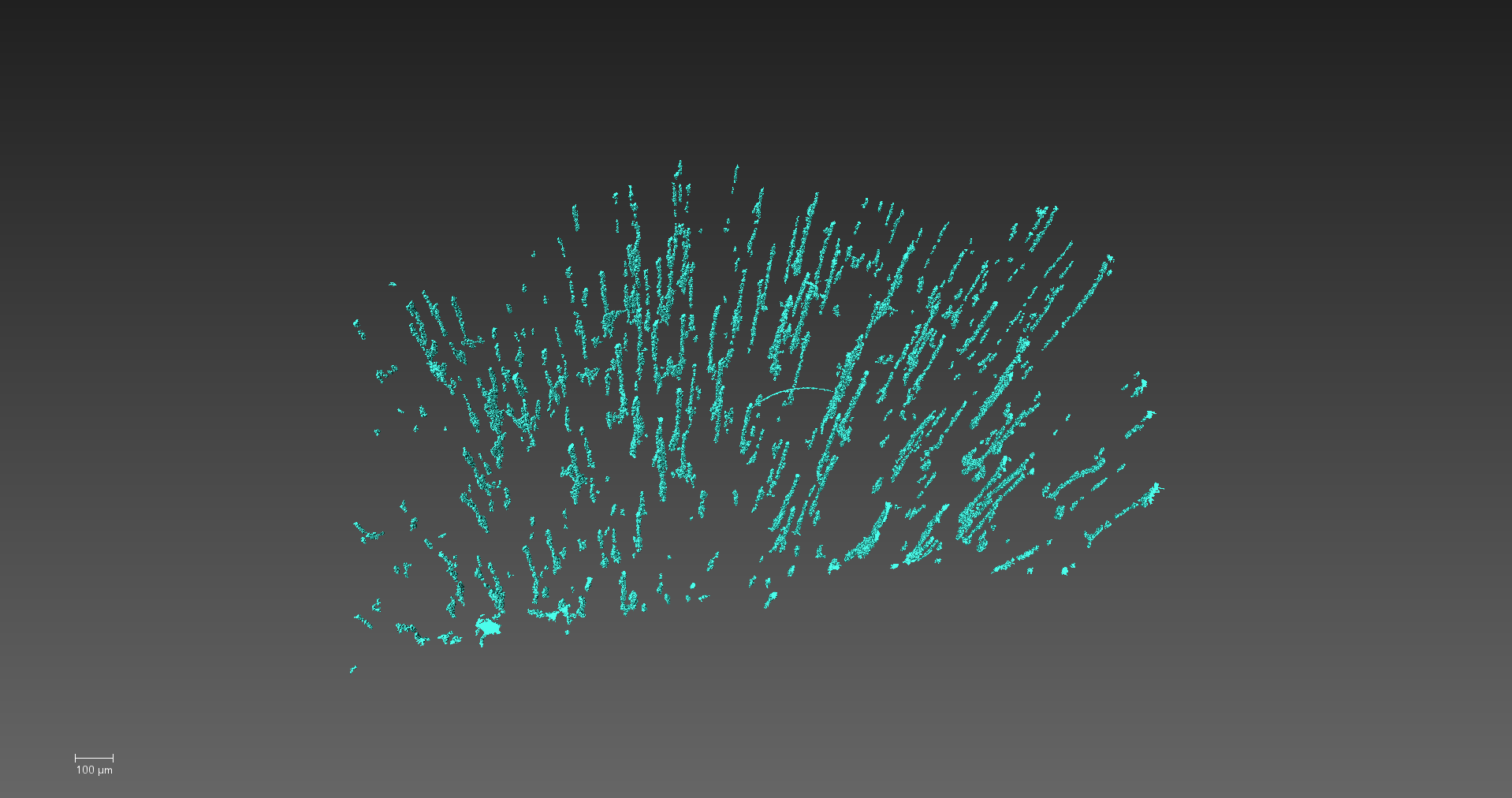

Supplement: Supplementary file 12 — Supplementary Data 12 [file 41467_2023_36405_MOESM12_ESM.zip › Micro_CT_raw_data/Southern_Aepyornis_thick/AD2409/Results/Pore stucture2.tif]

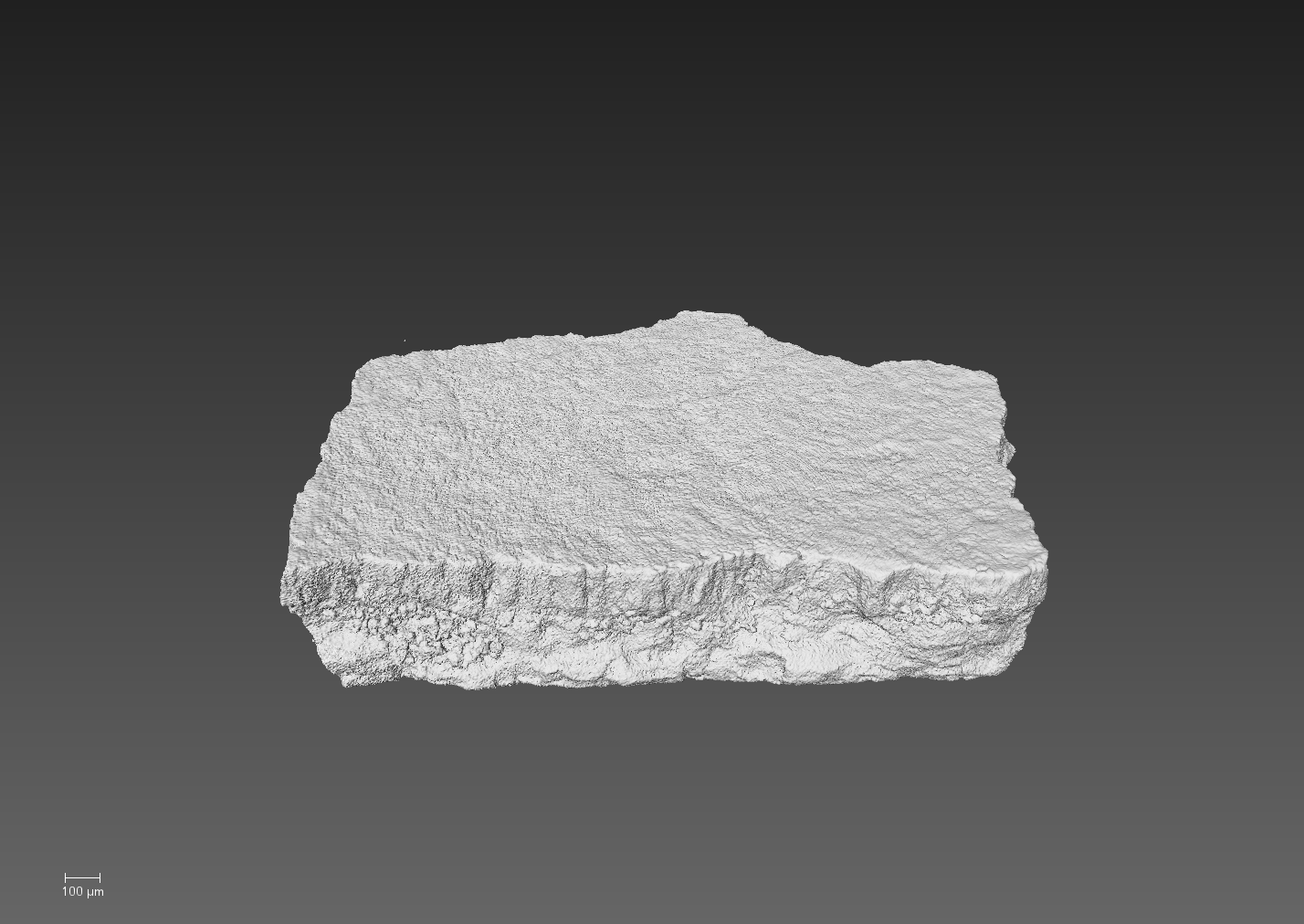

Supplement: Supplementary file 12 — Supplementary Data 12 [file 41467_2023_36405_MOESM12_ESM.zip › Micro_CT_raw_data/Southern_Aepyornis_thick/AD2409/Results/Inner surface.tif]

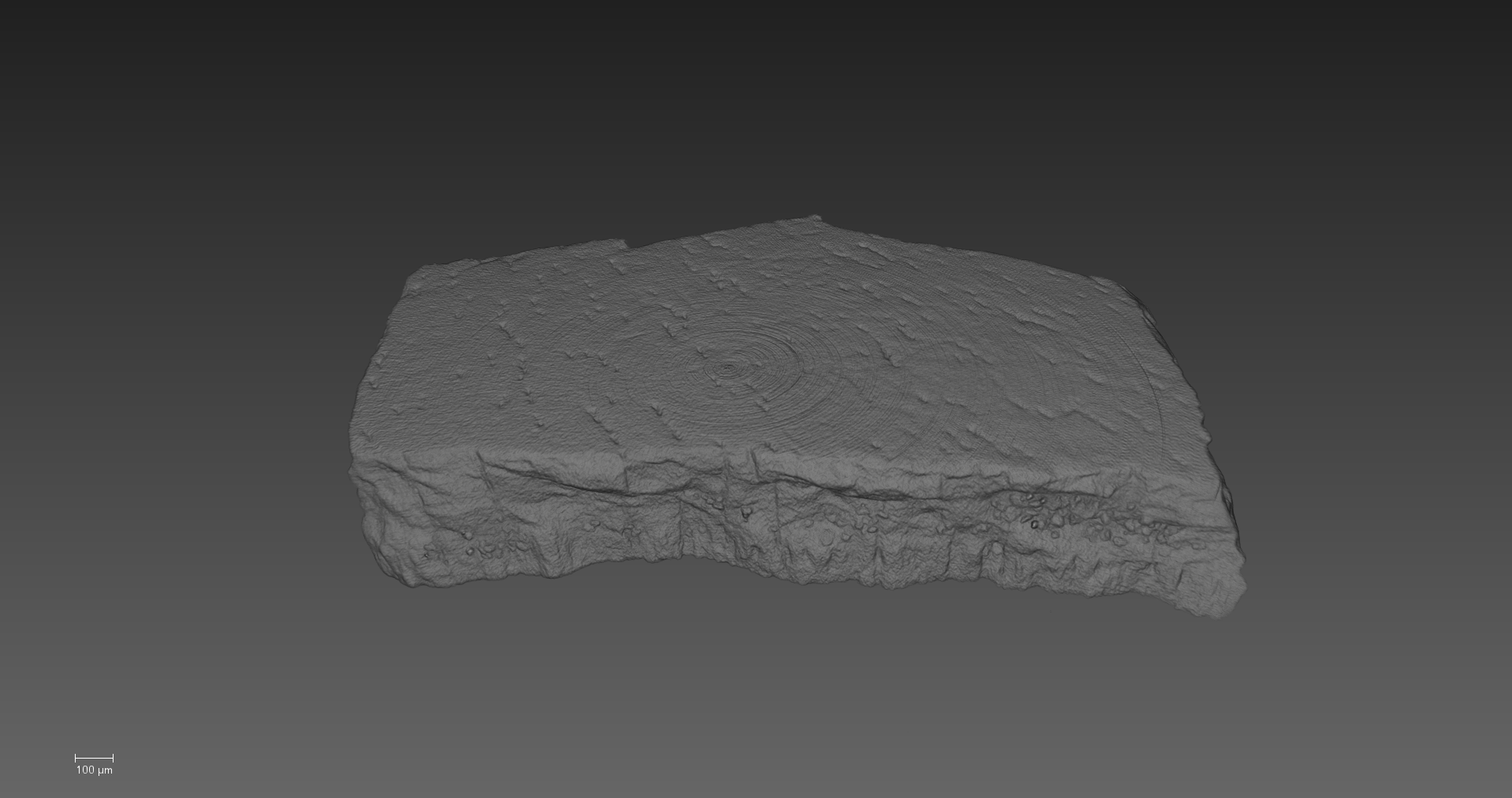

Supplement: Supplementary file 12 — Supplementary Data 12 [file 41467_2023_36405_MOESM12_ESM.zip › Micro_CT_raw_data/Southern_Aepyornis_thick/AD2409/Results/Outer surface.tif]

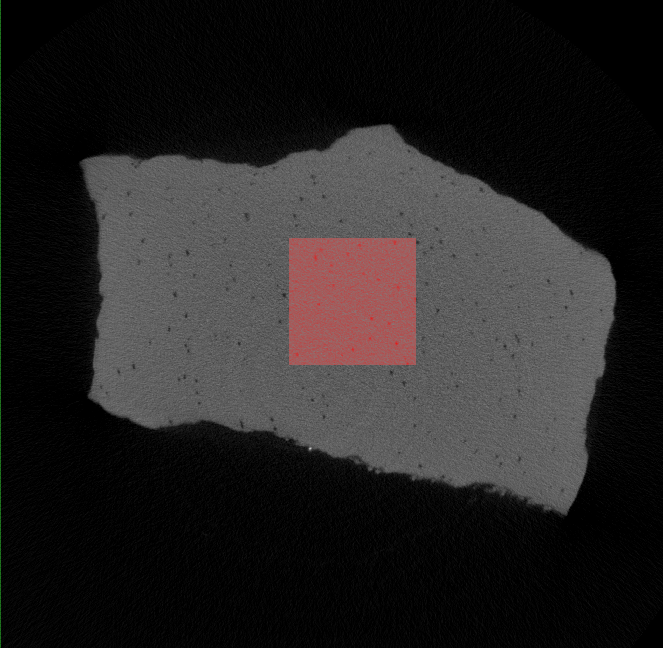

Supplement: Supplementary file 12 — Supplementary Data 12 [file 41467_2023_36405_MOESM12_ESM.zip › Micro_CT_raw_data/Southern_Aepyornis_thick/AD2409/Results/ROI Selection.tif]

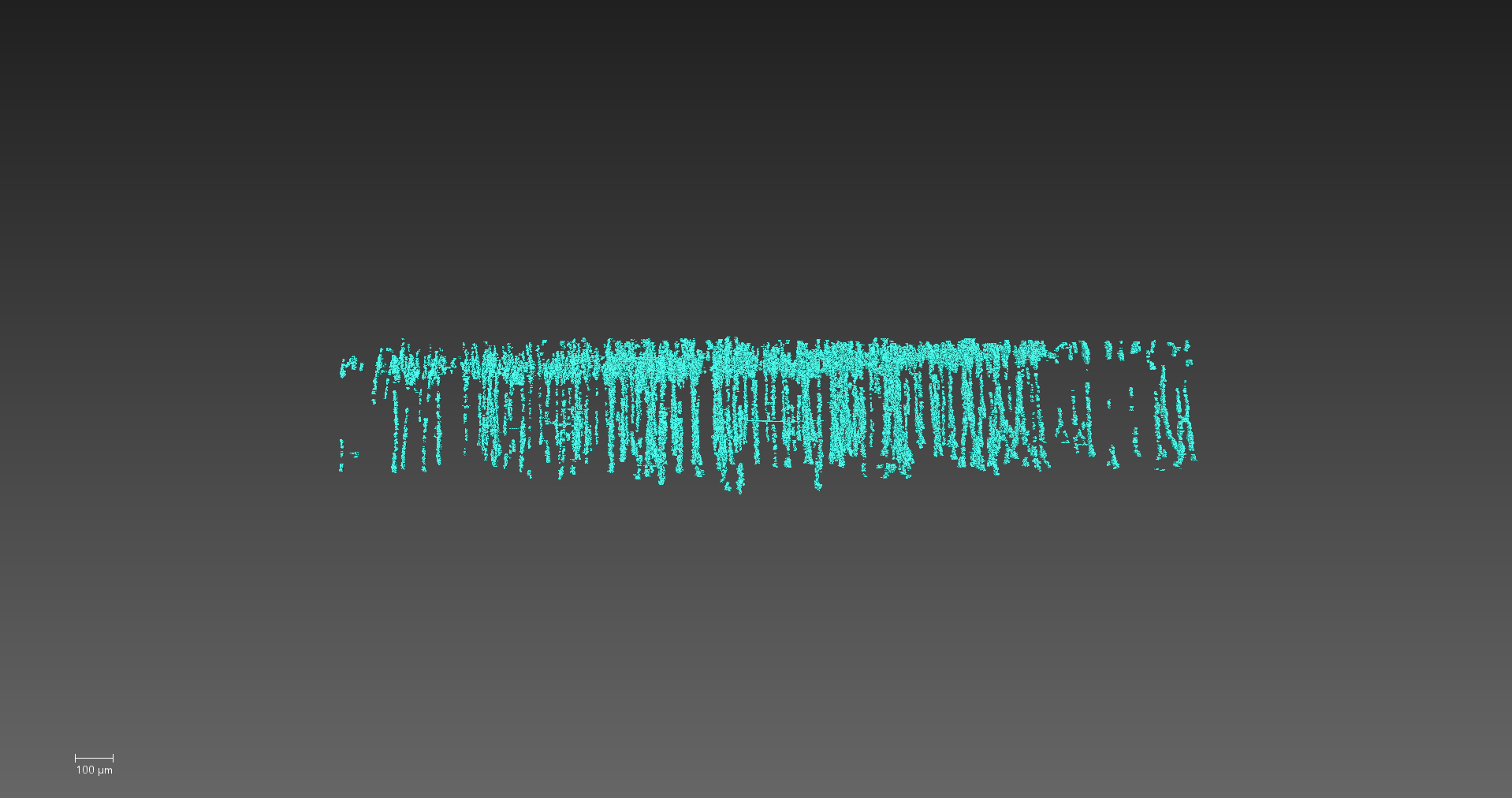

Supplement: Supplementary file 12 — Supplementary Data 12 [file 41467_2023_36405_MOESM12_ESM.zip › Micro_CT_raw_data/Southern_Aepyornis_thick/AD2409/Results/Pore structure1.tif]

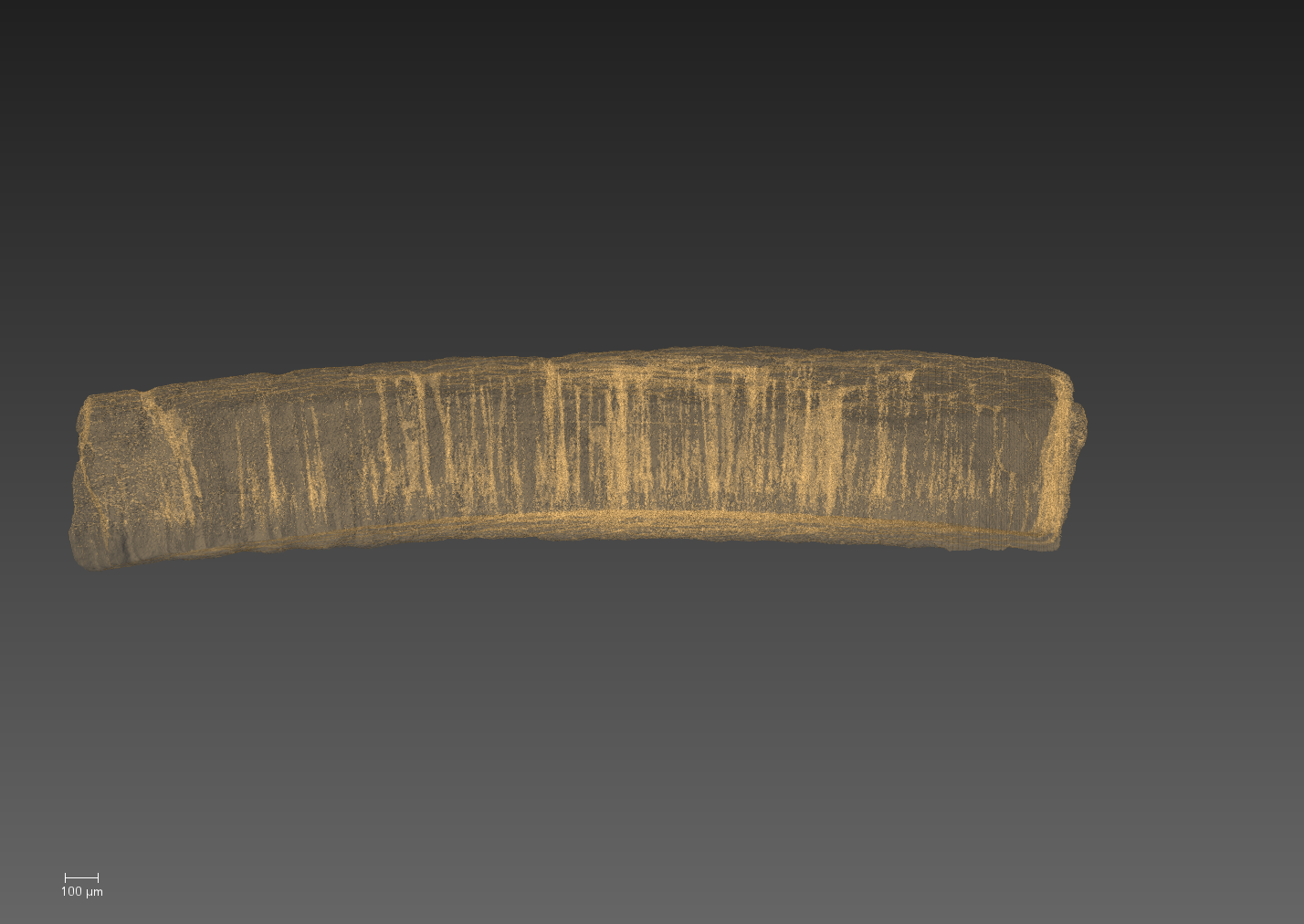

Supplement: Supplementary file 12 — Supplementary Data 12 [file 41467_2023_36405_MOESM12_ESM.zip › Micro_CT_raw_data/Southern_Aepyornis_thick/AD1739/Results/snapshot3.tif]

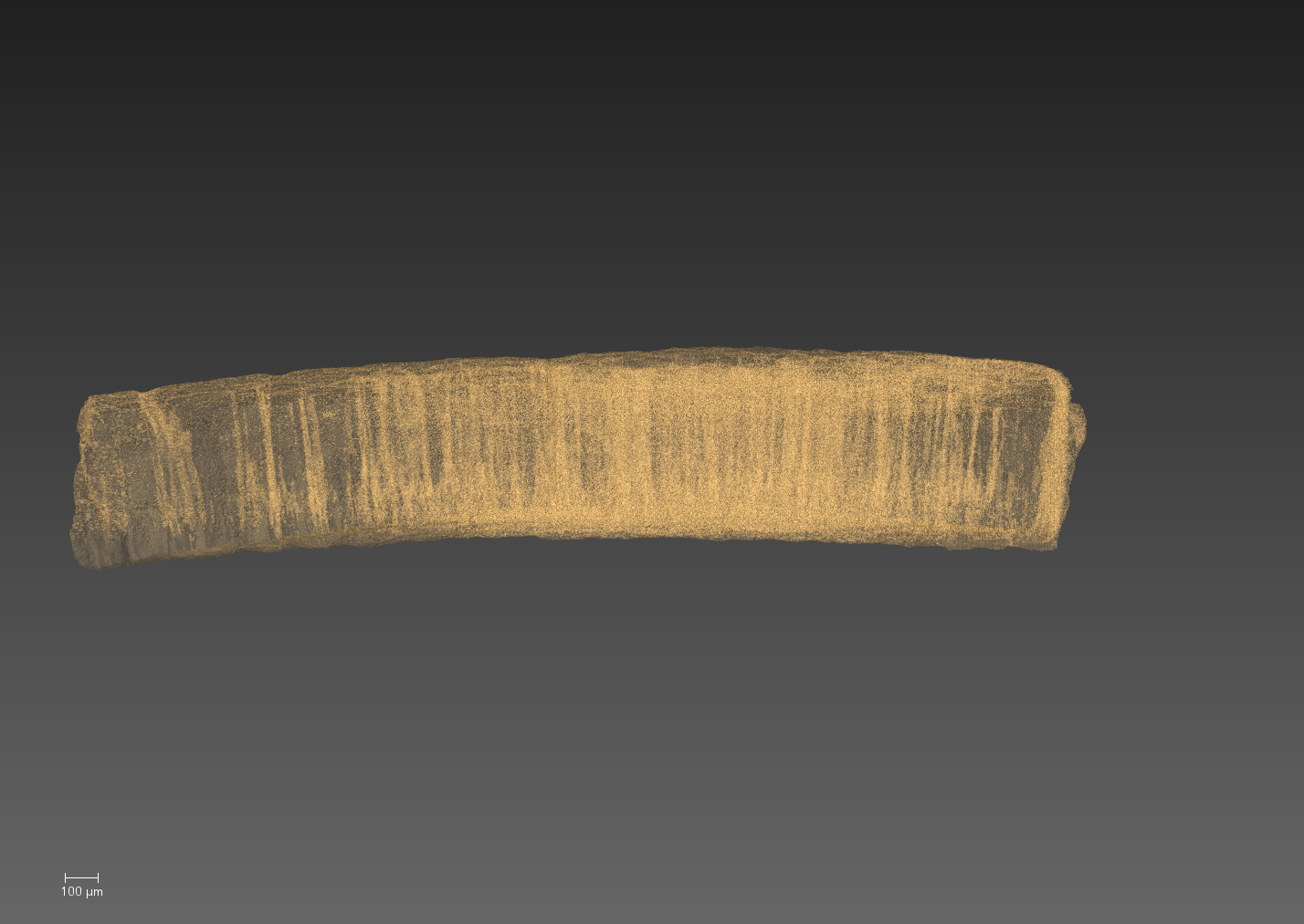

Supplement: Supplementary file 12 — Supplementary Data 12 [file 41467_2023_36405_MOESM12_ESM.zip › Micro_CT_raw_data/Southern_Aepyornis_thick/AD1739/Results/snapshot2.tif]

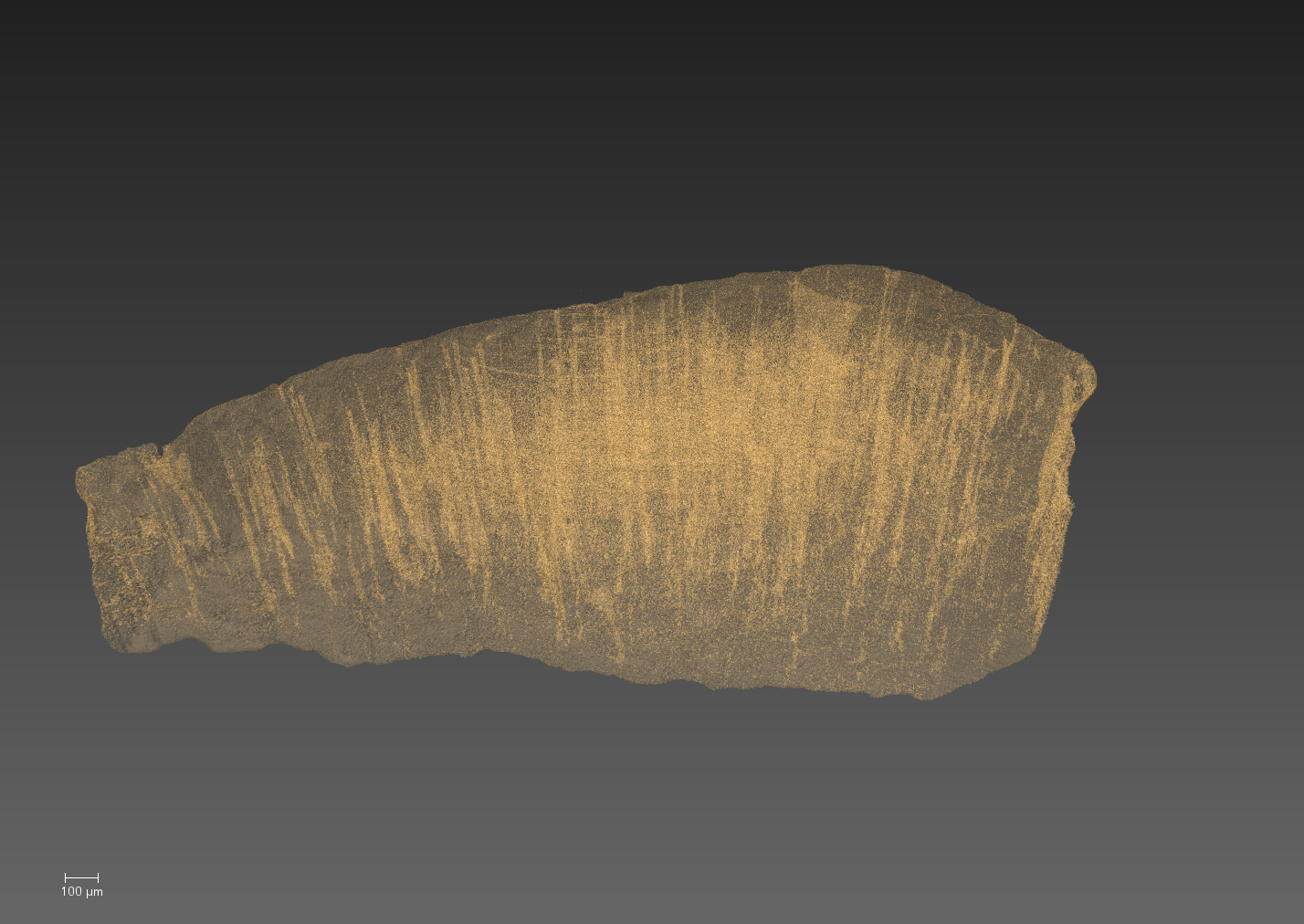

Supplement: Supplementary file 12 — Supplementary Data 12 [file 41467_2023_36405_MOESM12_ESM.zip › Micro_CT_raw_data/Southern_Aepyornis_thick/AD1739/Results/snapshot.tif]

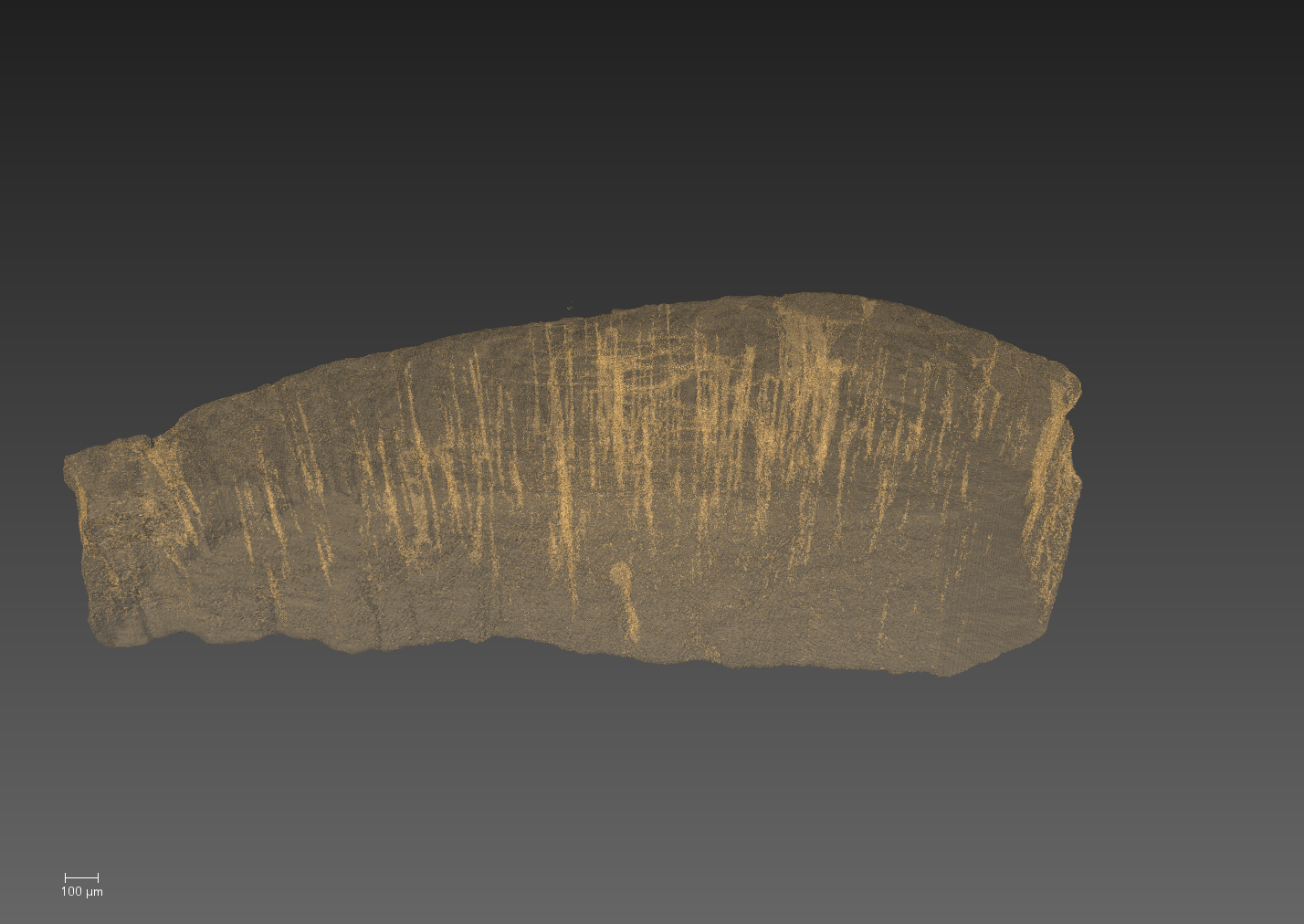

Supplement: Supplementary file 12 — Supplementary Data 12 [file 41467_2023_36405_MOESM12_ESM.zip › Micro_CT_raw_data/Southern_Aepyornis_thick/AD1739/Results/snapshot4.tif]

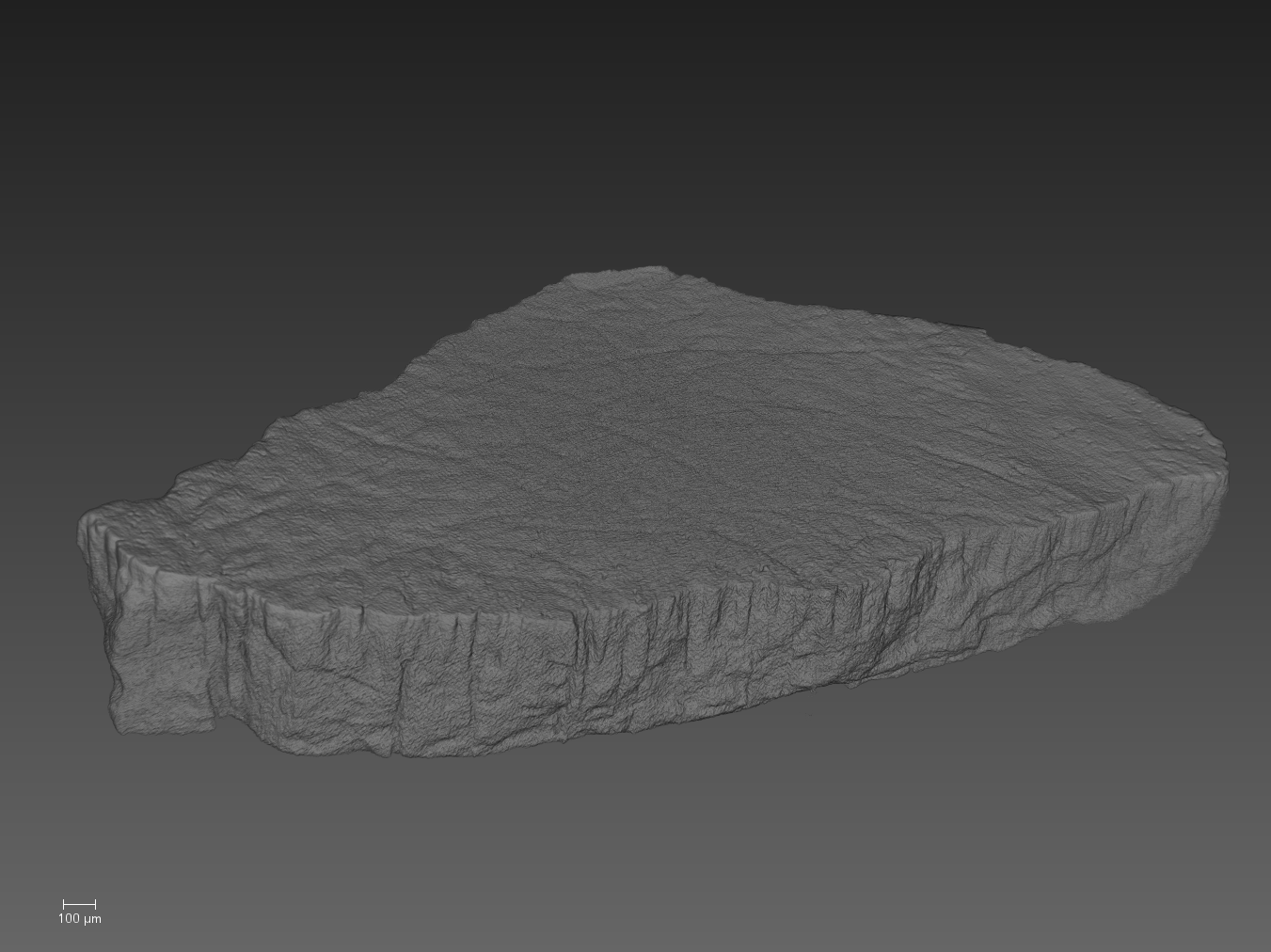

Supplement: Supplementary file 12 — Supplementary Data 12 [file 41467_2023_36405_MOESM12_ESM.zip › Micro_CT_raw_data/Southern_Aepyornis_thick/AD1739/Results/Inner surface.tif]

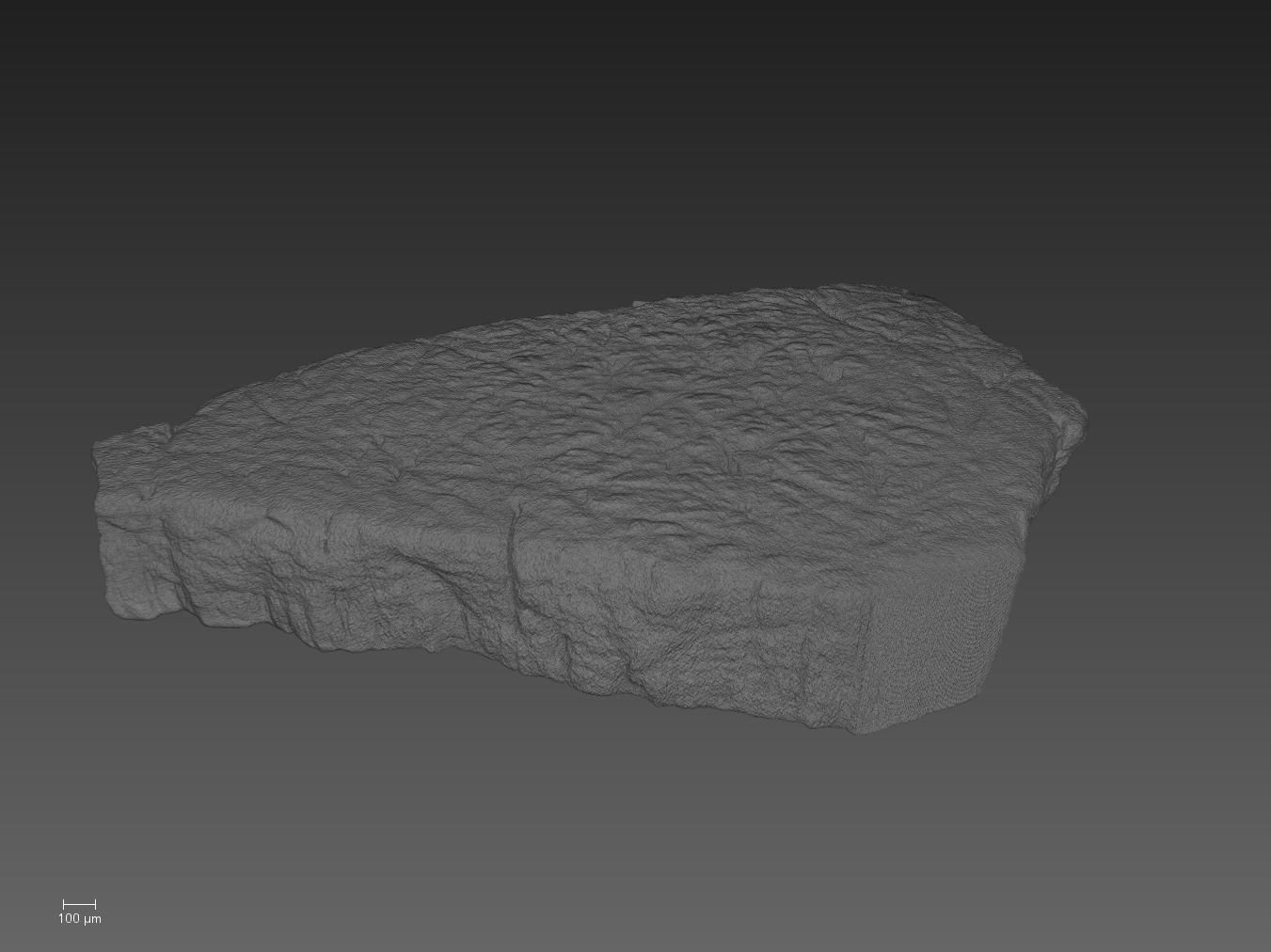

Supplement: Supplementary file 12 — Supplementary Data 12 [file 41467_2023_36405_MOESM12_ESM.zip › Micro_CT_raw_data/Southern_Aepyornis_thick/AD1739/Results/Outer surface.tif]

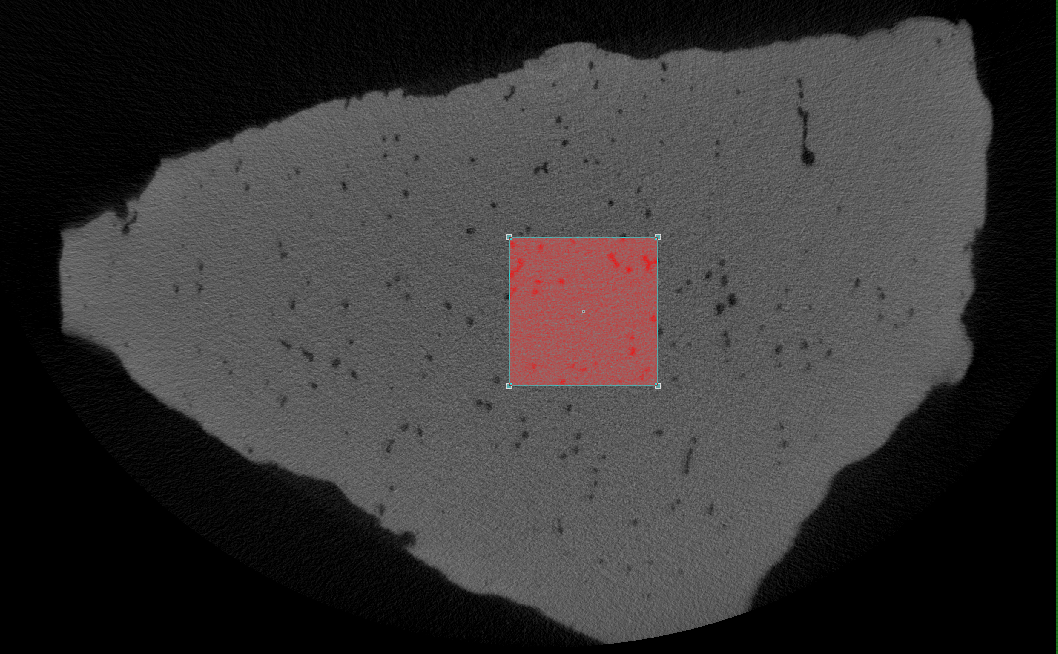

Supplement: Supplementary file 12 — Supplementary Data 12 [file 41467_2023_36405_MOESM12_ESM.zip › Micro_CT_raw_data/Southern_Aepyornis_thick/AD1739/Results/ROI Selection.tif]

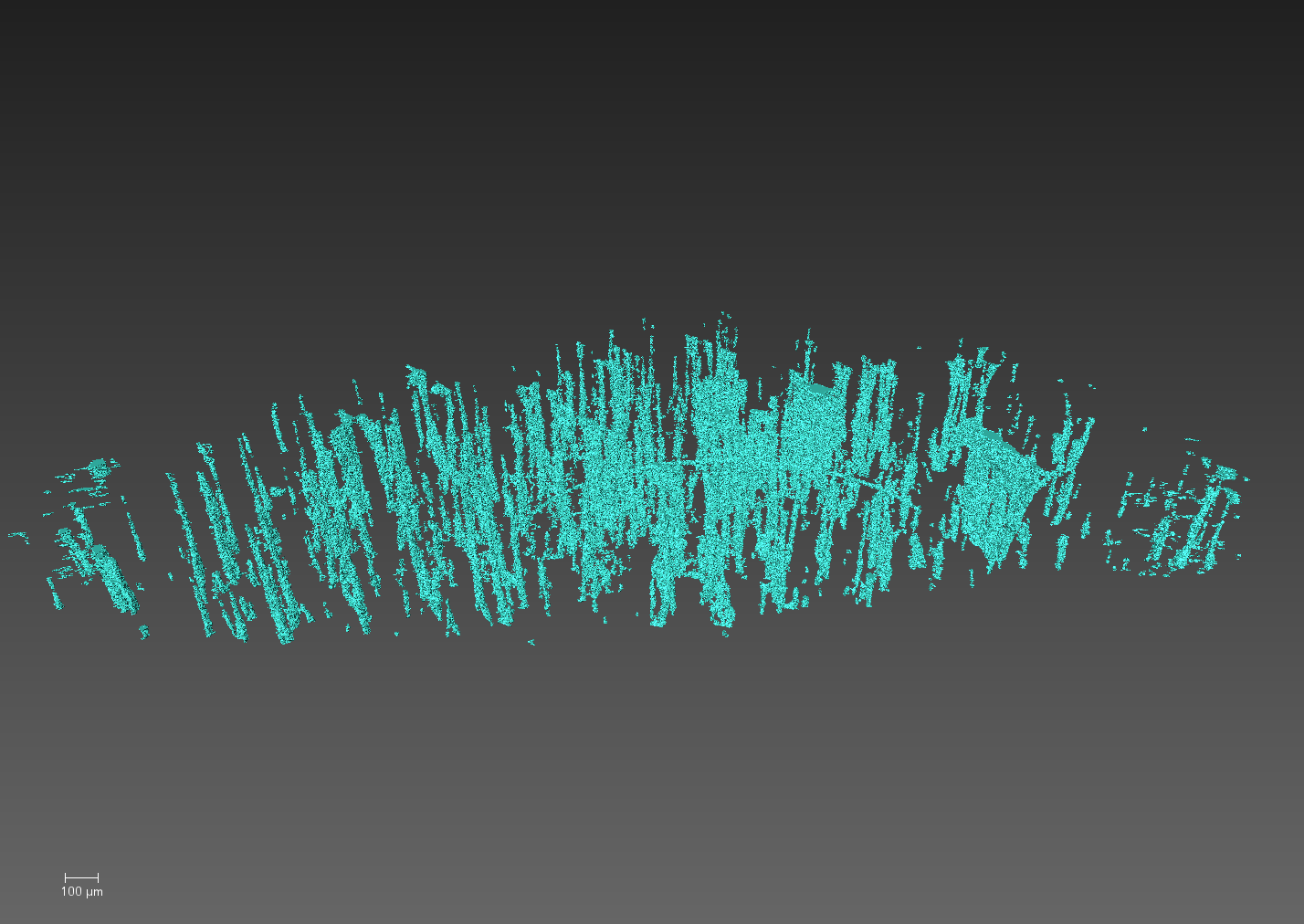

Supplement: Supplementary file 12 — Supplementary Data 12 [file 41467_2023_36405_MOESM12_ESM.zip › Micro_CT_raw_data/Southern_Aepyornis_thick/AD1739/Results/Pore structure1.tif]

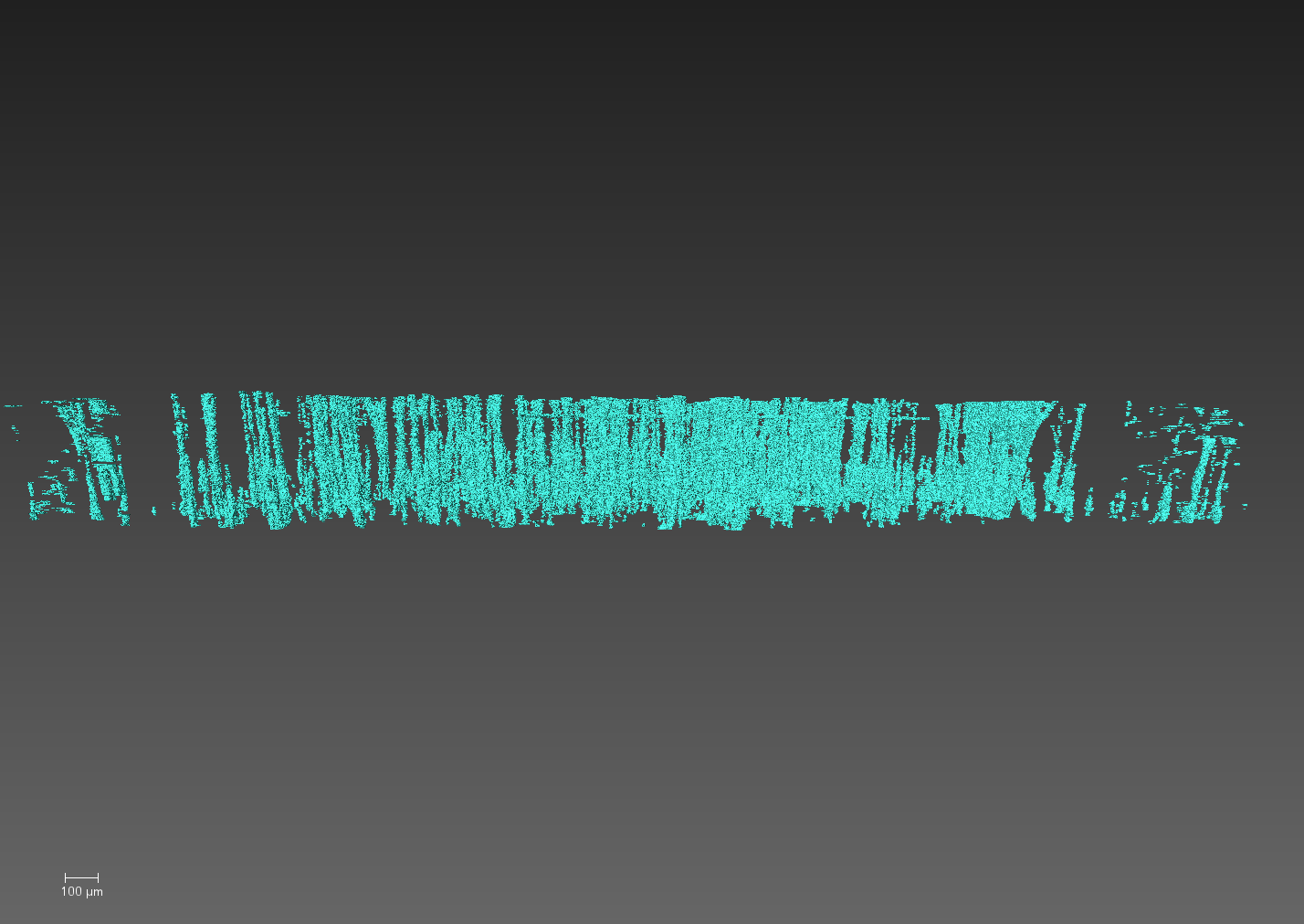

Supplement: Supplementary file 12 — Supplementary Data 12 [file 41467_2023_36405_MOESM12_ESM.zip › Micro_CT_raw_data/Southern_Aepyornis_thick/AD1739/Results/Pore structure2.tif]

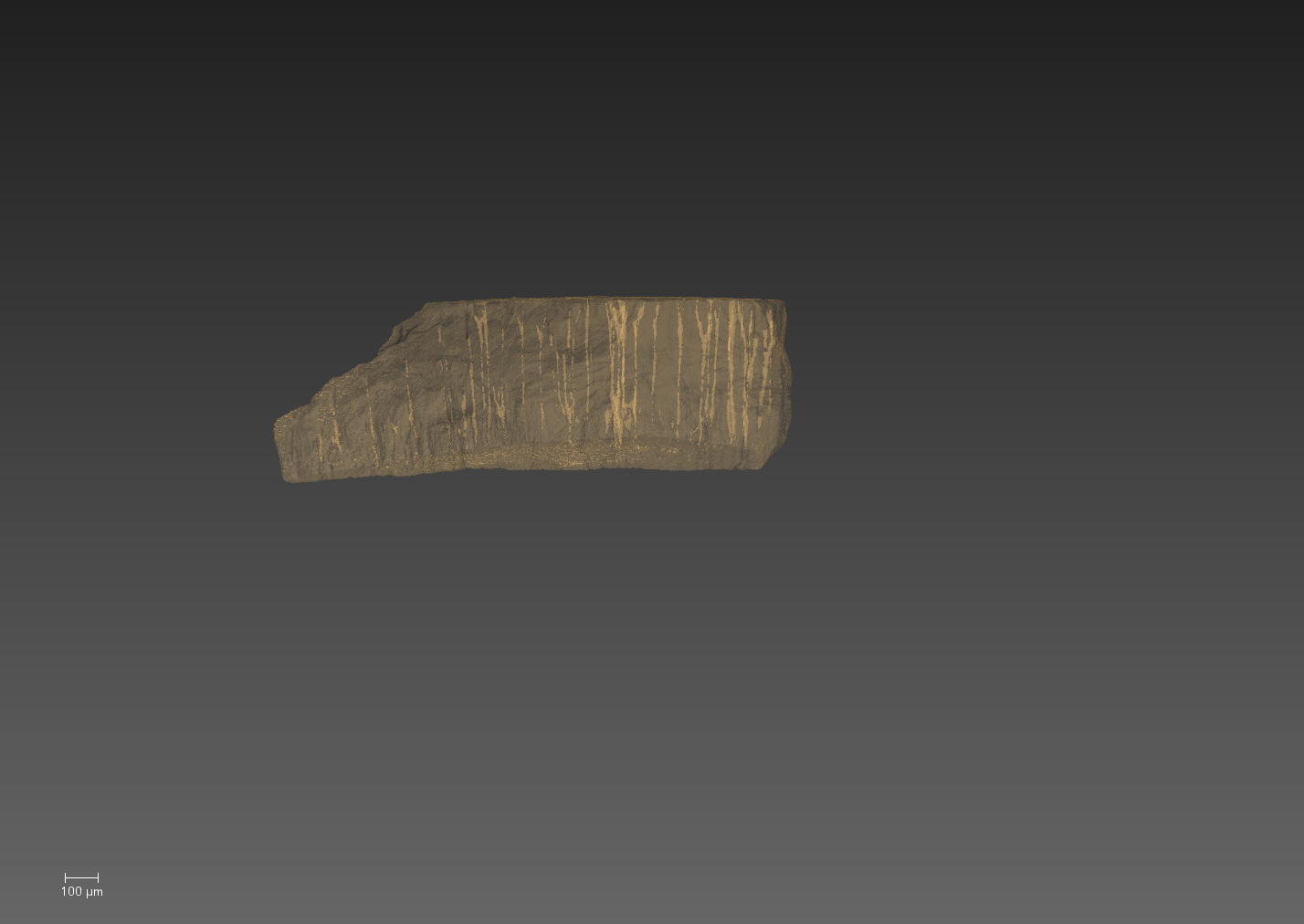

Supplement: Supplementary file 12 — Supplementary Data 12 [file 41467_2023_36405_MOESM12_ESM.zip › Micro_CT_raw_data/Southern_Aepyornis_thick/AD1666/Results/snapshot2.tif]

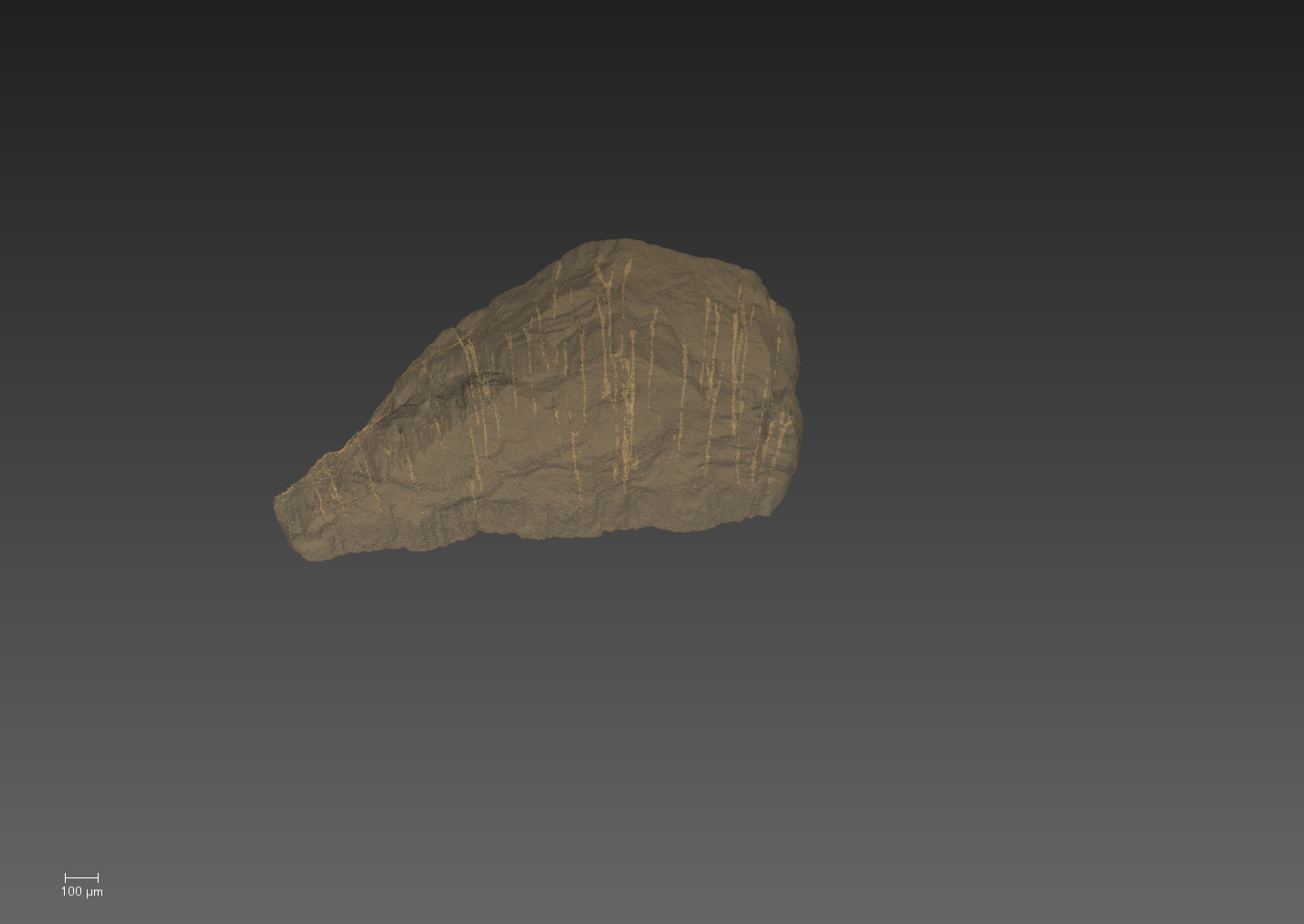

Supplement: Supplementary file 12 — Supplementary Data 12 [file 41467_2023_36405_MOESM12_ESM.zip › Micro_CT_raw_data/Southern_Aepyornis_thick/AD1666/Results/snapshot.tif]

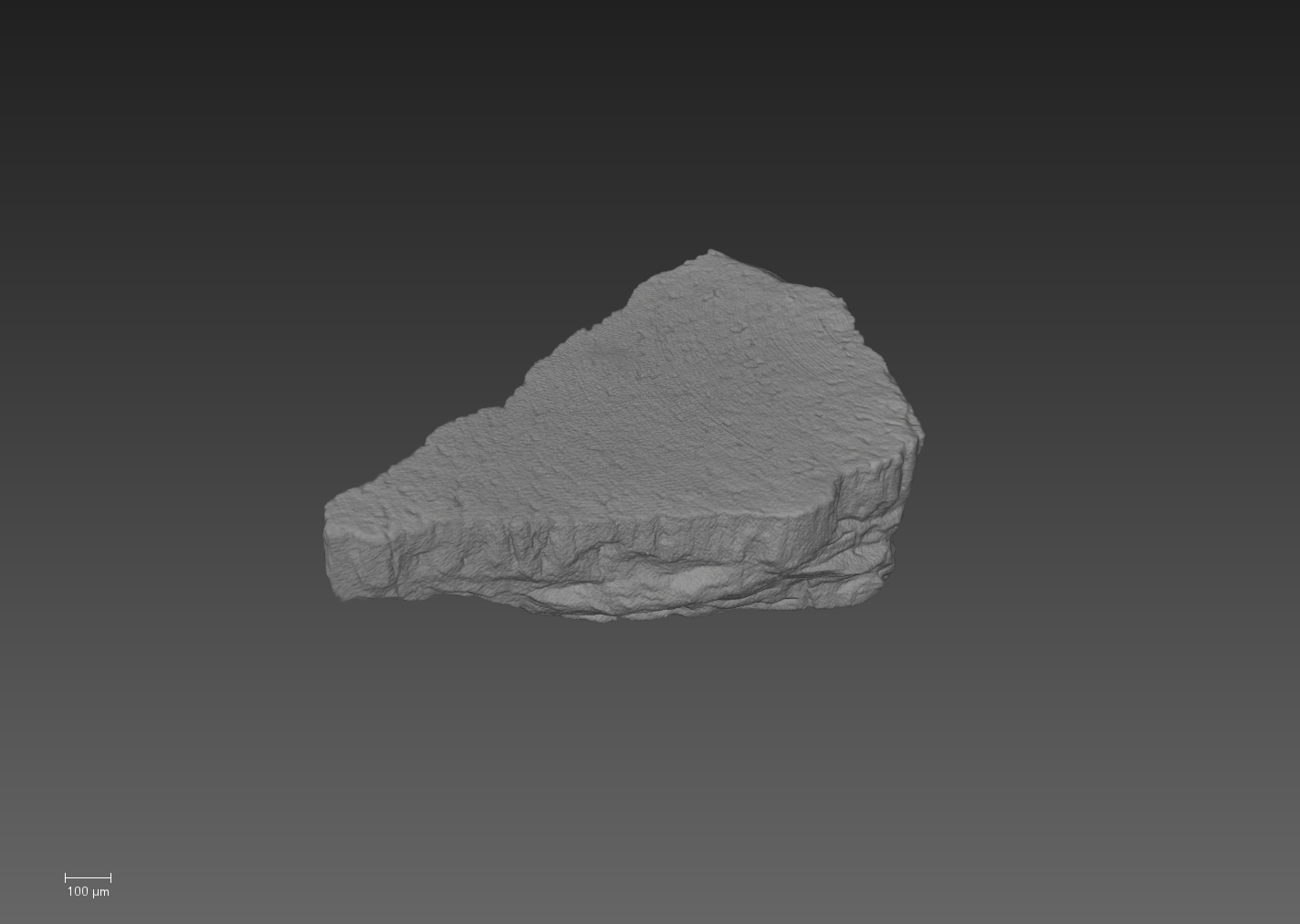

Supplement: Supplementary file 12 — Supplementary Data 12 [file 41467_2023_36405_MOESM12_ESM.zip › Micro_CT_raw_data/Southern_Aepyornis_thick/AD1666/Results/Inner surface.tif]

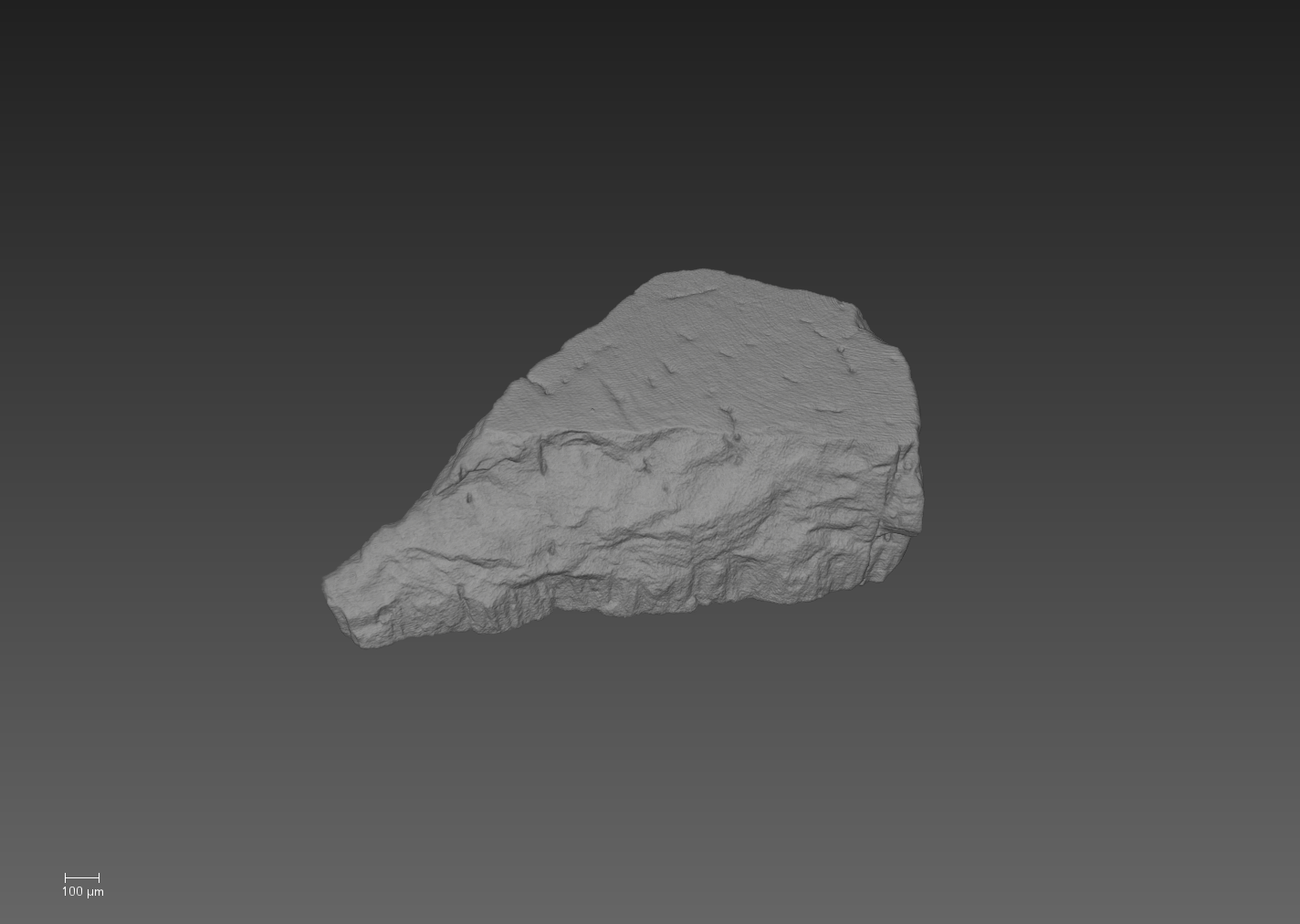

Supplement: Supplementary file 12 — Supplementary Data 12 [file 41467_2023_36405_MOESM12_ESM.zip › Micro_CT_raw_data/Southern_Aepyornis_thick/AD1666/Results/Outer surface.tif]

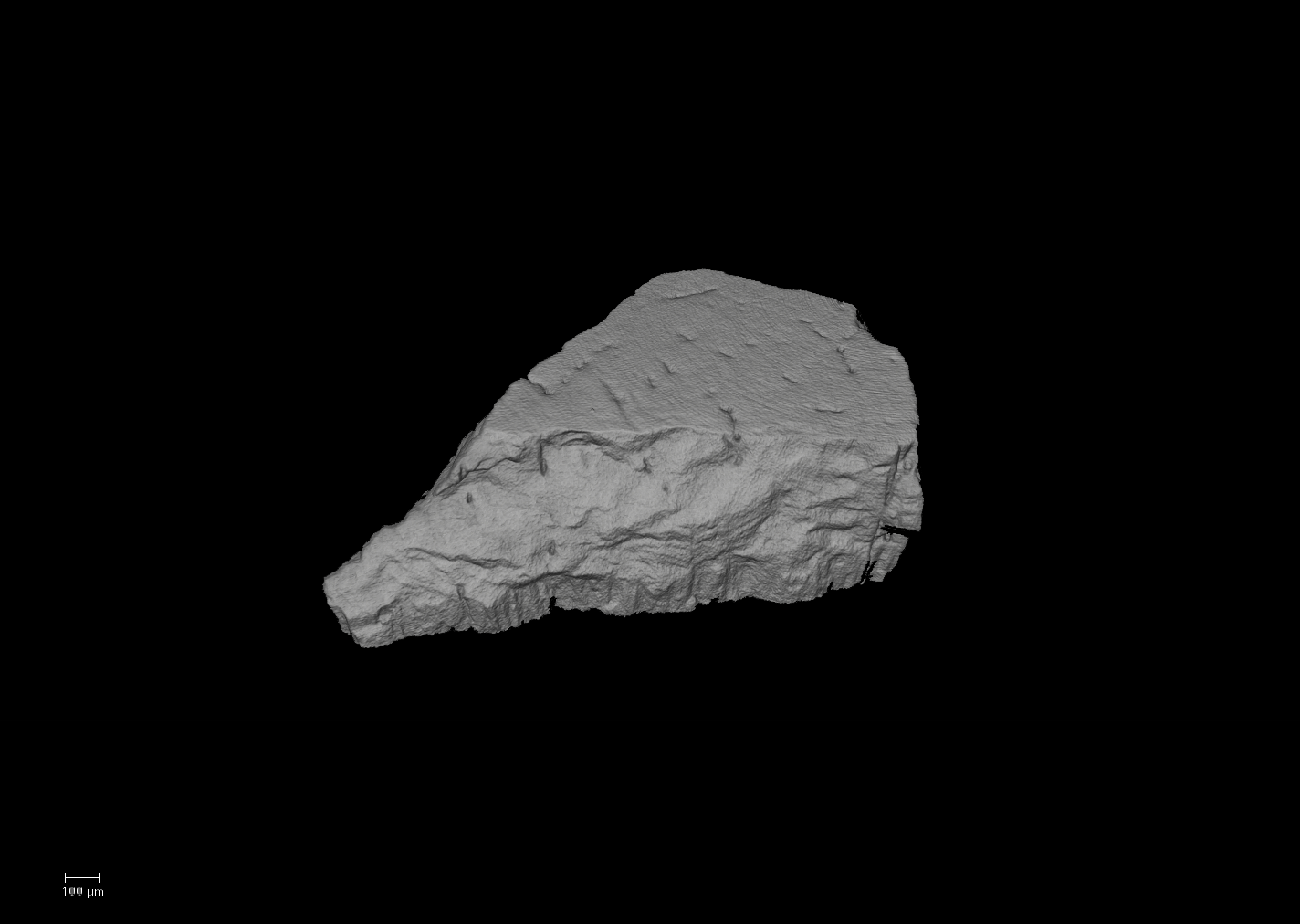

Supplement: Supplementary file 12 — Supplementary Data 12 [file 41467_2023_36405_MOESM12_ESM.zip › Micro_CT_raw_data/Southern_Aepyornis_thick/AD1666/Results/Outer surface2.tif]

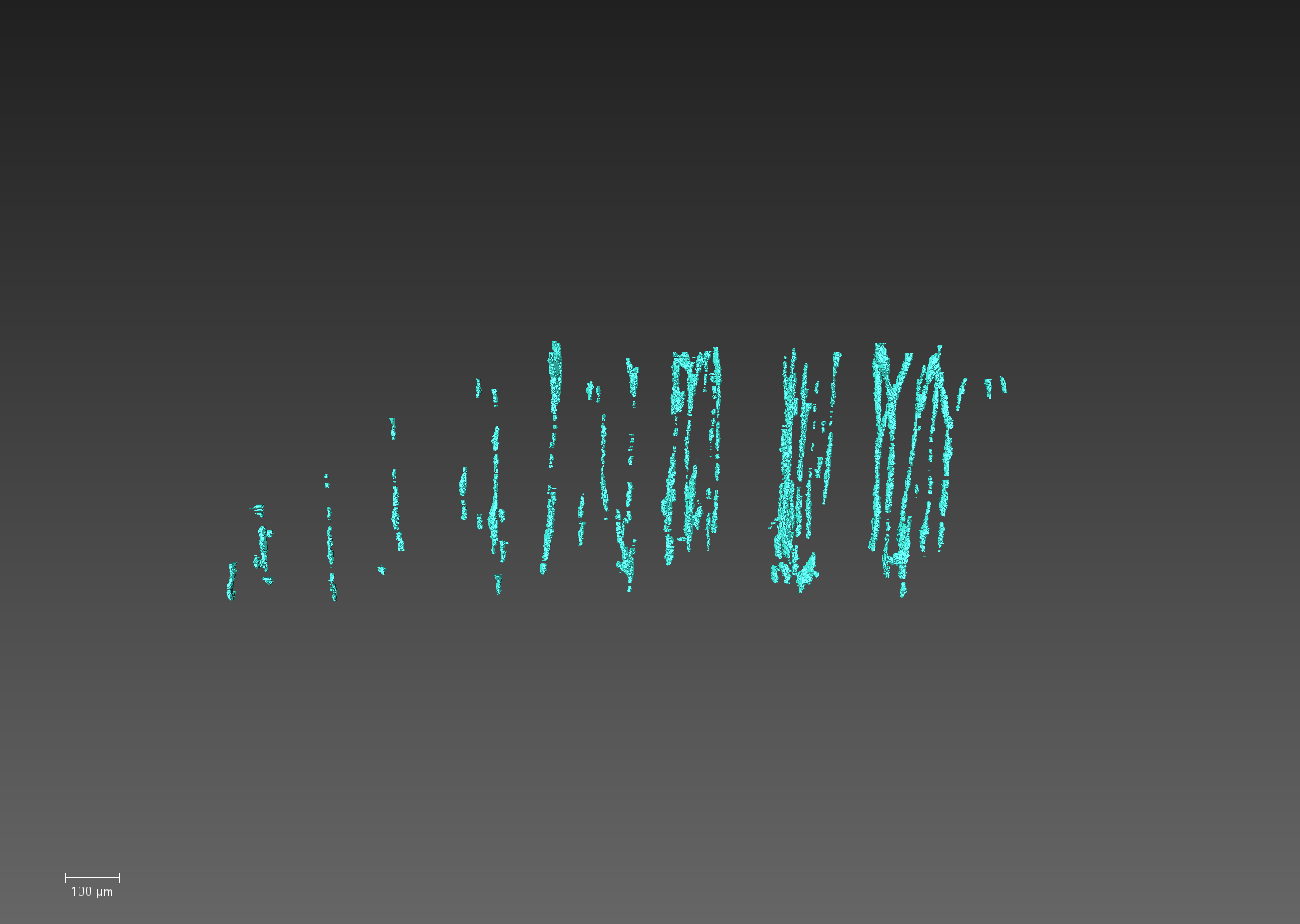

Supplement: Supplementary file 12 — Supplementary Data 12 [file 41467_2023_36405_MOESM12_ESM.zip › Micro_CT_raw_data/Southern_Aepyornis_thick/AD1666/Results/Pore structure.tif]

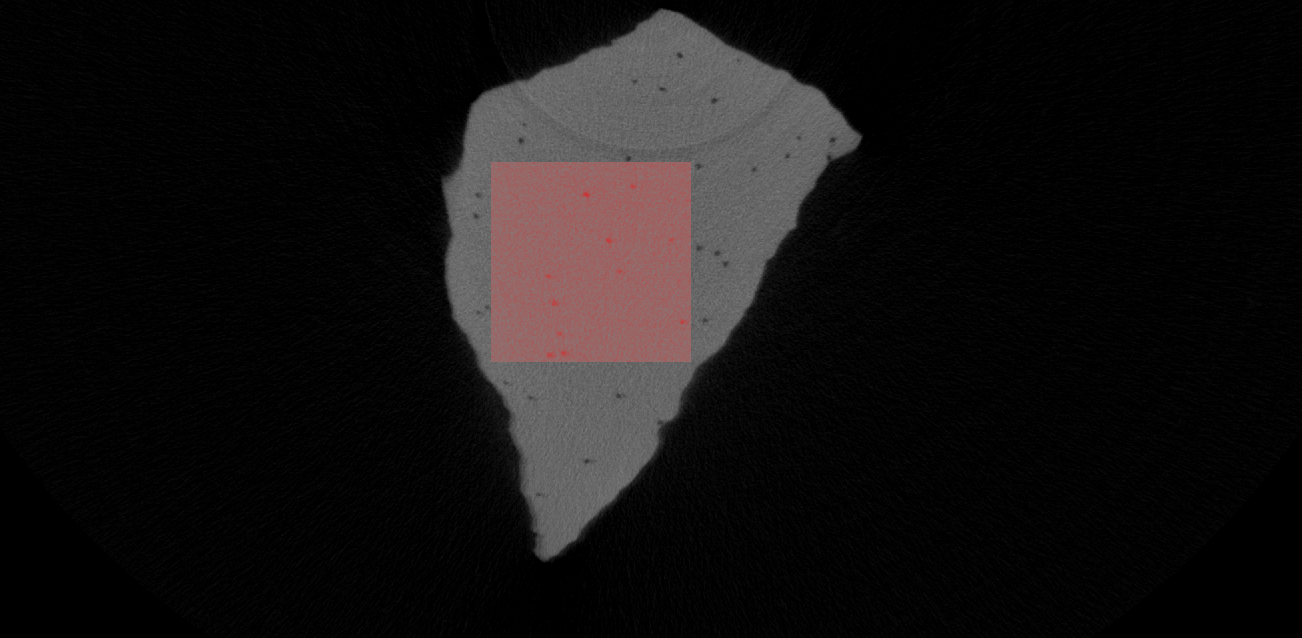

Supplement: Supplementary file 12 — Supplementary Data 12 [file 41467_2023_36405_MOESM12_ESM.zip › Micro_CT_raw_data/Southern_Aepyornis_thick/AD1666/Results/ROI Selection.tif]

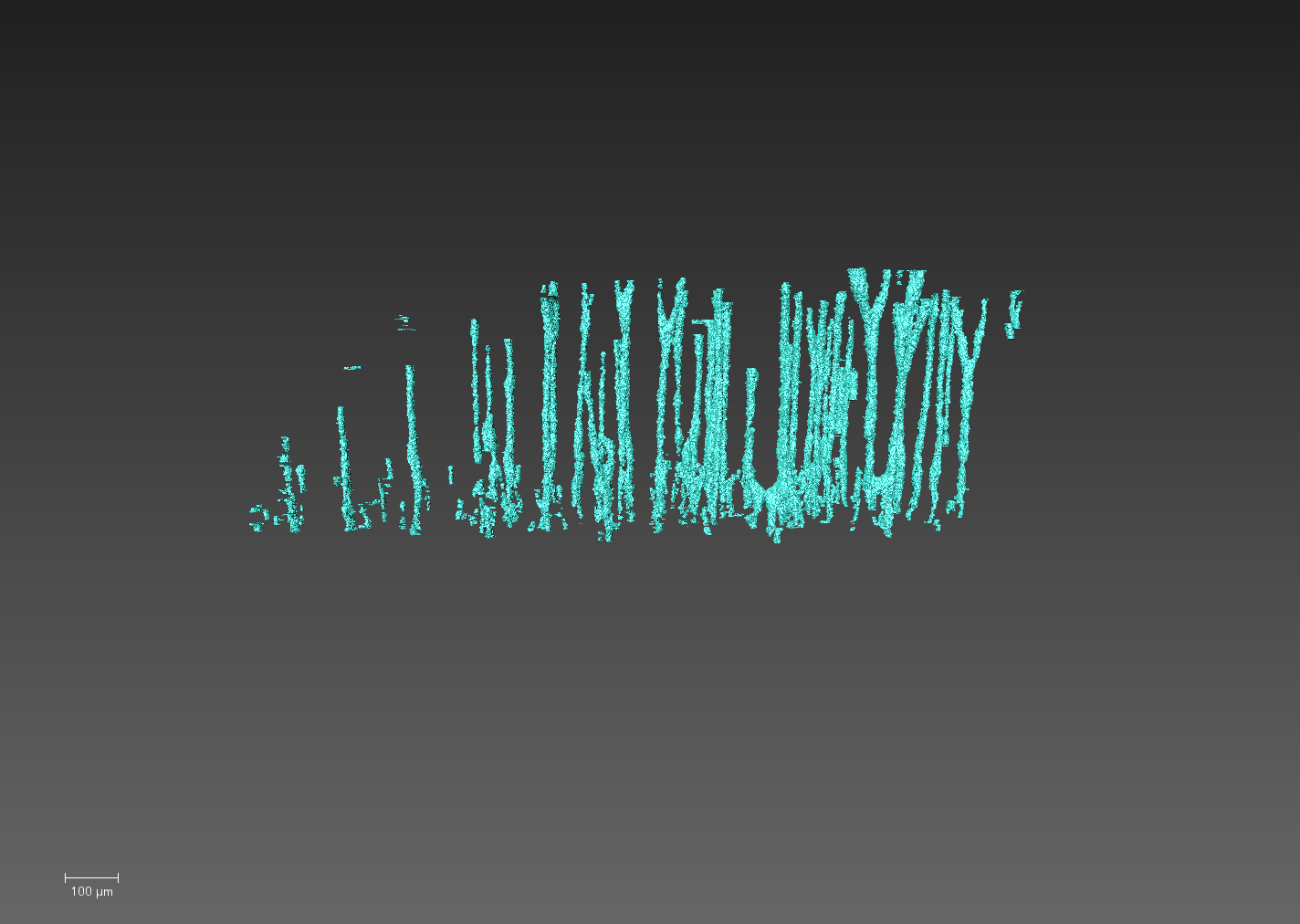

Supplement: Supplementary file 12 — Supplementary Data 12 [file 41467_2023_36405_MOESM12_ESM.zip › Micro_CT_raw_data/Southern_Aepyornis_thick/AD1666/Results/Pore structure3.tif]

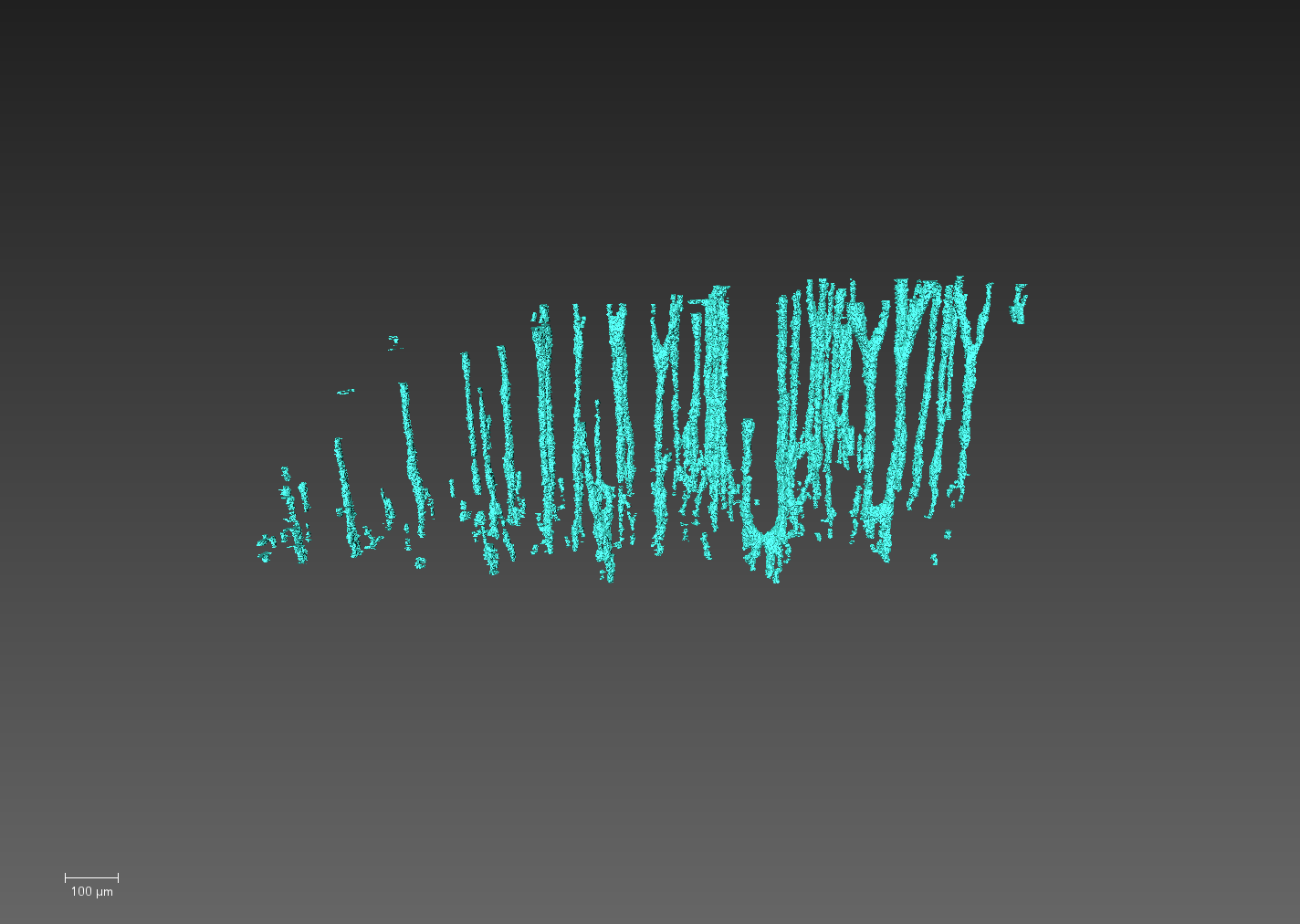

Supplement: Supplementary file 12 — Supplementary Data 12 [file 41467_2023_36405_MOESM12_ESM.zip › Micro_CT_raw_data/Southern_Aepyornis_thick/AD1666/Results/Pore structure2.tif]
