# Supplementary material for: Molecular exploration of fossil eggshell uncovers hidden lineage of giant extinct bird
Source: Nat Commun. 2023 Feb 28;14:914. doi: 10.1038/s41467-023-36405-3 (PMC9974994; doi:10.1038/s41467-023-36405-3)
Supplement: Supplementary file 14 — Reporting Summary [file 41467_2023_36405_MOESM14_ESM.pdf]

nature portfolio

Corresponding author(s): Alicia Grealy

Last updated by author(s): Jan 20, 2023

Reporting Summary

Nature Portfolio wishes to improve the reproducibility of the work that we publish. This form provides structure for consistency and transparency in reporting. For further information on Nature Portfolio policies, see our Editorial Policies and the Editorial Policy Checklist.

Statistics

For all statistical analyses, confirm that the following items are present in the figure legend, table legend, main text, or Methods section.

n/a	Confirmed	
☐	☒	The exact sample size (*n*) for each experimental group/condition, given as a discrete number and unit of measurement
☐	☒	A statement on whether measurements were taken from distinct samples or whether the same sample was measured repeatedly
☐	☒	The statistical test(s) used AND whether they are one- or two-sided<br>*Only common tests should be described solely by name; describe more complex techniques in the Methods section.*
☒	☐	A description of all covariates tested
☐	☒	A description of any assumptions or corrections, such as tests of normality and adjustment for multiple comparisons
☐	☒	A full description of the statistical parameters including central tendency (e.g. means) or other basic estimates (e.g. regression coefficient) AND variation (e.g. standard deviation) or associated estimates of uncertainty (e.g. confidence intervals)
☐	☒	For null hypothesis testing, the test statistic (e.g. *F*, *t*, *r*) with confidence intervals, effect sizes, degrees of freedom and *P* value noted<br>*Give P values as exact values whenever suitable.*
☐	☒	For Bayesian analysis, information on the choice of priors and Markov chain Monte Carlo settings
☒	☐	For hierarchical and complex designs, identification of the appropriate level for tests and full reporting of outcomes
☒	☐	Estimates of effect sizes (e.g. Cohen's *d*, Pearson's *r*), indicating how they were calculated

Our web collection on statistics for biologists contains articles on many of the points above.

Software and code

Policy information about availability of computer code

Data collection	No software was used for data collection. Code used for ancestral state reconstruction is available as described in the "Code availability statement" in the main text (Supplementary Code 1).

Data analysis	Details (parameters used, test statistics, etc.) of software and code used in data analysis are provided in the Supplementary Information as well as DataDryad (https://doi.org/10.5061/dryad.3j9kd51nc); software used is summarised below:<br><br>Statistical analysis: PAST v. 3.11 (Hammer 2001); R v. 4.2.0 and v1.3.1093 (R Core Team, 2022)<br>Radiocarbon dating: CALIB v.7.1 and SHcal13 (Stuiver and Reimer, 1993; Hogg et al. 2013)<br>Mitochondrial genome reconstruction: USEARCH v.8 (Edgar, 2010); Geneious v. 8.1.6 and 10.0.5 (Kearse et al. 2012); BLAST 2.2.30+ (Altschul et al., 1990); MEGAN v.4.70.4 (Huson et al., 2007); Picard Tools v.2.10.3 (broadinstitute.github.io/picard/); mapDamage 2.0.06 (Ginolhac et al., 2011; Jonsson et al., 2013)<br>Phylogenetic inference: MAFFT v.7.308 (Katoh et al., 2002); MUSCLE v. 3.8.425 (Edgar, 2004); ARWEN v1.2 (Laslett and Canback, 2008); Gblocks v0.9b (Castresana, 2000; Talavera and Castresana, 2007); PAUP v.4a150 (Swofford, 2003); MrBayes v3.2.6 (Huelsenbeck and Ronquist, 2001); CIPRES v. 3.3 (Miller et al., 2010); TreeStat v.1.2 (Rambaut and Drummond, 2007); jModelTest3.7 (Guindon and Gascuel, 2003; Darriba et al., 2012, Posada and Crandall, 1998); RAxML v1.5 (Stamatakis, 2014); Tracer v1.6.1 (Rambaut et al., 2003); MEGA v6.06 (Kumar et al., 2013; Stecher et al., 2020); SpeciesDelimitation v.1.03 (Masters et al., 2010)<br>Molecular dating: MCMCtree in PAML v4.4d (Yang et al., 2006-2007); FigTree v1.4.4 (Rambaut, 2006)<br>Protein analysis: PEAKS v.8.5 (Zhang et al., 2012); ColabFold AlphaFold2 (Steinegger et al. n. d.; Jumper et al. 2021)<br>micro-CT: Bruker CTAn v1.16.4.1+ (SkyScan 2003-2011); FEI Avizo Fire v8.1.1 (Konrad-Zeuse-Zentrum Berlin 1995-2014)<br>Stable isotope analysis: ISOERROR v.1.04 (Phillips and Gregg, 2001)<br>Ancestral state reconstruction: see Supplementary Code 1; Phytools v1.2-0 (Revell, 2012); StableTraits v1.4 (Elliot and Mooers, 2014)

For manuscripts utilizing custom algorithms or software that are central to the research but not yet described in published literature, software must be made available to editors and reviewers. We strongly encourage code deposition in a community repository (e.g. GitHub). See the Nature Portfolio guidelines for submitting code & software for further information.

Data

Policy information about availability of data

All manuscripts must include a data availability statement. This statement should provide the following information, where applicable:

- Accession codes, unique identifiers, or web links for publicly available datasets
- A description of any restrictions on data availability
- For clinical datasets or third party data, please ensure that the statement adheres to our policy

All supplementary methods and data related to this article can be found alongside the online version of the article and on DataDryad (https://doi.org/10.5061/dryad.3j9kd51nc). Source data for Figures 1, 2, 3, and 4 can be found in Supplementary Data 1-4, 6 and 10-11, 8 and 12, and 9. Mitochondrial genome sequences for the studied specimens can be found on GenBank (ncbi.nlm.nih.gov; accessions OP413790-OP413810, KJ749824, KJ749825, AP014697, AP014698). Short read data has been deposited on NCBI's Short Read Archive (BioProject ID PRJNA880433, https://www.ncbi.nlm.nih.gov/sra/PRJNA880433). Proteomics data has been deposited on ProteomeXchange under the dataset identifier PXD035725. Eggshell specimens are currently housed at the University of Colorado (Boulder) and Curtin University (Western Australia), and will be donated to the University of Colorado Museum in 2023; in the meantime, requests for fossil material should be directed to Giff Miller (gmiller@colorado.edu). Correspondence and requests for other materials should be addressed to AG (alicia.grealy@uqconnect.edu.au).

Human research participants

Policy information about studies involving human research participants and Sex and Gender in Research.

Reporting on sex and gender	N/A
Population characteristics	N/A
Recruitment	N/A
Ethics oversight	N/A

Note that full information on the approval of the study protocol must also be provided in the manuscript.

Field-specific reporting

Please select the one below that is the best fit for your research. If you are not sure, read the appropriate sections before making your selection.

☐ Life sciences ☐ Behavioural & social sciences ☒ Ecological, evolutionary & environmental sciences

For a reference copy of the document with all sections, see nature.com/documents/nr-reporting-summary-flat.pdf

Ecological, evolutionary & environmental sciences study design

All studies must disclose on these points even when the disclosure is negative.

Study description	Hundreds of fossil eggshell (n=965) were collected from localities across Madagascar (n=291). The thickness of eggshell fragments was measured to characterise eggshell morphotypes. A subset of eggshells from each morphotype were characterised molecularly in terms of genetics, stable isotope composition, micro morphology, and protein. A subset of eggshells were radiocarbon dated (n=21).

Stable isotope analysis was performed on 335 eggshell fragments representing each morphotype across 206 locations. The mitochondrial DNA from 21 eggshell fragments (>4 from each morphotype across several locations) was sequenced and used for phylogenetic inference alongside previously published mitochondrial genomes from skeletal remains (n=6). The protein from 12 eggshell fragments (4 from each morphotype across several locations) were sequenced. 22 eggshell fragments (>4 from each morphotype across several locations) were micro-CT scanned. Differences in stable isotope composition and micro-morphology were compared between eggshell morphotypes (thickness, i.e., "thin", "medium", "thick") and geographic area (southern, central, and northern Madagascar) with experimental units being eggshell fragments from different localities within a geographic area (to reduce the possibility of sampling more than one eggshell fragment from the same individual, i.e., ensure units were independent). This study was a descriptive, cross-sectional observational study, and did not involve manipulative experimentation or intervention in the design.

Research sample

Fossil eggshell of the extinct elephant birds (Order: Aepyornithiformes) of Madagascar. Eggshell was dated to 1290-6190 years BP. DNA extracted from the eggshell is likely female (maternal origin). Fossil eggshell is contemporaneous with all previously dated bone specimens, and eggshell was collected from deposits in sympatry with skeletal deposits, indicating that the eggshell are likely associated with the same extinct taxa that has been described morphologically from these areas. Large numbers of eggshell was collected across most of the areas that elephant birds were known to inhabit and therefore are a good representation of most populations.

Sampling strategy

Over 960 and elephant bird eggshell fragments were collected from 291 localities across southern, central, and northern Madagascar (where skeletal or eggshell fossils have been observed; collecting sites were chosen based on satellite imagery). Several eggshell fragments from each locality were collected randomly, with most found as surface exposures in recently wind-deflated sand dunes, rocky outcrops and beaches, though some were collected in situ in vertical exposures or incidentally during archaeological excavations. Samples sizes were not chosen but were opportunistically determined. All eggshell fragments were used for thickness measurements.

Samples for DNA extraction were prioritised based on age (youngest first), depositional context (in situ vs exposed), and low levels of amino-acid racemisation as measured by Giff Miller. The same locality was not sampled twice for DNA in order to minimise the chance that two eggshell fragments may have originated from the same egg or female. Samples were also chosen to represent each thickness morphotype and geographic location. No sample size calculation was performed as sample size was largely dictated by how many samples contained preserved DNA.

Eggshell fragments representing each thickness morphotype from each geographic location that were most successful for DNA were used for protein and micro-CT analysis. Four from each group was used (as the minimum for statistical analysis is typically three), but was again dictated by preservation condition. Ideally, the same specimens used for DNA analysis was used for both these subsequent analyses, however, it wasn't always possible (as some had too little material remaining after DNA analysis).

Data collection

Aepyornithiform eggshells were collected during field seasons conducted in 2006 and 2007 with the assistance of Ramilisonina of the Institut de Civilizations Musée d'Art et d'Archéologie, Antananarivo, Madagascar, and Retsihisatse Analamahery, Ambovombe, Madagascar, as well as Dr. Jean-Luc Schwenninger, Oxford University, Dr. John Magee, Australian National University, Canberra, and Steve DeVogel, University of Colorado. Some samples were collected by Kristina Douglass, Alicia Grealy, James Haile and the MAP team in 2014. Samples were placed in plastic-zip lock bags, and those collected by Alicia Grealy and James Haile were collected with gloves. Two eggshell fragments were donated for research by James Hansford. Samples were stored at room temperature. Giff Miller and Simon Clarke collected radiocarbon dates amino acid racemisation, and stable isotope data (see Supplementary Information for details).

Ancient DNA was extracted from eggshell samples by Alicia Grealy in the Trace Advanced Ultra-Clean Environment (TrACE) at Curtin University, WA (Australia). Eggshell thickness measurements were also collected by Alicia Grealy and performed in this facility. Personal protective equipment including coveralls, hairnet, double gloves, face mask, eye-wear, and boots were worn to minimise the introduction of further exogenous DNA contamination. All surfaces were decontaminated with a solution of 10% bleach, followed by 70% ethanol as is the standard protocol for contamination avoidance (Willerslev et al., 2005; Knapp et al., 2012). Alicia Grealy also prepared DNA libraries, performed hybridisation capture experiments, and sequenced the DNA. in-house (Curtin University). Genetic experiments were manually recorded in laboratory notebooks, which are also saved digitally (available upon request).

Diana Patalwala performed the micro-CT and 2D/3D analyses at the National Imaging Facility (Centre for Microscopy, Characterisation and Analysis, University of Western Australia). Beatrice Demarchi, Matthew Collins, Meaghan Mackie, Jorune Sakalauskaite, and Josefin Stiller performed ancient protein experiments at the Archaeobiomics Laboratory (University of Turin, Italy) and Novo Nordisk Centre for Protein Research (Copenhagen, Denmark).

Timing and spatial scale

Eggshell samples were collected over several field seasons by several researchers between 2006 and 2014. Since the eggshell is ca. 1000 years old (based on 21 new radiocarbon dates), the frequency and periodicity of collecting has little bearing on the outcomes of the study. Samples originate from across the entire length of Madagascar (ca. 1500 km), mostly from the far north and far south, with few samples available from central Madagascar.

Data exclusions

Although five eggshell specimens thinner than 0.7 mm were collected in northern Madagascar, these have not been confirmed to be ratite eggshells and were not available for molecular analysis. They were excluded from estimates of mean eggshell thickness.

DNA extraction was attempted on hundreds of eggshell fragments; however, only those that contained amplifiable avian mitochondrial DNA (n=35) were sequenced. Of these, only samples where more than half the mitochondrial genome could be reconstructed were used for phylogenetic analysis (n=21). For molecular dating, only the two best (most complete) eggshell genomes from each genus were used so that elephant birds were not over-represented among the remaining palaeognathae.

Reproducibility

Multiple DNA extractions and library preparations were performed for several samples (two were performed three times), showing that the data used for downstream analyses were repeatable (Supplementary Data 4). Samples that failed to yield DNA tended to fail repeatedly (though the number of attempts was not quantified); those that did yield DNA were consistently successful, however, library preparations were not made from replicate DNA extractions for most samples. As eggshell is very thick, most samples were

	not used in their entirety and experiments would be available to be repeated in the future. DNA has also been frozen for long-term storage. DNA and stable isotope results are also consistent with previous independent studies. DNA damage profiles were examined to assess the authenticity of ancient DNA (see Supplementary Information). Tissue-free extraction controls and DNA-free library controls were performed for every batch of extractions or library preparation, and were carried through to sequencing, in order to assess contamination.<br><br>Stable Isotope analysis, micro-CT analysis, and protein analysis were performed once on independent biological samples.
Randomization	Our study did not contain any manipulative experiments requiring randomisation. Samples carried through for analysis relied on DNA preservation which was largely unpredictable and thus samples were random but necessarily biased by DNA preservation / taphonomy. Samples were extracted randomly in batches of 11 (with one extraction control), and libraries were prepared on independent occasions over the course of several years.
Blinding	Eggshell specimens were allocated a laboratory ID linked to its metadata which was not visible during laboratory experiments or data analysis. Metadata was linked to data after phylogenetic analysis.

Did the study involve field work? ☒ Yes ☐ No

Field work, collection and transport

Field conditions	Field work was conducted during Madagascar's dry season (April to October). As sites are palaeontological the field conditions were largely irrelevant as environmental effects on the samples span over 1000 years.
Location	Specimens originated from 291 localities across Madagascar (these are detailed in Supplementary Data 2). Elevation was essentially 0 m as specimens were collected from beach deposits (except two from central Madagascar where the elevation is ca. 1500 m).
Access & import/export	Research permissions were granted by the Ministère de l'Enseignement Supérieur et de la Recherche Scientifique, Autorisation Numéro 128/13-MESupReS/SG/DGRP and by the Centre de Documentation et de Recherche sur l'Art et les Traditions Orales Malgaches (CEDRATOM), under the auspices of the Memorandum of Understanding between the University of Toliara, under the direction of Dr. Barthélémy Manjakahery, Director of the CEDRATOM, and Yale University, under the direction of Dr. Roderick McIntosh, Professor of Anthropology. Research permissions were also granted to Gifford Miller (University of Colorado, Boulder) by Director Jean-Aimé Rakotoarisoa of L'Institut de Civilisations – Musée d'Art et d'Archéologie de l'Université d'Antananarivo (2006-2007). Local permission to carry out archaeological research was granted by the Office du Maire, Commune de Befandefa and by the Chefs de Fokontany of Andavadoaka, Nosy Ve, Antsaragnagnangy, Lamboara, Ampasilava and Salary. Permits for the export of materials for the purposes of laboratory analysis were granted by the Secretariat Général of the Ministère de l'Artisanat de la Culture et des Patrimoines, Direction Régionale de la Culture et du Patrimoine Atsimo Andrefana, Visas de Sorties Numéro 09/06- MCP/SG/DRCP.AA; Numéro 05/14-MACP/SG/DRCP.AA; Numéro 08/14- MACP/SG/DRCP.AA in accordance with Avis Numéro 375, 02/02/1978. Permission to import fossils into Australia was granted by import permit IP15012450.
Disturbance	As most eggshell specimens were collected from surface deposits in recently wind-deflated dune systems, negligible disturbance was caused to the environment in collecting samples. Eggshell fragments are extremely plentiful across sites so the resource was not depleted.

Reporting for specific materials, systems and methods

We require information from authors about some types of materials, experimental systems and methods used in many studies. Here, indicate whether each material, system or method listed is relevant to your study. If you are not sure if a list item applies to your research, read the appropriate section before selecting a response.

Materials & experimental systems

n/a	Involved in the study	
☒	☐	Antibodies
☒	☐	Eukaryotic cell lines
☐	☒	Palaeontology and archaeology
☒	☐	Animals and other organisms
☒	☐	Clinical data
☒	☐	Dual use research of concern

Methods

n/a	Involved in the study	
☒	☐	ChIP-seq
☒	☐	Flow cytometry
☒	☐	MRI-based neuroimaging

Palaeontology and Archaeology

Specimen provenance	Specimens originated from across Madagascar (see Supplementary Data 2 for a full list of localities including GPS co-ordinates). Research permissions were granted by the Ministère de l'Enseignement Supérieur et de la Recherche Scientifique, Autorisation Numéro 128/13-MESupReS/SG/DGRP and by the Centre de Documentation et de Recherche sur l'Art et les Traditions Orales

Malgaches (CEDRATOM), under the auspices of the Memorandum of Understanding between the University of Toliara, under the direction of Dr. Barthélémy Manjakahery, Director of the CEDRATOM, and Yale University, under the direction of Dr. Roderick McIntosh, Professor of Anthropology. Research permissions were also granted to Gifford Miller (University of Colorado, Boulder) by Director Jean-Aimé Rakotoarisoa of L'Institut de Civilisations – Musée d'Art et d'Archéologie de l'Université d'Antananarivo (2006-2007). Local permission to carry out archaeological research was granted by the Office du Maire, Commune de Befandefa and by the Chefs de Fokontany of Andavadoaka, Nosy Ve, Antsaragnagnangy, Lamboara, Ampasilava and Salary. Permits for the export of materials for the purposes of laboratory analysis were granted by the Secretariat Général of the Ministère de l'Artisanat de la Culture et des Patrimoines, Direction Régionale de la Culture et du Patrimoine Atsimo Andrefana, Visas de Sorties Numéro 09/06- MCP/SG/DRCP.AA; Numéro 05/14-MACP/SG/DRCP.AA; Numéro 08/14- MACP/SG/DRCP.AA in accordance with Avis Numéro 375, 02/02/1978. Permission to import fossils into Australia was granted by import permit IP15012450.

Specimen deposition

Specimens and DNA extractions are housed within the Trace Advanced Ultra-Clean Environment (TrACE) at Curtin University, WA (Australia) in conditions of controlled temperature and humidity. Splits of some 300 samples are housed at the University Museum, Antananarivo and the University of Colorado (Boulder). Those at the University of Colorado (Boulder) and will be donated to the University of Colorado Museum in 2023; in the meantime, requests for fossil material should be directed to Giff Miller (gmiller@colorado.edu).

Dating methods

Eggshell samples for radiocarbon dating were mechanically cleaned then reduced by 50% with the stoichiometric addition of 2N HCl in vacuo. Cleaned fragments were converted to graphite at the INSTAAR Laboratory for AMS Radiocarbon Preparation and Research (NSRL) before measurement by Accelerator Mass Spectrometry at the Keck Carbon Cycle AMS Laboratory at the UC Irvine (KCCAMS). Conventional radiocarbon ages have been calibrated using CALIB v.7.1 and SHcal13 (Stuiver and Reimer, 1993; Hogg et al. 2013).

☒ Tick this box to confirm that the raw and calibrated dates are available in the paper or in Supplementary Information.

Ethics oversight

No ethical approval was required for the collection of palaeontological material beyond the permissions granted above. No humans or live animals were the subject of this research.

Note that full information on the approval of the study protocol must also be provided in the manuscript.
